# Differential expression of genes in olive leaves and buds of ON- versus OFF-crop trees

**DOI:** 10.1038/s41598-020-72895-7

**Published:** 2020-09-25

**Authors:** Ebrahim Dastkar, Ali Soleimani, Hossein Jafary, Juan de Dios Alche, Abbas Bahari, Mehrshad Zeinalabedini, Seyed Alireza Salami

**Affiliations:** 1 Department of Horticulture, Faculty of Agriculture, University of Zanjan, Zanjan, Iran; 2 Iranian Research Institute of Plant Protection, Agricultural Research, Education and Extension Organization (AREEO), Tehran, Iran; 3 Plant Reproductive Biology and Advanced Microscopy Laboratory, Department of Biotechnology, Cell and Molecular Biology of Plants, Estación Experimental del Zaidín (CSIC), Granada, Spain; 4 Research Institute of Modern Biological Techniques (RIMBT), University of Zanjan, Zanjan, Iran; 5 Department of Systems and Synthetic Biology, Agricultural Biotechnology Research Institute of Iran (ABRII), Agricultural Research, Education and Extension Organization, Karaj, Iran; 6 Faculty of Agricultural Science and Engineering, University of Tehran, Tehran, Iran

**Keywords:** Biotechnology, Plant biotechnology

## Abstract

Alternate bearing (AB) refers to the tendency of trees to have an irregular crop load from 1 year (ON) to the next year (OFF). Despite its economic importance, it is not fully understood how gene networks and their related metabolic pathways may influence the irregular bearing in olive trees. To unravel molecular mechanisms of this phenomenon in olive (cv. Conservalia), the whole transcriptome of leaves and buds from ON and OFF-trees was sequenced using Illumina next generation sequencing approach. The results indicated that expressed transcripts were involved in metabolism of carbohydrates, polyamins, phytohormones and polyphenol oxidase (POD) related to antioxidant system. Expression of POD was increased in leaf samples of ON- versus OFF-trees. The expression pattern of the greater number of genes was changed more in buds than in leaves. Up-regulation of gene homologues to the majority of enzymes that were involved in photorespiration metabolism pathway in buds of ON-trees was remarkable that may support the hypotheses of an increase in photorespiratory metabolism in these samples. The results indicated changes in expression pattern of homologous to those taking part of abscisic acid and cytokinin synthesis which are connected to photorespiration. Our data did not confirm expression of homologue (s) to those of chlorogenic acid metabolism, which has been addressed earlier that have a probable role in biennial bearing in olive. Current findings provide new candidate genes for further functional analysis, gene cloning and exploring of molecular basses of AB in olive.

## Introduction

Alternate bearing (AB) commonly termed as “Biennial bearing” or “irregular bearing” is a wide spread phenomenon occurring in many fruit trees such as pistachio, apple, citrus, olive, and mango by which cycle of heavy yield (ON-year) in one year is followed by a light yield (OFF-year) in the next year^1,2^. The olive tree (*Olea europaea* L.) is one of the most important Mediterranean evergreen trees prone to severe AB, particularly occurring in some genotypes^3^. As a result, fruit production in olive orchards may switch between 30 to 5 tons/ha within ON- versus OFF-years. The problems caused by AB in this valuable industrial crop are of great economic importance^4^. Many attempts have been made to address this phenomenon; however, the main reason(s) of irregular bearing is still not fully understood. Several parameters have been proposed to describe the intensity and the synchrony related to biennial bearing in trees and how the developing crop influences the following year’s return bloom and yield, but there has been rare deep insight into the molecular aspects of such a complicated phenomenon in olive trees^5,6^. Generally, occurrence of abiotic and biotic stresses, delayed harvest, management disciplines in orchards along with the genetic background of an olive cultivar are reported as important factors which affect fruit load and consequent AB in olive^4,7^. The physiological bases of AB is not very clear, however it is assumed that periodicity in the bearing is an adaptation mechanism of the tree to prevent depletion of its nutrients and reserves^8^. In olive trees, the fruits are developed on well-lignified shoots from growth of the previous year, and flower induction will begin at the time of pit hardening of the developing fruits of the current year and continue for about three months. Specific metabolic pathways are affected by the presence of fruit on the tree at this time and consequently the flower bud induction is influenced by the regulatory nature of the signals produced by the developing embryos^4^. The reverse competition between developing fruits and embryos and flower bud differentiation for the following year’s return bloom was also reported in other fruit trees^9^. The higher content of gibberellins (GAs) and lower content of abscisic acid (ABA) in leaves in the ON- compared to the OFF-years during flowering induction may confirm the effect of endogenous hormones on the flowering and fruit set processes in olive^10,11^. Indeed, previous studies have shown some differences in protein, antioxidant and phenolic contents in the leaves and fruits of olive tree during flower induction times under AB conditions^12,13^.

It is clear that the genetic background, in interaction with environmental factors, has a fundamental role in appearance of AB through activation and repression of endogenous metabolic pathways^11,14^. From this point of view, the flowering process is result of a balance between the presence of promoter and inhibitory signals, together with the differential genes expression, which is affected by these stimuli and occurs in a unified framework^15^. In this scenario, the study of the transcriptome pattern of the olive tissues under ON- and OFF-conditions reveals new deeper insights into better understanding the crucial molecular processes involved in AB, and lead to better understanding of this phenomenon in olive towards decodes of its undiscovered complexity. Yanik et al.^2^ indicated that the miRNA-targeted genes are involved in main biochemical processes such as hormone signal-transduction and carbohydrate metabolism pathways related to AB in olive. Also, Turktas et al.^6^ showed that the nutritional and hormonal status plays a crucial role in the periodicity of the olive tree. Shalom et al.^1^ reported that fruit load in citrus is influenced by many metabolic pathways and the genes responsible for controlling flowering in the buds of ON- and OFF-trees were expressed differentially. A probable hypothesis is that a signal of AB is produced in fruit or leaves which feels the presence of fruit and moves toward the buds. Under the influence of this signal and by changing the regulative and metabolic pathways, the bud is determined to continue either a vegetative or a reproductive growth.

Nowadays, next-generation sequencing (NGS) and other high-throughput sequencing approaches have provided an opportunity to deeply sequence and study the whole transcriptome of the cells and tissues in a very short period of time^16,17^. The RNA-Seq, i.e., sequencing of transcriptome using NGS techniques, is one of the most popular approaches of NGS which enable us to figure out the complete transcriptomics events in biological samples without previous knowledge about their genome sequence^17,18^. So far, the ability of the RNA-Seq technique has been used in numerous studies on resistance to pathogens^18,19^, the metabolomics studies^20^, the physiological stages of fruit and bud development^21,22^, the evaluation of reference genes and genome^16,23,24^ in olive and other fruit trees. It appears that, following perception of the AB signal, a series of physiological, biochemical and molecular events occurs in the buds which depend on fruit load. The goal of the present study was to provide better insight into molecular regulation of global gene networks in buds and leaves of ON and OFF olive trees using Illumina sequencing approach. To the best of our knowledge, this work provides the first report describing the whole transcriptome profile of the olive buds and leaves during the flower induction under biennial bearing conditions. An overview of different metabolism connection networks involved in alternate bearing in olive as well as flowering control genes is also being discussed.

## Results

A total of 325,125,070 clean reads were obtained from eight libraries after filtering. In every sample, an average of 20,320,317 paired-end 150 bp reads was obtained with average Q20 and GC of 97.36% and 45.3%, respectively (Table 1). In total, 512,658 contigs with N50 lengths of 1348 base, average length of 806.45 bases and mean of % GC 41.21 was obtained from de novo assembly analysis. On average about 93% of clean reads of each library were mapped with these contigs.

**Table 1:** Statistics of RNA-Seq and mapping analysis for olive’s leaves and buds transcriptome in ON- and OFF-trees.

Sample name	Paired-end reads	Clean reads	Clean bases	Read length (bp)	Q20 (%)	GC (%)	Mapping %
Leaf ON 1	20489656	40979312	6146896800	150	95.42–97.07	45.69	93.58
Leaf ON 2	20003294	40006588	6000988200	150	95.22–96.78	45.47	93.25
Leaf OFF 1	20185670	40371340	6055701000	150	98.31–97.36	44.78	92.83
Leaf OFF 2	20632407	41264814	6189722100	150	98.24–97.22	45.44	92.89
Bud ON 1	20474324	40948648	6142297200	150	98.29–97.19	45.21	92.55
Bud ON 2	20213825	40427650	6064147500	150	98.32–97.62	45.27	92.46
Bud OFF 1	20087285	40174570	6026185500	150	98.29–97.21	45.38	92.12
Bud OFF 2	20476074	40952148	6142822200	150	98.22–97.07	45.12	92.72
Average	20320316.88	40640633.75	6096095062.50	150	97.36	45.30	92.8
SUM	162562535	325125070	48768760500	–	–	–	–

### Comparing of ON- and OFF-transcriptome profiles

Towards comparing ON- and OFF-trees transcriptome profiles, a set of fragments with differential expression (DE) pattern were studied in leaves and buds.

### Comparison of leaf transcriptome in ON- vs. OFF-trees

**Figure 1:**
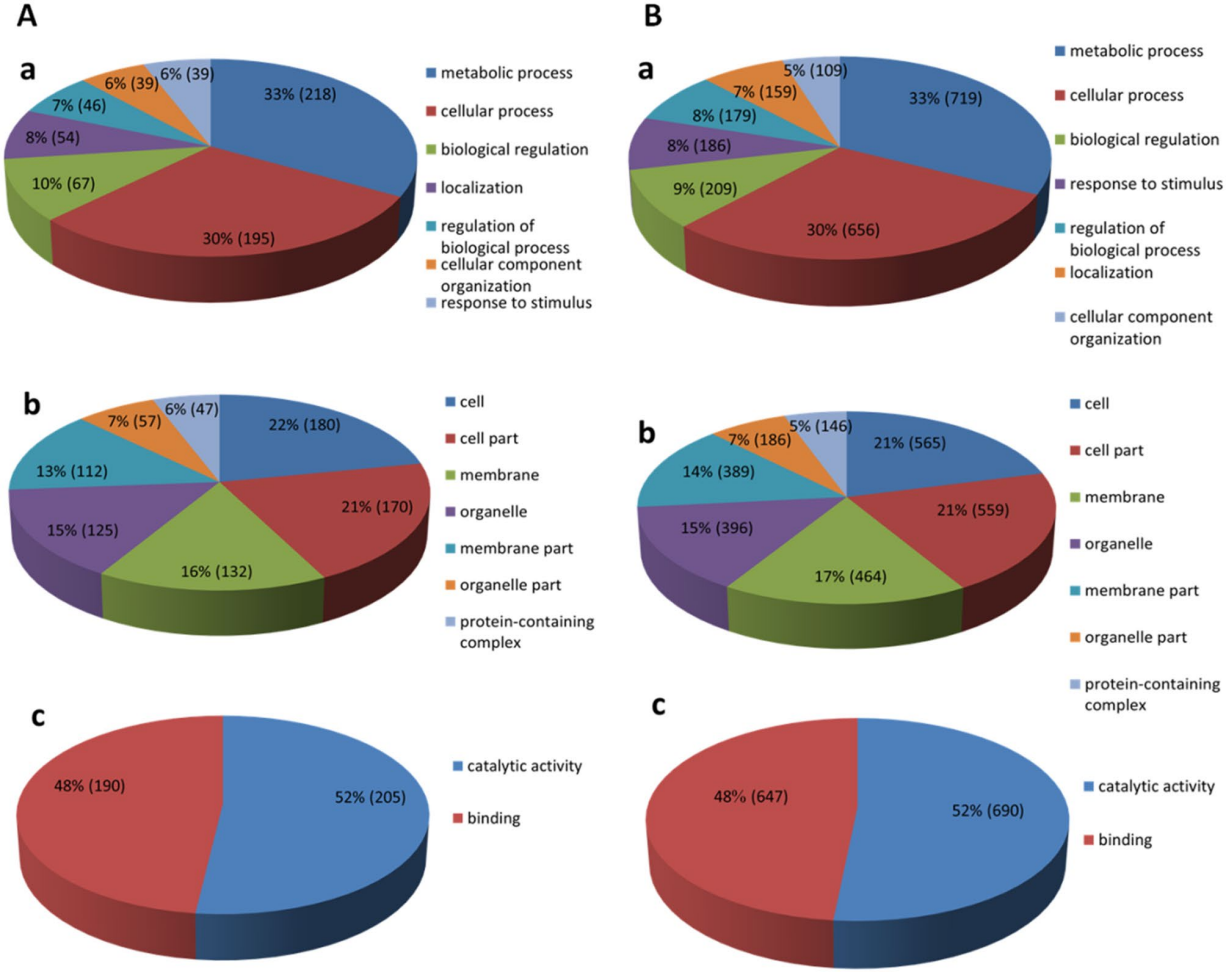
Gene Ontology classification (level 2 GO terms) containing the share of putative transcripts (%) of olive’s leaf (**A**) and bud (**B**) in ON- vs. OFF-trees within the functional categories; biological process (a), cellular component (b) and molecular function (c).

Out of that 454 significantly differentially expressed genes were identified in leaves, 277 were found to be up-regulated and 177 were found to be down-regulated genes in ON- vs. OFF-trees (see Supplementary Table S1). GO analysis of DEGs represented at least one GO ID at database for 368 of them (e-Value ≤ 0.05) (see Supplementary Table S2). Allocation of transcripts to three main groups of biological process, cellular component, and molecular function were shown in Fig. 1A. KEGG enrichment analysis revealed that only three pathways (out of 80 pathways covered) were significantly enriched (corrected P-Value ≤ 0.05) (see Supplementary Table S3). These pathways included photosynthesis, metabolic and fatty acid degradation pathways. Using the results of GO and KEGG, and based on the previous studies on AB in olive and other fruit trees, homologues genes from the photosynthesis, carbohydrates metabolism, phenolic compounds, antioxidant enzymes, polyamines, and phytohormones biosynthesis pathways were studied in more detail (Table 2).

**Table 2:** List of 26 differentially expressed transcripts associated with some biosynthetic pathways and flower-related genes in olive’s leaf samples, ON- vs. OFF-trees.

	Seq-ID	GenBank accession	Description	GO names	log2 fold change	padj*
Photosynthesis and carbohydrates metabolism	TRINITY_DN87930_c2_g2_i8	YP_003359367.1	Ribulose-1,5-bisphosphate carboxylase/oxygenase large subunit (chloroplast)	F:magnesium ion binding; F:monooxygenase activity; C:chloroplast; P:photorespiration; F:ribulose-bisphosphate carboxylase activity; P:reductive pentose-phosphate cycle; P:oxidation-reduction process	3.38	0.00
	TRINITY_DN79063_c1_g1_i9	XP_022872657.1	Long-chain-alcohol oxidase FAO4A-like	C:intracellular; C:integral component of membrane; F:lyase activity; F:long-chain-alcohol oxidase activity; F:flavin adenine dinucleotide binding; P:oxidation-reduction process	9.87	0.04
	TRINITY_DN84614_c6_g1_i3	XP_006492594.1	UDP-glucose 4-epimerase GEPI48	F:UDP-glucose 4-epimerase activity; P:galactose metabolic process	− 7.39	0.02
	TRINITY_DN82831_c0_g1_i8	XP_022881139.1	Probable trehalose-phosphate phosphatase F	F:trehalose-phosphatase activity; P:trehalose biosynthetic process; P:dephosphorylation	8.79	0.03
	TRINITY_DN83157_c0_g11_i1	XP_022871541.1	Probable galactinol--sucrose galactosyltransferase 6 isoform X1	F:galactinol-sucrose galactosyltransferase activity	− 2.99	0.04
	TRINITY_DN82827_c0_g2_i4	XP_022863390.1	Probable galactinol--sucrose galactosyltransferase 6	F:galactinol-sucrose galactosyltransferase activity	24.92	0.00
	TRINITY_DN82882_c2_g2_i16	XP_022872970.1	Probable alpha-amylase 2	F:alpha-amylase activity; F:calcium ion binding; P:carbohydrate metabolic process; F:alpha-amylase activity (releasing maltohexaose)	− 3.14	0.03
	TRINITY_DN82882_c2_g2_i18	XP_022872971.1	Probable alpha-amylase 2	F:alpha-amylase activity; F:calcium ion binding; P:carbohydrate metabolic process; F:alpha-amylase activity (releasing maltohexaose)	− 2.13	0.00
	TRINITY_DN82733_c0_g1_i6	XP_022869919.1	Bidirectional sugar transporter SWEET2	C:plasma membrane; C:integral component of membrane; P:carbohydrate transmembrane transport; F:sugar transmembrane transporter activity	− 9.10	0.02
Photosynthesis and carbohydrates metabolism	TRINITY_DN80957_c1_g1_i2	XP_022861297.1	Expansin-A1 isoform X1	C:extracellular region; C:cell wall; P:plant-type cell wall organization; C:membrane	3.19	0.01
	TRINITY_DN87529_c3_g4_i6	AUB30489.1	Ribosomal protein subunit S3 (mitochondrion)	F:structural constituent of ribosome; C:mitochondrion; C:ribosome; P:translation; F:rRNA binding	2.26	0.02
	TRINITY_DN78435_c0_g5_i3	XP_022871821.1	40S ribosomal protein S9	F:structural constituent of ribosome; F:rRNA binding; C:cytosolic small ribosomal subunit; P:positive regulation of translational fidelity	2.96	0.01
	TRINITY_DN82900_c2_g1_i6	XP_022880916.1	40S ribosomal protein S4-3-like	F:structural constituent of ribosome; C:ribosome; P:translation; F:rRNA binding	8.31	0.00
	TRINITY_DN82174_c3_g2_i7	XP_022887249.1	40S ribosomal protein S14-2	P:ribosomal small subunit assembly; P:maturation of SSU-rRNA from tricistronicrRNA transcript (SSU-rRNA, 5.8S rRNA, LSU-rRNA); F:structural constituent of ribosome; P:translation; C:cytosolic small ribosomal subunit; F:mRNA 5'-UTR binding; F:small ribosomal subunit rRNA binding	3.14	0.00
	TRINITY_DN84461_c2_g1_i3	XP_022897996.1	60S ribosomal protein L31	P:cytoplasmic translation; F:structural constituent of ribosome; C:cytosolic large ribosomal subunit	7.07	0.03
	TRINITY_DN88027_c3_g2_i2	XP_022876819.1	30S ribosomal protein S11, chloroplastic	C:integral component of membrane	2.94	0.00
	TRINITY_DN86837_c1_g2_i1	XP_022841910.1	Ribosomal protein S10	F:ATP binding; C:mitochondrion; C:ribosome; P:ATP synthesis coupled proton transport	2.48	0.00
Phenolic antioxidant compounds and enzymes related genes	TRINITY_DN84914_c2_g2_i13	XP_022862158.1	4-Coumarate--CoA ligase-like 7	P:metabolic process; C:integral component of membrane; F:ligase activity	− 6.85	0.05
	TRINITY_DN88365_c5_g2_i3	XP_022880295.1	Caffeic acid 3-O-methyltransferase-like	F:O-methyltransferase activity; F:S-adenosylmethionine-dependent methyltransferase activity; P:aromatic compound biosynthetic process; P:methylation; F:protein dimerization activity	− 3.58	0.02
	TRINITY_DN81593_c2_g2_i7	XP_022877955.1	Peroxidase 42-like	F:peroxidase activity; C:extracellular region; P:response to oxidative stress; F:heme binding; P:hydrogen peroxide catabolic process; F:metal ion binding; P:oxidation-reduction process; P:cellular oxidant detoxification	5.32	0.00
	TRINITY_DN84441_c0_g1_i6	XP_022893074.1	Pentatricopeptide repeat-containing protein	F:peroxidase activity; P:response to oxidative stress; F:heme binding; P:oxidation-reduction process; P:cellular oxidant detoxification	8.58	0.00
	TRINITY_DN78486_c0_g1_i14	XP_022888361.1	Superoxide dismutase (Fe), chloroplastic	F:superoxide dismutase activity; P:removal of superoxide radicals; F:metal ion binding; P:oxidation-reduction process	− 2.12	0.02
	TRINITY_DN86273_c4_g1_i17	XP_022895137.1	Monodehydroascorbatereductase 4, peroxisomal	C:integral component of membrane; F:monodehydroascorbate reductase (NADH) activity; F:flavin adenine dinucleotide binding; P:oxidation-reduction process	8.68	0.04
Polyamines	TRINITY_DN79434_c1_g2_i1	XP_022844785.1	S-adenosylmethionine decarboxylase proenzyme-like	F:adenosylmethionine decarboxylase activity; C:cytosol; P:S-adenosylmethioninamine biosynthetic process; P:spermine biosynthetic process; P:spermidine biosynthetic process	8.19	0.04
	TRINITY_DN83869_c1_g2_i1	XP_022880237.1	Lysine-specific histone demethylase 1 homolog 1	F:DNA binding; C:nucleus; F:methyltransferase activity; P:histone deacetylation; P:root development; P:histone H3-K4 methylation; acetylspermine:oxygen oxidoreductase (N1-acetylspermidine-forming) activity; F:spermine:oxygen oxidoreductase (spermidine-forming) activity; P:oxidation-reduction process	7.52	0.01
Phytohormones	TRINITY_DN84754_c0_g1_i11	XP_012839200.1	Zeaxanthinepoxidase, chloroplastic-like	F:monooxygenase activity; P:oxidation-reduction process; F:FAD binding	2.08	0.03

*Adjusted p-value.

### Comparison of bud transcriptome in ON- vs. OFF-trees

Out of the total 1809 significantly differentially expressed sequences were identified in buds, 1125 sequences were found to be up-regulated genes, and 684 were found to be down-regulated genes in ON- vs. OFF-trees (see Supplementary Table S4). GO analysis of 1809 DE sequences represented at least one GO ID at database for 1414 transcript (e-Value ≤ 0.05) (see Supplementary Table S5). Transcripts were allocated into three main groups of molecular function, cellular component, and biological process shown in Fig. 1B.

**Figure 2:**
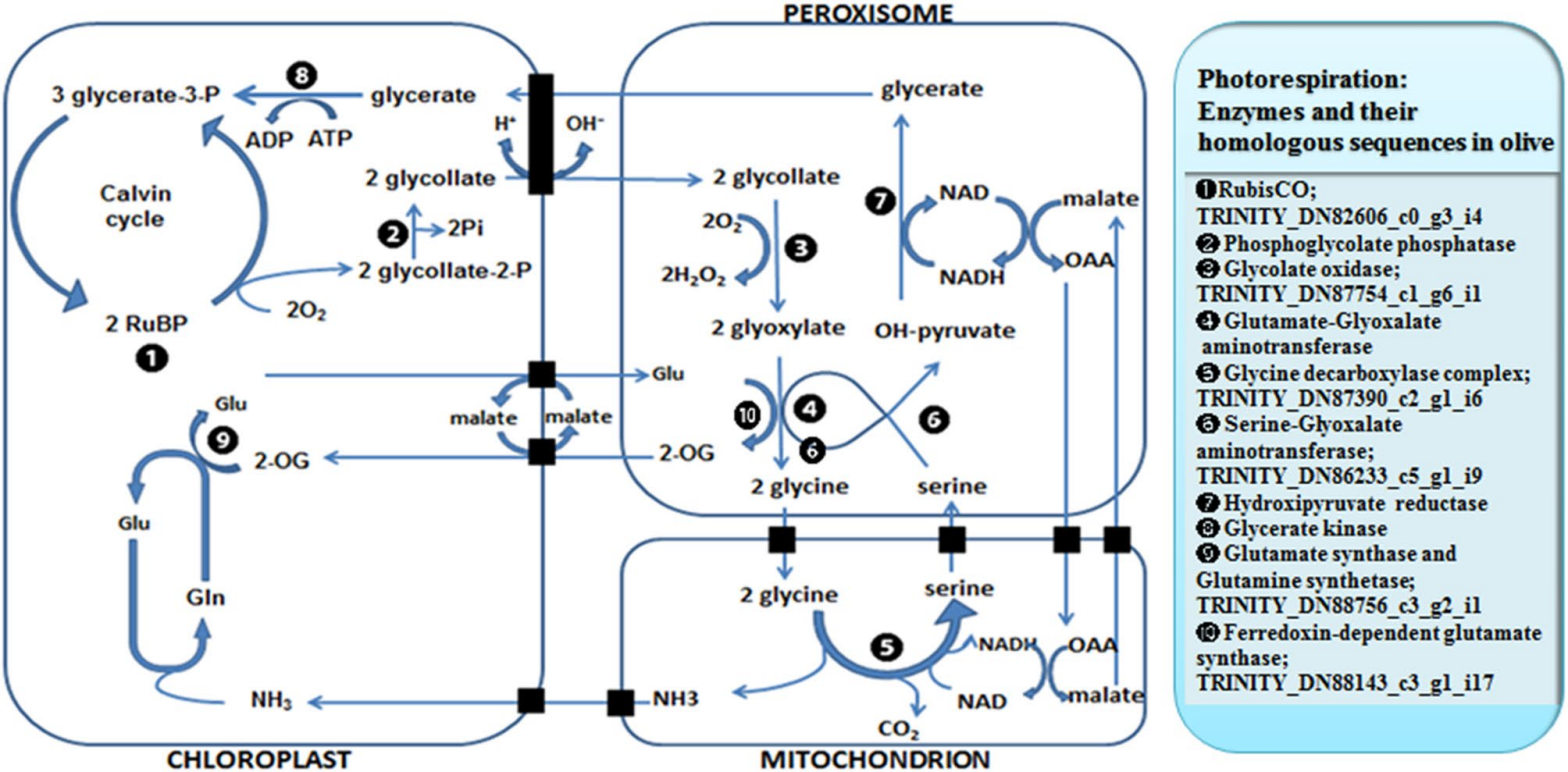
The schematic of the photorespiration metabolism pathway; Genes homologues to the ten enzymes through this pathway, corresponding to number 1–10, showed up-regulation pattern in olive’s bud samples of ON-tree. The homologue sequence identification code of each enzyme resulted from current research, has been noticed. Adapted from Wingler et al.^25^ and Buchanan et al.^26^.

Out of that 1809 DEGs were mapped to canonical pathways of KEGG, 106 were assigned to different KEGG pathways, and 33 pathways were significantly enriched in ON- vs. OFF-trees (corrected P-Value ≤ 0.05) (see Supplementary Table S6). The first five significant pathways were included metabolic pathways, carbon metabolism, and CO_2_ fixation in photosynthetic species, protein processing and photosynthesis. Merging the results of GO and KEGG along with previous reports, homologues genes from the photosynthesis, carbohydrates metabolism, phenolic compounds, antioxidant enzymes, polyamines, phytohormones biosynthesis pathways and flowering control genes were more account (see Supplementary Table S7). We found that the gene homologues of the majority of enzymes that are involved in photorespiration metabolism pathway were up-regulated in buds of ON-trees (Fig. 2).

### Validation of RNA-Seq results using Q-RT-PCR

**Figure 3:**
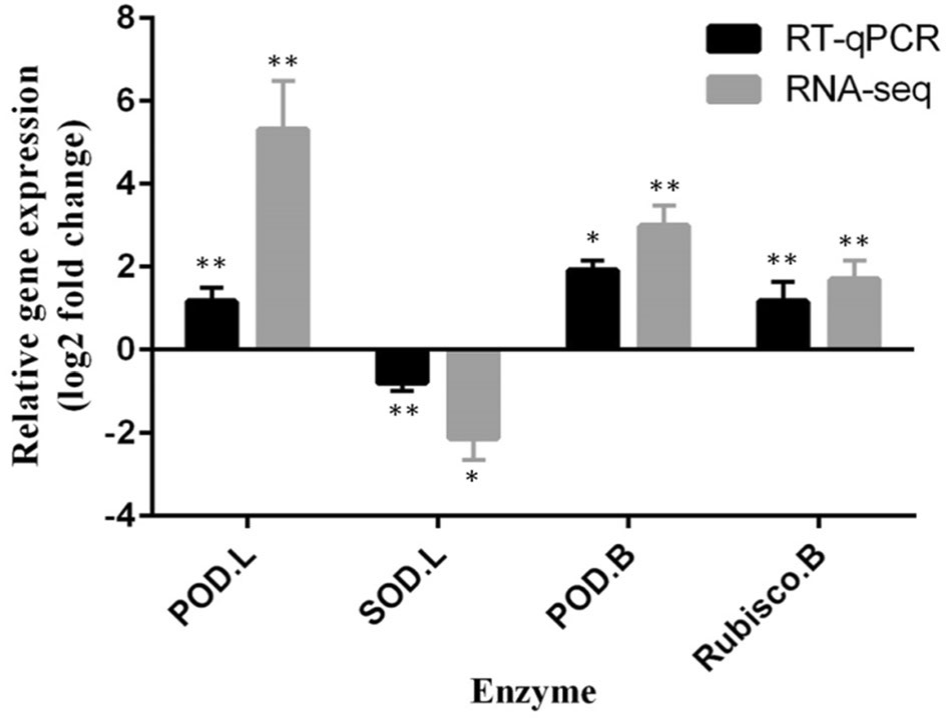
Validation of RNA-seq results by Q-RT-PCR reaction regarding the relative expression of: peroxidase (POD.L) and (POD.B) in leaf and bud samples, respectively, and superoxide dismutase (SOD.L) in leaf and Ribulose-1,5-bisphosphate carboxylase/oxygenase (rubisco.B) in bud samples of olive under alternate bearing condition (ON- vs. OFF-tree). * and **: statistical significance at p ≤ 0.05 and p ≤ 0.01 respectively.

The relative expression of four selected sequences of RNA-Seq data, were confirmed by Q-RT-PCR matched to those of the RNA-Seq (Fig. 3). In leaves, POD homologue was up-regulated while SOD homologue was down-regulated. In buds, POD and rubisco genes homologous both were up-regulated in ON vs. OFF-trees. comparative conditions.

**Figure 4:**
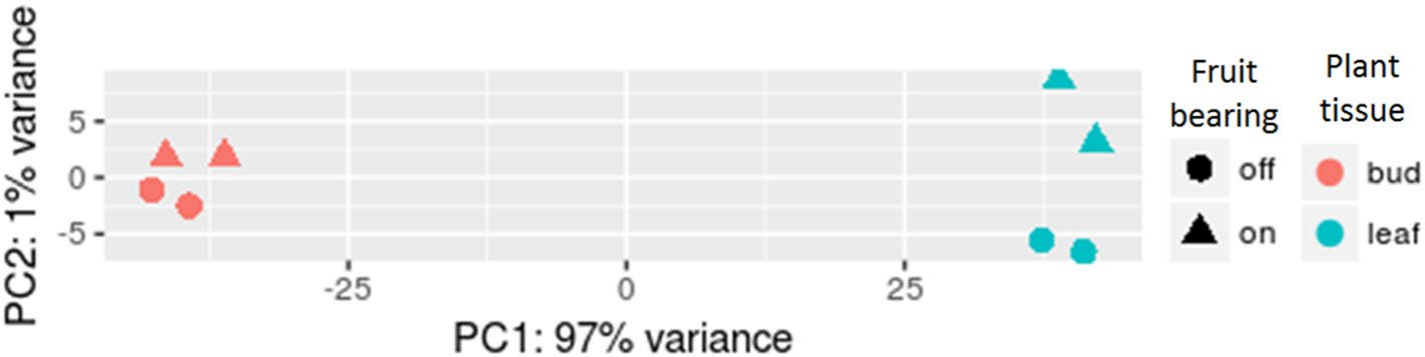
Principal component analysis of global expression profiles in olive’s leaf and bud samples in ON- and OFF-trees.

Quantification of nine housekeeping genes homologous including cyclophilin, 18 s rRNA, GAPDH, 25 s rRNA, ubiquitin (UBQ), beta-actin, RuBP, alpha-tubulin and beta-tubulin showed, that none of them had significant DE in any of compared groups. Prior to DE analysis, the Principal Component Analysis (PCA) was done to explore the similarity of biological replicates. The result indicated the accuracy of sample selection, and that the genetic distance between replications was not significant (Fig. 4).

### Biochemical parameters

**Figure 5:**
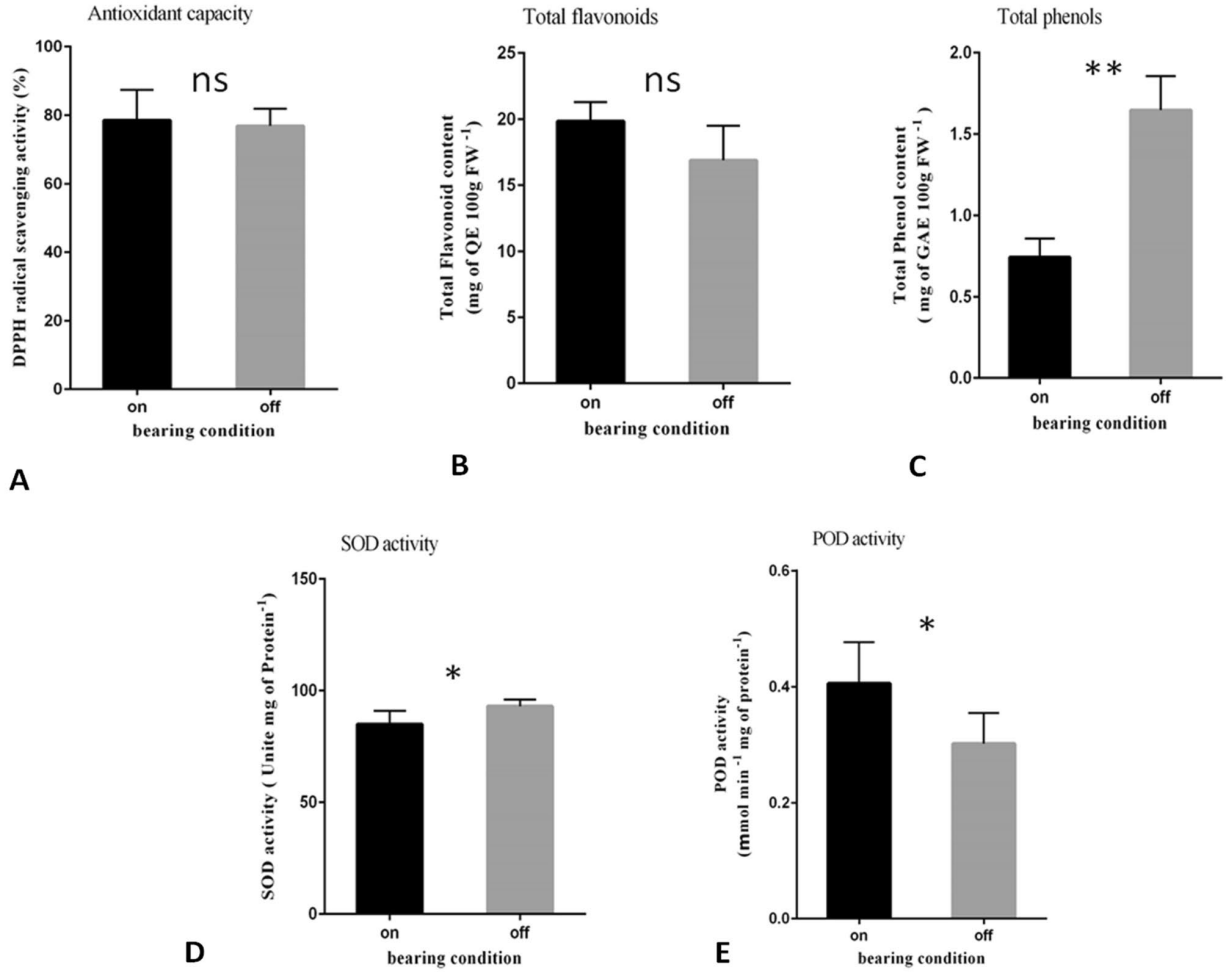
Means comparison of some biochemical parameters between ON- and OFF-olive trees using t-test (p ≤ 0.05). (**A**) Antioxidant Capacity (DPPH radical scavenging activity percent), (**B**) Total Flavonoid (mg of quercetin per 100 g fresh weight), (**C**) Total Phenolic Compounds (mg gallic acid per 100 g fresh weight), (**D**) Superoxide Dismutase (SOD) activity (Units per mg protein), and (**E**) Peroxidase (POD) activity (Micromole per minute per mg protein). * and **: statistical significance at p ≤ 0.05 and p ≤ 0.01 respectively and ns: non-significant**.**

No significant difference was seen between ON- versus OFF-trees in term of inhibition of DPPH activity and total flavonoid content in leaves (Fig. 5A,B). However, a significant difference was revealed in total phenol content (Fig. 5C). The activity of SOD and POD antioxidant enzymes was significantly different between trees. SOD, showed high amount of activity in OFF-trees, while the POD had its highest activity in ON-trees (Fig. 5D,E).

## Discussion

We sequenced and studied the eight cDNA libraries obtained from leaves and buds of ON and OFF olive trees during flower induction in July, 2018. The quantity of DEGs in the bud-derived libraries was nearly four times more than that of leaf-derived one. These results indicate that a large number of differentially expressed mRNAs are involved in buds metabolism than in the leaves under AB condition. No wonder, because the bud is the only organ in which a phase transition to the reproductive phase takes place.

### Carbohydrate metabolism and phytohormones

Photosynthesis is a key biological process in plant growth and development. Only one gene homologue to rubisco enzyme (YP_003359367.1), that has the main role in CO_2_ fixation, was up-regulated in the leaves of ON-trees while seven homologues genes of the rubisco in the buds had different transcript patterns with higher expression in ON-trees (i.e., OFF-buds for return bloom). There were significant differences in the conversion pathways of carbohydrate compounds between ON- and OFF-trees in both leaf and bud samples. In leaves, for instance, the homologue of galactosyl transferase 6 (XP_022863390.1) showed near 25-fold up-regulation in ON-trees. There are some contradictory reports on the involvement of photosynthesis, and carbohydrate contents on the flower induction events in olive trees^6,11^. While some researchers suggested that photosynthesis was not changed between leaves of ON- and OFF-trees^7,27^, others showed an increase rate of CO_2_ assimilation in high fruit-bearing trees^28,29^. Shalom et al.^1^ showed that induction of gene expression was related to photosynthesis in the citrus OFF-buds, which is in agreement with our finding in olive’s buds. Andreini et al.^30^ revealed that the anatomical and histochemical analysis of starch and total carbohydrate accumulation changes at the cellular level, did not allow discrimination of the buds sampled from ON- and OFF-shoots in the olive trees during flower induction stage. These all may suggest the oxygenase activity of rubisco (i.e., photorespiration) in olive’s OFF-buds. Gene homologues related to the majority of enzymes involved in photorespiration pathway were up-regulated in buds of ON-trees. Buds of ON-trees have been suggested to withstand a stressful conditions resulting from heavy fruit load^31^. Photorespiratory metabolism is prerequisite for rapid physiological acclimation against different unusual plant growth conditions. In this way, photorespiration in connection with phytohormones signaling contributes for mitigating stressor effects^32^. In *Pisum sativum*, for instance, Fedina et al.^33^ reported that the salinity stress resulted in increase of photorespiration when, treated with ABA eliminate the adverse effect of NaCl. Furthermore, the precursors for other metabolic processes, e.g., glycine for the synthesis of glutathione, are provided through the photorespiration pathway, which is also involved in stress protection^25^. Our data showed that a gene homologue to glutathione reductase (GR) (XP_022860602.1) in olive’s bud samples of ON-trees was up-regulated 6.85-fold in comparison to those of OFF-trees.

Endogenous plant hormones are involved in AB phenomenon. Hormones such as ABA, GAs and auxins^11,14^, and miRNAs that targeted genes involved in hormone-mediated signaling^2^ are expressed differentially in olive ON- and OFF-trees. We found one sequence homologue to zeaxanthin epoxidase (ZEP) (XP_012839200.1) in the leaves and four sequences (XP_022863205.1) in the buds which were up-regulated in samples of ON-trees. The conversion of zeaxanthin to violaxanthin is catalyzed by the ZEP enzyme. This reaction contributes to the biosynthesis of ABA^34^ which regulates the expression of stress-responsive genes. The high expression of ZEP might be related to the presence of increased amount of ABA in olive leaves, and particularly in buds, as a result of stressful conditions imposed by fruit load in bearing trees. An increasing level of ABA in citrus’ buds from ON-trees has previously been reported^31^. ABA is considered as an inhibitor of flower induction in *A. thaliana*, where mutants with high ABA levels showed late flowering^35^. It is likely that ABA plays the same role in olive buds of ON-trees, and up-regulation of sequence homologues to the biosynthesis of ABA may reflect its alleviating role for increasing photorespiration metabolisms in olive buds on ON-trees. However, relation between photorespiration metabolism and ABA-mediated signaling pathway remains unclear.

Two gene homologues to cytokininriboside 5′-monophosphate phosphoribohydrolase LOG1- and LOG3-like (XP_022894450.1 and XP_022887256.1) were up-regulated in olive’s bud of ON-trees. These enzymes mediate production of cytokinin nucleobases and contribute in regulating cytokinin activity during plant growth^36^. Cytokinins are a class of phytohormones which regulate leaf senescence, lateral root formation, cell division and stress tolerance^37^. There are contradictory reports about the involvement of cytokinin in relation to AB in olive. While, Ulger et al.^11^ suggested the possibly of positive effect of cytokinins during the flower induction period on the floral formation, Al-Shdiefat and Qrunfleh^10^ could not clarify any relationship between flower induction and initiation and cytokinin content. The results of current work showed no meaningful DE in the expression of gene(s) related to the cytokinin synthesis pathway(s) in leaf samples of ON- and OFF-trees. However, this difference was revealed between ON- and OFF-bud samples. Andreini et al.^30^ described the accumulation of Zeatin in olive OFF-axillary bud meristems in July by means of immunocytochemical studies. The possible role of up-regulated gene homologues to CK synthesis and increasing photorespiration metabolism in olive buds from ON-trees should not be ignored. The cytokinin-mediated occurrence of photorespiration has been reported in the *ipt* transgenic tobacco (*Nicotiana tabacum*) plants, suggesting the contribution of photorespiration in the protection of photosynthetic processes during drought stress^38^.

### Phenolic compounds, antioxidant enzymes-related genes and polyamines

Homologues of genes involved in few secondary metabolism pathways including caffeic acid and cinnamic showed DE pattern in leaves and mostly in buds. A gene homologue to chalcone synthase-like (XP_022852825.1), a key enzyme of the flavonoid/isoflavonoid biosynthesis pathway, was up-regulated more than seven-fold in buds of ON-trees compare with OFF-trees. One gene homologue to caffeic acid (XP_022880295.1), was in common with leaves and buds, and another homolog (XP_022880297.1) was down-regulated just in buds in ON-trees. The phenolic compounds are secondary plant metabolites that change in response to environmental stimuli, and even these changes may vary from one tissue to another^39^. In an attempt to establish a correlation between AB and total phenol content and its constituents, Mert et al.^40^ addressed lower accumulation of caffeic acid and chlorogenic acid in the leaves in ON- compared with OFF-years. The data from present study revealed that the gene homologue to caffeic acid metabolism pathway in bud samples of ON-trees was down-regulated. Total phenolic compounds in leaf samples, showed significant differences between ON- and OFF-trees parallel to the data from Mert et al.^40^. However, the analysis here could not detect gene homologue(s) to those of chlorogenic acid metabolism, which has been considered to have a probable role in olive flower induction and AB^41–43^. RNA-Seq results showed no significant differences between ON- and OFF-trees in terms of DPPH presence and enzymes which are involved in flavonoid biosynthesis such as flavonoid 3′,5′-methyltransferase, flavonol 3-*O*-glucosyltransferase and flavonoid 3′-monooxygenase.

Two gene homologues to the antioxidant enzyme POD (XP_022877955.1; XP_022893074.1) in leaves and another one (XP_022851859.1) in bud samples were up-regulated in ON-trees. The direct measurement of POD activity was consistent with the results of RNA-Seq in terms of its increasing expression in leaf samples from ON- versus OFF-trees. The electron transport processes in photosynthesis and respiration are the main production source of the reactive oxygen species (ROS) which in turn activates signal transduction processes. The imbalance between ROS detoxification and generation imposed oxidative stress to the plant cell^44^. From the point view of a stressful condition of the bearing tree and a high metabolism of oxidation–reduction process, one can explain the up-regulation of gene homologues to POD. Therefore, the accumulation of the antioxidant enzymes might dissipate the excess ROS resulting from the stressful condition in ON-trees. Unlike POD, the expression of a gene homologue to SOD (XP_022888361.1) showed DE just in leaves and decreased in samples of ON-trees. This result was in agreement with that of SOD activity determination in leaf samples carried out here. The involvement of copper-regulated microRNAs in down-regulation of copper/zinc SOD expression in *Arabidopsis thaliana* and response to low copper has been previously reported^45^. Since there is a high competition between vegetative organs and growing fruits for nutrients, one may assume that such micronutrient-regulated microRNA system might be involved in down-regulation of SOD in olive’s leaves in response to probably lower micronutrients such as Mn, Fe, Cu, and Zn under fruit bearing conditions.

Polyamines (PAs) such as spermine, spermidine, and putrescine (their obligate precursor) are a group of phytohormone-like aliphatic amine natural compounds, which contribute in the regulation of physiological processes and plant developmental^46^. Our results showed DE of seven gene homologues which involved in polyamines metabolism of which, up-regulation of a gene homologue to *S*-adenosyl methionine decarboxylase (SAMDC) (XP_022844785.1) in leaf and bud samples of ON-trees was in common. SAMDC is an enzyme which is involved in the synthesis of polyamines in plants, mammals, and other species^47^. PAs are well known for their anti-stress and anti-senescence effects due to their antioxidant properties and acid neutralizing, as well as for their cell wall and membrane stabilizing abilities^46^. The present study suggests that the up-regulation of the gene homologue to PAs biosynthesis in leaves and bud samples may represent a biochemical response of the tree to cope with high fruit load stress. Supporting this, it has been shown that the transformation of a plant with genes encoding SAMDC, spermidine synthase and/or arginine decarboxylase improved tolerance to environmental stress^47^. A gene homologue to thermospermine synthase (PIN17113.1) in bud samples of ON-trees showed more than eight-fold up-regulation in comparison to OFF-trees. In Arabidopsis, it has been assumed that thermospermine acts as a regulator of stem elongation, as sever dwarf plants were obtained in mutants that encoded thermospermine synthase^48^. Based on our data and earlier studies, it might be suggested that up-regulation of a gene homologue of thermospermine in olive’s buds of ON-trees should have a negative role in anatomical development of buds toward being flower meristematic apex.

### Flowering control genes

A complex genetic network controls the transition phase to flowering. In citrus, crop load negatively affected the expression of genes such as those homologues to *Flowering Locus T (FT)* and *LEAFY (LFY)*^49^. Haberman et al.^50^ revealed increased expression of two FT-encoding genes, *OeFT1/2* in leaf and *OeFT2* in bud samples of OFF-olive trees during flower initiation/formation. Our results, showed no significant DE of gene(s) in leaves responsible for transition to flowering in ON- and OFF-trees, based on sampling at flower induction period in July. In buds, however, one gene homologue to *MADS-box transcription factor 27-like* (XP_022855576.1) was down-regulated about 8.50-fold in ON-trees. MADS-type proteins have a pivotal role in the process of flower formation^51^. A gene homologue to *flowering time control protein FCA-like* (XP_022860886.1) was down-regulated about 5.16-fold in bud samples of ON-trees. The involvement of FCA, a plant-specific RNA-binding protein, in the autonomous flowering pathway in *A. thaliana* has been shown^52^. Three gene homologues to *flowering locus K (FLK)* homology domain showed DE in bud samples; two of them (XP_022876625.1; XP_022889081.1) were down-regulated and another one (XP_022876624.1) was up-regulated in ON-trees. The FLK, a protein containing three RNA-binding domains, acts in the autonomous flowering-promotive pathway^53^. Regardless of its role, simultaneous up- and down-regulation of different homologues of FLK is in the debate. Probably, different isoforms of these genes present in olive. This is probable because of relatively huge (2n = 46), complex (heterozygosity and different alleles at gene locus) and not well-known genome in olive to compare with model plant species^54^. A gene homologue to *Upstream of Flowering Locus C* (UFC) isoform X1 (XP_022850620.1) showed a down-regulation trend in buds of ON-trees. The UFC is component of a cluster containing three genes; *Flowering Locus C* (FLC), UFC and *Downstream of FLC* (DFC), which are coordinately regulated in response to environmental stimulus^55^. The UFC is considered as the flowering inhibitor. Thus, we expected to find its up-regulation in buds of ON-trees (OFF-buds for return blooming).

## Conclusions

This study clarified, to some extent, the transcriptional alterations of endogenous metabolic pathways in olive leaves and buds under AB conditions during flower induction using Illumina HiSeq 2000 system. De novo assembly strategy was used for creating the reference genome and further analysis and mapped the majority of clean reads in every library with constructed contigs and blasted over 69% of the obtained transcripts with available databases. Additionally, some transcripts could not be associated with a specific function. This may be mainly due to the lack of sufficient reference database of olive in GenBank, it’s relatively large genome size, and divergent gene functions in such species. DE of 57 gene homologues to carbohydrate metabolism were revealed in leaves’ and buds’ samples of ON- versus OFF-trees, supporting an important role for carbohydrate metabolism especially in sugar-mediating signaling pathway(s) under AB condition. The increase of oxygenase activity of rubisco (i.e., photorespiration) in olive’s buds from ON-trees was hypothesized as a contribution to multiple signaling pathways, particularly those that govern plant hormonal and defense responses. In connection with that, four homologous sequences of ABA biosynthesis pathway in buds and another homologue in leaves of ON-samples showed up-regulation. The results could not detect DE of gene homologue(s) to those involved in chlorogenic acid metabolism, which was considered to have a role in olive flower induction and AB. However, the down-regulation of gene homologous to those involved in caffeic acid metabolism, as a probable metabolic precursor of chlorogenic acid metabolism pathway, was shown in buds samples of ON-trees. It is suggested that a few number of genes corresponding to flower development are expressed differentially during flower induction period in olive’s buds under AB. More probably, the majority of these genes would be differentially expressed during flower initiation and developmental phases in late summer onwards. Indeed, genes such as MADS-box transcription factor 27-like and/or FCA-like are likely a component of a posttranscriptional cascade involved in the control of flowering. Proteomic analysis of olive buds and leaves is our ongoing project to shed much more light to the pathway for the transition to flowering in the olive tree under AB condition. From practical and horticultural view of points, we approved that the change in expression pattern of many genes from different metabolism pathway, especially those direct or indirect related to stressful condition, have been started in July, correspond to flower induction period in olive tree. Hence, general encouraging of tree tolerance to abiotic stress at this time or even sooner, improving nutritional status (especially those of micronutrients) and spraying with different antioxidant compounds for instances, could modulated to some extent the AB behavior in this fruit tree species. Of course, continuous treatment of tree and orchard later on, during flower initiation and development, should also be considered for getting satisfied results in this respect.

## Materials and methods

### Plant materials

The experiment was carried out on 12-year-old own-rooted olive trees cv. ‘Conservalia’, which is considered as a severely AB cultivar^3^, at Tarom Olive Research Station (49° 05′ E, 36° 47′ N). Two ON- and two OFF-trees (approximately 5 m apart from each other) with the same growth condition and under similar treatment were selected^5^. To insure on crop load of the given trees and on the reliability of their ON- and OFF-status, 14 trees (seven of each ON- and OFF-conditions) were selected and monitored for their crop load status of previous and the current year. Later, two trees in ON and OFF conditions each, with clear AB status were selected for sampling. The mean of crop yield was 5.2 and 60.8 kg/tree in OFF- and ON-trees, respectively.

The leaf and bud samples were collected at flower induction time in July parallel with fruit pit hardening^56^. Samples were collected from the middle part of current year growth (fifth and sixth nodes from the top of the shoot) which is responsible for the fruit production in the following year^57^. Although a little mix of vegetative and generative buds is possible in bud sampling however, the majority of collected bud sample from ‘ON’ tree were surly vegetative buds for return blooming in the next year and vice versa. According to literature, in mentioned sampling time there is no differences between OFF- and ON-buds from the morphological points of view however, molecular and histochemical evidence have confirmed their differences at flower induction period^30^. In order to minimize the environmental noise on transcriptome profiles, the sampling in all replicates was performed only from one side (southeast) of the trees at shoulder height.

For assessment of some biochemical traits, five branches (10 replications of each ‘OFF’ and ‘ON’ status in total) were selected. Collected samples were immediately immersed in liquid nitrogen after a surface cleaning with ethanol 70% and then transferred to the lab and stored in a − 80 °C freezer until use.

### RNA preparation and next-generation sequencing

Total RNA was extracted using Trizol Reagent kit (Invitrogen) according to the manufacturer’s instructions with two biological replicates. The RNA concentration and purity was examined using both Nanodrop spectrophotometer (Thermo Fisher Scientific) and Bioanalyzer (Agilent technologies). High-quality total RNA (260/280, 1.8–2.0; 260/230 > 2.0; RIN > 7.5) was subjected to cDNA library synthesis using SMART-Seq protocol. The libraries were sequenced on an Illumina HiSeq 2000, 150 base pair-end reads at Beijing Genomics Institute (BGI) (Hong Kong) according to standard procedures.

### Bioinformatics analysis

Raw reads were filtered for low-quality reads, adaptor sequences and unexpected contamination. The sequencing quality control of clean reads was obtained by FastQC v.0.11.5 software^58^ (https://www.bioinformatics.babraham.ac.uk/projects/fastqc). There was only one released data by “International Olive Genome Consortium” includes 50,684 genes (cds), at the time of analyzing and preparing the current manuscript, and we used this data for GO analysis. However, the results were very poor and unreliable, hence were ignored. The assembly of clean reads has been performed by Trinity v.2.4.0 software^59^. For this aim, first a de novo assembly file was done by the combination of all reads of every eight libraries. The output obtained was used as a reference genome for further analysis. This procedure is reliable and strong strategy which has been used for molecular study of wide plant species such as olive^18,20^, pear^60^ and pistachio^61^. By de novo assembly strategy, we mapped the majority of clean reads in every library with made contigs and blasted over 69% of the obtained transcripts with available databases.

Each sample read was mapped by Bowtie2 v.2.3.4.1 software^62^ on the contigs derived from the Trinity analysis as the reference genome. Using the Trinity output and by the Kallisto v.0.43.1 software^63^, the counting of the reads was done in the desired samples. The fragments per kilobaseof transcript per million mapped reads (FPKM) derived from the counting of the reads were used to compare the differences among the samples. DE analysis by RNA-Seq reads was performed for two groups of data including "Bud ON *vs.* OFF" and "Leaf ON vs. OFF". The significant threshold for the detection of genes with DE adjusted P-value ≤ 0.05 was considered and genes with log twofold change ≥ 1 and ≤ − 1 were considered as genes with DE. DE analysis was performed by the R package, DESeq2 v.3.7^64^. Then, BLASTx was performed with the Nr database by Diamond v.0.9.14 software and using blast result, the gene ontology (GO) analysis was done by Blast2GO software^65^ (https://www.blast2go.com) for differential expressed genes. GO terms with e-Values ≤ 0.05 were considered significant. Kyoto Encyclopedia of Genes and Genomes (KEGG) pathway enrichment analysis was performed using KOBAS v.3.0 (https://kobas.cbi.pku.edu.cn/anno_iden.php)^66^. Pathways with corrected P-values ≤ 0.05 were considered significant.

### Quantitative reverse transcription PCR (Q-RT-PCR) validation

In order to validate the RNA-Seq data, four cDNA sequences, including two from leaf samples (TRINITY_DN81593_c2_g2_i7 and TRINITY_DN 78486_c0_g1_i14, gene homologous of POD and SOD respectively) and another two from bud samples (TRINITY_DN81593_c2_g2_i7 and TRINITY_DN78486_c0_g1_i14, gene homologous of POD and rubisco respectively) were analyzed using Q-RT-PCR. Primers were designed using Primer3 (https://primer3.ut.ee/). The actin gene was used as an internal reference control. The primer sequences used for Q-RT-PCR have been shown in Supplementary Table S8. The Q-RT-PCR reaction was performed by a Rotor-Gene 3000 system (Qiagen). Amplification was done in two technical replicates with a reaction volume of 20 μl containing 0.5 μl (0.1 μM) of each forward and reverse primer, 2 μl (160 ng) of diluted cDNA, 4 μl of 5 × HOT FIREPol EvaGreen qPCR Mix Plus (Solis BioDyne) and 13 μl of sterile distilled water. The cycling parameters were as follows: one cycle at 95 °C for five min to activate the Taq enzyme, followed by 40 cycles of denaturation at 95 °C for 20 s, annealing at 55 °C for 20 s and extension at 72 °C for 12 s. The final followed by a melting step in a 50–99 °C range by an increase of 1 °C every 5 s to evaluate the “melting curve” for confirming the occurrence of a unique PCR product. Relative expression levels were calculated using the 2^−△△CT^ method^67^.

### Measurement of biochemical parameters in leaves

A number of biochemical parameters including antioxidant capacity, total flavonoid and phenol contents as well as the activity of peroxidase (POD; EC 1.11.1.7) and superoxide dismutase (SOD; EC 1.15.1.1) enzymes in leaf samples were assessed. Bud samples were excluded from those mentioned evaluations. Due to the small size of buds, it was necessary to ruin large amounts of shoots for gathering enough samples, which could result in irreparable damage to trees as well as non-uniformity in plant materials.

Total phenolic compounds were measured by reaction with the Folin-Ciocalteu reagent and gallic acid as standard^68^. The results were expressed in mg gallic acid equivalents (GAE) per 100 g FW. Total flavonoids were assessed by reaction with AlCl_3_, as described by Chang et al.^69^, and quercetin was used as standard. Results were expressed in mg quercetin acid equivalent (QE) per 100 g FW. To evaluate the antioxidant activity, DPPH free radical scavenging activity^70^ was used. The results of this analysis were expressed in terms of the ‘percentage of DPPH free radical scavenging’.

The enzymatic extract was prepared to measure total soluble protein, SOD and POD enzymes activity using 50 mM potassium phosphate buffer with a pH = 7. The SOD enzyme activity was measured by the method of Giannopolitis and Ries^71^ using spectrophotometer at a wavelength of 560 nm. The results of this enzyme activity were expressed in ‘units per mg protein’. The POD enzyme activity was measured by the method of Abeles and Biels^72^ using spectrophotometer. Absorbance changes were measured in 120 s at 470 nm and enzyme activity was expressed as ‘micromole per minute per mg protein’. Statistical analyses of this section were performed using unpaired t-test.

## Supplementary information

Supplementary Table 1.

Supplementary Table 2.

Supplementary Table 3.

Supplementary Table 4.

Supplementary Table 5.

Supplementary Table 6.

Supplementary Table 7.

Supplementary Table 8.

## Data Availability

All the data analyzed or generated during this study are included in this article. This Sequence Read Archive (SRA) submission has been released on 2020-07-01. The data that support the findings of this study are deposited at https://trace.ncbi.nlm.nih.gov/Traces/study/?acc=PRJNA549052 and received submission code of SUB5704553.
