# Author Correction: Differential expression of genes in olive leaves and buds of ON- versus OFF-crop trees

**DOI:** 10.1038/s41598-022-17904-7

**Published:** 2022-08-12

**Authors:** Ebrahim Dastkar, Ali Soleimani, Hossein Jafary, Juan de Dios Alche, Abbas Bahari, Mehrshad Zeinalabedini, Seyed Alireza Salami

**Affiliations:** 1 Department of Horticulture, Faculty of Agriculture, University of Zanjan, Zanjan, Iran; 2 Iranian Research Institute of Plant Protection, Agricultural Research, Education and Extension Organization (AREEO), Tehran, Iran; 3 Plant Reproductive Biology and Advanced Microscopy Laboratory, Department of Biotechnology, Cell and Molecular Biology of Plants, Estación Experimental del Zaidín (CSIC), Granada, Spain; 4 Research Institute of Modern Biological Techniques (RIMBT), University of Zanjan, Zanjan, Iran; 5 Department of Systems and Synthetic Biology, Agricultural Biotechnology Research Institute of Iran (ABRII), Agricultural Research, Education and Extension Organization, Karaj, Iran; 6 Faculty of Agricultural Science and Engineering, University of Tehran, Tehran, Iran

Correction to: *Scientific Reports* https://doi.org/10.1038/s41598-020-72895-7, published online 25 September 2020

The original version of this Article contained an error in the link in the Data Availability section where,

“All the data analyzed or generated during this study are included in this article. This Sequence Read Archive (SRA) submission has been released on 2020-07-01. The data that support the findings of this study are deposited at https://submit.ncbi.nlm.nih.gov/subs/sra/ and received submission code of SUB5704553.”

now reads:

“All the data analyzed or generated during this study are included in this article. This Sequence Read Archive (SRA) submission has been released on 2020-07-01. The data that support the findings of this study are deposited at https://trace.ncbi.nlm.nih.gov/Traces/study/?acc=PRJNA549052 and received submission code of SUB5704553.”

The original Article has been corrected.

