# Supplementary material for: Differential expression of genes in olive leaves and buds of ON- versus OFF-crop trees
Source: Sci Rep. 2020 Sep 25;10:15762. doi: 10.1038/s41598-020-72895-7 (PMC7519672; doi:10.1038/s41598-020-72895-7)
Supplement: Supplementary file 1 — Supplementary Table 1. [file 41598_2020_72895_MOESM1_ESM.pdf]

**Alternate bearing in olive: Differential expression of genes in leaves a**

**Ebrahim Dastkar[1], Ali Soleimani[1*], Hossein Jafary[2], Juan de Dios Alche[3], Abbas Bahari**

**Supplementary table S1. Results of differential expression (DE) anal**

| SeqName | baseMean | log2FoldChan | lfcSE |
|---|---|---|---|
| TRINITY_DN86226_c1_g1_i11 | 756.8841313 | -6.27889138 | 0.64017446 |
| TRINITY_DN87854_c1_g1_i1 | 254.1408358 | -5.88486658 | 0.61493923 |
| TRINITY_DN87899_c2_g1_i2 | 962.4946929 | 3.27204296 | 0.38818755 |
| TRINITY_DN83817_c3_g2_i11 | 237.9219051 | 12.3221766 | 1.61960897 |
| TRINITY_DN79244_c2_g1_i1 | 144.9778115 | -7.28294152 | 0.9668866 |
| TRINITY_DN86226_c1_g1_i10 | 306.9587533 | -7.77121474 | 1.05803603 |
| TRINITY_DN84189_c2_g7_i1 | 906.7314858 | 3.29288526 | 0.44744759 |
| TRINITY_DN82827_c0_g2_i4 | 666.1363814 | 24.9227195 | 3.40399279 |
| TRINITY_DN79101_c1_g3_i7 | 335.7001216 | -24.1706521 | 3.36033841 |
| TRINITY_DN85165_c2_g1_i5 | 20841.30453 | 3.10888933 | 0.43210571 |
| TRINITY_DN84355_c1_g3_i2 | 362.8197236 | 11.1289849 | 1.5586287 |
| TRINITY_DN88765_c4_g5_i2 | 6940.34858 | 3.42311026 | 0.49835397 |
| TRINITY_DN88027_c3_g6_i4 | 11374.55498 | 2.95129186 | 0.43469209 |
| TRINITY_DN80414_c1_g1_i2 | 2233.669808 | -3.08859033 | 0.4570599 |
| TRINITY_DN88736_c1_g2_i1 | 255.9232998 | 31.6227471 | 4.78503917 |
| TRINITY_DN87124_c0_g6_i4 | 286.1313334 | -10.6933973 | 1.64169419 |
| TRINITY_DN80700_c1_g1_i3 | 490.7761847 | 2.69099924 | 0.41750195 |
| TRINITY_DN78090_c4_g1_i14 | 94.31975218 | 10.6673665 | 1.68173902 |
| TRINITY_DN88734_c4_g1_i2 | 7998.723462 | 2.95520995 | 0.46540378 |
| TRINITY_DN87165_c1_g1_i3 | 95.47899054 | -10.9248571 | 1.75099371 |
| TRINITY_DN79244_c2_g4_i7 | 98.14684863 | -9.59786439 | 1.5425109 |
| TRINITY_DN86226_c1_g1_i18 | 159.1659613 | -7.42911041 | 1.20891989 |
| TRINITY_DN88027_c3_g2_i2 | 9359.860832 | 2.93760528 | 0.47865416 |
| TRINITY_DN85628_c2_g1_i10 | 149.9013634 | 3.2272405 | 0.52676519 |
| TRINITY_DN87131_c2_g3_i1 | 3420.227539 | 2.71240406 | 0.44454241 |
| TRINITY_DN80388_c3_g3_i4 | 385.440822 | 9.53432117 | 1.57619724 |
| TRINITY_DN88496_c1_g2_i16 | 592.6310004 | -4.90456135 | 0.81788766 |
| TRINITY_DN85868_c3_g8_i3 | 5905.548812 | 2.7358483 | 0.45811269 |
| TRINITY_DN82021_c1_g4_i1 | 564.6084173 | 2.87769138 | 0.48538066 |
| TRINITY_DN85074_c1_g2_i6 | 151.3882117 | 9.70818539 | 1.64133502 |
| TRINITY_DN83817_c3_g2_i20 | 74.60098457 | -6.08329433 | 1.02878127 |
| TRINITY_DN79826_c3_g6_i3 | 6828.924847 | 3.02696727 | 0.51321577 |

| | | | |
|---|---|---|---|
| TRINITY_DN79223_c1_g2_i9 | 91.1644473 | -8.40704315 | 1.42676349 |
| TRINITY_DN88846_c3_g2_i3 | 29469.33466 | 3.15074422 | 0.53780488 |
| TRINITY_DN81974_c3_g1_i12 | 66.5568655 | -10.3941998 | 1.77318708 |
| TRINITY_DN84336_c1_g3_i2 | 22979.12257 | 2.94600854 | 0.50208799 |
| TRINITY_DN88600_c0_g1_i1 | 1952.248927 | 2.50343266 | 0.43320416 |
| TRINITY_DN79244_c2_g2_i3 | 152.7387801 | -5.46729073 | 0.94725854 |
| TRINITY_DN78474_c3_g1_i1 | 13244.54646 | 3.15376953 | 0.54866519 |
| TRINITY_DN80836_c4_g1_i2 | 491.3198013 | 2.89238572 | 0.5059362 |
| TRINITY_DN88846_c4_g2_i2 | 15830.87748 | 3.08625067 | 0.54115941 |
| TRINITY_DN84553_c2_g1_i7 | 62.76287391 | -9.64176461 | 1.72202837 |
| TRINITY_DN86017_c1_g2_i5 | 3652.659119 | 3.66879544 | 0.65672255 |
| TRINITY_DN87131_c2_g1_i1 | 163.2282068 | 3.29494955 | 0.59148088 |
| TRINITY_DN82699_c1_g1_i7 | 108.4569431 | 8.57860768 | 1.55054447 |
| TRINITY_DN87128_c2_g1_i1 | 51.22371762 | -9.84523782 | 1.7788908 |
| TRINITY_DN79705_c0_g2_i8 | 22096.88959 | 3.12227613 | 0.56528599 |
| TRINITY_DN83746_c1_g1_i4 | 146.3874496 | 10.3325901 | 1.87255988 |
| TRINITY_DN79425_c0_g1_i3 | 101.5264233 | 9.29702514 | 1.70421267 |
| TRINITY_DN86837_c1_g2_i1 | 1713.160544 | 2.48182495 | 0.45687843 |
| TRINITY_DN82982_c0_g1_i5 | 101.9240457 | 8.59472535 | 1.59935568 |
| TRINITY_DN84045_c0_g4_i1 | 50580.80018 | 3.07166037 | 0.57201596 |
| TRINITY_DN85116_c0_g2_i6 | 32.83911125 | -9.03415106 | 1.69259653 |
| TRINITY_DN82813_c0_g2_i2 | 44.05221191 | 8.63399515 | 1.62127628 |
| TRINITY_DN86488_c0_g1_i5 | 971.2838806 | -2.10080503 | 0.39523998 |
| TRINITY_DN79532_c1_g2_i2 | 1258.541334 | 2.47062944 | 0.46815326 |
| TRINITY_DN87236_c2_g4_i1 | 27.76436486 | 8.89851698 | 1.68482636 |
| TRINITY_DN87633_c0_g1_i3 | 96.05188065 | 8.38068349 | 1.58859414 |
| TRINITY_DN80450_c2_g1_i14 | 184.3297246 | -8.52433722 | 1.62346108 |
| TRINITY_DN86226_c1_g1_i19 | 75.93256449 | -9.4786462 | 1.80907285 |
| TRINITY_DN87165_c1_g4_i2 | 1330.833177 | 8.42399456 | 1.61301932 |
| TRINITY_DN81228_c1_g4_i6 | 109.0754612 | 9.24219373 | 1.77026217 |
| TRINITY_DN79625_c0_g1_i7 | 62.78376535 | -9.16052762 | 1.75727658 |
| TRINITY_DN87173_c1_g4_i3 | 71.51267805 | -8.30198574 | 1.60680391 |
| TRINITY_DN88146_c0_g7_i6 | 48.07889282 | -8.55047103 | 1.66053452 |
| TRINITY_DN86621_c3_g3_i2 | 2321.643559 | 2.31629703 | 0.45013524 |
| TRINITY_DN82900_c2_g1_i6 | 140.0863714 | 8.31337878 | 1.61553462 |
| TRINITY_DN88846_c3_g2_i13 | 82.3985405 | 24.5866267 | 4.78518099 |

| | | | |
|---|---|---|---|
| TRINITY_DN83892_c1_g2_i19 | 719.1871116 | 9.66151647 | 1.88099765 |
| TRINITY_DN84441_c0_g1_i6 | 36.57063808 | 8.57676493 | 1.67399226 |
| TRINITY_DN86045_c3_g1_i1 | 297.9272017 | 2.62953474 | 0.51450215 |
| TRINITY_DN88600_c6_g4_i3 | 12417.42802 | 2.87702329 | 0.56325273 |
| TRINITY_DN78830_c5_g3_i4 | 900.3829943 | 5.5591234 | 1.08979291 |
| TRINITY_DN87668_c0_g1_i4 | 68.85928188 | 9.4541049 | 1.86091272 |
| TRINITY_DN83759_c1_g4_i5 | 40.75478267 | -8.32806638 | 1.65930336 |
| TRINITY_DN88632_c2_g1_i3 | 46.63028843 | -9.41290524 | 1.87553812 |
| TRINITY_DN81934_c0_g1_i5 | 47.71174445 | -7.29266044 | 1.45418939 |
| TRINITY_DN87075_c1_g2_i3 | 46.25957098 | -9.12710961 | 1.82314357 |
| TRINITY_DN85661_c3_g1_i2 | 224.7060062 | 8.05664789 | 1.61301675 |
| TRINITY_DN82239_c0_g1_i20 | 56.37850388 | -8.35273493 | 1.67692704 |
| TRINITY_DN76217_c1_g1_i4 | 138.2999233 | -7.68136796 | 1.54449137 |
| TRINITY_DN80085_c4_g1_i14 | 30.63853265 | 9.0589854 | 1.82657643 |
| TRINITY_DN81654_c1_g2_i2 | 783.6471654 | 4.05833776 | 0.8171524 |
| TRINITY_DN86856_c0_g1_i3 | 136.2505246 | 9.96228973 | 2.00730173 |
| TRINITY_DN80028_c1_g4_i6 | 183.6380623 | 9.19162997 | 1.853081 |
| TRINITY_DN82793_c0_g1_i5 | 104.714866 | 8.31871355 | 1.68006968 |
| TRINITY_DN85516_c1_g3_i1 | 284.4578292 | 8.00932759 | 1.61784675 |
| TRINITY_DN86226_c1_g1_i17 | 55.36822014 | -6.4273682 | 1.29821637 |
| TRINITY_DN82285_c1_g1_i4 | 69.96890134 | 8.51306789 | 1.72350633 |
| TRINITY_DN84287_c1_g1_i6 | 171.8875347 | 4.67383267 | 0.94807226 |
| TRINITY_DN86843_c0_g1_i5 | 123.0607694 | 9.02226099 | 1.83246475 |
| TRINITY_DN86869_c0_g1_i14 | 266.6046749 | 3.30326243 | 0.67078513 |
| TRINITY_DN81726_c4_g3_i4 | 2363.289659 | -7.94711191 | 1.61521206 |
| TRINITY_DN80700_c1_g1_i4 | 801.673658 | 2.53555671 | 0.51619234 |
| TRINITY_DN81790_c2_g1_i3 | 52.10820258 | -8.32921346 | 1.69498477 |
| TRINITY_DN79244_c2_g4_i2 | 296.7565298 | -4.80674625 | 0.97918151 |
| TRINITY_DN87790_c2_g4_i1 | 331.4736181 | 3.08615681 | 0.62962565 |
| TRINITY_DN83301_c0_g2_i3 | 26.80810219 | 8.66475566 | 1.76971077 |
| TRINITY_DN88535_c4_g2_i6 | 61.58796236 | -5.44397142 | 1.11194517 |
| TRINITY_DN87154_c2_g2_i19 | 196.9859582 | 5.95489271 | 1.21846616 |
| TRINITY_DN85641_c3_g1_i2 | 70.15691093 | 8.27657019 | 1.69688237 |
| TRINITY_DN83788_c0_g1_i1 | 259.8440491 | 7.8645854 | 1.61350204 |
| TRINITY_DN87827_c5_g2_i7 | 1929.330496 | -2.36833489 | 0.4864601 |
| TRINITY_DN82802_c0_g2_i10 | 263.2639905 | 2.42341744 | 0.49862034 |

| | | | |
|---|---|---|---|
| TRINITY_DN86683_c0_g8_i1 | 193.5963452 | 8.91646734 | 1.83610347 |
| TRINITY_DN80040_c1_g1_i9 | 315.2844802 | -3.59453656 | 0.74175596 |
| TRINITY_DN88004_c1_g11_i3 | 1677.400521 | 2.43011415 | 0.50285989 |
| TRINITY_DN81285_c2_g2_i20 | 40.75282595 | 8.60693202 | 1.78383573 |
| TRINITY_DN88427_c1_g2_i12 | 130.2912082 | -11.2371951 | 2.32890568 |
| TRINITY_DN84578_c2_g1_i3 | 102.0448648 | 7.63510883 | 1.58301885 |
| TRINITY_DN88734_c4_g1_i6 | 451.5820308 | 2.64370704 | 0.5506174 |
| TRINITY_DN81485_c0_g2_i14 | 47.86511727 | 7.77886797 | 1.61977156 |
| TRINITY_DN86493_c2_g1_i9 | 89.80865293 | -7.78846089 | 1.62367284 |
| TRINITY_DN77691_c0_g1_i3 | 58.7845107 | -8.50701145 | 1.77304515 |
| TRINITY_DN82174_c3_g2_i7 | 186.2710308 | 3.13677341 | 0.65483258 |
| TRINITY_DN82385_c5_g2_i8 | 56.191773 | 7.91480741 | 1.65852693 |
| TRINITY_DN86957_c0_g2_i1 | 38.85595225 | -7.83169834 | 1.64102495 |
| TRINITY_DN78859_c0_g1_i7 | 32.18986072 | -7.97912768 | 1.67090753 |
| TRINITY_DN82814_c3_g3_i1 | 288.203404 | 7.49623311 | 1.57156394 |
| TRINITY_DN86380_c0_g2_i2 | 81.18387979 | -8.08579741 | 1.70019677 |
| TRINITY_DN85778_c2_g2_i8 | 81.69254007 | 8.02722187 | 1.6895742 |
| TRINITY_DN86328_c2_g1_i1 | 80.54604302 | 7.95679484 | 1.67847717 |
| TRINITY_DN85379_c0_g1_i1 | 64.25350262 | 8.52442191 | 1.80052622 |
| TRINITY_DN81043_c1_g1_i3 | 112.5606763 | 8.47872533 | 1.79508538 |
| TRINITY_DN82948_c1_g1_i1 | 109.0238066 | 10.8744621 | 2.30411273 |
| TRINITY_DN83533_c1_g1_i4 | 68.10766011 | 7.84524186 | 1.67020382 |
| TRINITY_DN87930_c2_g2_i8 | 480.8074934 | 3.37738196 | 0.72386174 |
| TRINITY_DN88011_c2_g1_i22 | 119.010336 | -3.15048191 | 0.67924168 |
| TRINITY_DN76217_c1_g1_i9 | 35.08340488 | 8.12071744 | 1.75178185 |
| TRINITY_DN86045_c3_g3_i1 | 992.8863437 | 2.31901542 | 0.5033689 |
| TRINITY_DN77717_c3_g1_i5 | 38.1464268 | 8.83809931 | 1.92069436 |
| TRINITY_DN80500_c1_g1_i3 | 32.05653481 | 7.84178282 | 1.70487142 |
| TRINITY_DN82516_c0_g2_i5 | 39.84463951 | 8.00552382 | 1.74603356 |
| TRINITY_DN85755_c0_g3_i8 | 36.03744843 | 7.96902592 | 1.73875829 |
| TRINITY_DN81596_c0_g3_i1 | 71.00897438 | 2.86140988 | 0.62377609 |
| TRINITY_DN85978_c0_g1_i5 | 82.05189872 | 8.04596481 | 1.75558396 |
| TRINITY_DN79647_c0_g1_i11 | 25.59942466 | -7.89699342 | 1.72669812 |
| TRINITY_DN85390_c0_g1_i4 | 69.13592896 | -3.02771179 | 0.6644012 |
| TRINITY_DN88060_c2_g2_i9 | 18.33783727 | 8.15336619 | 1.79146258 |
| TRINITY_DN81593_c2_g2_i7 | 1130.949016 | 5.31759789 | 1.16908677 |

| | | | |
|---|---|---|---|
| TRINITY_DN85435_c0_g1_i4 | 66.76251993 | 7.39571092 | 1.62719479 |
| TRINITY_DN82882_c2_g2_i18 | 331.0766139 | -2.1311033 | 0.4692998 |
| TRINITY_DN83887_c0_g1_i16 | 21.41805685 | 7.80120092 | 1.72101362 |
| TRINITY_DN85402_c0_g2_i17 | 63.43605397 | 3.45587053 | 0.76289077 |
| TRINITY_DN84865_c0_g1_i2 | 47.03366764 | 5.23156311 | 1.15659641 |
| TRINITY_DN79276_c0_g1_i3 | 56.65899842 | -7.9316022 | 1.75494115 |
| TRINITY_DN82036_c1_g1_i1 | 182.9106788 | -7.06141112 | 1.56531717 |
| TRINITY_DN85030_c4_g1_i1 | 31.98737658 | 7.51058383 | 1.6680718 |
| TRINITY_DN82242_c0_g3_i3 | 370.0359269 | 4.37505594 | 0.97216875 |
| TRINITY_DN80085_c4_g1_i8 | 39.69032142 | -6.56202643 | 1.45832729 |
| TRINITY_DN86797_c1_g1_i6 | 36.25075053 | -9.52767971 | 2.12042474 |
| TRINITY_DN77073_c0_g1_i3 | 37.83332716 | 7.6716399 | 1.71771303 |
| TRINITY_DN85300_c1_g1_i2 | 50.48740633 | 7.54335074 | 1.6896505 |
| TRINITY_DN87363_c0_g6_i4 | 24.53878761 | 7.70223227 | 1.72831233 |
| TRINITY_DN84682_c1_g2_i3 | 24.48002217 | 8.52525851 | 1.91576192 |
| TRINITY_DN86156_c0_g2_i9 | 23.00136812 | -8.40785839 | 1.89014717 |
| TRINITY_DN78430_c3_g5_i1 | 291.2296738 | 7.25964013 | 1.63506218 |
| TRINITY_DN82989_c1_g2_i16 | 51.25215504 | -9.72811695 | 2.20260586 |
| TRINITY_DN80543_c1_g1_i3 | 54.84420752 | 4.50988974 | 1.02202922 |
| TRINITY_DN87124_c0_g2_i13 | 26.2433701 | 8.06438061 | 1.8299561 |
| TRINITY_DN86856_c0_g1_i10 | 134.3842195 | 8.25118441 | 1.87554662 |
| TRINITY_DN87124_c0_g2_i3 | 69.9278178 | 10.1786806 | 2.31410451 |
| TRINITY_DN65310_c0_g1_i2 | 16.67686199 | 8.02217149 | 1.82742321 |
| TRINITY_DN83053_c0_g1_i4 | 103.2413384 | -7.42667119 | 1.69412322 |
| TRINITY_DN80097_c1_g1_i4 | 70.39997665 | 7.1382667 | 1.63406875 |
| TRINITY_DN80586_c1_g3_i1 | 34.2037067 | -9.4437726 | 2.16432614 |
| TRINITY_DN88846_c3_g2_i12 | 76.57101707 | 2.99132562 | 0.68640411 |
| TRINITY_DN82102_c2_g1_i3 | 17.88718414 | 7.56430359 | 1.73670914 |
| TRINITY_DN78493_c1_g2_i13 | 24.33903363 | -7.75927759 | 1.78654857 |
| TRINITY_DN82945_c0_g1_i5 | 25.89684845 | 8.30139859 | 1.91773305 |
| TRINITY_DN82079_c1_g1_i15 | 31.70169752 | -7.2558557 | 1.67591734 |
| TRINITY_DN81204_c0_g1_i4 | 63.11584954 | 4.72497089 | 1.0935724 |
| TRINITY_DN87005_c1_g1_i1 | 41.03214015 | -7.05564861 | 1.63103656 |
| TRINITY_DN77865_c2_g7_i1 | 501.4516141 | 2.63643356 | 0.61019388 |
| TRINITY_DN77499_c3_g2_i15 | 147.6031349 | 6.93435075 | 1.60413041 |
| TRINITY_DN74124_c0_g1_i2 | 85.28821697 | 4.33877444 | 1.00372581 |

| | | | |
|---|---|---|---|
| TRINITY_DN83917_c3_g1_i1 | 177.9261766 | -3.70390376 | 0.85728672 |
| TRINITY_DN76209_c0_g1_i3 | 21.16002817 | 7.74574425 | 1.79490993 |
| TRINITY_DN82951_c0_g1_i2 | 201.936073 | 2.26018965 | 0.52426063 |
| TRINITY_DN79425_c0_g1_i2 | 536.4876845 | 6.65548875 | 1.54333816 |
| TRINITY_DN81113_c1_g2_i4 | 20.84383723 | 8.27095602 | 1.9197049 |
| TRINITY_DN76432_c0_g1_i7 | 54.79152079 | 7.84576202 | 1.82369482 |
| TRINITY_DN77150_c0_g1_i4 | 30.7299979 | -7.61140189 | 1.7743893 |
| TRINITY_DN80906_c1_g1_i11 | 27.97879801 | 7.1967908 | 1.67860734 |
| TRINITY_DN79702_c0_g5_i2 | 205.5701596 | 1.80934075 | 0.42219531 |
| TRINITY_DN87157_c0_g2_i4 | 116.1238882 | 7.23286323 | 1.68882914 |
| TRINITY_DN85621_c1_g1_i3 | 31.45353606 | 7.19392706 | 1.67943625 |
| TRINITY_DN83246_c2_g1_i1 | 572.2057654 | 4.86301469 | 1.13671869 |
| TRINITY_DN83869_c1_g2_i1 | 25.34090612 | 7.51977353 | 1.76152368 |
| TRINITY_DN81156_c1_g1_i3 | 114.2923432 | 9.48076424 | 2.22305596 |
| TRINITY_DN87105_c1_g1_i7 | 35.36828792 | 7.00370846 | 1.64197722 |
| TRINITY_DN85090_c1_g2_i5 | 51.45149588 | 9.8344906 | 2.30575313 |
| TRINITY_DN85068_c2_g1_i6 | 389.7848886 | 4.60010983 | 1.07905452 |
| TRINITY_DN86147_c0_g1_i15 | 52.32865148 | -6.82881391 | 1.60343719 |
| TRINITY_DN81764_c0_g2_i2 | 35.52338085 | -7.26693468 | 1.70582321 |
| TRINITY_DN85636_c0_g1_i17 | 27.821267 | 7.1642457 | 1.68196426 |
| TRINITY_DN81372_c1_g2_i1 | 633.1260042 | -2.29581259 | 0.53965329 |
| TRINITY_DN78176_c1_g1_i1 | 16.86064308 | -7.50198715 | 1.76326954 |
| TRINITY_DN80820_c0_g3_i1 | 329.1825988 | 6.69775669 | 1.57553091 |
| TRINITY_DN78435_c0_g5_i3 | 74.72279149 | 2.95539071 | 0.69606516 |
| TRINITY_DN84737_c0_g1_i3 | 21.63863359 | 8.59237921 | 2.02323853 |
| TRINITY_DN85110_c1_g2_i6 | 57.46824752 | -7.30714356 | 1.72026249 |
| TRINITY_DN84328_c0_g6_i1 | 44.16660202 | 7.4717532 | 1.75959337 |
| TRINITY_DN81653_c0_g2_i13 | 27.42530651 | -8.3114842 | 1.95924177 |
| TRINITY_DN80397_c0_g2_i3 | 97.87999885 | -7.16973954 | 1.69070517 |
| TRINITY_DN79896_c1_g5_i1 | 288.4708583 | 2.41714813 | 0.57130926 |
| TRINITY_DN82519_c0_g5_i3 | 33.28336083 | 7.00371563 | 1.65618901 |
| TRINITY_DN80957_c1_g1_i2 | 190.1495356 | 3.19347438 | 0.755519 |
| TRINITY_DN86485_c0_g1_i32 | 33.69988021 | -9.42234441 | 2.23214881 |
| TRINITY_DN88481_c1_g1_i10 | 26.66883077 | 8.28849608 | 1.96440867 |
| TRINITY_DN84811_c0_g3_i8 | 254.8339834 | -1.82431019 | 0.4326809 |
| TRINITY_DN85968_c2_g2_i9 | 41.37451281 | 6.93443436 | 1.64740579 |

| | | | |
|---|---|---|---|
| TRINITY_DN78091_c0_g1_i7 | 117.182545 | 2.17711933 | 0.517987 |
| TRINITY_DN84318_c0_g1_i3 | 21.66095439 | 8.54483111 | 2.03346132 |
| TRINITY_DN78161_c0_g2_i7 | 124.2361012 | -7.34797153 | 1.75187777 |
| TRINITY_DN86118_c2_g2_i4 | 31.07387247 | -9.3053289 | 2.22144119 |
| TRINITY_DN82851_c1_g1_i4 | 69.12649258 | 5.96971019 | 1.42761312 |
| TRINITY_DN79090_c0_g3_i1 | 239.3640602 | 2.98006548 | 0.71323331 |
| TRINITY_DN87473_c0_g1_i1 | 42.07262474 | -3.95778958 | 0.94752678 |
| TRINITY_DN83803_c3_g2_i1 | 18.75365993 | 7.22726411 | 1.7306611 |
| TRINITY_DN82022_c1_g1_i21 | 24.65715004 | -8.09299394 | 1.93983115 |
| TRINITY_DN83302_c3_g1_i8 | 180.705212 | -4.7655442 | 1.14203136 |
| TRINITY_DN87448_c1_g2_i22 | 18.55475632 | -8.37222833 | 2.00783575 |
| TRINITY_DN81772_c0_g3_i2 | 71.52790631 | 7.26273728 | 1.74199421 |
| TRINITY_DN82559_c0_g2_i7 | 88.11024223 | 7.1409732 | 1.71461868 |
| TRINITY_DN80166_c1_g1_i4 | 75.0194039 | 5.53070147 | 1.32852954 |
| TRINITY_DN82770_c0_g6_i1 | 50.76118927 | -6.85963927 | 1.64931933 |
| TRINITY_DN88846_c3_g2_i6 | 1912.977309 | 2.48407133 | 0.59743853 |
| TRINITY_DN88726_c0_g1_i1 | 46.95886626 | 3.17380469 | 0.76464178 |
| TRINITY_DN78311_c0_g1_i5 | 39.45623035 | -7.46039368 | 1.79864305 |
| TRINITY_DN83180_c0_g2_i21 | 41.57386212 | 8.27296322 | 1.99415715 |
| TRINITY_DN81334_c3_g1_i7 | 27.69642314 | -7.86899891 | 1.89596169 |
| TRINITY_DN84132_c2_g1_i6 | 48.5371883 | 6.86165978 | 1.65420887 |
| TRINITY_DN84879_c0_g1_i7 | 107.1601584 | -4.24875414 | 1.02324041 |
| TRINITY_DN80545_c0_g2_i4 | 37.86877092 | -8.07052667 | 1.94697051 |
| TRINITY_DN86918_c1_g2_i10 | 29.98086066 | 8.19505525 | 1.97725093 |
| TRINITY_DN87466_c4_g4_i2 | 153.5788886 | 3.39886723 | 0.82021641 |
| TRINITY_DN88782_c1_g4_i1 | 5903.145964 | 3.69852718 | 0.8934323 |
| TRINITY_DN79235_c0_g1_i1 | 1296.406667 | 2.66051385 | 0.64325693 |
| TRINITY_DN82638_c1_g3_i4 | 21.10388388 | -7.05402813 | 1.70764693 |
| TRINITY_DN83486_c0_g1_i14 | 209.5140378 | -7.03531524 | 1.70388168 |
| TRINITY_DN86196_c4_g1_i1 | 195.0080773 | -3.66093091 | 0.8872805 |
| TRINITY_DN85879_c0_g2_i13 | 47.4679645 | 7.1701942 | 1.73746578 |
| TRINITY_DN84046_c3_g2_i4 | 57.78840005 | 6.7007543 | 1.6239225 |
| TRINITY_DN81285_c2_g2_i11 | 146.6866913 | -3.01183175 | 0.73044385 |
| TRINITY_DN85899_c1_g1_i5 | 46.43265441 | -19.7554787 | 4.7913221 |
| TRINITY_DN80281_c1_g2_i1 | 236.9760977 | 2.06149617 | 0.50062906 |
| TRINITY_DN88636_c1_g2_i7 | 29.46146645 | -7.21696002 | 1.75270616 |

| | | | |
|---|---|---|---|
| TRINITY_DN80871_c0_g2_i13 | 118.0272945 | -2.36179582 | 0.57368681 |
| TRINITY_DN75673_c0_g1_i1 | 34.81596604 | 4.05701643 | 0.9866116 |
| TRINITY_DN83389_c1_g2_i6 | 1082.177979 | 1.6068748 | 0.39078508 |
| TRINITY_DN85768_c0_g1_i2 | 26.54073521 | 7.07450795 | 1.72104251 |
| TRINITY_DN78803_c2_g1_i5 | 21.27127946 | 7.64817485 | 1.86134511 |
| TRINITY_DN84811_c0_g3_i14 | 32.43994271 | -8.81318941 | 2.14804044 |
| TRINITY_DN77798_c0_g1_i2 | 32.16507745 | -9.35509218 | 2.28070778 |
| TRINITY_DN76141_c0_g1_i4 | 27.83478834 | -7.57048005 | 1.84728544 |
| TRINITY_DN84132_c2_g1_i2 | 19.70312739 | 7.16771906 | 1.74945558 |
| TRINITY_DN86952_c1_g1_i13 | 28.36511868 | 7.03418887 | 1.71852688 |
| TRINITY_DN88726_c1_g1_i8 | 77.42061762 | -5.65801174 | 1.38391861 |
| TRINITY_DN83389_c1_g1_i8 | 105.7462429 | 7.70878862 | 1.88859627 |
| TRINITY_DN85880_c2_g2_i4 | 39.18071578 | 6.99881122 | 1.71598594 |
| TRINITY_DN86743_c1_g1_i6 | 20.77659097 | -8.36961106 | 2.05576817 |
| TRINITY_DN78486_c0_g1_i14 | 1811.216268 | -2.12175618 | 0.52154277 |
| TRINITY_DN78493_c1_g2_i12 | 93.3405806 | -6.98783967 | 1.71865168 |
| TRINITY_DN83651_c2_g2_i11 | 31.2934613 | -5.36391713 | 1.3197318 |
| TRINITY_DN80488_c0_g3_i2 | 12.93806006 | 7.41780412 | 1.82491507 |
| TRINITY_DN84974_c1_g1_i6 | 23.35160919 | -5.5756392 | 1.37277995 |
| TRINITY_DN81556_c1_g1_i4 | 86.38925157 | -2.73490146 | 0.67332576 |
| TRINITY_DN85567_c1_g1_i3 | 19.4767827 | -7.10560449 | 1.74900512 |
| TRINITY_DN77893_c0_g1_i8 | 45.89263307 | 6.82361182 | 1.68095453 |
| TRINITY_DN86081_c5_g1_i4 | 137.8530607 | 2.92965624 | 0.72402968 |
| TRINITY_DN87235_c0_g1_i17 | 152.4947062 | 3.48879558 | 0.86231514 |
| TRINITY_DN77563_c1_g5_i6 | 127.8786849 | -7.06790473 | 1.74818535 |
| TRINITY_DN81087_c0_g1_i7 | 1605.503564 | 6.36605031 | 1.5752164 |
| TRINITY_DN82764_c2_g1_i2 | 109.2158935 | -19.1642785 | 4.74403301 |
| TRINITY_DN82055_c0_g4_i12 | 67.94646425 | 6.70022112 | 1.65989573 |
| TRINITY_DN87529_c3_g4_i6 | 118.6129259 | 2.25516184 | 0.55857641 |
| TRINITY_DN79244_c2_g4_i1 | 26.50240188 | -7.16313912 | 1.77516027 |
| TRINITY_DN84055_c1_g4_i3 | 60.40652611 | 6.77527937 | 1.67974567 |
| TRINITY_DN82599_c2_g2_i1 | 25.9705265 | 8.14045098 | 2.02156734 |
| TRINITY_DN80143_c3_g3_i1 | 38.29020086 | 6.86413996 | 1.70733151 |
| TRINITY_DN86896_c0_g1_i14 | 43.20066596 | 7.03795176 | 1.7502547 |
| TRINITY_DN81180_c2_g4_i4 | 363.8936269 | -4.08572749 | 1.01729424 |
| TRINITY_DN86631_c1_g2_i5 | 93.49256339 | -6.96149824 | 1.73484749 |

| | | | |
|---|---|---|---|
| TRINITY_DN76824_c3_g3_i5 | 20.68919496 | 6.89832362 | 1.72041708 |
| TRINITY_DN83059_c0_g1_i6 | 19.14978162 | -4.34637381 | 1.08670307 |
| TRINITY_DN78289_c1_g1_i2 | 31.65239245 | 8.2223339 | 2.05702622 |
| TRINITY_DN84040_c1_g2_i7 | 148.598584 | 2.38649251 | 0.59736247 |
| TRINITY_DN84614_c6_g1_i3 | 14.08989721 | -7.38559896 | 1.85031498 |
| TRINITY_DN84184_c0_g1_i5 | 39.84379069 | -6.66396219 | 1.67010713 |
| TRINITY_DN88365_c5_g2_i3 | 116.1014658 | -3.5763283 | 0.89657504 |
| TRINITY_DN83874_c1_g1_i8 | 78.34675787 | 19.1229185 | 4.79827848 |
| TRINITY_DN80159_c0_g3_i6 | 51.08304206 | -6.63376167 | 1.66616996 |
| TRINITY_DN88151_c1_g1_i2 | 19.55848585 | -7.90346166 | 1.98508107 |
| TRINITY_DN84561_c2_g2_i4 | 15.09204741 | -7.72986822 | 1.94368341 |
| TRINITY_DN86227_c0_g2_i16 | 36.38028168 | 6.50928132 | 1.63646298 |
| TRINITY_DN87049_c0_g1_i25 | 16.58252487 | 7.00152301 | 1.76218933 |
| TRINITY_DN79388_c1_g1_i12 | 16.92212783 | 7.31551115 | 1.84262875 |
| TRINITY_DN84440_c1_g1_i6 | 31.07488521 | -7.02286421 | 1.76877155 |
| TRINITY_DN81930_c1_g1_i5 | 227.0944172 | 2.54277695 | 0.64034114 |
| TRINITY_DN87154_c2_g2_i23 | 69.06115978 | 4.46327921 | 1.12361183 |
| TRINITY_DN82310_c0_g2_i1 | 18.94720456 | 7.0065331 | 1.76337025 |
| TRINITY_DN81605_c0_g1_i5 | 47.43622359 | 9.17655085 | 2.3122323 |
| TRINITY_DN84511_c3_g1_i4 | 416.2520658 | 1.57189587 | 0.39614577 |
| TRINITY_DN87294_c0_g1_i1 | 42.14471547 | 3.58118308 | 0.90353932 |
| TRINITY_DN88214_c1_g2_i1 | 27.21848338 | 7.33075866 | 1.8513515 |
| TRINITY_DN88732_c1_g1_i10 | 315.1999594 | -2.51184474 | 0.63451566 |
| TRINITY_DN87956_c3_g3_i2 | 43.10928134 | 3.83221173 | 0.96792224 |
| TRINITY_DN79059_c2_g3_i7 | 80.65362573 | -18.9956566 | 4.79586916 |
| TRINITY_DN87181_c0_g3_i12 | 408.655634 | -6.08292769 | 1.53833699 |
| TRINITY_DN81982_c1_g1_i2 | 61.66993905 | 6.78704855 | 1.71766079 |
| TRINITY_DN84543_c3_g1_i18 | 13.12578427 | 7.25425672 | 1.83630136 |
| TRINITY_DN80746_c0_g1_i14 | 86.38067506 | -6.27235245 | 1.58876637 |
| TRINITY_DN84141_c0_g1_i13 | 58.23032234 | 6.569501 | 1.66497577 |
| TRINITY_DN82424_c0_g1_i6 | 49.99174722 | 6.47199429 | 1.64002134 |
| TRINITY_DN87622_c2_g2_i7 | 27.32009629 | -6.8679804 | 1.74225381 |
| TRINITY_DN81673_c0_g2_i4 | 26.58216649 | -6.62859204 | 1.68204175 |
| TRINITY_DN80036_c3_g3_i1 | 19.02643541 | -8.4467988 | 2.14342445 |
| TRINITY_DN83688_c1_g4_i3 | 85.0331044 | 4.31169039 | 1.09463042 |
| TRINITY_DN84730_c0_g2_i3 | 28.37920181 | -7.44202813 | 1.8908473 |

| | | | |
|---|---|---|---|
| TRINITY_DN88636_c1_g1_i12 | 81.62443864 | 9.53364758 | 2.42531478 |
| TRINITY_DN83571_c1_g1_i2 | 55.60065611 | 2.83795291 | 0.72278593 |
| TRINITY_DN87924_c1_g2_i1 | 133.9063536 | -6.12974947 | 1.56385612 |
| TRINITY_DN82733_c0_g1_i6 | 30.30313252 | -9.10073638 | 2.32330575 |
| TRINITY_DN81692_c2_g2_i1 | 144.673982 | 9.17737327 | 2.34558568 |
| TRINITY_DN82857_c1_g2_i15 | 85.61879986 | 6.65626361 | 1.70232496 |
| TRINITY_DN87516_c0_g2_i2 | 17.33415292 | 7.13444168 | 1.82462517 |
| TRINITY_DN78945_c0_g1_i1 | 428.4087407 | -1.51232474 | 0.38666251 |
| TRINITY_DN82811_c0_g2_i5 | 30.8078796 | -4.97304038 | 1.27356374 |
| TRINITY_DN79902_c0_g2_i5 | 16.76099229 | -7.15852204 | 1.83317849 |
| TRINITY_DN88199_c2_g2_i9 | 83.18174258 | 4.20766656 | 1.07836263 |
| TRINITY_DN84279_c0_g1_i7 | 289.2655271 | 8.73208599 | 2.23876664 |
| TRINITY_DN84754_c0_g1_i11 | 101.6884789 | -2.08272531 | 0.53437789 |
| TRINITY_DN85445_c1_g2_i3 | 18.67458181 | -8.5238034 | 2.18853116 |
| TRINITY_DN82461_c5_g1_i4 | 80.463257 | 2.80455132 | 0.72170635 |
| TRINITY_DN88032_c0_g1_i2 | 88.5633091 | 9.60290884 | 2.47148358 |
| TRINITY_DN79320_c0_g11_i1 | 30.828924 | 8.62197729 | 2.22412587 |
| TRINITY_DN78771_c2_g2_i2 | 17.69606482 | 7.62692764 | 1.96851388 |
| TRINITY_DN88618_c2_g1_i1 | 115.0107071 | 2.7733902 | 0.71689681 |
| TRINITY_DN87044_c0_g1_i6 | 14.54710683 | -8.15348061 | 2.10908171 |
| TRINITY_DN79853_c1_g1_i2 | 28.95856942 | -9.20358181 | 2.38061922 |
| TRINITY_DN87245_c1_g2_i3 | 40.52720145 | 7.1723906 | 1.86157913 |
| TRINITY_DN79209_c1_g1_i8 | 26.56014427 | 7.49331326 | 1.94620871 |
| TRINITY_DN81551_c1_g1_i11 | 28.13765078 | 6.5148806 | 1.69331423 |
| TRINITY_DN78516_c0_g5_i1 | 43.29422242 | 6.47287433 | 1.68237649 |
| TRINITY_DN85636_c0_g1_i19 | 116.1607501 | 7.57663551 | 1.96971515 |
| TRINITY_DN86626_c1_g6_i2 | 239.2226825 | 2.09520258 | 0.54515086 |
| TRINITY_DN82831_c0_g1_i8 | 31.28577592 | 8.78805464 | 2.28817719 |
| TRINITY_DN83176_c2_g1_i10 | 101.4233225 | 8.94552303 | 2.3307834 |
| TRINITY_DN82802_c0_g2_i11 | 230.4149943 | 2.20547162 | 0.57464866 |
| TRINITY_DN84265_c1_g2_i6 | 85.50966952 | -17.4739083 | 4.55577646 |
| TRINITY_DN88675_c1_g1_i25 | 42.09934664 | 8.09317262 | 2.11207729 |
| TRINITY_DN82882_c2_g2_i16 | 81.37792613 | -3.1446508 | 0.82033194 |
| TRINITY_DN80652_c2_g1_i3 | 57.88203152 | 6.61156435 | 1.72519399 |
| TRINITY_DN81631_c1_g1_i3 | 155.3317149 | -8.86857845 | 2.31455259 |
| TRINITY_DN79278_c0_g2_i6 | 35.03421614 | 6.51993047 | 1.70234925 |

| | | | |
|---|---|---|---|
| TRINITY_DN82418_c0_g1_i5 | 48.9462248 | 6.6164401 | 1.72871065 |
| TRINITY_DN86788_c0_g1_i1 | 72.79118264 | -17.2785135 | 4.5175464 |
| TRINITY_DN85962_c1_g1_i6 | 64.12930908 | 9.32406651 | 2.43904479 |
| TRINITY_DN86905_c0_g4_i4 | 68.33671957 | 6.19347703 | 1.62097228 |
| TRINITY_DN88465_c3_g9_i5 | 723.2296323 | 2.93586406 | 0.76848865 |
| TRINITY_DN87078_c0_g2_i3 | 12.40058313 | -7.28127479 | 1.90825992 |
| TRINITY_DN81434_c0_g1_i12 | 81.34606496 | 7.11314082 | 1.86438238 |
| TRINITY_DN78013_c1_g1_i3 | 17.5421927 | -8.18100567 | 2.14414788 |
| TRINITY_DN84461_c2_g1_i3 | 20.82778456 | 7.06648541 | 1.85148333 |
| TRINITY_DN85514_c1_g1_i9 | 134.5576319 | -8.70116221 | 2.28259064 |
| TRINITY_DN79899_c2_g1_i5 | 143.8479662 | -6.53723523 | 1.7163173 |
| TRINITY_DN82236_c2_g2_i2 | 107.3463093 | -6.45742741 | 1.69594683 |
| TRINITY_DN88618_c2_g1_i12 | 214.0876964 | 2.78130472 | 0.73051981 |
| TRINITY_DN82353_c0_g1_i12 | 120.4756536 | -2.47053718 | 0.64978331 |
| TRINITY_DN86234_c0_g1_i3 | 15.80523378 | 6.90021098 | 1.81867935 |
| TRINITY_DN84349_c0_g1_i7 | 43.17410966 | 6.65629535 | 1.7543456 |
| TRINITY_DN88307_c1_g1_i16 | 17.1278099 | -8.2529535 | 2.17695496 |
| TRINITY_DN78909_c2_g1_i2 | 219.7266029 | 2.96060919 | 0.78110533 |
| TRINITY_DN77709_c1_g1_i5 | 52.56001825 | 6.70506492 | 1.76977209 |
| TRINITY_DN82634_c1_g2_i2 | 616.9740468 | 1.88123987 | 0.4964721 |
| TRINITY_DN77618_c2_g1_i1 | 84.27109662 | 6.31130058 | 1.66729786 |
| TRINITY_DN77456_c2_g4_i7 | 275.7400461 | 2.45454111 | 0.64912726 |
| TRINITY_DN82465_c0_g2_i4 | 23.45009089 | 7.07195151 | 1.87014135 |
| TRINITY_DN76711_c0_g1_i1 | 29.77548192 | -7.6165413 | 2.01441629 |
| TRINITY_DN83408_c3_g1_i3 | 26.96625534 | 6.65009104 | 1.75951846 |
| TRINITY_DN88761_c5_g2_i11 | 31.38133591 | -9.31960651 | 2.46851436 |
| TRINITY_DN87416_c0_g2_i14 | 31.23121969 | 4.87148803 | 1.2922842 |
| TRINITY_DN81990_c1_g1_i7 | 206.7751069 | 3.84854663 | 1.02255995 |
| TRINITY_DN83157_c0_g11_i1 | 49.6961023 | -2.99235253 | 0.79496787 |
| TRINITY_DN86165_c1_g1_i6 | 536.212583 | -1.96721442 | 0.52258125 |
| TRINITY_DN85900_c0_g4_i7 | 35.65286529 | 6.31448737 | 1.67839823 |
| TRINITY_DN83244_c3_g5_i1 | 363.6760966 | 1.98333156 | 0.52781411 |
| TRINITY_DN81967_c1_g2_i2 | 36.97877268 | -6.78100618 | 1.80465662 |
| TRINITY_DN87694_c0_g1_i17 | 76.34178399 | 7.50504944 | 1.99843701 |
| TRINITY_DN77909_c1_g1_i3 | 13.97194831 | -7.37605072 | 1.96471287 |
| TRINITY_DN84032_c1_g1_i3 | 25.51477766 | -9.02094678 | 2.40199474 |

| | | | |
|---|---|---|---|
| TRINITY_DN80986_c1_g4_i4 | 147.8731339 | -5.9190818 | 1.57650058 |
| TRINITY_DN85140_c0_g1_i11 | 18.44891232 | -7.90914336 | 2.1078313 |
| TRINITY_DN78144_c1_g3_i1 | 52.05022084 | 7.10299958 | 1.89340028 |
| TRINITY_DN78993_c2_g4_i4 | 455.6012338 | 6.75830655 | 1.80183772 |
| TRINITY_DN86606_c1_g1_i9 | 38.39765447 | 9.05293379 | 2.41473319 |
| TRINITY_DN74580_c0_g1_i1 | 201.3965284 | -3.36903153 | 0.89946809 |
| TRINITY_DN87499_c0_g1_i3 | 33.97273503 | -6.5271815 | 1.74315702 |
| TRINITY_DN86717_c2_g3_i4 | 43.52181424 | -3.11732337 | 0.83318151 |
| TRINITY_DN86273_c4_g1_i17 | 18.05766233 | 8.68078404 | 2.32043754 |
| TRINITY_DN79063_c1_g1_i9 | 107.2885756 | 9.8661526 | 2.63912365 |
| TRINITY_DN88087_c3_g1_i2 | 31.26061454 | 9.45608718 | 2.52887051 |
| TRINITY_DN79434_c1_g2_i1 | 274.0076676 | 8.19334731 | 2.19146186 |
| TRINITY_DN85810_c3_g4_i10 | 25.40598186 | -9.01477897 | 2.41227916 |
| TRINITY_DN85326_c0_g2_i5 | 14.85387458 | -6.73106197 | 1.80350096 |
| TRINITY_DN80732_c0_g1_i15 | 134.6871057 | 6.55595499 | 1.75702528 |
| TRINITY_DN79786_c0_g2_i6 | 161.1873488 | 5.85913773 | 1.57067117 |
| TRINITY_DN79722_c0_g1_i3 | 13.30939562 | 8.22611559 | 2.20486002 |
| TRINITY_DN79431_c0_g1_i17 | 22.34785828 | 6.47151361 | 1.7357673 |
| TRINITY_DN82522_c1_g3_i5 | 79.46626626 | 8.81341557 | 2.36642769 |
| TRINITY_DN82575_c1_g1_i13 | 35.22260254 | 4.50131501 | 1.20871185 |
| TRINITY_DN80969_c1_g1_i3 | 28.83896573 | 8.70564436 | 2.33875478 |
| TRINITY_DN79513_c1_g1_i2 | 33.59792904 | -6.43983107 | 1.73119562 |
| TRINITY_DN87726_c0_g2_i12 | 273.1400333 | 3.64647502 | 0.98042024 |
| TRINITY_DN80150_c1_g2_i6 | 135.8131182 | 3.46103754 | 0.9310201 |
| TRINITY_DN85941_c0_g2_i1 | 33.54756112 | -8.71829867 | 2.34502073 |
| TRINITY_DN76436_c0_g1_i5 | 14.795218 | 7.25326397 | 1.95273406 |
| TRINITY_DN84469_c2_g9_i4 | 121.891449 | 2.65629566 | 0.71562424 |
| TRINITY_DN88389_c2_g2_i5 | 17.22175938 | -6.7066952 | 1.80650505 |
| TRINITY_DN82235_c5_g2_i2 | 129.475183 | 8.62357012 | 2.32308368 |
| TRINITY_DN85467_c0_g1_i3 | 36.84548591 | 8.55268586 | 2.30674974 |
| TRINITY_DN78013_c1_g1_i11 | 16.67851061 | -7.43805159 | 2.00631729 |
| TRINITY_DN86197_c3_g5_i1 | 21.69818193 | -7.07373976 | 1.90799215 |
| TRINITY_DN83360_c2_g1_i19 | 17.52944509 | 7.72212714 | 2.08397597 |
| TRINITY_DN80144_c1_g4_i12 | 35.85918223 | -6.62516956 | 1.78972841 |
| TRINITY_DN80951_c0_g1_i8 | 24.7459448 | -8.97685794 | 2.42633286 |
| TRINITY_DN85380_c2_g1_i4 | 586.3006644 | 1.4626737 | 0.39530918 |

| | | | |
|---|---|---|---|
| TRINITY_DN84424_c2_g1_i1 | 28.49735246 | -7.18937746 | 1.94423341 |
| TRINITY_DN88032_c0_g1_i15 | 28.40401084 | -8.92427471 | 2.41484979 |
| TRINITY_DN84587_c1_g1_i2 | 44.06146153 | -9.5047645 | 2.57348121 |
| TRINITY_DN86812_c2_g2_i6 | 19.63351027 | -6.47883669 | 1.75415381 |
| TRINITY_DN79374_c2_g1_i2 | 51.70411725 | 6.32965765 | 1.71469398 |
| TRINITY_DN87016_c1_g2_i33 | 24.22605732 | -8.94620304 | 2.42358892 |
| TRINITY_DN86918_c1_g2_i15 | 31.13101577 | 8.12214735 | 2.20086537 |
| TRINITY_DN81861_c1_g1_i16 | 21.92521226 | -7.53447735 | 2.04555017 |
| TRINITY_DN86524_c1_g1_i16 | 53.99076003 | -2.51797678 | 0.68349636 |
| TRINITY_DN88109_c4_g2_i3 | 27.90404138 | -5.56722596 | 1.51166749 |
| TRINITY_DN84891_c2_g1_i12 | 25.50751536 | -9.02060492 | 2.44986439 |
| TRINITY_DN83413_c1_g1_i6 | 14.70928103 | -7.28862047 | 1.98089861 |
| TRINITY_DN79705_c0_g2_i7 | 91.47840114 | 2.93300052 | 0.79751317 |
| TRINITY_DN88066_c1_g1_i6 | 29.53500669 | -6.79925329 | 1.84887523 |
| TRINITY_DN80152_c2_g2_i5 | 19.50269203 | 8.5932338 | 2.3383696 |
| TRINITY_DN88136_c2_g8_i2 | 406.0798287 | 2.32052692 | 0.63224112 |
| TRINITY_DN79430_c0_g1_i17 | 24.49623123 | -8.96222899 | 2.44335179 |
| TRINITY_DN87926_c0_g1_i9 | 118.46563 | 8.23822515 | 2.2486946 |
| TRINITY_DN84914_c2_g2_i13 | 18.63210743 | -6.85008649 | 1.86934592 |
| TRINITY_DN78356_c0_g1_i5 | 14.07179585 | -7.37463691 | 2.01328795 |
| TRINITY_DN87105_c1_g1_i10 | 131.0118133 | 2.44248677 | 0.6666167 |
| TRINITY_DN77091_c0_g1_i2 | 14.76897345 | -7.4860903 | 2.04351683 |
| TRINITY_DN84332_c1_g3_i11 | 32.48668139 | -9.36942277 | 2.55738008 |
| TRINITY_DN85006_c1_g1_i3 | 314.5029973 | 7.89021393 | 2.15357738 |
| TRINITY_DN88407_c0_g1_i1 | 115.1448024 | -4.5632597 | 1.24746106 |
| TRINITY_DN79777_c1_g1_i3 | 12.99030772 | 7.99124348 | 2.18666641 |

## and buds of ON- *versus* OFF-crop trees

[illegible]*, Mehrshad Zeinalabedini[b] and Seyed Alireza Salami[a]

## ysis of olive's leaf samples ON- *vs.* OFF-trees

| stat | pvalue | padj |
|---|---|---|
| -9.808094177 | 1.04E-22 | 9.14E-18 |
| -9.569834377 | 1.07E-21 | 4.71E-17 |
| 8.429026117 | 3.49E-17 | 1.02E-12 |
| 7.608118248 | 2.78E-14 | 6.12E-10 |
| -7.532363725 | 4.98E-14 | 8.77E-10 |
| -7.344943379 | 2.06E-13 | 2.59E-09 |
| 7.359264777 | 1.85E-13 | 2.59E-09 |
| 7.32161348 | 2.45E-13 | 2.69E-09 |
| -7.192922014 | 6.34E-13 | 5.58E-09 |
| 7.194742565 | 6.26E-13 | 5.58E-09 |
| 7.140241207 | 9.32E-13 | 7.45E-09 |
| 6.868833131 | 6.47E-12 | 4.75E-08 |
| 6.789384707 | 1.13E-11 | 7.62E-08 |
| -6.757517605 | 1.40E-11 | 8.82E-08 |
| 6.608670492 | 3.88E-11 | 2.27E-07 |
| -6.513635363 | 7.34E-11 | 4.03E-07 |
| 6.445477117 | 1.15E-10 | 5.96E-07 |
| 6.34305702 | 2.25E-10 | 1.04E-06 |
| 6.349776447 | 2.16E-10 | 1.04E-06 |
| -6.239232654 | 4.40E-10 | 1.93E-06 |
| -6.222234392 | 4.90E-10 | 2.05E-06 |
| -6.14524625 | 7.98E-10 | 3.19E-06 |
| 6.137218804 | 8.40E-10 | 3.21E-06 |
| 6.126525779 | 8.98E-10 | 3.29E-06 |
| 6.101564243 | 1.05E-09 | 3.70E-06 |
| 6.048939131 | 1.46E-09 | 4.93E-06 |
| -5.996619783 | 2.01E-09 | 6.57E-06 |
| 5.971998549 | 2.34E-09 | 7.37E-06 |
| 5.928731051 | 3.05E-09 | 9.26E-06 |
| 5.914810365 | 3.32E-09 | 9.53E-06 |
| -5.91310759 | 3.36E-09 | 9.53E-06 |
| 5.898040251 | 3.68E-09 | 1.01E-05 |

| | | |
|---|---|---|
| -5.892387347 | 3.81E-09 | 1.02E-05 |
| 5.858526658 | 4.67E-09 | 1.14E-05 |
| -5.86187433 | 4.58E-09 | 1.14E-05 |
| 5.867514433 | 4.42E-09 | 1.14E-05 |
| 5.778874992 | 7.52E-09 | 1.79E-05 |
| -5.771698518 | 7.85E-09 | 1.82E-05 |
| 5.748076603 | 9.03E-09 | 2.04E-05 |
| 5.716898136 | 1.08E-08 | 2.39E-05 |
| 5.703034262 | 1.18E-08 | 2.53E-05 |
| -5.599074181 | 2.15E-08 | 4.51E-05 |
| 5.586522742 | 2.32E-08 | 4.74E-05 |
| 5.57067803 | 2.54E-08 | 5.07E-05 |
| 5.532642129 | 3.15E-08 | 6.03E-05 |
| -5.534481279 | 3.12E-08 | 6.03E-05 |
| 5.523356673 | 3.33E-08 | 6.23E-05 |
| 5.517895701 | 3.43E-08 | 6.29E-05 |
| 5.455319803 | 4.89E-08 | 8.78E-05 |
| 5.432134224 | 5.57E-08 | 9.80E-05 |
| 5.373867388 | 7.71E-08 | 0.000132968 |
| 5.36988581 | 7.88E-08 | 0.000133323 |
| -5.337451022 | 9.43E-08 | 0.000156502 |
| 5.325431125 | 1.01E-07 | 0.000164117 |
| -5.31526447 | 1.07E-07 | 0.000170394 |
| 5.277394459 | 1.31E-07 | 0.000200825 |
| 5.281563249 | 1.28E-07 | 0.000200825 |
| 5.275534687 | 1.32E-07 | 0.000200825 |
| -5.25071854 | 1.52E-07 | 0.000225964 |
| -5.239504963 | 1.61E-07 | 0.000236132 |
| 5.222500712 | 1.77E-07 | 0.000252839 |
| 5.220805074 | 1.78E-07 | 0.000252839 |
| -5.21291168 | 1.86E-07 | 0.000259654 |
| -5.166769688 | 2.38E-07 | 0.000327471 |
| -5.149228098 | 2.62E-07 | 0.000349893 |
| 5.145780295 | 2.66E-07 | 0.000349893 |
| 5.145899476 | 2.66E-07 | 0.000349893 |
| 5.138076638 | 2.78E-07 | 0.000357187 |

| | | |
|---|---|---|
| 5.1363788 | 2.80E-07 | 0.000357187 |
| 5.123539167 | 3.00E-07 | 0.000376937 |
| 5.110833378 | 3.21E-07 | 0.000397515 |
| 5.107872803 | 3.26E-07 | 0.000398185 |
| 5.101082369 | 3.38E-07 | 0.000407087 |
| 5.080359109 | 3.77E-07 | 0.000447968 |
| -5.019013743 | 5.19E-07 | 0.000602092 |
| -5.018775737 | 5.20E-07 | 0.000602092 |
| -5.01493168 | 5.31E-07 | 0.000606278 |
| -5.006248405 | 5.55E-07 | 0.000626131 |
| 4.994770138 | 5.89E-07 | 0.00065613 |
| -4.980976957 | 6.33E-07 | 0.000695865 |
| -4.973396476 | 6.58E-07 | 0.000714714 |
| 4.959543572 | 7.07E-07 | 0.000731487 |
| 4.966439238 | 6.82E-07 | 0.000731487 |
| 4.963025523 | 6.94E-07 | 0.000731487 |
| 4.960187913 | 7.04E-07 | 0.000731487 |
| 4.951409835 | 7.37E-07 | 0.000739772 |
| 4.950609557 | 7.40E-07 | 0.000739772 |
| -4.950922181 | 7.39E-07 | 0.000739772 |
| 4.939388813 | 7.84E-07 | 0.000774829 |
| 4.929827448 | 8.23E-07 | 0.000804688 |
| 4.923565909 | 8.50E-07 | 0.000812818 |
| 4.924471752 | 8.46E-07 | 0.000812818 |
| -4.920166269 | 8.65E-07 | 0.000818171 |
| 4.912038624 | 9.01E-07 | 0.000834881 |
| -4.914034404 | 8.92E-07 | 0.000834881 |
| -4.908943026 | 9.16E-07 | 0.000839331 |
| 4.901574132 | 9.51E-07 | 0.000862458 |
| 4.896142231 | 9.77E-07 | 0.000869793 |
| -4.895899163 | 9.79E-07 | 0.000869793 |
| 4.887204023 | 1.02E-06 | 0.000899996 |
| 4.87751557 | 1.07E-06 | 0.000935974 |
| 4.874233304 | 1.09E-06 | 0.000942339 |
| -4.868508011 | 1.12E-06 | 0.000960632 |
| 4.860245867 | 1.17E-06 | 0.000991975 |

| | | |
|---|---|---|
| 4.856190018 | 1.20E-06 | 0.001002857 |
| -4.845982691 | 1.26E-06 | 0.001045866 |
| 4.832586963 | 1.35E-06 | 0.001108328 |
| 4.824957736 | 1.40E-06 | 0.001130477 |
| -4.825096715 | 1.40E-06 | 0.001130477 |
| 4.823131966 | 1.41E-06 | 0.001130507 |
| 4.801350296 | 1.58E-06 | 0.00123821 |
| 4.802447551 | 1.57E-06 | 0.00123821 |
| -4.796816626 | 1.61E-06 | 0.001244335 |
| -4.797966624 | 1.60E-06 | 0.001244335 |
| 4.790191448 | 1.67E-06 | 0.00127495 |
| 4.772191074 | 1.82E-06 | 0.001358945 |
| -4.772443185 | 1.82E-06 | 0.001358945 |
| -4.775325718 | 1.79E-06 | 0.001358945 |
| 4.769919257 | 1.84E-06 | 0.001362812 |
| -4.755800946 | 1.98E-06 | 0.001449433 |
| 4.751032469 | 2.02E-06 | 0.001471775 |
| 4.740484389 | 2.13E-06 | 0.001537806 |
| 4.734405865 | 2.20E-06 | 0.001571732 |
| 4.723299198 | 2.32E-06 | 0.001646706 |
| 4.719587704 | 2.36E-06 | 0.00166362 |
| 4.697176339 | 2.64E-06 | 0.001842187 |
| 4.665783228 | 3.07E-06 | 0.002130197 |
| -4.638234055 | 3.51E-06 | 0.002415729 |
| 4.6356899 | 3.56E-06 | 0.002426677 |
| 4.606989858 | 4.09E-06 | 0.002765344 |
| 4.601512606 | 4.19E-06 | 0.00281741 |
| 4.599632994 | 4.23E-06 | 0.002821412 |
| 4.584977071 | 4.54E-06 | 0.002964641 |
| 4.583170626 | 4.58E-06 | 0.002964641 |
| 4.587238784 | 4.49E-06 | 0.002964641 |
| 4.583070355 | 4.58E-06 | 0.002964641 |
| -4.573465 | 4.80E-06 | 0.003081263 |
| -4.557053484 | 5.19E-06 | 0.003307861 |
| 4.551234439 | 5.33E-06 | 0.003376228 |
| 4.54850574 | 5.40E-06 | 0.003395863 |

| | | |
|---|---|---|
| 4.545067979 | 5.49E-06 | 0.003427282 |
| -4.541027517 | 5.60E-06 | 0.003469031 |
| 4.532910623 | 5.82E-06 | 0.003579885 |
| 4.529967652 | 5.90E-06 | 0.003604906 |
| 4.523239966 | 6.09E-06 | 0.003695798 |
| -4.519583011 | 6.20E-06 | 0.003734457 |
| -4.511169523 | 6.45E-06 | 0.003859279 |
| 4.502554274 | 6.71E-06 | 0.003991976 |
| 4.500305051 | 6.79E-06 | 0.003992121 |
| -4.499693903 | 6.81E-06 | 0.003992121 |
| -4.493288326 | 7.01E-06 | 0.004086915 |
| 4.466194167 | 7.96E-06 | 0.004609511 |
| 4.464444424 | 8.03E-06 | 0.004616964 |
| 4.456504846 | 8.33E-06 | 0.004760113 |
| 4.450061579 | 8.58E-06 | 0.004873542 |
| -4.448255953 | 8.66E-06 | 0.004883177 |
| 4.439978011 | 9.00E-06 | 0.00504251 |
| -4.416639903 | 1.00E-05 | 0.005583061 |
| 4.412681817 | 1.02E-05 | 0.005650385 |
| 4.406871067 | 1.05E-05 | 0.005767774 |
| 4.399349146 | 1.09E-05 | 0.005919657 |
| 4.398539712 | 1.09E-05 | 0.005919657 |
| 4.38988157 | 1.13E-05 | 0.006122531 |
| -4.383784547 | 1.17E-05 | 0.006258115 |
| 4.368400482 | 1.25E-05 | 0.006674811 |
| -4.363377779 | 1.28E-05 | 0.006788841 |
| 4.357965784 | 1.31E-05 | 0.006917192 |
| 4.355538541 | 1.33E-05 | 0.006952661 |
| -4.343166336 | 1.40E-05 | 0.00731263 |
| 4.328756069 | 1.50E-05 | 0.007716488 |
| -4.329483033 | 1.49E-05 | 0.007716488 |
| 4.320674969 | 1.56E-05 | 0.007739531 |
| -4.32586784 | 1.52E-05 | 0.007739531 |
| 4.320648983 | 1.56E-05 | 0.007739531 |
| 4.322809868 | 1.54E-05 | 0.007739531 |
| 4.322669003 | 1.54E-05 | 0.007739531 |

| | | |
|---|---|---|
| -4.32049588 | 1.56E-05 | 0.007739531 |
| 4.31539439 | 1.59E-05 | 0.007875958 |
| 4.311194728 | 1.62E-05 | 0.007937874 |
| 4.31239823 | 1.61E-05 | 0.007937874 |
| 4.308451795 | 1.64E-05 | 0.007992537 |
| 4.302124408 | 1.69E-05 | 0.0081791 |
| -4.289589599 | 1.79E-05 | 0.008607334 |
| 4.287358112 | 1.81E-05 | 0.008646976 |
| 4.285553882 | 1.82E-05 | 0.008670336 |
| 4.282767912 | 1.85E-05 | 0.00868575 |
| 4.283536856 | 1.84E-05 | 0.00868575 |
| 4.278116257 | 1.88E-05 | 0.008822041 |
| 4.268902892 | 1.96E-05 | 0.009145742 |
| 4.264743855 | 2.00E-05 | 0.00917218 |
| 4.265411474 | 2.00E-05 | 0.00917218 |
| 4.265196691 | 2.00E-05 | 0.00917218 |
| 4.263093059 | 2.02E-05 | 0.009192354 |
| -4.258859623 | 2.05E-05 | 0.009224769 |
| -4.260074933 | 2.04E-05 | 0.009224769 |
| 4.259451802 | 2.05E-05 | 0.009224769 |
| -4.254236259 | 2.10E-05 | 0.009322303 |
| -4.254588973 | 2.09E-05 | 0.009322303 |
| 4.251110943 | 2.13E-05 | 0.009405859 |
| 4.24585353 | 2.18E-05 | 0.00943943 |
| 4.246844384 | 2.17E-05 | 0.00943943 |
| -4.247691049 | 2.16E-05 | 0.00943943 |
| 4.246295379 | 2.17E-05 | 0.00943943 |
| -4.24219427 | 2.21E-05 | 0.009547671 |
| -4.240679974 | 2.23E-05 | 0.009565429 |
| 4.230892614 | 2.33E-05 | 0.009942828 |
| 4.228814207 | 2.35E-05 | 0.009986635 |
| 4.226861792 | 2.37E-05 | 0.010025216 |
| -4.221199032 | 2.43E-05 | 0.010231261 |
| 4.219333883 | 2.45E-05 | 0.010267139 |
| -4.216294756 | 2.48E-05 | 0.010357099 |
| 4.209305573 | 2.56E-05 | 0.010632318 |

| | | |
|---|---|---|
| 4.203038528 | 2.63E-05 | 0.010873422 |
| 4.202111455 | 2.64E-05 | 0.010873422 |
| -4.194340305 | 2.74E-05 | 0.011200612 |
| -4.188870237 | 2.80E-05 | 0.011420881 |
| 4.18160222 | 2.89E-05 | 0.011737887 |
| 4.178247758 | 2.94E-05 | 0.011857671 |
| -4.176968553 | 2.95E-05 | 0.011865814 |
| 4.176013503 | 2.97E-05 | 0.011865814 |
| -4.172009472 | 3.02E-05 | 0.011967521 |
| -4.172866337 | 3.01E-05 | 0.011967521 |
| -4.169777504 | 3.05E-05 | 0.012007354 |
| 4.169208621 | 3.06E-05 | 0.012007354 |
| 4.164758789 | 3.12E-05 | 0.012189533 |
| 4.163024838 | 3.14E-05 | 0.012228159 |
| -4.159072858 | 3.20E-05 | 0.012386826 |
| 4.15786931 | 3.21E-05 | 0.012397635 |
| 4.150707929 | 3.31E-05 | 0.012623901 |
| -4.147790011 | 3.36E-05 | 0.012623901 |
| 4.14860145 | 3.35E-05 | 0.012623901 |
| -4.150399745 | 3.32E-05 | 0.012623901 |
| 4.148000848 | 3.35E-05 | 0.012623901 |
| -4.15225406 | 3.29E-05 | 0.012623901 |
| -4.145171505 | 3.40E-05 | 0.0126794 |
| 4.144671325 | 3.40E-05 | 0.0126794 |
| 4.143866433 | 3.41E-05 | 0.0126794 |
| 4.139683765 | 3.48E-05 | 0.012858543 |
| 4.13600493 | 3.53E-05 | 0.013011648 |
| -4.130846951 | 3.61E-05 | 0.01325165 |
| -4.128992819 | 3.64E-05 | 0.013303522 |
| -4.12601303 | 3.69E-05 | 0.013311276 |
| 4.126811738 | 3.68E-05 | 0.013311276 |
| 4.126277149 | 3.69E-05 | 0.013311276 |
| -4.123289897 | 3.73E-05 | 0.013366622 |
| -4.123179004 | 3.74E-05 | 0.013366622 |
| 4.117811599 | 3.82E-05 | 0.013571987 |
| -4.117609775 | 3.83E-05 | 0.013571987 |

| | | |
|---|---|---|
| -4.116873113 | 3.84E-05 | 0.013571987 |
| 4.112070482 | 3.92E-05 | 0.01375638 |
| 4.111914415 | 3.92E-05 | 0.01375638 |
| 4.110594548 | 3.95E-05 | 0.013780354 |
| 4.108950463 | 3.97E-05 | 0.013823956 |
| -4.102897342 | 4.08E-05 | 0.014134918 |
| -4.101837274 | 4.10E-05 | 0.014144161 |
| -4.098164729 | 4.16E-05 | 0.014314273 |
| 4.097114071 | 4.18E-05 | 0.014323422 |
| 4.093150323 | 4.26E-05 | 0.014514115 |
| -4.088399213 | 4.34E-05 | 0.014757345 |
| 4.081755705 | 4.47E-05 | 0.015127276 |
| 4.078594737 | 4.53E-05 | 0.015275642 |
| -4.071281571 | 4.68E-05 | 0.015703142 |
| -4.068230469 | 4.74E-05 | 0.015849644 |
| -4.065884749 | 4.79E-05 | 0.015930504 |
| -4.064399386 | 4.82E-05 | 0.015930504 |
| 4.064739363 | 4.81E-05 | 0.015930504 |
| -4.061568053 | 4.87E-05 | 0.015945153 |
| -4.061780525 | 4.87E-05 | 0.015945153 |
| -4.062655046 | 4.85E-05 | 0.015945153 |
| 4.059367271 | 4.92E-05 | 0.016036557 |
| 4.046320619 | 5.20E-05 | 0.016865976 |
| 4.04584752 | 5.21E-05 | 0.016865976 |
| -4.042995046 | 5.28E-05 | 0.017010025 |
| 4.041381441 | 5.31E-05 | 0.017065007 |
| -4.039659601 | 5.35E-05 | 0.017128253 |
| 4.03653132 | 5.42E-05 | 0.017232816 |
| 4.037338132 | 5.41E-05 | 0.017232816 |
| -4.035206977 | 5.46E-05 | 0.017267969 |
| 4.033515005 | 5.49E-05 | 0.017330495 |
| 4.026801781 | 5.65E-05 | 0.017768911 |
| 4.020390829 | 5.81E-05 | 0.018129987 |
| 4.02110147 | 5.79E-05 | 0.018129987 |
| -4.01626918 | 5.91E-05 | 0.018384649 |
| -4.012743654 | 6.00E-05 | 0.018595785 |

| | | |
|---|---|---|
| 4.009680977 | 6.08E-05 | 0.018772525 |
| -3.999596514 | 6.35E-05 | 0.019522142 |
| 3.997194514 | 6.41E-05 | 0.019652512 |
| 3.995049319 | 6.47E-05 | 0.019762451 |
| -3.991536054 | 6.56E-05 | 0.019988173 |
| -3.990140564 | 6.60E-05 | 0.02003681 |
| -3.988877849 | 6.64E-05 | 0.02007453 |
| 3.985370701 | 6.74E-05 | 0.020303598 |
| -3.981443561 | 6.85E-05 | 0.02050279 |
| -3.981430161 | 6.85E-05 | 0.02050279 |
| -3.976917314 | 6.98E-05 | 0.020754461 |
| 3.977652646 | 6.96E-05 | 0.020754461 |
| 3.973195661 | 7.09E-05 | 0.020928715 |
| 3.970149242 | 7.18E-05 | 0.020928715 |
| -3.970475558 | 7.17E-05 | 0.020928715 |
| 3.970972354 | 7.16E-05 | 0.020928715 |
| 3.972260784 | 7.12E-05 | 0.020928715 |
| 3.97337603 | 7.09E-05 | 0.020928715 |
| 3.968697633 | 7.23E-05 | 0.020981724 |
| 3.967973351 | 7.25E-05 | 0.020981724 |
| 3.963505525 | 7.39E-05 | 0.021308394 |
| 3.959679535 | 7.51E-05 | 0.021461952 |
| -3.958680422 | 7.54E-05 | 0.021461952 |
| 3.959214474 | 7.52E-05 | 0.021461952 |
| -3.960837117 | 7.47E-05 | 0.021461952 |
| -3.954223117 | 7.68E-05 | 0.021795434 |
| 3.951332296 | 7.77E-05 | 0.021989505 |
| 3.950471784 | 7.80E-05 | 0.021997987 |
| -3.947938832 | 7.88E-05 | 0.022160947 |
| 3.945703674 | 7.96E-05 | 0.022226689 |
| 3.946286639 | 7.94E-05 | 0.022226689 |
| -3.942009113 | 8.08E-05 | 0.022472379 |
| -3.940801137 | 8.12E-05 | 0.022472379 |
| -3.940796147 | 8.12E-05 | 0.022472379 |
| 3.938946252 | 8.18E-05 | 0.022575341 |
| -3.935816574 | 8.29E-05 | 0.022800133 |

| | | |
|---|---|---|
| 3.930890804 | 8.46E-05 | 0.023199897 |
| 3.926408629 | 8.62E-05 | 0.023562882 |
| -3.919637731 | 8.87E-05 | 0.024159714 |
| -3.91714969 | 8.96E-05 | 0.024334994 |
| 3.912614803 | 9.13E-05 | 0.024720397 |
| 3.910101624 | 9.23E-05 | 0.024752134 |
| 3.910086187 | 9.23E-05 | 0.024752134 |
| -3.911226755 | 9.18E-05 | 0.024752134 |
| -3.904822526 | 9.43E-05 | 0.025143787 |
| -3.904978204 | 9.42E-05 | 0.025143787 |
| 3.901903178 | 9.54E-05 | 0.025372158 |
| 3.900400257 | 9.60E-05 | 0.025453292 |
| -3.897476574 | 9.72E-05 | 0.025685085 |
| -3.894759903 | 9.83E-05 | 0.025896888 |
| 3.886000614 | 0.000101909 | 0.02674587 |
| 3.885483573 | 0.000102126 | 0.02674587 |
| 3.876568957 | 0.00010594 | 0.027662232 |
| 3.87445967 | 0.000106862 | 0.027820358 |
| 3.868604484 | 0.00010946 | 0.028412789 |
| -3.86589129 | 0.000110684 | 0.028562051 |
| -3.866045325 | 0.000110614 | 0.028562051 |
| 3.852852922 | 0.00011675 | 0.030039106 |
| 3.850210523 | 0.000118016 | 0.030276524 |
| 3.847413841 | 0.000119371 | 0.030446585 |
| 3.847458853 | 0.000119349 | 0.030446585 |
| 3.846564061 | 0.000119786 | 0.030464027 |
| 3.84334453 | 0.000121369 | 0.030777683 |
| 3.840635535 | 0.000122716 | 0.031029919 |
| 3.837989843 | 0.000124046 | 0.03119216 |
| 3.837947936 | 0.000124067 | 0.03119216 |
| -3.835549975 | 0.000125284 | 0.031408356 |
| 3.831854386 | 0.000127181 | 0.031549699 |
| -3.833388204 | 0.00012639 | 0.031549699 |
| 3.832359953 | 0.00012692 | 0.031549699 |
| -3.831659949 | 0.000127282 | 0.031549699 |
| 3.829960546 | 0.000128164 | 0.031679141 |

| | | |
|---|---|---|
| 3.827384362 | 0.000129512 | 0.031922761 |
| -3.82475617 | 0.000130902 | 0.032175095 |
| 3.822835296 | 0.000131926 | 0.032336546 |
| 3.820840813 | 0.000132997 | 0.032488589 |
| 3.82030893 | 0.000133285 | 0.032488589 |
| -3.815661958 | 0.000135818 | 0.032794073 |
| 3.815280001 | 0.000136029 | 0.032794073 |
| -3.815504404 | 0.000135905 | 0.032794073 |
| 3.816661632 | 0.000135269 | 0.032794073 |
| -3.81196788 | 0.000137865 | 0.033145936 |
| -3.808873344 | 0.000139601 | 0.033472013 |
| -3.807564781 | 0.000140342 | 0.033503648 |
| 3.807295433 | 0.000140495 | 0.033503648 |
| -3.802093935 | 0.000143478 | 0.034122631 |
| 3.794077813 | 0.000148193 | 0.035054464 |
| 3.794175656 | 0.000148135 | 0.035054464 |
| -3.791053865 | 0.000150009 | 0.03538895 |
| 3.79028163 | 0.000150477 | 0.035404241 |
| 3.788660111 | 0.000151462 | 0.035446531 |
| 3.789215702 | 0.000151124 | 0.035446531 |
| 3.785346765 | 0.000153494 | 0.035826894 |
| 3.781294154 | 0.000156015 | 0.036168097 |
| 3.78150642 | 0.000155882 | 0.036168097 |
| -3.781016542 | 0.000156189 | 0.036168097 |
| 3.779494884 | 0.000157147 | 0.036294317 |
| -3.775390843 | 0.000159757 | 0.036800562 |
| 3.769672354 | 0.000163462 | 0.037555713 |
| 3.763639125 | 0.000167458 | 0.038174897 |
| -3.764117551 | 0.000167138 | 0.038174897 |
| -3.764418326 | 0.000166937 | 0.038174897 |
| 3.762210465 | 0.000168418 | 0.038294475 |
| 3.75763266 | 0.000171528 | 0.038820946 |
| -3.757504945 | 0.000171616 | 0.038820946 |
| 3.755459588 | 0.000173024 | 0.038926387 |
| -3.75426396 | 0.000173852 | 0.038926387 |
| -3.755606379 | 0.000172922 | 0.038926387 |

| | | |
|---|---|---|
| -3.754570019 | 0.000173639 | 0.038926387 |
| -3.752265824 | 0.000175244 | 0.039138457 |
| 3.751451646 | 0.000175814 | 0.039166388 |
| 3.750785364 | 0.000176282 | 0.039171453 |
| 3.749041029 | 0.000177512 | 0.039345523 |
| -3.745582048 | 0.000179976 | 0.03979143 |
| -3.744459877 | 0.000180782 | 0.039869512 |
| -3.741469698 | 0.000182947 | 0.040218974 |
| 3.741011731 | 0.000183281 | 0.040218974 |
| 3.738419987 | 0.00018518 | 0.040334035 |
| 3.739253207 | 0.000184568 | 0.040334035 |
| 3.738758805 | 0.000184931 | 0.040334035 |
| -3.737038029 | 0.000186201 | 0.04045614 |
| -3.732219795 | 0.0001898 | 0.04113653 |
| 3.731280971 | 0.000190509 | 0.041140659 |
| 3.730340159 | 0.000191221 | 0.041140659 |
| 3.73090151 | 0.000190796 | 0.041140659 |
| 3.728330171 | 0.000192753 | 0.041368969 |
| 3.724354486 | 0.000195816 | 0.041871223 |
| 3.724059624 | 0.000196045 | 0.041871223 |
| 3.722341663 | 0.000197384 | 0.042055151 |
| -3.719874864 | 0.000199322 | 0.042360002 |
| 3.719297963 | 0.000199777 | 0.042360002 |
| 3.717468103 | 0.000201229 | 0.042463261 |
| -3.717791735 | 0.000200972 | 0.042463261 |
| 3.714414633 | 0.000203675 | 0.042876429 |
| 3.711858132 | 0.000205743 | 0.043003282 |
| -3.712525009 | 0.000205202 | 0.043003282 |
| 3.712122032 | 0.000205529 | 0.043003282 |
| 3.707678264 | 0.000209168 | 0.043471969 |
| -3.707315703 | 0.000209468 | 0.043471969 |
| -3.707426024 | 0.000209377 | 0.043471969 |
| 3.705478007 | 0.000210992 | 0.043685364 |
| -3.701773705 | 0.000214098 | 0.044224207 |
| -3.69976357 | 0.0002158 | 0.044367658 |
| 3.700075173 | 0.000215536 | 0.044367658 |

| | | |
|---|---|---|
| -3.697795449 | 0.00021748 | 0.044608752 |
| -3.695581706 | 0.000219384 | 0.044894622 |
| -3.693349099 | 0.00022132 | 0.045081114 |
| -3.693425651 | 0.000221253 | 0.045081114 |
| 3.691421167 | 0.000223005 | 0.045235806 |
| -3.691303815 | 0.000223107 | 0.045235806 |
| 3.690433523 | 0.000223872 | 0.045286514 |
| -3.683350065 | 0.000230189 | 0.04633842 |
| -3.683965196 | 0.000229634 | 0.04633842 |
| -3.682837653 | 0.000230652 | 0.04633842 |
| -3.682083365 | 0.000231336 | 0.046369904 |
| -3.679451561 | 0.000233736 | 0.04674456 |
| 3.677682859 | 0.000235362 | 0.046888945 |
| -3.677507921 | 0.000235524 | 0.046888945 |
| 3.674882623 | 0.000237959 | 0.04726678 |
| 3.670319526 | 0.000242247 | 0.048010282 |
| -3.668005987 | 0.000244449 | 0.048337821 |
| 3.663558925 | 0.000248735 | 0.048532818 |
| -3.664429588 | 0.00024789 | 0.048532818 |
| -3.662981688 | 0.000249296 | 0.048532818 |
| 3.664004794 | 0.000248302 | 0.048532818 |
| -3.663336757 | 0.000248951 | 0.048532818 |
| -3.663680206 | 0.000248617 | 0.048532818 |
| 3.663770808 | 0.000248529 | 0.048532818 |
| -3.658037787 | 0.000254154 | 0.049369193 |
| 3.654532515 | 0.000257651 | 0.049938328 |
