# Supplementary material for: Differential expression of genes in olive leaves and buds of ON- versus OFF-crop trees
Source: Sci Rep. 2020 Sep 25;10:15762. doi: 10.1038/s41598-020-72895-7 (PMC7519672; doi:10.1038/s41598-020-72895-7)
Supplement: Supplementary file 2 — Supplementary Table 2. [file 41598_2020_72895_MOESM2_ESM.pdf]

Alternate bearing in olive: Differential expression of genes in leaves and buds

Ebrahim Dastkar[1], Ali Soleimani[1*], Hossein Jafary[2], Juan de Dios Alche[3], Abbas Bahari[4], Mehrsha

Supplementary table S2. Results of gene ontology (GO) analysis of olive's leaf samp

	Tags	SeqName	Description	Length	#Hits
TRUE	[BLASTED,	TRINITY_DN88109_c4_g2_i3	DNA helicase Pif1-like	4656	20
TRUE	[BLASTED,	TRINITY_DN88146_c0_g7_i6	DNA-binding protein REB	2371	20
TRUE	[BLASTED,	TRINITY_DN88136_c2_g8_i2	diacylglycerol O-acyltrans	1218	20
TRUE	[BLASTED,	TRINITY_DN88199_c2_g2_i9	cytochrome b6 (chloropl	1109	20
TRUE	[BLASTED]	TRINITY_DN88151_c1_g1_i2	putative pentatricopepti	3246	20
TRUE	[BLASTED,	TRINITY_DN77909_c1_g1_i3	ribosomal RNA small sub	1839	20
TRUE	[BLASTED,	TRINITY_DN87466_c4_g4_i2	photosystem II cytochror	1401	20
TRUE	[NO-BLAST	TRINITY_DN87473_c0_g1_i1	---NA---	951	
TRUE	[BLASTED,	TRINITY_DN87448_c1_g2_i22	type IV inositol polyphos	3161	20
TRUE	[BLASTED,	TRINITY_DN87499_c0_g1_i3	3-hydroxy-3-methylgluta	1356	20
TRUE	[BLASTED,	TRINITY_DN87416_c0_g2_i14	proline-rich receptor-like	1463	20
TRUE	[BLASTED]	TRINITY_DN88214_c1_g2_i1	WD repeat-containing pr	888	20
TRUE	[BLASTED,	TRINITY_DN88365_c5_g2_i3	caffeic acid 3-O-methyltr	1345	20
TRUE	[BLASTED,	TRINITY_DN88307_c1_g1_i16	tripeptidyl-peptidase 2	2808	20
TRUE	[BLASTED,	TRINITY_DN88389_c2_g2_i5	Retrovirus-related Pol po	5009	20
TRUE	[NO-BLAST	TRINITY_DN80143_c3_g3_i1	---NA---	969	
TRUE	[BLASTED,	TRINITY_DN80150_c1_g2_i6	glyoxylate/succinic semia	1340	20
TRUE	[BLASTED,	TRINITY_DN80159_c0_g3_i6	scarecrow-like protein 1	2118	20
TRUE	[BLASTED]	TRINITY_DN80152_c2_g2_i5	serine carboxypeptidase-	2574	20
TRUE	[BLASTED,	TRINITY_DN80166_c1_g1_i4	receptor-like protein kin	3527	20
TRUE	[BLASTED,	TRINITY_DN80144_c1_g4_i12	beta-ureidopropionase	3175	20
TRUE	[BLASTED,	TRINITY_DN88481_c1_g1_i10	FAD-dependent urate hy	2503	20
TRUE	[NO-BLAST	TRINITY_DN88427_c1_g2_i12	---NA---	1975	
TRUE	[BLASTED,	TRINITY_DN88465_c3_g9_i5	photosystem II 44 kDa pr	1657	20
TRUE	[BLASTED]	TRINITY_DN88407_c0_g1_i1	sacsin	8420	20
TRUE	[BLASTED]	TRINITY_DN88496_c1_g2_i16	uncharacterized WD repe	1936	20
TRUE	[BLASTED,	TRINITY_DN86165_c1_g1_i6	calreticulin-3-like isoforr	4258	20
TRUE	[BLASTED,	TRINITY_DN86147_c0_g1_i15	ubiquitin receptor RAD2	1416	20
TRUE	[BLASTED,	TRINITY_DN86196_c4_g1_i1	xyloglucan endotransglu	1520	20
TRUE	[BLASTED]	TRINITY_DN86156_c0_g2_i9	Actin cytoskeleton-regul	4486	20
TRUE	[BLASTED,	TRINITY_DN86118_c2_g2_i4	UDP-D-apiose/UDP-D-xyl	1600	20
TRUE	[BLASTED,	TRINITY_DN86197_c3_g5_i1	linoleate 13S-lipoxygena	944	20
TRUE	[BLASTED,	TRINITY_DN81180_c2_g4_i4	transcription factor bHL	1247	20
TRUE	[BLASTED]	TRINITY_DN81113_c1_g2_i4	SAP domain-containing p	1026	20
TRUE	[BLASTED,	TRINITY_DN81156_c1_g1_i3	E3 ubiquitin-protein liga	4756	20
TRUE	[BLASTED]	TRINITY_DN81485_c0_g2_i14	UHRF1-binding protein 1	4188	20
TRUE	[BLASTED,	TRINITY_DN81434_c0_g1_i12	probable methyltransfer	1195	20
TRUE	[BLASTED,	TRINITY_DN86328_c2_g1_i1	DnaJ subfamily C membe	2579	20
TRUE	[BLASTED,	TRINITY_DN86380_c0_g2_i2	BEL1-like homeodomain	3082	20
TRUE	[BLASTED,	TRINITY_DN79431_c0_g1_i17	protein Iojap, chloroplas	3334	20
TRUE	[BLASTED,	TRINITY_DN79434_c1_g2_i1	S-adenosylmethionine de	1130	20
TRUE	[BLASTED,	TRINITY_DN79425_c0_g1_i2	DNA ligase 1-like	1025	20
TRUE	[BLASTED,	TRINITY_DN79425_c0_g1_i3	uncharacterized protein	2489	20
TRUE	[BLASTED,	TRINITY_DN79430_c0_g1_i17	peptidyl-prolyl cis-trans i	2275	20
TRUE	[BLASTED,	TRINITY_DN84737_c0_g1_i3	stAR-related lipid transfe	2258	20

TRUE	[BLASTED, I	TRINITY_DN84754_c0_g1_i11	zeaxanthin epoxidase, ch	615	20
TRUE	[BLASTED, I	TRINITY_DN84730_c0_g2_i3	probable UDP-N-acetylgl	3131	20
TRUE	[BLASTED, I	TRINITY_DN77091_c0_g1_i2	ATP-dependent Clp prote	1735	20
TRUE	[BLASTED, I	TRINITY_DN77073_c0_g1_i3	probable receptor-like pr	1660	20
TRUE	[BLASTED, I	TRINITY_DN82634_c1_g2_i2	UDP-glucosyltransferase	1533	20
TRUE	[BLASTED]	TRINITY_DN82699_c1_g1_i7	rhodanese-like domain-c	4439	20
TRUE	[BLASTED]	TRINITY_DN82638_c1_g3_i4	uncharacterized protein I	1038	20
TRUE	[BLASTED, I	TRINITY_DN85768_c0_g1_i2	probable serine/threonir	1490	20
TRUE	[BLASTED, I	TRINITY_DN85778_c2_g2_i8	lysine-specific demethyla	2696	20
TRUE	[BLASTED, I	TRINITY_DN85755_c0_g3_i8	NADH--cytochrome b5 re	3959	20
TRUE	[BLASTED, I	TRINITY_DN80040_c1_g1_i9	ferritin-2, chloroplastic	1262	20
TRUE	[BLASTED, I	TRINITY_DN80028_c1_g4_i6	equilibrative nucleotide t	1341	20
TRUE	[BLASTED, I	TRINITY_DN80097_c1_g1_i4	Intracellular protein trans	1196	20
TRUE	[BLASTED, I	TRINITY_DN80036_c3_g3_i1	uncharacterized protein I	3579	20
TRUE	[BLASTED, I	TRINITY_DN80085_c4_g1_i8	glutamate 5-kinase isofor	1846	20
TRUE	[BLASTED, I	TRINITY_DN80085_c4_g1_i14	Isopentenyl phosphate ki	2300	20
TRUE	[BLASTED, I	TRINITY_DN81764_c0_g2_i2	F-box-like/WD repeat-co	1461	20
TRUE	[BLASTED, I	TRINITY_DN81790_c2_g1_i3	Serine/threonine protein	1503	20
TRUE	[BLASTED, I	TRINITY_DN81726_c4_g3_i4	heat shock 70 kDa protei	2517	20
TRUE	[BLASTED, I	TRINITY_DN81772_c0_g3_i2	xyloglucan endotransgluc	1162	20
TRUE	[BLASTED, I	TRINITY_DN86493_c2_g1_i9	Actin-7 like	438	20
TRUE	[BLASTED, I	TRINITY_DN86485_c0_g1_i32	5'-nucleotidase domain-c	1751	20
TRUE	[BLASTED, I	TRINITY_DN86488_c0_g1_i5	beta-glucosidase-like	2680	20
TRUE	[BLASTED, I	TRINITY_DN77618_c2_g1_i1	protein kinase PINOID-lik	1877	20
TRUE	[BLASTED, I	TRINITY_DN77691_c0_g1_i3	protein FAR1-RELATED SI	2700	20
TRUE	[BLASTED, I	TRINITY_DN83176_c2_g1_i10	AAA+-type ATPase	2697	20
TRUE	[BLASTED, I	TRINITY_DN83180_c0_g2_i21	telomere repeat-binding	2959	20
TRUE	[BLASTED, I	TRINITY_DN83157_c0_g11_i1	probable galactinol--sucr	1076	20
TRUE	[BLASTED, I	TRINITY_DN84189_c2_g7_i1	ORF64d (chloroplast)	291	20
TRUE	[BLASTED, I	TRINITY_DN84132_c2_g1_i2	early endosome antigen	2417	20
TRUE	[BLASTED, I	TRINITY_DN84132_c2_g1_i6	myosin heavy chain, non-	1296	20
TRUE	[BLASTED, I	TRINITY_DN84184_c0_g1_i5	dolichyl-diphosphooligos	2704	20
TRUE	[BLASTED, I	TRINITY_DN84141_c0_g1_i13	histidine--tRNA ligase, cy	1152	20
TRUE	[BLASTED, I	TRINITY_DN78144_c1_g3_i1	probable sugar phosphat	1577	20
TRUE	[BLASTED, I	TRINITY_DN78161_c0_g2_i7	protein TPLATE	3807	20
TRUE	[BLASTED, I	TRINITY_DN78176_c1_g1_i1	eukaryotic translation ini	1550	20
TRUE	[BLASTED, I	TRINITY_DN80388_c3_g3_i4	eukaryotic translation ini	1359	20
TRUE	[BLASTED, I	TRINITY_DN80397_c0_g2_i3	signal recognition particl	1829	20
TRUE	[BLASTED]	TRINITY_DN82353_c0_g1_i12	extensin-like	978	1
TRUE	[BLASTED, I	TRINITY_DN82310_c0_g2_i1	Complement factor I ligh	1330	20
TRUE	[BLASTED, I	TRINITY_DN82385_c5_g2_i8	elongation factor Ts, mit	1397	20
TRUE	[BLASTED]	TRINITY_DN76141_c0_g1_i4	pentatricopeptide repeat	3570	20
TRUE	[BLASTED]	TRINITY_DN88535_c4_g2_i6	stress response protein r	3821	13
TRUE	[BLASTED, I	TRINITY_DN84974_c1_g1_i6	mannosyl-oligosaccharid	4267	20
TRUE	[BLASTED, I	TRINITY_DN84914_c2_g2_i13	4-coumarate--CoA ligase-	2599	20
TRUE	[BLASTED, I	TRINITY_DN80450_c2_g1_i14	aldehyde dehydrogenase	2124	20
TRUE	[NO-BLAST	TRINITY_DN80488_c0_g3_i2	---NA---	602	
TRUE	[BLASTED, I	TRINITY_DN80414_c1_g1_i2	mitochondrial uncouplin	759	20
TRUE	[BLASTED, I	TRINITY_DN78771_c2_g2_i2	Caffeoylshikimate estera	1644	20
TRUE	[BLASTED, I	TRINITY_DN80871_c0_g2_i13	squamosa promoter-binc	2445	20

TRUE	[BLASTED, I	TRINITY_DN80836_c4_g1_i2	photosystem I P700 apop	2055	20
TRUE	[BLASTED]	TRINITY_DN80820_c0_g3_i1	F-box protein At4g18380	1083	20
TRUE	[BLASTED, I	TRINITY_DN83817_c3_g2_i11	transcription factor bHLH	1331	20
TRUE	[BLASTED, I	TRINITY_DN83817_c3_g2_i20	transcription factor bHLH	2209	20
TRUE	[BLASTED, I	TRINITY_DN83887_c0_g1_i16	riboflavin biosynthesis pr	1488	20
TRUE	[BLASTED, I	TRINITY_DN83874_c1_g1_i8	V-type proton ATPase sul	1704	20
TRUE	[BLASTED, I	TRINITY_DN83892_c1_g2_i19	long chain acyl-CoA synth	2426	20
TRUE	[BLASTED, I	TRINITY_DN83869_c1_g2_i1	lysine-specific histone de	2453	20
TRUE	[BLASTED, I	TRINITY_DN83803_c3_g2_i1	protein TRAUCO	1833	20
TRUE	[BLASTED, I	TRINITY_DN84879_c0_g1_i7	E3 ubiquitin-protein ligas	5985	20
TRUE	[BLASTED, I	TRINITY_DN84811_c0_g3_i8	ABC transporter C family	5593	20
TRUE	[BLASTED, I	TRINITY_DN84811_c0_g3_i14	ABC transporter C family	5409	20
TRUE	[BLASTED]	TRINITY_DN84891_c2_g1_i12	probable LRR receptor-lik	1740	2
TRUE	[BLASTED, I	TRINITY_DN84865_c0_g1_i2	chaperone protein dnaJ C	1545	20
TRUE	[BLASTED, I	TRINITY_DN79101_c1_g3_i7	thaumatin-like protein 1	3989	20
TRUE	[BLASTED, I	TRINITY_DN86621_c3_g3_i2	glycosyl transferase (glyc	1197	20
TRUE	[BLASTED, I	TRINITY_DN86606_c1_g1_i9	autophagy-related protei	2219	20
TRUE	[BLASTED, I	TRINITY_DN86683_c0_g8_i1	dicarboxylate transporte	2067	20
TRUE	[BLASTED, I	TRINITY_DN86631_c1_g2_i5	alkaline/neutral invertase	3199	20
TRUE	[BLASTED, I	TRINITY_DN86626_c1_g6_i2	ATPase subunit 1 (mitoch	2961	20
TRUE	[BLASTED, I	TRINITY_DN74580_c0_g1_i1	cytochrome P450 78A5-li	2027	20
TRUE	[BLASTED]	TRINITY_DN77456_c2_g4_i7	stem-specific protein TSJT	1043	20
TRUE	[BLASTED, I	TRINITY_DN77499_c3_g2_i15	probable LRR receptor-lik	863	20
TRUE	[NO-BLAST	TRINITY_DN88732_c1_g1_i10	---NA---	3240	
TRUE	[BLASTED, I	TRINITY_DN88734_c4_g1_i2	Photosystem II protein D	2104	20
TRUE	[BLASTED, I	TRINITY_DN88734_c4_g1_i6	Photosystem II protein D	1514	20
TRUE	[BLASTED, I	TRINITY_DN88726_c0_g1_i1	apocytochrome b (mitoch	1720	20
TRUE	[BLASTED, I	TRINITY_DN88726_c1_g1_i8	DEAD-box ATP-depender	2575	20
TRUE	[BLASTED]	TRINITY_DN88765_c4_g5_i2	uncharacterized protein I	378	20
TRUE	[BLASTED, I	TRINITY_DN88736_c1_g2_i1	coat protein	260	4
TRUE	[BLASTED, I	TRINITY_DN88761_c5_g2_i11	E3 ubiquitin-protein ligas	5139	20
TRUE	[BLASTED, I	TRINITY_DN88782_c1_g4_i1	pentatricopeptide repeat	4148	20
TRUE	[BLASTED, I	TRINITY_DN79532_c1_g2_i2	Altered inheritance of mi	685	20
TRUE	[BLASTED, I	TRINITY_DN79513_c1_g1_i2	B-box zinc finger protein	1468	20
TRUE	[BLASTED, I	TRINITY_DN88846_c3_g2_i3	rRNA intron-encoded hor	352	20
TRUE	[BLASTED, I	TRINITY_DN88846_c3_g2_i6	rRNA intron-encoded hor	506	20
TRUE	[BLASTED, I	TRINITY_DN88846_c3_g2_i12	rRNA intron-encoded hor	412	20
TRUE	[BLASTED, I	TRINITY_DN88846_c3_g2_i13	rRNA intron-encoded hor	836	20
TRUE	[BLASTED, I	TRINITY_DN88846_c4_g2_i2	hypothetical protein OXY	525	20
TRUE	[BLASTED, I	TRINITY_DN78356_c0_g1_i5	glutamine synthetase cyt	1785	20
TRUE	[BLASTED, I	TRINITY_DN78311_c0_g1_i5	probable magnesium tra	1920	20
TRUE	[BLASTED, I	TRINITY_DN85514_c1_g1_i9	mechanosensitive ion cha	6918	20
TRUE	[BLASTED, I	TRINITY_DN85567_c1_g1_i3	ras-related protein RABE	605	20
TRUE	[BLASTED, I	TRINITY_DN85516_c1_g3_i1	succinate-semialdehyde c	1075	20
TRUE	[BLASTED]	TRINITY_DN85140_c0_g1_i11	ubiquitin domain-contair	1539	20
TRUE	[BLASTED, I	TRINITY_DN85165_c2_g1_i5	unknow protein	699	20
TRUE	[BLASTED, I	TRINITY_DN85110_c1_g2_i6	DUF3456 domain proteir	1325	20
TRUE	[BLASTED, I	TRINITY_DN85116_c0_g2_i6	UPF0496 protein	2321	20
TRUE	[BLASTED, I	TRINITY_DN83688_c1_g4_i3	vacuolar cation/proton e	1202	20
TRUE	[BLASTED, I	TRINITY_DN83651_c2_g2_i11	glycogen phosphorylase 1	3280	20

TRUE	[BLASTED, I	TRINITY_DN81605_c0_g1_i5	F-box protein At3g54460	3889	20
TRUE	[BLASTED, I	TRINITY_DN81631_c1_g1_i3	heat shock 70 kDa protei	3307	20
TRUE	[BLASTED]	TRINITY_DN81654_c1_g2_i2	protein CROWDED NUCL	3523	20
TRUE	[BLASTED, I	TRINITY_DN81692_c2_g2_i1	bZIP transcription factor	1506	20
TRUE	[BLASTED, I	TRINITY_DN81653_c0_g2_i13	probable ubiquitin-conju	2918	20
TRUE	[BLASTED, I	TRINITY_DN81673_c0_g2_i4	growth-regulating factor	1725	20
TRUE	[BLASTED, I	TRINITY_DN78289_c1_g1_i2	photosystem II repair prc	961	20
TRUE	[BLASTED, I	TRINITY_DN83746_c1_g1_i4	btbpoz domain-containir	1745	20
TRUE	[BLASTED, I	TRINITY_DN83759_c1_g4_i5	thiamine-phosphate pyrc	1816	20
TRUE	[BLASTED, I	TRINITY_DN83788_c0_g1_i1	delta(24)-sterol reductas	783	20
TRUE	[BLASTED, I	TRINITY_DN79899_c2_g1_i5	probable WRKY transcrip	2166	20
TRUE	[BLASTED, I	TRINITY_DN79826_c3_g6_i3	Uncharacterised protein	471	20
TRUE	[BLASTED, I	TRINITY_DN79853_c1_g1_i2	uncharacterized protein I	2568	20
TRUE	[BLASTED, I	TRINITY_DN79896_c1_g5_i1	orf108 (mitochondrion)	294	20
TRUE	[BLASTED]	TRINITY_DN65310_c0_g1_i2	uncharacterized protein I	674	20
TRUE	[BLASTED]	TRINITY_DN80281_c1_g2_i1	hypothetical protein CFP.	643	12
TRUE	[BLASTED, I	TRINITY_DN78909_c2_g1_i2	chaperonin CPN60-2, mit	1222	20
TRUE	[BLASTED, I	TRINITY_DN78945_c0_g1_i1	molybdopterin biosynthe	2328	20
TRUE	[BLASTED, I	TRINITY_DN78993_c2_g4_i4	GDSL esterase/lipase At2	995	20
TRUE	[BLASTED]	TRINITY_DN75673_c0_g1_i1	ethylene-overproduction	1775	20
TRUE	[BLASTED, I	TRINITY_DN86788_c0_g1_i1	Plant calmodulin-binding	1287	20
TRUE	[BLASTED]	TRINITY_DN86717_c2_g3_i4	uncharacterized protein /	2324	20
TRUE	[BLASTED]	TRINITY_DN86797_c1_g1_i6	serine/threonine-protein	1130	20
TRUE	[BLASTED, I	TRINITY_DN86743_c1_g1_i6	paired amphipathic helix	4329	20
TRUE	[BLASTED, I	TRINITY_DN76711_c0_g1_i1	transcription initiation fa	1453	20
TRUE	[BLASTED, I	TRINITY_DN85879_c0_g2_i13	E3 ubiquitin-protein ligas	2053	20
TRUE	[BLASTED, I	TRINITY_DN85868_c3_g8_i3	Uncharacterised protein	503	20
TRUE	[BLASTED, I	TRINITY_DN85810_c3_g4_i10	putative late blight resist	3039	20
TRUE	[BLASTED, I	TRINITY_DN85899_c1_g1_i5	probable serine/threonir	3164	20
TRUE	[BLASTED, I	TRINITY_DN85880_c2_g2_i4	mannan endo-1,4-beta-m	1045	20
TRUE	[BLASTED, I	TRINITY_DN81974_c3_g1_i12	protein LHY	2478	20
TRUE	[BLASTED]	TRINITY_DN81982_c1_g1_i2	uncharacterized protein I	901	20
TRUE	[BLASTED, I	TRINITY_DN81930_c1_g1_i5	ADP-ribosyl cyclase/cycli	1593	20
TRUE	[BLASTED, I	TRINITY_DN81967_c1_g2_i2	U-box domain-containing	1447	20
TRUE	[BLASTED, I	TRINITY_DN81934_c0_g1_i5	psbP domain-containing	2253	20
TRUE	[BLASTED, I	TRINITY_DN81990_c1_g1_i7	mitochondrial import inn	1271	20
TRUE	[BLASTED, I	TRINITY_DN83244_c3_g5_i1	elongation factor Tu, chlc	695	20
TRUE	[BLASTED, I	TRINITY_DN83246_c2_g1_i1	factor of DNA methylatio	1091	20
TRUE	[BLASTED, I	TRINITY_DN77563_c1_g5_i6	chaperone protein ClpB1	1049	20
TRUE	[NO-BLAST	TRINITY_DN85390_c0_g1_i4	---NA---	908	
TRUE	[BLASTED, I	TRINITY_DN85380_c2_g1_i4	aquaporin PIP1-1	1033	20
TRUE	[BLASTED, I	TRINITY_DN85326_c0_g2_i5	myb-related protein B isc	1491	20
TRUE	[BLASTED, I	TRINITY_DN85300_c1_g1_i2	5'-3' exoribonuclease 3	1904	20
TRUE	[BLASTED, I	TRINITY_DN85379_c0_g1_i1	ketol-acid reductoisomer	2033	20
TRUE	[NO-BLAST	TRINITY_DN80500_c1_g1_i3	---NA---	1946	
TRUE	[BLASTED, I	TRINITY_DN80543_c1_g1_i3	mannose-1-phosphate gu	1757	20
TRUE	[BLASTED]	TRINITY_DN80545_c0_g2_i4	J protein JJJ2-like	2553	20
TRUE	[BLASTED, I	TRINITY_DN80586_c1_g3_i1	ATP sulfurylase 2	908	20
TRUE	[BLASTED, I	TRINITY_DN78516_c0_g5_i1	Spindle pole body protei	1208	20
TRUE	[BLASTED, I	TRINITY_DN87157_c0_g2_i4	uncharacterized membra	1305	20

TRUE	[BLASTED, I	TRINITY_DN87173_c1_g4_i3	protein DENND6A isoforr	3508	20
TRUE	[BLASTED, I	TRINITY_DN87124_c0_g2_i3	protein TIME FOR COFFE	2235	20
TRUE	[BLASTED, I	TRINITY_DN87124_c0_g2_i13	protein TIME FOR COFFE	2137	20
TRUE	[BLASTED, I	TRINITY_DN87124_c0_g6_i4	protein TIME FOR COFFE	2511	20
TRUE	[BLASTED, I	TRINITY_DN87131_c2_g1_i1	Cell wall-associated hydr	316	20
TRUE	[BLASTED, I	TRINITY_DN87131_c2_g3_i1	signal anchor, putative	361	20
TRUE	[BLASTED, I	TRINITY_DN87181_c0_g3_i12	BTB/POZ domain-contair	2851	20
TRUE	[BLASTED]	TRINITY_DN87105_c1_g1_i7	Dentin sialoprotein	2183	20
TRUE	[BLASTED]	TRINITY_DN87105_c1_g1_i10	uncharacterized protein I	1541	20
TRUE	[BLASTED, I	TRINITY_DN87165_c1_g4_i2	phosphatidylinositol 4-kir	1564	20
TRUE	[BLASTED, I	TRINITY_DN87165_c1_g1_i3	phosphatidylinositol 4-kir	1585	20
TRUE	[BLASTED, I	TRINITY_DN87154_c2_g2_i19	putative pentatricopeptic	6297	20
TRUE	[BLASTED, I	TRINITY_DN87154_c2_g2_i23	putative pentatricopeptic	5422	20
TRUE	[BLASTED, I	TRINITY_DN87128_c2_g1_i1	U1 small nuclear ribonuc	1508	20
TRUE	[BLASTED, I	TRINITY_DN87529_c3_g4_i6	ribosomal protein subuni	2566	20
TRUE	[BLASTED, I	TRINITY_DN87516_c0_g2_i2	phosphoinositide phosph	1098	20
TRUE	[BLASTED, I	TRINITY_DN83360_c2_g1_i19	ABC transporter B family	2375	20
TRUE	[BLASTED, I	TRINITY_DN83301_c0_g2_i3	transcription factor SRM1	878	20
TRUE	[BLASTED, I	TRINITY_DN83389_c1_g1_i8	CLP protease regulatory s	1814	20
TRUE	[BLASTED]	TRINITY_DN83389_c1_g2_i6	Glyoxalase-like domain p	1266	20
TRUE	[BLASTED, I	TRINITY_DN83302_c3_g1_i8	Ferritin-2, chloroplastic	1174	20
TRUE	[BLASTED, I	TRINITY_DN87363_c0_g6_i4	ER membrane protein co	1176	20
TRUE	[BLASTED, I	TRINITY_DN74124_c0_g1_i2	uncharacterized protein I	945	20
TRUE	[BLASTED, I	TRINITY_DN78493_c1_g2_i12	protein IQ-DOMAIN 32	2259	20
TRUE	[BLASTED]	TRINITY_DN78493_c1_g2_i13	protein IQ-DOMAIN 32	2896	20
TRUE	[BLASTED, I	TRINITY_DN78474_c3_g1_i1	hypothetical protein GLY	316	20
TRUE	[BLASTED, I	TRINITY_DN78435_c0_g5_i3	40S ribosomal protein S9	484	20
TRUE	[BLASTED, I	TRINITY_DN78486_c0_g1_i14	superoxide dismutase [Fe	1128	20
TRUE	[BLASTED]	TRINITY_DN78430_c3_g5_i1	Zinc finger protein 706	1225	20
TRUE	[BLASTED, I	TRINITY_DN83917_c3_g1_i1	dehydration-responsive e	1629	20
TRUE	[BLASTED, I	TRINITY_DN82764_c2_g1_i2	desiccation protectant pr	3531	20
TRUE	[BLASTED, I	TRINITY_DN82770_c0_g6_i1	cyclic dof factor 2-like	1819	20
TRUE	[BLASTED, I	TRINITY_DN82733_c0_g1_i6	bidirectional sugar transp	997	20
TRUE	[BLASTED, I	TRINITY_DN82793_c0_g1_i5	U-box domain-containing	3131	20
TRUE	[BLASTED, I	TRINITY_DN87005_c1_g1_i1	aspartic proteinase-like p	2731	20
TRUE	[BLASTED, I	TRINITY_DN87016_c1_g2_i33	DEAD-box ATP-dependen	1674	20
TRUE	[BLASTED, I	TRINITY_DN87044_c0_g1_i6	putative disease resistanc	4892	20
TRUE	[BLASTED, I	TRINITY_DN87049_c0_g1_i25	DNA-directed RNA polym	3311	20
TRUE	[BLASTED, I	TRINITY_DN87075_c1_g2_i3	V-type proton ATPase su	3397	20
TRUE	[BLASTED, I	TRINITY_DN87078_c0_g2_i3	homeobox-leucine zipper	1234	20
TRUE	[BLASTED, I	TRINITY_DN87668_c0_g1_i4	alpha-mannosidase-like i	1063	20
TRUE	[BLASTED]	TRINITY_DN87633_c0_g1_i3	protein OBERON 4	4513	20
TRUE	[BLASTED, I	TRINITY_DN87622_c2_g2_i7	inositol hexakisphosphat	1499	20
TRUE	[BLASTED, I	TRINITY_DN87694_c0_g1_i17	ABC transporter G family	1193	20
TRUE	[BLASTED, I	TRINITY_DN80652_c2_g1_i3	vacuolar protein-sorting-	1407	20
TRUE	[BLASTED, I	TRINITY_DN77709_c1_g1_i5	vacuolar protein 8	856	20
TRUE	[BLASTED, I	TRINITY_DN77798_c0_g1_i2	NAP1-related protein 2-li	968	20
TRUE	[BLASTED, I	TRINITY_DN77717_c3_g1_i5	protein YIPF6 homolog	981	20
TRUE	[BLASTED, I	TRINITY_DN82951_c0_g1_i2	oleosin 1-like	843	20
TRUE	[BLASTED, I	TRINITY_DN82948_c1_g1_i1	glutamyl-tRNA reductase	2196	20

TRUE	[BLASTED, I	TRINITY_DN82982_c0_g1_i5	shaggy-related protein ki	1590	20
TRUE	[BLASTED, I	TRINITY_DN82945_c0_g1_i5	acylamino-acid-releasing	2908	20
TRUE	[BLASTED, I	TRINITY_DN82900_c2_g1_i6	40S ribosomal protein S4	1176	20
TRUE	[BLASTED]	TRINITY_DN82989_c1_g2_i16	uncharacterized protein I	2166	20
TRUE	[BLASTED, I	TRINITY_DN87790_c2_g4_i1	photosystem II protein D	1178	20
TRUE	[BLASTED, I	TRINITY_DN87726_c0_g2_i12	probable E3 ubiquitin liga	1124	20
TRUE	[BLASTED, I	TRINITY_DN78859_c0_g1_i7	ruvB-like protein 1	2343	20
TRUE	[BLASTED, I	TRINITY_DN78830_c5_g3_i4	GDSL esterase/lipase APC	1239	20
TRUE	[BLASTED]	TRINITY_DN78803_c2_g1_i5	uncharacterized protein I	2232	1
TRUE	[BLASTED, I	TRINITY_DN86017_c1_g2_i5	maturase K (chloroplast)	4211	20
TRUE	[BLASTED, I	TRINITY_DN86045_c3_g1_i1	PsbB (chloroplast)	1086	20
TRUE	[BLASTED, I	TRINITY_DN86045_c3_g3_i1	putative serine acetyltrar	597	16
TRUE	[BLASTED, I	TRINITY_DN86081_c5_g1_i4	ATPase subunit 9 (mitoch	1101	20
TRUE	[BLASTED, I	TRINITY_DN77865_c2_g7_i1	UDP-glycosyltransferase	1126	20
TRUE	[BLASTED, I	TRINITY_DN77893_c0_g1_i8	protein POLLEN DEFECTIV	1211	20
TRUE	[BLASTED, I	TRINITY_DN84032_c1_g1_i3	uridine-cytidine kinase C-	985	20
TRUE	[BLASTED, I	TRINITY_DN84045_c0_g4_i1	hypothetical protein GLY	503	20
TRUE	[BLASTED, I	TRINITY_DN84040_c1_g2_i7	DExH-box ATP-dependen	2058	20
TRUE	[BLASTED, I	TRINITY_DN84046_c3_g2_i4	uncharacterized membra	1070	20
TRUE	[BLASTED, I	TRINITY_DN84055_c1_g4_i3	Transcription factor DIVA	1131	20
TRUE	[BLASTED, I	TRINITY_DN79059_c2_g3_i7	methyl-CpG-binding dom	1647	20
TRUE	[NO-BLAST	TRINITY_DN79090_c0_g3_i1	---NA---	770	
TRUE	[BLASTED, I	TRINITY_DN79063_c1_g1_i9	long-chain-alcohol oxidas	2853	20
TRUE	[BLASTED, I	TRINITY_DN87956_c3_g3_i2	NADH dehydrogenase su	3200	20
TRUE	[BLASTED, I	TRINITY_DN87924_c1_g2_i1	Protein GIGANTEA	1092	20
TRUE	[BLASTED, I	TRINITY_DN87930_c2_g2_i8	ribulose-1,5-bisphosphat	1521	20
TRUE	[BLASTED, I	TRINITY_DN87926_c0_g1_i9	exocyst complex compor	4592	20
TRUE	[BLASTED, I	TRINITY_DN82174_c3_g2_i7	40S ribosomal protein S1	649	20
TRUE	[BLASTED, I	TRINITY_DN82102_c2_g1_i3	at-rich interactive domai	3202	20
TRUE	[BLASTED, I	TRINITY_DN83533_c1_g1_i4	transcription factor bHLH	3442	20
TRUE	[BLASTED, I	TRINITY_DN83571_c1_g1_i2	histone-lysine N-methylt	2090	20
TRUE	[BLASTED, I	TRINITY_DN86957_c0_g2_i1	protein FAR1-RELATED SE	1610	20
TRUE	[BLASTED]	TRINITY_DN86952_c1_g1_i13	ARM REPEAT PROTEIN IN	935	20
TRUE	[BLASTED, I	TRINITY_DN86918_c1_g2_i10	probable acyl-CoA dehyd	3191	20
TRUE	[BLASTED, I	TRINITY_DN86918_c1_g2_i15	probable acyl-CoA dehyd	3783	20
TRUE	[BLASTED, I	TRINITY_DN86905_c0_g4_i4	transcription factor LHW-	3469	20
TRUE	[BLASTED, I	TRINITY_DN76824_c3_g3_i5	Anaphase-promoting cor	821	20
TRUE	[BLASTED, I	TRINITY_DN80700_c1_g1_i3	orf187 (mitochondrion)	406	20
TRUE	[BLASTED, I	TRINITY_DN80700_c1_g1_i4	orf187 (mitochondrion)	1021	20
TRUE	[BLASTED, I	TRINITY_DN80732_c0_g1_i15	predicted protein	954	1
TRUE	[BLASTED, I	TRINITY_DN80746_c0_g1_i14	myosin heavy chain-like p	794	20
TRUE	[BLASTED, I	TRINITY_DN86227_c0_g2_i16	ribonuclease II, chloropla	1038	20
TRUE	[BLASTED]	TRINITY_DN86234_c0_g1_i3	protein EFR3 homolog isc	920	20
TRUE	[BLASTED, I	TRINITY_DN86226_c1_g1_i10	ferritin-3, chloroplastic-li	1507	20
TRUE	[BLASTED, I	TRINITY_DN86226_c1_g1_i11	ferritin-3, chloroplastic	1365	20
TRUE	[BLASTED, I	TRINITY_DN86226_c1_g1_i17	ferritin-3, chloroplastic	1467	20
TRUE	[BLASTED, I	TRINITY_DN86226_c1_g1_i18	ferritin-3, chloroplastic	649	20
TRUE	[BLASTED, I	TRINITY_DN86226_c1_g1_i19	ferritin-3, chloroplastic-li	870	20
TRUE	[BLASTED, I	TRINITY_DN86273_c4_g1_i17	monodehydroascorbate r	2785	20
TRUE	[BLASTED, I	TRINITY_DN85978_c0_g1_i5	transcriptional repressor	998	20

TRUE	[BLASTED, I	TRINITY_DN85962_c1_g1_i6	Insulin-degrading enzym	2777	20
TRUE	[BLASTED, I	TRINITY_DN85968_c2_g2_i9	Serine/threonine protein	2357	20
TRUE	[BLASTED, I	TRINITY_DN85941_c0_g2_i1	Retrovirus-related Pol po	4592	20
TRUE	[BLASTED]	TRINITY_DN85900_c0_g4_i7	QWRF motif-containing p	1307	20
TRUE	[BLASTED, I	TRINITY_DN82239_c0_g1_i20	mitogen-activated protei	1271	20
TRUE	[BLASTED, I	TRINITY_DN82285_c1_g1_i4	mitogen-activated protei	1247	20
TRUE	[BLASTED, I	TRINITY_DN82235_c5_g2_i2	CBL-interacting serine/th	1982	20
TRUE	[BLASTED, I	TRINITY_DN82236_c2_g2_i2	hydroperoxide lyase	2092	2
TRUE	[BLASTED, I	TRINITY_DN82242_c0_g3_i3	pectinesterase-like	1670	20
TRUE	[BLASTED, I	TRINITY_DN76436_c0_g1_i5	methyl-CpG-binding dom	2044	20
TRUE	[BLASTED, I	TRINITY_DN76432_c0_g1_i7	protein FAR1-RELATED SI	4627	20
TRUE	[BLASTED, I	TRINITY_DN83413_c1_g1_i6	K(+) efflux antiporter 5	1409	20
TRUE	[BLASTED]	TRINITY_DN83408_c3_g1_i3	tubby-like F-box protein	733	20
TRUE	[BLASTED, I	TRINITY_DN83486_c0_g1_i14	3-phosphoshikimate 1-ca	2774	20
TRUE	[BLASTED, I	TRINITY_DN79902_c0_g2_i5	universal stress protein P	1165	20
TRUE	[BLASTED, I	TRINITY_DN77150_c0_g1_i4	uncharacterized protein	2602	20
TRUE	[BLASTED, I	TRINITY_DN84578_c2_g1_i3	monosaccharide-sensing	1705	20
TRUE	[BLASTED, I	TRINITY_DN84543_c3_g1_i18	LRR receptor-like serine/	772	20
TRUE	[BLASTED, I	TRINITY_DN84587_c1_g1_i2	protein phosphatase 2C	4651	20
TRUE	[BLASTED, I	TRINITY_DN84553_c2_g1_i7	guanylate-binding protei	2307	20
TRUE	[BLASTED, I	TRINITY_DN84511_c3_g1_i4	plasma membrane-assoc	1228	20
TRUE	[BLASTED]	TRINITY_DN84561_c2_g2_i4	uncharacterized protein	3190	20
TRUE	[BLASTED, I	TRINITY_DN81372_c1_g2_i1	glycine-rich RNA-binding	1114	20
TRUE	[BLASTED, I	TRINITY_DN81334_c3_g1_i7	kelch repeat-containing p	2580	20
TRUE	[BLASTED, I	TRINITY_DN82814_c3_g3_i1	ADP,ATP carrier protein	1161	20
TRUE	[BLASTED, I	TRINITY_DN82802_c0_g2_i10	cyclin-dependent kinase	1385	20
TRUE	[BLASTED, I	TRINITY_DN82802_c0_g2_i11	cyclin-dependent kinase	2197	20
TRUE	[BLASTED, I	TRINITY_DN82813_c0_g2_i2	mitogen-activated protei	1723	20
TRUE	[BLASTED, I	TRINITY_DN82882_c2_g2_i16	probable alpha-amylase	774	20
TRUE	[BLASTED, I	TRINITY_DN82882_c2_g2_i18	probable alpha-amylase	1975	20
TRUE	[BLASTED, I	TRINITY_DN82827_c0_g2_i4	probable galactinol--sucr	1863	20
TRUE	[BLASTED, I	TRINITY_DN82831_c0_g1_i8	probable trehalose-phos	2155	20
TRUE	[BLASTED, I	TRINITY_DN82851_c1_g1_i4	protein BPS1, chloroplast	2348	20
TRUE	[BLASTED]	TRINITY_DN82811_c0_g2_i5	uncharacterized protein	6269	20
TRUE	[BLASTED, I	TRINITY_DN82857_c1_g2_i15	elongator complex prote	2635	20
TRUE	[BLASTED, I	TRINITY_DN84349_c0_g1_i7	calcineurin B-like protein	2000	20
TRUE	[BLASTED]	TRINITY_DN84336_c1_g3_i2	RRNA intron-encoded ho	352	20
TRUE	[BLASTED]	TRINITY_DN84328_c0_g6_i1	auxilin-like protein 1	4124	20
TRUE	[BLASTED]	TRINITY_DN84332_c1_g3_i11	protein TSS	6491	20
TRUE	[BLASTED]	TRINITY_DN84355_c1_g3_i2	Vacuolar protein sorting-	4946	20
TRUE	[BLASTED, I	TRINITY_DN84318_c0_g1_i3	ethylene-responsive tran	1358	20
TRUE	[BLASTED, I	TRINITY_DN84469_c2_g9_i4	photosystem I P700 apo	1059	20
TRUE	[BLASTED, I	TRINITY_DN84441_c0_g1_i6	Pentatricopeptide repeat	1320	20
TRUE	[BLASTED, I	TRINITY_DN84424_c2_g1_i1	LRR receptor-like serine/	3408	20
TRUE	[BLASTED, I	TRINITY_DN84461_c2_g1_i3	60S ribosomal protein L3	505	20
TRUE	[BLASTED, I	TRINITY_DN84440_c1_g1_i6	peptidyl-prolyl cis-trans i	2155	20
TRUE	[BLASTED, I	TRINITY_DN82021_c1_g4_i1	Uncharacterised protein	494	20
TRUE	[BLASTED, I	TRINITY_DN82079_c1_g1_i15	probable aspartyl amino	1773	20
TRUE	[BLASTED, I	TRINITY_DN82022_c1_g1_i21	uncharacterized protein	2336	20
TRUE	[BLASTED, I	TRINITY_DN82055_c0_g4_i12	putative glutathione-spe	1466	20

TRUE	[BLASTED,	TRINITY_DN82036_c1_g1_i1	GTP-binding nuclear prot	1883	20
TRUE	[BLASTED,	TRINITY_DN86524_c1_g1_i16	acyl-coenzyme A thioeste	880	20
TRUE	[BLASTED,	TRINITY_DN79278_c0_g2_i6	DNA repair protein RAD1	1585	20
TRUE	[BLASTED,	TRINITY_DN79276_c0_g1_i3	WPP domain-interacting	2937	20
TRUE	[BLASTED,	TRINITY_DN79223_c1_g2_i9	ABC transporter A family	6354	20
TRUE	[BLASTED]	TRINITY_DN79235_c0_g1_i1	putative defensin-like pro	432	9
TRUE	[BLASTED,	TRINITY_DN79209_c1_g1_i8	ubiquitin-conjugating enz	1153	20
TRUE	[BLASTED,	TRINITY_DN79244_c2_g1_i1	ferritin-3, chloroplastic-li	424	20
TRUE	[BLASTED,	TRINITY_DN79244_c2_g4_i1	ferritin-3, chloroplastic	1312	20
TRUE	[BLASTED,	TRINITY_DN79244_c2_g4_i2	ferritin-3, chloroplastic-li	1057	20
TRUE	[BLASTED,	TRINITY_DN79244_c2_g4_i7	ferritin-3, chloroplastic-li	787	20
TRUE	[BLASTED,	TRINITY_DN79244_c2_g2_i3	ferritin-3, chloroplastic-li	452	20
TRUE	[BLASTED,	TRINITY_DN81043_c1_g1_i3	carbon catabolite repress	1166	20
TRUE	[BLASTED]	TRINITY_DN81087_c0_g1_i7	Aconitate hydratase, mit	909	2
TRUE	[BLASTED,	TRINITY_DN80957_c1_g1_i2	expansin-A1 isoform X1	1072	20
TRUE	[BLASTED,	TRINITY_DN80951_c0_g1_i8	haloacid dehalogenase-li	4084	20
TRUE	[BLASTED,	TRINITY_DN80906_c1_g1_i11	R-linalool synthase	725	20
TRUE	[BLASTED,	TRINITY_DN80969_c1_g1_i3	Transmembrane protein	3491	20
TRUE	[BLASTED]	TRINITY_DN80986_c1_g4_i4	DNA polymerase	1799	20
TRUE	[BLASTED,	TRINITY_DN81285_c2_g2_i11	DExH-box ATP-dependen	4967	20
TRUE	[BLASTED,	TRINITY_DN81285_c2_g2_i20	DExH-box ATP-dependen	2860	20
TRUE	[BLASTED,	TRINITY_DN81204_c0_g1_i4	choline transporter-like p	2614	20
TRUE	[BLASTED,	TRINITY_DN81228_c1_g4_i6	cullin-associated NEDD8-	2618	20
TRUE	[BLASTED,	TRINITY_DN87245_c1_g2_i3	DNA mismatch repair pro	1590	20
TRUE	[BLASTED,	TRINITY_DN87294_c0_g1_i1	pentatricopeptide repeat	2562	20
TRUE	[BLASTED,	TRINITY_DN87236_c2_g4_i1	probable protein phosph	1035	8
TRUE	[BLASTED,	TRINITY_DN87235_c0_g1_i17	(3S,6E)-nerolidol synthas	1499	20
TRUE	[BLASTED,	TRINITY_DN83053_c0_g1_i4	Zinc finger protein like	2158	20
TRUE	[BLASTED,	TRINITY_DN83059_c0_g1_i6	eukaryotic initiation facto	622	20
TRUE	[BLASTED,	TRINITY_DN82424_c0_g1_i6	Heterogeneous nuclear r	1083	20
TRUE	[BLASTED]	TRINITY_DN82465_c0_g2_i4	uncharacterized protein	2385	20
TRUE	[BLASTED,	TRINITY_DN82461_c5_g1_i4	photosystem II phosphop	903	20
TRUE	[BLASTED]	TRINITY_DN82418_c0_g1_i5	protein BLISTER-like isofo	1511	20
TRUE	[BLASTED,	TRINITY_DN85435_c0_g1_i4	NAD-dependent malic en	2956	20
TRUE	[BLASTED,	TRINITY_DN85467_c0_g1_i3	DNA helicase INO80 isofo	4292	20
TRUE	[BLASTED,	TRINITY_DN85445_c1_g2_i3	U-box domain-containing	4093	20
TRUE	[BLASTED,	TRINITY_DN85402_c0_g2_i17	histone acetyltransferase	1185	20
TRUE	[BLASTED,	TRINITY_DN84287_c1_g1_i6	scarecrow-like protein 14	3240	20
TRUE	[BLASTED,	TRINITY_DN84279_c0_g1_i7	probable metal-nicotiana	1994	20
TRUE	[BLASTED,	TRINITY_DN84265_c1_g2_i6	probable inactive recepto	2715	20
TRUE	[BLASTED,	TRINITY_DN79388_c1_g1_i12	trafficking protein particl	985	20
TRUE	[BLASTED,	TRINITY_DN79374_c2_g1_i2	ATP-dependent Clp prote	989	20
TRUE	[BLASTED,	TRINITY_DN79320_c0_g11_i1	galactan beta-1,4-galacto	2269	20
TRUE	[BLASTED,	TRINITY_DN82599_c2_g2_i1	protein RETICULATA-RELA	2171	20
TRUE	[BLASTED,	TRINITY_DN82516_c0_g2_i5	ubiquitin-conjugating enz	2341	20
TRUE	[BLASTED,	TRINITY_DN82522_c1_g3_i5	reticulon-like protein B8	2933	20
TRUE	[BLASTED,	TRINITY_DN82519_c0_g5_i3	beta-glucuronosyltransfe	543	20
TRUE	[BLASTED]	TRINITY_DN82575_c1_g1_i13	PITH domain-containing	997	20
TRUE	[BLASTED,	TRINITY_DN82559_c0_g2_i7	elongator complex prote	1480	20
TRUE	[BLASTED,	TRINITY_DN79625_c0_g1_i7	Eukaryotic translation ini	2332	20

TRUE	[BLASTED, I	TRINITY_DN79647_c0_g1_i11	probable ADP-ribosylatio	2596	20
TRUE	[BLASTED, I	TRINITY_DN81556_c1_g1_i4	protein CIA1	716	20
TRUE	[BLASTED, I	TRINITY_DN81593_c2_g2_i7	peroxidase 42-like	1339	20
TRUE	[BLASTED, I	TRINITY_DN81596_c0_g3_i1	monothiol glutaredoxin-S	636	20
TRUE	[NO-BLAST	TRINITY_DN81551_c1_g1_i11	---NA---	743	
TRUE	[BLASTED, I	TRINITY_DN78090_c4_g1_i14	vacuolar protein-sorting-	3562	20
TRUE	[BLASTED, I	TRINITY_DN78013_c1_g1_i3	rhomboid-like protein 19	4715	20
TRUE	[BLASTED, I	TRINITY_DN78013_c1_g1_i11	rhomboid-like protein 19	4823	20
TRUE	[BLASTED, I	TRINITY_DN78091_c0_g1_i7	transcription factor UNE1	1792	20
TRUE	[BLASTED, I	TRINITY_DN88004_c1_g11_i3	Cell wall-associated hydr	990	20
TRUE	[BLASTED, I	TRINITY_DN88011_c2_g1_i22	aldo-keto reductase fami	1086	20
TRUE	[BLASTED, I	TRINITY_DN88032_c0_g1_i2	DNA polymerase zeta cat	6329	20
TRUE	[BLASTED, I	TRINITY_DN88032_c0_g1_i15	DNA polymerase zeta cat	6333	20
TRUE	[BLASTED, I	TRINITY_DN88087_c3_g1_i2	lysine-specific demethyla	4619	20
TRUE	[BLASTED, I	TRINITY_DN88066_c1_g1_i6	cationic amino acid trans	3688	20
TRUE	[BLASTED]	TRINITY_DN88060_c2_g2_i9	serine carboxypeptidase-	1098	20
TRUE	[BLASTED, I	TRINITY_DN88027_c3_g6_i4	retrotransposon protein	546	20
TRUE	[BLASTED, I	TRINITY_DN88027_c3_g2_i2	30S ribosomal protein S1	564	20
TRUE	[BLASTED, I	TRINITY_DN81861_c1_g1_i16	heat stress transcription	1646	20
TRUE	[BLASTED, I	TRINITY_DN79786_c0_g2_i6	oxalate--CoA ligase-like	1652	20
TRUE	[BLASTED, I	TRINITY_DN79722_c0_g1_i3	aspartic proteinase nepe	1605	20
TRUE	[BLASTED]	TRINITY_DN79777_c1_g1_i3	hemiasterlin resistant pro	1955	20
TRUE	[BLASTED]	TRINITY_DN79702_c0_g5_i2	LOB domain-containing p	1129	20
TRUE	[BLASTED]	TRINITY_DN79705_c0_g2_i7	Protein TAR1	576	20
TRUE	[BLASTED]	TRINITY_DN79705_c0_g2_i8	Protein TAR1	437	20
TRUE	[BLASTED]	TRINITY_DN85074_c1_g2_i6	uncharacterized protein /	3903	20
TRUE	[BLASTED, I	TRINITY_DN85006_c1_g1_i3	RNA-dependent RNA poly	1769	20
TRUE	[BLASTED, I	TRINITY_DN85068_c2_g1_i6	polyadenylate-binding pr	1302	20
TRUE	[BLASTED]	TRINITY_DN85030_c4_g1_i1	putative Zn2+-binding pro	3218	20
TRUE	[BLASTED, I	TRINITY_DN85090_c1_g2_i5	heavy metal-associated is	921	20
TRUE	[BLASTED, I	TRINITY_DN85621_c1_g1_i3	poly(ADP-ribose) glycohy	1287	20
TRUE	[BLASTED]	TRINITY_DN85628_c2_g1_i10	hypothetical protein F51	849	20
TRUE	[BLASTED, I	TRINITY_DN85636_c0_g1_i17	UDP-galactose transporte	1657	20
TRUE	[BLASTED, I	TRINITY_DN85636_c0_g1_i19	UDP-galactose transporte	1287	20
TRUE	[BLASTED, I	TRINITY_DN85661_c3_g1_i2	Glutaredoxin-related pro	834	20
TRUE	[BLASTED, I	TRINITY_DN85641_c3_g1_i2	homeobox-leucine zipper	2047	20
TRUE	[BLASTED, I	TRINITY_DN84682_c1_g2_i3	CDP-diacylglycerol--serin	2250	20
TRUE	[BLASTED, I	TRINITY_DN84614_c6_g1_i3	UDP-glucose 4-epimerase	1412	20
TRUE	[BLASTED, I	TRINITY_DN86856_c0_g1_i3	probable bifunctional me	1629	20
TRUE	[BLASTED, I	TRINITY_DN86856_c0_g1_i10	probable bifunctional me	1587	20
TRUE	[BLASTED, I	TRINITY_DN86843_c0_g1_i5	Nuclear factor related to	1625	20
TRUE	[BLASTED, I	TRINITY_DN86869_c0_g1_i14	Long chain acyl-CoA synt	1098	20
TRUE	[BLASTED, I	TRINITY_DN86812_c2_g2_i6	muscle M-line assembly p	2948	20
TRUE	[BLASTED, I	TRINITY_DN86896_c0_g1_i14	DDT domain-containing p	1978	20
TRUE	[BLASTED, I	TRINITY_DN86837_c1_g2_i1	ribosomal protein S10	784	20
TRUE	[BLASTED, I	TRINITY_DN88636_c1_g2_i7	protein arginine N-methy	2967	20
TRUE	[BLASTED, I	TRINITY_DN88636_c1_g1_i12	katanin p60 atpase-conta	3821	20
TRUE	[BLASTED, I	TRINITY_DN88675_c1_g1_i25	vacuolar cation/proton e	3995	20
TRUE	[BLASTED, I	TRINITY_DN88618_c2_g1_i1	photosystem I P700 chlor	683	20
TRUE	[BLASTED, I	TRINITY_DN88618_c2_g1_i12	photosystem I P700 apop	1019	20

TRUE	[BLASTED, I	TRINITY_DN88600_c0_g1_i1	signal anchor, putative	264	20
TRUE	[BLASTED, I	TRINITY_DN88600_c6_g4_i3	NAD(P)H-quinone oxidor	1376	20
TRUE	[BLASTED, I	TRINITY_DN88632_c2_g1_i3	E3 ubiquitin-protein ligas	2036	20
TRUE	[BLASTED, I	TRINITY_DN87899_c2_g1_i2	ATPase 10, plasma memb	1146	20
TRUE	[BLASTED, I	TRINITY_DN87854_c1_g1_i1	nuclear poly(A) polymera	4859	20
TRUE	[BLASTED, I	TRINITY_DN87827_c5_g2_i7	mitochondrial uncoupling	1564	20
TRUE	[BLASTED, I	TRINITY_DN76209_c0_g1_i3	DNA mismatch repair pro	2625	20
TRUE	[BLASTED]	TRINITY_DN76217_c1_g1_i4	Zinc finger, FYVE/PHD-ty	1651	20
TRUE	[BLASTED]	TRINITY_DN76217_c1_g1_i9	Zinc finger, FYVE/PHD-ty	1681	20

of ON- *versus* OFF-crop trees

d Zeinalabedini[5] and Seyed Alireza Salami[6]

ples ON- vs. OFF-trees

e-Value	sim mean	#GO	GO IDs	GO Names
0	68.73	5	F:GO:0000166; F:GO:0	F:nucleotid
0	85.44	1	F:GO:0003677	F:DNA bind
2.17E-80	60.29	3	F:GO:0004144; F:GO:0	F:diacylglyc
6.10E-153	98.16	10	F:GO:0005506; C:GO:0	F:iron ion b
0	79.9			
2.90E-42	87.24	6	P:GO:0000462; C:GO:0	P:maturatio
2.29E-79	90.48	7	F:GO:0005506; F:GO:0	F:iron ion b
8.63E-89	91.92	2	F:GO:0016787; P:GO:0	F:hydrolase
0	88.38	7	F:GO:0004420; C:GO:0	F:hydroxym
4.09E-68	80.58	4	F:GO:0004674; F:GO:0	F:protein se
1.18E-65	77.64			
0	82.09	5	F:GO:0008171; F:GO:0	F:O-methyl
0	85.46	3	F:GO:0004252; P:GO:0	F:serine-typ
0	51.68	3	F:GO:0003824; F:GO:0	F:catalytic a
1.71E-82	95.9	4	F:GO:0030267; F:GO:0	F:glyoxylate
0	83.2	1	P:GO:0006355	P:regulation
0	80.73			
0	81.31	9	F:GO:0004674; F:GO:0	F:protein se
0	91.71	2	F:GO:0003837; P:GO:0	F:beta-urei
2.13E-131	73.56	4	F:GO:0004497; P:GO:0	F:monooxy
0	99.21	8	C:GO:0009523; C:GO:0	C:photosys
0	80.89			
0	92.69			
1.73E-94	69.81	1	F:GO:0005488	F:binding
0	79.23	4	F:GO:0003684; C:GO:0	F:damaged
1.26E-176	93.58	8	F:GO:0004553; C:GO:0	F:hydrolase
0	89.91			
2.02E-159	80.38	2	F:GO:0048040; F:GO:0	F:UDP-gluc
0	90.4	4	F:GO:0016702; P:GO:0	F:oxidoredu
3.38E-158	72.34	1	P:GO:0055072	P:iron ion h
1.91E-105	72.69			
1.26E-113	66.44	1	F:GO:0016874	F:ligase act
0	84.52			
1.61E-135	91.18	4	C:GO:0016021; P:GO:0	C:integral c
0	83.56	2	C:GO:0009507; F:GO:0	C:chloropla
0	61.41	3	F:GO:0003677; C:GO:0	F:DNA bind
9.52E-149	82.17	4	C:GO:0009507; P:GO:0	C:chloropla
1.89E-156	81.89	5	F:GO:0004014; C:GO:0	F:adenosyl
4.99E-99	63.44	1	F:GO:0016740	F:transfera
1.54E-52	75.66	1	C:GO:0016021	C:integral c
1.08E-58	97.17	2	P:GO:0000413; F:GO:0	P:protein p
6.90E-178	75.06	1	C:GO:0016020	C:membrar

1.00E-69	80.64	3	F:GO:0004497; P:GO:0	F:monooxy
1.38E-41	70.69	2	F:GO:0016740; F:GO:0	F:transferas
5.59E-85	83.48	6	F:GO:0005524; P:GO:0	F:ATP bindi
7.88E-79	93.87	7	P:GO:0000186; F:GO:0	P:activatior
5.89E-165	81.86	2	P:GO:0008152; F:GO:0	P:metabolic
0	90.65			
1.45E-68	76.48			
0	71.87	1	F:GO:0016301	F:kinase act
0	70.25	4	F:GO:0003677; F:GO:0	F:DNA bind
1.05E-165	96.4	3	F:GO:0004128; C:GO:0	F:cytochror
1.94E-144	90.12	6	F:GO:0004322; P:GO:0	F:ferroxida
0	89.55	4	F:GO:0005337; C:GO:0	F:nucleosid
0	80.85	3	C:GO:0010008; P:GO:0	C:endosom
0	70.69	2	F:GO:0003676; F:GO:0	F:nucleic ac
5.63E-71	92.14	7	F:GO:0004349; F:GO:0	F:glutamate
1.90E-101	67.91	1	F:GO:0016740	F:transferas
0	89.91	5	C:GO:0000118; F:GO:0	C:histone d
0	76.41	7	F:GO:0004672; F:GO:0	F:protein ki
0	94.45	1	F:GO:0005524	F:ATP bindi
0	84.66	7	F:GO:0004553; C:GO:0	F:hydrolase
1.13E-101	99.43	1	F:GO:0005524	F:ATP bindi
0	86.87	3	F:GO:0008253; P:GO:0	F:5'-nucleo
0	79.43	7	F:GO:0004573; C:GO:0	F:mannosy
0	86.68	3	F:GO:0004674; F:GO:0	F:protein se
0	94.76	2	P:GO:0006355; F:GO:0	P:regulatior
0	91.58	2	F:GO:0005524; F:GO:0	F:ATP bindi
0	72.6	1	F:GO:0003677	F:DNA bind
0	88.98	1	F:GO:0047274	F:galactino
9.81E-37	83.53	4	F:GO:0003824; C:GO:0	F:catalytic a
0	79.06	2	C:GO:0000795; P:GO:0	C:synapton
0	76.86	2	C:GO:0000795; P:GO:0	C:synapton
5.03E-148	88.46	4	C:GO:0005789; C:GO:0	C:endoplas
1.25E-155	92.13	5	F:GO:0004821; F:GO:0	F:histidine-
0	93.9	1	C:GO:0016021	C:integral c
0	94.1	1	P:GO:0006897	P:endocyto
0	73.04	2	F:GO:0003743; P:GO:0	F:translatio
0	84.44	6	P:GO:0001732; F:GO:0	P:formatior
0	88.73	3	C:GO:0005786; P:GO:0	C:signal rec
7.22E-12	100			
3.76E-36	85.12	1	C:GO:0016021	C:integral c
0	87.64	3	F:GO:0003746; C:GO:0	F:translatio
0	75.35			
5.93E-41	72.38			
8.85E-129	89.91	8	C:GO:0000139; F:GO:0	C:Golgi me
0	87.72	3	P:GO:0008152; C:GO:0	P:metabolic
0	95.69	2	F:GO:0004030; P:GO:0	F:aldehyde
1.85E-148	88.89	15	C:GO:0005743; P:GO:0	C:mitochon
1.08E-139	94.83	3	P:GO:0006629; C:GO:0	P:lipid meta
9.23E-139	76.34	1	F:GO:0005488	F:binding

0	100	10	F:GO:0000287; F:GO:0	F:magnesiu
1.38E-126	63.17			
8.93E-152	74.7	1	F:GO:0046983	F:protein di
3.77E-107	73.07	1	F:GO:0046983	F:protein di
0	91.37	12	F:GO:0008703; F:GO:0	F:5-amino-
0	93.38	8	C:GO:0000221; C:GO:0	C:vacuolar
0	91.03	8	P:GO:0001676; F:GO:0	P:long-chai
0	87.4	10	F:GO:0003677; C:GO:0	F:DNA bind
0	91.99	4	F:GO:0008168; F:GO:0	F:methyltra
0	74.79	3	F:GO:0016787; P:GO:0	F:hydrolase
0	80.05	4	F:GO:0005524; C:GO:0	F:ATP bindi
0	80.05	4	F:GO:0005524; C:GO:0	F:ATP bindi
1.42E-11	96.3			
1.09E-130	79.16	1	C:GO:0009507	C:chloropla
3.92E-157	87.5	1	C:GO:0016021	C:integral c
4.07E-67	84.67	2	C:GO:0005739; F:GO:0	C:mitochon
0	77.75	4	C:GO:0005737; C:GO:0	C:cytoplasn
0	96.22	16	C:GO:0005739; P:GO:0	C:mitochon
0	81.07	8	F:GO:0004575; C:GO:0	F:sucrose a
0	98.52	6	F:GO:0005524; C:GO:0	F:ATP bindi
0	85.83	6	F:GO:0004497; F:GO:0	F:monooxy
0	85.37			
1.47E-66	96.46	6	F:GO:0004675; F:GO:0	F:transmen
8.62E-86	85.49	4	C:GO:0005739; C:GO:0	C:mitochon
4.77E-88	86.37	3	C:GO:0005739; C:GO:0	C:mitochon
0	99.58	6	C:GO:0005743; P:GO:0	C:mitochor
0	96.52	3	F:GO:0003676; F:GO:0	F:nucleic ac
3.07E-49	89.59			
1.49E-43	85.49	1	C:GO:0019028	C:viral caps
0	80.56	2	F:GO:0016740; F:GO:0	F:transfera
9.48E-104	60.74	3	F:GO:0004674; F:GO:0	F:protein se
3.01E-70	76.22	2	C:GO:0016021; P:GO:0	C:integral c
3.36E-157	60.73	2	C:GO:0005622; F:GO:0	C:intracellu
1.15E-56	96.85	2	F:GO:0004519; P:GO:0	F:endonucl
8.43E-56	95.99	2	F:GO:0004519; P:GO:0	F:endonucl
9.57E-34	98.36	2	F:GO:0004519; P:GO:0	F:endonucl
9.20E-61	94.42	2	F:GO:0004519; P:GO:0	F:endonucl
1.51E-41	83.85	1	C:GO:0016021	C:integral c
3.72E-24	44.56	9	F:GO:0000166; F:GO:0	F:nucleotid
5.40E-178	84.36	5	C:GO:0005769; C:GO:0	C:early end
0	76.08	3	C:GO:0005886; C:GO:0	C:plasma m
2.55E-69	98.54	5	F:GO:0003924; F:GO:0	F:GTPase a
2.65E-117	93.91	5	F:GO:0004777; F:GO:0	F:succinate
1.80E-157	77.24			
9.56E-86	95.38	7	F:GO:0003735; F:GO:0	F:structura
6.65E-142	54.74	1	F:GO:0016884	F:carbon-ni
0	82.58	1	C:GO:0016021	C:integral c
2.04E-165	89.79	3	F:GO:0015369; C:GO:0	F:calcium:p
0	89.44	6	C:GO:0005737; P:GO:0	C:cytoplasr

0	80.55	2	F:GO:0005524; F:GO:0	F:ATP bindi
0	91.33	1	F:GO:0005524	F:ATP bindi
0	71.62			
1.94E-95	84.22	8	F:GO:0003700; C:GO:0	F:DNA-bind
0	68.21	5	C:GO:0005737; P:GO:0	C:cytoplasn
0	55	5	F:GO:0005524; C:GO:0	F:ATP bindi
4.37E-86	88.91	2	C:GO:0009543; P:GO:0	C:chloropla
0	89.75	3	C:GO:0005634; C:GO:0	C:nucleus; (
3.00E-58	93.16	2	F:GO:0004789; C:GO:0	F:thiamine-
1.37E-93	96.7	4	C:GO:0016021; F:GO:0	C:integral c
0	72.9	3	F:GO:0003700; P:GO:0	F:DNA-bind
4.90E-40	79.76	4	F:GO:0003676; F:GO:0	F:nucleic ac
0	85.3	4	C:GO:0005774; C:GO:0	C:vacuolar
5.65E-54	95.44	2	C:GO:0005739; C:GO:0	C:mitochon
6.95E-66	55.33			
4.18E-22	61.65			
2.25E-124	94.96	10	F:GO:0003735; F:GO:0	F:structural
0	85.18	11	C:GO:0005829; P:GO:0	C:cytosol; F
1.09E-167	86.49	1	F:GO:0004806	F:triglycerid
0	93.53			
0	74.89	1	F:GO:0005516	F:calmoduli
1.38E-74	61.7			
1.99E-177	98.47			
0	83.89	2	C:GO:0005634; P:GO:0	C:nucleus; I
0	83.17	6	F:GO:0003713; F:GO:0	F:transcript
2.40E-134	94.26	4	C:GO:0005737; P:GO:0	C:cytoplasn
3.89E-75	79.02	1	F:GO:0016787	F:hydrolase
0	77.72	1	F:GO:0043531	F:ADP bindi
0	87.22	5	F:GO:0004693; F:GO:0	F:cyclin-dep
0	89.52	3	C:GO:0016021; F:GO:0	C:integral c
7.47E-55	94.62	4	F:GO:0003677; C:GO:0	F:DNA bind
2.65E-77	68.58			
0	78.07	1	C:GO:0016020	C:membrar
0	91.41	4	F:GO:0004672; F:GO:0	F:protein ki
6.01E-100	94.37	4	F:GO:0005509; C:GO:0	F:calcium ic
3.09E-129	91.27	3	C:GO:0005744; P:GO:0	C:mitochon
1.44E-91	98.19	5	F:GO:0003746; F:GO:0	F:translatic
4.88E-152	83.72	1	P:GO:0031047	P:gene siler
4.30E-125	96.57	2	F:GO:0005524; P:GO:0	F:ATP bindi
1.20E-95	97.46	6	F:GO:0005515; C:GO:0	F:protein bi
0	72.31	1	F:GO:0003677	F:DNA bind
0	73.27	4	F:GO:0004527; F:GO:0	F:exonucle
0	96.18	6	F:GO:0004455; P:GO:0	F:ketol-acic
0	96.39	2	F:GO:0008928; P:GO:0	F:mannose
0	75.36			
0	84.54	7	P:GO:0000103; F:GO:0	P:sulfate as
0	70.54	3	P:GO:0015031; F:GO:0	P:protein tr
1.50E-157	85.76	1	C:GO:0016021	C:integral c

0	88.42	3	F:GO:0017112; P:GO:0	F:Rab guan
0	65.01	1	P:GO:0042752	P:regulatio
0	66.25	1	P:GO:0042752	P:regulatio
1.30E-133	65.55	1	P:GO:0042752	P:regulatio
3.08E-27	89.95	3	C:GO:0009507; C:GO:0	C:chloropla
6.52E-69	96.2	1	C:GO:0009507	C:chloropla
0	91.94	5	C:GO:0005737; C:GO:0	C:cytoplasn
0	63.33			
8.03E-143	77.4			
8.37E-109	78.24	2	F:GO:0016301; P:GO:0	F:kinase act
0	94.3	2	F:GO:0016301; P:GO:0	F:kinase act
0	79.88	6	F:GO:0003723; F:GO:0	F:RNA bind
0	79.88	6	F:GO:0003723; F:GO:0	F:RNA bind
2.26E-101	89.64	7	C:GO:0000243; P:GO:0	C:commitm
0	96.93	5	F:GO:0003735; C:GO:0	F:structural
1.60E-126	83.57	3	P:GO:0007033; P:GO:0	P:vacuole o
0	83.83	5	F:GO:0005524; F:GO:0	F:ATP bindi
6.59E-96	82.55	3	F:GO:0003677; C:GO:0	F:DNA bind
0	94.09	5	F:GO:0005524; P:GO:0	F:ATP bindi
3.24E-105	83.01			
3.45E-179	90.26	6	F:GO:0004322; C:GO:0	F:ferroxida
0	88.42	1	C:GO:0072546	C:ER memb
1.55E-133	80.82	1	F:GO:0008270	F:zinc ion b
0	64.72	1	C:GO:0043229	C:intracellu
0	64.41			
3.03E-29	77.41	1	C:GO:0016021	C:integral c
7.90E-46	99.61	4	F:GO:0003735; F:GO:0	F:structural
2.74E-142	84.04	4	F:GO:0004784; P:GO:0	F:superoxic
1.59E-64	94.99			
0	63.15	5	F:GO:0003677; F:GO:0	F:DNA bind
4.68E-103	78.87	2	P:GO:0009269; C:GO:0	P:response
0	73.59	3	F:GO:0003677; C:GO:0	F:DNA bind
2.07E-76	86.9	4	C:GO:0005886; C:GO:0	C:plasma m
0	77.85	3	F:GO:0004842; P:GO:0	F:ubiquitin-
0	91.23	4	F:GO:0004190; P:GO:0	F:aspartic-t
0	59.9	2	F:GO:0097159; F:GO:1	F:organic cy
0	72.69	1	F:GO:0043531	F:ADP bindi
5.69E-50	60.74	3	F:GO:0003899; C:GO:0	F:DNA-dire
1.75E-156	94.66	2	F:GO:0042626; P:GO:0	F:ATPase ac
0	92.8	3	F:GO:0003677; C:GO:0	F:DNA bind
1.32E-161	88.31	4	F:GO:0004559; P:GO:0	F:alpha-ma
0	73.15			
1.61E-117	94.66	4	F:GO:0000829; P:GO:0	F:inositol h
8.36E-142	96.1	7	F:GO:0005524; F:GO:0	F:ATP bindi
0	95.18	1	P:GO:0006904	P:vesicle dc
1.35E-99	91.46	2	C:GO:0005634; C:GO:0	C:nucleus; (
8.91E-146	91.03	2	C:GO:0005634; P:GO:0	C:nucleus; I
3.31E-40	96.4	1	C:GO:0016021	C:integral c
1.24E-60	83.39	5	C:GO:0005576; C:GO:0	C:extracellu
0	88.63	6	P:GO:0006782; F:GO:0	P:protopor

0	90.03	3	F:GO:0004674; F:GO:0	F:protein s
0	86.47	2	F:GO:0004252; P:GO:0	F:serine-typ
1.22E-174	99.36	4	F:GO:0003735; C:GO:0	F:structural
1.44E-157	75.05			
0	99.57	8	F:GO:0005506; C:GO:0	F:iron ion b
2.38E-151	82.2	3	F:GO:0008270; C:GO:0	F:zinc ion b
1.56E-153	98.47	12	F:GO:0005524; C:GO:0	F:ATP bindi
3.54E-116	92.45	5	F:GO:0016788; F:GO:0	F:hydrolase
1.06E-09	100			
0	98.38	5	F:GO:0003723; P:GO:0	F:RNA bind
0	100	13	F:GO:0005506; C:GO:0	F:iron ion b
2.20E-100	71.34	3	P:GO:0010215; C:GO:0	P:cellulose
5.76E-30	89.15	7	C:GO:0000276; F:GO:0	C:mitochor
1.43E-74	75.74	4	P:GO:0008152; C:GO:0	P:metaboli
0	90.46	1	C:GO:0016021	C:integral c
0	97.21	4	F:GO:0005524; C:GO:0	F:ATP bindi
1.32E-68	74.54	2	C:GO:0016020; C:GO:0	C:membrar
0	88.22	3	F:GO:0003723; F:GO:0	F:RNA bind
8.76E-93	83.63	1	C:GO:0016021	C:integral c
1.14E-143	77.45	5	F:GO:0003677; C:GO:0	F:DNA bind
0	65.66	2	F:GO:0003677; C:GO:0	F:DNA bind
0	79.12	6	C:GO:0005622; C:GO:0	C:intracellu
0	99.22	5	F:GO:0008137; C:GO:0	F:NADH de
6.04E-134	89.93	9	C:GO:0005654; P:GO:0	C:nucleopla
0	99.65	7	F:GO:0000287; F:GO:0	F:magnesiu
0	93.1	3	C:GO:0000145; P:GO:0	C:exocyst;
4.11E-79	99.55	7	P:GO:0000028; P:GO:0	P:ribosoma
0	66.6	1	F:GO:0003677	F:DNA bind
0	64.71	1	F:GO:0046983	F:protein di
0	82.8	5	C:GO:0005634; C:GO:0	C:nucleus;
0	83.98	2	P:GO:0006355; F:GO:0	P:regulatio
3.33E-85	94.57			
0	85.09	4	F:GO:0003995; P:GO:0	F:acyl-CoA
0	83.07	5	F:GO:0003995; F:GO:0	F:acyl-CoA
0	64.28	9	F:GO:0003700; F:GO:0	F:DNA-bind
9.85E-66	77.19	12	C:GO:0000923; F:GO:0	C:equatoria
3.16E-68	87.47	3	C:GO:0005739; C:GO:0	C:mitochon
6.95E-95	81.46	3	C:GO:0005739; C:GO:0	C:mitochon
7.99E-09	66.29	2	C:GO:0005874; F:GO:0	C:microtub
1.39E-41	80.36	1	C:GO:0016021	C:integral c
2.49E-82	92.84	10	F:GO:0000175; C:GO:0	F:3'-5'-exor
3.26E-151	79.44			
1.85E-114	79.59	6	F:GO:0004322; C:GO:0	F:ferroxida
1.66E-94	94.11	6	F:GO:0004322; C:GO:0	F:ferroxida
8.63E-119	92.74	6	F:GO:0004322; C:GO:0	F:ferroxida
4.50E-45	90.12	6	F:GO:0004322; C:GO:0	F:ferroxida
6.33E-111	93.39	6	F:GO:0004322; C:GO:0	F:ferroxida
0	90.91	4	C:GO:0016021; F:GO:0	C:integral c
5.04E-89	84.63	5	P:GO:0000390; F:GO:0	P:spliceoso

0	87.98	4	F:GO:0004222; P:GO:0	F:metalloe
0	66.48	5	F:GO:0004674; F:GO:0	F:protein s
2.89E-73	58.1	8	F:GO:0003676; F:GO:0	F:nucleic ac
0	84.24			
2.19E-89	88.13	7	P:GO:0000186; F:GO:0	P:activatio
0	94.88	6	P:GO:0000165; F:GO:0	P:MAPK ca
0	94.62	7	F:GO:0004674; F:GO:0	F:protein s
2.24E-09	100	6	F:GO:0005506; C:GO:0	F:iron ion b
0	83.24	7	F:GO:0004857; C:GO:0	F:enzyme i
0	69.16	1	P:GO:0006281	P:DNA repa
0	91.41	2	P:GO:0006355; F:GO:0	P:regulatio
0	93.75	3	F:GO:0015299; C:GO:0	F:solute:pr
4.50E-30	85.26			
9.87E-131	63.86	1	F:GO:0016740	F:transfera
1.21E-17	77.68	2	C:GO:0005886; P:GO:0	C:plasma m
0	91.31	6	F:GO:0005509; C:GO:0	F:calcium ic
0	90.55	3	C:GO:0016021; F:GO:0	C:integral c
1.12E-133	79.16	4	C:GO:0016020; C:GO:0	C:membrar
1.90E-136	91.2	3	F:GO:0004722; P:GO:0	F:protein s
0	92.89	3	F:GO:0003924; F:GO:0	F:GTPase a
4.59E-91	86.47	2	C:GO:0046658; P:GO:0	C:anchored
9.82E-73	79.34			
4.08E-51	97.24	1	F:GO:0003723	F:RNA bind
0	92.69	3	C:GO:0005768; C:GO:0	C:endosom
6.35E-130	99.39	4	C:GO:0005743; C:GO:0	C:mitochon
2.23E-137	94.59	4	F:GO:0004693; F:GO:0	F:cyclin-de
0	92.62	5	F:GO:0004693; F:GO:0	F:cyclin-de
0	78.25	7	P:GO:0000186; F:GO:0	P:activatio
3.99E-132	80.04	4	F:GO:0004556; F:GO:0	F:alpha-am
0	92.09	4	F:GO:0004556; F:GO:0	F:alpha-am
0	89.16	1	F:GO:0047274	F:galactino
0	88.55	3	F:GO:0004805; P:GO:0	F:trehalose
0	82.51	1	C:GO:0016021	C:integral c
0	73.36			
0	87.93	3	P:GO:0016573; P:GO:0	P:histone a
2.21E-143	96.71	1	F:GO:0005509	F:calcium ic
5.08E-43	79.41			
0	73.49			
0	79.68			
0	86.18			
7.55E-163	63.92	5	F:GO:0003677; F:GO:0	F:DNA bind
1.15E-104	97.2	10	F:GO:0000287; F:GO:0	F:magnesiu
1.12E-147	84.8	5	F:GO:0004601; P:GO:0	F:peroxidas
0	84.53	4	F:GO:0004674; F:GO:0	F:protein s
4.56E-47	97.6	3	P:GO:0002181; F:GO:0	P:cytoplasn
0	92.26	5	P:GO:0000413; F:GO:0	P:protein p
6.10E-57	86.4	3	F:GO:0003824; C:GO:0	F:catalytic a
1.06E-153	91.53	7	C:GO:0005774; C:GO:0	C:vacuolar
0	80.26	1	C:GO:0016021	C:integral c
3.41E-114	95.34	3	F:GO:0003839; P:GO:0	F:gamma-g

7.37E-124	99.25	8	P:GO:0000054; F:GO:0	P:ribosoma
1.09E-83	90.27	3	P:GO:0006637; F:GO:0	P:acyl-CoA
4.02E-172	88.27	3	F:GO:0004386; F:GO:0	F:helicase a
0	58.47	2	C:GO:0016020; C:GO:0	C:membrar
0	86.23	4	F:GO:0005524; C:GO:0	F:ATP bindi
1.73E-74	78.95			
3.73E-125	80.76	3	F:GO:0005524; F:GO:0	F:ATP bindi
1.01E-43	82.97	6	F:GO:0004322; C:GO:0	F:ferroxida
5.00E-78	92.87	6	F:GO:0004322; C:GO:0	F:ferroxida
4.79E-121	89.04	6	F:GO:0004322; C:GO:0	F:ferroxida
2.04E-119	88.87	6	F:GO:0004322; C:GO:0	F:ferroxida
2.15E-73	84.98	6	F:GO:0004322; C:GO:0	F:ferroxida
2.43E-130	94.85	2	F:GO:0004535; P:GO:0	F:poly(A)-sp
4.47E-12	94.74			
1.52E-156	93.21	4	C:GO:0005576; C:GO:0	C:extracellu
6.34E-41	87.65	4	P:GO:0008152; C:GO:0	P:metabolic
2.01E-40	79.08	2	C:GO:0016021; P:GO:0	C:integral c
2.50E-44	88.95	1	C:GO:0016021	C:integral c
4.39E-99	85.87			
0	82.5	5	F:GO:0003723; F:GO:0	F:RNA bind
0	88.4	5	F:GO:0003723; F:GO:0	F:RNA bind
0	92.77	1	C:GO:0016021	C:integral c
0	94.98	9	C:GO:0005618; C:GO:0	C:cell wall;
0	85.49	13	F:GO:0000404; F:GO:0	F:heterodu
0	82.73	5	F:GO:0003723; F:GO:0	F:RNA bind
1.40E-13	76.64	2	F:GO:0004722; P:GO:0	F:protein se
0	88.44	3	F:GO:0000287; P:GO:0	F:magnesiu
0	59.23	5	F:GO:0003676; F:GO:0	F:nucleic ac
1.14E-61	91.37	4	F:GO:0003743; F:GO:0	F:translatic
0	82.85	2	F:GO:0003676; F:GO:0	F:nucleic ac
2.14E-116	56.06			
5.92E-66	97.7	10	C:GO:0009523; C:GO:0	C:photosys
3.12E-141	82.03			
0	93.48	12	F:GO:0004471; F:GO:0	F:malate de
0	89.84	11	F:GO:0003677; F:GO:0	F:DNA bind
0	78.06	2	F:GO:0004842; P:GO:0	F:ubiquitin-
0	99.4	4	F:GO:0004402; C:GO:0	F:histone a
0	78.64	4	F:GO:0003700; C:GO:0	F:DNA-bind
0	86.99	4	C:GO:0005886; C:GO:0	C:plasma m
2.64E-136	79.2	4	F:GO:0004672; F:GO:0	F:protein ki
3.90E-70	98.12	2	C:GO:0005737; P:GO:0	C:cytoplasm
0	77.35	3	F:GO:0005524; P:GO:0	F:ATP bindi
2.34E-123	91.1	2	C:GO:0016021; F:GO:0	C:integral c
0	74.34	2	C:GO:0009941; C:GO:0	C:chloropla
8.64E-60	95.92	6	F:GO:0005524; C:GO:0	F:ATP bindi
3.02E-19	79.87	2	C:GO:0005789; C:GO:0	C:endoplas
2.20E-35	87.96	2	F:GO:0008375; C:GO:0	F:acetylgluc
5.50E-112	94.87			
0	98.49	15	P:GO:0002098; F:GO:0	P:tRNA wol
0	67.22	3	F:GO:0003729; F:GO:0	F:mRNA bir

3.93E-157	89.7	4	F:GO:0005096; C:GO:0	F:GTPase a
1.06E-149	93.69	3	P:GO:0016226; F:GO:0	P:iron-sulfu
0	98	8	F:GO:0004601; C:GO:0	F:peroxidas
6.07E-67	92.75	8	F:GO:0004791; C:GO:0	F:thioredox
1.41E-56	92.02	3	C:GO:0000813; P:GO:0	C:ESCRT I c
1.89E-135	92	2	P:GO:0006890; C:GO:0	P:retrograd
2.93E-135	92	2	P:GO:0006890; C:GO:0	P:retrograd
1.05E-141	82.46	5	F:GO:0001046; F:GO:0	F:core pron
3.46E-49	78.79	1	F:GO:0016787	F:hydrolase
3.79E-152	92.33	4	C:GO:0005634; C:GO:0	C:nucleus;
0	73.88	4	P:GO:0006259; F:GO:0	P:DNA met
0	73.74	4	P:GO:0006259; F:GO:0	P:DNA met
0	65.42	3	F:GO:0003677; F:GO:0	F:DNA bind
1.52E-93	77.26	3	C:GO:0016021; F:GO:0	C:integral c
1.27E-148	73.05			
5.16E-59	91.82	4	F:GO:0004866; C:GO:0	F:endopept
3.03E-52	89.78	1	C:GO:0016021	C:integral c
1.12E-149	78.29	1	F:GO:0003677	F:DNA bind
1.20E-35	96.3	10	C:GO:0009506; C:GO:0	C:plasmode
0	84.61	3	F:GO:0004190; P:GO:0	F:aspartic-t
3.23E-49	90.75			
6.54E-145	80.85			
1.97E-36	80.05			
3.53E-37	78.09			
0	63.72			
0	83.67	10	P:GO:0001172; F:GO:0	P:transcrip
4.12E-85	92.86	2	F:GO:0003723; C:GO:0	F:RNA bind
0	88.3			
1.18E-67	72.43	2	P:GO:0030001; F:GO:0	P:metal ion
0	77.48	1	F:GO:0016787	F:hydrolase
1.19E-103	91.71			
0	94.88	1	C:GO:0016021	C:integral c
0	86.19	1	C:GO:0016021	C:integral c
7.73E-26	80.83	5	C:GO:0005623; F:GO:0	C:cell; F:ele
0	88.78	5	C:GO:0005634; P:GO:0	C:nucleus;
0	92.97	5	F:GO:0003882; C:GO:0	F:CDP-diac
8.01E-29	81.05	2	F:GO:0003978; P:GO:0	F:UDP-gluc
0	91.7	10	F:GO:0000287; C:GO:0	F:magnesiu
0	90.22	10	F:GO:0000287; C:GO:0	F:magnesiu
0	63.04	1	C:GO:0031011	C:Ino80 cor
9.85E-171	88.14	3	P:GO:0001676; F:GO:0	P:long-chai
0	55.57	1	F:GO:0005516	F:calmoduli
0	76.29	1	C:GO:0005634	C:nucleus
2.01E-85	94.91	4	F:GO:0005524; C:GO:0	F:ATP bindi
0	85.88	9	C:GO:0005829; P:GO:0	C:cytosol; F
0	85.08	3	F:GO:0005524; C:GO:0	F:ATP bindi
3.96E-122	74.24	4	P:GO:0006816; F:GO:0	P:calcium ic
1.82E-149	99.82	12	C:GO:0009522; C:GO:0	C:photosyst
0	99.84	10	F:GO:0000287; F:GO:0	F:magnesiu

1.36E-31	88.62	4	F:GO:0005524; C:GO:0	F:ATP bindi
2.62E-68	81.41	2	C:GO:0009536; C:GO:0	C:plastid; C
0	78.69	2	F:GO:0004842; P:GO:0	F:ubiquitin-
6.47E-29	92.41	8	F:GO:0005524; C:GO:0	F:ATP bindi
0	86.15	4	F:GO:0003723; F:GO:0	F:RNA bind
3.78E-176	91.24	15	C:GO:0005743; P:GO:0	C:mitochon
0	82.11	7	F:GO:0000404; F:GO:0	F:heterodu
1.24E-43	79.53			
1.30E-43	79.47			

Enzyme Codes
EC:3.6.1.15
ing
:erol O-acyltransferase activity
inding; C:photosystem II;

on of SSU-rRNA from tricistronic rRNA transcript
inding; F:electron transfer activity;

: activity;
EC:1.1.1.34
EC:2.7.11

transferase activity; P:methylation;
EC:3.4.21; EC:3.4.14
activity; F:binding; P:metabolic process

EC:1.1.1.79
n of transcription, DNA-templated

EC:2.7.11
EC:3.5.1.6
genase activity; P:secondary metabolite biosynthetic process;

tem II; C:chloroplast thylakoid membrane;

DNA binding; C:nucleus; P:nucleotide-excision repair;
EC:2.4.1.207

EC:4.1.1.35
EC:1.13.11
omeostasis

ivity

EC:2.1.1.161; EC:2.1.1.162; EC:2.1.1.156; EC:2.1.1.157
st; F:transferase activity
ing; C:nucleus; P:regulation of transcription
st; P:negative regulation of translation;
EC:4.1.1.50
se activity
omponent of membrane
EC:5.2.1.8
ıe

genase activity;
se activity; F:transferase activity,
ng; P:proteolysis; F:peptidase activity;
EC:2.7.11.25; EC:2.7.11
: process; F:transferase activity, transferring hexosyl groups

tivity
ing; F:catalytic activity;
EC:1.6.2.2
EC:1.16.3.1; EC:1.16.3
e transmembrane transporter activity;
e membrane; P:protein transport;
:id binding; F:RNA binding
EC:2.7.2.11; EC:2.7.4.26
se activity
eacetylase complex; F:transcription corepressor activity;
inase activity; F:drug binding;
ng
EC:2.4.1.207
ng
EC:3.1.3.41; EC:3.1.3.5; EC:3.1.3.31
EC:3.2.1.106; EC:3.2.1.21
EC:2.7.11
n of transcription, DNA-templated; F:zinc ion binding
EC:3.6.1.3; EC:3.6.1.15
ing
EC:2.4.1.82
activity; C:chloroplast; C:membrane;
emal complex; P:reciprocal meiotic recombination
emal complex; P:reciprocal meiotic recombination
mic reticulum membrane;
EC:6.1.1.21
omponent of membrane
sis
n initiation factor activity; P:translational initiation
n of cytoplasmic translation initiation complex;
:ognition particle, endoplasmic reticulum targeting;

omponent of membrane
n elongation factor activity;

EC:3.2.1.113; EC:3.2.1.24
: process; C:integral component of membrane; F:ligase activity
EC:1.2.1.5

ndrial inner membrane; P:mitochondrial transport;
EC:3.1.1.23; EC:3.1.1.1

ım ion binding; F:electron transfer activity;

imerization activity
imerization activity
EC:3.5.4.26; EC:1.1.1.193
proton-transporting V-type ATPase,
EC:6.2.1.3
EC:1.5.3.17; EC:1.5.3.16; EC:1.5.3.13
ınsferase activity; F:carbohydrate binding;
activity; P:cellular protein metabolic process;
EC:3.6.1.3; EC:3.6.1.15
EC:3.6.1.3; EC:3.6.1.15

st
omponent of membrane
ıdrion; F:transferase activity
n; C:membrane; P:vesicle-mediated transport;
ıdrion; P:sodium ion transport;
EC:3.2.1.97; EC:3.2.1.20; EC:3.2.1.26; EC:3.2.1.48
EC:3.6.1.3; EC:3.6.1.15
genase activity; F:iron ion binding;

EC:2.7.11

ıdrion; C:chloroplast; C:membrane; P:photosynthesis, light reaction
ıdrion; C:membrane; P:photosynthesis, light reaction
EC:1.10.2; EC:1.10.2.2
EC:3.6.1.15

id
se activity; F:ligase activity
erine/threonine kinase activity; F:ATP binding;
omponent of membrane; P:electron transport chain
lar; F:zinc ion binding
ease activity; P:nucleic acid phosphodiester bond hydrolysis
ease activity; P:nucleic acid phosphodiester bond hydrolysis
ease activity; P:nucleic acid phosphodiester bond hydrolysis
ease activity; P:nucleic acid phosphodiester bond hydrolysis
omponent of membrane
e binding; F:catalytic activity;
osome; C:plasma membrane;
ıembrane; C:integral component of membrane;
EC:3.6.1.15
EC:1.2.1.16; EC:1.2.1.24

EC:6.1.1.2
trogen ligase activity, with glutamine as amido-N-donor
omponent of membrane
roton antiporter activity;
EC:2.4.1.1

ng; F:zinc ion binding
ng

ling transcription factor activity;
n; P:protein ubiquitination;
ng; C:nucleus; P:transcription, DNA-templated;
st thylakoid lumen; P:photosystem II assembly
C:cytosol; C:plasma membrane
EC:2.5.1.3
omponent of membrane;
ling transcription factor activity;
EC:3.1.30; EC:3.1.26; EC:3.1.26.4
membrane; C:proteasome regulatory particle, base subcomplex; C:plasmodesma; P:proteasome regulatory particle ass
drion; C:ribosome

constituent of ribosome; F:copper ion binding;
EC:2.10.1.1; EC:1.7.99.4; EC:2.7.7.75
EC:3.1.1.1; EC:3.1.1.3

in binding

P:regulation of transcription, DNA-templated
tion coactivator activity;
n; P:protein ubiquitination; F:ligase activity; F:ubiquitin protein ligase activity
activity
ing
EC:2.7.11; EC:2.7.11.22
EC:3.2.1.25; EC:3.2.1.78
ing; C:nucleus;

ne
nase activity; F:ATP binding;
on binding; C:photosystem II oxygen evolving complex;
drial inner membrane presequence translocase complex;
EC:3.6.1.15
ncing by RNA
ng; P:protein metabolic process

inding; C:integral component of plasma membrane;
ing
ase activity; F:binding;
EC:1.1.1.86

EC:2.7.7.22

EC:2.7.7.4; EC:2.7.1.25
ansport; F:kinase activity; P:phosphorylation
omponent of membrane

yl-nucleotide exchange factor activity;
n of circadian rhythm
n of circadian rhythm
n of circadian rhythm
st; C:integral component of membrane; F:hydrolase activity
st
n; C:SCF ubiquitin ligase complex;

tivity; P:phosphorylation
tivity; P:phosphorylation
ing; F:endonuclease activity;
ing; F:endonuclease activity;
nent complex; P:mRNA splicing,
l constituent of ribosome;
rganization; P:phosphatidylinositol-3-phosphate biosynthetic process;
EC:3.6.1.3; EC:3.6.3.43; EC:3.6.1.15
ing; C:nucleus; P:regulation of transcription, DNA-templated
ng; P:protein folding; P:proteolysis;

EC:1.16.3.1; EC:1.16.3
rane protein complex
inding
lar organelle

omponent of membrane
l constituent of ribosome;
EC:1.15.1.1

ing; F:DNA-binding transcription factor activity;
to desiccation; C:membrane
ing; C:nucleus; P:regulation of transcription, DNA-templated
nembrane; C:integral component of membrane;
-protein transferase activity; P:protein ubiquitination; F:ligase activity
EC:3.4.23
yclic compound binding;
ing
cted 5'-3' RNA polymerase activity;
EC:3.6.1.3; EC:3.6.1.15
ing; C:nucleus; F:lipid binding
EC:3.2.1.24

EC:2.7.4.24
EC:3.6.1.3; EC:3.6.3.25; EC:3.6.1.15
ocking involved in exocytosis
C:cytoplasm
P:nucleosome assembly
omponent of membrane
ular region; C:monolayer-surrounded lipid storage body;
EC:1.2.1.70

EC:2.7.11
EC:3.4.21
l constituent of ribosome;

inding; C:chloroplast thylakoid membrane;
inding; C:integral component of membrane; F:ligase activity
EC:3.6.1.3; EC:3.6.1.15
EC:1.3.5.6

ing; P:mRNA processing;
inding; C:photosystem II;
microfibril organization;
EC:3.6.1.3; EC:3.6.1.15
c process; C:intracellular membrane-bounded organelle;
omponent of membrane
ng; C:integral component of membrane;
ne; C:integral component of membrane
EC:3.6.1.15
omponent of membrane
ing; C:nucleus;
ing; C:nucleus

EC:1.1.3.20
EC:1.6.99.5; EC:1.6.99.3; EC:1.6.5.3
asm; P:regulation of transcription, DNA-templated; P:response to cold;
EC:4.1.1.39
P:exocytosis; P:vesicle docking
l small subunit assembly;
ing
imerization activity
EC:2.1.1.43
n of transcription, DNA-templated; F:zinc ion binding

EC:1.3.99.3
EC:1.3.99.3
ling transcription factor activity;
al microtubule organizing center;
ndrion; C:integral component of membrane; P:electron transport chain
ndrion; C:integral component of membrane; P:electron transport chain
ule; F:microtubule binding
omponent of membrane
EC:3.1.13; EC:3.1.15; EC:3.1.13.1

EC:1.16.3.1; EC:1.16.3
EC:1.16.3.1; EC:1.16.3
EC:1.16.3.1; EC:1.16.3
EC:1.16.3.1; EC:1.16.3
EC:1.16.3.1; EC:1.16.3
EC:1.6.5.4
mal complex disassembly;

EC:3.4.24
EC:2.7.11
:id binding; F:endonuclease activity;

EC:2.7.11.25; EC:2.7.11
EC:2.7.11; EC:2.7.11.24
EC:2.7.11
inding; C:integral component of membrane;
EC:3.1.1.11; EC:3.1.1.1
ir
n of transcription, DNA-templated; F:zinc ion binding
oton antiporter activity;

se activity
embrane; P:response to stress
on binding; C:Golgi apparatus;
omponent of membrane; F:transmembrane transporter activity;
ie; C:integral component of membrane;
EC:3.1.3.16; EC:3.1.3.41
EC:3.6.1.15
l component of plasma membrane;

ing
e; C:trans-Golgi network; C:integral component of membrane
drial inner membrane; C:integral component of membrane;
EC:2.7.11; EC:2.7.11.22
EC:2.7.11; EC:2.7.11.22
EC:2.7.11.25; EC:2.7.11
EC:3.2.1.1
EC:3.2.1.1
EC:2.4.1.82
EC:3.1.3.12; EC:3.1.3.41
omponent of membrane

cetylation; P:regulation of DNA methylation; P:regulation of DNA demethylation
on binding

ing; F:DNA-binding transcription factor activity;
m ion binding; F:electron transfer activity; C:photosystem I;
EC:1.11.1.7
EC:2.7.11
nic translation; F:structural constituent of ribosome;
EC:5.2.1.8
activity; C:chloroplast; F:metal ion binding
EC:3.4.11
omponent of membrane
EC:4.3.2.9

EC:3.6.1.15
EC:3.1.2.20; EC:3.1.2.2
EC:3.6.1.15
ne; C:integral component of membrane
EC:3.6.1.3; EC:3.6.1.15

ng; F:transferase activity; F:ligase activity
EC:1.16.3.1; EC:1.16.3
EC:1.16.3.1; EC:1.16.3
EC:1.16.3.1; EC:1.16.3
EC:1.16.3.1; EC:1.16.3
EC:1.16.3.1; EC:1.16.3
EC:3.1.13; EC:3.1.15; EC:3.1.13.4

ular region; C:cell wall; P:plant-type cell wall organization; C:membrane
c process; C:chloroplast stroma; C:chloroplast envelope;
omponent of membrane;
omponent of membrane

EC:3.6.1.3; EC:3.6.1.15
EC:3.6.1.3; EC:3.6.1.15
omponent of membrane
C:nucleus; C:cytosol; C:plasma membrane;
EC:3.6.1.3; EC:3.6.1.15
ing; F:endonuclease activity; P:RNA modification;
EC:3.1.3.16; EC:3.1.3.41
m ion binding; P:metabolic process;
cid binding; F:deoxyribodipyrimidine photo-lyase activity;
EC:3.6.1.15
cid binding; F:RNA binding

tem II; C:chloroplast thylakoid membrane;

EC:1.1.1.38; EC:1.1.1.39
EC:3.6.1.3; EC:3.6.1.15
-protein transferase activity; P:protein ubiquitination
EC:2.3.1.5; EC:2.3.1.48
ling transcription factor activity; C:nucleus;
nembrane; C:integral component of membrane;
inase activity; F:ATP binding;
n; P:ER to Golgi vesicle-mediated transport
ng; P:proteolysis; F:peptidase activity
omponent of membrane;
st envelope; C:membrane
ng; C:cytoplasm;
mic reticulum membrane; C:integral component of membrane
cosaminyltransferase activity; C:integral component of membrane

EC:2.3.1.5; EC:2.3.1.48
nding; F:translation initiation factor activity;

ctivator activity; C:integral component of membrane;
ır cluster assembly; F:transferase activity, transferring acyl groups;
EC:1.11.1.7
EC:1.8.1.9

omplex; P:protein transport; P:endosome transport via multivesicular body sorting pathway
le vesicle-mediated transport, Golgi to ER; C:integral component of membrane
le vesicle-mediated transport, Golgi to ER; C:integral component of membrane
noter sequence-specific DNA binding;
: activity
EC:1.1.1.210
abolic process; F:transferase activity;
abolic process; F:transferase activity;
ing; F:methyltransferase activity; P:methylation
omponent of membrane; F:transmembrane transporter activity;

:idase inhibitor activity; C:mitochondrion;
omponent of membrane
ing
EC:6.2.1.8
EC:3.4.23

EC:2.7.7.48
ing; C:cytoplasm

ı transport; F:metal ion binding
: activity

omponent of membrane
omponent of membrane
:ctron transfer activity; F:protein disulfide oxidoreductase activity;
P:regulation of transcription, DNA-templated;
EC:2.7.8.8
EC:5.1.3.2
EC:4.2.1.109; EC:3.1.3.77; EC:3.1.3.41
EC:4.2.1.109; EC:3.1.3.77; EC:3.1.3.41
nplex
EC:6.2.1.3
in binding

ng; C:mitochondrion; C:ribosome; P:ATP synthesis coupled proton transport
EC:2.1.1.125
EC:3.6.1.3; EC:3.6.1.15
on transport; F:cation transmembrane transporter activity; C:membrane;
tem I; C:chloroplast thylakoid membrane; C:chloroplast envelope;
ım ion binding; F:electron transfer activity;

ng; C:photosystem I; P:photosynthesis; C:integral component of membrane
:integral component of membrane
-protein transferase activity; P:protein ubiquitination
EC:3.6.1.3; EC:3.6.3.6; EC:3.6.1.15
EC:2.7.7.19
ndrial inner membrane; P:mitochondrial transport;
EC:3.6.1.3; EC:3.6.1.15

sembly
