# Supplementary material for: Differential expression of genes in olive leaves and buds of ON- versus OFF-crop trees
Source: Sci Rep. 2020 Sep 25;10:15762. doi: 10.1038/s41598-020-72895-7 (PMC7519672; doi:10.1038/s41598-020-72895-7)
Supplement: Supplementary file 3 — Supplementary Table 3. [file 41598_2020_72895_MOESM3_ESM.pdf]

Alternate bearing in olive: Differential expression of genes in leaves and buds of Ol

Ebrahim Dastkar[1], Ali Soleimani[1*], Hossein Jafary[2], Juan de Dios Alche[3], Abbas Bahari[4], Mehrshad Zein

Supplementary table S3. Results of KEGG pathway enrichment analysis of olive's

##Databases: KEGG PATHWAY

##Statistical test method: hypergeometric test / Fisher's exact test

##FDR correction method: Benjamini and Hochberg

#Term	Databas	ID	Input nu	Backgrc	P-Value	Corrected	Input
Photosynthesis	KEGG l	cic0019	9	47	1.97E-07	1.58E-05	TRINITY_DN88199_c2
Metabolic pathways	KEGG l	cic0110	52	1810	3.18E-05	0.00127	TRINITY_DN86856_c0
Fatty acid degradation	KEGG l	cic0007	5	38	0.00053	0.01417	TRINITY_DN86918_c1
beta-Alanine metabolism	KEGG l	cic0041	4	44	0.0066	0.1069	TRINITY_DN86918_c1
RNA degradation	KEGG l	cic0301	6	101	0.00668	0.1069	TRINITY_DN85300_c1
Ubiquitin mediated proteolysis	KEGG l	cic0412	6	129	0.0194	0.25866	TRINITY_DN85879_c0
Fatty acid metabolism	KEGG l	cic0121	4	68	0.02612	0.28071	TRINITY_DN86918_c1
Oxidative phosphorylation	KEGG l	cic0019	5	104	0.02814	0.28071	TRINITY_DN87899_c2
Butanoate metabolism	KEGG l	cic0065	2	17	0.03482	0.28071	TRINITY_DN80150_c1
Ribosome biogenesis in eukaryo	KEGG l	cic0300	4	75	0.03509	0.28071	TRINITY_DN77909_c1
Valine, leucine and isoleucine d	KEGG l	cic0028	3	47	0.04303	0.30421	TRINITY_DN86918_c1
Phagosome	KEGG l	cic0414	4	82	0.04563	0.30421	TRINITY_DN86165_c1
Protein processing in endoplasm	KEGG l	cic0414	7	208	0.05231	0.32192	TRINITY_DN81726_c4
Galactose metabolism	KEGG l	cic0005	3	56	0.06454	0.36878	TRINITY_DN83157_c0
Lysine degradation	KEGG l	cic0031	2	27	0.07472	0.39852	TRINITY_DN80450_c2
Pantothenate and CoA biosynth	KEGG l	cic0077	2	30	0.08859	0.44293	TRINITY_DN85379_c0
Propanoate metabolism	KEGG l	cic0064	2	32	0.09822	0.44399	TRINITY_DN86918_c1
Ascorbate and aldarate metaboli	KEGG l	cic0005	2	36	0.11828	0.44399	TRINITY_DN86273_c4
Mismatch repair	KEGG l	cic0343	2	38	0.12867	0.44399	TRINITY_DN76209_c0
Starch and sucrose metabolism	KEGG l	cic0050	6	213	0.12887	0.44399	TRINITY_DN83651_c2
Monoterpenoid biosynthesis	KEGG l	cic0090	1	8	0.13191	0.44399	TRINITY_DN87235_c0
mRNA surveillance pathway	KEGG l	cic0301	4	123	0.13744	0.44399	TRINITY_DN87854_c1
Amino sugar and nucleotide sug	KEGG l	cic0052	4	125	0.14307	0.44399	TRINITY_DN80543_c1
Monobactam biosynthesis	KEGG l	cic0026	1	9	0.14546	0.44399	TRINITY_DN80586_c1
Spliceosome	KEGG l	cic0304	5	176	0.15368	0.44399	TRINITY_DN87128_c2

Alanine, aspartate and glutamat	KEGG l	cic0025	2	43	0.15546	0.44399	TRINITY_DN85516_c1
N-Glycan biosynthesis	KEGG l	cic0051	2	43	0.15546	0.44399	TRINITY_DN84974_c1
Riboflavin metabolism	KEGG l	cic0074	1	10	0.15879	0.44399	TRINITY_DN83887_c0
Fatty acid biosynthesis	KEGG l	cic0006	2	44	0.16095	0.44399	TRINITY_DN86869_c0
Lysine biosynthesis	KEGG l	cic0030	1	12	0.18483	0.48015	TRINITY_DN80450_c2
Endocytosis	KEGG l	cic0414	4	140	0.18801	0.48015	TRINITY_DN85567_c1
Arginine and proline metabolisr	KEGG l	cic0033	2	52	0.20586	0.48015	TRINITY_DN80450_c2
Peroxisome	KEGG l	cic0414	3	98	0.20908	0.48015	TRINITY_DN78486_c0
Cysteine and methionine metab	KEGG l	cic0027	3	98	0.20908	0.48015	TRINITY_DN86856_c0
AGE-RAGE signaling pathway	KEGG l	cic0493	1	14	0.21006	0.48015	TRINITY_DN82285_c1
Histidine metabolism	KEGG l	cic0034	1	16	0.23452	0.49492	TRINITY_DN80450_c2
Linoleic acid metabolism	KEGG l	cic0059	1	16	0.23452	0.49492	TRINITY_DN86197_c3
Fructose and mannose metaboli	KEGG l	cic0005	2	59	0.24611	0.49492	TRINITY_DN80543_c1
Selenocompound metabolism	KEGG l	cic0045	1	17	0.24647	0.49492	TRINITY_DN80586_c1
Glyoxylate and dicarboxylate m	KEGG l	cic0063	2	60	0.25189	0.49492	TRINITY_DN80150_c1
RNA transport	KEGG l	cic0301	4	160	0.25365	0.49492	TRINITY_DN82036_c1
Valine, leucine and isoleucine b	KEGG l	cic0029	1	20	0.28119	0.53561	TRINITY_DN85379_c0
Ribosome	KEGG l	cic0301	6	281	0.2916	0.54251	TRINITY_DN82900_c2
ABC transporters	KEGG l	cic0201	1	22	0.30345	0.54399	TRINITY_DN79223_c1
Pyruvate metabolism	KEGG l	cic0062	2	70	0.30967	0.54399	TRINITY_DN80450_c2
Other glycan degradation	KEGG l	cic0051	1	23	0.31433	0.54399	TRINITY_DN87668_c0
Phenylpropanoid biosynthesis	KEGG l	cic0094	4	179	0.31959	0.54399	TRINITY_DN84914_c2
Biosynthesis of unsaturated fatt	KEGG l	cic0104	1	28	0.36619	0.59787	TRINITY_DN86524_c1
Arginine biosynthesis	KEGG l	cic0022	1	28	0.36619	0.59787	TRINITY_DN78356_c0
Nitrogen metabolism	KEGG l	cic0091	1	31	0.39542	0.61757	TRINITY_DN78356_c0
Fatty acid elongation	KEGG l	cic0006	1	32	0.40486	0.61757	TRINITY_DN86524_c1
RNA polymerase	KEGG l	cic0302	1	33	0.41415	0.61757	TRINITY_DN87049_c0
Sulfur metabolism	KEGG l	cic0092	1	34	0.4233	0.61757	TRINITY_DN80586_c1
Tryptophan metabolism	KEGG l	cic0038	1	34	0.4233	0.61757	TRINITY_DN80450_c2
Biosynthesis of secondary metal	KEGG l	cic0111	18	1069	0.43093	0.61757	TRINITY_DN83651_c2
Steroid biosynthesis	KEGG l	cic0010	1	35	0.4323	0.61757	TRINITY_DN83788_c0
alpha-Linolenic acid metabolisn	KEGG l	cic0059	1	39	0.46694	0.65535	TRINITY_DN86197_c3
Pyrimidine metabolism	KEGG l	cic0024	2	102	0.48401	0.6645	TRINITY_DN80144_c1

Porphyrin and chlorophyll meta	KEGG l	cic0086	1	42	0.49153	0.6645	TRINITY_DN82948_c1
Circadian rhythm - plant	KEGG l	cic0471	1	43	0.49947	0.6645	TRINITY_DN87924_c1
Carbon metabolism	KEGG l	cic0120	4	233	0.50673	0.6645	TRINITY_DN86918_c1
Basal transcription factors	KEGG l	cic0302	1	45	0.51499	0.6645	TRINITY_DN76711_c0
Protein export	KEGG l	cic0306	1	48	0.53736	0.6717	TRINITY_DN80397_c0
Phosphatidylinositol signaling s	KEGG l	cic0407	1	48	0.53736	0.6717	TRINITY_DN87622_c2
Phenylalanine metabolism	KEGG l	cic0036	1	52	0.56561	0.67535	TRINITY_DN84914_c2
Cyanoamino acid metabolism	KEGG l	cic0046	1	52	0.56561	0.67535	TRINITY_DN86488_c0
Aminoacyl-tRNA biosynthesis	KEGG l	cic0097	1	52	0.56561	0.67535	TRINITY_DN84141_c0
Terpenoid backbone biosynthes	KEGG l	cic0090	1	56	0.59213	0.68276	TRINITY_DN87499_c0
Glycerolipid metabolism	KEGG l	cic0056	1	58	0.60478	0.68276	TRINITY_DN80450_c2
2-Oxocarboxylic acid metabolis	KEGG l	cic0121	1	58	0.60478	0.68276	TRINITY_DN85379_c0
Carbon fixation in photosynthet	KEGG l	cic0071	1	60	0.61703	0.68276	TRINITY_DN85435_c0
Nucleotide excision repair	KEGG l	cic0342	1	61	0.62302	0.68276	TRINITY_DN86147_c0
Ubiquinone and other terpenoid	KEGG l	cic0013	1	61	0.62302	0.68276	TRINITY_DN84914_c2
Glycine, serine and threonine m	KEGG l	cic0026	1	64	0.64042	0.69141	TRINITY_DN80450_c2
Purine metabolism	KEGG l	cic0023	2	139	0.6482	0.69141	TRINITY_DN87049_c0
Biosynthesis of amino acids	KEGG l	cic0123	3	216	0.66642	0.70149	TRINITY_DN85379_c0
Glycerophospholipid metabolisi	KEGG l	cic0056	1	74	0.69285	0.71985	TRINITY_DN84682_c1
Pentose and glucuronate interco	KEGG l	cic0004	1	88	0.75369	0.77301	TRINITY_DN82242_c0
Glycolysis / Gluconeogenesis	KEGG l	cic0001	1	106	0.81458	0.82489	TRINITY_DN80450_c2
Plant hormone signal transducti	KEGG l	cic0407	1	255	0.98247	0.98247	TRINITY_DN84587_c1

#Term	Databas	ID	Input nu	Backgrc	P-Value	Corrected	Input

#Term	Databas	ID	Input nu	Backgrc	P-Value	Corrected	Input

N- *versus* OFF-crop trees

alabedini[illegible] and Seyed Alireza Salami [illegible]

leaf samples ON- vs. OFF-trees

Hyperlink

http://www.genome.jp/kegg-bin/show_pathway?cic00195/cic:CICLE_v10003393mg%09red/cic:CICLE_v1

http://www.genome.jp/kegg-bin/show_pathway?cic01100/cic:CICLE_v10025937mg%09red/cic:CICLE_v1

http://www.genome.jp/kegg-bin/show_pathway?cic00071/cic:CICLE_v10019200mg%09red/cic:CICLE_v1

http://www.genome.jp/kegg-bin/show_pathway?cic00410/cic:CICLE_v100285991m%09red/cic:CICLE_v1

http://www.genome.jp/kegg-bin/show_pathway?cic03018/cic:CICLE_v10000087mg%09red/cic:CICLE_v1

http://www.genome.jp/kegg-bin/show_pathway?cic04120/cic:CICLE_v10022463mg%09red/cic:CICLE_v1

http://www.genome.jp/kegg-bin/show_pathway?cic01212/cic:CICLE_v10019200mg%09red/cic:CICLE_v1

http://www.genome.jp/kegg-bin/show_pathway?cic00190/cic:CICLE_v10028070mg%09red/cic:CICLE_v1

http://www.genome.jp/kegg-bin/show_pathway?cic00650/cic:CICLE_v10025937mg%09red/cic:CICLE_v1

http://www.genome.jp/kegg-bin/show_pathway?cic03008/cic:CICLE_v10009426mg%09red/cic:CICLE_v1

http://www.genome.jp/kegg-bin/show_pathway?cic00280/cic:CICLE_v10025411mg%09red/cic:CICLE_v1

http://www.genome.jp/kegg-bin/show_pathway?cic04145/cic:CICLE_v10011848mg%09red/cic:CICLE_v1

http://www.genome.jp/kegg-bin/show_pathway?cic04141/cic:CICLE_v10011848mg%09red/cic:CICLE_v1

http://www.genome.jp/kegg-bin/show_pathway?cic00052/cic:CICLE_v10020919mg%09red/cic:CICLE_v1

http://www.genome.jp/kegg-bin/show_pathway?cic00310/cic:CICLE_v10025411mg%09red/cic:CICLE_v1

http://www.genome.jp/kegg-bin/show_pathway?cic00770/cic:CICLE_v100285991m%09red/cic:CICLE_v1

http://www.genome.jp/kegg-bin/show_pathway?cic00640/cic:CICLE_v10004291mg%09red

http://www.genome.jp/kegg-bin/show_pathway?cic00053/cic:CICLE_v10025411mg%09red/cic:CICLE_v1

http://www.genome.jp/kegg-bin/show_pathway?cic03430/cic:CICLE_v10018525mg%09red/cic:CICLE_v1

http://www.genome.jp/kegg-bin/show_pathway?cic00500/cic:CICLE_v10007964mg%09red/cic:CICLE_v1

http://www.genome.jp/kegg-bin/show_pathway?cic00902/cic:CICLE_v10014707mg%09red

http://www.genome.jp/kegg-bin/show_pathway?cic03015/cic:CICLE_v10000537mg%09red/cic:CICLE_v1

http://www.genome.jp/kegg-bin/show_pathway?cic00520/cic:CICLE_v10028621mg%09red/cic:CICLE_v1

http://www.genome.jp/kegg-bin/show_pathway?cic00261/cic:CICLE_v10001007mg%09red

http://www.genome.jp/kegg-bin/show_pathway?cic03040/cic:CICLE_v10004187mg%09red/cic:CICLE_v1

http://www.genome.jp/kegg-bin/show_pathway?cic00250/cic:CICLE_v10011785mg%09red/cic:CICLE_v1

http://www.genome.jp/kegg-bin/show_pathway?cic00510/cic:CICLE_v10028484mg%09red/cic:CICLE_v1

http://www.genome.jp/kegg-bin/show_pathway?cic00740/cic:CICLE_v10011346mg%09red

http://www.genome.jp/kegg-bin/show_pathway?cic00061/cic:CICLE_v10019200mg%09red/cic:CICLE_v1

http://www.genome.jp/kegg-bin/show_pathway?cic00300/cic:CICLE_v10025411mg%09red

http://www.genome.jp/kegg-bin/show_pathway?cic04144/cic:CICLE_v10009355mg%09red/cic:CICLE_v1

http://www.genome.jp/kegg-bin/show_pathway?cic00330/cic:CICLE_v10013866mg%09red/cic:CICLE_v1

http://www.genome.jp/kegg-bin/show_pathway?cic04146/cic:CICLE_v10032406mg%09red/cic:CICLE_v1

http://www.genome.jp/kegg-bin/show_pathway?cic00270/cic:CICLE_v10013866mg%09red/cic:CICLE_v1

http://www.genome.jp/kegg-bin/show_pathway?cic04933/cic:CICLE_v10028684mg%09red

http://www.genome.jp/kegg-bin/show_pathway?cic00340/cic:CICLE_v10025411mg%09red

http://www.genome.jp/kegg-bin/show_pathway?cic00591/cic:CICLE_v10014202mg%09red

http://www.genome.jp/kegg-bin/show_pathway?cic00051/cic:CICLE_v10015394mg%09red/cic:CICLE_v1

http://www.genome.jp/kegg-bin/show_pathway?cic00450/cic:CICLE_v10001007mg%09red

http://www.genome.jp/kegg-bin/show_pathway?cic00630/cic:CICLE_v10025937mg%09red/cic:CICLE_v1

http://www.genome.jp/kegg-bin/show_pathway?cic03013/cic:CICLE_v10000537mg%09red/cic:CICLE_v1

http://www.genome.jp/kegg-bin/show_pathway?cic00290/cic:CICLE_v10004615mg%09red

http://www.genome.jp/kegg-bin/show_pathway?cic03010/cic:CICLE_v10012499mg%09red/cic:CICLE_v1

http://www.genome.jp/kegg-bin/show_pathway?cic02010/cic:CICLE_v10004128mg%09red

http://www.genome.jp/kegg-bin/show_pathway?cic00620/cic:CICLE_v10024268mg%09red/cic:CICLE_v1

http://www.genome.jp/kegg-bin/show_pathway?cic00511/cic:CICLE_v10024800mg%09red

http://www.genome.jp/kegg-bin/show_pathway?cic00940/cic:CICLE_v10020880mg%09red/cic:CICLE_v1

http://www.genome.jp/kegg-bin/show_pathway?cic01040/cic:CICLE_v10020242mg%09red

http://www.genome.jp/kegg-bin/show_pathway?cic00220/cic:CICLE_v10011785mg%09red

http://www.genome.jp/kegg-bin/show_pathway?cic00910/cic:CICLE_v10011785mg%09red

http://www.genome.jp/kegg-bin/show_pathway?cic00062/cic:CICLE_v10020242mg%09red

http://www.genome.jp/kegg-bin/show_pathway?cic03020/cic:CICLE_v10025048mg%09red

http://www.genome.jp/kegg-bin/show_pathway?cic00920/cic:CICLE_v10001007mg%09red

http://www.genome.jp/kegg-bin/show_pathway?cic00380/cic:CICLE_v10025411mg%09red

http://www.genome.jp/kegg-bin/show_pathway?cic01110/cic:CICLE_v10020880mg%09red/cic:CICLE_v1

http://www.genome.jp/kegg-bin/show_pathway?cic00100/cic:CICLE_v10030984mg%09red

http://www.genome.jp/kegg-bin/show_pathway?cic00592/cic:CICLE_v10014202mg%09red

http://www.genome.jp/kegg-bin/show_pathway?cic00240/cic:CICLE_v100285991m%09red/cic:CICLE_v1

http://www.genome.jp/kegg-bin/show_pathway?cic00860/cic:CICLE_v10000765mg%09red

http://www.genome.jp/kegg-bin/show_pathway?cic04712/cic:CICLE_v10004044mg%09red

http://www.genome.jp/kegg-bin/show_pathway?cic01200/cic:CICLE_v10025937mg%09red/cic:CICLE_v1[illegible]

http://www.genome.jp/kegg-bin/show_pathway?cic03022/cic:CICLE_v10028561mg%09red

http://www.genome.jp/kegg-bin/show_pathway?cic03060/cic:CICLE_v100309551m%09red

http://www.genome.jp/kegg-bin/show_pathway?cic04070/cic:CICLE_v100109631m%09red

http://www.genome.jp/kegg-bin/show_pathway?cic00360/cic:CICLE_v10028138mg%09red

http://www.genome.jp/kegg-bin/show_pathway?cic00460/cic:CICLE_v10031212mg%09red

http://www.genome.jp/kegg-bin/show_pathway?cic00970/cic:CICLE_v10018820mg%09red

http://www.genome.jp/kegg-bin/show_pathway?cic00900/cic:CICLE_v10028082mg%09red

http://www.genome.jp/kegg-bin/show_pathway?cic00561/cic:CICLE_v10025411mg%09red

http://www.genome.jp/kegg-bin/show_pathway?cic01210/cic:CICLE_v10004615mg%09red

http://www.genome.jp/kegg-bin/show_pathway?cic00710/cic:CICLE_v10024268mg%09red

http://www.genome.jp/kegg-bin/show_pathway?cic03420/cic:CICLE_v10011886mg%09red

http://www.genome.jp/kegg-bin/show_pathway?cic00130/cic:CICLE_v10028138mg%09red

http://www.genome.jp/kegg-bin/show_pathway?cic00260/cic:CICLE_v10025411mg%09red

http://www.genome.jp/kegg-bin/show_pathway?cic00230/cic:CICLE_v10001007mg%09red/cic:CICLE_v1[illegible]

http://www.genome.jp/kegg-bin/show_pathway?cic01230/cic:CICLE_v10011785mg%09red/cic:CICLE_v1[illegible]

http://www.genome.jp/kegg-bin/show_pathway?cic00564/cic:CICLE_v10015341mg%09red

http://www.genome.jp/kegg-bin/show_pathway?cic00040/cic:CICLE_v10007964mg%09red

http://www.genome.jp/kegg-bin/show_pathway?cic00010/cic:CICLE_v10025411mg%09red

http://www.genome.jp/kegg-bin/show_pathway?cic04075/cic:CICLE_v10007908mg%09red

Hyperlink

Hyperlink

0016927mg%09red/cic:CICLE_v10003146mg%09red/cic:CICLE_v10030467mg%09red/cic:CICLE_v1002

0028070mg%09red/cic:CICLE_v10007808mg%09red/cic:CICLE_v10030984mg%09red/cic:CICLE_v1000

0019205mg%09red/cic:CICLE_v10025411mg%09red/cic:CICLE_v10004291mg%09red

0025411mg%09red/cic:CICLE_v10004291mg%09red

0027731mg%09red/cic:CICLE_v10025257mg%09red/cic:CICLE_v10011329mg%09red/cic:CICLE_v1000

0007821mg%09red/cic:CICLE_v10012469mg%09red/cic:CICLE_v10007219mg%09red/cic:CICLE_v1001

0019205mg%09red/cic:CICLE_v10004291mg%09red

0030453mg%09red/cic:CICLE_v10015638mg%09red/cic:CICLE_v100248071m%09red/cic:CICLE_v1000

0004713mg%09red

00041222m%09red/cic:CICLE_v10005540mg%09red/cic:CICLE_v10000087mg%09red

0004291mg%09red

0028070mg%09red/cic:CICLE_v10015638mg%09red/cic:CICLE_v10009240mg%09red

0025230mg%09red/cic:CICLE_v10011886mg%09red/cic:CICLE_v10028484mg%09red/cic:CICLE_v1002

0007519mg%09red

00233292m%09red

0004615mg%09red

0015090mg%09red

0004302mg%09red

0031212mg%09red/cic:CICLE_v10027815mg%09red/cic:CICLE_v100013481m%09red/cic:CICLE_v1002

0031706mg%09red/cic:CICLE_v10031047mg%09red/cic:CICLE_v10025039mg%09red

0020919mg%09red/cic:CICLE_v10033733mg%09red/cic:CICLE_v10032349mg%09red

0025102mg%09red/cic:CICLE_v10031706mg%09red/cic:CICLE_v10025502mg%09red

0004713mg%09red
0025230mg%09red

0019205mg%09red

0012725mg%09red/cic:CICLE_v10008023mg%09red/cic:CICLE_v10025102mg%09red
0025411mg%09red
0019200mg%09red/cic:CICLE_v10019205mg%09red
0007808mg%09red

0033733mg%09red

0011785mg%09red
0031706mg%09red/cic:CICLE_v100110482m%09red/cic:CICLE_v10009426mg%09red

0013231mg%09red/cic:CICLE_v10033755mg%09red/cic:CICLE_v10013022mg%09red/cic:CICLE_v1002

0025411mg%09red

0031212mg%09red/cic:CICLE_v10028138mg%09red/cic:CICLE_v10015870mg%09red

0015870mg%09red/cic:CICLE_v10028082mg%09red/cic:CICLE_v10030984mg%09red/cic:CICLE_v1003

0025048mg%09red

0024268mg%09red/cic:CICLE_v10004291mg%09red

0025048mg%09red

0025411mg%09red/cic:CICLE_v10004615mg%09red

3978mg%09red

4615mg%09red/cic:CICLE_v10025230mg%09red/cic:CICLE_v10028138mg%09red/cic:CICLE_v1001509

0537mg%09red/cic:CICLE_v10007274mg%09red

09402m%09red/cic:CICLE_v10000001mg%09red

9240mg%09red

5102mg%09red/cic:CICLE_v10027715mg%09red/cic:CICLE_v10025307mg%09red

0410mg%09red

2860mg%09red/cic:CICLE_v10009930mg%09red

3733mg%09red/cic:CICLE_v10004615mg%09red/cic:CICLE_v10028138mg%09red/cic:CICLE_v1002781[illegible]

0mg%09red/cic:CICLE_v10028484mg%09red/cic:CICLE_v10025411mg%09red/cic:CICLE_v10003393mg

5mg%09red/cic:CICLE_v10004291mg%09red/cic:CICLE_v10014707mg%09red/cic:CICLE_v10020242mg

;%09red/cic:CICLE_v10031212mg%09red/cic:CICLE_v10011785mg%09red/cic:CICLE_v10016927mg%0

;%09red/cic:CICLE_v10025411mg%09red/cic:CICLE_v10000765mg%09red/cic:CICLE_v10014202mg%0

9red/cic:CICLE_v10020410mg%09red/cic:CICLE_v10033733mg%09red/cic:CICLE_v10028082mg%09rec

9red/cic:CICLE_v10031212mg%09red/cic:CICLE_v10015341mg%09red/cic:CICLE_v10011346mg%09rec

d/cic:CICLE_v10028621mg%09red/cic:CICLE_v10001007mg%09red/cic:CICLE_v10027815mg%09red/cic

d

:CICLE_v10020919mg%09red/cic:CICLE_v10003146mg%09red/cic:CICLE_v10013866mg%09red/cic:CI

CLE_v100013481m%09red/cic:CICLE_v10020880mg%09red/cic:CICLE_v10003639mg%09red/cic:CICLE

E_v10023978mg%09red/cic:CICLE_v10007964mg%09red/cic:CICLE_v10011346mg%09red/cic:CICLE_v1

10019205mg%09red/cic:CICLE_v10019200mg%09red/cic:CICLE_v10020242mg%09red/cic:CICLE_v100[illegible]

14202mg%09red/cic:CICLE_v10004713mg%09red/cic:CICLE_v10030453m
