# Supplementary material for: Differential expression of genes in olive leaves and buds of ON- versus OFF-crop trees
Source: Sci Rep. 2020 Sep 25;10:15762. doi: 10.1038/s41598-020-72895-7 (PMC7519672; doi:10.1038/s41598-020-72895-7)
Supplement: Supplementary file 4 — Supplementary Table 4. [file 41598_2020_72895_MOESM4_ESM.pdf]

Alternate bearing in olive: Differential expression of genes in leaves and buds of ON- *versus* OFF-c

Ebrahim Dastkar[1], Ali Soleimani[1*], Hossein Jafary[2], Juan de Dios Alche[3], Abbas Bahari[4], Mehrshad Zeinalabedini[5] and Seyed

Supplementary table S4. Results of differential expression (DE) analysis of olive's bud sampl

SeqName	baseMean	log2FoldChange	lfcSE	stat	pvalue
TRINITY_DN87407_c2_g2_i1	1623.442376	3.769914984	0.404148028	9.328054877	1.07832E-20
TRINITY_DN85536_c0_g1_i9	62.94110718	21.65241246	2.497796184	8.668606589	4.37441E-18
TRINITY_DN79832_c2_g1_i1	291.4688904	8.967158362	1.099196539	8.15792085	3.40841E-16
TRINITY_DN78161_c0_g2_i1	369.0700046	-3.452730117	0.448954632	-7.690599165	1.46448E-14
TRINITY_DN81947_c0_g5_i2	145.690232	4.066686369	0.533309575	7.625376627	2.43323E-14
TRINITY_DN87593_c3_g1_i3	244.9644876	-7.16133914	0.962695541	-7.438841085	1.01572E-13
TRINITY_DN81974_c3_g1_i4	222.7367704	3.769791186	0.519476482	7.256904432	3.96049E-13
TRINITY_DN84932_c0_g2_i1	522.8785721	3.5193588	0.492077098	7.152047539	8.54929E-13
TRINITY_DN79223_c1_g2_i1	162.4883839	-23.59064505	3.343124964	-7.056465225	1.70791E-12
TRINITY_DN82872_c3_g1_i2	153.9943925	-10.86416653	1.544528358	-7.033970254	2.00738E-12
TRINITY_DN88686_c6_g4_i2	3387.204102	3.033149938	0.434477236	6.981148117	2.92777E-12
TRINITY_DN84622_c0_g1_i3	686.9597446	4.368875127	0.631077425	6.922882921	4.42544E-12
TRINITY_DN88686_c6_g1_i6	18860.08592	3.16394377	0.463306816	6.829046453	8.54809E-12
TRINITY_DN87112_c1_g1_i1	2672.463427	2.232402619	0.331839455	6.727357425	1.72772E-11
TRINITY_DN79281_c0_g3_i1	115.9797403	-10.30598278	1.541031086	-6.68771894	2.26676E-11
TRINITY_DN84317_c2_g1_i1	275.4242611	10.23304234	1.54819966	6.609639964	3.85256E-11
TRINITY_DN81834_c0_g7_i1	106.5505666	4.137233073	0.626112018	6.607816096	3.90031E-11
TRINITY_DN78161_c0_g2_i7	124.2361012	11.48778915	1.74363341	6.588419954	4.44532E-11
TRINITY_DN85217_c1_g1_i6	99.63330375	-11.14777847	1.725923122	-6.459023768	1.05381E-10
TRINITY_DN83153_c0_g1_i7	137.8248861	10.45392446	1.62954034	6.415259691	1.40583E-10
TRINITY_DN86488_c0_g1_i1	149.5682843	-10.44238235	1.627471398	-6.416323113	1.39605E-10
TRINITY_DN81213_c3_g1_i1	874.4422273	2.238521392	0.349773132	6.399923793	1.55455E-10
TRINITY_DN87158_c0_g1_i1	779.8882879	3.65372169	0.571630531	6.391753929	1.63994E-10
TRINITY_DN81443_c2_g2_i1	241.7016029	3.109099528	0.487699472	6.375031569	1.82925E-10
TRINITY_DN79257_c0_g3_i3	90.62331279	-10.41630382	1.638952417	-6.35546445	2.07798E-10
TRINITY_DN78450_c1_g5_i2	955.1768385	3.03568622	0.477078046	6.363080939	1.97746E-10
TRINITY_DN87907_c1_g1_i3	207.1439946	12.20197968	1.920394176	6.353893295	2.09932E-10
TRINITY_DN86418_c1_g1_i9	481.3516017	9.740274609	1.535411685	6.343754387	2.24232E-10
TRINITY_DN82197_c2_g1_i3	119.240803	-10.18361549	1.634439105	-6.230648461	4.64508E-10
TRINITY_DN88686_c6_g2_i2	20855.42188	2.956287932	0.476643778	6.202300473	5.56437E-10
TRINITY_DN85449_c2_g1_i1	392.5446373	11.46168122	1.847617918	6.203491053	5.52242E-10
TRINITY_DN87790_c2_g1_i7	1739.903218	5.36240951	0.869402569	6.167924622	6.91921E-10
TRINITY_DN81755_c0_g3_i2	174.0090786	9.571874655	1.555925496	6.151884957	7.65674E-10
TRINITY_DN86430_c0_g3_i2	1007.304489	2.960757438	0.483025577	6.129607995	8.80959E-10
TRINITY_DN79244_c2_g7_i1	83.69749559	10.53522015	1.721743699	6.118924759	9.42089E-10
TRINITY_DN86856_c0_g1_i2	344.653168	10.01821536	1.640103106	6.108283878	1.00708E-09
TRINITY_DN85155_c0_g1_i6	461.6438595	10.68066885	1.752276893	6.095308848	1.09226E-09
TRINITY_DN78706_c0_g4_i3	160.5227146	2.986288835	0.491982607	6.06990734	1.27984E-09
TRINITY_DN81560_c0_g1_i1	71.77800976	10.24419519	1.695704036	6.041263672	1.52912E-09
TRINITY_DN73004_c0_g1_i1	339.3272832	2.937927838	0.486099091	6.043886714	1.50445E-09
TRINITY_DN88009_c0_g1_i8	147.9477173	-6.861201477	1.150755349	-5.962345935	2.48642E-09
TRINITY_DN81443_c2_g1_i1	399.5660308	2.726435362	0.457819115	5.955267641	2.59647E-09
TRINITY_DN86910_c0_g2_i2	102.5895443	5.785567058	0.972102483	5.951601975	2.6553E-09
TRINITY_DN84738_c1_g1_i5	309.3478154	9.136973101	1.541540718	5.927169483	3.08201E-09
TRINITY_DN86198_c0_g1_i2	190.6732371	-9.676538358	1.634172843	-5.921367743	3.19275E-09
TRINITY_DN84317_c2_g1_i9	120.6363141	9.107331309	1.558807748	5.842498101	5.14238E-09
TRINITY_DN85145_c0_g1_i5	70.21356361	-9.576101662	1.64109898	-5.83517617	5.37338E-09
TRINITY_DN78590_c0_g1_i7	190.8244745	3.623106427	0.623865048	5.80751629	6.34064E-09
TRINITY_DN84464_c3_g1_i3	49.06145917	9.267461872	1.598285527	5.798376896	6.69598E-09
TRINITY_DN79400_c3_g1_i2	281.0075986	3.877818591	0.671047288	5.77875607	7.52549E-09
TRINITY_DN86251_c2_g1_i1	6274.254461	2.305507972	0.400738387	5.753149801	8.75958E-09
TRINITY_DN78907_c1_g2_i3	351.6353358	2.79839722	0.48732388	5.742376544	9.33569E-09

TRINITY_DN79965_c0_g2_i1	67.73282347	9.519564245	1.663649088	5.722098678	1.05216E-08
TRINITY_DN78079_c0_g1_i9	189.1159359	8.750582704	1.53338783	5.706698942	1.15188E-08
TRINITY_DN85978_c0_g1_i2	204.5469577	8.80824319	1.543027962	5.708414499	1.14033E-08
TRINITY_DN88750_c4_g2_i1	111.0166139	-6.704634821	1.174558113	-5.708218903	1.14165E-08
TRINITY_DN85119_c0_g1_i2	90.18414766	9.239233395	1.624924466	5.685946386	1.3009E-08
TRINITY_DN81196_c0_g1_i1	191.5741043	9.145212563	1.621221366	5.640940068	1.69124E-08
TRINITY_DN79794_c0_g2_i1	414.9156103	-8.679809506	1.540164606	-5.635637563	1.74412E-08
TRINITY_DN83000_c1_g1_i8	206.2351465	8.970589891	1.593489469	5.629525681	1.80706E-08
TRINITY_DN87627_c3_g1_i5	82.0177335	-9.007465864	1.60160574	-5.624021968	1.86562E-08
TRINITY_DN81834_c0_g4_i6	2250.171177	2.665310604	0.473831357	5.625019458	1.85487E-08
TRINITY_DN79326_c0_g1_i2	76.59626897	4.210074469	0.750528815	5.609477456	2.02938E-08
TRINITY_DN87464_c1_g1_i1	39.15126453	-7.046755663	1.256253898	-5.609340337	2.03099E-08
TRINITY_DN78140_c4_g1_i1	73.01753597	10.48754095	1.872446048	5.600984317	2.13138E-08
TRINITY_DN77988_c0_g5_i1	416.794525	3.62140735	0.646742693	5.59945615	2.15025E-08
TRINITY_DN86462_c1_g2_i2	204.6306624	-9.456763801	1.694363505	-5.581307535	2.38717E-08
TRINITY_DN86356_c0_g1_i9	53.40775436	-9.314610196	1.67014098	-5.577140078	2.44505E-08
TRINITY_DN87796_c0_g1_i2	357.659904	8.467606414	1.522589137	5.561320653	2.67741E-08
TRINITY_DN80669_c1_g1_i8	153.1242859	3.120090757	0.562640812	5.545439806	2.93216E-08
TRINITY_DN85288_c1_g5_i1	1823.112377	1.980322551	0.357602037	5.537783198	3.06324E-08
TRINITY_DN77816_c0_g5_i3	78.59943977	-9.914787996	1.790194498	-5.538385918	3.05272E-08
TRINITY_DN77988_c0_g1_i1	767.6196091	4.198801068	0.759096976	5.531310491	3.17847E-08
TRINITY_DN82364_c0_g1_i1	64.46532749	-8.910924469	1.616889099	-5.51115378	3.56489E-08
TRINITY_DN83938_c0_g1_i1	66.7945838	-9.269421475	1.681607469	-5.512238526	3.54298E-08
TRINITY_DN87261_c0_g1_i1	48.78636206	-8.795700853	1.59480565	-5.515218016	3.48348E-08
TRINITY_DN86430_c0_g4_i1	780.9745751	2.944549969	0.536413749	5.489326058	4.0347E-08
TRINITY_DN86166_c1_g3_i3	1340.035389	1.946724663	0.354859745	5.485898839	4.11372E-08
TRINITY_DN84771_c2_g6_i1	53.94817113	10.38034772	1.894322885	5.479714048	4.26014E-08
TRINITY_DN85947_c2_g1_i7	54.42249729	-9.141561397	1.672926513	-5.464413006	4.64441E-08
TRINITY_DN87144_c2_g2_i2	63.83980775	-9.486506345	1.737601162	-5.459541897	4.77365E-08
TRINITY_DN87790_c2_g1_i5	90.44024795	5.60153072	1.027882152	5.449584574	5.04876E-08
TRINITY_DN85424_c1_g1_i2	58.82128583	-8.753584699	1.608054496	-5.443587092	5.22182E-08
TRINITY_DN81694_c0_g1_i3	99.9094644	3.883640095	0.713739904	5.441253984	5.29068E-08
TRINITY_DN88648_c2_g1_i1	26688.51375	2.750284735	0.507224675	5.422221892	5.88628E-08
TRINITY_DN87613_c4_g1_i2	497.4918765	2.385069803	0.440021055	5.42035381	5.94812E-08
TRINITY_DN86051_c2_g2_i2	89.96338737	8.81857869	1.627850135	5.417316067	6.05003E-08
TRINITY_DN88319_c4_g1_i1	1191.613348	2.552463442	0.472194907	5.405529378	6.46173E-08
TRINITY_DN85288_c1_g3_i9	7155.838033	2.140219117	0.396507375	5.397677952	6.75089E-08
TRINITY_DN78090_c4_g1_i1	94.31975218	-9.04845656	1.683509774	-5.374757368	7.66859E-08
TRINITY_DN84165_c4_g4_i8	74.84052997	-9.498475856	1.768773682	-5.370091127	7.86969E-08
TRINITY_DN82516_c0_g2_i5	39.84463951	-9.347976678	1.74123833	-5.368579658	7.93591E-08
TRINITY_DN78849_c2_g3_i3	2502.731547	2.181771101	0.407571681	5.353097877	8.64611E-08
TRINITY_DN80860_c0_g1_i4	40.14298614	8.891486791	1.661135731	5.352655191	8.66729E-08
TRINITY_DN86367_c1_g1_i6	82.51581784	-8.826294665	1.650741287	-5.346867333	8.94896E-08
TRINITY_DN86518_c0_g1_i1	185.7299422	8.827850347	1.654022258	5.337201664	9.4392E-08
TRINITY_DN79694_c1_g5_i7	115.7181774	3.504767236	0.658355832	5.323515137	1.01781E-07
TRINITY_DN79163_c2_g2_i2	134.5483174	8.368488254	1.574221004	5.31595515	1.06099E-07
TRINITY_DN79579_c0_g1_i6	91.97924008	5.521638615	1.039614399	5.311237146	1.08884E-07
TRINITY_DN88821_c1_g1_i4	38.82471117	-8.696124473	1.644162102	-5.289091909	1.22925E-07
TRINITY_DN87095_c2_g2_i4	149.2104317	8.454048055	1.599502298	5.285424137	1.25414E-07
TRINITY_DN79725_c1_g3_i2	136.9812345	8.454220587	1.599355506	5.286017122	1.25008E-07
TRINITY_DN79400_c3_g11_i	219.5665364	3.04949688	0.578708432	5.269487558	1.36805E-07
TRINITY_DN83819_c0_g1_i5	143.3226305	8.513395877	1.61533375	5.270363403	1.36154E-07
TRINITY_DN85908_c0_g1_i1	49.32539269	9.544611112	1.816087957	5.255588571	1.47552E-07
TRINITY_DN81683_c2_g1_i1	703.387965	2.582049247	0.491179031	5.256839328	1.46552E-07
TRINITY_DN80704_c0_g1_i5	84.97469038	9.050850361	1.727928236	5.237978159	1.62345E-07
TRINITY_DN76217_c1_g1_i9	35.08340488	9.145125944	1.748341094	5.23074472	1.68829E-07
TRINITY_DN84298_c0_g1_i6	133.5223657	4.837305415	0.924979997	5.229632459	1.69847E-07

TRINITY_DN87692_c0_g1_i4	55.23235245	-8.3881796	1.604489024	-5.227944519	1.71405E-07
TRINITY_DN87158_c0_g2_i1	1575.854142	5.394147268	1.031624345	5.228790201	1.70623E-07
TRINITY_DN83039_c0_g2_i9	162.1378923	3.459385538	0.663185247	5.216318601	1.82514E-07
TRINITY_DN78705_c1_g4_i6	35.77082343	9.085219808	1.741560976	5.216710717	1.82128E-07
TRINITY_DN85411_c0_g3_i3	33.10700928	-8.834971992	1.695449853	-5.210989859	1.87836E-07
TRINITY_DN87790_c2_g5_i2	345.0146322	7.986279051	1.533396072	5.208229757	1.90651E-07
TRINITY_DN87901_c1_g1_i1	1576.583525	2.055176788	0.394803175	5.205573099	1.93399E-07
TRINITY_DN80853_c2_g2_i1	521.3963934	8.067311781	1.550784424	5.202084607	1.97065E-07
TRINITY_DN83817_c3_g2_i1	237.9219051	8.443337822	1.624650664	5.197017434	2.02511E-07
TRINITY_DN84705_c4_g1_i2	30.1923713	-8.853428078	1.707780236	-5.184172935	2.16976E-07
TRINITY_DN78578_c0_g1_i4	344.637521	8.666998861	1.673753573	5.178180947	2.2406E-07
TRINITY_DN78846_c0_g12_i	824.1012147	-3.153119151	0.609253854	-5.175378261	2.2745E-07
TRINITY_DN80448_c0_g1_i2	840.1872341	4.911732785	0.951057148	5.164498048	2.41085E-07
TRINITY_DN86779_c1_g2_i1	1266.054247	8.082881592	1.564986183	5.164826168	2.40662E-07
TRINITY_DN83365_c0_g2_i3	107.4407537	8.174335207	1.584777693	5.158032728	2.49558E-07
TRINITY_DN83700_c1_g1_i4	45.62074749	9.223928329	1.790505672	5.151577275	2.58305E-07
TRINITY_DN85681_c2_g1_i7	89.50316695	-8.426996717	1.638863275	-5.141976666	2.71863E-07
TRINITY_DN85125_c0_g2_i1	149.616704	3.47934229	0.677075418	5.138780992	2.76526E-07
TRINITY_DN83458_c0_g1_i1	46.22687573	-8.579565357	1.671007967	-5.13436532	2.83098E-07
TRINITY_DN80385_c0_g2_i4	361.7711768	8.5585896	1.667697113	5.13198082	2.86709E-07
TRINITY_DN85053_c0_g1_i7	29.41679369	8.593294402	1.67694353	5.124379114	2.9852E-07
TRINITY_DN85268_c3_g2_i1	1762.620021	2.618338361	0.511118287	5.122764	3.01089E-07
TRINITY_DN82158_c0_g2_i3	99.11336537	-3.68002062	0.718223779	-5.123779981	2.9947E-07
TRINITY_DN77944_c2_g3_i7	51.3972821	-4.409181541	0.863273736	-5.107512665	3.26427E-07
TRINITY_DN86903_c0_g2_i9	326.715178	4.530938111	0.887690195	5.10418853	3.32217E-07
TRINITY_DN87035_c0_g4_i6	26.06628352	-8.502163086	1.667293085	-5.099381245	3.40766E-07
TRINITY_DN71870_c0_g1_i1	93.90998632	-24.38629652	4.784956266	-5.096451288	3.4608E-07
TRINITY_DN86439_c3_g1_i7	35.75642738	8.442846044	1.656805103	5.095859513	3.47163E-07
TRINITY_DN81372_c1_g2_i1	633.1260042	-2.311828714	0.454088359	-5.091142877	3.55912E-07
TRINITY_DN84241_c1_g2_i2	52.04682287	8.467264524	1.663704092	5.089405362	3.59188E-07
TRINITY_DN76808_c0_g3_i2	45.35549681	-8.70541776	1.712026276	-5.084862238	3.67893E-07
TRINITY_DN82758_c0_g1_i1	40.64121769	8.466133126	1.668357055	5.074533118	3.88449E-07
TRINITY_DN86886_c0_g2_i1	59.68355107	8.162694033	1.608136765	5.07587054	3.85726E-07
TRINITY_DN79499_c2_g1_i2	55.00668036	8.260531244	1.628436922	5.072674988	3.92262E-07
TRINITY_DN82460_c3_g2_i1	45.34196543	10.12961897	2.000156059	5.064414312	4.09658E-07
TRINITY_DN83510_c0_g2_i1	72.66225301	8.002641251	1.581054048	5.061586137	4.15783E-07
TRINITY_DN83278_c1_g2_i1	153.8599006	6.267410446	1.2385725	5.060188602	4.18842E-07
TRINITY_DN78556_c1_g1_i7	172.689848	6.320697649	1.249493314	5.058608618	4.22327E-07
TRINITY_DN79304_c2_g1_i2	309.6717549	3.121254383	0.618567615	5.045938886	4.513E-07
TRINITY_DN84021_c1_g1_i1	60.1687173	8.844172636	1.754590878	5.040589658	4.641E-07
TRINITY_DN87053_c0_g1_i1	53.37001569	-8.107693421	1.608179032	-5.041536583	4.61809E-07
TRINITY_DN87940_c1_g5_i2	41.08241041	9.132010214	1.8141359	5.033807122	4.80833E-07
TRINITY_DN84933_c2_g4_i2	3926.070186	2.238462295	0.445556183	5.023973135	5.06133E-07
TRINITY_DN87766_c1_g7_i4	560.4062265	4.675974196	0.930870254	5.023228722	5.081E-07
TRINITY_DN87606_c1_g3_i1	100.981896	8.478730416	1.689624598	5.018114927	5.2181E-07
TRINITY_DN86152_c1_g1_i7	55.28725193	8.314357088	1.659270129	5.010852026	5.41896E-07
TRINITY_DN83458_c0_g1_i3	41.9305927	8.45428968	1.688256533	5.007704408	5.5083E-07
TRINITY_DN87613_c5_g1_i2	18577.34817	2.724214541	0.545167005	4.997027547	5.82208E-07
TRINITY_DN88309_c0_g1_i3	78.51265146	23.90527635	4.785001516	4.995876441	5.85692E-07
TRINITY_DN86462_c1_g2_i5	34.33200014	-8.814663359	1.763629387	-4.998024769	5.79205E-07
TRINITY_DN87158_c0_g2_i1	439.4994913	3.093034118	0.61886208	4.997937694	5.79467E-07
TRINITY_DN87492_c0_g2_i3	287.8490845	2.823151544	0.565409172	4.99311239	5.9414E-07
TRINITY_DN87272_c0_g1_i2	183.3211836	4.797472308	0.962344849	4.985190405	6.19008E-07
TRINITY_DN87555_c1_g1_i3	57.359754	-8.270122891	1.660223025	-4.981332487	6.31479E-07
TRINITY_DN85908_c0_g1_i2	41.88723132	9.028966256	1.81255575	4.981345406	6.31437E-07
TRINITY_DN80986_c1_g3_i1	20.72234046	8.548794516	1.716828097	4.979412052	6.37777E-07
TRINITY_DN84788_c0_g1_i2	101.2417845	8.286779997	1.66643042	4.972772879	6.6002E-07

TRINITY_DN81419_c1_g1_i8	2829.89124	2.044483124	0.411198444	4.972010844	6.6262E-07
TRINITY_DN85792_c1_g4_i3	45.35868168	9.091938199	1.82924096	4.970333814	6.68377E-07
TRINITY_DN86072_c1_g2_i8	130.6334513	8.245397654	1.660189759	4.966539282	6.81583E-07
TRINITY_DN70596_c0_g1_i2	90.1268069	5.5541337	1.118583794	4.965326449	6.85856E-07
TRINITY_DN78798_c1_g1_i5	41.45462767	8.204507352	1.653282203	4.962557109	6.95711E-07
TRINITY_DN86233_c5_g1_i9	1164.296586	7.514471638	1.51553294	4.958303076	7.11116E-07
TRINITY_DN82639_c0_g2_i5	158.1983682	-6.06259048	1.224457855	-4.951244713	7.37403E-07
TRINITY_DN78530_c1_g1_i2	48.85874741	-8.068495008	1.630088113	-4.949729371	7.43167E-07
TRINITY_DN86981_c0_g1_i1	1188.870607	2.403056234	0.486572885	4.938738491	7.86296E-07
TRINITY_DN83556_c2_g1_i7	657.3611296	2.05141447	0.415530158	4.936860616	7.93902E-07
TRINITY_DN81642_c1_g2_i3	66.33181349	8.004128571	1.622311786	4.933779461	8.06535E-07
TRINITY_DN88128_c0_g1_i3	910.2399712	3.253720137	0.659672505	4.932326439	8.1256E-07
TRINITY_DN79790_c1_g1_i5	496.424716	-8.177150483	1.659240363	-4.928249495	8.29696E-07
TRINITY_DN87950_c0_g1_i2	99.86769719	7.648020919	1.553164979	4.92415231	8.47268E-07
TRINITY_DN86363_c1_g2_i1	505.0846675	2.258362586	0.45906672	4.91946484	8.67812E-07
TRINITY_DN79824_c6_g1_i1	29.97326081	8.49150584	1.727103188	4.916617547	8.80523E-07
TRINITY_DN88736_c1_g2_i1	255.9232998	23.52068914	4.784884347	4.915623332	8.85004E-07
TRINITY_DN81973_c2_g1_i9	1858.087975	2.149762771	0.437700646	4.911490969	9.03865E-07
TRINITY_DN78547_c0_g1_i7	106.6376488	8.082453812	1.646930929	4.907585177	9.22046E-07
TRINITY_DN64854_c0_g1_i2	288.4549649	3.122686966	0.636236229	4.908062169	9.19807E-07
TRINITY_DN80757_c2_g2_i1	89.12630689	8.343260106	1.701279626	4.904108635	9.38525E-07
TRINITY_DN78772_c0_g4_i5	29.28365809	-9.278701756	1.893227524	-4.900996651	9.53517E-07
TRINITY_DN85041_c1_g1_i1	32.1662405	-9.384692665	1.918681827	-4.891218822	1.00213E-06
TRINITY_DN77393_c0_g3_i6	37.35602878	5.777461739	1.181314859	4.890704368	1.00476E-06
TRINITY_DN79455_c1_g2_i9	401.6682398	5.257370557	1.075008616	4.890538067	1.00561E-06
TRINITY_DN83989_c0_g4_i3	37.38955136	9.139109562	1.870375151	4.886244108	1.02778E-06
TRINITY_DN84690_c0_g1_i3	67.15842769	7.668645731	1.569909159	4.884770363	1.03549E-06
TRINITY_DN78458_c0_g3_i2	79.66688795	-23.35908957	4.785254912	-4.881472356	1.05297E-06
TRINITY_DN84863_c1_g3_i4	27.81398418	8.145632468	1.668816791	4.881082519	1.05505E-06
TRINITY_DN82042_c5_g1_i1	4411.477938	3.120472915	0.639535321	4.879281581	1.06473E-06
TRINITY_DN85899_c1_g1_i5	46.43265441	-23.34403931	4.785265732	-4.878316193	1.06995E-06
TRINITY_DN78671_c4_g1_i7	360.5494785	2.71340824	0.557388064	4.868077409	1.12689E-06
TRINITY_DN88726_c1_g1_i8	77.42061762	-9.366744065	1.924545911	-4.86698915	1.13311E-06
TRINITY_DN78116_c2_g1_i1	156.3676488	4.874306156	1.001739566	4.865841705	1.13971E-06
TRINITY_DN65695_c0_g1_i1	41.29185901	-23.27707907	4.785283715	-4.864304909	1.1486E-06
TRINITY_DN87018_c0_g1_i6	25.41406693	8.503948843	1.749026856	4.862103067	1.16145E-06
TRINITY_DN85629_c3_g1_i1	48.89554181	-8.592204678	1.76766143	-4.860775108	1.16927E-06
TRINITY_DN80456_c0_g4_i1	96.18496146	7.605175044	1.566541202	4.854755837	1.20535E-06
TRINITY_DN81974_c3_g3_i1	26.3630606	-9.142488921	1.882937619	-4.85543909	1.2012E-06
TRINITY_DN83651_c1_g1_i8	29.91164954	6.405216072	1.318777943	4.856932972	1.19218E-06
TRINITY_DN88584_c2_g2_i6	61.21122066	8.20434884	1.689527345	4.856002398	1.19779E-06
TRINITY_DN88095_c4_g2_i1	41.03707496	-23.21412377	4.785285455	-4.851147123	1.22749E-06
TRINITY_DN78140_c4_g1_i5	88.13308214	10.96126128	2.25893369	4.852405065	1.21973E-06
TRINITY_DN87066_c0_g1_i1	102.694622	5.056464071	1.042313918	4.851191167	1.22722E-06
TRINITY_DN86903_c0_g2_i5	945.177334	2.159927248	0.445504314	4.848274597	1.2454E-06
TRINITY_DN86785_c0_g2_i5	42.31544549	-9.912393675	2.045378984	-4.846238157	1.25824E-06
TRINITY_DN85620_c2_g6_i3	40.93473811	-23.19331979	4.785286916	-4.846798154	1.2547E-06
TRINITY_DN86705_c1_g1_i1	581.5674803	2.099560843	0.433286157	4.845667945	1.26186E-06
TRINITY_DN86795_c2_g1_i2	625.2075078	-7.815469498	1.612317206	-4.847352288	1.2512E-06
TRINITY_DN88365_c5_g2_i3	116.1014658	-2.98471346	0.616250216	-4.843346721	1.2767E-06
TRINITY_DN82493_c0_g1_i6	78.32386475	4.192278109	0.86561803	4.843103961	1.27826E-06
TRINITY_DN78728_c3_g1_i6	44.58950232	-23.14063532	4.785238744	-4.835837157	1.32586E-06
TRINITY_DN74328_c0_g1_i1	612.2856837	1.911239651	0.395299728	4.834912639	1.33204E-06
TRINITY_DN81963_c3_g1_i4	78.93893237	7.604187228	1.574088182	4.830852117	1.3595E-06
TRINITY_DN78610_c0_g4_i1	591.4989782	2.618226917	0.541902571	4.831545478	1.35477E-06
TRINITY_DN79503_c1_g2_i3	44.47122778	-23.12761067	4.785240087	-4.83311396	1.34414E-06
TRINITY_DN86759_c2_g1_i1	142.7548946	-7.422411658	1.5363653	-4.831150287	1.35746E-06

TRINITY_DN85887_c1_g3_i1	43.30962022	8.591931249	1.779519132	4.828232019	1.37751E-06
TRINITY_DN85671_c2_g2_i1	27.54221423	8.405433979	1.745783704	4.814705259	1.47417E-06
TRINITY_DN81799_c4_g3_i9	37.55429079	8.781089385	1.825463226	4.810334856	1.50678E-06
TRINITY_DN86518_c0_g1_i8	88.33267154	7.538044668	1.567424509	4.809191526	1.51542E-06
TRINITY_DN87860_c0_g1_i3	108.3927595	-7.731626032	1.608549021	-4.806584027	1.53531E-06
TRINITY_DN78710_c0_g1_i2	149.9376178	5.079983008	1.056837495	4.806777801	1.53382E-06
TRINITY_DN79869_c1_g3_i4	62.54523079	-7.946297251	1.654362845	-4.803237254	1.56121E-06
TRINITY_DN81591_c0_g1_i1	47.12455985	-7.61641635	1.586017902	-4.802225964	1.56911E-06
TRINITY_DN85150_c0_g2_i1	1003.055822	2.958815143	0.616322061	4.80076137	1.58064E-06
TRINITY_DN87166_c2_g2_i1	818.8270874	2.431301446	0.50700914	4.79537991	1.62367E-06
TRINITY_DN76141_c0_g1_i4	27.83478834	8.83637236	1.843731707	4.792656289	1.64587E-06
TRINITY_DN75744_c0_g1_i1	46.9690285	3.859471127	0.80557983	4.79092324	1.66016E-06
TRINITY_DN84608_c0_g1_i1	60.12485231	-7.974456107	1.664396565	-4.79119957	1.65787E-06
TRINITY_DN82731_c0_g2_i3	185.4413056	2.272539723	0.4748301	4.786006029	1.70133E-06
TRINITY_DN81022_c0_g1_i8	91.54022086	7.646898154	1.598043362	4.785163115	1.70849E-06
TRINITY_DN87889_c1_g1_i1	44.43887556	22.89282507	4.785255231	4.784034281	1.71811E-06
TRINITY_DN80108_c0_g3_i1	924.4567523	1.983852448	0.414841517	4.782193604	1.73393E-06
TRINITY_DN81161_c2_g3_i3	34.40053216	5.953041256	1.2456946	4.778893041	1.76263E-06
TRINITY_DN87720_c0_g2_i6	21.28270788	-8.329026472	1.74322745	-4.777934441	1.77105E-06
TRINITY_DN79856_c1_g2_i1	4425.038208	1.607338551	0.336529802	4.776214599	1.78626E-06
TRINITY_DN88361_c0_g2_i1	83.42564392	-7.73607404	1.619630274	-4.776444453	1.78422E-06
TRINITY_DN83975_c2_g2_i9	78.42786354	8.55847939	1.792583741	4.774381913	1.8026E-06
TRINITY_DN80125_c0_g2_i3	35.50402803	-7.963705822	1.66885813	-4.771948961	1.82452E-06
TRINITY_DN82098_c1_g2_i7	121.4644716	7.626411762	1.598365222	4.771382446	1.82966E-06
TRINITY_DN78347_c0_g1_i1	116.7255574	2.478831546	0.519383583	4.77264131	1.81825E-06
TRINITY_DN86622_c1_g1_i1	30.79852204	7.864490319	1.649045199	4.769117501	1.85035E-06
TRINITY_DN85184_c0_g2_i8	179.3656173	2.298583185	0.48283844	4.760563775	1.93053E-06
TRINITY_DN83065_c1_g2_i1	32.7676365	8.302814279	1.744294406	4.759984467	1.93608E-06
TRINITY_DN80860_c0_g1_i2	42.30736972	-9.314958471	1.956722636	-4.760489965	1.93124E-06
TRINITY_DN86836_c2_g1_i6	37.35996608	8.145358682	1.712218624	4.75719547	1.96301E-06
TRINITY_DN83763_c0_g1_i3	27.8948649	-8.626285324	1.814216942	-4.754825691	1.98618E-06
TRINITY_DN80540_c0_g1_i1	3476.764778	2.311151824	0.486726666	4.748356697	2.05076E-06
TRINITY_DN87747_c1_g2_i1	45.59554272	-8.560784836	1.802802139	-4.748599223	2.0483E-06
TRINITY_DN85342_c4_g5_i1	751.5762952	1.911761602	0.402719304	4.747131772	2.06322E-06
TRINITY_DN87182_c1_g1_i2	19083.76468	1.872947856	0.394774271	4.744351378	2.09175E-06
TRINITY_DN79086_c0_g5_i1	170.9621093	5.478555525	1.154761654	4.744317155	2.09211E-06
TRINITY_DN76827_c2_g2_i3	1056.518889	1.884134025	0.397377652	4.741419192	2.12226E-06
TRINITY_DN84185_c0_g1_i2	48.43935204	7.647322761	1.613869768	4.738500537	2.15305E-06
TRINITY_DN85086_c0_g2_i1	87.31029725	7.312939354	1.544380778	4.735191903	2.18848E-06
TRINITY_DN86918_c1_g2_i1	36.31908388	8.443287695	1.784403306	4.73171489	2.22631E-06
TRINITY_DN81476_c0_g1_i6	22.55591987	-8.789327375	1.860511115	-4.724146664	2.31084E-06
TRINITY_DN79004_c0_g3_i5	130.1027663	6.296409522	1.334116284	4.71953577	2.36383E-06
TRINITY_DN88420_c1_g1_i3	144.3293706	-7.492696402	1.588140302	-4.717905837	2.38285E-06
TRINITY_DN87038_c2_g1_i8	29.4943667	8.003205421	1.696179275	4.718372367	2.37739E-06
TRINITY_DN83499_c1_g1_i6	129.0003894	-4.8063649	1.021763773	-4.703988363	2.55128E-06
TRINITY_DN86072_c1_g2_i3	92.64405794	-7.708974733	1.639566974	-4.701835824	2.57833E-06
TRINITY_DN83082_c0_g1_i3	220.8831339	2.329037333	0.495990492	4.6957298	2.65657E-06
TRINITY_DN88096_c1_g1_i7	109.7668154	7.647674362	1.629720909	4.692628242	2.69717E-06
TRINITY_DN87512_c0_g1_i4	367.4703119	4.725208243	1.007361762	4.690676599	2.72303E-06
TRINITY_DN83998_c0_g1_i6	50.07153324	-7.47983572	1.595498821	-4.688086023	2.75772E-06
TRINITY_DN80545_c0_g2_i2	51.29511784	8.718808846	1.859750739	4.688159905	2.75673E-06
TRINITY_DN76989_c1_g1_i3	106.3820421	-7.461730146	1.593378781	-4.682960659	2.82761E-06
TRINITY_DN87820_c0_g1_i1	54.2255625	7.418273343	1.585230729	4.679617424	2.87411E-06
TRINITY_DN87806_c3_g3_i1	667.91344	2.649952247	0.566423712	4.678392146	2.89133E-06
TRINITY_DN81610_c1_g1_i1	150.5463766	-2.751375061	0.588312784	-4.676721531	2.91498E-06
TRINITY_DN81834_c0_g4_i1	1249.830796	2.376954522	0.508855524	4.671177596	2.99478E-06
TRINITY_DN88021_c1_g1_i1	78.4601995	8.187904274	1.752900272	4.671061101	2.99648E-06

TRINITY_DN84078_c0_g4_i1	31.67789299	8.393874511	1.797072962	4.670859051	2.99943E-06
TRINITY_DN88566_c0_g2_i9	342.2538092	8.573873007	1.83734105	4.666457001	3.06438E-06
TRINITY_DN88076_c1_g1_i7	37.96908659	-7.623625904	1.633472044	-4.667129707	3.05437E-06
TRINITY_DN80360_c0_g1_i5	277.6469089	7.203805197	1.54521967	4.661994238	3.1316E-06
TRINITY_DN86156_c0_g2_i1	170.3125367	7.60422708	1.63123339	4.661642611	3.13696E-06
TRINITY_DN79575_c5_g2_i3	33.83491287	-7.774172419	1.66763541	-4.661793802	3.13465E-06
TRINITY_DN84692_c1_g1_i1	33.87954141	8.378788706	1.799401485	4.656430917	3.21738E-06
TRINITY_DN79400_c3_g2_i3	283.7989684	3.315567819	0.711749259	4.658336878	3.18774E-06
TRINITY_DN85294_c1_g2_i1	411.1603407	7.084005456	1.521213023	4.656813574	3.21141E-06
TRINITY_DN79499_c2_g1_i1	68.71601635	-7.374864601	1.583044043	-4.658660404	3.18274E-06
TRINITY_DN76299_c0_g1_i7	42.96807297	8.393029906	1.802295656	4.656855204	3.21076E-06
TRINITY_DN87820_c0_g1_i2	59.58144859	-7.78551373	1.671879651	-4.65674292	3.21251E-06
TRINITY_DN79754_c2_g1_i1	176.0547061	4.440366952	0.954035693	4.65429856	3.25085E-06
TRINITY_DN83476_c0_g4_i5	61.87047142	-5.384670191	1.157530182	-4.65186159	3.28952E-06
TRINITY_DN87261_c0_g1_i1	48.81539051	-7.54341575	1.621428017	-4.652328485	3.28208E-06
TRINITY_DN84790_c0_g1_i1	100.4219737	4.738218271	1.019243855	4.648758239	3.33939E-06
TRINITY_DN78803_c2_g1_i9	73.0493214	-7.821132159	1.682933744	-4.647320303	3.36275E-06
TRINITY_DN81560_c0_g1_i1	145.7397108	3.368892998	0.726002656	4.640331504	3.47851E-06
TRINITY_DN81605_c0_g1_i1	125.1787272	-7.40888876	1.597024291	-4.639183513	3.49788E-06
TRINITY_DN87649_c2_g1_i7	59.1984004	-7.57830753	1.633594731	-4.639037692	3.50035E-06
TRINITY_DN87104_c0_g1_i5	46.68028741	-8.660109996	1.867644774	-4.636914962	3.53648E-06
TRINITY_DN86140_c1_g3_i6	57.21103776	8.175527861	1.764260066	4.633969798	3.58719E-06
TRINITY_DN86948_c1_g4_i1	65.55880049	-7.278959268	1.572733116	-4.628222802	3.68817E-06
TRINITY_DN84995_c1_g2_i1	59.24155972	-9.482844066	2.049688955	-4.626479566	3.71934E-06
TRINITY_DN81827_c1_g1_i5	52.3150974	7.648707723	1.653874935	4.624719536	3.75106E-06
TRINITY_DN81450_c0_g2_i2	42.38865615	7.709075177	1.668091212	4.621494989	3.80985E-06
TRINITY_DN80205_c0_g2_i4	94.23935569	-7.588996132	1.642838238	-4.619442107	3.84773E-06
TRINITY_DN80426_c0_g1_i6	41.17126227	-9.459673212	2.050592359	-4.613141744	3.96628E-06
TRINITY_DN84672_c0_g1_i3	233.7848075	7.231885018	1.567882673	4.612516704	3.97823E-06
TRINITY_DN82261_c3_g1_i8	79.77481481	-8.219492674	1.782880904	-4.610230922	4.02222E-06
TRINITY_DN80812_c2_g1_i1	173.550535	-8.083686184	1.753095353	-4.611093271	4.00557E-06
TRINITY_DN86449_c1_g1_i2	43.23268885	7.536986681	1.634756729	4.610463776	4.01772E-06
TRINITY_DN83139_c1_g1_i1	70.87100352	-8.020963296	1.7410937	-4.606853321	4.08808E-06
TRINITY_DN84424_c2_g1_i1	28.49735246	-8.923197876	1.938116511	-4.604056477	4.1434E-06
TRINITY_DN86603_c1_g1_i8	21.01055622	8.668709348	1.88384126	4.601613485	4.19231E-06
TRINITY_DN84464_c3_g1_i1	21.08912824	-8.585787697	1.866390476	-4.600209768	4.22066E-06
TRINITY_DN77340_c1_g1_i8	33.13269703	-7.819453246	1.700920646	-4.597188743	4.2823E-06
TRINITY_DN85014_c1_g11_i	418.8525107	2.541570301	0.553368746	4.592905396	4.37117E-06
TRINITY_DN78130_c1_g2_i7	106.1280375	4.394903389	0.956987911	4.59243355	4.38107E-06
TRINITY_DN85339_c2_g2_i2	135.0811524	-7.224942825	1.57470489	-4.588124968	4.47245E-06
TRINITY_DN81469_c0_g2_i3	20.36011467	8.083911207	1.763193923	4.584811177	4.54397E-06
TRINITY_DN79989_c0_g4_i1	43.58133801	7.605214808	1.659214302	4.583624188	4.56985E-06
TRINITY_DN79179_c2_g3_i2	170.8155497	2.204302328	0.481443637	4.578526246	4.68263E-06
TRINITY_DN76548_c0_g2_i1	47.57927871	-7.63782366	1.667502696	-4.58039659	4.64095E-06
TRINITY_DN84279_c0_g3_i2	1467.45802	-1.953927735	0.426757048	-4.578548246	4.68214E-06
TRINITY_DN80913_c1_g5_i2	77.5227539	10.73263838	2.343551079	4.579647729	4.6576E-06
TRINITY_DN87947_c2_g3_i1	31.96850935	-7.586203367	1.657912022	-4.5757575	4.745E-06
TRINITY_DN80258_c2_g1_i1	41.74838857	-7.951802869	1.737843878	-4.575671595	4.74695E-06
TRINITY_DN77813_c2_g2_i8	277.7605539	2.375809931	0.520357315	4.565727935	4.97764E-06
TRINITY_DN80538_c4_g2_i7	27.05479599	-7.813639137	1.711706282	-4.564824714	4.99912E-06
TRINITY_DN86333_c3_g1_i2	85.49597081	2.480032199	0.543582789	4.562381756	5.05766E-06
TRINITY_DN88181_c1_g2_i1	26.33626812	8.838444523	1.93810036	4.560364729	5.10648E-06
TRINITY_DN77754_c2_g3_i6	24.43298144	8.083392598	1.773971759	4.556663633	5.19726E-06
TRINITY_DN83520_c3_g5_i3	23.32453716	-7.645332045	1.678660373	-4.554424567	5.25292E-06
TRINITY_DN82728_c0_g1_i9	64.49656448	-8.036920944	1.764431735	-4.554962816	5.23948E-06
TRINITY_DN82502_c1_g1_i2	31.33358838	-8.121415605	1.784291075	-4.55162037	5.32343E-06
TRINITY_DN84218_c1_g1_i7	943.0326232	7.536087054	1.65561709	4.551829708	5.31814E-06

TRINITY_DN86749_c2_g2_i7	91.50655585	-9.33853556	2.052145653	-4.550620248	5.3488E-06
TRINITY_DN88409_c1_g1_i4	29.66598581	7.970754948	1.75192965	4.549700353	5.37224E-06
TRINITY_DN86918_c1_g2_i9	50.62739672	8.314721262	1.827822487	4.548976348	5.39075E-06
TRINITY_DN88368_c1_g1_i1	22.44573947	-8.332068715	1.831925449	-4.548257527	5.40919E-06
TRINITY_DN81297_c0_g3_i1	114.1238553	-3.549230228	0.78065031	-4.546504604	5.45442E-06
TRINITY_DN77966_c2_g6_i1	235.2414283	3.010505671	0.662829064	4.541903536	5.57486E-06
TRINITY_DN87722_c2_g3_i6	21.55708306	-8.568453605	1.888194964	-4.537907245	5.68152E-06
TRINITY_DN88319_c4_g1_i4	2003.976908	1.709869426	0.376905321	4.536601984	5.71679E-06
TRINITY_DN86112_c1_g9_i2	273.2649926	3.368634165	0.74302671	4.533664966	5.7969E-06
TRINITY_DN81742_c2_g1_i1	40.24206061	-8.267510256	1.824098666	-4.532381066	5.83225E-06
TRINITY_DN86865_c0_g1_i9	86.59455739	7.627466824	1.683330837	4.531175128	5.86565E-06
TRINITY_DN88669_c3_g1_i8	58.03152982	8.500625385	1.876636573	4.529713163	5.90638E-06
TRINITY_DN82326_c0_g1_i1	29.71302322	9.513310871	2.100714389	4.528607468	5.93737E-06
TRINITY_DN77419_c0_g1_i3	31.07716145	-7.657779952	1.691063605	-4.528380796	5.94374E-06
TRINITY_DN87952_c0_g1_i2	122.631419	7.287183832	1.609499145	4.527609631	5.96547E-06
TRINITY_DN88675_c1_g1_i2	42.09934664	9.541602295	2.108494064	4.525316176	6.03053E-06
TRINITY_DN85605_c2_g2_i2	67.59925249	7.668697391	1.697328494	4.51809854	6.23974E-06
TRINITY_DN84218_c1_g1_i8	1055.680462	7.259809501	1.607459369	4.516325353	6.2922E-06
TRINITY_DN78377_c1_g3_i1	58.16700512	-8.324413932	1.843885867	-4.514603686	6.34353E-06
TRINITY_DN87555_c1_g1_i1	205.7996674	8.416578797	1.864973788	4.512974311	6.39248E-06
TRINITY_DN80198_c0_g3_i3	22.58649779	-8.920065116	1.977225533	-4.511404979	6.43996E-06
TRINITY_DN85154_c3_g3_i1	62.60968883	4.390360229	0.973080506	4.511816032	6.42749E-06
TRINITY_DN86957_c0_g1_i1	41.40827801	7.287901465	1.616012874	4.509804088	6.48875E-06
TRINITY_DN79102_c1_g3_i3	994.0137184	2.134502765	0.473516477	4.507768721	6.55129E-06
TRINITY_DN88750_c4_g2_i3	86.11634755	-7.364456962	1.634412016	-4.50587544	6.60999E-06
TRINITY_DN88493_c3_g1_i9	61.58187688	5.004551766	1.111470533	4.502640077	6.71145E-06
TRINITY_DN79705_c0_g3_i3	31.49058331	-8.717300318	1.937507006	-4.499235507	6.81983E-06
TRINITY_DN85179_c1_g5_i1	271.5546169	4.052477762	0.902757525	4.488999151	7.15586E-06
TRINITY_DN82705_c2_g1_i4	40.14616597	-7.482038368	1.666365928	-4.490033217	7.12121E-06
TRINITY_DN87613_c5_g1_i1	7305.783315	2.625554782	0.584832841	4.489410642	7.14205E-06
TRINITY_DN84479_c2_g3_i1	64.04111355	8.623981412	1.920712327	4.489991182	7.12261E-06
TRINITY_DN77748_c4_g1_i1	126.9490695	-2.702861729	0.602205165	-4.488273909	7.18026E-06
TRINITY_DN86377_c2_g1_i1	29.53211115	7.882150301	1.756912547	4.486364627	7.24487E-06
TRINITY_DN79513_c1_g10_i	1550.412462	1.774703002	0.395936828	4.482288282	7.38469E-06
TRINITY_DN79627_c1_g5_i3	34.95800351	-7.877079601	1.757610501	-4.481698076	7.40515E-06
TRINITY_DN88672_c1_g1_i1	29.87228242	7.560922578	1.687938226	4.479383465	7.48589E-06
TRINITY_DN78972_c1_g1_i6	31.20149089	7.953602498	1.77600539	4.478366192	7.52165E-06
TRINITY_DN81700_c1_g5_i2	22.58181065	-8.349550159	1.864680474	-4.477737755	7.54382E-06
TRINITY_DN83567_c3_g1_i2	24.66084227	-7.537697121	1.684378016	-4.475062633	7.63889E-06
TRINITY_DN82571_c0_g1_i2	47.88065989	8.929036039	1.997268765	4.470623181	7.7992E-06
TRINITY_DN88430_c1_g1_i2	72.11795655	-4.622715533	1.0345188	-4.468469333	7.87813E-06
TRINITY_DN86445_c0_g5_i3	158.9011111	-2.587140633	0.579337328	-4.465689521	7.98113E-06
TRINITY_DN83302_c3_g3_i2	27.82536017	7.668035056	1.717157747	4.465539098	7.98674E-06
TRINITY_DN81685_c1_g2_i3	83.05768	-7.511504342	1.681769524	-4.466429101	7.9536E-06
TRINITY_DN85405_c4_g1_i5	310.6149736	3.182072407	0.712873663	4.463725586	8.05467E-06
TRINITY_DN85566_c2_g4_i5	1028.532098	2.729190045	0.611817223	4.460793094	8.16569E-06
TRINITY_DN88356_c1_g1_i1	29.34824387	7.367648885	1.652499354	4.458488208	8.25397E-06
TRINITY_DN84300_c1_g1_i4	65.60045206	7.417328194	1.66438837	4.456488838	8.33129E-06
TRINITY_DN81568_c0_g1_i7	21.72011617	-8.298338416	1.864733003	-4.450148308	8.5811E-06
TRINITY_DN85541_c1_g2_i7	374.9201977	3.974680779	0.893473311	4.448572476	8.64429E-06
TRINITY_DN82698_c0_g1_i9	20.54645789	7.690017404	1.72972311	4.445808325	8.7562E-06
TRINITY_DN85224_c1_g1_i8	28.82077078	-7.473963814	1.686740853	-4.431008949	9.37932E-06
TRINITY_DN84479_c2_g3_i2	369.4722881	1.700870919	0.384031088	4.428992782	9.46742E-06
TRINITY_DN81742_c2_g1_i1	28.75803019	-8.209486723	1.855958706	-4.423313244	9.71987E-06
TRINITY_DN79694_c1_g5_i8	466.6753145	1.738179187	0.392934065	4.42358996	9.70742E-06
TRINITY_DN85704_c1_g3_i1	282.3950268	3.915886422	0.885692294	4.421271866	9.81216E-06
TRINITY_DN87030_c1_g3_i5	358.7643017	-8.307855455	1.87908305	-4.421228458	9.81413E-06

TRINITY_DN85916_c0_g1_i7	148.5647101	2.324077732	0.525664645	4.421217513	9.81463E-06
TRINITY_DN74725_c0_g1_i1	35.53254023	4.270265885	0.966084662	4.420177705	9.86198E-06
TRINITY_DN86813_c4_g5_i1	1320.180848	2.461182704	0.55734877	4.415875369	1.00602E-05
TRINITY_DN78947_c4_g2_i4	53.22470762	8.635822488	1.955411686	4.416370501	1.00372E-05
TRINITY_DN86690_c3_g1_i1	20.60588438	-8.022459748	1.819116466	-4.410085829	1.0333E-05
TRINITY_DN86511_c0_g1_i2	19.65438503	-7.622982907	1.728917274	-4.409107957	1.03797E-05
TRINITY_DN82503_c2_g2_i3	44.1291176	7.468352624	1.694209156	4.408164479	1.0425E-05
TRINITY_DN88612_c0_g2_i6	69.80725435	-7.307669717	1.658434173	-4.406367064	1.05119E-05
TRINITY_DN82810_c0_g2_i2	295.2468792	2.648564658	0.601630854	4.402308559	1.07105E-05
TRINITY_DN82316_c1_g1_i3	27.96278892	-7.983780495	1.814369239	-4.400306356	1.08098E-05
TRINITY_DN83207_c0_g1_i3	28.17937471	-8.150047467	1.853054611	-4.398169066	1.09168E-05
TRINITY_DN87762_c3_g5_i1	2279.229806	2.284177572	0.519556851	4.396395826	1.10063E-05
TRINITY_DN81285_c2_g2_i1	41.58497964	7.91667888	1.800736004	4.396357301	1.10083E-05
TRINITY_DN82410_c2_g2_i5	18.0099021	7.953362567	1.80988412	4.394404304	1.11077E-05
TRINITY_DN87838_c0_g4_i3	25.39227593	-8.030229572	1.827313738	-4.39455437	1.11E-05
TRINITY_DN87681_c1_g2_i8	29.22356988	-7.318875876	1.66567177	-4.39394844	1.1131E-05
TRINITY_DN79553_c1_g2_i3	52.10059454	7.019417683	1.598251151	4.391936574	1.12345E-05
TRINITY_DN83051_c4_g1_i7	38.75916741	-7.669462529	1.746104467	-4.392327421	1.12144E-05
TRINITY_DN77717_c2_g2_i2	84.07782627	-8.04916454	1.833052506	-4.39112601	1.12765E-05
TRINITY_DN87737_c1_g1_i7	23.78648051	7.807047391	1.778579007	4.389485855	1.13619E-05
TRINITY_DN79702_c0_g5_i2	205.5701596	2.170044698	0.494678385	4.386778892	1.15042E-05
TRINITY_DN72088_c0_g1_i1	144.2403524	4.717485138	1.075857768	4.384859485	1.16061E-05
TRINITY_DN81425_c0_g3_i2	18.39288026	7.710076638	1.761322418	4.377436272	1.20083E-05
TRINITY_DN84452_c0_g1_i1	30.34287419	-8.621372191	1.970277659	-4.375714333	1.21035E-05
TRINITY_DN87355_c1_g3_i1	46.829308	-7.129903124	1.630053867	-4.374029145	1.21974E-05
TRINITY_DN85059_c1_g2_i8	38.28634357	-8.143097253	1.861946287	-4.373432957	1.22308E-05
TRINITY_DN80150_c2_g1_i1	79.6351556	-2.658126153	0.607894586	-4.372676143	1.22733E-05
TRINITY_DN74883_c0_g1_i4	38.41174776	-7.981131051	1.825392814	-4.372281403	1.22955E-05
TRINITY_DN86183_c3_g1_i3	33.0429397	7.442633991	1.702097881	4.372623968	1.22762E-05
TRINITY_DN87215_c1_g3_i4	30.61927352	-5.93096865	1.356463886	-4.372374902	1.22902E-05
TRINITY_DN87144_c2_g2_i2	57.46549796	4.688117626	1.072928715	4.369458623	1.24555E-05
TRINITY_DN76983_c1_g5_i2	150.7191724	4.875346509	1.11581539	4.369312837	1.24638E-05
TRINITY_DN85360_c0_g1_i6	101.3845078	7.291733357	1.670413727	4.365225955	1.26991E-05
TRINITY_DN87053_c0_g1_i9	54.11540738	-7.138534665	1.634792138	-4.366631391	1.26177E-05
TRINITY_DN81786_c1_g1_i2	51.68408428	-7.238612998	1.657508716	-4.367164363	1.2587E-05
TRINITY_DN86696_c0_g1_i1	47.24346019	8.187823782	1.876275698	4.363870295	1.27781E-05
TRINITY_DN85003_c1_g1_i6	58.66099729	7.667418375	1.75663142	4.364841872	1.27215E-05
TRINITY_DN84212_c0_g1_i4	29.31729008	7.648282485	1.751077932	4.367756766	1.25529E-05
TRINITY_DN87178_c0_g1_i9	32.80264152	8.356793737	1.913879529	4.366415759	1.26302E-05
TRINITY_DN77319_c0_g1_i1	40.10178792	7.204340873	1.650970376	4.363700875	1.2788E-05
TRINITY_DN83180_c0_g2_i1	22.10129066	-7.714782926	1.767814218	-4.364023577	1.27692E-05
TRINITY_DN86430_c0_g1_i1	705.2211252	2.573160521	0.589457602	4.365302125	1.26947E-05
TRINITY_DN79201_c0_g1_i7	48.69702738	9.059970905	2.076464517	4.36317155	1.2819E-05
TRINITY_DN81210_c1_g1_i6	40.71719254	-3.334104344	0.764822733	-4.359316479	1.30469E-05
TRINITY_DN77988_c0_g4_i1	167.7442536	4.294548142	0.985080185	4.359592454	1.30305E-05
TRINITY_DN87728_c0_g7_i1	29.84986999	-7.189182549	1.649955357	-4.357198223	1.31738E-05
TRINITY_DN81469_c0_g1_i9	28.3346041	7.367470041	1.690718897	4.357596081	1.31499E-05
TRINITY_DN81512_c0_g5_i1	36.44228383	7.08405921	1.626511246	4.355370569	1.32842E-05
TRINITY_DN82811_c0_g2_i1	62.30634822	-7.446411111	1.709903548	-4.354872017	1.33145E-05
TRINITY_DN86952_c1_g1_i7	52.98004764	6.82857061	1.569020413	4.352123499	1.34825E-05
TRINITY_DN87754_c1_g6_i1	610.2460999	2.231336652	0.513064031	4.349041283	1.36734E-05
TRINITY_DN83234_c1_g4_i3	61.17850747	7.203562442	1.656933369	4.347526928	1.37681E-05
TRINITY_DN80982_c1_g3_i2	94.8663711	8.870524625	2.040158855	4.347957808	1.37411E-05
TRINITY_DN82314_c0_g3_i3	21.30850596	-7.835377436	1.802591725	-4.346728839	1.38183E-05
TRINITY_DN85937_c3_g1_i1	29.46691579	9.258548678	2.130847604	4.345007434	1.39271E-05
TRINITY_DN87158_c0_g2_i6	831.0473194	3.263164105	0.751205411	4.34390389	1.39973E-05
TRINITY_DN83615_c0_g1_i6	143.225399	4.682395904	1.07816692	4.342922992	1.40599E-05

TRINITY_DN77819_c1_g5_i1	53.42056788	2.706089213	0.623471532	4.340357296	1.42251E-05
TRINITY_DN88648_c2_g1_i9	45176.93892	2.479189349	0.57144808	4.338433248	1.43502E-05
TRINITY_DN76515_c0_g1_i6	31.19236963	8.2023161	1.891332497	4.336792242	1.44577E-05
TRINITY_DN76616_c0_g1_i1	898.6646764	2.278539837	0.525512791	4.33584087	1.45204E-05
TRINITY_DN87521_c1_g7_i2	3239.565509	2.479259705	0.57225196	4.332461707	1.47451E-05
TRINITY_DN88394_c0_g1_i5	17.15873697	7.690022538	1.776188932	4.32950707	1.49443E-05
TRINITY_DN88793_c1_g1_i1	29.33775996	-7.363879303	1.701584058	-4.327661199	1.50701E-05
TRINITY_DN86145_c2_g1_i1	71.58856277	6.985969865	1.614544411	4.326898547	1.51224E-05
TRINITY_DN79694_c1_g5_i1	2183.127945	1.583805717	0.366453491	4.321982882	1.54633E-05
TRINITY_DN85473_c0_g4_i1	55.69136457	7.584274785	1.755424499	4.320479056	1.55691E-05
TRINITY_DN78821_c3_g5_i8	21.25569794	-8.398860052	1.944510563	-4.319266869	1.56548E-05
TRINITY_DN88294_c3_g3_i3	62.81505823	-7.441048751	1.724271246	-4.315474591	1.5926E-05
TRINITY_DN85741_c1_g2_i2	63.04166926	-5.161079	1.196120368	-4.314849189	1.59712E-05
TRINITY_DN85826_c2_g1_i6	57.9975962	7.441333628	1.725215135	4.313278662	1.60851E-05
TRINITY_DN79736_c0_g2_i2	15.420195	-7.683558964	1.781303672	-4.31344699	1.60729E-05
TRINITY_DN87949_c2_g3_i4	18.65952533	-7.716012777	1.789072822	-4.312855621	1.61159E-05
TRINITY_DN76917_c0_g3_i2	21.96741972	7.969333723	1.849290933	4.309399663	1.63698E-05
TRINITY_DN84780_c0_g2_i4	49.94940039	4.753891678	1.103355453	4.308576776	1.64308E-05
TRINITY_DN86474_c2_g1_i6	19.32561032	8.491962728	1.97218622	4.30586252	1.66336E-05
TRINITY_DN83085_c0_g3_i4	23.81924773	-4.688718002	1.089653398	-4.302944412	1.68543E-05
TRINITY_DN85605_c2_g2_i1	88.36625512	6.952714104	1.616127468	4.302082751	1.692E-05
TRINITY_DN82403_c1_g1_i5	50.62854025	7.810002082	1.814873057	4.30333243	1.68248E-05
TRINITY_DN82501_c0_g2_i3	75.11620173	-7.151311144	1.662309547	-4.302033371	1.69238E-05
TRINITY_DN83917_c1_g1_i3	4354.723043	2.205321711	0.512697853	4.301406171	1.69718E-05
TRINITY_DN86509_c1_g1_i6	49.05656949	-4.505362115	1.047599289	-4.30065404	1.70295E-05
TRINITY_DN82136_c2_g1_i1	66.28944028	7.624471876	1.774421859	4.296876662	1.73222E-05
TRINITY_DN79494_c1_g1_i1	628.7197879	1.764244161	0.410867862	4.293945384	1.75526E-05
TRINITY_DN86696_c1_g2_i1	34.18960659	-8.002477522	1.864183567	-4.292751884	1.76472E-05
TRINITY_DN78849_c2_g3_i7	2985.681975	1.825635771	0.425448366	4.291086574	1.77801E-05
TRINITY_DN84151_c0_g1_i1	22.33411168	8.838079175	2.061374689	4.287468563	1.80721E-05
TRINITY_DN79400_c3_g4_i2	123.9156666	3.054308241	0.712592539	4.286191723	1.81762E-05
TRINITY_DN85053_c0_g1_i5	33.12183672	9.42878587	2.201562798	4.282769439	1.84581E-05
TRINITY_DN79093_c1_g1_i9	25.47229805	-9.172638366	2.142827079	-4.280624627	1.86369E-05
TRINITY_DN82501_c0_g1_i4	44.89054334	-7.348976483	1.716957128	-4.280232956	1.86698E-05
TRINITY_DN87518_c0_g3_i3	27.85353794	-7.768666278	1.814991552	-4.280276825	1.86661E-05
TRINITY_DN87108_c2_g2_i2	16857.9164	1.626897442	0.380721441	4.273196269	1.92691E-05
TRINITY_DN84280_c0_g3_i3	190.8333973	2.231946646	0.522580638	4.27100907	1.9459E-05
TRINITY_DN87849_c0_g1_i3	155.8150494	2.114553443	0.49508192	4.27111829	1.94495E-05
TRINITY_DN85339_c2_g2_i4	60.49885562	6.846090161	1.603376979	4.269794473	1.95653E-05
TRINITY_DN80835_c0_g1_i4	309.5845525	2.972741108	0.696179083	4.270081049	1.95402E-05
TRINITY_DN81485_c0_g3_i1	101.9086369	2.526333856	0.591924257	4.268001904	1.97232E-05
TRINITY_DN79702_c0_g6_i1	23.74326108	7.536381842	1.76799536	4.262670601	2.01998E-05
TRINITY_DN88435_c1_g2_i3	54.80901527	7.748197809	1.817994159	4.261948681	2.02652E-05
TRINITY_DN86447_c1_g2_i2	21.80632643	-7.546382888	1.771211106	-4.260577897	2.03899E-05
TRINITY_DN87220_c1_g2_i3	85.17373814	3.949578451	0.927114731	4.260075174	2.04358E-05
TRINITY_DN76957_c0_g1_i6	13.03074918	-7.815884959	1.835547767	-4.258066775	2.06202E-05
TRINITY_DN86987_c0_g1_i1	28.68404442	4.806318294	1.128676243	4.258367556	2.05925E-05
TRINITY_DN87128_c2_g1_i6	28.48903961	-7.526009465	1.768646341	-4.255237065	2.08828E-05
TRINITY_DN81139_c2_g1_i3	38.24736025	7.203354867	1.693059646	4.254637387	2.09388E-05
TRINITY_DN83558_c1_g1_i1	226.9673592	3.560111168	0.836957135	4.253636201	2.10327E-05
TRINITY_DN88723_c4_g2_i8	24978.12439	1.48863144	0.349979779	4.253478423	2.10475E-05
TRINITY_DN85694_c0_g1_i1	267.6441704	-2.899169949	0.681752795	-4.252523746	2.11375E-05
TRINITY_DN87033_c0_g2_i1	27.5226751	7.419238224	1.745489684	4.250519665	2.13275E-05
TRINITY_DN87348_c1_g4_i5	25.72607449	7.341237428	1.727301465	4.250119378	2.13657E-05
TRINITY_DN82277_c0_g1_i1	26.2984938	-7.765661246	1.826889193	-4.250756572	2.1305E-05
TRINITY_DN83817_c3_g3_i2	17.45230883	8.625005475	2.030184058	4.248385974	2.15316E-05
TRINITY_DN86215_c1_g1_i1	287.8591722	2.468562136	0.58139204	4.245951041	2.17668E-05

TRINITY_DN88240_c0_g1_i1	85.19338657	-6.881717867	1.620642254	-4.246290537	2.17339E-05
TRINITY_DN83902_c0_g1_i1	22.44319366	-8.926589212	2.102172969	-4.246362855	2.17269E-05
TRINITY_DN78997_c1_g5_i2	81.62729325	2.760721571	0.650317435	4.245190767	2.18408E-05
TRINITY_DN86333_c3_g5_i4	109.3030936	2.333545467	0.549805201	4.244313193	2.19264E-05
TRINITY_DN88481_c1_g1_i1	26.66883077	8.327368008	1.96408135	4.239828461	2.23691E-05
TRINITY_DN88375_c0_g6_i1	24.16973201	7.490395288	1.767449773	4.237967836	2.25552E-05
TRINITY_DN82690_c1_g2_i4	21.64648827	7.342250271	1.732319756	4.238392043	2.25126E-05
TRINITY_DN83369_c0_g1_i5	206.4875781	-7.006940037	1.653881023	-4.236665116	2.26864E-05
TRINITY_DN84829_c3_g1_i5	51.10238691	7.825245459	1.849157549	4.231789477	2.31839E-05
TRINITY_DN84509_c0_g1_i2	446.9504514	-6.499924581	1.535971577	-4.23180004	2.31828E-05
TRINITY_DN74245_c0_g1_i1	25.70304144	-7.218913942	1.706285651	-4.230776915	2.32886E-05
TRINITY_DN82945_c0_g1_i5	25.89684845	-8.107653011	1.917416893	-4.22842473	2.35333E-05
TRINITY_DN86430_c0_g3_i5	165.8678732	2.516625517	0.595138732	4.228636754	2.35112E-05
TRINITY_DN78202_c1_g1_i2	67.46693532	-3.360204941	0.794950445	-4.226936362	2.36895E-05
TRINITY_DN79556_c1_g1_i5	144.383902	-7.168838682	1.697191664	-4.223941724	2.40066E-05
TRINITY_DN86949_c0_g1_i5	35.75848758	-4.538038795	1.075198117	-4.220653591	2.43595E-05
TRINITY_DN84780_c0_g1_i2	25.92029505	7.625168211	1.807907987	4.217674941	2.46834E-05
TRINITY_DN83296_c1_g1_i1	54.33863005	7.441576173	1.764339616	4.217768565	2.46732E-05
TRINITY_DN77563_c1_g5_i5	47.09015482	3.954113231	0.937770944	4.216502179	2.48121E-05
TRINITY_DN84775_c0_g5_i4	158.3658189	8.019693995	1.902139647	4.216143649	2.48515E-05
TRINITY_DN80679_c1_g1_i3	43.87557841	7.950567053	1.886242316	4.215029525	2.49746E-05
TRINITY_DN87571_c0_g2_i3	1794.281118	2.745834556	0.651816566	4.212587868	2.52461E-05
TRINITY_DN84719_c0_g2_i7	33.35231089	7.667023484	1.820967379	4.210412319	2.54905E-05
TRINITY_DN79161_c1_g1_i8	45.68408754	7.366911278	1.750549697	4.208341694	2.57252E-05
TRINITY_DN80650_c0_g3_i2	20.6200283	8.162099284	1.939518035	4.208313167	2.57284E-05
TRINITY_DN82177_c1_g4_i1	1746.595621	1.918615712	0.456082067	4.206733501	2.59088E-05
TRINITY_DN83009_c1_g1_i1	104.8465926	7.341129738	1.745397301	4.205993521	2.59938E-05
TRINITY_DN81649_c0_g1_i4	23.93569414	7.491830637	1.782878249	4.202098849	2.64452E-05
TRINITY_DN87261_c0_g1_i5	50.42747108	8.403290378	2.000192945	4.201239885	2.65457E-05
TRINITY_DN76939_c1_g4_i1	120.5400649	-2.451624324	0.58379115	-4.199488675	2.67518E-05
TRINITY_DN79941_c1_g1_i3	14.6184743	-7.658938821	1.824137964	-4.198662038	2.68497E-05
TRINITY_DN81548_c3_g1_i1	1941.055854	1.592760064	0.379324928	4.198933279	2.68175E-05
TRINITY_DN83306_c1_g3_i1	33.68009726	7.114932225	1.695373227	4.196676055	2.70861E-05
TRINITY_DN86251_c2_g4_i1	799.303106	2.261572324	0.538894668	4.196687138	2.70848E-05
TRINITY_DN88274_c1_g3_i3	55.45373341	-6.802913006	1.6208396	-4.19715375	2.7029E-05
TRINITY_DN85616_c2_g2_i8	32.5941884	-5.38649529	1.283147169	-4.197878014	2.69428E-05
TRINITY_DN83662_c0_g1_i6	42.62013965	7.261500701	1.731057682	4.194834625	2.73071E-05
TRINITY_DN82521_c2_g1_i7	168.3300501	-4.890060073	1.16593773	-4.194100548	2.73956E-05
TRINITY_DN87835_c4_g2_i2	7291.574292	2.838273545	0.676917459	4.1929389	2.75364E-05
TRINITY_DN85631_c0_g1_i4	748.4478834	1.855770338	0.442658795	4.192326824	2.76108E-05
TRINITY_DN76657_c0_g3_i1	1482.00124	2.257133405	0.538466701	4.191778994	2.76776E-05
TRINITY_DN85838_c0_g2_i1	63.59183402	-7.10428112	1.694999293	-4.191318042	2.77339E-05
TRINITY_DN87613_c5_g1_i5	1650.427593	2.690537642	0.64202449	4.190708742	2.78084E-05
TRINITY_DN86693_c2_g2_i1	31.07636987	7.051659171	1.68408738	4.187228794	2.82381E-05
TRINITY_DN82940_c4_g1_i1	41.62320974	-7.068920571	1.688463115	-4.186600528	2.83164E-05
TRINITY_DN77739_c2_g1_i2	31.00771271	7.902060903	1.887129188	4.187344963	2.82237E-05
TRINITY_DN84848_c1_g2_i8	174.7504147	20.04119555	4.786565454	4.186967826	2.82706E-05
TRINITY_DN80090_c0_g1_i8	680.6848717	-1.732399864	0.414016606	-4.184372896	2.85954E-05
TRINITY_DN79991_c0_g4_i2	16.66790791	-7.647897377	1.82796071	-4.183841226	2.86624E-05
TRINITY_DN86183_c3_g1_i1	26.26826552	7.114619278	1.701294646	4.181885422	2.89102E-05
TRINITY_DN86810_c4_g4_i3	22.73273893	7.145772249	1.709586365	4.179825247	2.91733E-05
TRINITY_DN85274_c2_g4_i9	1369.288772	2.26847885	0.542812811	4.179118114	2.92642E-05
TRINITY_DN87090_c0_g1_i1	37.64239296	9.489802268	2.271611525	4.177563886	2.94648E-05
TRINITY_DN86189_c0_g1_i8	202.6168582	2.205669317	0.528089558	4.176695566	2.95774E-05
TRINITY_DN86964_c3_g2_i9	108.8166548	6.64775145	1.591999703	4.175724052	2.9704E-05
TRINITY_DN86759_c2_g1_i1	23.45692641	7.603968379	1.823155239	4.170773951	3.03567E-05
TRINITY_DN79037_c0_g3_i1	136.3237792	3.268302598	0.783602458	4.17086823	3.03441E-05

TRINITY_DN83556_c2_g7_i1	178.4739984	2.016711925	0.483462868	4.171389488	3.02748E-05
TRINITY_DN88394_c0_g1_i2	22.53762503	7.933686215	1.902963984	4.169120531	3.05777E-05
TRINITY_DN81156_c1_g1_i3	114.2923432	-7.852065168	1.883761261	-4.168291031	3.06892E-05
TRINITY_DN88601_c2_g2_i4	387.2176261	7.202194306	1.727980846	4.167982719	3.07307E-05
TRINITY_DN78169_c2_g8_i1	71.33746932	3.103821439	0.744784249	4.167410148	3.0808E-05
TRINITY_DN83473_c2_g2_i8	39.55352838	6.918477698	1.660217451	4.167211767	3.08348E-05
TRINITY_DN85256_c0_g1_i6	16.68876077	-8.331954308	1.999680073	-4.166643664	3.09117E-05
TRINITY_DN87952_c0_g2_i1	49.55587647	4.443079438	1.06702167	4.164001127	3.12719E-05
TRINITY_DN81694_c0_g1_i2	90.96596162	6.881043514	1.652589566	4.163794602	3.13002E-05
TRINITY_DN87493_c1_g4_i6	49.93117195	-6.807240553	1.63595044	-4.161031036	3.16814E-05
TRINITY_DN78805_c0_g1_i1	24.75832705	7.231877398	1.738254813	4.160424204	3.17657E-05
TRINITY_DN87579_c0_g1_i1	837.1163841	-2.167401498	0.521315027	-4.157565747	3.21657E-05
TRINITY_DN86215_c1_g1_i1	183.4136427	2.752916195	0.662224087	4.157076506	3.22346E-05
TRINITY_DN82629_c2_g1_i1	646.9692871	1.698381436	0.408666732	4.155908231	3.23998E-05
TRINITY_DN87963_c2_g1_i1	32.85349202	7.145252158	1.720667751	4.152604217	3.28713E-05
TRINITY_DN83670_c2_g1_i8	1507.506154	2.375694998	0.572076859	4.152754932	3.28496E-05
TRINITY_DN86507_c1_g1_i2	32.0389615	7.46553572	1.798337805	4.151353376	3.30515E-05
TRINITY_DN81025_c2_g2_i5	31.15234076	-9.470242611	2.282041109	-4.149900093	3.3262E-05
TRINITY_DN81237_c2_g1_i3	13.58684714	-7.678684237	1.851858317	-4.146475011	3.37633E-05
TRINITY_DN87754_c1_g8_i2	1090.640764	2.308156936	0.557020061	4.143759084	3.41659E-05
TRINITY_DN85932_c0_g1_i7	150.5462438	-7.524374726	1.816940677	-4.141233019	3.45444E-05
TRINITY_DN79953_c1_g1_i7	17.09197971	-7.558599808	1.826017464	-4.139390756	3.48229E-05
TRINITY_DN77078_c0_g1_i5	255.622544	2.676799075	0.646579756	4.139936412	3.47402E-05
TRINITY_DN86933_c1_g2_i3	141.2021578	-3.027756365	0.73161017	-4.138483155	3.4961E-05
TRINITY_DN82733_c0_g1_i3	127.9210082	6.729713331	1.626244533	4.138192747	3.50052E-05
TRINITY_DN79513_c1_g1_i2	33.59792904	4.171610277	1.007610743	4.140101034	3.47153E-05
TRINITY_DN87033_c0_g2_i7	57.47784767	7.174635799	1.733712986	4.138306547	3.49879E-05
TRINITY_DN86243_c0_g1_i1	280.8022305	6.367524879	1.538721058	4.13819311	3.50052E-05
TRINITY_DN87038_c2_g1_i6	48.32721881	-6.902610977	1.668229116	-4.13768763	3.50824E-05
TRINITY_DN87658_c1_g4_i6	22.58577904	-7.541874189	1.8235901	-4.13572885	3.5383E-05
TRINITY_DN87055_c0_g1_i2	1506.745711	3.523255141	0.852234978	4.134135809	3.56293E-05
TRINITY_DN79201_c0_g1_i8	33.03716087	-8.352590088	2.021051027	-4.132795251	3.58378E-05
TRINITY_DN75908_c0_g1_i4	28.04656653	4.999945015	1.210313342	4.131116168	3.61006E-05
TRINITY_DN76080_c0_g1_i2	167.7734115	2.581136468	0.624909593	4.13041582	3.62108E-05
TRINITY_DN88435_c1_g2_i1	92.71742851	6.881385807	1.666264218	4.12982871	3.63034E-05
TRINITY_DN81471_c0_g3_i6	23.54496488	-7.032614778	1.703594843	-4.128102881	3.65768E-05
TRINITY_DN83971_c0_g2_i6	43.01209455	7.202729847	1.745352429	4.126805411	3.67837E-05
TRINITY_DN77074_c1_g1_i4	42.50874442	7.535613865	1.826938233	4.124722845	3.71181E-05
TRINITY_DN88360_c3_g1_i6	43.10051577	7.082880499	1.717718612	4.123423039	3.73283E-05
TRINITY_DN82022_c1_g1_i2	24.65715004	-7.999473189	1.940338818	-4.122719762	3.74425E-05
TRINITY_DN66077_c0_g1_i1	29.74020295	9.521166635	2.309936608	4.121830271	3.75874E-05
TRINITY_DN86515_c0_g2_i9	26.06697266	-7.476771111	1.814179013	-4.121297324	3.76745E-05
TRINITY_DN84128_c1_g2_i9	25.35017338	-7.259967895	1.761931102	-4.120460718	3.78116E-05
TRINITY_DN86972_c0_g2_i1	22.68148103	-7.002333625	1.699498313	-4.120235702	3.78485E-05
TRINITY_DN83223_c1_g1_i5	34.62393921	-7.38523676	1.792711501	-4.119590215	3.79547E-05
TRINITY_DN80870_c3_g1_i1	200.5858803	-1.794735502	0.435729157	-4.118924506	3.80645E-05
TRINITY_DN84056_c1_g1_i6	52.29248548	-4.725829943	1.147973834	-4.116670436	3.84385E-05
TRINITY_DN86085_c1_g4_i4	26.50190091	7.260924108	1.763747563	4.116759258	3.84237E-05
TRINITY_DN82570_c0_g2_i4	23.99623335	7.051872171	1.712760339	4.117255644	3.83411E-05
TRINITY_DN85620_c2_g5_i4	51.74124449	7.083416518	1.721213633	4.115361615	3.86573E-05
TRINITY_DN86688_c1_g4_i8	30.28833292	6.84575629	1.663633738	4.11494197	3.87277E-05
TRINITY_DN87112_c1_g1_i3	96.37091041	9.305718767	2.261267065	4.115267459	3.86731E-05
TRINITY_DN81693_c1_g2_i5	27.925008	-7.658066825	1.861606312	-4.113687612	3.89388E-05
TRINITY_DN86086_c2_g2_i9	18.80643996	7.825590075	1.904081273	4.109903388	3.95825E-05
TRINITY_DN83526_c1_g1_i2	22.82197104	-7.121753899	1.73332522	-4.108723404	3.97852E-05
TRINITY_DN88381_c2_g1_i1	27.57542975	7.366722863	1.794288497	4.105651278	4.03177E-05
TRINITY_DN82269_c0_g2_i1	29.95773512	8.005091429	1.950685023	4.103733476	4.06536E-05

TRINITY_DN87907_c1_g1_i1	26.24195976	8.593756917	2.094251228	4.103498569	4.06949E-05
TRINITY_DN84862_c2_g3_i5	13.83302428	-7.864768628	1.917043817	-4.102550269	4.08621E-05
TRINITY_DN85640_c1_g2_i8	57.6654734	2.692289413	0.65693727	4.098244286	4.16296E-05
TRINITY_DN81808_c1_g3_i3	29.63329553	7.583979408	1.850467056	4.098413632	4.15991E-05
TRINITY_DN82599_c2_g2_i1	25.9705265	-8.277609767	2.02015665	-4.097508858	4.1762E-05
TRINITY_DN76464_c2_g2_i6	107.4923947	2.658887067	0.649169308	4.095829907	4.20659E-05
TRINITY_DN83510_c0_g2_i4	28.37932584	7.286808736	1.77893585	4.09616161	4.20057E-05
TRINITY_DN85225_c1_g1_i2	703.5698406	1.935503066	0.472657676	4.094936283	4.22285E-05
TRINITY_DN81752_c0_g1_i1	24.44274766	-7.676247434	1.875219054	-4.093520391	4.24873E-05
TRINITY_DN83429_c1_g1_i1	16.45683906	-8.441586021	2.063521526	-4.090864046	4.29769E-05
TRINITY_DN88214_c1_g3_i3	13.81024227	-7.64881535	1.870929244	-4.088244051	4.34651E-05
TRINITY_DN82890_c0_g3_i3	24.86084378	8.440574555	2.065071145	4.087304485	4.36414E-05
TRINITY_DN88060_c3_g2_i1	43.48008634	-5.194119064	1.270978116	-4.08671007	4.37533E-05
TRINITY_DN80727_c3_g3_i3	46.94203611	-6.819269543	1.668744223	-4.086467806	4.3799E-05
TRINITY_DN87256_c0_g2_i1	28.04224484	9.436361375	2.310034716	4.084943534	4.40875E-05
TRINITY_DN78772_c0_g4_i1	36.82121825	9.387942488	2.298475903	4.08442067	4.41869E-05
TRINITY_DN87628_c1_g4_i3	88.23665578	-7.083005215	1.734350795	-4.083951896	4.42762E-05
TRINITY_DN82746_c1_g1_i8	83.85739212	6.688604345	1.638505617	4.082136964	4.46235E-05
TRINITY_DN88009_c0_g1_i1	39.56444117	9.531017103	2.33542956	4.081055265	4.48317E-05
TRINITY_DN80675_c1_g1_i8	14.32095805	-7.975673584	1.955650759	-4.078270902	4.53719E-05
TRINITY_DN83033_c0_g2_i2	401.2318473	4.495292126	1.102613003	4.076944598	4.56314E-05
TRINITY_DN86779_c1_g2_i2	80.51850055	-4.416559409	1.083532634	-4.076074196	4.58024E-05
TRINITY_DN82244_c0_g1_i3	20.13320134	7.670146716	1.882604503	4.074220955	4.61686E-05
TRINITY_DN80832_c0_g3_i4	40.02587487	4.89082446	1.200972633	4.072386269	4.65339E-05
TRINITY_DN82422_c0_g5_i4	24.89450105	-7.378150644	1.811711908	-4.072474553	4.65163E-05
TRINITY_DN79168_c1_g1_i1	10898.17668	1.501305994	0.368764563	4.071177503	4.67761E-05
TRINITY_DN80633_c0_g2_i7	18.74871602	7.114722191	1.748312821	4.069478932	4.71184E-05
TRINITY_DN87806_c3_g3_i1	223.6041641	2.577600152	0.633638976	4.067931817	4.74323E-05
TRINITY_DN86652_c0_g1_i1	238.029268	9.337733522	2.295233473	4.068315329	4.73543E-05
TRINITY_DN81843_c1_g1_i3	838.7591251	2.82758782	0.695094972	4.067915803	4.74355E-05
TRINITY_DN79123_c0_g2_i4	22.28828166	-7.576414351	1.863832318	-4.064965651	4.80395E-05
TRINITY_DN81566_c0_g1_i1	53.70734431	4.777089397	1.175337343	4.064441094	4.81477E-05
TRINITY_DN88655_c1_g1_i9	33.48777928	-8.073769546	1.987951225	-4.06135193	4.87894E-05
TRINITY_DN87180_c3_g1_i2	155.3857827	2.132040371	0.525022476	4.060855426	4.88932E-05
TRINITY_DN86196_c1_g2_i2	1121.894113	3.395373818	0.836356626	4.059720114	4.91316E-05
TRINITY_DN79031_c0_g3_i1	54.47355528	3.973987687	0.97917898	4.05848958	4.93911E-05
TRINITY_DN82613_c0_g1_i3	26.57164722	7.443588532	1.83437921	4.0578243	4.9532E-05
TRINITY_DN85150_c0_g2_i1	335.9421387	6.559070156	1.616759195	4.056924603	4.97231E-05
TRINITY_DN87866_c0_g1_i1	58.38879994	-9.194975694	2.266572078	-4.056776214	4.97547E-05
TRINITY_DN86414_c0_g5_i4	20.42696131	7.790622166	1.920449741	4.056665479	4.97783E-05
TRINITY_DN81933_c1_g3_i5	17.6179598	-5.755077213	1.418937317	-4.05590659	4.99402E-05
TRINITY_DN86699_c0_g1_i5	19.63314075	-7.017532395	1.730106933	-4.056126394	4.98933E-05
TRINITY_DN82547_c0_g3_i1	19.25658487	-7.434804137	1.833434311	-4.055124361	5.01076E-05
TRINITY_DN76475_c0_g1_i1	103.0007646	4.233216425	1.044030683	4.054685839	5.02017E-05
TRINITY_DN78846_c0_g6_i2	37.9753151	-5.094269182	1.256341028	-4.054845834	5.01674E-05
TRINITY_DN85505_c1_g6_i2	20.17258342	8.952488119	2.20832438	4.05397332	5.0355E-05
TRINITY_DN80256_c2_g1_i1	81.34726267	9.01102949	2.223860452	4.05197614	5.07868E-05
TRINITY_DN77540_c0_g1_i4	26.27001884	-6.956725688	1.717065301	-4.051520746	5.08858E-05
TRINITY_DN84238_c1_g5_i1	23.75621281	6.8460037	1.690784668	4.049009805	5.14348E-05
TRINITY_DN79770_c0_g1_i1	17.41953536	-7.755041078	1.915495	-4.048583305	5.15286E-05
TRINITY_DN78424_c2_g2_i2	271.884628	2.619559879	0.64719405	4.047564837	5.17532E-05
TRINITY_DN87158_c0_g2_i1	176.0928162	4.056822685	1.002434914	4.046968665	5.18852E-05
TRINITY_DN81814_c0_g6_i1	817.4215614	1.644824133	0.40670125	4.044305579	5.24784E-05
TRINITY_DN79004_c0_g3_i4	114.8774387	5.225532593	1.292055177	4.044357149	5.24669E-05
TRINITY_DN86769_c0_g4_i1	150.9703722	-6.433070098	1.590738126	-4.044078654	5.25293E-05
TRINITY_DN87967_c2_g2_i6	80.91512264	-6.440226376	1.592875416	-4.043145063	5.2739E-05
TRINITY_DN70904_c0_g1_i1	27.51053142	-9.090342093	2.248884628	-4.042155823	5.2962E-05

TRINITY_DN77739_c2_g1_i9	30.91232233	8.244385852	2.039945099	4.041474378	5.31162E-05
TRINITY_DN75631_c2_g1_i4	40.83339281	4.784154092	1.183830695	4.041248562	5.31674E-05
TRINITY_DN88653_c2_g2_i2	25.20744664	-7.090251305	1.755702848	-4.038411917	5.38143E-05
TRINITY_DN86704_c0_g2_i5	50.90346124	6.560020902	1.624660393	4.037779792	5.39595E-05
TRINITY_DN79304_c2_g1_i6	226.9391556	6.166835328	1.528414835	4.034791594	5.46508E-05
TRINITY_DN86592_c0_g3_i3	1274.257408	1.953712387	0.484418486	4.033108656	5.50438E-05
TRINITY_DN77787_c0_g2_i6	103.6841087	6.559332412	1.627255432	4.030917508	5.55595E-05
TRINITY_DN80085_c4_g1_i1	30.63853265	-7.383404329	1.831872808	-4.030522369	5.5653E-05
TRINITY_DN87158_c0_g1_i1	128.6499784	5.246414722	1.301674291	4.030512671	5.56553E-05
TRINITY_DN83178_c1_g1_i1	55.54552018	-8.155825614	2.023513706	-4.0305265	5.56521E-05
TRINITY_DN82161_c0_g3_i6	14.42883859	-7.958979621	1.974880307	-4.030107339	5.57514E-05
TRINITY_DN79199_c3_g2_i1	88.27406937	3.711000954	0.921455802	4.027323879	5.64153E-05
TRINITY_DN87790_c2_g1_i6	635.8170194	6.51329681	1.619579982	4.021596267	5.78051E-05
TRINITY_DN81638_c0_g2_i3	31.98298562	6.769298276	1.68349842	4.02097097	5.79587E-05
TRINITY_DN81576_c1_g2_i4	17.42779595	7.052536492	1.754047635	4.020721189	5.80202E-05
TRINITY_DN80451_c0_g2_i3	3457.118176	1.637396573	0.407357998	4.019551801	5.8309E-05
TRINITY_DN77748_c4_g1_i2	14.13113818	-7.699048934	1.91640459	-4.017444423	5.88327E-05
TRINITY_DN86865_c0_g1_i2	24.53782919	6.808221986	1.694585298	4.0176331	5.87856E-05
TRINITY_DN84276_c3_g2_i2	129.6302677	6.844011905	1.703641684	4.017283663	5.88728E-05
TRINITY_DN84519_c0_g2_i9	26.33281637	6.98603651	1.738857684	4.017601081	5.87936E-05
TRINITY_DN84718_c2_g9_i1	216.6612336	-1.598408067	0.398079589	-4.015297719	5.93708E-05
TRINITY_DN83852_c1_g3_i3	35.53543485	7.113314355	1.77267832	4.012749676	6.00155E-05
TRINITY_DN85223_c0_g1_i1	30.29050787	-7.028637245	1.75164523	-4.012591776	6.00557E-05
TRINITY_DN70521_c0_g1_i1	717.3767632	3.02836311	0.755174128	4.010152094	6.06796E-05
TRINITY_DN81043_c1_g1_i1	28.68325275	-6.68311629	1.666796607	-4.009557171	6.08327E-05
TRINITY_DN83733_c0_g1_i9	231.1455896	2.203525735	0.549867305	4.007377262	6.13967E-05
TRINITY_DN80563_c1_g1_i7	31.53784266	-8.039283141	2.006067196	-4.007484473	6.13689E-05
TRINITY_DN84245_c1_g1_i3	77.6754678	-6.43526049	1.606169942	-4.006587548	6.16023E-05
TRINITY_DN83365_c0_g2_i8	20.14665096	7.558722548	1.886701379	4.006316331	6.1673E-05
TRINITY_DN83865_c0_g2_i1	1857.597844	2.442587541	0.610080854	4.003711191	6.23565E-05
TRINITY_DN84826_c0_g1_i2	19.81141399	-7.221569597	1.803751405	-4.00363907	6.23755E-05
TRINITY_DN82226_c0_g1_i7	28.17722465	-8.590142694	2.146696632	-4.00156341	6.29253E-05
TRINITY_DN85840_c2_g3_i5	26.31774975	-6.818769477	1.70420048	-4.001154534	6.30342E-05
TRINITY_DN80052_c0_g2_i1	48.59817162	6.559737809	1.639697646	4.000577683	6.3188E-05
TRINITY_DN83541_c0_g1_i6	28.74215319	9.189007239	2.298311139	3.998156334	6.38378E-05
TRINITY_DN84776_c3_g1_i1	22.15582428	-7.075750772	1.770779394	-3.995839795	6.44653E-05
TRINITY_DN87124_c0_g1_i8	28.57843803	-6.968366052	1.743968101	-3.99569582	6.45045E-05
TRINITY_DN84298_c0_g1_i1	23.55922549	-7.28450867	1.823514727	-3.994762731	6.47591E-05
TRINITY_DN82258_c0_g6_i1	103.1438804	7.081863124	1.773083038	3.994095581	6.49417E-05
TRINITY_DN87266_c4_g1_i9	64.54626988	-4.289565642	1.075002837	-3.990283091	6.59945E-05
TRINITY_DN85620_c2_g5_i3	36.55415612	-7.06851762	1.772683199	-3.987468051	6.67822E-05
TRINITY_DN81979_c0_g1_i7	67.52120337	5.41097223	1.357572989	3.985768923	6.72619E-05
TRINITY_DN86517_c0_g1_i1	31.1514502	-6.711443753	1.684531344	-3.984160803	6.7719E-05
TRINITY_DN81752_c0_g1_i2	22.41515089	-6.921323434	1.73760058	-3.983264918	6.79749E-05
TRINITY_DN80451_c0_g2_i5	119.7713333	2.707154875	0.679767273	3.982473092	6.82018E-05
TRINITY_DN86993_c1_g12_i	10704.54461	1.979889073	0.497192763	3.982135741	6.82988E-05
TRINITY_DN87754_c1_g1_i9	750.2680013	2.609133017	0.655684371	3.9792515	6.91326E-05
TRINITY_DN88655_c1_g1_i2	21.01775041	-7.726590107	1.943718516	-3.975158976	7.03323E-05
TRINITY_DN85389_c0_g1_i2	18.36822445	-7.778363759	1.956678649	-3.975289332	7.02937E-05
TRINITY_DN87128_c2_g1_i1	51.22371762	-7.107537346	1.787978803	-3.975179871	7.03261E-05
TRINITY_DN88274_c1_g3_i1	22.46519559	7.204656754	1.812757505	3.974418384	7.05514E-05
TRINITY_DN81168_c0_g1_i1	165.8081699	-1.870615681	0.470805791	-3.973221482	7.09071E-05
TRINITY_DN81875_c1_g1_i1	585.3477007	2.082157578	0.524188293	3.972155819	7.12251E-05
TRINITY_DN80601_c0_g1_i6	16.88711817	-7.965443499	2.006933421	-3.968962505	7.21862E-05
TRINITY_DN86692_c1_g3_i1	14.06845533	-7.425194705	1.871797038	-3.966880252	7.28196E-05
TRINITY_DN77339_c2_g2_i3	18.35782471	7.862200519	1.982257767	3.966285641	7.30014E-05
TRINITY_DN84307_c1_g1_i9	147.6370331	-9.365546036	2.362098641	-3.964925881	7.34188E-05

TRINITY_DN82627_c0_g1_i6	353.618653	2.101087977	0.530373673	3.961523893	7.44729E-05
TRINITY_DN84773_c1_g1_i3	18.12723835	-8.176794886	2.064921472	-3.959857553	7.49945E-05
TRINITY_DN85330_c1_g1_i1	21.95135576	7.342826029	1.854925279	3.958556236	7.54042E-05
TRINITY_DN77966_c3_g1_i9	40.48063935	6.881723159	1.739286226	3.956636381	7.60125E-05
TRINITY_DN70058_c0_g1_i3	72.72557509	3.036120895	0.767424352	3.956247789	7.61362E-05
TRINITY_DN85610_c1_g1_i1	21.19235298	-7.385486594	1.867722901	-3.95427319	7.67677E-05
TRINITY_DN87559_c2_g1_i5	20.75559558	6.917421854	1.749421527	3.954119547	7.6817E-05
TRINITY_DN84434_c0_g4_i2	3739.991758	1.80777078	0.457142301	3.95450339	7.66938E-05
TRINITY_DN84122_c2_g1_i3	1235.39153	1.778655483	0.449774169	3.954552323	7.66781E-05
TRINITY_DN76893_c0_g1_i1	65.21318339	2.565047932	0.648440107	3.955720668	7.63043E-05
TRINITY_DN86416_c0_g1_i1	63.34753451	3.902583291	0.986762995	3.954934782	7.65555E-05
TRINITY_DN85397_c0_g1_i6	25.52417161	7.116122379	1.801306559	3.950533763	7.79771E-05
TRINITY_DN79563_c0_g1_i5	22.56963024	-7.208742764	1.825235197	-3.949487044	7.83188E-05
TRINITY_DN84682_c1_g2_i3	24.48002217	-7.574689751	1.918409623	-3.948421473	7.86682E-05
TRINITY_DN86126_c1_g3_i4	2401.742083	1.529219316	0.387355171	3.947847943	7.88569E-05
TRINITY_DN80005_c1_g1_i2	19.56542802	-6.994960533	1.772730758	-3.945867414	7.95116E-05
TRINITY_DN83317_c2_g2_i2	111.3727336	-6.424682513	1.628756539	-3.944532138	7.99559E-05
TRINITY_DN80259_c2_g6_i2	46.40093054	6.213528751	1.575615055	3.943557616	8.02817E-05
TRINITY_DN88193_c1_g1_i1	20.33874318	-7.142180971	1.81101994	-3.943734032	8.02226E-05
TRINITY_DN86586_c1_g1_i2	19.51839582	-7.372043428	1.869734947	-3.942828067	8.05264E-05
TRINITY_DN86868_c2_g2_i3	47.90692675	2.756043551	0.699198137	3.941720389	8.08993E-05
TRINITY_DN88288_c4_g1_i2	16.52909258	6.986266801	1.772635338	3.941175407	8.10833E-05
TRINITY_DN88686_c6_g6_i2	256.4848282	6.204356396	1.574582794	3.940317664	8.13738E-05
TRINITY_DN81213_c3_g1_i3	367.6222454	1.679142092	0.4262228	3.939587678	8.16218E-05
TRINITY_DN82403_c1_g1_i4	17.27239523	-7.158979174	1.817068212	-3.93985164	8.1532E-05
TRINITY_DN84676_c2_g1_i2	18.53722116	7.393130688	1.877687533	3.937359416	8.23832E-05
TRINITY_DN88391_c0_g2_i2	28.76548751	-6.645199627	1.688239731	-3.936170619	8.27921E-05
TRINITY_DN80486_c1_g3_i3	35.04162833	6.648168113	1.689114127	3.935890421	8.28888E-05
TRINITY_DN81963_c3_g1_i1	31.88032523	-7.336959375	1.864898487	-3.934240619	8.34601E-05
TRINITY_DN86597_c0_g1_i1	36.49007524	-6.487769918	1.64937249	-3.933477706	8.37256E-05
TRINITY_DN84546_c1_g1_i9	182.1932132	-2.125530384	0.540151794	-3.93506123	8.31755E-05
TRINITY_DN82269_c0_g2_i9	15.70793284	7.444396357	1.892059783	3.934546056	8.33541E-05
TRINITY_DN81694_c0_g1_i5	8754.675766	1.741925382	0.442689085	3.934873123	8.32407E-05
TRINITY_DN80969_c0_g1_i5	225.813162	2.877885952	0.731638088	3.933482961	8.37238E-05
TRINITY_DN84343_c0_g1_i7	70.02760507	-7.031933129	1.78766497	-3.933585569	8.3688E-05
TRINITY_DN88804_c7_g4_i1	3040.547021	1.945851581	0.495016944	3.930878743	8.4636E-05
TRINITY_DN85716_c1_g3_i5	20.14358331	-8.437952302	2.147000494	-3.930111952	8.49063E-05
TRINITY_DN87880_c1_g5_i3	1902.217756	2.273917076	0.578533002	3.930488093	8.47736E-05
TRINITY_DN78098_c3_g2_i4	34.7214313	6.729415925	1.712354083	3.929920799	8.49738E-05
TRINITY_DN83010_c0_g1_i1	120.7651089	7.085935854	1.803341017	3.929337705	8.51801E-05
TRINITY_DN79168_c1_g1_i5	39.57162592	6.560006599	1.670421763	3.927155851	8.59563E-05
TRINITY_DN85407_c1_g5_i1	14.81614826	7.114718539	1.811625668	3.927256423	8.59204E-05
TRINITY_DN82406_c1_g2_i3	501.7824772	4.662931508	1.187736319	3.925897889	8.64068E-05
TRINITY_DN84118_c1_g1_i3	60.36479054	-6.439856079	1.640636306	-3.92521856	8.6651E-05
TRINITY_DN77319_c0_g5_i3	161.4016465	-18.84007426	4.800283798	-3.924783419	8.68078E-05
TRINITY_DN83552_c0_g1_i1	497.6183813	2.215778318	0.565167732	3.920567637	8.83406E-05
TRINITY_DN85299_c1_g1_i1	33.54935142	7.825704894	1.996818374	3.919086981	8.8885E-05
TRINITY_DN79432_c0_g1_i6	12.64221578	-7.306513146	1.864515686	-3.918719054	8.90208E-05
TRINITY_DN87493_c1_g4_i8	23.50638593	8.457173335	2.158468529	3.91813604	8.92363E-05
TRINITY_DN79494_c1_g1_i2	1621.024298	1.502282532	0.38346187	3.91768426	8.94037E-05
TRINITY_DN88119_c1_g2_i1	27.1213326	-7.002361825	1.788081905	-3.916130355	8.99816E-05
TRINITY_DN77494_c2_g1_i7	23.21440388	-7.093131094	1.811474716	-3.915666628	9.01548E-05
TRINITY_DN78219_c0_g1_i4	12.17679198	8.113853834	2.073689475	3.912762219	9.12464E-05
TRINITY_DN81548_c3_g1_i2	58.15795684	6.768490566	1.73080468	3.910603342	9.20659E-05
TRINITY_DN84956_c5_g4_i3	42.2628446	-6.72029588	1.718980053	-3.909467051	9.25E-05
TRINITY_DN84037_c1_g1_i3	62.78929852	7.466112635	1.909995183	3.908969353	9.26907E-05
TRINITY_DN83652_c1_g2_i8	22.55713959	-7.010436692	1.793650927	-3.908473264	9.28812E-05

TRINITY_DN87474_c1_g2_i4	239.3488002	3.308323803	0.846979583	3.906025447	9.38266E-05
TRINITY_DN85389_c0_g1_i2	20.57675359	-7.286705138	1.86591171	-3.905171451	9.41586E-05
TRINITY_DN83892_c1_g2_i1	16.11660356	7.417092793	1.899845558	3.904050391	9.45961E-05
TRINITY_DN85050_c0_g1_i1	33.92592087	-6.893151567	1.767018247	-3.901007575	9.57932E-05
TRINITY_DN82395_c2_g9_i2	217.4763739	3.681722631	0.943839029	3.90079507	9.58773E-05
TRINITY_DN73379_c0_g1_i1	138.9852622	2.315470799	0.593813524	3.899323113	9.6462E-05
TRINITY_DN83649_c0_g4_i1	145.4209728	2.610370245	0.66966752	3.898009335	9.69867E-05
TRINITY_DN82842_c2_g1_i8	155.6409174	-9.015630465	2.312577632	-3.898520136	9.67824E-05
TRINITY_DN78569_c0_g4_i1	122.3659602	2.810920538	0.720992001	3.898684779	9.67166E-05
TRINITY_DN81957_c4_g1_i3	1977.53333	1.946810158	0.499443014	3.897962536	9.70054E-05
TRINITY_DN81932_c0_g1_i2	61.68143905	6.883701209	1.766290413	3.897264661	9.72853E-05
TRINITY_DN80007_c0_g5_i3	21.42979424	-6.975583822	1.790055463	-3.896853458	9.74505E-05
TRINITY_DN83752_c1_g4_i1	101.3670342	-9.569644862	2.45634542	-3.895887274	9.78399E-05
TRINITY_DN78060_c0_g2_i1	51.38767232	6.807178089	1.748541339	3.893060997	9.89873E-05
TRINITY_DN85792_c1_g4_i2	37.51176883	-7.241158209	1.860170179	-3.892739649	9.91185E-05
TRINITY_DN80170_c0_g1_i1	211.3841012	-6.404408605	1.645778428	-3.891416061	9.96609E-05
TRINITY_DN86793_c2_g1_i6	53.89488503	7.017868018	1.803593422	3.891047689	9.98123E-05
TRINITY_DN87272_c0_g1_i3	151.8765028	5.29736661	1.361634749	3.890446108	0.00010006
TRINITY_DN78533_c0_g1_i1	44.25155001	6.916310657	1.777651962	3.890700094	9.99554E-05
TRINITY_DN85931_c1_g1_i1	183.3555527	8.833623015	2.271048158	3.889667854	0.000100382
TRINITY_DN79780_c7_g1_i8	57.79886003	6.603329579	1.698452226	3.887851232	0.000101136
TRINITY_DN88729_c3_g2_i1	204.8690316	3.979317574	1.023541461	3.88779324	0.00010116
TRINITY_DN84224_c1_g2_i1	44.1867203	-7.594018841	1.954208624	-3.885981644	0.000101917
TRINITY_DN84746_c0_g2_i6	42.61696941	3.502240299	0.901409907	3.885291554	0.000102207
TRINITY_DN87041_c0_g2_i1	81.06030228	6.558981476	1.688331805	3.884888892	0.000102377
TRINITY_DN83320_c0_g3_i5	31.84955543	-7.227200828	1.861043549	-3.883413062	0.000103
TRINITY_DN79849_c0_g3_i9	18.40260265	-7.141497468	1.840591163	-3.880002041	0.000104456
TRINITY_DN85921_c0_g1_i8	23.66990802	6.604474806	1.702115547	3.88015656	0.000104389
TRINITY_DN77572_c2_g3_i1	124.5455922	2.113424922	0.544840911	3.878976192	0.000104897
TRINITY_DN86652_c1_g1_i1	60.87614288	6.46793067	1.668308125	3.876940101	0.000105778
TRINITY_DN81753_c0_g1_i6	57.73871279	-2.936684391	0.757737324	-3.875596856	0.000106364
TRINITY_DN87627_c3_g1_i1	12.33246352	7.174739655	1.851116017	3.875899505	0.000106232
TRINITY_DN84821_c2_g2_i1	40.54561758	-6.467865155	1.668559953	-3.876315707	0.00010605
TRINITY_DN88846_c3_g2_i1	82.3985405	-18.61535122	4.80300876	-3.875768743	0.000106289
TRINITY_DN79268_c3_g1_i7	45.20608818	-4.704776324	1.214723765	-3.873124457	0.000107449
TRINITY_DN78433_c1_g1_i4	100.0837358	7.230916031	1.867193756	3.87261151	0.000107675
TRINITY_DN79434_c1_g2_i3	64.72854478	6.366718202	1.644510158	3.871498253	0.000108168
TRINITY_DN80711_c0_g1_i5	71.44564695	-2.978007877	0.769692354	-3.869088555	0.000109243
TRINITY_DN86435_c0_g1_i9	10675.64982	1.80563125	0.46680923	3.868028164	0.000109719
TRINITY_DN84797_c0_g2_i1	23.5188001	8.066554066	2.08609796	3.866814609	0.000110266
TRINITY_DN85855_c0_g3_i2	26.63861567	-9.244724477	2.390631273	-3.867064144	0.000110153
TRINITY_DN84672_c0_g1_i9	55.61675167	-6.507553828	1.683133442	-3.866332678	0.000110484
TRINITY_DN87951_c0_g1_i2	27.68083484	-6.774554554	1.75304483	-3.864450263	0.00011134
TRINITY_DN79382_c0_g2_i1	438.7849356	2.931485647	0.758730328	3.863672688	0.000111695
TRINITY_DN78762_c2_g1_i2	157.9124223	6.465662029	1.673794183	3.862877584	0.000112059
TRINITY_DN79668_c0_g1_i6	17.65457984	-7.395161752	1.915500512	-3.860694217	0.000113065
TRINITY_DN86515_c0_g2_i1	19.92219824	6.917536458	1.791824518	3.86061045	0.000113104
TRINITY_DN85644_c8_g4_i1	954.4018492	2.271092436	0.588491317	3.859177477	0.000113769
TRINITY_DN76141_c0_g1_i5	50.80644362	-8.972481336	2.325374438	-3.858510349	0.00011408
TRINITY_DN78743_c2_g4_i9	20.86265972	-7.096556867	1.839489109	-3.857895559	0.000114367
TRINITY_DN84904_c2_g2_i6	545.6657894	3.306171029	0.857304837	3.856470753	0.000115036
TRINITY_DN77181_c0_g3_i1	35.26084493	-7.060230274	1.83098878	-3.855965887	0.000115274
TRINITY_DN82606_c0_g3_i4	227.8500805	1.532043746	0.397611619	3.853116144	0.000116624
TRINITY_DN87790_c2_g1_i4	323.3227629	3.385284868	0.87882894	3.852040726	0.000117138
TRINITY_DN79332_c1_g3_i1	38.39344347	-7.332060232	1.903364964	-3.852156771	0.000117082
TRINITY_DN80388_c3_g5_i4	13.23008977	-8.146190019	2.114804783	-3.851982029	0.000117166
TRINITY_DN87464_c1_g1_i1	439.0576749	8.415492968	2.185284348	3.850983043	0.000117645

TRINITY_DN80642_c1_g1_i3	33.80681111	7.143632099	1.855111409	3.850783336	0.000117741
TRINITY_DN87774_c0_g3_i9	140.843913	-2.167709727	0.562918462	-3.850841415	0.000117713
TRINITY_DN86628_c1_g1_i1	12.91540993	-7.043250757	1.829948367	-3.848879501	0.000118659
TRINITY_DN86778_c0_g1_i3	42.24074069	7.01901936	1.823664912	3.848853653	0.000118672
TRINITY_DN85196_c1_g1_i1	13.21040342	-7.13640456	1.85393023	-3.849338256	0.000118437
TRINITY_DN87367_c0_g3_i6	43.34611297	9.686931623	2.517409221	3.84797654	0.000119097
TRINITY_DN76939_c1_g4_i2	553.570124	-2.556787384	0.664672058	-3.846690036	0.000119724
TRINITY_DN76464_c2_g2_i5	101.9483144	2.30655963	0.599611106	3.846759353	0.00011969
TRINITY_DN78433_c1_g1_i5	173.6768442	3.820493871	0.99334716	3.846081233	0.000120022
TRINITY_DN85422_c0_g1_i9	17.64227736	7.627915627	1.983454633	3.845772674	0.000120173
TRINITY_DN87521_c1_g4_i3	13.22950628	7.145468978	1.858299702	3.845165002	0.000120471
TRINITY_DN82955_c2_g4_i1	31.36512951	-6.716613754	1.747537773	-3.843472718	0.000121305
TRINITY_DN83128_c2_g2_i2	26.52849363	-3.992274603	1.038693373	-3.843554513	0.000121265
TRINITY_DN86779_c1_g2_i5	19.57495273	8.749612566	2.277356911	3.842003213	0.000122034
TRINITY_DN81113_c1_g2_i4	20.84383723	-7.386145955	1.922477536	-3.841993374	0.000122039
TRINITY_DN81817_c3_g1_i4	82.697743	6.368583729	1.657671443	3.841885408	0.000122093
TRINITY_DN80993_c0_g1_i3	13.90055058	-7.750974749	2.017472278	-3.841923795	0.000122074
TRINITY_DN84622_c1_g6_i3	38.85518484	7.08306186	1.843575518	3.842024256	0.000122024
TRINITY_DN84673_c0_g3_i1	205.1167198	6.019718527	1.567293351	3.84083715	0.000122615
TRINITY_DN81990_c1_g2_i1	19.71162975	-8.483522668	2.208713029	-3.840934769	0.000122567
TRINITY_DN79409_c2_g2_i5	50.99866515	6.605762939	1.720259431	3.839980656	0.000123044
TRINITY_DN85386_c1_g1_i4	11.86092623	7.394424114	1.92548158	3.840298547	0.000122885
TRINITY_DN87384_c1_g3_i2	159.7158843	2.159693643	0.562396203	3.840163986	0.000122952
TRINITY_DN77356_c0_g1_i1	25.85971439	-8.929890027	2.325935502	-3.839268123	0.000123402
TRINITY_DN78060_c0_g2_i1	47.49236213	-3.131491561	0.815913591	-3.83801863	0.000124031
TRINITY_DN85677_c0_g1_i4	77.32314413	-6.078695429	1.58500466	-3.835127796	0.000125499
TRINITY_DN87419_c0_g2_i3	12.30098431	-7.193218583	1.876214288	-3.833900332	0.000126127
TRINITY_DN81469_c0_g1_i6	18.80021925	7.231435825	1.886043875	3.834182186	0.000125983
TRINITY_DN82207_c3_g2_i7	266.8242101	6.512747419	1.698767725	3.833806896	0.000126175
TRINITY_DN79493_c0_g1_i4	72.44920176	-3.046791788	0.794912309	-3.83286528	0.000126659
TRINITY_DN82931_c0_g1_i2	22.83017297	7.175575389	1.872367232	3.832354714	0.000126923
TRINITY_DN88062_c1_g4_i4	1181.089619	1.436454616	0.374880194	3.831769827	0.000127225
TRINITY_DN81346_c1_g6_i1	48.99226159	6.688110747	1.745728902	3.831127927	0.000127557
TRINITY_DN85262_c4_g2_i1	2207.050037	2.708853469	0.707449314	3.829042473	0.000128643
TRINITY_DN87949_c2_g3_i1	628.8210011	-1.782499251	0.46561762	-3.828246989	0.000129059
TRINITY_DN86806_c2_g2_i8	4958.436184	1.55781155	0.406974197	3.82778948	0.000129299
TRINITY_DN75260_c0_g1_i1	19.72768974	7.231944713	1.889590808	3.827254388	0.000129581
TRINITY_DN82474_c0_g1_i1	48.79256905	6.558960876	1.713780247	3.82718898	0.000129615
TRINITY_DN82570_c0_g2_i1	1200.407742	2.451581206	0.640636124	3.826792017	0.000129824
TRINITY_DN84501_c3_g1_i4	16.08179423	-6.979527324	1.824004023	-3.826486804	0.000129985
TRINITY_DN83431_c1_g1_i2	18.01383509	6.844732912	1.789641755	3.824638587	0.000130964
TRINITY_DN86296_c1_g1_i1	38.63212758	4.099599789	1.072203456	3.823527863	0.000131556
TRINITY_DN82541_c0_g1_i4	56.14371179	-6.250549988	1.634902717	-3.823193834	0.000131734
TRINITY_DN78377_c1_g3_i2	27.82582915	-7.472956589	1.955153963	-3.822183179	0.000132275
TRINITY_DN77433_c0_g1_i1	57.26349678	7.646709899	2.000769975	3.821883571	0.000132436
TRINITY_DN84345_c1_g2_i5	20.60675474	-8.855080793	2.317474133	-3.821005235	0.000132909
TRINITY_DN83290_c4_g1_i5	31.05833385	7.084627422	1.854623961	3.81998053	0.000133462
TRINITY_DN86972_c0_g2_i1	14.79323969	8.083375181	2.116258301	3.819654329	0.000133639
TRINITY_DN84503_c1_g1_i1	53.43682834	-6.305995589	1.651361976	-3.818663431	0.000134177
TRINITY_DN78103_c0_g4_i5	31.32361038	6.417883995	1.681424935	3.816931616	0.000135122
TRINITY_DN87388_c3_g1_i1	227.1535665	-16.70789807	4.377829862	-3.816479533	0.000135369
TRINITY_DN86435_c0_g2_i3	36.14653196	6.95151431	1.821559571	3.816243191	0.000135499
TRINITY_DN78964_c0_g5_i2	1598.807414	1.247126432	0.326843424	3.815669346	0.000135814
TRINITY_DN83714_c0_g8_i1	95.05685026	6.367005341	1.669788465	3.813061039	0.000137256
TRINITY_DN86172_c1_g1_i1	26.24610504	-6.855889429	1.798416569	-3.812180976	0.000137746
TRINITY_DN88128_c0_g1_i2	89.61232606	6.465790138	1.69658263	3.811067037	0.000138368
TRINITY_DN77150_c0_g1_i3	157.2534435	-4.104773622	1.077027379	-3.811206382	0.00013829

TRINITY_DN86495_c2_g4_i1	22.02958762	6.844472173	1.795975187	3.811005977	0.000138402
TRINITY_DN84643_c3_g1_i3	1032.327874	1.268377954	0.332920834	3.809848541	0.000139052
TRINITY_DN87586_c1_g3_i3	131.7228992	6.01924911	1.580245453	3.809059598	0.000139496
TRINITY_DN88417_c2_g1_i1	142.9046427	6.880186715	1.806913396	3.807701426	0.000140264
TRINITY_DN80911_c0_g2_i3	23.74808676	-6.917563739	1.816783615	-3.807588137	0.000140329
TRINITY_DN79175_c3_g1_i4	12892.18586	1.275595319	0.33504155	3.807275011	0.000140506
TRINITY_DN82498_c1_g1_i3	21.07978245	-7.375930772	1.937579257	-3.806776289	0.00014079
TRINITY_DN82902_c2_g2_i1	11.09419185	-7.64595882	2.008835466	-3.80616479	0.000141138
TRINITY_DN80531_c3_g1_i6	24.78564749	-9.140567009	2.401746944	-3.805799371	0.000141347
TRINITY_DN80648_c1_g1_i1	185.2469532	2.77866833	0.730225588	3.805219066	0.000141679
TRINITY_DN77069_c2_g1_i7	29.25442695	-7.673677718	2.016501185	-3.80544171	0.000141551
TRINITY_DN88046_c1_g1_i2	26.44597113	-7.395222404	1.944599133	-3.802954697	0.000142981
TRINITY_DN86076_c1_g5_i1	26.9833281	-7.354302119	1.933752257	-3.803125292	0.000142882
TRINITY_DN78604_c0_g1_i5	38.06649765	5.788518255	1.522723651	3.801424014	0.000143867
TRINITY_DN85206_c0_g1_i6	16.11568288	6.807958193	1.790952849	3.801305096	0.000143936
TRINITY_DN85432_c0_g1_i1	13.12003424	-7.108667585	1.869848931	-3.801733642	0.000143687
TRINITY_DN87166_c2_g1_i4	8337.598492	1.374408248	0.361804763	3.79875665	0.000145424
TRINITY_DN83343_c0_g2_i4	16.16591819	7.052199268	1.856392758	3.798872431	0.000145356
TRINITY_DN86487_c3_g1_i1	17.3849204	-8.509290359	2.240777239	-3.797472685	0.000146179
TRINITY_DN86910_c0_g2_i1	41.09905974	3.359462402	0.885132862	3.795432919	0.000147386
TRINITY_DN82195_c0_g1_i2	25.72518138	-8.567854498	2.25771161	-3.794928662	0.000147686
TRINITY_DN87465_c0_g5_i4	89.26721185	8.949811958	2.35857348	3.794586869	0.000147889
TRINITY_DN80713_c0_g2_i1	2136.949644	1.439533502	0.379410072	3.794136237	0.000148158
TRINITY_DN81280_c0_g1_i1	22.47097373	-7.016261554	1.849558137	-3.793479867	0.000148551
TRINITY_DN79131_c3_g1_i1	32.76862455	6.809789345	1.795159994	3.793416389	0.000148589
TRINITY_DN86467_c2_g2_i6	11.31983044	-7.505068324	1.979187515	-3.791994576	0.000149442
TRINITY_DN81151_c0_g1_i1	10.01632281	-7.41300977	1.955311583	-3.791216619	0.000149911
TRINITY_DN87261_c0_g1_i3	19.92101057	7.26012029	1.915100904	3.790985777	0.000150051
TRINITY_DN81031_c0_g5_i4	23.21823326	7.314197425	1.929710088	3.790308953	0.00015046
TRINITY_DN86733_c0_g2_i1	175.019955	1.761097568	0.464780057	3.789098822	0.000151195
TRINITY_DN82663_c1_g1_i5	53.08119967	6.466403515	1.706667327	3.788906844	0.000151312
TRINITY_DN87152_c2_g2_i3	23.62165458	6.559846131	1.731618184	3.788275146	0.000151697
TRINITY_DN75918_c0_g1_i4	49.93562643	9.099448472	2.402967364	3.786754914	0.000152628
TRINITY_DN87241_c2_g1_i1	23.1637161	6.952603978	1.836053504	3.786710988	0.000152654
TRINITY_DN84431_c1_g8_i1	374.8636263	3.375455391	0.891332776	3.786975506	0.000152492
TRINITY_DN78409_c1_g7_i2	33.98229121	4.104485808	1.0839545	3.78658496	0.000152732
TRINITY_DN88648_c2_g1_i4	48.73955986	6.315749994	1.668071092	3.786259484	0.000152932
TRINITY_DN81945_c1_g1_i1	986.8583989	1.432524498	0.378444304	3.785298081	0.000153524
TRINITY_DN79318_c3_g1_i4	24.61256484	-6.905771696	1.824981527	-3.784022794	0.000154314
TRINITY_DN85220_c3_g1_i5	14.87404712	-7.086925838	1.873745623	-3.782224093	0.000155433
TRINITY_DN84091_c0_g4_i1	20.77992805	6.769407968	1.789814812	3.782183453	0.000155459
TRINITY_DN84272_c1_g1_i1	101.1330524	8.884878774	2.348261052	3.783599258	0.000154577
TRINITY_DN84646_c3_g1_i1	25.27102383	6.418512721	1.696896266	3.782501528	0.00015526
TRINITY_DN87100_c0_g2_i1	15.3952074	-7.310845208	1.933118677	-3.781891559	0.000155641
TRINITY_DN82702_c2_g1_i7	21.9639738	-7.303816636	1.930533545	-3.78331506	0.000154753
TRINITY_DN80862_c0_g1_i8	21.81966615	-8.700040721	2.300088593	-3.782480705	0.000155273
TRINITY_DN75654_c0_g2_i1	12.80684002	-6.97217519	1.843871705	-3.781269146	0.000156031
TRINITY_DN85388_c4_g4_i9	419.9402591	6.843410228	1.809769424	3.781371339	0.000155967
TRINITY_DN72196_c0_g1_i2	16.25742835	-7.31464602	1.934371908	-3.781406249	0.000155945
TRINITY_DN82873_c1_g2_i1	30.77905054	-7.635132709	2.019547749	-3.780615097	0.000156441
TRINITY_DN88062_c1_g4_i3	191.0719238	2.328431485	0.616280689	3.778199653	0.000157966
TRINITY_DN75952_c0_g1_i1	272.531125	1.881699094	0.498122613	3.777582156	0.000158358
TRINITY_DN85836_c0_g1_i4	22.02755021	-5.323901719	1.409433532	-3.777334369	0.000158516
TRINITY_DN84952_c3_g1_i1	23.78367503	6.418513596	1.699699734	3.776263224	0.000159199
TRINITY_DN85294_c1_g1_i2	131.3659239	6.020378656	1.594254105	3.776298043	0.000159177
TRINITY_DN86799_c0_g1_i1	50.89031472	3.174005167	0.840670685	3.775563039	0.000159647
TRINITY_DN81631_c1_g1_i9	2222.623825	1.329764983	0.352309075	3.774427276	0.000160376

TRINITY_DN78887_c2_g2_i3	87.21113624	6.218442194	1.64795561	3.773428215	0.00016102
TRINITY_DN88175_c6_g1_i4	23.72765819	-6.993826635	1.853818929	-3.772658982	0.000161517
TRINITY_DN83393_c0_g1_i5	227.5451114	2.370161489	0.628217357	3.772836683	0.000161402
TRINITY_DN86189_c0_g1_i4	307.8470693	1.699613314	0.4505773	3.772079318	0.000161893
TRINITY_DN80321_c0_g2_i5	18.02991236	-6.885229409	1.825530737	-3.771631597	0.000162184
TRINITY_DN87990_c0_g1_i2	70.00210797	6.316408577	1.675332687	3.770241354	0.00016309
TRINITY_DN78372_c0_g2_i1	14.11961739	6.808089601	1.805946614	3.769817751	0.000163367
TRINITY_DN81842_c2_g1_i4	1247.829054	2.741307068	0.727245695	3.769437328	0.000163616
TRINITY_DN87333_c0_g3_i1	15.92972057	-6.715906034	1.781873764	-3.769013367	0.000163894
TRINITY_DN78996_c1_g5_i3	1848.082423	2.263959726	0.600962139	3.767225219	0.000165072
TRINITY_DN85830_c0_g1_i3	61.92822451	-6.015633796	1.596998099	-3.766838419	0.000165328
TRINITY_DN86719_c0_g4_i6	34.66822661	6.918144851	1.836673667	3.76667068	0.000165439
TRINITY_DN87158_c0_g1_i2	43.95616518	6.559238598	1.742807528	3.763604697	0.000167482
TRINITY_DN86259_c1_g5_i1	72.85652226	5.257783268	1.397208138	3.76306373	0.000167844
TRINITY_DN84885_c0_g2_i9	68.72317652	-9.015253125	2.397267198	-3.760637584	0.000169481
TRINITY_DN77155_c2_g2_i4	22.34596747	6.807028733	1.809989097	3.760812009	0.000169363
TRINITY_DN80087_c1_g2_i1	892.0010397	1.619449554	0.430651914	3.760460596	0.000169601
TRINITY_DN80028_c1_g1_i8	241.1136093	-1.845727632	0.490951349	-3.759491925	0.000170259
TRINITY_DN85288_c1_g5_i2	1382.354674	1.462424062	0.389025374	3.759199678	0.000170458
TRINITY_DN78013_c1_g1_i1	16.67851061	-7.538003661	2.006085261	-3.757568936	0.000171572
TRINITY_DN84303_c2_g1_i9	12.65949536	-7.02500935	1.869543545	-3.757606701	0.000171546
TRINITY_DN79487_c1_g1_i3	15.17889477	8.329442514	2.21632626	3.758220377	0.000171126
TRINITY_DN87118_c2_g3_i1	124.6194631	2.58602444	0.688123274	3.75808309	0.00017122
TRINITY_DN82261_c2_g2_i2	19.75511955	7.491840763	1.993917099	3.75734817	0.000171723
TRINITY_DN86430_c0_g2_i2	154.1749216	2.914334522	0.775370502	3.75863476	0.000170843
TRINITY_DN79432_c0_g1_i1	14.89189282	7.204560095	1.916838717	3.75856353	0.000170892
TRINITY_DN84407_c3_g1_i1	183.9981321	6.084102607	1.619735482	3.75623222	0.000172491
TRINITY_DN85546_c2_g1_i7	955.3347157	1.674180059	0.445699727	3.756295904	0.000172447
TRINITY_DN87445_c0_g2_i7	52.83746413	-3.834345977	1.021477837	-3.753724102	0.000174227
TRINITY_DN88483_c0_g2_i2	498.1523047	1.49680148	0.398731599	3.753907348	0.000174099
TRINITY_DN85253_c2_g2_i1	702.2977078	4.118768344	1.097323373	3.75346816	0.000174405
TRINITY_DN83180_c0_g2_i2	41.57386212	5.717324201	1.523685276	3.75229996	0.00017522
TRINITY_DN79400_c3_g9_i6	34.36985543	-6.390993155	1.703147676	-3.75245978	0.000175108
TRINITY_DN85830_c0_g1_i2	137.314664	-7.079618708	1.886987937	-3.751809203	0.000175563
TRINITY_DN86147_c0_g1_i1	45.76805306	-9.888583093	2.636869218	-3.750122692	0.000176748
TRINITY_DN84832_c0_g8_i2	20.99097452	6.690067336	1.785557322	3.746767048	0.000179128
TRINITY_DN87937_c2_g2_i2	19.88183519	-7.499941268	2.002126772	-3.745987204	0.000179686
TRINITY_DN78488_c2_g4_i2	2395.013882	1.2754035	0.34053164	3.745330386	0.000180157
TRINITY_DN78128_c1_g1_i7	614.632287	1.247828026	0.333347823	3.74332136	0.000181604
TRINITY_DN81039_c1_g4_i2	347.0815848	-5.683883334	1.51868729	-3.74262916	0.000182105
TRINITY_DN84798_c1_g1_i9	26.67677681	6.561344766	1.753157444	3.74258729	0.000182135
TRINITY_DN86143_c0_g1_i4	17.935329	7.339966121	1.960560886	3.743809322	0.000181251
TRINITY_DN84571_c0_g2_i2	22.36228603	-7.806491032	2.085494289	-3.743232995	0.000181668
TRINITY_DN83814_c1_g2_i1	32.95978124	3.811256954	1.018348	3.742587949	0.000182135
TRINITY_DN88630_c5_g1_i4	7476.697198	1.441990575	0.385342983	3.742096366	0.000182492
TRINITY_DN85185_c0_g1_i2	1575.220403	4.401972489	1.176419237	3.741839942	0.000182678
TRINITY_DN80989_c1_g2_i3	3384.414511	1.698747094	0.454320957	3.73909032	0.000184687
TRINITY_DN82179_c0_g2_i1	26.41669484	-7.310355844	1.95468011	-3.739924404	0.000184076
TRINITY_DN83219_c1_g1_i6	36.10463139	-4.593902078	1.228669226	-3.738924994	0.000184809
TRINITY_DN86530_c0_g2_i2	28.49424666	9.459422942	2.529512597	3.739622785	0.000184297
TRINITY_DN78592_c1_g1_i1	18.05138787	-7.45515494	1.993912529	-3.738957868	0.000184785
TRINITY_DN86227_c0_g2_i4	68.34487936	-6.274331325	1.677863939	-3.739475639	0.000184405
TRINITY_DN84461_c3_g2_i1	26.50271934	7.627673373	2.03940924	3.740138674	0.000183919
TRINITY_DN86166_c1_g3_i1	155.6872678	4.561818801	1.220235434	3.738474291	0.00018514
TRINITY_DN79244_c2_g7_i7	47.85554576	-7.517330718	2.011233502	-3.737671788	0.000185732
TRINITY_DN87969_c1_g3_i8	80.05331172	3.729292158	0.998041977	3.736608524	0.000186519
TRINITY_DN81554_c0_g4_i1	83.26518933	8.742860231	2.340099493	3.736106202	0.000186892

TRINITY_DN84637_c2_g1_i4	30.0000416	6.51425545	1.745104946	3.73287318	0.000189308
TRINITY_DN80389_c3_g1_i3	35.33998696	9.041992428	2.422947658	3.731815006	0.000190105
TRINITY_DN86507_c1_g1_i1	34.69898405	6.260393015	1.678532306	3.729682766	0.000191721
TRINITY_DN79260_c1_g1_i1	120.3519525	-1.837863293	0.492817168	-3.729300466	0.000192012
TRINITY_DN78743_c2_g3_i4	269.3652229	-3.670801645	0.984357052	-3.72913633	0.000192137
TRINITY_DN83115_c1_g1_i9	213.5433296	1.886943485	0.506157537	3.727976659	0.000193023
TRINITY_DN87684_c0_g3_i5	32.78894085	-7.365751629	1.976047071	-3.727518305	0.000193375
TRINITY_DN76091_c0_g4_i2	57.0650731	6.771561534	1.81662189	3.727556941	0.000193345
TRINITY_DN77648_c2_g2_i9	26.9788493	7.053799941	1.892474391	3.727289507	0.00019355
TRINITY_DN84455_c4_g1_i3	81.62173873	-4.044625023	1.085382086	-3.726452716	0.000194193
TRINITY_DN85429_c3_g2_i1	191.6673579	3.382847326	0.90782917	3.726303842	0.000194308
TRINITY_DN77564_c0_g1_i3	33.16189305	6.366826167	1.708803325	3.725897576	0.000194621
TRINITY_DN78597_c0_g1_i1	22.12340971	6.559838652	1.760884209	3.725309488	0.000195076
TRINITY_DN86813_c4_g3_i1	33.18300593	7.173229357	1.92596571	3.724484459	0.000195715
TRINITY_DN82757_c1_g1_i1	98.6004826	8.795900416	2.361765527	3.724290288	0.000195865
TRINITY_DN77231_c0_g3_i2	13.05448332	-7.299979863	1.96027451	-3.723957958	0.000196124
TRINITY_DN87089_c0_g1_i1	20.6128506	-6.565046641	1.763225105	-3.72331736	0.000196622
TRINITY_DN86046_c3_g6_i1	932.9633096	1.473143043	0.395753956	3.72237098	0.000197361
TRINITY_DN86594_c0_g1_i5	30.59678501	6.689782122	1.797211509	3.722312088	0.000197407
TRINITY_DN85228_c1_g2_i5	1642.371857	2.172856587	0.583962468	3.720883973	0.000198527
TRINITY_DN88618_c2_g2_i9	14.85939958	-8.340655062	2.241979929	-3.720218436	0.000199051
TRINITY_DN87593_c3_g1_i2	40.5037344	-6.463228513	1.738499284	-3.717705595	0.00020104
TRINITY_DN85185_c0_g1_i5	2725.136198	2.755993014	0.741436512	3.71709913	0.000201523
TRINITY_DN87629_c1_g3_i4	191.5661428	6.019013978	1.619740318	3.716036397	0.000202372
TRINITY_DN75085_c0_g1_i2	39.77074322	6.145256139	1.653956986	3.715487278	0.000202812
TRINITY_DN82742_c0_g2_i1	13.67481871	7.730591933	2.081160699	3.714557908	0.000203559
TRINITY_DN79214_c0_g2_i4	28.48981871	8.881581599	2.391662166	3.713560271	0.000204364
TRINITY_DN79884_c1_g1_i1	32.52385771	4.849470145	1.305822542	3.713728311	0.000204228
TRINITY_DN86993_c1_g6_i1	205.1056215	1.563179707	0.421000817	3.713008728	0.00020481
TRINITY_DN87940_c1_g1_i9	60.72546219	4.843558344	1.30585383	3.709112178	0.000207987
TRINITY_DN85577_c0_g1_i1	18.37535267	6.605502427	1.780805391	3.709278093	0.000207851
TRINITY_DN86351_c0_g1_i5	62.98172836	3.961043059	1.068008714	3.708811555	0.000208234
TRINITY_DN78710_c0_g4_i2	19.75608674	7.146655861	1.927053576	3.708592201	0.000208415
TRINITY_DN81856_c0_g1_i1	36.27810988	-6.41304967	1.728857997	-3.709413777	0.00020774
TRINITY_DN88123_c0_g1_i2	17.71914648	-7.554503685	2.036937746	-3.708755312	0.000208281
TRINITY_DN79111_c0_g1_i8	35.86440476	-6.146519981	1.657233369	-3.708904308	0.000208158
TRINITY_DN88583_c1_g1_i1	40.68012535	-8.650486444	2.332618739	-3.708487074	0.000208501
TRINITY_DN81978_c4_g2_i1	13.82939466	7.626808798	2.055986679	3.709561388	0.000207619
TRINITY_DN82380_c0_g2_i4	26.62566106	6.691060169	1.804450177	3.708088068	0.00020883
TRINITY_DN83285_c1_g5_i2	74.77558698	-8.843273098	2.385254454	-3.707475773	0.000209335
TRINITY_DN78616_c0_g1_i7	273.7474097	1.579647845	0.426148813	3.706798654	0.000209896
TRINITY_DN82401_c1_g1_i6	47.09947573	-6.147271987	1.659187512	-3.704989305	0.0002114
TRINITY_DN85150_c0_g2_i6	2489.824232	2.018227003	0.544702205	3.705193377	0.00021123
TRINITY_DN87160_c1_g1_i7	13.39403633	8.342543616	2.251439211	3.705426989	0.000211035
TRINITY_DN84974_c1_g1_i1	126.8192785	2.45821985	0.664137744	3.701370498	0.000214438
TRINITY_DN86462_c1_g1_i9	20.33014838	6.418518757	1.734811103	3.699837259	0.000215738
TRINITY_DN79503_c1_g2_i7	1143.104463	2.081905603	0.56281384	3.699101645	0.000216364
TRINITY_DN80011_c0_g1_i7	5993.405107	2.210263542	0.59753581	3.698964153	0.000216481
TRINITY_DN81391_c0_g3_i6	50.1803078	-2.4313226	0.657466345	-3.698018337	0.000217289
TRINITY_DN81453_c2_g1_i8	13.72439222	7.340348092	1.985478398	3.697017354	0.000218147
TRINITY_DN80150_c2_g1_i4	59.58417837	3.796012556	1.026771824	3.697036156	0.000218131
TRINITY_DN85694_c0_g1_i4	54.15784738	6.513003786	1.761633577	3.697138764	0.000218043
TRINITY_DN81213_c3_g2_i5	32.0091774	9.519618108	2.575118329	3.696769194	0.000218361
TRINITY_DN87667_c0_g2_i5	43.2714439	4.077391193	1.103162524	3.696092918	0.000218943
TRINITY_DN74976_c0_g1_i2	108.6137376	4.630027449	1.252884076	3.695495486	0.000219458
TRINITY_DN82381_c0_g2_i5	32.77966375	-9.543588331	2.584422216	-3.692735758	0.000221855
TRINITY_DN79104_c0_g3_i2	151.5628135	-2.021249584	0.547423364	-3.692296891	0.000222238

TRINITY_DN85480_c1_g1_i1	18.77427854	-8.419098977	2.280590763	-3.691630745	0.000222821
TRINITY_DN85179_c1_g5_i1	1941.679615	2.227629009	0.603522034	3.691048351	0.000223332
TRINITY_DN86244_c2_g1_i1	31.60838438	-6.301824675	1.707488403	-3.690698376	0.000223639
TRINITY_DN84615_c1_g3_i4	32.43542541	7.391258031	2.003107355	3.689896107	0.000224346
TRINITY_DN80850_c0_g1_i5	27.89486777	8.36736743	2.269043392	3.687618958	0.000226362
TRINITY_DN85106_c0_g1_i1	35.9538916	-6.200238361	1.681339504	-3.687677799	0.00022631
TRINITY_DN79901_c1_g1_i1	105.6403831	2.468480254	0.669469635	3.687217652	0.000226719
TRINITY_DN84410_c0_g2_i1	14.22351482	6.769269049	1.836262904	3.686437838	0.000227415
TRINITY_DN84262_c2_g5_i2	938.9227732	3.054701662	0.828546767	3.686818639	0.000227075
TRINITY_DN84614_c6_g1_i2	23.46094419	-6.985690487	1.895015027	-3.686350972	0.000227493
TRINITY_DN82943_c1_g1_i9	285.5089379	2.104485234	0.571083991	3.685071318	0.000228639
TRINITY_DN87493_c1_g4_i2	62.77759483	-2.843680479	0.771715	-3.684884289	0.000228807
TRINITY_DN81575_c1_g1_i1	1843.407392	1.963061512	0.532774985	3.684597752	0.000229064
TRINITY_DN86270_c1_g2_i8	69.97928704	-4.852159768	1.31709836	-3.683976774	0.000229623
TRINITY_DN80228_c2_g2_i6	63.35941019	6.083762259	1.651870382	3.682953775	0.000230547
TRINITY_DN83227_c2_g1_i8	13.39540047	8.342691026	2.266989627	3.680074636	0.000233166
TRINITY_DN86150_c0_g2_i7	48.54382863	-4.323967369	1.174920644	-3.680220779	0.000233032
TRINITY_DN81107_c0_g5_i3	19.65926898	-6.534947643	1.77578432	-3.680034546	0.000233202
TRINITY_DN86419_c0_g1_i3	24.24040994	-9.108315191	2.475930727	-3.678743953	0.000234385
TRINITY_DN87396_c2_g1_i2	17.99706997	7.202354822	1.958783696	3.67695261	0.000236037
TRINITY_DN87163_c2_g1_i1	12.80034562	-7.010489025	1.907030857	-3.676127735	0.000236801
TRINITY_DN85283_c2_g3_i4	630.2564659	1.807273561	0.491825312	3.674624951	0.000238199
TRINITY_DN83176_c2_g1_i2	71.60540374	-6.164624926	1.678285488	-3.673168224	0.000239562
TRINITY_DN80476_c2_g1_i1	23.64790037	-7.743263694	2.108724118	-3.672013626	0.000240647
TRINITY_DN86769_c0_g4_i4	48.09349382	-4.435861311	1.208320201	-3.671097533	0.000241511
TRINITY_DN80998_c2_g4_i1	15.93164342	-6.89210641	1.878098954	-3.669724855	0.000242812
TRINITY_DN84255_c3_g1_i1	1731.515349	1.355648282	0.36943258	3.669541767	0.000242986
TRINITY_DN80186_c0_g2_i1	894.92119	1.724683299	0.470093891	3.668806027	0.000243686
TRINITY_DN87033_c0_g2_i6	27.63250516	7.31740792	1.995179225	3.667544162	0.000244891
TRINITY_DN81627_c0_g1_i1	99.63437721	8.488245396	2.314397132	3.667583787	0.000244853
TRINITY_DN81198_c2_g2_i2	486.0420687	4.325956002	1.179774348	3.666765607	0.000245638
TRINITY_DN79261_c1_g5_i4	159.5610043	8.186879275	2.232749871	3.666724779	0.000245677
TRINITY_DN79086_c0_g5_i4	184.8632885	2.780106251	0.758430152	3.665606179	0.000246753
TRINITY_DN88723_c4_g1_i5	5991.018407	1.807253992	0.493126292	3.664890764	0.000247444
TRINITY_DN81133_c0_g4_i2	97.72929298	16.75094739	4.571777148	3.663990357	0.000248316
TRINITY_DN87549_c0_g2_i8	12.8511144	7.668378357	2.092895699	3.664004069	0.000248303
TRINITY_DN86545_c0_g1_i1	27.92774845	-8.326224185	2.27377332	-3.661853234	0.000250397
TRINITY_DN87313_c2_g3_i8	20.65505793	-8.848935329	2.416972216	-3.661165515	0.00025107
TRINITY_DN81330_c0_g1_i8	132.7667196	2.751032132	0.751374356	3.661333543	0.000250906
TRINITY_DN79990_c2_g3_i9	108.5401873	-8.598498931	2.350795756	-3.657697147	0.000254491
TRINITY_DN76284_c0_g3_i2	18.42038891	7.174403401	1.961393889	3.657808583	0.000254381
TRINITY_DN81177_c0_g4_i2	18.55144856	-6.601675771	1.805104372	-3.657226626	0.000254959
TRINITY_DN77488_c1_g5_i3	315.9513919	-2.012011201	0.550226781	-3.656694426	0.000255489
TRINITY_DN88319_c4_g2_i1	1575.639146	1.894452904	0.518123503	3.656373224	0.000255809
TRINITY_DN78997_c1_g5_i9	85.92425508	2.539899641	0.694815555	3.655501988	0.000256679
TRINITY_DN78047_c0_g2_i6	13.54345551	-8.046995425	2.201217541	-3.655702026	0.000256479
TRINITY_DN84964_c1_g4_i2	20.23122454	6.76885212	1.852726655	3.653454276	0.000258736
TRINITY_DN87888_c1_g1_i3	43.90287493	-9.197110227	2.517980645	-3.652573837	0.000259625
TRINITY_DN82051_c1_g2_i1	35.37890199	6.605951774	1.808589904	3.652542657	0.000259656
TRINITY_DN79148_c0_g1_i2	22.56188525	-6.702049927	1.834916188	-3.652510109	0.000259689
TRINITY_DN77328_c1_g4_i5	57.58154569	-3.010205701	0.82428377	-3.651904613	0.000260303
TRINITY_DN85378_c4_g2_i1	18.33054322	-8.011103832	2.194253351	-3.650947521	0.000261275
TRINITY_DN82820_c0_g1_i1	30.72005352	6.561513419	1.797207236	3.650949811	0.000261272
TRINITY_DN87063_c0_g3_i8	10.00062021	6.986497286	1.913652876	3.650869692	0.000261354
TRINITY_DN78430_c3_g5_i5	85.03996088	3.027147123	0.829231968	3.650543199	0.000261686
TRINITY_DN77362_c3_g2_i6	16.06169848	-6.839599068	1.874075012	-3.649586609	0.000262663
TRINITY_DN82664_c3_g5_i1	1097.407679	2.911900006	0.798008101	3.648960457	0.000263304

TRINITY_DN85712_c1_g2_i3	98.58359452	-8.547476375	2.34256211	-3.648772572	0.000263496
TRINITY_DN64854_c0_g1_i1	22.30430978	8.932841108	2.448584445	3.648165424	0.00026412
TRINITY_DN81491_c1_g1_i5	31.82703108	6.561820246	1.798655005	3.648181685	0.000264103
TRINITY_DN84255_c3_g1_i3	36.9851542	6.646281924	1.82193326	3.647928312	0.000264363
TRINITY_DN78169_c2_g8_i2	147.9729146	2.122151158	0.581877821	3.647073461	0.000265244
TRINITY_DN85342_c4_g5_i2	3101.660502	1.531941902	0.419999066	3.647488828	0.000264816
TRINITY_DN85389_c0_g1_i1	22.27724271	-6.711173116	1.84005525	-3.647267176	0.000265044
TRINITY_DN77542_c3_g1_i7	95.83210625	3.854159701	1.056964673	3.64644136	0.000265897
TRINITY_DN86848_c0_g7_i5	16.39993049	6.880934335	1.887142033	3.646219635	0.000266127
TRINITY_DN78376_c2_g4_i1	19.26955985	6.418028222	1.760480982	3.645610653	0.000266758
TRINITY_DN86306_c2_g2_i1	112.2120734	-2.90896945	0.798023697	-3.645216881	0.000267166
TRINITY_DN86223_c1_g1_i6	22.46846281	-6.535689441	1.793509972	-3.644077559	0.000268352
TRINITY_DN81629_c3_g2_i3	13212.29629	1.461167457	0.4009919	3.643882728	0.000268556
TRINITY_DN86851_c2_g3_i7	61.61411712	-9.087096889	2.494133349	-3.643388552	0.000269072
TRINITY_DN86518_c0_g1_i7	23.30714345	-7.29131522	2.001617687	-3.642711227	0.000269781
TRINITY_DN84143_c2_g2_i1	114.3742853	-5.43047248	1.490695824	-3.642911177	0.000269572
TRINITY_DN80708_c2_g1_i1	10.87136595	-6.847619942	1.879709777	-3.642913404	0.00026957
TRINITY_DN84929_c4_g1_i1	303.731184	1.839460642	0.505070298	3.641989344	0.000270539
TRINITY_DN84629_c1_g2_i4	59.83827935	8.707248242	2.391155773	3.641439148	0.000271118
TRINITY_DN88569_c3_g6_i2	22.47401675	6.314577989	1.734605823	3.640353274	0.000272264
TRINITY_DN77560_c1_g1_i4	74.23287495	6.020094312	1.653707421	3.640362398	0.000272255
TRINITY_DN80800_c2_g3_i1	1697.855203	1.799521777	0.494399035	3.639816524	0.000272832
TRINITY_DN88478_c0_g2_i3	14.64362105	-8.108960031	2.227893918	-3.639742433	0.000272911
TRINITY_DN85975_c2_g3_i1	16.91100726	-6.550286221	1.799944089	-3.63916094	0.000273528
TRINITY_DN88684_c2_g3_i2	14.58165225	-6.611100612	1.816621389	-3.639228655	0.000273456
TRINITY_DN86194_c1_g3_i1	403.4610011	2.7955959	0.768329766	3.638536503	0.000274192
TRINITY_DN86774_c0_g2_i4	85.84840322	8.383126309	2.30419031	3.638209168	0.00027454
TRINITY_DN84622_c0_g1_i2	63.64447689	6.728240751	1.849492875	3.637884114	0.000274887
TRINITY_DN84822_c0_g2_i9	14.7821899	-6.654955682	1.830034113	-3.636520016	0.000276346
TRINITY_DN82786_c1_g2_i3	30.06313165	9.443250427	2.596646318	3.636710306	0.000276142
TRINITY_DN81497_c1_g1_i4	701.3651483	-1.603170839	0.440949475	-3.635724564	0.0002772
TRINITY_DN83121_c0_g1_i4	17.42068447	-6.650055121	1.828967504	-3.635961331	0.000276946
TRINITY_DN78647_c3_g3_i4	662.55682	1.171517516	0.32223066	3.635648811	0.000277282
TRINITY_DN85962_c2_g1_i9	96.94831275	2.623270241	0.721769862	3.63449678	0.000278524
TRINITY_DN87178_c0_g1_i7	134.7677033	5.88221554	1.618410673	3.634562993	0.000278452
TRINITY_DN85435_c0_g1_i1	216.2362553	-2.528583462	0.695807304	-3.634028341	0.00027903
TRINITY_DN87985_c1_g2_i5	34.02535277	8.971583435	2.469019784	3.633662028	0.000279427
TRINITY_DN88146_c0_g3_i1	18.41503885	6.417696975	1.766494433	3.633012851	0.000280131
TRINITY_DN82507_c0_g2_i3	1243.886817	1.692200746	0.46589521	3.632148837	0.000281071
TRINITY_DN78354_c0_g2_i2	1047.312842	1.587567431	0.437062302	3.632359563	0.000280841
TRINITY_DN77808_c4_g1_i2	19.11313156	8.65014035	2.383384804	3.629351138	0.000284135
TRINITY_DN83360_c2_g1_i1	17.52944509	-7.560962667	2.083388838	-3.62916539	0.000284339
TRINITY_DN83579_c2_g5_i5	22.19052737	6.559212222	1.807029593	3.629831103	0.000283607
TRINITY_DN88067_c0_g2_i1	133.4592204	2.053568656	0.565865198	3.629077496	0.000284436
TRINITY_DN83176_c2_g1_i1	89.90382792	8.476370076	2.335348083	3.629596006	0.000283865
TRINITY_DN86314_c3_g1_i8	134.9058259	-4.232019889	1.166438872	-3.628154024	0.000285455
TRINITY_DN82865_c0_g1_i4	14.90935191	7.260518552	2.001193438	3.628094322	0.000285521
TRINITY_DN87059_c1_g2_i2	2227.633594	1.642747286	0.452863208	3.627469082	0.000286213
TRINITY_DN78741_c3_g2_i6	28.11260127	6.730209019	1.855754273	3.626670361	0.000287099
TRINITY_DN79426_c2_g1_i3	15.79440821	7.805933016	2.152768479	3.62599745	0.000287848
TRINITY_DN76436_c0_g1_i3	10.41184749	-6.937827684	1.913340598	-3.626028576	0.000287814
TRINITY_DN78450_c1_g3_i4	110.573418	2.480459121	0.684284845	3.624892676	0.000289081
TRINITY_DN87390_c2_g1_i6	991.7092954	7.534841771	2.07892866	3.624386886	0.000289648
TRINITY_DN79400_c3_g1_i1	35.82574786	3.421830833	0.944365698	3.623417115	0.000290736
TRINITY_DN87670_c0_g1_i2	96.5486164	-7.137338009	1.97043996	-3.62220527	0.000292102
TRINITY_DN77665_c3_g1_i5	430.8579519	1.710460012	0.472269563	3.621787526	0.000292574
TRINITY_DN80909_c0_g1_i3	1354.438812	2.161719767	0.59683011	3.622001856	0.000292332

TRINITY_DN78590_c0_g1_i4	32.49826399	-8.833153301	2.438933037	-3.621728504	0.000292641
TRINITY_DN82588_c2_g5_i2	223.4426596	2.079752194	0.574348308	3.621064369	0.000293393
TRINITY_DN80847_c1_g1_i3	13.18730096	-7.021198734	1.939111619	-3.620832687	0.000293656
TRINITY_DN81414_c0_g1_i1	181.0874003	2.49987059	0.690506183	3.620344973	0.00029421
TRINITY_DN86286_c0_g3_i3	9.963932698	7.626418847	2.106512362	3.620400708	0.000294147
TRINITY_DN79518_c0_g1_i7	27.13594738	-6.49798468	1.795258303	-3.619526322	0.000295143
TRINITY_DN86732_c3_g1_i4	17.57881632	-6.911802099	1.909968817	-3.618803637	0.000295968
TRINITY_DN88164_c1_g1_i3	208.0995523	2.676625899	0.739637148	3.618836488	0.000295931
TRINITY_DN86457_c1_g6_i1	149.8833444	2.168849646	0.599301114	3.618964817	0.000295784
TRINITY_DN82059_c0_g1_i4	11.09690541	-7.315860479	2.021689642	-3.618686235	0.000296102
TRINITY_DN86215_c1_g1_i8	1906.94345	2.381445971	0.658254779	3.617817972	0.000297097
TRINITY_DN81072_c1_g3_i5	5083.738938	1.502165093	0.415290294	3.617144714	0.000297871
TRINITY_DN88461_c5_g2_i3	234.5311553	-1.769731412	0.489221517	-3.617443937	0.000297527
TRINITY_DN87631_c1_g1_i4	83.65002941	2.520599311	0.696820052	3.617288718	0.000297705
TRINITY_DN88395_c0_g1_i7	17.14527988	6.846017254	1.893328519	3.615863377	0.000299348
TRINITY_DN82035_c0_g2_i2	354.4011439	5.730560281	1.585003131	3.615488303	0.000299782
TRINITY_DN82096_c1_g2_i1	14.18262395	7.898706756	2.185288249	3.614491937	0.000300937
TRINITY_DN83558_c1_g1_i2	47.47807116	-5.905343404	1.634254077	-3.613479377	0.000302115
TRINITY_DN87806_c3_g3_i1	105.3700845	1.737352515	0.480805981	3.613417022	0.000302188
TRINITY_DN77458_c0_g1_i4	381.3363864	7.623647768	2.110545284	3.61216972	0.000303646
TRINITY_DN87845_c1_g4_i1	13.44987308	8.130501882	2.251265227	3.611525547	0.000304401
TRINITY_DN83865_c0_g2_i1	32.4091127	3.204777428	0.887419211	3.611345562	0.000304612
TRINITY_DN88286_c0_g1_i1	13.74412974	6.954082283	1.926137566	3.610376748	0.000305753
TRINITY_DN81702_c0_g1_i4	14.78790843	-7.148639226	1.980764735	-3.609029937	0.000307344
TRINITY_DN85205_c4_g2_i1	728.0984181	4.554418757	1.262244589	3.608190358	0.00030834
TRINITY_DN84379_c1_g6_i1	314.9979245	2.180148228	0.604275893	3.607868946	0.000308722
TRINITY_DN78103_c0_g3_i3	29.02583885	-6.460995459	1.790919363	-3.607641746	0.000308993
TRINITY_DN85473_c0_g4_i2	63.38495114	6.020674698	1.66922831	3.606861123	0.000309923
TRINITY_DN82265_c1_g1_i3	532.399691	1.296470043	0.359534143	3.605971968	0.000310987
TRINITY_DN83177_c0_g1_i6	9.617485378	-6.952313439	1.928306312	-3.605398891	0.000311674
TRINITY_DN82929_c1_g2_i1	13.15622142	-6.701988479	1.859243626	-3.604685467	0.000312531
TRINITY_DN86402_c2_g1_i1	9.529465476	-6.917453207	1.919640185	-3.603515524	0.000313942
TRINITY_DN81421_c1_g2_i7	77.96540193	-7.529687162	2.089423021	-3.603715996	0.0003137
TRINITY_DN81359_c0_g1_i1	25.63945536	6.31613683	1.752714367	3.603631572	0.000313802
TRINITY_DN84425_c0_g1_i7	17.49126515	6.367138808	1.766963379	3.603435637	0.000314039
TRINITY_DN78520_c1_g2_i6	128.3505372	-8.886743814	2.466519254	-3.602949297	0.000314627
TRINITY_DN78706_c0_g1_i1	22.221428	-6.560594275	1.82120086	-3.602345254	0.000315359
TRINITY_DN79201_c0_g1_i9	30.31449617	8.626534143	2.394737766	3.602287593	0.000315429
TRINITY_DN86430_c0_g1_i3	401.130111	2.604303604	0.723039172	3.601884523	0.000315919
TRINITY_DN80843_c2_g1_i8	22.38007662	-6.369520201	1.768596997	-3.601453702	0.000316443
TRINITY_DN75430_c0_g1_i1	23.53405787	9.183490512	2.55025572	3.601007711	0.000316986
TRINITY_DN85825_c1_g4_i5	6236.306095	1.38582114	0.384824994	3.601172378	0.000316785
TRINITY_DN78542_c2_g5_i4	42.53046395	8.656892831	2.40464542	3.600070414	0.000318131
TRINITY_DN79023_c4_g1_i1	16.19297888	-7.071201199	1.964380827	-3.599709946	0.000318572
TRINITY_DN87272_c0_g1_i1	86.90114522	2.914875963	0.810199936	3.597724259	0.000321014
TRINITY_DN84939_c2_g3_i1	44.85702757	-3.482012532	0.967760428	-3.598010861	0.00032066
TRINITY_DN88543_c4_g3_i1	2351.365828	1.475163963	0.41000501	3.597916922	0.000320776
TRINITY_DN84902_c5_g1_i2	5412.150039	2.280317109	0.633989501	3.596774246	0.000322188
TRINITY_DN88106_c1_g4_i1	34.64394721	4.642607745	1.290980908	3.596186214	0.000322917
TRINITY_DN79102_c2_g1_i6	113.1116279	2.292258043	0.637468205	3.595878231	0.000323299
TRINITY_DN85077_c5_g2_i7	78.03645826	-5.820916636	1.619839948	-3.593513448	0.000326249
TRINITY_DN85990_c1_g1_i9	30.13190841	-3.556304125	0.989795882	-3.592967187	0.000326934
TRINITY_DN82444_c1_g1_i4	900.8541817	3.273964851	0.911289639	3.592672088	0.000327304
TRINITY_DN32426_c0_g1_i1	128.0854595	2.060418994	0.573543212	3.592438983	0.000327597
TRINITY_DN79175_c3_g3_i4	95.85541918	6.018564041	1.675923052	3.591193542	0.000329167
TRINITY_DN84070_c2_g1_i2	23.450777	-4.669892942	1.300342164	-3.591280104	0.000329058
TRINITY_DN79521_c0_g2_i4	16.73673849	7.082026757	1.972241171	3.590852305	0.000329598

TRINITY_DN82755_c5_g1_i3	72.5283325	-5.928739468	1.651388118	-3.590155096	0.000330481
TRINITY_DN81222_c3_g5_i2	17.20209342	-5.449290392	1.518166064	-3.589390199	0.000331452
TRINITY_DN87108_c2_g7_i2	3615.233787	1.301162976	0.362562781	3.588793567	0.000332212
TRINITY_DN86260_c0_g1_i2	14.46312398	8.00125672	2.229669862	3.588538758	0.000332537
TRINITY_DN88032_c0_g1_i1	127.854388	-8.401427582	2.341733955	-3.587695161	0.000333614
TRINITY_DN87965_c1_g3_i7	19.17411883	6.514725479	1.816172159	3.587063841	0.000334422
TRINITY_DN84087_c3_g4_i1	52.79112882	-6.910856345	1.926589655	-3.587093041	0.000334385
TRINITY_DN86233_c5_g1_i1	7025.918526	1.566779165	0.436737364	3.587463073	0.000333911
TRINITY_DN83774_c0_g5_i1	108.4735307	3.515686908	0.980241888	3.58655037	0.000335081
TRINITY_DN86348_c1_g1_i3	141.3323587	7.915090181	2.207339114	3.585806156	0.000336039
TRINITY_DN86147_c0_g1_i1	16.96132946	-8.169650992	2.278340197	-3.585790657	0.000336058
TRINITY_DN84280_c0_g3_i1	433.5113587	1.910510494	0.532787993	3.585873779	0.000335951
TRINITY_DN79794_c0_g1_i3	920.0962579	1.620459197	0.451978773	3.585255089	0.000336749
TRINITY_DN84616_c2_g3_i2	460.787704	-1.650988755	0.460543637	-3.58486932	0.000337247
TRINITY_DN80910_c1_g1_i1	26.4765944	6.605361876	1.843104241	3.583824359	0.0003386
TRINITY_DN79966_c1_g7_i2	17.54929394	-7.194624109	2.007628757	-3.583642686	0.000338836
TRINITY_DN88756_c3_g2_i1	66.15422307	6.882457926	1.920497378	3.583685145	0.00033878
TRINITY_DN82071_c6_g2_i3	305.9893372	7.772375201	2.168903431	3.583550604	0.000338955
TRINITY_DN80352_c0_g1_i6	322.2134291	3.688936036	1.029849331	3.582015276	0.000340954
TRINITY_DN79927_c0_g2_i8	76.85545302	8.325698305	2.32442743	3.581827592	0.000341199
TRINITY_DN88476_c2_g1_i1	195.8901835	5.730398466	1.59997267	3.581560218	0.000341548
TRINITY_DN84958_c3_g3_i7	38.55756205	-6.182587886	1.726656485	-3.58067047	0.000342714
TRINITY_DN87784_c1_g3_i1	2229.746474	1.212595239	0.338651808	3.580654852	0.000342734
TRINITY_DN81990_c1_g2_i1	21.82953451	5.361762359	1.497608938	3.58021525	0.000343311
TRINITY_DN83000_c0_g1_i7	20.54201281	-8.489336129	2.372311914	-3.578507564	0.000345562
TRINITY_DN88490_c0_g2_i1	14.46010192	-6.503830939	1.817418684	-3.578609044	0.000345428
TRINITY_DN86050_c1_g1_i1	47.10309708	-6.769856972	1.891656971	-3.578797359	0.000345179
TRINITY_DN78973_c1_g2_i1	13.47590735	7.988060236	2.232447725	3.578162277	0.000346019
TRINITY_DN86419_c0_g8_i1	20.91169562	-6.882477017	1.923810346	-3.577523654	0.000346865
TRINITY_DN88804_c7_g6_i1	195.7958349	2.465089992	0.689327981	3.576077081	0.000348789
TRINITY_DN85179_c1_g5_i1	184.0086359	6.512479102	1.821590486	3.57516091	0.000350013
TRINITY_DN82188_c5_g1_i1	468.4104576	1.734468623	0.485134523	3.575232311	0.000349917
TRINITY_DN87442_c1_g1_i1	91.19192002	16.29528183	4.557543894	3.575452527	0.000349623
TRINITY_DN86051_c2_g2_i1	25.3347854	9.289855307	2.598607559	3.574935845	0.000350314
TRINITY_DN83671_c3_g1_i2	9.620620006	-6.932966158	1.939425419	-3.574752651	0.000350559
TRINITY_DN84723_c2_g2_i1	123.982476	7.989202023	2.236400706	3.572348194	0.000353795
TRINITY_DN87221_c2_g5_i1	154.1672073	1.770373096	0.495680081	3.571604278	0.000354801
TRINITY_DN83555_c0_g2_i1	47.0434163	5.882419122	1.647211159	3.57113846	0.000355433
TRINITY_DN79911_c3_g2_i1	18.60151184	-6.77101497	1.89652528	-3.57022131	0.00035668
TRINITY_DN82786_c0_g1_i1	213.7102898	-7.853974154	2.199871367	-3.570197	0.000356713
TRINITY_DN84143_c2_g2_i1	38.97063853	-2.766841028	0.774993678	-3.570146577	0.000356782
TRINITY_DN82378_c1_g1_i2	90.50803852	-8.287714336	2.321482842	-3.57000887	0.000356969
TRINITY_DN80881_c1_g1_i3	27.41202194	6.204056676	1.738084856	3.569478586	0.000357692
TRINITY_DN88804_c7_g5_i1	1724.085296	2.049101299	0.574237021	3.568389397	0.000359182
TRINITY_DN83872_c0_g3_i5	15.23117512	-7.532044289	2.111568731	-3.567037236	0.00036104
TRINITY_DN81631_c1_g1_i5	25.98303653	6.846935526	1.919007174	3.567957232	0.000359775
TRINITY_DN86564_c1_g1_i1	1618.429556	1.269663433	0.355877894	3.567694013	0.000360137
TRINITY_DN83868_c0_g2_i3	67.59051979	4.374460067	1.226262693	3.567310734	0.000360664
TRINITY_DN87253_c3_g2_i6	48.73753324	-7.01817541	1.967551118	-3.566959632	0.000361147
TRINITY_DN85011_c0_g1_i4	30.32206571	-6.78330018	1.901464068	-3.567409079	0.000360528
TRINITY_DN65085_c0_g1_i1	21.8268158	7.727949671	2.167248582	3.565788316	0.000362764
TRINITY_DN88427_c1_g10_i	26.27398414	8.559563403	2.401073591	3.564890071	0.000364009
TRINITY_DN80478_c0_g1_i3	45.86752709	-8.863426463	2.486613126	-3.564457362	0.00036461
TRINITY_DN82083_c1_g3_i1	13.53013248	-7.686449993	2.156711769	-3.563967196	0.000365292
TRINITY_DN87550_c0_g1_i1	13.83670334	-6.415491887	1.800103875	-3.563956489	0.000365307
TRINITY_DN82384_c0_g2_i2	14.40713103	-6.911596975	1.939468892	-3.563654463	0.000365727
TRINITY_DN85653_c1_g1_i1	15.12122025	6.560914272	1.841134504	3.563517091	0.000365919

TRINITY_DN88380_c1_g1_i5	32.75776079	-8.194615726	2.300327106	-3.562369762	0.000367522
TRINITY_DN82252_c0_g2_i9	75.25351626	2.188769738	0.614533922	3.561674399	0.000368497
TRINITY_DN84308_c2_g4_i3	17.36031312	-6.369548973	1.788388413	-3.561613867	0.000368582
TRINITY_DN79794_c0_g2_i5	1031.74334	1.139968014	0.320129385	3.560960245	0.000369501
TRINITY_DN80301_c0_g1_i2	429.6751131	-1.941820154	0.545371391	-3.560546419	0.000370084
TRINITY_DN85500_c3_g1_i7	17.25229797	-7.450881234	2.092678141	-3.560452556	0.000370216
TRINITY_DN87030_c1_g1_i5	26.07223293	-6.620758352	1.860660058	-3.558284773	0.000373284
TRINITY_DN88710_c2_g2_i3	14.45866887	7.41605366	2.084516765	3.557684824	0.000374138
TRINITY_DN77345_c0_g1_i1	19.6715441	-6.581141878	1.849817569	-3.55772482	0.000374081
TRINITY_DN84474_c2_g3_i5	92.18588269	3.738207441	1.051234709	3.556015996	0.000376521
TRINITY_DN80190_c2_g6_i1	395.842004	1.97401751	0.555147882	3.555840837	0.000376772
TRINITY_DN88827_c2_g4_i2	12.28310452	-6.498047077	1.827801461	-3.555116469	0.000377812
TRINITY_DN78877_c2_g1_i2	94.62114444	6.143733798	1.728063467	3.555270923	0.00037759
TRINITY_DN77790_c0_g7_i2	658.7059719	2.006853739	0.564489306	3.555166977	0.000377739
TRINITY_DN84797_c0_g2_i1	11.24088836	7.174296658	2.018195925	3.55480683	0.000378257
TRINITY_DN87978_c0_g2_i1	48.96339117	-6.03406268	1.697610417	-3.554444895	0.000378778
TRINITY_DN87528_c0_g2_i1	2924.55551	1.993806364	0.560996424	3.554044693	0.000379355
TRINITY_DN85711_c1_g2_i3	13.1107468	6.559513945	1.846574131	3.552261365	0.000381935
TRINITY_DN84267_c2_g1_i2	17.43684963	-6.796678633	1.913440749	-3.552071646	0.000382211
TRINITY_DN82174_c3_g1_i3	13.80465005	8.233815856	2.318068949	3.552015077	0.000382293
TRINITY_DN87080_c0_g2_i6	103.2050365	-2.145975529	0.604126406	-3.55219621	0.00038203
TRINITY_DN88059_c1_g2_i1	49.70960978	8.655531275	2.43755417	3.550908275	0.000383904
TRINITY_DN82421_c1_g3_i2	152.5915894	7.727475277	2.17632858	3.550693285	0.000384218
TRINITY_DN82989_c1_g2_i9	63.2575091	-7.380261728	2.078708946	-3.550406488	0.000384637
TRINITY_DN88310_c0_g1_i1	18.83766712	6.730776285	1.895966825	3.550049609	0.000385159
TRINITY_DN85195_c0_g2_i4	24.12284305	9.153190737	2.578775856	3.549432463	0.000386062
TRINITY_DN81450_c0_g4_i5	23.15948327	-4.983149937	1.40413534	-3.548909992	0.000386829
TRINITY_DN84032_c2_g1_i1	19.37033774	-8.184859818	2.306645769	-3.54838178	0.000387606
TRINITY_DN80290_c0_g4_i1	258.0097533	1.741218559	0.490739328	3.548153692	0.000387942
TRINITY_DN82381_c0_g2_i1	304.9700968	7.786355704	2.19463376	3.547906646	0.000388306
TRINITY_DN87990_c0_g1_i1	96.70973115	8.201372447	2.311870071	3.547505783	0.000388897
TRINITY_DN66081_c0_g1_i1	79.25752846	-9.36002143	2.638887299	-3.546957626	0.000389707
TRINITY_DN81357_c1_g3_i1	880.642246	1.317644975	0.37157289	3.546127857	0.000390936
TRINITY_DN87707_c1_g1_i2	18.77648138	-6.630909646	1.87012027	-3.545712944	0.000391552
TRINITY_DN84511_c3_g1_i4	416.2520658	1.391041964	0.392305807	3.545810284	0.000391408
TRINITY_DN83302_c3_g3_i2	15.65647237	6.513448531	1.837253234	3.545209996	0.0003923
TRINITY_DN78047_c0_g1_i9	35.93343811	6.083175537	1.716175676	3.544611208	0.000393193
TRINITY_DN88307_c1_g1_i1	108.1001399	6.083748481	1.716667446	3.543929544	0.000394211
TRINITY_DN80236_c1_g2_i2	12.66714801	6.604914301	1.863766973	3.543851993	0.000394327
TRINITY_DN87970_c1_g3_i3	28.32573507	6.512962897	1.838463651	3.542611731	0.000396186
TRINITY_DN83267_c1_g4_i1	1816.47182	2.387899649	0.674199342	3.54183029	0.000397361
TRINITY_DN79020_c1_g1_i1	13.4555497	-7.925175686	2.238195877	-3.540876725	0.0003988
TRINITY_DN86084_c1_g1_i5	170.4213192	1.878201542	0.530486066	3.540529457	0.000399325
TRINITY_DN83980_c1_g1_i3	143.6847901	15.86004493	4.479276414	3.540760484	0.000398976
TRINITY_DN82573_c3_g1_i1	9.212896466	-7.187642559	2.03019921	-3.540363193	0.000399577
TRINITY_DN84191_c2_g1_i1	33.74885691	-4.089969517	1.155141957	-3.540663978	0.000399122
TRINITY_DN86938_c3_g1_i2	59.34479209	8.806170912	2.488112918	3.539297131	0.000401194
TRINITY_DN82387_c0_g3_i4	39.20346317	-4.01904321	1.135660802	-3.538946844	0.000401727
TRINITY_DN88566_c0_g2_i1	33.72743293	6.144302732	1.736469868	3.538387187	0.000402579
TRINITY_DN87464_c1_g1_i2	63.30664372	8.364865619	2.364058829	3.53834918	0.000402637
TRINITY_DN81139_c2_g1_i5	42.60531159	8.767872321	2.478603128	3.53742486	0.000404049
TRINITY_DN83488_c2_g6_i1	151.9166809	5.730211901	1.620910848	3.535180178	0.000407497
TRINITY_DN77488_c0_g1_i2	16.67307483	6.26109741	1.771500554	3.534346854	0.000408784
TRINITY_DN85272_c0_g3_i5	42.69067068	-4.254136265	1.203781693	-3.533976541	0.000409357
TRINITY_DN82024_c2_g1_i1	80.29577278	2.332194821	0.660070816	3.533249411	0.000410485
TRINITY_DN86039_c1_g3_i5	34.08767006	3.297948127	0.933502236	3.532876515	0.000411064
TRINITY_DN87570_c0_g2_i3	56.66283777	6.810498229	1.928310845	3.531846666	0.000412669

TRINITY_DN78772_c0_g4_i1	20.7295393	8.909841305	2.523431643	3.530843139	0.000414237
TRINITY_DN81378_c3_g1_i1	29.56602391	4.630062083	1.311311634	3.530863271	0.000414206
TRINITY_DN88408_c1_g1_i7	40.69359515	7.861605169	2.226518598	3.530895801	0.000414155
TRINITY_DN87178_c0_g1_i1	3638.833478	1.835945776	0.519996312	3.530689994	0.000414477
TRINITY_DN86196_c4_g7_i1	23.10361084	5.341096169	1.512541033	3.531207454	0.000413667
TRINITY_DN87907_c1_g1_i5	27.02993936	8.313273788	2.355531572	3.529255939	0.00041673
TRINITY_DN74021_c0_g1_i1	98.92893663	3.17313226	0.899053575	3.529413983	0.000416481
TRINITY_DN86363_c1_g3_i1	1096.684653	2.468865892	0.699591412	3.529011149	0.000417116
TRINITY_DN76877_c0_g1_i5	24.75544191	-4.444768904	1.259544519	-3.528870028	0.000417338
TRINITY_DN88359_c0_g1_i1	150.582311	-7.919741706	2.244714915	-3.528172622	0.000418439
TRINITY_DN85116_c0_g2_i1	14.68701722	7.512745834	2.129339374	3.528205004	0.000418388
TRINITY_DN83760_c2_g5_i3	441.1942569	-1.342380433	0.380473845	-3.528180583	0.000418427
TRINITY_DN83011_c0_g1_i1	56.40597549	8.782117179	2.489331659	3.527901615	0.000418868
TRINITY_DN80737_c1_g1_i3	20.15232791	-7.263050248	2.059191413	-3.527137013	0.000420079
TRINITY_DN78840_c1_g1_i4	31.19688581	-3.778885698	1.071341024	-3.527248198	0.000419903
TRINITY_DN87301_c3_g2_i9	50.03985229	-5.994734943	1.699749391	-3.526834589	0.000420559
TRINITY_DN85239_c3_g4_i1	19.76542173	-8.787106381	2.491873981	-3.52630448	0.000421402
TRINITY_DN76869_c2_g1_i1	14.96696363	-6.755544095	1.915667214	-3.526470592	0.000421138
TRINITY_DN79591_c0_g3_i1	306.5282347	-2.72460089	0.772950641	-3.524935159	0.000423586
TRINITY_DN79175_c3_g1_i3	62.4823402	6.02091431	1.7089633	3.523138449	0.000426469
TRINITY_DN81574_c0_g2_i1	14.93341175	8.514224903	2.41706354	3.522549061	0.000427418
TRINITY_DN81625_c0_g1_i8	736.4773585	-7.352769891	2.087528158	-3.522237467	0.000427921
TRINITY_DN88159_c0_g2_i1	74.21010963	8.102102969	2.300467816	3.52193711	0.000428406
TRINITY_DN84158_c2_g1_i2	28.32062234	-8.220030589	2.334638038	-3.520901508	0.000430082
TRINITY_DN82950_c1_g2_i4	29.20761827	-7.586822	2.154807907	-3.520880898	0.000430116
TRINITY_DN80559_c0_g2_i1	2128.449382	1.525632647	0.433373947	3.52036078	0.00043096
TRINITY_DN78610_c0_g4_i2	157.8169419	2.38539446	0.677743205	3.519613979	0.000432175
TRINITY_DN81626_c0_g1_i8	8.68052926	-7.072189611	2.00959816	-3.519205856	0.000432841
TRINITY_DN85184_c0_g2_i5	22.69504467	-8.37220335	2.380540642	-3.516933592	0.000436563
TRINITY_DN88072_c1_g1_i3	20.48874826	3.457459016	0.983259365	3.516324522	0.000437566
TRINITY_DN82335_c4_g1_i7	37.8677171	-9.579372677	2.724593071	-3.515891154	0.000438281
TRINITY_DN83124_c0_g1_i3	141.04612	7.645017373	2.17470991	3.515419385	0.00043906
TRINITY_DN82874_c0_g1_i1	22.00046135	-6.516770666	1.854046375	-3.514890865	0.000439935
TRINITY_DN85494_c0_g2_i4	66.68562377	-8.255028031	2.350028758	-3.512734899	0.00044352
TRINITY_DN80211_c0_g6_i2	538.4096446	1.416228405	0.403186265	3.512590895	0.00044376
TRINITY_DN81152_c2_g2_i1	112.0561796	2.423962367	0.690211031	3.511914847	0.000444891
TRINITY_DN78509_c1_g3_i2	66.96580701	4.41710943	1.257849373	3.511636229	0.000445357
TRINITY_DN85074_c1_g2_i8	56.62272961	8.01899975	2.283769701	3.511299649	0.000445921
TRINITY_DN83571_c0_g2_i3	27.50491329	4.664158377	1.328469303	3.510926722	0.000446547
TRINITY_DN86278_c2_g1_i4	62.55358777	-6.255139485	1.781577336	-3.511012044	0.000446404
TRINITY_DN79513_c1_g1_i1	260.1701333	2.084409466	0.593785512	3.510374412	0.000447476
TRINITY_DN81894_c0_g6_i1	422.4885605	1.879965543	0.535557296	3.510297694	0.000447605
TRINITY_DN84723_c2_g2_i2	25.71450117	6.468166435	1.843166356	3.509268935	0.00044934
TRINITY_DN83005_c1_g2_i1	14.04024985	6.846416345	1.951167027	3.508882761	0.000449993
TRINITY_DN82001_c1_g3_i5	11.62516166	-7.512595551	2.141178418	-3.508626598	0.000450427
TRINITY_DN84974_c1_g1_i9	14.19044323	-7.842322915	2.235358558	-3.508306481	0.000450969
TRINITY_DN82230_c4_g1_i1	38.52832339	-3.549098704	1.012113505	-3.50662123	0.000453835
TRINITY_DN86018_c0_g1_i2	161.8262526	7.64501494	2.180103923	3.506720418	0.000453666
TRINITY_DN85432_c0_g1_i3	9.663832904	7.262050159	2.071414848	3.505840544	0.000455168
TRINITY_DN86227_c0_g2_i2	24.51529207	6.261096068	1.786340693	3.504984292	0.000456634
TRINITY_DN83281_c2_g7_i3	21.69187142	8.260691758	2.356780141	3.505075256	0.000456478
TRINITY_DN84943_c2_g1_i1	33.45183772	-9.453792113	2.697491294	-3.504660844	0.000457189
TRINITY_DN78209_c1_g1_i1	52.81060299	8.538458448	2.436698053	3.504110177	0.000458136
TRINITY_DN77433_c0_g1_i2	24.66806377	7.05078752	2.012153823	3.504099657	0.000458154
TRINITY_DN85222_c0_g1_i2	12.41361369	-6.591133104	1.881372373	-3.503364458	0.00045942
TRINITY_DN85492_c1_g3_i1	99.02788912	-3.351911561	0.956955368	-3.502683274	0.000460597
TRINITY_DN85358_c4_g4_i2	2445.799729	1.632222469	0.465988037	3.502713245	0.000460545

TRINITY_DN83379_c1_g1_i9	148.5649827	7.623644318	2.17688822	3.502083501	0.000461635
TRINITY_DN79470_c1_g2_i1	16.4788597	6.260917719	1.787764079	3.502093925	0.000461617
TRINITY_DN86197_c3_g1_i1	36.82338781	-3.774220744	1.077836295	-3.501664179	0.000462362
TRINITY_DN87091_c2_g1_i1	19.85596026	6.691095356	1.911070499	3.50122895	0.000463118
TRINITY_DN84933_c2_g7_i1	3606.418035	1.971941356	0.563241914	3.501055776	0.000463419
TRINITY_DN83876_c1_g4_i1	24.19705675	-6.197821104	1.770645159	-3.500317991	0.000464703
TRINITY_DN79512_c1_g4_i1	42.65711637	4.029961962	1.151331498	3.500262061	0.000464801
TRINITY_DN85514_c1_g1_i1	19.73905538	-5.606493435	1.601864261	-3.499980349	0.000465292
TRINITY_DN84137_c0_g3_i2	50.7865214	6.416312869	1.833796746	3.498922595	0.000467142
TRINITY_DN82087_c1_g4_i2	36.54792386	5.882014381	1.68097862	3.499160733	0.000466725
TRINITY_DN87356_c2_g1_i1	19.41313891	8.838095499	2.525828983	3.499087055	0.000466854
TRINITY_DN88493_c3_g4_i2	601.0072064	2.299809594	0.657552291	3.497531109	0.000469586
TRINITY_DN85218_c0_g1_i8	26.98088345	6.019322444	1.721062941	3.49744469	0.000469738
TRINITY_DN86179_c1_g2_i5	22.19203808	-6.997831227	2.000499315	-3.498042301	0.000468687
TRINITY_DN80713_c0_g6_i2	30.06958722	-5.85419496	1.673778273	-3.497592875	0.000469477
TRINITY_DN80808_c0_g6_i1	11.41751084	-6.529831444	1.866868965	-3.497744923	0.00046921
TRINITY_DN86507_c1_g1_i3	49.87904708	-4.061207923	1.1615346	-3.496415795	0.000471553
TRINITY_DN88493_c3_g1_i7	25.70846517	-8.478970703	2.425501937	-3.495759197	0.000472715
TRINITY_DN84668_c0_g1_i2	79.01872423	-2.781078981	0.795637036	-3.495411669	0.000473331
TRINITY_DN87784_c1_g3_i3	890.3827661	1.432130748	0.409726822	3.495330718	0.000473475
TRINITY_DN81706_c3_g1_i6	44.65798861	8.481815415	2.428396165	3.492764293	0.000478048
TRINITY_DN83147_c0_g1_i1	136.7207478	5.951059164	1.704148208	3.492101882	0.000479235
TRINITY_DN79023_c4_g1_i1	20.10892338	7.666956636	2.195422122	3.492247144	0.000478975
TRINITY_DN85191_c0_g2_i3	18.70955552	7.365246815	2.109336577	3.491736167	0.000479892
TRINITY_DN81605_c0_g1_i2	111.367565	-7.807200185	2.236217916	-3.491251961	0.000480763
TRINITY_DN78031_c2_g3_i7	119.3549397	-3.962497171	1.135015152	-3.491140329	0.000480964
TRINITY_DN87727_c2_g3_i2	15.45326649	6.646256876	1.904110917	3.490477795	0.000482158
TRINITY_DN83967_c2_g1_i2	141.9472891	-7.631993971	2.187047603	-3.489633221	0.000483684
TRINITY_DN84043_c0_g2_i4	3416.086768	1.119673509	0.320859487	3.489606995	0.000483731
TRINITY_DN83584_c1_g1_i9	20.94888846	-6.741674781	1.931877339	-3.489701259	0.000483561
TRINITY_DN86414_c0_g5_i2	17.71955845	6.514243688	1.866913444	3.48931211	0.000484265
TRINITY_DN80297_c2_g2_i1	109.451457	15.31930836	4.390699524	3.489035921	0.000484766
TRINITY_DN87390_c2_g1_i8	4505.762744	1.420422706	0.407147716	3.488715891	0.000485347
TRINITY_DN82996_c1_g3_i2	41.26875286	-4.015155669	1.15105311	-3.488245358	0.000486202
TRINITY_DN82588_c2_g2_i7	75.92346906	2.529836975	0.725287506	3.488047089	0.000486562
TRINITY_DN81849_c0_g1_i1	57.87949536	-2.269413922	0.650697969	-3.487660992	0.000487265
TRINITY_DN83972_c2_g1_i6	33.47624843	-6.28963578	1.803872582	-3.486740607	0.000488945
TRINITY_DN82372_c0_g2_i3	68.57772043	-5.804923847	1.665002773	-3.486434943	0.000489504
TRINITY_DN85788_c1_g2_i4	112.279824	2.375822926	0.681853532	3.484359639	0.000493316
TRINITY_DN80453_c0_g1_i8	25.81649495	9.173974356	2.633365093	3.483745714	0.000494449
TRINITY_DN86869_c0_g1_i1	26.64565147	-8.591618375	2.466616736	-3.483159037	0.000495534
TRINITY_DN86579_c1_g3_i1	19.7893889	6.203677162	1.780999817	3.483255362	0.000495356
TRINITY_DN81306_c2_g1_i2	28.74804852	6.316517897	1.813638909	3.482786936	0.000496223
TRINITY_DN82962_c3_g1_i2	11.20432856	7.751615689	2.22563319	3.482881061	0.000496049
TRINITY_DN87146_c1_g4_i4	21.20639838	6.203865373	1.781714756	3.48196329	0.000497752
TRINITY_DN79508_c1_g5_i1	1379.12236	1.813028126	0.520865417	3.480799579	0.000499919
TRINITY_DN81067_c1_g3_i6	15.42304435	8.561396463	2.460342027	3.479758656	0.000501866
TRINITY_DN84003_c1_g1_i1	17.64688673	-6.565946136	1.88710062	-3.479383168	0.000502569
TRINITY_DN79093_c1_g1_i1	63.2824036	8.478886147	2.437186262	3.478965182	0.000503354
TRINITY_DN80180_c0_g3_i2	44.93881495	6.465681334	1.858942573	3.478150121	0.000504887
TRINITY_DN80750_c1_g6_i2	35.55678372	6.602603052	1.898808734	3.477234402	0.000506615
TRINITY_DN86126_c1_g3_i1	1354.8004	1.442482197	0.414962592	3.476174053	0.000508622
TRINITY_DN83734_c1_g2_i1	23.08160408	-6.220332638	1.789338721	-3.476330427	0.000508326
TRINITY_DN88400_c1_g1_i1	48.37979074	-6.794147654	1.954326273	-3.476465393	0.00050807
TRINITY_DN82096_c1_g2_i2	11.10118122	7.709999659	2.218233639	3.475738318	0.000509449
TRINITY_DN80581_c0_g1_i6	52.62061364	5.808113011	1.671114941	3.475591575	0.000509728
TRINITY_DN84696_c0_g1_i1	28.63074904	-6.686978182	1.924859228	-3.474008947	0.000512744

TRINITY_DN79959_c0_g1_i2	410.548792	1.832393843	0.527649228	3.472749977	0.000515155
TRINITY_DN83555_c0_g1_i5	18.16469973	-6.444031227	1.856139255	-3.471739101	0.000517099
TRINITY_DN86465_c0_g1_i9	34.8599468	5.9535437	1.714964872	3.471525159	0.000517511
TRINITY_DN76952_c0_g1_i1	191.4868591	1.9648648	0.566077068	3.471019954	0.000518485
TRINITY_DN87010_c1_g2_i5	54.95053375	-8.220821538	2.368764725	-3.470509946	0.000519471
TRINITY_DN82646_c2_g2_i5	20.52235592	-8.275331259	2.384613238	-3.470303329	0.000519871
TRINITY_DN79019_c1_g1_i3	62.9063237	3.712229755	1.069863721	3.46981553	0.000520816
TRINITY_DN81341_c1_g1_i6	21.49801256	-6.265502706	1.80575744	-3.469736615	0.000520969
TRINITY_DN85417_c0_g1_i9	23.63968746	6.559655198	1.890533125	3.469738303	0.000520966
TRINITY_DN79952_c1_g3_i1	107.3700877	2.562248418	0.738580887	3.469150723	0.000522106
TRINITY_DN86018_c0_g1_i6	112.758288	-7.851480292	2.2634738	-3.468774541	0.000522838
TRINITY_DN87247_c2_g1_i2	46.37645096	-4.370370272	1.260274961	-3.467791083	0.000524755
TRINITY_DN83330_c1_g1_i4	22.54844347	-6.649113714	1.91788567	-3.466897854	0.000526502
TRINITY_DN84992_c1_g1_i2	96.84990743	1.914460198	0.55227927	3.466471224	0.000527338
TRINITY_DN84992_c1_g5_i2	96.84990743	1.914460198	0.55227927	3.466471224	0.000527338
TRINITY_DN87313_c2_g3_i1	24.25691366	-9.044048876	2.609254698	-3.466142606	0.000527983
TRINITY_DN88143_c3_g1_i1	77.75221683	5.730428494	1.653939175	3.464715377	0.000530793
TRINITY_DN78688_c1_g2_i8	29.02653467	6.019111753	1.73723963	3.464756185	0.000530712
TRINITY_DN86294_c3_g1_i7	151.3631013	7.488301753	2.161817299	3.463892049	0.00053242
TRINITY_DN78604_c0_g1_i1	28.89464704	-9.213375899	2.660168471	-3.463455792	0.000533284
TRINITY_DN79557_c0_g1_i1	21.10152106	-8.908307278	2.572079101	-3.463465519	0.000533265
TRINITY_DN77656_c0_g1_i3	14.31913945	6.604300859	1.907073738	3.463054797	0.00053408
TRINITY_DN77091_c0_g1_i2	14.76897345	-7.085903005	2.046051979	-3.463207718	0.000533776
TRINITY_DN80444_c1_g2_i6	51.87861936	-5.779184383	1.66913612	-3.462380517	0.00053542
TRINITY_DN82758_c0_g1_i1	12.39107077	6.604607208	1.907546386	3.462357328	0.000535466
TRINITY_DN77659_c0_g1_i3	16.09967691	-6.888310731	1.989727261	-3.461937153	0.000536302
TRINITY_DN77788_c0_g1_i6	17.45676335	8.217903171	2.373833657	3.461869852	0.000536436
TRINITY_DN80148_c0_g4_i5	26.41082698	-6.220493144	1.797395612	-3.460836947	0.000538499
TRINITY_DN85948_c0_g1_i1	17.6297174	6.315628329	1.824939536	3.46073292	0.000538707
TRINITY_DN87554_c3_g1_i2	121.3409518	-2.441850759	0.705533591	-3.460998583	0.000538176
TRINITY_DN86507_c1_g1_i1	56.70415438	-5.49760971	1.588950467	-3.459899993	0.000540376
TRINITY_DN86889_c2_g3_i6	8.826289921	-7.058303969	2.040207711	-3.459600673	0.000540977
TRINITY_DN88757_c3_g8_i1	203.2353202	1.772816378	0.512449192	3.459496874	0.000541186
TRINITY_DN84499_c0_g1_i7	20.35865559	-8.407788353	2.430463444	-3.459335451	0.00054151
TRINITY_DN82361_c2_g1_i8	10.18505723	6.690232407	1.934324049	3.458692668	0.000542804
TRINITY_DN80704_c0_g1_i2	79.98130676	4.023756972	1.163362247	3.458730918	0.000542727
TRINITY_DN79223_c1_g3_i1	214.3628818	-3.916739895	1.132540614	-3.458365947	0.000543463
TRINITY_DN83113_c3_g1_i6	42.30152152	-5.839990683	1.688872295	-3.457923196	0.000544357
TRINITY_DN84682_c1_g2_i2	24.73291894	6.083754135	1.759757966	3.45715391	0.000545913
TRINITY_DN78741_c3_g2_i5	10.27149052	6.647304639	1.923136807	3.456490779	0.000547258
TRINITY_DN88365_c5_g2_i5	16.48052658	-8.530444638	2.468128602	-3.456239935	0.000547767
TRINITY_DN83486_c0_g1_i1	701.7610517	-1.172377166	0.339282915	-3.455455948	0.000549363
TRINITY_DN87238_c0_g7_i2	309.8751536	1.712526908	0.495684352	3.454873853	0.00055055
TRINITY_DN85978_c0_g1_i1	21.24412189	-8.918224671	2.581748492	-3.454335191	0.000551651
TRINITY_DN84280_c1_g3_i9	47.53484743	8.427869473	2.440054529	3.453967676	0.000552404
TRINITY_DN82383_c2_g1_i1	5473.448573	1.451905311	0.42036818	3.45388966	0.000552563
TRINITY_DN82944_c0_g1_i9	22.28138219	-6.40477267	1.855175601	-3.452380824	0.000555663
TRINITY_DN81644_c2_g2_i7	11.24908153	6.919426981	2.004576107	3.451815552	0.000556828
TRINITY_DN83079_c0_g3_i7	72.38016438	2.631228071	0.762565343	3.450495223	0.000559559
TRINITY_DN78322_c1_g1_i7	56.62371668	8.054798272	2.334383424	3.450503541	0.000559542
TRINITY_DN88535_c4_g1_i1	86.66342967	-3.665063586	1.062087984	-3.450809765	0.000558907
TRINITY_DN83275_c4_g2_i1	20.29411245	8.969805211	2.599764096	3.450238129	0.000560092
TRINITY_DN85365_c0_g2_i4	41.26795045	-4.626129934	1.340912151	-3.449987332	0.000560613
TRINITY_DN78597_c0_g1_i3	19.6168353	7.826592713	2.269041426	3.449294765	0.000562053
TRINITY_DN87870_c0_g1_i1	60.70419872	-8.340398738	2.418135751	-3.449102778	0.000562453
TRINITY_DN87136_c1_g1_i6	51.76012291	3.976948421	1.153318853	3.448264468	0.000564201
TRINITY_DN83243_c0_g1_i1	14.91996371	6.559814774	1.902649386	3.447726535	0.000565326

TRINITY_DN80983_c0_g1_i2	13.75280957	7.844148992	2.275312735	3.447503664	0.000565793
TRINITY_DN84902_c5_g1_i3	609.1013626	-5.462160803	1.584478614	-3.447292224	0.000566236
TRINITY_DN88687_c1_g1_i1	18.07322558	-6.689016175	1.941533281	-3.445223546	0.000570587
TRINITY_DN79568_c2_g1_i1	84.6957009	7.752758696	2.25050854	3.444891924	0.000571288
TRINITY_DN83208_c0_g1_i5	13.77966995	-6.611719241	1.920280024	-3.44310161	0.000575083
TRINITY_DN84717_c0_g1_i5	22.72523021	-8.274560306	2.403249548	-3.443071617	0.000575147
TRINITY_DN81337_c0_g4_i5	34.17517911	8.1610222	2.37063677	3.442544342	0.000576269
TRINITY_DN80669_c1_g1_i5	66.62485368	-5.854465265	1.700576561	-3.442635515	0.000576075
TRINITY_DN77445_c1_g1_i5	57.55560809	4.234168746	1.230083708	3.442179356	0.000577048
TRINITY_DN87340_c1_g7_i1	103.6947534	5.8807138	1.708698863	3.441632652	0.000578215
TRINITY_DN82344_c0_g2_i1	23.61596933	8.418459845	2.446478376	3.44105222	0.000579457
TRINITY_DN84929_c4_g1_i1	362.1381038	1.399852829	0.406846997	3.440735315	0.000580136
TRINITY_DN84420_c0_g1_i1	422.439079	1.639335072	0.476539592	3.440081576	0.000581539
TRINITY_DN75611_c1_g1_i1	83.04412996	-2.173059543	0.631671294	-3.440174601	0.000581339
TRINITY_DN86961_c2_g7_i2	26.67672423	-6.095916115	1.772356728	-3.43944084	0.000582917
TRINITY_DN80336_c2_g1_i1	15.75821009	-6.712873876	1.951757032	-3.439400379	0.000583004
TRINITY_DN79301_c3_g7_i1	400.5732114	1.500142548	0.436174684	3.43931595	0.000583186
TRINITY_DN87688_c1_g2_i3	84.6445331	8.157424016	2.372154156	3.438825422	0.000584244
TRINITY_DN87178_c0_g4_i4	112.4120357	5.560080828	1.61679647	3.438949139	0.000583977
TRINITY_DN81683_c2_g1_i1	477.0574393	1.550153789	0.450861266	3.438205732	0.000585583
TRINITY_DN86064_c1_g1_i8	19.55997017	6.985382683	2.03161092	3.43834669	0.000585278
TRINITY_DN80713_c0_g3_i8	1091.210068	1.288323916	0.374753208	3.437792899	0.000586476
TRINITY_DN86279_c0_g1_i1	223.7965674	5.29875405	1.541543227	3.437304876	0.000587534
TRINITY_DN88724_c3_g1_i6	448.8168463	-1.726748636	0.502389523	-3.437071349	0.000588041
TRINITY_DN83215_c0_g1_i7	242.6641085	2.626101148	0.764285682	3.43602034	0.000590327
TRINITY_DN83670_c2_g2_i4	16.31207211	3.745080973	1.089880504	3.436230815	0.000589868
TRINITY_DN84960_c0_g1_i1	17.99641281	6.732065352	1.959402551	3.435774516	0.000590863
TRINITY_DN88794_c5_g1_i1	89.74524389	3.677622329	1.070350867	3.435903535	0.000590581
TRINITY_DN86126_c1_g5_i1	4511.576974	1.121215813	0.326445936	3.434614099	0.000593398
TRINITY_DN79703_c0_g3_i1	326.4264117	-1.941555439	0.565348565	-3.434262613	0.000594168
TRINITY_DN85949_c1_g1_i1	9.664635462	-6.774270868	1.973057364	-3.433387691	0.000596089
TRINITY_DN86881_c0_g2_i8	13.58920465	-6.492901322	1.891727956	-3.432259539	0.000598574
TRINITY_DN78488_c2_g3_i1	21.32700725	6.514076702	1.897989037	3.432093955	0.00059894
TRINITY_DN78805_c0_g1_i1	59.4664157	-8.041960211	2.343292788	-3.431905842	0.000599356
TRINITY_DN86391_c2_g2_i7	20.23261427	-8.439727486	2.461288816	-3.428987054	0.000605838
TRINITY_DN65813_c0_g1_i2	35.26971379	9.767159876	2.848319623	3.429095456	0.000605596
TRINITY_DN84626_c2_g2_i4	72.74719695	16.48862777	4.808731496	3.428893417	0.000606047
TRINITY_DN77778_c2_g1_i2	20.52834308	7.509506231	2.190747358	3.4278285	0.00060843
TRINITY_DN80454_c1_g1_i9	10.47780703	-7.026277846	2.049812114	-3.427766768	0.000608568
TRINITY_DN80295_c0_g2_i4	19.30659412	-4.681013179	1.365857267	-3.427161309	0.000609927
TRINITY_DN88417_c2_g2_i1	11.32530105	7.954335049	2.321036515	3.427061573	0.000610151
TRINITY_DN84650_c1_g1_i1	14.30996931	-4.456537267	1.300749916	-3.426129197	0.000612249
TRINITY_DN86474_c1_g1_i5	19.2777537	8.845287946	2.581845127	3.425956055	0.000612639
TRINITY_DN87648_c0_g2_i1	16.28004719	-8.512715282	2.48612924	-3.424083971	0.000616876
TRINITY_DN81886_c0_g1_i5	15.00564971	-6.813632326	1.990026084	-3.423890964	0.000617314
TRINITY_DN86157_c0_g2_i2	28.41944908	6.082741649	1.776601751	3.423807077	0.000617504
TRINITY_DN82101_c3_g1_i3	85.59981561	5.883726706	1.71875755	3.423244137	0.000618785
TRINITY_DN77865_c2_g8_i1	56.55186685	8.016956155	2.341337978	3.424091792	0.000616858
TRINITY_DN86422_c0_g4_i3	11.06706428	7.232030542	2.112578798	3.423318717	0.000618615
TRINITY_DN78996_c1_g5_i1	617.2054468	2.735674993	0.799046077	3.423676145	0.000617802
TRINITY_DN84775_c0_g5_i1	70.22997632	7.810867668	2.281548397	3.423494185	0.000618216
TRINITY_DN76413_c0_g1_i3	70.4208444	3.326933635	0.971914587	3.423072028	0.000619177
TRINITY_DN84601_c0_g1_i5	62.4320059	-7.976747356	2.330804732	-3.422314726	0.000620904
TRINITY_DN85470_c0_g2_i3	29.45729976	-5.861889767	1.713084985	-3.421832435	0.000622006
TRINITY_DN85743_c0_g1_i4	10.57981849	6.688472485	1.954923465	3.421347486	0.000623117
TRINITY_DN88445_c0_g1_i6	37.61581866	-5.808657637	1.698196483	-3.420486201	0.000625093
TRINITY_DN87730_c1_g1_i1	224.3799364	3.955912902	1.156844948	3.419570539	0.000627201

TRINITY_DN82984_c2_g1_i2	31.18854629	-4.082861263	1.193934116	-3.419670489	0.00062697
TRINITY_DN76957_c0_g2_i6	31.40245087	-2.935504355	0.858717203	-3.418476241	0.000629728
TRINITY_DN83589_c0_g1_i1	21.03836766	8.15951917	2.387007648	3.418304578	0.000630125
TRINITY_DN85976_c0_g2_i2	12.14592451	-6.571559158	1.922827141	-3.417654669	0.000631632
TRINITY_DN87455_c1_g2_i3	13.79780713	-6.805770974	1.992195049	-3.416217191	0.000634976
TRINITY_DN81875_c6_g5_i2	3918.834057	1.51072389	0.442233438	3.416123159	0.000635195
TRINITY_DN75409_c0_g1_i6	60.81870566	7.97233323	2.333695162	3.416184495	0.000635052
TRINITY_DN81985_c0_g1_i1	30.48830024	2.922580126	0.855720736	3.415343352	0.000637017
TRINITY_DN87090_c0_g1_i3	18.81224312	8.187825151	2.398722415	3.413410865	0.000641551
TRINITY_DN87676_c0_g4_i2	21.01471796	-6.15703631	1.803944246	-3.413096788	0.000642291
TRINITY_DN87406_c1_g2_i2	116.6489728	1.675312912	0.490668899	3.414345019	0.000639356
TRINITY_DN79848_c3_g1_i1	27.88748615	6.365874466	1.864516993	3.414221748	0.000639645
TRINITY_DN68159_c0_g1_i2	725.8561917	1.854114895	0.543244897	3.413036928	0.000642432
TRINITY_DN83769_c0_g2_i1	32.81316301	-4.558498407	1.335333001	-3.413754024	0.000640744
TRINITY_DN81010_c0_g1_i1	35.21690311	5.808551692	1.701851622	3.413077625	0.000642336
TRINITY_DN81885_c3_g4_i2	167.2728625	5.95078801	1.743461458	3.413203076	0.000642041
TRINITY_DN87712_c0_g2_i6	34.95570643	-6.476679482	1.897309085	-3.413613277	0.000641075
TRINITY_DN87190_c4_g2_i2	17.25122326	-8.103417938	2.37405091	-3.413329471	0.000641743
TRINITY_DN81384_c1_g1_i8	26.08962611	-8.521646321	2.496812781	-3.413009733	0.000642496
TRINITY_DN84984_c0_g1_i6	535.7356418	5.467272385	1.601867623	3.413061296	0.000642375
TRINITY_DN86856_c0_g1_i9	17.60450822	6.366408089	1.865660098	3.412415849	0.000643898
TRINITY_DN81293_c0_g1_i1	34.0166023	-6.443673384	1.888813192	-3.411493213	0.000646081
TRINITY_DN82535_c0_g5_i4	160.2264915	-3.708902298	1.087299827	-3.411112747	0.000646983
TRINITY_DN87745_c0_g1_i2	31.74186795	9.500840285	2.785217882	3.411165908	0.000646857
TRINITY_DN84123_c3_g1_i2	36.6551254	8.820337568	2.586287475	3.410424268	0.000648619
TRINITY_DN84904_c2_g2_i8	724.1823791	2.287882756	0.671330634	3.407982058	0.000654452
TRINITY_DN84176_c2_g1_i6	35.18096245	-3.912837157	1.148100312	-3.408096936	0.000654177
TRINITY_DN83323_c1_g2_i1	132.2609567	5.561160387	1.63225445	3.407042564	0.000656709
TRINITY_DN85095_c0_g2_i1	12.9524259	-7.811535457	2.293263428	-3.406296617	0.000658506
TRINITY_DN85491_c2_g3_i9	85.8202414	2.992430441	0.878499086	3.406298866	0.000658501
TRINITY_DN83033_c0_g2_i6	53.16293701	8.249446731	2.421968701	3.406091387	0.000659001
TRINITY_DN83449_c1_g1_i1	36.57167002	-5.905241965	1.734145377	-3.405275038	0.000660974
TRINITY_DN84782_c0_g1_i5	14.54641823	-7.132066225	2.094495852	-3.40514698	0.000661285
TRINITY_DN88570_c1_g1_i6	927.0595084	1.704130771	0.50063065	3.403968117	0.000664145
TRINITY_DN82744_c2_g1_i3	13.00528832	-6.487602185	1.906295169	-3.403251653	0.000665889
TRINITY_DN79900_c2_g1_i1	19.90492143	8.355030935	2.455783832	3.402185008	0.000668494
TRINITY_DN88572_c3_g5_i3	17.90436362	-6.065823864	1.78365049	-3.400791745	0.00067191
TRINITY_DN88504_c0_g1_i3	24.33201046	6.516188979	1.916372591	3.400272477	0.000673187
TRINITY_DN81829_c1_g1_i3	24.21862161	6.732355086	1.980509561	3.399304512	0.000675575
TRINITY_DN87611_c1_g2_i1	14.05444965	6.514402933	1.916495755	3.399122026	0.000676025
TRINITY_DN87745_c0_g1_i1	244.4814853	7.142557545	2.101655389	3.398538877	0.000677468
TRINITY_DN81412_c0_g1_i2	10.4928866	7.51551253	2.211620066	3.398193317	0.000678325
TRINITY_DN85230_c1_g1_i2	11.16886547	7.987112246	2.350392989	3.398202889	0.000678301
TRINITY_DN85482_c0_g1_i8	23.25645766	6.020141308	1.771597118	3.398143544	0.000678448
TRINITY_DN81317_c1_g1_i1	78.56906728	2.435277527	0.716584463	3.398451476	0.000677685
TRINITY_DN81576_c1_g3_i5	1868.222718	1.764944964	0.519447707	3.397733673	0.000679465
TRINITY_DN83285_c1_g4_i2	13.42177866	6.204247088	1.826219909	3.397316532	0.000680502
TRINITY_DN78767_c0_g1_i1	17.52855674	-7.545113801	2.221098041	-3.397019701	0.000681241
TRINITY_DN77418_c0_g1_i7	16.40830699	6.145453477	1.809169838	3.396836133	0.000681698
TRINITY_DN85294_c1_g1_i2	58.45595777	-6.258696487	1.842573391	-3.396714897	0.000682
TRINITY_DN82283_c0_g1_i1	17.93651135	6.369058767	1.875350425	3.396196615	0.000683293
TRINITY_DN77944_c2_g3_i2	10.35457232	-6.678687189	1.966703581	-3.395878897	0.000684086
TRINITY_DN78875_c2_g1_i1	12.50546623	-7.553023489	2.224769403	-3.39496915	0.000686363
TRINITY_DN86530_c0_g2_i4	26.69715683	9.365426239	2.758872989	3.394656541	0.000687147
TRINITY_DN87232_c0_g1_i4	72.41860663	7.686827825	2.264462424	3.394548632	0.000687418
TRINITY_DN87614_c1_g3_i7	8.706936326	-6.804735068	2.005514764	-3.393011705	0.000691287
TRINITY_DN75342_c0_g1_i2	13.86036378	-6.381224915	1.880738624	-3.392935538	0.000691479

TRINITY_DN84131_c2_g2_i1	16.61923098	-6.214669607	1.832176584	-3.391959957	0.000693946
TRINITY_DN83364_c0_g1_i2	11.40800966	-6.735732417	1.986289124	-3.391113778	0.000696092
TRINITY_DN83552_c0_g3_i1	196.4134665	1.93561863	0.570803025	3.391044802	0.000696267
TRINITY_DN83369_c1_g1_i2	28.51216113	5.95185815	1.755255576	3.390878361	0.00069669
TRINITY_DN83505_c0_g1_i1	12.42463745	-6.487651063	1.913689888	-3.390126636	0.000698603
TRINITY_DN82230_c4_g1_i9	30.45082583	-5.730919754	1.690431964	-3.390210239	0.00069839
TRINITY_DN82641_c0_g2_i1	21.51337607	-3.097175156	0.913701873	-3.389699909	0.000699692
TRINITY_DN77456_c2_g4_i7	275.7400461	1.993982851	0.588393039	3.388862069	0.000701833
TRINITY_DN83713_c0_g1_i1	10.80086471	-6.387182456	1.884906946	-3.388592986	0.000702522
TRINITY_DN79061_c1_g1_i1	108.9815478	8.049363272	2.375662458	3.388260502	0.000703374
TRINITY_DN86808_c2_g1_i1	13.56576319	-7.141794561	2.107968771	-3.387998275	0.000704047
TRINITY_DN85640_c1_g2_i3	25.20436571	2.915066403	0.860745744	3.386675361	0.00070745
TRINITY_DN88533_c0_g4_i9	10.95499723	-7.278187329	2.149456772	-3.386058945	0.000709042
TRINITY_DN81078_c1_g2_i1	12.50266253	7.342071177	2.168328687	3.386050842	0.000709062
TRINITY_DN86115_c1_g1_i1	19.87181063	-6.182740083	1.826156819	-3.385656707	0.000710082
TRINITY_DN78509_c1_g3_i5	20.06640921	-8.835827322	2.610017142	-3.385352218	0.00071087
TRINITY_DN79826_c3_g3_i7	95.43786762	-7.617380534	2.250372607	-3.384941902	0.000711933
TRINITY_DN85848_c0_g1_i1	11.79594228	-7.420309812	2.192366708	-3.38461161	0.00071279
TRINITY_DN82177_c1_g4_i2	13.88893766	-6.621262784	1.95619448	-3.384767135	0.000712387
TRINITY_DN83368_c2_g3_i1	65.99728791	8.623571816	2.548176104	3.384213438	0.000713825
TRINITY_DN83160_c0_g3_i2	151.6190018	7.285498242	2.152905571	3.384030558	0.000714301
TRINITY_DN86457_c1_g5_i3	249.6118087	1.44178074	0.426058813	3.383994643	0.000714394
TRINITY_DN85901_c0_g1_i1	14.55515801	-6.681751082	1.975534647	-3.382249505	0.000718948
TRINITY_DN82629_c4_g1_i3	233.4535137	1.406290018	0.415776553	3.382321605	0.000718759
TRINITY_DN88542_c0_g3_i1	49.14505606	5.881335683	1.739318843	3.381401695	0.00072117
TRINITY_DN86868_c3_g2_i1	43.95471208	5.731127705	1.695178623	3.380840006	0.000722646
TRINITY_DN78209_c1_g1_i1	25.3375926	6.6874259	1.978032995	3.380846485	0.000722629
TRINITY_DN85849_c4_g1_i7	602.4988739	1.22590528	0.362647095	3.380435954	0.000723709
TRINITY_DN81773_c0_g1_i1	22.25849845	-4.595735129	1.359901542	-3.379461665	0.000726279
TRINITY_DN84842_c0_g2_i5	12.61737719	-6.29817238	1.863668094	-3.379449591	0.000726311
TRINITY_DN88181_c1_g2_i1	28.04065104	-6.187610834	1.831357063	-3.37870258	0.000728288
TRINITY_DN81596_c0_g3_i1	71.00897438	2.276665455	0.673924298	3.378221355	0.000729563
TRINITY_DN88034_c2_g1_i1	54.48620374	-6.875918668	2.035672677	-3.377713296	0.000730912
TRINITY_DN86993_c1_g5_i2	776.4814758	-6.810336752	2.016983102	-3.376496682	0.000734153
TRINITY_DN84745_c0_g2_i1	9.45104797	-6.5138765	1.929431226	-3.376060474	0.000735318
TRINITY_DN79572_c2_g3_i8	30.31652697	5.730676051	1.697620584	3.375710748	0.000736253
TRINITY_DN87512_c0_g1_i1	127.5969109	5.367842216	1.5903818	3.375190924	0.000737645
TRINITY_DN77859_c3_g4_i1	26.43549756	6.560440112	1.943735534	3.375171157	0.000737698
TRINITY_DN83837_c1_g1_i1	13.97470481	-7.00555307	2.075733987	-3.374976328	0.00073822
TRINITY_DN85744_c2_g1_i7	125.0108434	-7.47067221	2.214052483	-3.374207372	0.000740286
TRINITY_DN83179_c1_g4_i2	8.501889309	-7.456216039	2.210169839	-3.37359415	0.000741937
TRINITY_DN79046_c2_g1_i1	51.74067789	3.132709501	0.928719917	3.373147753	0.000743141
TRINITY_DN85185_c1_g1_i8	733.3719205	-6.917103626	2.050438823	-3.373474764	0.000742258
TRINITY_DN88096_c1_g4_i2	18.95242481	3.43543092	1.018233818	3.373911628	0.000741082
TRINITY_DN85836_c0_g3_i8	10.2055397	-6.566527752	1.946492757	-3.373517692	0.000742143
TRINITY_DN80729_c0_g2_i3	48.15145936	3.799828002	1.126533077	3.373028347	0.000743463
TRINITY_DN87108_c2_g2_i1	374.0388537	2.807082427	0.832223751	3.372990044	0.000743566
TRINITY_DN86040_c0_g1_i7	69.00404996	6.14524274	1.822409283	3.37204315	0.000746128
TRINITY_DN82779_c1_g1_i5	166.0763066	7.258018585	2.152843893	3.371363157	0.000747972
TRINITY_DN88684_c2_g2_i1	464.3169814	-1.916270155	0.568442014	-3.371091701	0.000748709
TRINITY_DN82692_c1_g1_i1	32.10713519	-8.127693583	2.411008588	-3.371076164	0.000748752
TRINITY_DN87428_c7_g4_i3	12031.14292	1.434101825	0.425480914	3.370543256	0.000750201
TRINITY_DN84717_c0_g1_i1	23.51260664	-5.939350599	1.762184508	-3.37044763	0.000750462
TRINITY_DN81367_c0_g2_i2	10.30217263	-6.6786871	1.982174282	-3.369374309	0.00075339
TRINITY_DN77748_c5_g2_i2	256.5327367	1.901206282	0.564261685	3.369369804	0.000753403
TRINITY_DN79320_c0_g11_i	87.88212789	-7.631760675	2.26563691	-3.368483556	0.000755829
TRINITY_DN84112_c1_g1_i2	10.83727233	-6.73743228	1.999868448	-3.368937734	0.000754585

TRINITY_DN81294_c0_g1_i2	12.62074518	-6.581390702	1.953788547	-3.368527629	0.000755708
TRINITY_DN78406_c0_g1_i7	160.5250506	1.408260454	0.418035778	3.368755804	0.000755083
TRINITY_DN78084_c1_g1_i4	51.56413995	-2.771393181	0.822843501	-3.368068384	0.000756968
TRINITY_DN88193_c1_g6_i5	100.0995169	-2.528381526	0.750722085	-3.36793279	0.000757341
TRINITY_DN85717_c0_g2_i6	22.99441896	-8.954399176	2.658561842	-3.368136499	0.000756781
TRINITY_DN83178_c1_g1_i7	149.3937726	3.84679109	1.14224793	3.367737414	0.000757877
TRINITY_DN88009_c0_g1_i4	110.6702825	-7.577252816	2.250636258	-3.366715874	0.00076069
TRINITY_DN83077_c4_g1_i2	47.71987298	-2.723257304	0.809065157	-3.365930767	0.000762859
TRINITY_DN77329_c4_g4_i1	15.14831387	7.934893444	2.357468777	3.365853038	0.000763074
TRINITY_DN86573_c2_g1_i3	681.1807133	1.28904013	0.382955139	3.366034293	0.000762572
TRINITY_DN78125_c0_g4_i5	23.92271819	9.106168848	2.705863217	3.365347069	0.000764474
TRINITY_DN77739_c2_g1_i1	11.43194567	6.880641709	2.045152097	3.364366747	0.000767195
TRINITY_DN84787_c0_g1_i3	35.70782296	2.729360169	0.811211565	3.3645479	0.000766692
TRINITY_DN81879_c0_g2_i1	19.36392048	-7.750244778	2.303532365	-3.364504401	0.000766813
TRINITY_DN87504_c1_g1_i1	13.27632584	-6.644508688	1.97465068	-3.36490335	0.000765705
TRINITY_DN84242_c1_g2_i4	167.5877672	-7.200795877	2.140171069	-3.364588925	0.000766578
TRINITY_DN86507_c1_g1_i4	77.41348429	7.540207911	2.24135043	3.364136107	0.000767837
TRINITY_DN82384_c0_g2_i6	11.35282452	6.690968735	1.98899953	3.363987086	0.000768251
TRINITY_DN83895_c1_g1_i2	14.46620843	8.142616877	2.421111822	3.363172574	0.000770522
TRINITY_DN82660_c2_g3_i6	27.8345215	-6.171398975	1.834930987	-3.363286695	0.000770203
TRINITY_DN86989_c0_g1_i7	279.101369	-1.697044179	0.50463671	-3.362902745	0.000771275
TRINITY_DN83010_c0_g1_i5	18.77483884	-6.508043901	1.935417441	-3.36260476	0.000772108
TRINITY_DN85155_c0_g1_i1	151.5174774	-5.311554776	1.580003302	-3.361736504	0.00077454
TRINITY_DN81165_c2_g1_i6	16.71170873	-4.963468439	1.476503259	-3.361637308	0.000774818
TRINITY_DN83817_c3_g2_i7	70.01874897	5.647868884	1.680497654	3.360831162	0.000777083
TRINITY_DN79884_c5_g2_i3	439.3070943	1.362445517	0.405413568	3.360631277	0.000777646
TRINITY_DN77832_c0_g1_i6	20.41032787	-8.860192867	2.636785044	-3.360225699	0.000778788
TRINITY_DN78316_c0_g1_i1	49.18101159	7.902797377	2.352395442	3.359468071	0.000780927
TRINITY_DN83725_c2_g2_i1	21.47731503	-7.325979121	2.180800609	-3.35930717	0.000781382
TRINITY_DN84863_c1_g1_i2	29.35201328	-3.350421428	0.997696028	-3.358158533	0.000784636
TRINITY_DN79379_c0_g1_i4	14.41864657	6.770085761	2.016096321	3.358017021	0.000785038
TRINITY_DN87807_c1_g2_i6	28.67617549	-3.936684863	1.172423657	-3.357732367	0.000785847
TRINITY_DN85491_c1_g1_i1	8.610418145	-7.426302633	2.211739289	-3.357675414	0.000786009
TRINITY_DN84529_c1_g1_i5	237.8361423	6.989142642	2.082011324	3.356918649	0.000788163
TRINITY_DN74508_c0_g1_i3	12.39926299	6.315270688	1.881512198	3.35648671	0.000789395
TRINITY_DN78598_c0_g1_i4	30.8399595	-3.369360905	1.004523896	-3.354186913	0.000795986
TRINITY_DN81578_c0_g1_i4	14.69753374	-6.332672863	1.888236285	-3.353750224	0.000797243
TRINITY_DN81655_c1_g1_i2	258.7057629	6.08167227	1.813551903	3.353459176	0.000798082
TRINITY_DN78697_c1_g5_i1	379.3373433	1.460537608	0.435575343	3.353122785	0.000799053
TRINITY_DN83666_c4_g2_i1	11.97215683	6.26092121	1.867339339	3.352856699	0.000799821
TRINITY_DN87981_c1_g7_i1	31.82590778	5.882887478	1.754627213	3.352784817	0.000800029
TRINITY_DN85274_c2_g4_i1	117.163694	4.617153668	1.377204553	3.352554752	0.000800694
TRINITY_DN88059_c1_g2_i2	55.15507065	7.966912835	2.376666201	3.352137895	0.000801901
TRINITY_DN84448_c2_g1_i3	8.716273042	-6.705501615	2.00073879	-3.351512775	0.000803713
TRINITY_DN88249_c1_g2_i1	28.03220744	9.408289912	2.807728609	3.350854453	0.000805626
TRINITY_DN80297_c2_g2_i1	2802.611269	1.914271366	0.571323262	3.350592377	0.000806389
TRINITY_DN81332_c1_g4_i2	170.8727992	2.055180882	0.613553179	3.349637739	0.000809173
TRINITY_DN88159_c0_g2_i1	127.4529618	-7.333834487	2.189487751	-3.349566347	0.000809382
TRINITY_DN77185_c0_g1_i5	20.92411827	-8.47064421	2.529148622	-3.349207768	0.00081043
TRINITY_DN86869_c0_g1_i1	35.04031015	8.390392361	2.505604033	3.348650565	0.000812061
TRINITY_DN78834_c1_g1_i2	1927.265791	6.557688075	1.95850115	3.348319747	0.000813031
TRINITY_DN83909_c2_g1_i1	27.57875951	3.937209749	1.176000534	3.347965953	0.00081407
TRINITY_DN88590_c3_g1_i3	225.7577322	1.697245755	0.507040079	3.347360151	0.000815851
TRINITY_DN88108_c1_g1_i6	15.35729228	-7.369086927	2.201472751	-3.347344147	0.000815899
TRINITY_DN85137_c2_g2_i1	2070.649415	1.421225329	0.424737102	3.34612946	0.000819481
TRINITY_DN84322_c1_g5_i1	8.379441372	7.202757246	2.152217189	3.346668395	0.00081789
TRINITY_DN85244_c1_g2_i8	155.2480669	-7.259621522	2.169348642	-3.346452193	0.000818528

TRINITY_DN80884_c1_g3_i5	64.77997378	-8.267660727	2.470644592	-3.34635777	0.000818807
TRINITY_DN84741_c0_g3_i3	724.2984892	2.313045591	0.691227716	3.346285944	0.000819019
TRINITY_DN70413_c0_g1_i2	11.45809897	-7.739113213	2.313225553	-3.345593863	0.000821066
TRINITY_DN82220_c4_g2_i5	141.4296385	7.117485206	2.127711784	3.345135963	0.000822422
TRINITY_DN83111_c0_g1_i2	557.790097	4.227358153	1.264494731	3.343120417	0.000828419
TRINITY_DN87272_c0_g1_i1	32.40769852	3.809315299	1.139438946	3.343149989	0.000828331
TRINITY_DN86183_c3_g1_i3	14.2535282	6.084371689	1.820310529	3.34249107	0.0008303
TRINITY_DN82018_c0_g2_i3	21.56495773	-8.873678618	2.654818812	-3.342479938	0.000830334
TRINITY_DN87613_c5_g1_i4	1388.409813	2.578922293	0.77168547	3.341934498	0.000831967
TRINITY_DN83028_c0_g2_i7	16.42492533	8.129622684	2.433485322	3.340732163	0.000835578
TRINITY_DN83105_c0_g3_i3	13.42774281	6.260921006	1.874548218	3.339962636	0.000837897
TRINITY_DN81951_c0_g1_i7	8.369583211	6.689056462	2.003083845	3.339379168	0.000839659
TRINITY_DN84132_c2_g3_i1	1186.237549	1.147000227	0.343479143	3.339359171	0.000839719
TRINITY_DN81288_c4_g2_i8	100.6709828	5.466609081	1.636934678	3.339540151	0.000839172
TRINITY_DN76994_c1_g1_i6	41.11550404	5.648344249	1.69114812	3.339946503	0.000837945
TRINITY_DN87825_c0_g4_i3	100.573063	7.897367761	2.364857244	3.339469129	0.000839387
TRINITY_DN88127_c1_g1_i3	10.3337993	6.560289513	1.965028676	3.338521006	0.000842257
TRINITY_DN85057_c0_g1_i2	16.24212525	8.615906958	2.581015672	3.338184673	0.000843277
TRINITY_DN84929_c4_g1_i7	71.68935176	3.059922582	0.916724876	3.337885404	0.000844186
TRINITY_DN79407_c1_g2_i8	20.20565418	-8.845645674	2.650953446	-3.336778956	0.000847553
TRINITY_DN81395_c0_g1_i1	40.60879047	7.9892109	2.394429622	3.336582051	0.000848154
TRINITY_DN87820_c1_g1_i8	9.242832796	6.688906959	2.004952162	3.336192796	0.000849342
TRINITY_DN81196_c0_g3_i1	731.5669185	1.180819415	0.35397787	3.335856605	0.00085037
TRINITY_DN79786_c0_g2_i7	19.0995387	8.88227921	2.663059796	3.335366041	0.000851872
TRINITY_DN85514_c1_g1_i3	15.28177966	6.468357573	1.939545649	3.334985993	0.000853037
TRINITY_DN79632_c0_g1_i2	37.96125761	6.084983979	1.824790414	3.33462075	0.000854158
TRINITY_DN87006_c0_g5_i2	16.0900361	7.114372111	2.134189251	3.333524478	0.000857531
TRINITY_DN79500_c2_g2_i2	28.10887294	-8.150555183	2.445373042	-3.333051867	0.000858989
TRINITY_DN78848_c1_g1_i8	90.13871733	4.099018994	1.229758084	3.33319134	0.000858559
TRINITY_DN78887_c2_g3_i6	19.77115516	7.232953274	2.170366094	3.332595959	0.000860398
TRINITY_DN77439_c1_g1_i1	19.65973921	4.758537887	1.428163003	3.331929113	0.000862462
TRINITY_DN80517_c0_g1_i4	23.56151348	-6.310129949	1.893750686	-3.332080615	0.000861993
TRINITY_DN78778_c1_g2_i2	329.3074761	2.177215596	0.653469013	3.331780929	0.000862922
TRINITY_DN78347_c0_g1_i3	73.2854341	2.214692524	0.664801121	3.331360994	0.000864224
TRINITY_DN86658_c0_g1_i7	11.79156572	-7.017353482	2.106987252	-3.330515396	0.000866854
TRINITY_DN78632_c4_g2_i1	19.77126248	5.808338095	1.744270225	3.32995313	0.000868606
TRINITY_DN80886_c1_g1_i4	13.0139854	-6.189254505	1.858752245	-3.329789928	0.000869115
TRINITY_DN86127_c1_g2_i6	142.0529465	2.384623429	0.716218369	3.329464215	0.000870132
TRINITY_DN87242_c1_g1_i1	34.4195958	8.46438769	2.54252005	3.329133113	0.000871168
TRINITY_DN87008_c0_g4_i3	29.15809468	-6.031113397	1.811798122	-3.328799895	0.00087221
TRINITY_DN72088_c0_g1_i2	144.4764214	2.463489902	0.740398811	3.327247244	0.000877085
TRINITY_DN87170_c1_g1_i1	15.85914293	-5.161381884	1.551456789	-3.326797061	0.000878503
TRINITY_DN85342_c4_g3_i1	1663.183891	1.618661947	0.486619485	3.326340182	0.000879945
TRINITY_DN194941_c0_g1_i	1052.784219	2.208392925	0.663928478	3.326251244	0.000880225
TRINITY_DN86881_c0_g2_i1	29.65445222	5.730688312	1.723012699	3.325969863	0.000881115
TRINITY_DN87065_c1_g1_i1	22.55525472	3.293616034	0.99054974	3.325038513	0.000884063
TRINITY_DN80385_c0_g1_i3	28.41202699	7.70836518	2.318542293	3.324660155	0.000885264

:rop trees

Alireza Salami [6]

es ON- *vs.* OFF-trees

padj
1.10207E-15
2.23539E-13
1.16116E-11
3.74185E-10
4.97367E-10
1.73017E-09
5.78249E-09
1.0922E-08
1.93949E-08
2.0516E-08
2.72025E-08
3.76911E-08
6.72031E-08
1.26127E-07
1.54446E-07
2.34484E-07
2.34484E-07
2.52403E-07
5.66853E-07
6.84191E-07
6.84191E-07
7.22178E-07
7.28724E-07
7.7898E-07
7.94657E-07
7.94657E-07
7.94657E-07
8.18472E-07
1.63704E-06
1.8345E-06
1.8345E-06
2.20989E-06
2.37134E-06
2.64814E-06
2.75098E-06
2.85908E-06
3.0171E-06
3.4422E-06
3.90701E-06
3.90701E-06
6.19804E-06
6.31117E-06
6.31117E-06
7.15888E-06
7.2513E-06
1.14254E-05
1.16846E-05
1.35007E-05
1.39663E-05
1.53826E-05
1.7554E-05
1.83488E-05

2.02894E-05
2.10225E-05
2.10225E-05
2.10225E-05
2.33256E-05
2.98017E-05
3.02126E-05
3.07535E-05
3.07535E-05
3.07535E-05
3.24334E-05
3.24334E-05
3.32973E-05
3.32973E-05
3.64143E-05
3.67487E-05
3.96578E-05
4.28108E-05
4.34823E-05
4.34823E-05
4.44999E-05
4.79398E-05
4.79398E-05
4.79398E-05
5.35531E-05
5.39019E-05
5.51138E-05
5.93341E-05
6.02322E-05
6.29266E-05
6.42995E-05
6.43719E-05
7.06879E-05
7.06879E-05
7.10726E-05
7.50463E-05
7.75237E-05
8.70836E-05
8.81602E-05
8.81602E-05
9.42365E-05
9.42365E-05
9.62748E-05
0.000100491
0.00010724
0.00011065
0.000112406
0.000125633
0.000125663
0.000125663
0.000134441
0.000134441
0.000142266
0.000142266
0.000155067
0.000157821
0.000157821

0.000157821
0.000157821
0.000165075
0.000165075
0.000168398
0.000169435
0.000170396
0.000172143
0.000175401
0.000186349
0.00019083
0.000192116
0.000200322
0.000200322
0.00020569
0.000211196
0.000220517
0.000222534
0.000226042
0.000227151
0.000233123
0.000233123
0.000233123
0.000250841
0.000253385
0.00025798
0.000258986
0.000258986
0.000263589
0.000264101
0.00026857
0.000279582
0.000279582
0.000280352
0.000290752
0.000293064
0.000293198
0.000293626
0.00031165
0.000316216
0.000316216
0.000325448
0.000339407
0.000339407
0.000346302
0.000357312
0.000360875
0.000374121
0.000374121
0.000374121
0.000374121
0.000377161
0.000390522
0.000393531
0.000393531
0.000395047
0.000405519

0.000405519
0.000406608
0.000412188
0.000412333
0.000415811
0.000422547
0.000435635
0.000436517
0.00045921
0.000461018
0.000465708
0.000466551
0.000473729
0.000481074
0.000490016
0.000494263
0.000494263
0.000502053
0.000506645
0.000506645
0.000512942
0.000518363
0.000538095
0.000538095
0.000538095
0.000547093
0.000548345
0.000552971
0.000552971
0.000555088
0.000555088
0.000581675
0.000581947
0.000582407
0.00058403
0.000587643
0.000588684
0.000595123
0.000595123
0.000595123
0.000595123
0.000597398
0.000597398
0.000597398
0.000599843
0.000599843
0.000599843
0.000599843
0.000599843
0.000602039
0.000602039
0.000621593
0.000621637
0.000623071
0.000623071
0.000623071
0.000623071

0.000628505
0.000669622
0.000681403
0.000682293
0.000685211
0.000685211
0.000693739
0.000694235
0.000696318
0.000712205
0.00071886
0.000718953
0.000718953
0.000733665
0.000733665
0.000734713
0.000738385
0.000747494
0.000747962
0.0007482
0.0007482
0.000751964
0.000754018
0.000754018
0.000754018
0.000759482
0.00078521
0.00078521
0.00078521
0.000792986
0.000799186
0.000818726
0.000818726
0.000820493
0.000825559
0.000825559
0.000834237
0.000843098
0.000853699
0.000865154
0.0008946
0.000911664
0.000912113
0.000912113
0.000972941
0.000979602
0.001005589
0.001017193
0.001023169
0.00102864
0.00102864
0.001050873
0.001064284
0.001066797
0.001071652
0.001090927
0.001090927

0.001090927
0.001106673
0.001106673
0.001121001
0.001121001
0.001121001
0.001126117
0.001126117
0.001126117
0.001126117
0.001126117
0.001126117
0.001133949
0.001139656
0.001139656
0.001153027
0.001157182
0.001192488
0.001192488
0.001192488
0.001200793
0.00121398
0.001244033
0.001250419
0.001256948
0.001272476
0.001280944
0.001315814
0.001315814
0.001317573
0.001317573
0.001317573
0.00133487
0.001348624
0.00136021
0.001365075
0.001380642
0.001403632
0.001403632
0.00142843
0.001446752
0.001450474
0.001468035
0.001468035
0.001468035
0.001468035
0.001479123
0.001479123
0.001546291
0.001548257
0.001561655
0.001571982
0.00159512
0.001602579
0.001602579
0.001614453
0.001614453

0.001617348
0.001619642
0.001620444
0.001621219
0.001629994
0.001661128
0.001687991
0.001693544
0.001712312
0.001717791
0.001722663
0.001729656
0.001730678
0.001730678
0.001732069
0.001746
0.001801471
0.001811497
0.001821145
0.001830057
0.00183338
0.00183338
0.001842139
0.001854742
0.001866189
0.001889615
0.001914854
0.001987365
0.001987365
0.001987365
0.001987365
0.001988737
0.00200121
0.002034333
0.002034484
0.002051155
0.002055442
0.002056003
0.002076377
0.002114328
0.002130076
0.002142437
0.002142437
0.002142437
0.002155004
0.002179003
0.002196825
0.002211645
0.002272058
0.002282874
0.002306469
0.002464253
0.002481022
0.002534182
0.002534182
0.002539455
0.002539455

0.002539455
0.002545262
0.002583374
0.002583374
0.002646767
0.002652098
0.002657032
0.002672501
0.002716243
0.002734643
0.002754886
0.002764319
0.002764319
0.002774692
0.002774692
0.002774692
0.002786904
0.002786904
0.002790542
0.002804879
0.002833157
0.002851382
0.002943139
0.002959375
0.002963764
0.002963764
0.002963764
0.002963764
0.002963764
0.002963764
0.002990232
0.002990232
0.002997652
0.002997652
0.002997652
0.002997652
0.002997652
0.002997652
0.002997652
0.002997652
0.002997652
0.002997652
0.002998041
0.003037439
0.003037439
0.003053064
0.003053064
0.003071686
0.003071735
0.003103502
0.003140365
0.003147971
0.003147971
0.003152389
0.003170136
0.00317903
0.003186183

0.003216483
0.003237603
0.003254674
0.0032616
0.003304817
0.003342138
0.003362904
0.003367212
0.003435647
0.003451642
0.003463142
0.003515526
0.003517895
0.003526974
0.003526974
0.003526974
0.003574884
0.003580558
0.003617037
0.003649074
0.003649074
0.003649074
0.003649074
0.003651716
0.003656436
0.00371148
0.003752981
0.003765341
0.00378579
0.003839961
0.003854074
0.00390575
0.003926147
0.003926147
0.003926147
0.004043854
0.004067021
0.004067021
0.004072575
0.004072575
0.004097087
0.00418759
0.00419264
0.004209917
0.004210891
0.004231827
0.004231827
0.004277116
0.004280018
0.0042851
0.0042851
0.004294858
0.004315484
0.004315484
0.004315484
0.004340426
0.004362027

0.004362027
0.004362027
0.004368279
0.004376842
0.004456502
0.004476136
0.004476136
0.004493448
0.004574262
0.004574262
0.00458605
0.004616461
0.004616461
0.004638188
0.004691297
0.004751172
0.004796047
0.004796047
0.004810422
0.004810422
0.004825093
0.004868362
0.004906226
0.004933435
0.004933435
0.004958728
0.004965684
0.00504249
0.005052237
0.005081698
0.005081698
0.005081698
0.00508875
0.00508875
0.00508875
0.00508875
0.005120853
0.005128053
0.005144971
0.005149462
0.005152512
0.005153606
0.005158088
0.005214444
0.005214444
0.005214444
0.005214444
0.005256366
0.005259222
0.005295171
0.005333812
0.005340867
0.005367897
0.005378832
0.005392247
0.005481527
0.005481527

0.005481527
0.005511703
0.005519106
0.005519106
0.005519106
0.005519106
0.005523199
0.005573121
0.005573121
0.00563119
0.005636372
0.005697449
0.005699781
0.005719094
0.00578235
0.00578235
0.005804059
0.005831013
0.005908751
0.005968981
0.006024811
0.006033118
0.006033118
0.006033118
0.006033118
0.006033118
0.006033118
0.006033118
0.006036232
0.006077725
0.006109763
0.006135227
0.006169884
0.006178379
0.006183854
0.006220072
0.006244865
0.006291187
0.006316335
0.006325185
0.006339185
0.006343402
0.006351774
0.006351774
0.006359151
0.00636711
0.006398259
0.006398259
0.006398259
0.006415049
0.006415049
0.006415049
0.006439588
0.006535456
0.006558337
0.006635416
0.006675988

0.006675988
0.006692676
0.006796594
0.006796594
0.006807342
0.006835069
0.006835069
0.006850594
0.006881658
0.00694995
0.00701779
0.007035148
0.007038351
0.007038351
0.007073593
0.007078426
0.007081628
0.007126022
0.007148102
0.007222963
0.007252973
0.007268857
0.007315617
0.007350706
0.007350706
0.007377556
0.007420095
0.007435662
0.007435662
0.007435662
0.007518813
0.007524219
0.007612853
0.007617433
0.007642913
0.007671615
0.007681821
0.007685032
0.007685032
0.007685032
0.007686807
0.007686807
0.007692304
0.007692304
0.007692304
0.007704234
0.007758696
0.007762213
0.007834262
0.00783687
0.007859342
0.007867688
0.00793006
0.00793006
0.00793006
0.007949972
0.007971837

0.007979243
0.007979243
0.00806449
0.008074406
0.008165898
0.008212617
0.008255648
0.008255648
0.008255648
0.008255648
0.008257912
0.008344157
0.008537358
0.008544442
0.008544442
0.008574605
0.008607984
0.008607984
0.008607984
0.008607984
0.008668391
0.008743411
0.008743411
0.008821682
0.008831373
0.008888006
0.008888006
0.008902782
0.008902782
0.008978822
0.008978822
0.009045228
0.009048148
0.009057514
0.009137833
0.009207479
0.009207479
0.009230923
0.009244057
0.009380854
0.00947964
0.009534498
0.009585991
0.009608907
0.009627671
0.009628051
0.009732172
0.009860313
0.009860313
0.009860313
0.009877493
0.009913699
0.009944562
0.010065007
0.010139478
0.010150965
0.010195132

0.010327485
0.01038572
0.010428328
0.010498251
0.010501143
0.010509943
0.010509943
0.010509943
0.010509943
0.010509943
0.010509943
0.010654403
0.010686811
0.01072017
0.010731568
0.010806283
0.010852239
0.010867589
0.010867589
0.010886294
0.010922254
0.010932661
0.010957371
0.010961879
0.010961879
0.011049615
0.011088329
0.011088329
0.011098584
0.011098584
0.011098584
0.011098584
0.011098584
0.011098584
0.011098584
0.011204726
0.011205912
0.011205912
0.011205912
0.011218642
0.011291761
0.011291761
0.011336374
0.01135384
0.011359818
0.011545624
0.011601938
0.011604838
0.011618117
0.011625097
0.011685375
0.011693003
0.011819585
0.011910642
0.011951674
0.011961199
0.011970668

0.012077284
0.01210477
0.012145731
0.012279381
0.012279381
0.012338803
0.012346507
0.012346507
0.012346507
0.012346507
0.012366726
0.012372345
0.012406365
0.0125363
0.012537387
0.012590407
0.012593972
0.012594141
0.012594141
0.012619056
0.01268568
0.01268568
0.012765005
0.012785652
0.012791205
0.012853415
0.013003258
0.013003258
0.013042324
0.013135928
0.013144722
0.013144722
0.013144722
0.013144722
0.013262805
0.013274725
0.013319444
0.01343557
0.013477897
0.013512632
0.013512632
0.013523134
0.013611541
0.01363865
0.013666806
0.013761403
0.013761403
0.013825878
0.0138472
0.0138656
0.013930106
0.013942372
0.014089038
0.01410445
0.01410445
0.01410445
0.014123762

0.014123762
0.014123762
0.014185522
0.014185522
0.014185522
0.014219763
0.014261282
0.014261282
0.014280101
0.01428146
0.014300271
0.014365915
0.014365915
0.014375869
0.014375869
0.014375869
0.014375869
0.014375869
0.014404214
0.014404214
0.014404887
0.014404887
0.014404887
0.014430223
0.014487255
0.014641983
0.014670631
0.014670631
0.014670631
0.014710185
0.014724025
0.014742347
0.014764127
0.014872942
0.014904224
0.014915094
0.01491784
0.01491784
0.0149251
0.014926819
0.015022357
0.015073306
0.015076847
0.015121856
0.015123328
0.015160359
0.015206508
0.015209676
0.015253903
0.015344259
0.015352994
0.015352994
0.015371679
0.015517694
0.015555857
0.015578351
0.015578351

0.015578351
0.015634243
0.015666971
0.015725896
0.015725896
0.015728568
0.015743063
0.015764769
0.01577083
0.015773412
0.015773412
0.015883736
0.015883736
0.015937903
0.015937903
0.015937903
0.016067835
0.016067835
0.016133823
0.01624951
0.016265025
0.016269914
0.016281961
0.016294215
0.016294215
0.016370245
0.016401729
0.016401729
0.016428916
0.016486677
0.016486677
0.016511041
0.016553189
0.016553189
0.016553189
0.016553189
0.016557317
0.016603872
0.016671587
0.016680777
0.016680777
0.016680777
0.016680777
0.016680777
0.016680777
0.016680777
0.016680777
0.016680777
0.016680777
0.016707187
0.016852423
0.016875829
0.016875829
0.016913295
0.016913295
0.016943267
0.017002983

0.017053557
0.017070858
0.017070858
0.0170929
0.017105933
0.017183775
0.017195239
0.01720375
0.017215282
0.017321212
0.017324141
0.017324141
0.01752008
0.017540084
0.017669429
0.017669429
0.017669429
0.017719916
0.017722578
0.017727936
0.017727936
0.017727936
0.017727936
0.017727936
0.017727936
0.017727936
0.017771232
0.017771232
0.017913968
0.017913968
0.017914254
0.017961858
0.017961858
0.017979029
0.018082265
0.018307449
0.018346077
0.018375786
0.018467031
0.018467031
0.018467031
0.018467031
0.018467031
0.018467031
0.018484816
0.018485371
0.01857229
0.01857229
0.01857229
0.01857229
0.01857229
0.01857229
0.01857229
0.018587334
0.018628446
0.018689018
0.01870803

0.018931348
0.018992484
0.019135216
0.019139373
0.019139373
0.019205242
0.019205242
0.019205242
0.019205242
0.019243098
0.019243098
0.019255463
0.019281746
0.019322429
0.019322429
0.019329238
0.019359701
0.019399585
0.019399585
0.019490892
0.019523573
0.019699835
0.019728252
0.019792412
0.019816485
0.019870455
0.019910953
0.019910953
0.019935408
0.020122246
0.020122246
0.020122246
0.020122246
0.020122246
0.020122246
0.020122246
0.020122246
0.020122246
0.020134958
0.020164665
0.020199594
0.020287022
0.020287022
0.020287022
0.020540034
0.020645175
0.020677593
0.020677593
0.020735395
0.020759146
0.020759146
0.020759146
0.02076011
0.020796113
0.020825724
0.021033577
0.02105039

0.021086069
0.021114863
0.021124397
0.02117156
0.021322492
0.021322492
0.02133647
0.02135025
0.02135025
0.02135025
0.021434205
0.021434205
0.02143868
0.021471342
0.021538019
0.021726509
0.021726509
0.021726509
0.021816843
0.02195057
0.02200161
0.022111406
0.022217713
0.022298123
0.022357938
0.022453762
0.022453762
0.022498128
0.02256864
0.02256864
0.022600291
0.022600291
0.022678901
0.022721956
0.022761125
0.022761125
0.022931322
0.02295184
0.02295184
0.023223031
0.023223031
0.023244932
0.023272465
0.023280882
0.023318566
0.023318566
0.023484532
0.023508443
0.023508443
0.023508443
0.023543101
0.023575591
0.023575591
0.023575591
0.023584765
0.023651906
0.02368523

0.02368523
0.023699572
0.023699572
0.02370064
0.023717186
0.023717186
0.023717186
0.023754525
0.023754525
0.023790076
0.02380576
0.023887912
0.023887912
0.02391303
0.023913679
0.023913679
0.023913679
0.023960074
0.023990558
0.02405032
0.02405032
0.024065843
0.024065843
0.024078694
0.024078694
0.024116369
0.024126272
0.02413598
0.024222477
0.024222477
0.024242121
0.024242121
0.024242121
0.024309104
0.024309104
0.024332528
0.024346343
0.024386912
0.024427118
0.024427118
0.0246149
0.0246149
0.0246149
0.0246149
0.0246149
0.024667031
0.024667031
0.024705938
0.024761538
0.024784287
0.024784287
0.024869524
0.024897283
0.024969859
0.02504925
0.02504925
0.02504925

0.02504925
0.025092633
0.025094116
0.025099495
0.025099495
0.025155903
0.025155903
0.025155903
0.025155903
0.025155903
0.025219458
0.025222276
0.025222276
0.025222276
0.025326397
0.025342118
0.025418746
0.025482285
0.025482285
0.025584092
0.025623298
0.025623298
0.025698051
0.025810595
0.025872985
0.025883799
0.025885233
0.025941936
0.026009636
0.026045789
0.0260961
0.026136548
0.026136548
0.026136548
0.026136548
0.026164211
0.026188304
0.026188304
0.026207661
0.026229847
0.026232338
0.026232338
0.02630578
0.026320977
0.026458517
0.026458517
0.026458517
0.026533898
0.026572507
0.026582566
0.026803542
0.026838243
0.026847101
0.026849587
0.026935038
0.026935038
0.026948755

0.026999342
0.027057053
0.027097397
0.02710226
0.027168408
0.0271693
0.0271693
0.0271693
0.027201208
0.027215677
0.027215677
0.027215677
0.027249998
0.027268717
0.027320366
0.027320366
0.027320366
0.027320366
0.027457918
0.027457918
0.027464414
0.027516464
0.027516464
0.027541164
0.027656586
0.027656586
0.027656586
0.02767147
0.027717457
0.027849427
0.027881788
0.027881788
0.027881788
0.027881865
0.027881865
0.028117315
0.028175408
0.028203658
0.028237862
0.028237862
0.028237862
0.028237862
0.028273195
0.028369027
0.028392551
0.028392551
0.028392551
0.028392551
0.028392551
0.028392551
0.028497755
0.028573566
0.028598777
0.02860952
0.02860952
0.028613623
0.028613623

0.028717031
0.028755882
0.028755882
0.028805579
0.02881737
0.02881737
0.02903409
0.029056239
0.029056239
0.029216421
0.029216421
0.029230498
0.029230498
0.029230498
0.029242805
0.029260949
0.029283378
0.0294213
0.0294213
0.0294213
0.0294213
0.02952307
0.029524979
0.029534959
0.029552823
0.029599958
0.029636517
0.029673776
0.029677256
0.029682887
0.029705876
0.02974552
0.029817075
0.029819552
0.029819552
0.029854266
0.029899898
0.029941572
0.029941572
0.030060395
0.030127218
0.030183253
0.030183253
0.030183253
0.030183253
0.030183253
0.030283036
0.030300869
0.030324784
0.030324784
0.030408714
0.030645648
0.030719836
0.030740305
0.03080235
0.030823197
0.030920796

0.030942889
0.030942889
0.030942889
0.030942889
0.030942889
0.031065678
0.031065678
0.031065696
0.031065696
0.031079753
0.031079753
0.031079753
0.031088995
0.031133696
0.031133696
0.031146691
0.031163942
0.031163942
0.031302825
0.031493036
0.031540357
0.031554671
0.031567667
0.031648035
0.031648035
0.031687354
0.031753855
0.031779899
0.032030185
0.03208073
0.032110104
0.032144164
0.032185147
0.032418601
0.032418601
0.032477961
0.03248882
0.032506048
0.032506048
0.032506048
0.032536701
0.032536701
0.032639607
0.032663819
0.032672087
0.03268823
0.032849336
0.032849336
0.032922517
0.032981899
0.032981899
0.032998671
0.03302165
0.03302165
0.0330896
0.033127638
0.033127638

0.033155638
0.033155638
0.033184539
0.033213742
0.033213742
0.033266144
0.033266144
0.033278016
0.033340315
0.033340315
0.033340315
0.033408938
0.033408938
0.033408938
0.033408938
0.033408938
0.033514716
0.03357394
0.033581217
0.033581217
0.033882086
0.033919181
0.033919181
0.033942148
0.033970918
0.033970918
0.034031741
0.034072224
0.034072224
0.034072224
0.034086345
0.0340981
0.034115468
0.034152077
0.034153939
0.034179817
0.03427413
0.034289802
0.034533143
0.034588756
0.034617259
0.034617259
0.034618083
0.034618083
0.03470105
0.034828401
0.034940174
0.034965354
0.034996114
0.03507884
0.035174961
0.03524251
0.03524251
0.03524251
0.035271309
0.035271309
0.035456

0.03559864
0.035708804
0.03571314
0.035756255
0.035800071
0.035803481
0.035806719
0.035806719
0.035806719
0.035860784
0.035886914
0.035994324
0.036089924
0.036098824
0.036098824
0.036118784
0.036262453
0.036262453
0.036349322
0.036359738
0.036359738
0.036365449
0.036365449
0.036411313
0.036411313
0.03642885
0.03642885
0.036510258
0.036510258
0.036510258
0.036599107
0.036603142
0.036603142
0.036603142
0.036642131
0.036642131
0.036662385
0.036698465
0.036779131
0.036845456
0.036855485
0.036938515
0.036994015
0.037043637
0.037056193
0.037056193
0.037239615
0.037293261
0.037402626
0.037402626
0.037402626
0.037413797
0.037424112
0.037495746
0.037497939
0.037590004
0.037640406

0.03764695
0.037651915
0.037916602
0.037938482
0.038145196
0.038145196
0.038170097
0.038170097
0.038196883
0.038249383
0.038306735
0.038326834
0.038369928
0.038369928
0.038404238
0.038404238
0.038404238
0.038424373
0.038424373
0.038462918
0.038462918
0.038496861
0.038541543
0.038550046
0.038635906
0.038635906
0.038635906
0.038635906
0.038776903
0.038802414
0.038902997
0.039039201
0.039039201
0.03904139
0.039401953
0.039401953
0.039401953
0.039515553
0.039515553
0.039568041
0.039568041
0.039678943
0.039679084
0.039874942
0.039874942
0.039874942
0.039874942
0.039874942
0.039874942
0.039874942
0.039874942
0.039875056
0.039961114
0.040006869
0.040053075
0.040154863
0.040239669

0.040239669
0.040376478
0.040376625
0.040447794
0.040599654
0.040599654
0.040599654
0.040690628
0.040735153
0.040735153
0.040735153
0.040735153
0.040735153
0.040735153
0.040735153
0.040735153
0.040735153
0.040735153
0.040735153
0.040735153
0.040798705
0.040911664
0.040918092
0.040918092
0.040996167
0.041313746
0.041313746
0.041430626
0.041492776
0.041492776
0.041498395
0.04159093
0.04159093
0.041745152
0.041829055
0.041966866
0.04215544
0.042209672
0.042333382
0.042335681
0.042357626
0.042357626
0.042357626
0.042357626
0.042357626
0.042395235
0.042434014
0.042449713
0.042449713
0.042449713
0.042504292
0.042527774
0.0426434
0.042657081
0.042657081
0.042857027
0.042857027

0.042983845
0.043075421
0.043075421
0.043075513
0.043141612
0.043141612
0.043182725
0.04328874
0.043305103
0.043331501
0.043346821
0.043530139
0.043576855
0.043576855
0.043613259
0.043635448
0.043674496
0.043674647
0.043674647
0.043694319
0.043694319
0.043694319
0.043920296
0.043920296
0.044029731
0.044067182
0.044067182
0.044105707
0.044211548
0.044211548
0.044305458
0.044356666
0.044412273
0.044582647
0.044626876
0.044657118
0.044691727
0.044691727
0.044696884
0.044795398
0.044808206
0.044808206
0.044808206
0.044808206
0.044808206
0.044808206
0.044808206
0.04493605
0.045014502
0.045014502
0.045014502
0.045064299
0.045064299
0.045187806
0.045187806
0.045227165
0.045227165

0.045227165
0.045227165
0.045238152
0.045238152
0.045238152
0.045243777
0.045385183
0.045447787
0.045447787
0.045447787
0.0455047
0.045534061
0.045534061
0.045534061
0.045534061
0.045534061
0.045543849
0.045543849
0.045625515
0.045625515
0.045643688
0.045666538
0.045773843
0.045773843
0.045881124
0.045887828
0.045928732
0.046028291
0.046028556
0.046190683
0.046190683
0.046194612
0.046194612
0.04629461
0.046340352
0.04670042
0.046747344
0.046769702
0.04679975
0.046803287
0.046803287
0.046815407
0.04685915
0.046938235
0.047023089
0.047040744
0.047161481
0.047161481
0.047195655
0.047263733
0.04729326
0.04732674
0.047379134
0.047379134
0.047452375
0.047452375
0.047452375

0.047452375
0.047452375
0.047517198
0.047568783
0.047861476
0.047861476
0.047917889
0.047917889
0.047985053
0.04816614
0.048241606
0.048241606
0.048241606
0.048241606
0.048241606
0.048241606
0.048360198
0.04839159
0.048416554
0.048582438
0.048589614
0.048630443
0.048662026
0.048720682
0.048760032
0.048796823
0.04896216
0.04899067
0.04899067
0.049043642
0.04910533
0.04910533
0.04910533
0.049152107
0.04927422
0.049346383
0.049347883
0.049378205
0.049409511
0.049441223
0.049689986
0.049742753
0.049785105
0.049785105
0.049807826
0.049946884
0.049987076
