# Supplementary material for: Differential expression of genes in olive leaves and buds of ON- versus OFF-crop trees
Source: Sci Rep. 2020 Sep 25;10:15762. doi: 10.1038/s41598-020-72895-7 (PMC7519672; doi:10.1038/s41598-020-72895-7)
Supplement: Supplementary file 5 — Supplementary Table 5. [file 41598_2020_72895_MOESM5_ESM.pdf]

Alternate bearing in olive: Differential expression of genes in leaves and buds

Ebrahim Dastkar[1], Ali Soleimani[1*], Hossein Jafary[2], Juan de Dios Alche[3], Abbas Bahari[4], Mehrshac

Supplementary table S5. Results of gene ontology (GO) analysis of olive's bud samp

	Tags	SeqName	Description	Length	#Hits
TRUE	[BLASTED, I	TRINITY_DN194941_c0_g1_i1	22.0 kDa class IV heat sh	979	20
TRUE	[BLASTED, I	TRINITY_DN88143_c3_g1_i17	ferredoxin-dependent gl	2933	20
TRUE	[BLASTED, I	TRINITY_DN88164_c1_g1_i3	putative LOV domain-cor	4055	20
TRUE	[BLASTED, I	TRINITY_DN88128_c0_g1_i2	ABC transporter G family	3129	20
TRUE	[BLASTED, I	TRINITY_DN88128_c0_g1_i3	ABC transporter G family	2721	20
TRUE	[BLASTED, I	TRINITY_DN88175_c6_g1_i4	maltose excess protein 1	1897	20
TRUE	[NO-BLAST	TRINITY_DN88146_c0_g3_i1	---NA---	324	
TRUE	[BLASTED, I	TRINITY_DN88127_c1_g1_i3	NADP-dependent malic e	1544	20
TRUE	[BLASTED, I	TRINITY_DN88108_c1_g1_i6	carbamoyl-phosphate sy	4732	20
TRUE	[BLASTED, I	TRINITY_DN88123_c0_g1_i23	histidine kinase 2	4646	20
TRUE	[BLASTED, I	TRINITY_DN88193_c1_g6_i5	hypothetical protein CDL	1753	1
TRUE	[BLASTED, I	TRINITY_DN88193_c1_g1_i16	probable galacturonosylt	2214	20
TRUE	[BLASTED, I	TRINITY_DN88159_c0_g2_i10	long chain acyl-CoA synth	5116	20
TRUE	[BLASTED, I	TRINITY_DN88159_c0_g2_i19	long chain acyl-CoA synth	5134	20
TRUE	[BLASTED, I	TRINITY_DN88119_c1_g2_i16	protein transport protein	1358	20
TRUE	[BLASTED, I	TRINITY_DN88181_c1_g2_i11	flowering locus K homolo	2496	20
TRUE	[BLASTED, I	TRINITY_DN88181_c1_g2_i12	flowering locus K homolo	1271	20
TRUE	[BLASTED]	TRINITY_DN88106_c1_g4_i11	mediator of RNA polyme	484	20
TRUE	[BLASTED, I	TRINITY_DN77944_c2_g3_i2	post-illumination chlorop	838	20
TRUE	[BLASTED]	TRINITY_DN77944_c2_g3_i7	post-illumination chlorop	1330	20
TRUE	[BLASTED, I	TRINITY_DN77988_c0_g1_i1	CONSTANS-like 1 protein	1577	20
TRUE	[BLASTED, I	TRINITY_DN77988_c0_g4_i1	zinc finger protein CONS	666	20
TRUE	[BLASTED, I	TRINITY_DN77988_c0_g5_i1	zinc finger protein CONS	1155	20
TRUE	[BLASTED, I	TRINITY_DN77966_c2_g6_i1	cinnamoyl-CoA reductase	514	20
TRUE	[BLASTED, I	TRINITY_DN77966_c3_g1_i9	histone acetyltransferase	4735	20
TRUE	[BLASTED, I	TRINITY_DN87407_c2_g2_i1	glutaredoxin domain-cor	1557	20
TRUE	[BLASTED, I	TRINITY_DN87442_c1_g1_i10	29 kDa ribonucleoprotein	498	20
TRUE	[BLASTED, I	TRINITY_DN87428_c7_g4_i3	glyceraldehyde-3-phosph	826	20
TRUE	[BLASTED]	TRINITY_DN87474_c1_g2_i4	Ribonuclease H protein	1862	20
TRUE	[BLASTED, I	TRINITY_DN87492_c0_g2_i3	probable protein phosph	704	20
TRUE	[BLASTED, I	TRINITY_DN87493_c1_g4_i2	luminal-binding protein 5	1610	20
TRUE	[BLASTED, I	TRINITY_DN87493_c1_g4_i6	luminal-binding protein	1101	20
TRUE	[BLASTED, I	TRINITY_DN87493_c1_g4_i8	luminal-binding protein	1840	20
TRUE	[BLASTED, I	TRINITY_DN87445_c0_g2_i7	glyoxysomal processing p	1760	20
TRUE	[BLASTED, I	TRINITY_DN87464_c1_g1_i15	probable splicing factor 3	3754	20
TRUE	[BLASTED, I	TRINITY_DN87464_c1_g1_i16	probable splicing factor 3	3696	20
TRUE	[BLASTED, I	TRINITY_DN87464_c1_g1_i24	probable splicing factor 3	3742	20
TRUE	[BLASTED, I	TRINITY_DN87419_c0_g2_i3	premnaspirodiene oxyge	1240	20
TRUE	[BLASTED, I	TRINITY_DN87465_c0_g5_i4	histidine kinase 3-like	1142	20
TRUE	[BLASTED]	TRINITY_DN87455_c1_g2_i3	transcription elongation	1159	20
TRUE	[BLASTED]	TRINITY_DN87406_c1_g2_i2	F-box domain, cyclin-like	521	20
TRUE	[BLASTED, I	TRINITY_DN88274_c1_g3_i1	putative disease resistan	1408	20
TRUE	[BLASTED, I	TRINITY_DN88274_c1_g3_i3	putative disease resistan	1380	20
TRUE	[BLASTED]	TRINITY_DN88294_c3_g3_i3	protein FAR1-RELATED SI	3141	20
TRUE	[BLASTED, I	TRINITY_DN88240_c0_g1_i15	probable ubiquitin-conju	4621	20

TRUE	[BLASTED, I	TRINITY_DN88214_c1_g3_i3	WD repeat-containing pr	3013	20
TRUE	[BLASTED, I	TRINITY_DN88286_c0_g1_i15	mitogen-activated protei	3903	20
TRUE	[BLASTED, I	TRINITY_DN88249_c1_g2_i1	berberine bridge enzyme	1027	20
TRUE	[BLASTED, I	TRINITY_DN88288_c4_g1_i26	probable tRNA N6-adenc	1452	20
TRUE	[BLASTED, I	TRINITY_DN88361_c0_g2_i10	transcription factor MYB	3573	20
TRUE	[BLASTED, I	TRINITY_DN88360_c3_g1_i6	probable amino acid per	1697	20
TRUE	[BLASTED, I	TRINITY_DN88310_c0_g1_i15	ABC transporter C family	1485	20
TRUE	[BLASTED]	TRINITY_DN88395_c0_g1_i7	BUD13 homolog isoform	754	20
TRUE	[BLASTED, I	TRINITY_DN88368_c1_g1_i14	protein SCAR2	1427	20
TRUE	[BLASTED, I	TRINITY_DN88365_c5_g2_i3	caffeic acid 3-O-methyltr	1345	20
TRUE	[BLASTED, I	TRINITY_DN88365_c5_g2_i5	caffeic acid 3-O-methyltr	1078	20
TRUE	[BLASTED, I	TRINITY_DN88391_c0_g2_i21	protein GLE1-like isoforn	1613	20
TRUE	[BLASTED, I	TRINITY_DN88375_c0_g6_i1	dynein light chain 2, cyto	1362	20
TRUE	[BLASTED, I	TRINITY_DN88356_c1_g1_i18	N-terminal acetyltransfe	2155	20
TRUE	[BLASTED]	TRINITY_DN88319_c4_g1_i4	protein RRNAD1 isoform	1822	20
TRUE	[BLASTED, I	TRINITY_DN88319_c4_g2_i1	photosystem II 10 kDa pc	288	20
TRUE	[BLASTED, I	TRINITY_DN88319_c4_g1_i16	photosystem II 10 kDa pc	740	20
TRUE	[BLASTED, I	TRINITY_DN88307_c1_g1_i11	tripeptidyl-peptidase 2	3636	20
TRUE	[BLASTED, I	TRINITY_DN88381_c2_g1_i11	fatty acyl-CoA reductase	1052	20
TRUE	[BLASTED, I	TRINITY_DN88309_c0_g1_i3	polyprotein	2449	20
TRUE	[BLASTED, I	TRINITY_DN88394_c0_g1_i2	RAVE (regulator of V-ATF	2534	20
TRUE	[BLASTED, I	TRINITY_DN88394_c0_g1_i5	RAVE (regulator of V-ATF	2631	20
TRUE	[BLASTED, I	TRINITY_DN88359_c0_g1_i14	putative disease resistan	2870	20
TRUE	[BLASTED]	TRINITY_DN88380_c1_g1_i5	HEAT repeat-containing	4586	20
TRUE	[BLASTED, I	TRINITY_DN80108_c0_g3_i1	Aldo/keto reductase	965	20
TRUE	[BLASTED, I	TRINITY_DN80190_c2_g6_i1	CUB and sushi domain-cc	803	20
TRUE	[BLASTED, I	TRINITY_DN80125_c0_g2_i3	cysteine-rich receptor-lik	2457	20
TRUE	[BLASTED, I	TRINITY_DN80148_c0_g4_i5	histidine--tRNA ligase, ch	1055	20
TRUE	[BLASTED, I	TRINITY_DN80150_c2_g1_i4	R3H domain-containing	4190	20
TRUE	[BLASTED, I	TRINITY_DN80150_c2_g1_i12	R3H domain-containing	3616	20
TRUE	[NO-BLAST	TRINITY_DN80180_c0_g3_i2	---NA---	440	
TRUE	[BLASTED, I	TRINITY_DN80198_c0_g3_i3	uncharacterized protein	1555	20
TRUE	[BLASTED, I	TRINITY_DN80170_c0_g1_i11	exocyst complex compor	2152	20
TRUE	[BLASTED, I	TRINITY_DN80186_c0_g2_i1	fructose-bisphosphate al	459	20
TRUE	[BLASTED, I	TRINITY_DN75342_c0_g1_i2	pentatricopeptide repea	2443	20
TRUE	[BLASTED, I	TRINITY_DN88435_c1_g2_i12	SUPPRESSOR OF ABI3-5 i	2990	20
TRUE	[BLASTED, I	TRINITY_DN88435_c1_g2_i31	SUPPRESSOR OF ABI3-5 i	3306	20
TRUE	[BLASTED, I	TRINITY_DN88481_c1_g1_i10	FAD-dependent urate hy	2503	20
TRUE	[BLASTED, I	TRINITY_DN88420_c1_g1_i3	probable ascorbate-spec	4144	20
TRUE	[NO-BLAST	TRINITY_DN88427_c1_g10_i1	---NA---	1517	
TRUE	[BLASTED, I	TRINITY_DN88461_c5_g2_i3	putative late blight resist	2225	20
TRUE	[BLASTED, I	TRINITY_DN88417_c2_g2_i1	beta-glucosidase-like	447	20
TRUE	[BLASTED]	TRINITY_DN88417_c2_g1_i11	beta-glucosidase-like	731	20
TRUE	[BLASTED, I	TRINITY_DN88483_c0_g2_i2	peptidyl-prolyl cis-trans i	835	20
TRUE	[BLASTED, I	TRINITY_DN88476_c2_g1_i1	Retrovirus-related Pol pc	4122	20
TRUE	[BLASTED, I	TRINITY_DN88430_c1_g1_i2	beta-glucosidase 24-like	747	20
TRUE	[BLASTED, I	TRINITY_DN88409_c1_g1_i4	DEAD-box ATP-depender	1280	20
TRUE	[BLASTED, I	TRINITY_DN88445_c0_g1_i6	folate-biopterin transpor	1961	20
TRUE	[BLASTED, I	TRINITY_DN88493_c3_g1_i7	Retrovirus-related Pol pc	5360	20
TRUE	[BLASTED, I	TRINITY_DN88493_c3_g1_i9	Retrovirus-related Pol pc	5245	20

TRUE	[BLASTED, I	TRINITY_DN88493_c3_g4_i2	aquaporin TIP2-1-like	511	20
TRUE	[BLASTED, I	TRINITY_DN88400_c1_g1_i12	pantothenate kinase 2	3787	20
TRUE	[NO-BLAST	TRINITY_DN88408_c1_g1_i7	---NA---	688	
TRUE	[BLASTED, I	TRINITY_DN88478_c0_g2_i37	vacuolar protein sorting-	4428	20
TRUE	[BLASTED, I	TRINITY_DN88490_c0_g2_i10	glutamate receptor 2.7-li	2067	20
TRUE	[BLASTED, I	TRINITY_DN86140_c1_g3_i6	PWWP domain containin	1877	20
TRUE	[BLASTED, I	TRINITY_DN86143_c0_g1_i4	probable mediator of RN	1660	20
TRUE	[BLASTED, I	TRINITY_DN86115_c1_g1_i1	ankyrin repeat-containin	616	20
TRUE	[BLASTED, I	TRINITY_DN86172_c1_g1_i12	Putative finger family pro	3848	20
TRUE	[BLASTED]	TRINITY_DN86198_c0_g1_i2	Serine-rich adhesin for pl	4258	20
TRUE	[BLASTED]	TRINITY_DN86145_c2_g1_i11	CCR4-NOT transcription c	2240	20
TRUE	[BLASTED, I	TRINITY_DN86147_c0_g1_i12	ubiquitin receptor RAD23	2233	20
TRUE	[BLASTED, I	TRINITY_DN86147_c0_g1_i19	ubiquitin receptor RAD23	1926	20
TRUE	[NO-BLAST	TRINITY_DN86196_c1_g2_i2	---NA---	668	
TRUE	[NO-BLAST	TRINITY_DN86196_c4_g7_i1	---NA---	712	
TRUE	[BLASTED, I	TRINITY_DN86194_c1_g3_i1	nitrate reductase	1368	20
TRUE	[BLASTED, I	TRINITY_DN86166_c1_g3_i3	probable aldo-keto reduc	2430	20
TRUE	[BLASTED, I	TRINITY_DN86166_c1_g3_i13	probable aldo-keto reduc	712	20
TRUE	[BLASTED, I	TRINITY_DN86127_c1_g2_i6	organic cation/carnitine t	2013	20
TRUE	[BLASTED, I	TRINITY_DN86126_c1_g3_i1	heat shock cognate prote	1575	20
TRUE	[BLASTED, I	TRINITY_DN86126_c1_g5_i1	heat shock cognate prote	1148	20
TRUE	[BLASTED, I	TRINITY_DN86126_c1_g3_i4	heat shock cognate prote	1569	20
TRUE	[BLASTED]	TRINITY_DN86157_c0_g2_i2	hypothetical protein CDL	3290	20
TRUE	[BLASTED]	TRINITY_DN86156_c0_g2_i10	Actin cytoskeleton-regula	5019	20
TRUE	[BLASTED, I	TRINITY_DN86112_c1_g9_i2	histone H2B-like	599	20
TRUE	[BLASTED, I	TRINITY_DN86152_c1_g1_i7	glycerol-3-phosphate deh	1132	20
TRUE	[BLASTED, I	TRINITY_DN86179_c1_g2_i5	26S protease regulatory s	2007	20
TRUE	[BLASTED]	TRINITY_DN86183_c3_g1_i14	Protein neuralized like	2682	20
TRUE	[BLASTED]	TRINITY_DN86183_c3_g1_i31	Protein neuralized like	2641	20
TRUE	[BLASTED]	TRINITY_DN86183_c3_g1_i35	Protein neuralized like	2323	20
TRUE	[BLASTED, I	TRINITY_DN86189_c0_g1_i4	bZIP transcription factor	2244	20
TRUE	[BLASTED, I	TRINITY_DN86189_c0_g1_i8	ABSCISIC ACID-INSENSITI	915	20
TRUE	[BLASTED, I	TRINITY_DN86150_c0_g2_i7	histone-lysine N-methylt	1499	20
TRUE	[BLASTED, I	TRINITY_DN86197_c3_g1_i13	linoleate 13S-lipoxygenas	2852	20
TRUE	[BLASTED, I	TRINITY_DN81198_c2_g2_i2	mutant iridoid synthase	1937	20
TRUE	[BLASTED, I	TRINITY_DN81151_c0_g1_i1	scarecrow-like protein 21	1822	20
TRUE	[BLASTED, I	TRINITY_DN81161_c2_g3_i3	sucrose synthase	678	20
TRUE	[BLASTED, I	TRINITY_DN81133_c0_g4_i2	14-3-3-like protein	1531	20
TRUE	[BLASTED, I	TRINITY_DN81152_c2_g2_i1	cyclin-dependent protein	854	20
TRUE	[BLASTED]	TRINITY_DN81113_c1_g2_i4	SAP domain-containing p	1026	20
TRUE	[BLASTED, I	TRINITY_DN81168_c0_g1_i1	protein PIN-LIKES 6	1162	20
TRUE	[BLASTED, I	TRINITY_DN81139_c2_g1_i3	CTL-like protein DDB_G0	1856	20
TRUE	[BLASTED, I	TRINITY_DN81139_c2_g1_i5	CTL-like protein DDB_G0	2181	20
TRUE	[BLASTED, I	TRINITY_DN81107_c0_g5_i3	probable methyltransfera	691	20
TRUE	[BLASTED]	TRINITY_DN81177_c0_g4_i2	golgin family A protein	1089	20
TRUE	[BLASTED, I	TRINITY_DN81196_c0_g1_i1	cyclic dof factor 1-like	1543	20
TRUE	[BLASTED, I	TRINITY_DN81196_c0_g3_i1	cyclic dof factor 1-like	3177	20
TRUE	[BLASTED, I	TRINITY_DN81156_c1_g1_i3	E3 ubiquitin-protein ligas	4756	20
TRUE	[BLASTED, I	TRINITY_DN81165_c2_g1_i6	oligouridylate-binding pr	2672	20
TRUE	[BLASTED, I	TRINITY_DN81450_c0_g4_i5	protein EIN4	2104	20

TRUE	[BLASTED, I	TRINITY_DN81450_c0_g2_i2	protein EIN4	1952	20
TRUE	[BLASTED]	TRINITY_DN81497_c1_g1_i4	hypersensitive-induced r	1341	20
TRUE	[BLASTED, I	TRINITY_DN81485_c0_g3_i1	mannan endo-1,4-beta-n	1624	20
TRUE	[BLASTED, I	TRINITY_DN81469_c0_g1_i6	protein decapping 5	2217	20
TRUE	[BLASTED]	TRINITY_DN81469_c0_g2_i3	protein decapping 5-like	930	20
TRUE	[BLASTED, I	TRINITY_DN81469_c0_g1_i9	protein decapping 5	2202	20
TRUE	[BLASTED, I	TRINITY_DN81471_c0_g3_i6	30s ribosomal protein s1	788	20
TRUE	[BLASTED, I	TRINITY_DN81421_c1_g2_i7	cullin-4-like	3153	20
TRUE	[BLASTED, I	TRINITY_DN81453_c2_g1_i8	Cysteine-rich RLK (RECEP	2121	20
TRUE	[BLASTED]	TRINITY_DN81443_c2_g2_i1	GBF-interacting protein l	1110	20
TRUE	[BLASTED]	TRINITY_DN81443_c2_g1_i11	Ribosomal RNA-processi	1026	20
TRUE	[BLASTED, I	TRINITY_DN81419_c1_g1_i8	sedoheptulose-1,7-bisph	1546	20
TRUE	[BLASTED, I	TRINITY_DN81491_c1_g1_i5	GPI ethanolamine phosp	1148	20
TRUE	[NO-BLAST	TRINITY_DN81412_c0_g1_i2	---NA---	773	
TRUE	[BLASTED, I	TRINITY_DN81476_c0_g1_i6	probable choline kinase 2	4630	20
TRUE	[BLASTED, I	TRINITY_DN81414_c0_g1_i11	uncharacterized protein	541	20
TRUE	[BLASTED, I	TRINITY_DN81425_c0_g3_i2	GPI-anchored wall transf	2021	20
TRUE	[BLASTED, I	TRINITY_DN86377_c2_g1_i11	histone H1-like	4033	20
TRUE	[BLASTED, I	TRINITY_DN86367_c1_g1_i6	protein MEI2-like 5 isofo	4107	20
TRUE	[BLASTED, I	TRINITY_DN86314_c3_g1_i8	WAT1-related protein At	1134	20
TRUE	[BLASTED, I	TRINITY_DN86356_c0_g1_i9	pre-mRNA-splicing factor	3516	20
TRUE	[BLASTED, I	TRINITY_DN86391_c2_g2_i7	transmembrane protein	2121	20
TRUE	[BLASTED, I	TRINITY_DN86351_c0_g1_i5	uncharacterized protein	1596	20
TRUE	[BLASTED, I	TRINITY_DN86333_c3_g1_i2	heat shock cognate 70 kD	627	20
TRUE	[BLASTED, I	TRINITY_DN86333_c3_g5_i4	heat shock cognate 70 kD	1006	20
TRUE	[BLASTED, I	TRINITY_DN86363_c1_g2_i1	chaperone protein ClpB1	2602	20
TRUE	[BLASTED, I	TRINITY_DN86363_c1_g3_i1	chaperone protein ClpB1	3283	20
TRUE	[BLASTED, I	TRINITY_DN86306_c2_g2_i1	uncharacterized protein	1013	20
TRUE	[BLASTED, I	TRINITY_DN86348_c1_g1_i3	cyclin-dependent kinase	5416	20
TRUE	[BLASTED]	TRINITY_DN79409_c2_g2_i5	transcriptional corepress	1718	20
TRUE	[BLASTED]	TRINITY_DN79426_c2_g1_i32	Sanguinarine reductase	1460	20
TRUE	[BLASTED, I	TRINITY_DN79455_c1_g2_i9	sulfate transporter 3.1-lil	2292	20
TRUE	[BLASTED, I	TRINITY_DN79499_c2_g1_i2	uncharacterized protein	2111	20
TRUE	[BLASTED, I	TRINITY_DN79499_c2_g1_i16	uncharacterized protein	2203	20
TRUE	[BLASTED]	TRINITY_DN79432_c0_g1_i1	COBW domain-containin	1919	20
TRUE	[BLASTED]	TRINITY_DN79432_c0_g1_i6	COBW domain-containin	1739	20
TRUE	[BLASTED, I	TRINITY_DN79494_c1_g1_i1	phospholipase A1-IIdelta	618	20
TRUE	[BLASTED, I	TRINITY_DN79494_c1_g1_i2	phospholipase A1-IIdelta	1449	20
TRUE	[BLASTED, I	TRINITY_DN79487_c1_g1_i3	cystathionine gamma-syı	1485	20
TRUE	[BLASTED]	TRINITY_DN79470_c1_g2_i15	uncharacterized protein	1629	20
TRUE	[BLASTED, I	TRINITY_DN79434_c1_g2_i3	S-adenosylmethionine de	2830	20
TRUE	[BLASTED]	TRINITY_DN79407_c1_g2_i8	protein UPSTREAM OF FL	3046	20
TRUE	[BLASTED, I	TRINITY_DN79493_c0_g1_i4	universal stress protein A	774	20
TRUE	[BLASTED, I	TRINITY_DN79400_c3_g1_i1	histone 3	367	20
TRUE	[BLASTED, I	TRINITY_DN79400_c3_g1_i2	histone H3.2	639	20
TRUE	[BLASTED, I	TRINITY_DN79400_c3_g4_i2	histone H3.2	674	20
TRUE	[BLASTED, I	TRINITY_DN79400_c3_g2_i3	predicted protein	906	20
TRUE	[BLASTED, I	TRINITY_DN79400_c3_g11_i1	histone H3.2	958	20
TRUE	[NO-BLAST	TRINITY_DN79400_c3_g9_i6	---NA---	922	
TRUE	[BLASTED, I	TRINITY_DN84787_c0_g1_i3	UDP-N-acetylglucosamin	547	20

TRUE	[BLASTED, I	TRINITY_DN84717_c0_g1_i5	adenylate kinase 5, chlor	4520	20
TRUE	[BLASTED, I	TRINITY_DN84717_c0_g1_i11	adenylate kinase 5, chlor	906	20
TRUE	[BLASTED, I	TRINITY_DN84776_c3_g1_i17	probable zinc metallopro	715	20
TRUE	[BLASTED, I	TRINITY_DN84719_c0_g2_i7	serine/threonine-protein	3151	20
TRUE	[BLASTED, I	TRINITY_DN84741_c0_g3_i3	acid phosphatase 1-like	1030	20
TRUE	[BLASTED, I	TRINITY_DN84746_c0_g2_i6	sialyltransferase-like pro	1403	20
TRUE	[BLASTED, I	TRINITY_DN84745_c0_g2_i14	Pseudouridine-5'-phosph	2055	20
TRUE	[BLASTED]	TRINITY_DN84705_c4_g1_i2	uncharacterized protein	4286	3
TRUE	[BLASTED]	TRINITY_DN84775_c0_g5_i4	ankyrin repeat protein SI	3106	20
TRUE	[BLASTED]	TRINITY_DN84775_c0_g5_i12	ankyrin repeat protein SI	2710	20
TRUE	[NO-BLAST	TRINITY_DN84773_c1_g1_i3	---NA---	2198	
TRUE	[BLASTED, I	TRINITY_DN84788_c0_g1_i23	AP3-complex subunit bet	3740	20
TRUE	[BLASTED, I	TRINITY_DN84780_c0_g1_i2	protein SICKLE-like isofor	981	20
TRUE	[BLASTED, I	TRINITY_DN84780_c0_g2_i4	protein SICKLE-like	1784	20
TRUE	[BLASTED]	TRINITY_DN84771_c2_g6_i1	protein SRC2	1098	20
TRUE	[BLASTED]	TRINITY_DN84782_c0_g1_i5	hybrid signal transductio	1903	20
TRUE	[BLASTED, I	TRINITY_DN84723_c2_g2_i2	calcium-dependent prote	2328	20
TRUE	[BLASTED, I	TRINITY_DN84723_c2_g2_i10	calcium-dependent prote	1960	20
TRUE	[BLASTED, I	TRINITY_DN84718_c2_g9_i1	4-alpha-glucanotransfera	1217	20
TRUE	[BLASTED, I	TRINITY_DN84738_c1_g1_i5	protein SUPPRESSOR OF	3481	20
TRUE	[BLASTED]	TRINITY_DN84790_c0_g1_i1	uncharacterized protein	613	1
TRUE	[BLASTED, I	TRINITY_DN84797_c0_g2_i11	alpha-1,3-mannosyl-glyc	3162	20
TRUE	[BLASTED, I	TRINITY_DN84797_c0_g2_i19	alpha-1,3-mannosyl-glyc	3197	20
TRUE	[BLASTED, I	TRINITY_DN84798_c1_g1_i9	OTU domain-containing p	2627	20
TRUE	[BLASTED, I	TRINITY_DN77091_c0_g1_i2	ATP-dependent Clp prote	1735	20
TRUE	[BLASTED, I	TRINITY_DN77069_c2_g1_i7	protein transport proteir	1666	20
TRUE	[BLASTED, I	TRINITY_DN77074_c1_g1_i4	Alanine--tRNA ligase	1074	20
TRUE	[BLASTED]	TRINITY_DN77078_c0_g1_i5	inactive purple acid phos	2153	20
TRUE	[BLASTED, I	TRINITY_DN82606_c0_g3_i4	ruBisCO large subunit-bir	987	20
TRUE	[BLASTED, I	TRINITY_DN82627_c0_g1_i6	3-oxo-Delta(4,5)-steroid	1018	20
TRUE	[BLASTED]	TRINITY_DN82629_c2_g1_i1	uncharacterized protein	716	1
TRUE	[NO-BLAST	TRINITY_DN82629_c4_g1_i3	---NA---	1657	
TRUE	[BLASTED, I	TRINITY_DN82639_c0_g2_i5	uncharacterized protein	3799	20
TRUE	[BLASTED, I	TRINITY_DN82646_c2_g2_i5	lysM domain receptor-lik	2702	20
TRUE	[BLASTED, I	TRINITY_DN82690_c1_g2_i4	fatty-acid-binding protei	1247	20
TRUE	[BLASTED, I	TRINITY_DN82641_c0_g2_i11	calmodulin-binding prote	1073	20
TRUE	[BLASTED, I	TRINITY_DN82664_c3_g5_i1	aquaporin TIP1-3-like	1242	20
TRUE	[BLASTED, I	TRINITY_DN82660_c2_g3_i6	cathepsin B	1986	20
TRUE	[BLASTED, I	TRINITY_DN82663_c1_g1_i5	trans-cinnamate 4-mono	1723	20
TRUE	[BLASTED, I	TRINITY_DN82613_c0_g1_i3	cleavage and polyadenyl	2202	20
TRUE	[BLASTED]	TRINITY_DN82698_c0_g1_i9	D-lactate dehydrogenase	591	1
TRUE	[BLASTED, I	TRINITY_DN82692_c1_g1_i1	Molecular chaperone (Dr	2303	20
TRUE	[BLASTED, I	TRINITY_DN85788_c1_g2_i4	anthocyanidin 3-O-gluco	2839	20
TRUE	[BLASTED, I	TRINITY_DN85743_c0_g1_i4	probable serine/threonir	1098	20
TRUE	[BLASTED, I	TRINITY_DN85792_c1_g4_i2	crocetin glucosyltransfer	1499	20
TRUE	[BLASTED, I	TRINITY_DN85792_c1_g4_i3	crocetin glucosyltransfer	1777	20
TRUE	[BLASTED, I	TRINITY_DN85717_c0_g2_i6	histone chaperone ASF1E	2013	20
TRUE	[BLASTED, I	TRINITY_DN85712_c1_g2_i3	protein MEI2-like 2	1459	20
TRUE	[BLASTED, I	TRINITY_DN85704_c1_g3_i15	protein CIA1	2114	20
TRUE	[BLASTED, I	TRINITY_DN85716_c1_g3_i5	dnaJ homolog subfamily	1465	20

TRUE	[BLASTED]	TRINITY_DN85741_c1_g2_i2	chaperone protein dnaJ :	1872	20
TRUE	[BLASTED, I	TRINITY_DN85711_c1_g2_i3	histone-lysine N-methylt	615	20
TRUE	[BLASTED, I	TRINITY_DN85744_c2_g1_i7	myosin-2 isoform X1	2725	20
TRUE	[BLASTED, I	TRINITY_DN80090_c0_g1_i8	glycine-rich RNA-binding	1135	20
TRUE	[BLASTED]	TRINITY_DN80007_c0_g5_i3	uncharacterized LOC1078	1805	20
TRUE	[BLASTED]	TRINITY_DN80005_c1_g1_i2	VIN3-like protein 1	2070	20
TRUE	[BLASTED, I	TRINITY_DN80028_c1_g1_i8	serine/threonine-protein	1239	20
TRUE	[BLASTED, I	TRINITY_DN80087_c1_g2_i10	transcription factor MYB	1190	20
TRUE	[NO-BLAST	TRINITY_DN80011_c0_g1_i7	---NA---	482	
TRUE	[BLASTED, I	TRINITY_DN80052_c0_g2_i10	photosynthetic NDH subu	1846	20
TRUE	[BLASTED, I	TRINITY_DN80085_c4_g1_i14	Isopentenyl phosphate k	2300	20
TRUE	[BLASTED, I	TRINITY_DN81799_c4_g3_i9	60S acidic ribosomal prot	500	20
TRUE	[BLASTED, I	TRINITY_DN81700_c1_g5_i2	zinc finger CCHC domain-	1961	20
TRUE	[BLASTED, I	TRINITY_DN81742_c2_g1_i14	acetyl-CoA acetyltransfer	1788	20
TRUE	[BLASTED, I	TRINITY_DN81742_c2_g1_i18	acetyl-CoA acetyltransfer	1758	20
TRUE	[BLASTED, I	TRINITY_DN81786_c1_g1_i2	probable protein phosph	1270	20
TRUE	[BLASTED]	TRINITY_DN81753_c0_g1_i6	uncharacterized protein	1608	20
TRUE	[BLASTED, I	TRINITY_DN81752_c0_g1_i12	cytosolic 5'-nucleotidase	1247	20
TRUE	[BLASTED, I	TRINITY_DN81752_c0_g1_i21	cytosolic 5'-nucleotidase	1234	20
TRUE	[BLASTED, I	TRINITY_DN81706_c3_g1_i6	probable plastidic glucos	2031	20
TRUE	[NO-BLAST	TRINITY_DN81773_c0_g1_i18	---NA---	933	
TRUE	[BLASTED]	TRINITY_DN81755_c0_g3_i20	IQ domain-containing pr	469	1
TRUE	[BLASTED, I	TRINITY_DN81702_c0_g1_i4	2,3-bisphosphoglycerate-	1585	20
TRUE	[BLASTED, I	TRINITY_DN86462_c1_g2_i2	casein kinase 1-like prote	3128	20
TRUE	[BLASTED, I	TRINITY_DN86462_c1_g1_i9	casein kinase 1-like prote	3209	20
TRUE	[BLASTED, I	TRINITY_DN86462_c1_g2_i5	casein kinase 1-like prote	3264	20
TRUE	[BLASTED]	TRINITY_DN86495_c2_g4_i1	protein SRC2-like	1374	1
TRUE	[BLASTED, I	TRINITY_DN86465_c0_g1_i9	Serine/threonine protein	951	20
TRUE	[BLASTED, I	TRINITY_DN86449_c1_g1_i25	vesicle-associated memb	2698	20
TRUE	[BLASTED, I	TRINITY_DN86430_c0_g1_i1	early light-induced prote	555	20
TRUE	[BLASTED, I	TRINITY_DN86430_c0_g4_i1	early light-induced prote	374	20
TRUE	[BLASTED, I	TRINITY_DN86430_c0_g2_i2	early light-induced prote	356	20
TRUE	[BLASTED, I	TRINITY_DN86430_c0_g3_i2	early light-induced prote	408	20
TRUE	[BLASTED, I	TRINITY_DN86430_c0_g3_i5	early light-induced prote	499	20
TRUE	[BLASTED, I	TRINITY_DN86430_c0_g1_i3	early light-induced prote	516	20
TRUE	[BLASTED, I	TRINITY_DN86445_c0_g5_i3	secoisolariciresinol dehy	775	20
TRUE	[BLASTED]	TRINITY_DN86439_c3_g1_i7	sister chromatid cohesio	4382	20
TRUE	[BLASTED, I	TRINITY_DN86414_c0_g5_i2	uncharacterized protein	1583	20
TRUE	[BLASTED, I	TRINITY_DN86414_c0_g5_i4	uncharacterized protein	1754	20
TRUE	[BLASTED, I	TRINITY_DN86447_c1_g2_i2	probable polygalacturona	1505	20
TRUE	[NO-BLAST	TRINITY_DN86422_c0_g4_i3	---NA---	809	
TRUE	[BLASTED, I	TRINITY_DN86419_c0_g1_i3	DEAD-box ATP-depender	1715	20
TRUE	[BLASTED, I	TRINITY_DN86419_c0_g8_i1	proline-rich receptor-like	987	20
TRUE	[BLASTED, I	TRINITY_DN86435_c0_g2_i3	sodium transporter HKT1	2110	20
TRUE	[BLASTED, I	TRINITY_DN86435_c0_g1_i9	oxygen-evolving enhance	1153	20
TRUE	[BLASTED, I	TRINITY_DN86467_c2_g2_i6	protein CHROMATIN REN	2132	20
TRUE	[BLASTED, I	TRINITY_DN86474_c1_g1_i5	transcription initiation fa	3110	20
TRUE	[BLASTED, I	TRINITY_DN86474_c2_g1_i6	beta-amylase 1, chloropl	1430	20
TRUE	[BLASTED, I	TRINITY_DN86416_c0_g1_i14	maltose excess protein 1	3889	20
TRUE	[BLASTED, I	TRINITY_DN86418_c1_g1_i9	dnaJ protein homolog	843	20

TRUE	[BLASTED,	TRINITY_DN86487_c3_g1_i1	MADS-box transcription	3194	20
TRUE	[BLASTED,	TRINITY_DN86402_c2_g1_i19	suppressor of RPS4-RLD	1095	20
TRUE	[BLASTED]	TRINITY_DN86457_c1_g6_i1	large proline-rich protein	788	20
TRUE	[BLASTED]	TRINITY_DN86457_c1_g5_i3	large proline-rich protein	1845	20
TRUE	[BLASTED,	TRINITY_DN86488_c0_g1_i1	beta-glucosidase-like	2361	20
TRUE	[BLASTED,	TRINITY_DN77648_c2_g2_i9	ubiquitin-conjugating en	4116	20
TRUE	[BLASTED,	TRINITY_DN77656_c0_g1_i3	cytokinin riboside 5'-mor	2863	20
TRUE	[BLASTED,	TRINITY_DN77659_c0_g1_i3	5'-3' exoribonuclease	1710	20
TRUE	[BLASTED,	TRINITY_DN77665_c3_g1_i5	photosystem I reaction c	859	20
TRUE	[BLASTED,	TRINITY_DN83176_c2_g1_i2	AAA+-type ATPase	3504	20
TRUE	[BLASTED,	TRINITY_DN83176_c2_g1_i18	protein msp1	3539	20
TRUE	[BLASTED,	TRINITY_DN83147_c0_g1_i1	eukaryotic translation ini	686	20
TRUE	[BLASTED,	TRINITY_DN83177_c0_g1_i6	gibberellin 2-beta-dioxyg	1996	20
TRUE	[BLASTED,	TRINITY_DN83124_c0_g1_i3	methionine--tRNA ligase,	2608	20
TRUE	[BLASTED,	TRINITY_DN83128_c2_g2_i2	endonuclease 4-like	1769	20
TRUE	[BLASTED,	TRINITY_DN83113_c3_g1_i6	thioredoxin reductase N	1184	20
TRUE	[BLASTED,	TRINITY_DN83153_c0_g1_i7	Non-specific serine/thre	3930	20
TRUE	[BLASTED,	TRINITY_DN83180_c0_g2_i14	telomere repeat-binding	3113	20
TRUE	[BLASTED,	TRINITY_DN83180_c0_g2_i21	telomere repeat-binding	2959	20
TRUE	[BLASTED,	TRINITY_DN83121_c0_g1_i4	telomeric repeat-binding	2621	20
TRUE	[BLASTED,	TRINITY_DN83179_c1_g4_i2	methyl-CpG-binding dom	962	20
TRUE	[BLASTED,	TRINITY_DN83105_c0_g3_i3	scarecrow-like transcript	822	20
TRUE	[BLASTED,	TRINITY_DN83178_c1_g1_i7	RNA polymerase sigma fa	3506	20
TRUE	[BLASTED,	TRINITY_DN83178_c1_g1_i14	RNA polymerase sigma fa	3518	20
TRUE	[BLASTED,	TRINITY_DN83115_c1_g1_i9	CDPK-related protein kin	5823	20
TRUE	[BLASTED,	TRINITY_DN83160_c0_g3_i2	transcription factor bHLH	1948	20
TRUE	[BLASTED]	TRINITY_DN83111_c0_g1_i2	F-box protein PP2-B11-lil	233	4
TRUE	[BLASTED]	TRINITY_DN83139_c1_g1_i1	Ten-2 intracellular doma	1395	20
TRUE	[BLASTED,	TRINITY_DN84185_c0_g1_i2	phosphatidate phosphat	2597	20
TRUE	[BLASTED,	TRINITY_DN84191_c2_g1_i18	E3 ubiquitin-protein ligas	1722	20
TRUE	[BLASTED,	TRINITY_DN84132_c2_g3_i1	golgin subfamily A memb	3048	20
TRUE	[BLASTED,	TRINITY_DN84143_c2_g2_i10	probable magnesium tra	688	20
TRUE	[BLASTED,	TRINITY_DN84143_c2_g2_i14	probable magnesium tra	3304	20
TRUE	[BLASTED,	TRINITY_DN84137_c0_g3_i2	serine/threonine-protein	1066	20
TRUE	[BLASTED,	TRINITY_DN84131_c2_g2_i1	Transmembrane protein	1843	20
TRUE	[BLASTED,	TRINITY_DN84151_c0_g1_i1	PHD finger protein EHD3	2442	20
TRUE	[BLASTED,	TRINITY_DN84128_c1_g2_i9	protein DETOXIFICATION	1974	20
TRUE	[BLASTED,	TRINITY_DN84165_c4_g4_i8	ankyrin repeat domain-c	2760	20
TRUE	[BLASTED,	TRINITY_DN84158_c2_g1_i2	calmodulin-binding trans	1273	20
TRUE	[NO-BLAST	TRINITY_DN84112_c1_g1_i2	---NA---	555	
TRUE	[BLASTED,	TRINITY_DN84118_c1_g1_i3	sulfite exporter TauE/Saf	2220	20
TRUE	[BLASTED,	TRINITY_DN84123_c3_g1_i2	1-phosphatidylinositol-3-	1946	20
TRUE	[BLASTED,	TRINITY_DN84122_c2_g1_i3	1,2-dihydroxy-3-keto-5-n	3344	20
TRUE	[BLASTED,	TRINITY_DN84176_c2_g1_i6	pyruvate kinase 1, cytoso	1876	20
TRUE	[BLASTED,	TRINITY_DN78103_c0_g3_i3	DNA ligase 1-like	1526	20
TRUE	[BLASTED,	TRINITY_DN78103_c0_g4_i5	shikimate O-hydroxycinn	1898	20
TRUE	[BLASTED,	TRINITY_DN78140_c4_g1_i1	histone H2AX-like	844	20
TRUE	[BLASTED,	TRINITY_DN78140_c4_g1_i5	histone H2AX-like	928	20
TRUE	[BLASTED,	TRINITY_DN78169_c2_g8_i1	Chlorophyll A-B binding p	559	20
TRUE	[BLASTED,	TRINITY_DN78169_c2_g8_i2	Chlorophyll A-B binding p	611	20

TRUE	[BLASTED, I	TRINITY_DN78125_c0_g4_i5	SNF1-related protein kin	889	20
TRUE	[BLASTED, I	TRINITY_DN78161_c0_g2_i1	protein TPLATE	3892	20
TRUE	[BLASTED, I	TRINITY_DN78161_c0_g2_i7	protein TPLATE	3807	20
TRUE	[BLASTED, I	TRINITY_DN78116_c2_g1_i16	bifunctional 3-dehydroqu	2070	20
TRUE	[BLASTED, I	TRINITY_DN78130_c1_g2_i7	ADP,ATP carrier protein I	1328	20
TRUE	[BLASTED, I	TRINITY_DN78128_c1_g1_i7	phosphoglucan phosphat	1400	20
TRUE	[BLASTED, I	TRINITY_DN75260_c0_g1_i1	uncharacterized protein	1089	13
TRUE	[BLASTED, I	TRINITY_DN80360_c0_g1_i5	50S ribosomal protein L1	907	20
TRUE	[BLASTED, I	TRINITY_DN80352_c0_g1_i6	BEL1-like homeodomain	2854	20
TRUE	[BLASTED, I	TRINITY_DN80385_c0_g1_i3	cinnamoyl-coa reductase	1278	20
TRUE	[BLASTED, I	TRINITY_DN80385_c0_g2_i4	cinnamoyl-CoA reductase	1486	20
TRUE	[BLASTED, I	TRINITY_DN80301_c0_g1_i2	heptahelical transmemb	1465	20
TRUE	[BLASTED]	TRINITY_DN80321_c0_g2_i5	pentatricopeptide repeat	5319	20
TRUE	[BLASTED, I	TRINITY_DN80389_c3_g1_i3	sodium/hydrogen exchar	2536	20
TRUE	[BLASTED, I	TRINITY_DN80388_c3_g5_i4	ethylene-responsive tran	1994	20
TRUE	[BLASTED, I	TRINITY_DN80336_c2_g1_i1	pentatricopeptide repeat	1585	20
TRUE	[BLASTED, I	TRINITY_DN82372_c0_g2_i3	protein NRT1/ PTR FAMI	2354	20
TRUE	[BLASTED, I	TRINITY_DN82380_c0_g2_i4	probable 1-acylglycerol-3	985	20
TRUE	[BLASTED, I	TRINITY_DN82381_c0_g2_i5	protein DETOXIFICATION	1810	20
TRUE	[BLASTED, I	TRINITY_DN82381_c0_g2_i10	protein DETOXIFICATION	1860	20
TRUE	[BLASTED]	TRINITY_DN82316_c1_g1_i3	mediator of RNA polyme	2120	20
TRUE	[BLASTED, I	TRINITY_DN82361_c2_g1_i8	histidinol-phosphate ami	753	20
TRUE	[BLASTED, I	TRINITY_DN82387_c0_g3_i4	cytochrome P450 CYP73	944	20
TRUE	[BLASTED, I	TRINITY_DN82364_c0_g1_i19	probable N-acetyl-gamm	1438	20
TRUE	[BLASTED, I	TRINITY_DN82378_c1_g1_i2	guanine nucleotide-bindi	1963	20
TRUE	[BLASTED, I	TRINITY_DN82314_c0_g3_i3	la-related protein 1C-like	2250	20
TRUE	[BLASTED]	TRINITY_DN82383_c2_g1_i1	dehydrin DHN1-like	1067	1
TRUE	[BLASTED, I	TRINITY_DN82384_c0_g2_i2	probable inactive purple	2613	20
TRUE	[BLASTED]	TRINITY_DN82384_c0_g2_i6	protein At-4/1	1068	20
TRUE	[BLASTED, I	TRINITY_DN82335_c4_g1_i7	cyclin-dependent kinase	1829	20
TRUE	[BLASTED, I	TRINITY_DN82344_c0_g2_i1	mRNA cap guanine-N7 m	905	20
TRUE	[BLASTED, I	TRINITY_DN82395_c2_g9_i2	rop guanine nucleotide e	2146	20
TRUE	[BLASTED]	TRINITY_DN82326_c0_g1_i17	protein mard1	1780	20
TRUE	[BLASTED]	TRINITY_DN76141_c0_g1_i4	pentatricopeptide repeat	3570	20
TRUE	[BLASTED]	TRINITY_DN76141_c0_g1_i5	pentatricopeptide repeat	3552	20
TRUE	[BLASTED, I	TRINITY_DN88504_c0_g1_i36	protein argonaute 4	4184	20
TRUE	[BLASTED, I	TRINITY_DN88584_c2_g2_i6	cellulose synthase A cata	1827	20
TRUE	[BLASTED]	TRINITY_DN88569_c3_g6_i2	protein FAR1-RELATED SI	1363	20
TRUE	[NO-BLAST	TRINITY_DN88566_c0_g2_i1	---NA---	6696	
TRUE	[NO-BLAST	TRINITY_DN88566_c0_g2_i9	---NA---	6934	
TRUE	[BLASTED, I	TRINITY_DN88533_c0_g4_i9	callose synthase 11-like	2888	20
TRUE	[BLASTED, I	TRINITY_DN88590_c3_g1_i3	Dehydrin Xero 1	457	7
TRUE	[BLASTED]	TRINITY_DN88572_c3_g5_i3	zinc finger MYM-type pr	2215	20
TRUE	[BLASTED]	TRINITY_DN88583_c1_g1_i16	putative late blight resist	1837	20
TRUE	[BLASTED, I	TRINITY_DN88543_c4_g3_i1	alcohol dehydrogenase c	392	20
TRUE	[BLASTED]	TRINITY_DN88535_c4_g1_i1	uncharacterized protein	1099	20
TRUE	[BLASTED, I	TRINITY_DN88570_c1_g1_i6	TPR transcription factor	1347	20
TRUE	[BLASTED, I	TRINITY_DN88542_c0_g3_i15	histone-lysine N-methylt	1423	20
TRUE	[BLASTED, I	TRINITY_DN84974_c1_g1_i9	mannosyl-oligosaccharid	1102	20
TRUE	[BLASTED, I	TRINITY_DN84974_c1_g1_i17	mannosyl-oligosaccharid	1378	20

TRUE	[BLASTED]	TRINITY_DN84929_c4_g1_i7	protein LNK3-like isoforn	903	12
TRUE	[BLASTED]	TRINITY_DN84929_c4_g1_i11	protein LNK3-like isoforn	4194	13
TRUE	[BLASTED]	TRINITY_DN84929_c4_g1_i12	protein LNK4-like	1556	20
TRUE	[BLASTED, I	TRINITY_DN84995_c1_g2_i13	5'-nucleotidase SurE-like	2613	20
TRUE	[BLASTED, I	TRINITY_DN84956_c5_g4_i3	line-1 retrotransposable	4160	20
TRUE	[BLASTED, I	TRINITY_DN84943_c2_g1_i14	auxin response factor 9-l	1957	20
TRUE	[BLASTED, I	TRINITY_DN84960_c0_g1_i16	membrane-anchored ubi	727	20
TRUE	[BLASTED, I	TRINITY_DN84932_c0_g2_i1	cyclic dof factor 1-like	1917	20
TRUE	[BLASTED, I	TRINITY_DN84984_c0_g1_i6	alanine--tRNA ligase, chl	1794	20
TRUE	[BLASTED, I	TRINITY_DN84904_c2_g2_i6	sedoheptulose-1,7-bisph	707	20
TRUE	[BLASTED, I	TRINITY_DN84904_c2_g2_i8	sedoheptulose-1,7-bisph	1207	20
TRUE	[BLASTED]	TRINITY_DN84933_c2_g4_i2	metallothionein-like prot	233	1
TRUE	[NO-BLAST	TRINITY_DN84933_c2_g7_i1	---NA---	483	
TRUE	[BLASTED, I	TRINITY_DN84902_c5_g1_i2	geraniol 10-hydroxylase	1943	20
TRUE	[BLASTED, I	TRINITY_DN84902_c5_g1_i3	geraniol 10-hydroxylase	2260	20
TRUE	[BLASTED, I	TRINITY_DN84952_c3_g1_i13	divinyl chlorophyllide a 8	824	20
TRUE	[BLASTED]	TRINITY_DN84964_c1_g4_i2	uncharacterized protein	1914	20
TRUE	[BLASTED, I	TRINITY_DN84958_c3_g3_i7	3-hydroxy-3-methylgluta	708	20
TRUE	[BLASTED, I	TRINITY_DN84939_c2_g3_i16	signal recognition particl	2296	20
TRUE	[BLASTED, I	TRINITY_DN84992_c1_g1_i2	methyl-CpG-binding dom	1522	20
TRUE	[BLASTED, I	TRINITY_DN84992_c1_g5_i20	methyl-CpG-binding dom	1522	20
TRUE	[BLASTED]	TRINITY_DN80451_c0_g2_i3	uncharacterized protein	2537	20
TRUE	[BLASTED]	TRINITY_DN80451_c0_g2_i5	high chlorophyll fluoresc	795	20
TRUE	[BLASTED]	TRINITY_DN80486_c1_g3_i3	probable E3 ubiquitin-pr	952	20
TRUE	[NO-BLAST	TRINITY_DN80453_c0_g1_i8	---NA---	1987	
TRUE	[BLASTED]	TRINITY_DN80448_c0_g1_i2	protein WEAK CHLOROPI	2823	20
TRUE	[BLASTED, I	TRINITY_DN80444_c1_g2_i6	protein PHOSPHATE STAI	2694	20
TRUE	[BLASTED, I	TRINITY_DN80478_c0_g1_i3	CRS2-associated factor 2	1860	20
TRUE	[BLASTED, I	TRINITY_DN80426_c0_g1_i6	neurofilament medium p	1657	20
TRUE	[BLASTED, I	TRINITY_DN80476_c2_g1_i10	conserved oligomeric Go	2229	20
TRUE	[BLASTED, I	TRINITY_DN80454_c1_g1_i9	protein HESO1-like	1902	20
TRUE	[BLASTED, I	TRINITY_DN80456_c0_g4_i11	ABC transporter C family	5261	20
TRUE	[BLASTED, I	TRINITY_DN78762_c2_g1_i2	inositol monophosphatas	1007	20
TRUE	[BLASTED, I	TRINITY_DN78741_c3_g2_i5	aspartic proteinase Asp1	2367	20
TRUE	[BLASTED, I	TRINITY_DN78741_c3_g2_i6	aspartic proteinase Asp1	2278	20
TRUE	[BLASTED]	TRINITY_DN78706_c0_g1_i1	uncharacterized protein	1341	20
TRUE	[BLASTED, I	TRINITY_DN78706_c0_g4_i3	protein OVEREXPRESSOR	1556	20
TRUE	[BLASTED, I	TRINITY_DN78767_c0_g1_i10	probable galacturonosylt	2013	20
TRUE	[BLASTED, I	TRINITY_DN78728_c3_g1_i6	HYP1_DAVTARecName: I	598	1
TRUE	[BLASTED, I	TRINITY_DN78798_c1_g1_i5	Telomeric repeat-binding	2342	20
TRUE	[BLASTED, I	TRINITY_DN78710_c0_g4_i2	ATPase, F0 complex, sub	2929	20
TRUE	[BLASTED]	TRINITY_DN78710_c0_g1_i26	F-box protein SKIP17-like	3627	20
TRUE	[BLASTED, I	TRINITY_DN78772_c0_g4_i5	zinc finger protein CONS	1018	20
TRUE	[BLASTED, I	TRINITY_DN78772_c0_g4_i13	Zinc finger protein	1049	20
TRUE	[BLASTED, I	TRINITY_DN78772_c0_g4_i15	Zinc finger protein	1103	20
TRUE	[BLASTED, I	TRINITY_DN78743_c2_g3_i4	squamosa promoter-binc	2011	20
TRUE	[BLASTED, I	TRINITY_DN78743_c2_g4_i9	APO protein 4, mitochon	1804	20
TRUE	[BLASTED, I	TRINITY_DN78705_c1_g4_i6	guanine nucleotide-bindi	2174	20
TRUE	[BLASTED, I	TRINITY_DN78778_c1_g2_i2	CCR4-NOT transcription	666	20
TRUE	[BLASTED, I	TRINITY_DN70904_c0_g1_i1	FAR1-RELATED SEQUENC	2581	20

TRUE	[BLASTED]	TRINITY_DN70521_c0_g1_i1	Pollen Ole e 1 allergen ar	693	20
TRUE	[NO-BLAST	TRINITY_DN70596_c0_g1_i2	---NA---	1045	
TRUE	[BLASTED]	TRINITY_DN80860_c0_g1_i2	protein phosphatase 1 re	1509	20
TRUE	[BLASTED]	TRINITY_DN80860_c0_g1_i4	protein phosphatase 1 re	1490	20
TRUE	[BLASTED]	TRINITY_DN80808_c0_g6_i1	F-box protein SKIP14-like	1713	20
TRUE	[BLASTED, I	TRINITY_DN80847_c1_g1_i3	cytochrome P450 CYP74	1474	20
TRUE	[BLASTED, I	TRINITY_DN80812_c2_g1_i17	RNA polymerase II C-terr	4883	20
TRUE	[BLASTED]	TRINITY_DN80853_c2_g2_i1	J domain-containing prot	2530	20
TRUE	[BLASTED]	TRINITY_DN80870_c3_g1_i11	arginine/serine-rich coile	1577	20
TRUE	[BLASTED, I	TRINITY_DN80850_c0_g1_i5	60S ribosomal export pro	2050	20
TRUE	[NO-BLAST	TRINITY_DN80843_c2_g1_i8	---NA---	1919	
TRUE	[BLASTED, I	TRINITY_DN80832_c0_g3_i4	tobamovirus multiplicati	2286	5
TRUE	[BLASTED, I	TRINITY_DN80800_c2_g3_i1	DELLA protein GAI1-like	2431	20
TRUE	[BLASTED]	TRINITY_DN80886_c1_g1_i4	Rad60/SUMO-like domai	4768	20
TRUE	[BLASTED, I	TRINITY_DN80862_c0_g1_i8	nuclear transcription fac	1625	20
TRUE	[BLASTED, I	TRINITY_DN80884_c1_g3_i5	glutamyl-tRNA(Gln) amid	1404	20
TRUE	[NO-BLAST	TRINITY_DN80881_c1_g1_i3	---NA---	2060	
TRUE	[NO-BLAST	TRINITY_DN80835_c0_g1_i4	---NA---	884	
TRUE	[BLASTED]	TRINITY_DN83865_c0_g2_i11	chaperonin-like RbcX pro	2430	20
TRUE	[BLASTED]	TRINITY_DN83865_c0_g2_i17	chaperonin-like RbcX pro	681	20
TRUE	[BLASTED, I	TRINITY_DN83895_c1_g1_i2	aldehyde dehydrogenase	1941	20
TRUE	[BLASTED, I	TRINITY_DN83817_c3_g2_i7	transcription factor bHLH	2040	20
TRUE	[BLASTED, I	TRINITY_DN83817_c3_g2_i11	transcription factor bHLH	1331	20
TRUE	[BLASTED, I	TRINITY_DN83817_c3_g3_i2	transcription factor bHLH	1305	20
TRUE	[BLASTED, I	TRINITY_DN83852_c1_g3_i3	topless-related protein 4	1603	20
TRUE	[BLASTED, I	TRINITY_DN83892_c1_g2_i1	long chain acyl-CoA synth	2646	20
TRUE	[NO-BLAST	TRINITY_DN83876_c1_g4_i1	---NA---	2205	
TRUE	[BLASTED, I	TRINITY_DN83868_c0_g2_i3	BI1-like protein	1860	20
TRUE	[NO-BLAST	TRINITY_DN83872_c0_g3_i5	---NA---	2888	
TRUE	[BLASTED, I	TRINITY_DN83814_c1_g2_i1	spermidine hydroxycinna	1747	20
TRUE	[BLASTED, I	TRINITY_DN83819_c0_g1_i5	E3 ubiquitin-protein ligas	5642	20
TRUE	[NO-BLAST	TRINITY_DN83837_c1_g1_i13	---NA---	2710	
TRUE	[BLASTED, I	TRINITY_DN84821_c2_g2_i1	AUGMIN subunit 8	908	20
TRUE	[BLASTED]	TRINITY_DN84822_c0_g2_i9	endoribonuclease YBEY,	1293	4
TRUE	[NO-BLAST	TRINITY_DN84832_c0_g8_i2	---NA---	2372	
TRUE	[BLASTED, I	TRINITY_DN84829_c3_g1_i5	acyl-protein thioesterase	1904	20
TRUE	[BLASTED, I	TRINITY_DN84842_c0_g2_i5	alpha-amylase 3, chlorop	3016	20
TRUE	[BLASTED, I	TRINITY_DN84863_c1_g3_i4	ABC transporter G family	2781	20
TRUE	[BLASTED, I	TRINITY_DN84863_c1_g1_i21	DUF21 domain-containin	1315	20
TRUE	[BLASTED, I	TRINITY_DN84862_c2_g3_i5	chloride channel protein	1630	20
TRUE	[BLASTED, I	TRINITY_DN84885_c0_g2_i9	DNA-directed RNA polym	3833	20
TRUE	[NO-BLAST	TRINITY_DN84826_c0_g1_i2	---NA---	1235	
TRUE	[BLASTED, I	TRINITY_DN84848_c1_g2_i8	proline--tRNA ligase, cyto	1943	20
TRUE	[BLASTED, I	TRINITY_DN79102_c1_g3_i3	protein DETOXIFICATION	2181	20
TRUE	[BLASTED, I	TRINITY_DN79102_c2_g1_i6	very-long-chain enoyl-Co	499	20
TRUE	[BLASTED, I	TRINITY_DN79104_c0_g3_i2	E3 ubiquitin-protein ligas	687	20
TRUE	[BLASTED, I	TRINITY_DN79148_c0_g1_i2	CMP-sialic acid transport	1448	20
TRUE	[BLASTED, I	TRINITY_DN79175_c3_g3_i4	DNA photolyase class 1,	971	20
TRUE	[BLASTED, I	TRINITY_DN79175_c3_g1_i3	fructose-bisphosphate al	297	20
TRUE	[BLASTED, I	TRINITY_DN79175_c3_g1_i4	fructose-bisphosphate al	779	20

TRUE	[BLASTED, I	TRINITY_DN79123_c0_g2_i4	UDP-N-acetylglucosamin	2378	20
TRUE	[BLASTED, I	TRINITY_DN79111_c0_g1_i8	CTL-like protein DDB_G0	2239	20
TRUE	[BLASTED, I	TRINITY_DN79168_c1_g1_i1	glutathione S-transferase	1223	20
TRUE	[BLASTED, I	TRINITY_DN79168_c1_g1_i5	glutathione S-transferase	1147	20
TRUE	[BLASTED, I	TRINITY_DN79163_c2_g2_i2	Aspartate--tRNA ligase	1735	20
TRUE	[BLASTED, I	TRINITY_DN79199_c3_g2_i1	photosystem I reaction c	262	3
TRUE	[BLASTED, I	TRINITY_DN79179_c2_g3_i2	E3 ubiquitin-protein ligas	679	20
TRUE	[BLASTED]	TRINITY_DN79131_c3_g1_i13	uncharacterized protein	3315	20
TRUE	[BLASTED]	TRINITY_DN79161_c1_g1_i8	regulatory protein NPR3-	5028	20
TRUE	[BLASTED]	TRINITY_DN86696_c0_g1_i13	uncharacterized protein	1283	20
TRUE	[BLASTED, I	TRINITY_DN86696_c1_g2_i11	Acetylglucosaminyltransf	2282	20
TRUE	[BLASTED, I	TRINITY_DN86628_c1_g1_i1	BTB/POZ and TAZ domai	2279	20
TRUE	[BLASTED, I	TRINITY_DN86652_c0_g1_i11	protein MEI2-like 5 isofo	971	20
TRUE	[BLASTED, I	TRINITY_DN86652_c1_g1_i13	protein SDA1 homolog is	1432	20
TRUE	[BLASTED, I	TRINITY_DN86688_c1_g4_i8	glutathione reductase, c	977	20
TRUE	[BLASTED, I	TRINITY_DN86658_c0_g1_i7	putative DEAD-box ATP-c	865	20
TRUE	[BLASTED, I	TRINITY_DN86622_c1_g1_i12	alpha-galactosidase 3-like	905	20
TRUE	[BLASTED, I	TRINITY_DN86693_c2_g2_i12	bifunctional nuclease 2-li	1327	20
TRUE	[BLASTED]	TRINITY_DN86603_c1_g1_i8	angio-associated migrato	1722	20
TRUE	[BLASTED, I	TRINITY_DN86690_c3_g1_i10	cell division control prote	2428	20
TRUE	[BLASTED, I	TRINITY_DN86692_c1_g3_i18	Colanic acid biosynthesis	4086	20
TRUE	[BLASTED, I	TRINITY_DN86699_c0_g1_i5	N-acetylglucosaminyl-ph	2326	20
TRUE	[BLASTED]	TRINITY_DN74508_c0_g1_i3	EKC/KEOPS complex, sub	1969	20
TRUE	[BLASTED, I	TRINITY_DN74328_c0_g1_i1	protein SENESCENCE-ASS	845	20
TRUE	[BLASTED]	TRINITY_DN77494_c2_g1_i7	L10-interacting MYB dom	1636	20
TRUE	[BLASTED, I	TRINITY_DN77433_c0_g1_i2	deoxyribodipyrimidine pl	1847	20
TRUE	[BLASTED, I	TRINITY_DN77433_c0_g1_i10	deoxyribodipyrimidine pl	1773	20
TRUE	[BLASTED, I	TRINITY_DN77488_c0_g1_i2	phosphoserine phosphat	682	20
TRUE	[BLASTED, I	TRINITY_DN77488_c1_g5_i3	non-specific phospholipa	1937	20
TRUE	[BLASTED, I	TRINITY_DN77458_c0_g1_i4	CBL-interacting serine/th	1787	20
TRUE	[BLASTED, I	TRINITY_DN77419_c0_g1_i3	transcriptional corepress	2297	20
TRUE	[BLASTED, I	TRINITY_DN77418_c0_g1_i7	retrovirus-related Pol po	2667	20
TRUE	[BLASTED]	TRINITY_DN77456_c2_g4_i7	stem-specific protein TSJ	1043	20
TRUE	[BLASTED, I	TRINITY_DN77439_c1_g1_i13	chaperonin CPN60-like 2	2554	20
TRUE	[BLASTED, I	TRINITY_DN77445_c1_g1_i5	ABC transporter G family	2401	20
TRUE	[BLASTED, I	TRINITY_DN88710_c2_g2_i3	formin-like protein 2	2762	20
TRUE	[BLASTED, I	TRINITY_DN88757_c3_g8_i1	ethylene-responsive tran	1152	20
TRUE	[BLASTED, I	TRINITY_DN88793_c1_g1_i1	6-phosphofructo-2-kinas	4001	20
TRUE	[BLASTED, I	TRINITY_DN88726_c1_g1_i8	DEAD-box ATP-depender	2575	20
TRUE	[BLASTED]	TRINITY_DN88724_c3_g1_i6	LRR receptor-like serine/	1821	20
TRUE	[BLASTED, I	TRINITY_DN88736_c1_g2_i1	coat protein	260	4
TRUE	[BLASTED]	TRINITY_DN88750_c4_g2_i3	B3 domain-containing pr	2886	20
TRUE	[BLASTED]	TRINITY_DN88750_c4_g2_i15	B3 domain-containing pr	2896	20
TRUE	[BLASTED, I	TRINITY_DN88794_c5_g1_i1	SURP and G-patch domai	892	20
TRUE	[BLASTED, I	TRINITY_DN88756_c3_g2_i1	glutamate synthase 1 [N/	3486	20
TRUE	[BLASTED, I	TRINITY_DN88723_c4_g1_i5	CYP76A26-like protein	484	20
TRUE	[BLASTED, I	TRINITY_DN88723_c4_g2_i8	CYP76A26-like protein	1639	20
TRUE	[BLASTED, I	TRINITY_DN88729_c3_g2_i16	phosphoenolpyruvate/pl	1128	20
TRUE	[BLASTED, I	TRINITY_DN79503_c1_g2_i3	FLS	1260	20
TRUE	[BLASTED, I	TRINITY_DN79503_c1_g2_i7	flavonol synthase	1672	20

TRUE	[BLASTED, I	TRINITY_DN79508_c1_g5_i1	uncharacterized protein	747	20
TRUE	[BLASTED, I	TRINITY_DN79563_c0_g1_i5	protein CHROMATIN REN	2454	20
TRUE	[BLASTED, I	TRINITY_DN79500_c2_g2_i2	heat stress transcription	1752	20
TRUE	[BLASTED, I	TRINITY_DN79572_c2_g3_i8	GDT1-like protein 3	4185	20
TRUE	[BLASTED, I	TRINITY_DN79575_c5_g2_i3	cationic amino acid trans	2453	20
TRUE	[BLASTED, I	TRINITY_DN79556_c1_g1_i5	chlorophyll a-b binding p	3418	20
TRUE	[BLASTED, I	TRINITY_DN79557_c0_g1_i10	Folylpolyglutamate synth	1193	20
TRUE	[BLASTED]	TRINITY_DN79591_c0_g3_i1	TPRXL isoform X1	1555	20
TRUE	[BLASTED]	TRINITY_DN79553_c1_g2_i3	ubiquitin carboxyl-termir	1755	3
TRUE	[BLASTED, I	TRINITY_DN79579_c0_g1_i6	UMP-CMP kinase 3-like is	1035	20
TRUE	[BLASTED, I	TRINITY_DN79518_c0_g1_i7	LRR receptor-like serine/	1467	20
TRUE	[BLASTED, I	TRINITY_DN79521_c0_g2_i4	protein NUCLEAR FUSION	2100	20
TRUE	[BLASTED, I	TRINITY_DN79568_c2_g1_i11	thylakoid lumenal 29 kDa	1431	20
TRUE	[BLASTED, I	TRINITY_DN79513_c1_g1_i1	B-box zinc finger protein	1297	20
TRUE	[BLASTED, I	TRINITY_DN79513_c1_g1_i2	B-box zinc finger protein	1468	20
TRUE	[BLASTED, I	TRINITY_DN79513_c1_g10_i1	B-box zinc finger protein	1339	20
TRUE	[BLASTED]	TRINITY_DN79512_c1_g4_i1	uncharacterized protein	518	20
TRUE	[BLASTED, I	TRINITY_DN88804_c7_g4_i1	S-adenosylmethionine sy	437	20
TRUE	[BLASTED, I	TRINITY_DN88804_c7_g5_i1	S-adenosylmethionine sy	434	20
TRUE	[NO-BLAST	TRINITY_DN88804_c7_g6_i1	---NA---	354	
TRUE	[BLASTED, I	TRINITY_DN88827_c2_g4_i2	tRNA-splicing ligase (DUF	902	20
TRUE	[BLASTED, I	TRINITY_DN88846_c3_g2_i13	rRNA intron-encoded hor	836	20
TRUE	[BLASTED]	TRINITY_DN88821_c1_g1_i4	alpha-N-acetylglucosami	2437	20
TRUE	[BLASTED, I	TRINITY_DN77340_c1_g1_i8	haloacid dehalogenase-li	1505	20
TRUE	[BLASTED, I	TRINITY_DN77362_c3_g2_i6	cyclin-dependent kinases	942	20
TRUE	[BLASTED, I	TRINITY_DN77356_c0_g1_i1	carbohydrate binding do	1574	20
TRUE	[NO-BLAST	TRINITY_DN77329_c4_g4_i1	---NA---	546	
TRUE	[BLASTED]	TRINITY_DN77345_c0_g1_i1	pentatricopeptide repeat	2576	20
TRUE	[BLASTED, I	TRINITY_DN77339_c2_g2_i3	PRA1 family protein A1-li	1145	20
TRUE	[BLASTED, I	TRINITY_DN77319_c0_g1_i1	50S ribosomal protein L2	1061	20
TRUE	[BLASTED, I	TRINITY_DN77319_c0_g5_i3	50S ribosomal protein L2	1417	20
TRUE	[BLASTED, I	TRINITY_DN77393_c0_g3_i6	probable alpha-mannosi	2323	20
TRUE	[BLASTED, I	TRINITY_DN77328_c1_g4_i5	pyrrolidone-carboxylate	1123	20
TRUE	[BLASTED, I	TRINITY_DN78354_c0_g2_i2	zinc finger protein CONS	1026	20
TRUE	[BLASTED, I	TRINITY_DN78347_c0_g1_i3	serine carboxypeptidase-	2449	20
TRUE	[BLASTED, I	TRINITY_DN78347_c0_g1_i14	serine carboxypeptidase-	2364	20
TRUE	[BLASTED, I	TRINITY_DN78372_c0_g2_i1	auxin-induced protein AU	1026	20
TRUE	[BLASTED, I	TRINITY_DN78322_c1_g1_i7	NADH dehydrogenase [u	939	20
TRUE	[BLASTED, I	TRINITY_DN78316_c0_g1_i10	1-acyl-sn-glycerol-3-phos	1679	20
TRUE	[BLASTED, I	TRINITY_DN78376_c2_g4_i1	cyclin-B1-2-like	2279	20
TRUE	[BLASTED, I	TRINITY_DN78377_c1_g3_i10	pleiotropic drug resistan	2737	20
TRUE	[BLASTED, I	TRINITY_DN78377_c1_g3_i25	pleiotropic drug resistan	2996	20
TRUE	[BLASTED, I	TRINITY_DN85577_c0_g1_i17	probable methionine--tR	3128	20
TRUE	[BLASTED, I	TRINITY_DN85546_c2_g1_i7	ruBisCO large subunit-bir	2776	20
TRUE	[BLASTED, I	TRINITY_DN85566_c2_g4_i5	chloroplast stem-loop bi	452	20
TRUE	[BLASTED]	TRINITY_DN85536_c0_g1_i9	putative endosomal targ	2042	20
TRUE	[BLASTED]	TRINITY_DN85541_c1_g2_i7	Inhibitor of Bruton tyrosi	2212	20
TRUE	[BLASTED, I	TRINITY_DN85500_c3_g1_i7	probable mediator of RN	2593	20
TRUE	[BLASTED, I	TRINITY_DN85505_c1_g6_i2	uncharacterized protein	1505	20
TRUE	[BLASTED, I	TRINITY_DN85514_c1_g1_i3	peroxisome biogenesis fa	1507	20

TRUE	[BLASTED,	TRINITY_DN85514_c1_g1_i19	mechanosensitive ion ch	7394	20
TRUE	[BLASTED,	TRINITY_DN85185_c0_g1_i2	aquaporin TIP2-1-like	2785	20
TRUE	[BLASTED,	TRINITY_DN85185_c0_g1_i5	aquaporin TIP2-1-like	612	20
TRUE	[BLASTED,	TRINITY_DN85185_c1_g1_i8	probable sucrose-phosph	3621	20
TRUE	[BLASTED,	TRINITY_DN85154_c3_g3_i1	UDP-glycosyltransferase	606	20
TRUE	[BLASTED,	TRINITY_DN85191_c0_g2_i3	DNA oxidative demethyla	1680	20
TRUE	[BLASTED,	TRINITY_DN85196_c1_g1_i1	N-terminal acetyltransfe	4514	20
TRUE	[BLASTED,	TRINITY_DN85106_c0_g1_i11	senescence-associated p	3230	20
TRUE	[BLASTED,	TRINITY_DN85155_c0_g1_i6	chlorophyll a-b binding p	1644	20
TRUE	[BLASTED,	TRINITY_DN85155_c0_g1_i15	chlorophyll a-b binding p	1702	20
TRUE	[BLASTED,	TRINITY_DN85145_c0_g1_i5	zinc finger protein CONS	2723	20
TRUE	[BLASTED,	TRINITY_DN85195_c0_g2_i4	respiratory burst oxidase	2575	20
TRUE	[BLASTED,	TRINITY_DN85119_c0_g1_i24	UTP--glucose-1-phospha	1742	20
TRUE	[BLASTED,	TRINITY_DN85137_c2_g2_i1	ATP-dependent zinc met	788	20
TRUE	[BLASTED,	TRINITY_DN85125_c0_g2_i1	beta-glucosidase-like SF	1977	20
TRUE	[BLASTED,	TRINITY_DN85150_c0_g2_i6	zeaxanthin epoxidase, ch	954	20
TRUE	[BLASTED,	TRINITY_DN85150_c0_g2_i12	zeaxanthin epoxidase, ch	3068	20
TRUE	[BLASTED,	TRINITY_DN85150_c0_g2_i13	zeaxanthin epoxidase	1848	20
TRUE	[BLASTED,	TRINITY_DN85184_c0_g2_i5	myc-like anthocyanin reg	1840	20
TRUE	[BLASTED,	TRINITY_DN85184_c0_g2_i8	myc-like anthocyanin reg	1898	20
TRUE	[BLASTED,	TRINITY_DN85116_c0_g2_i13	UPF0496 protein	2129	20
TRUE	[BLASTED,	TRINITY_DN85179_c1_g5_i1	GATA-type transcription	465	20
TRUE	[BLASTED,	TRINITY_DN85179_c1_g5_i12	GATA-type transcription	2380	20
TRUE	[BLASTED,	TRINITY_DN85179_c1_g5_i14	GATA-type transcription	2873	20
TRUE	[BLASTED,	TRINITY_DN83652_c1_g2_i8	squamosa promoter-bin	2046	20
TRUE	[BLASTED,	TRINITY_DN83649_c0_g4_i1	peroxisomal fatty acid be	1213	20
TRUE	[BLASTED,	TRINITY_DN83615_c0_g1_i6	ATPase family AAA doma	1344	20
TRUE	[BLASTED,	TRINITY_DN83662_c0_g1_i6	switch 2	3217	20
TRUE	[BLASTED]	TRINITY_DN83670_c2_g1_i8	17.3 kDa class II heat sho	925	20
TRUE	[BLASTED,	TRINITY_DN83670_c2_g2_i4	ninja-family protein AFP	2848	20
TRUE	[BLASTED,	TRINITY_DN83666_c4_g2_i11	transcription factor TCP4	1499	20
TRUE	[NO-BLAST	TRINITY_DN83671_c3_g1_i24	---NA---	2268	
TRUE	[BLASTED,	TRINITY_DN83651_c1_g1_i8	protein TPX2	3212	20
TRUE	[NO-BLAST	TRINITY_DN81649_c0_g1_i4	---NA---	476	
TRUE	[BLASTED]	TRINITY_DN81683_c2_g1_i10	hsp70-Hsp90 organizing	1218	20
TRUE	[BLASTED,	TRINITY_DN81683_c2_g1_i11	hsp70-Hsp90 organizing	672	20
TRUE	[BLASTED]	TRINITY_DN81638_c0_g2_i3	protein SGT1 homolog	485	20
TRUE	[BLASTED,	TRINITY_DN81629_c3_g2_i3	fructose-bisphosphate al	1071	20
TRUE	[BLASTED,	TRINITY_DN81655_c1_g1_i23	probable vacuolar amino	1726	20
TRUE	[BLASTED,	TRINITY_DN81626_c0_g1_i8	transmembrane ascorba	1156	20
TRUE	[BLASTED,	TRINITY_DN81694_c0_g1_i2	acid phosphatase 1-like	802	20
TRUE	[BLASTED,	TRINITY_DN81694_c0_g1_i3	acid phosphatase 1-like	629	20
TRUE	[BLASTED,	TRINITY_DN81694_c0_g1_i5	acid phosphatase 1-like	1060	20
TRUE	[BLASTED,	TRINITY_DN81605_c0_g1_i1	F-box protein At3g54460	6230	20
TRUE	[BLASTED,	TRINITY_DN81605_c0_g1_i2	F-box protein At3g54460	4041	20
TRUE	[BLASTED,	TRINITY_DN81644_c2_g2_i7	transcription factor RF2b	1486	20
TRUE	[BLASTED,	TRINITY_DN81685_c1_g2_i3	divinyl chlorophyllide a 8	1829	20
TRUE	[BLASTED,	TRINITY_DN81631_c1_g1_i5	heat shock 70 kDa protei	2725	20
TRUE	[BLASTED,	TRINITY_DN81631_c1_g1_i9	heat shock 70 kDa protei	3079	20
TRUE	[BLASTED]	TRINITY_DN81610_c1_g1_i16	WD repeat-containing pr	1582	20

TRUE	[BLASTED, I	TRINITY_DN81627_c0_g1_i16	transforming growth fact	3573	20
TRUE	[BLASTED, I	TRINITY_DN81642_c1_g2_i3	alpha-(1,4)-fucosyltransf	1744	20
TRUE	[BLASTED, I	TRINITY_DN81625_c0_g1_i8	putative GTP diphosphok	3070	20
TRUE	[BLASTED]	TRINITY_DN81693_c1_g2_i5	UPF0565 protein C2orf69	1499	20
TRUE	[BLASTED, I	TRINITY_DN78209_c1_g1_i1	Aspartate-semialdehyde	1311	20
TRUE	[BLASTED, I	TRINITY_DN78209_c1_g1_i10	putative GPI inositol-dea	1088	20
TRUE	[BLASTED, I	TRINITY_DN78202_c1_g1_i2	uncharacterized membra	1650	20
TRUE	[BLASTED, I	TRINITY_DN78219_c0_g1_i4	histone H3-like centrome	1011	20
TRUE	[BLASTED]	TRINITY_DN83769_c0_g2_i13	uncharacterized protein	993	7
TRUE	[BLASTED]	TRINITY_DN83714_c0_g8_i1	tetrapyrrole-binding prot	1516	20
TRUE	[BLASTED, I	TRINITY_DN83752_c1_g4_i17	CSC1-like protein ERD4	2873	20
TRUE	[BLASTED, I	TRINITY_DN83763_c0_g1_i3	HVA22-like protein	2565	20
TRUE	[BLASTED, I	TRINITY_DN83713_c0_g1_i11	flowering locus K homolo	2448	20
TRUE	[BLASTED, I	TRINITY_DN83734_c1_g2_i11	regulator of nonsense tra	3816	20
TRUE	[BLASTED]	TRINITY_DN83700_c1_g1_i4	DUF724 domain-containi	4538	20
TRUE	[BLASTED]	TRINITY_DN83725_c2_g2_i13	proteoglycan 4	2779	20
TRUE	[BLASTED]	TRINITY_DN83760_c2_g5_i3	plant UBX domain-contai	2387	20
TRUE	[BLASTED]	TRINITY_DN83774_c0_g5_i1	vegetative cell wall prote	988	2
TRUE	[BLASTED, I	TRINITY_DN83733_c0_g1_i9	protein REVEILLE 8	1826	20
TRUE	[BLASTED]	TRINITY_DN79826_c3_g3_i7	F-box only protein 6	1581	20
TRUE	[BLASTED, I	TRINITY_DN79849_c0_g3_i9	hydroxyproline-rich glyco	852	20
TRUE	[BLASTED]	TRINITY_DN79832_c2_g1_i1	protein DEHYDRATION-IN	3387	20
TRUE	[BLASTED, I	TRINITY_DN79869_c1_g3_i4	Sorting nexin-16 like	2135	20
TRUE	[BLASTED]	TRINITY_DN79856_c1_g2_i1	uncharacterized protein	1608	20
TRUE	[NO-BLAST	TRINITY_DN79884_c1_g1_i1	---NA---	421	
TRUE	[BLASTED]	TRINITY_DN79884_c5_g2_i3	dnaJ homolog subfamily	1475	20
TRUE	[BLASTED, I	TRINITY_DN79848_c3_g1_i12	protein LOW PSII ACCUM	1394	20
TRUE	[BLASTED, I	TRINITY_DN79824_c6_g1_i11	B3 domain-containing pr	2734	20
TRUE	[BLASTED, I	TRINITY_DN75918_c0_g1_i4	NAC domain-containing p	1654	20
TRUE	[BLASTED, I	TRINITY_DN75908_c0_g1_i4	outer envelope pore pro	922	20
TRUE	[BLASTED, I	TRINITY_DN75952_c0_g1_i1	CBL-interacting protein k	2380	20
TRUE	[BLASTED, I	TRINITY_DN74976_c0_g1_i2	protein STRICTOSIDINE S	981	20
TRUE	[BLASTED]	TRINITY_DN70058_c0_g1_i3	nuclear transport factor	723	20
TRUE	[BLASTED, I	TRINITY_DN80290_c0_g4_i1	branched-chain-amino-a	2366	20
TRUE	[BLASTED, I	TRINITY_DN80297_c2_g2_i10	heavy metal-associated i	943	20
TRUE	[BLASTED, I	TRINITY_DN80297_c2_g2_i13	heavy metal-associated i	2032	20
TRUE	[BLASTED]	TRINITY_DN80295_c0_g2_i4	phosphatidylinositol/pho	1795	20
TRUE	[BLASTED, I	TRINITY_DN80211_c0_g6_i2	ethylene-responsive tran	839	20
TRUE	[BLASTED]	TRINITY_DN80258_c2_g1_i13	uncharacterized protein	2331	3
TRUE	[BLASTED]	TRINITY_DN80259_c2_g6_i2	uncharacterized protein	1711	20
TRUE	[BLASTED, I	TRINITY_DN80236_c1_g2_i2	root border cell-specific p	2420	20
TRUE	[BLASTED, I	TRINITY_DN80256_c2_g1_i10	uncharacterized protein	1739	20
TRUE	[BLASTED, I	TRINITY_DN80228_c2_g2_i6	neutral ceramidase	2201	20
TRUE	[BLASTED, I	TRINITY_DN80205_c0_g2_i4	chlorophyll a-b binding p	2573	20
TRUE	[BLASTED, I	TRINITY_DN78972_c1_g1_i6	endoplasmic reticulum o	1963	20
TRUE	[BLASTED, I	TRINITY_DN78907_c1_g2_i3	probable adenylate kinas	1623	20
TRUE	[BLASTED, I	TRINITY_DN78997_c1_g5_i2	B-box zinc finger protein	1236	20
TRUE	[BLASTED, I	TRINITY_DN78997_c1_g5_i9	B-box zinc finger protein	1254	20
TRUE	[BLASTED, I	TRINITY_DN78973_c1_g2_i1	THO complex subunit 4A	1519	20
TRUE	[BLASTED, I	TRINITY_DN78947_c4_g2_i4	reticulon-like protein B2	990	20

TRUE	[BLASTED, I	TRINITY_DN78964_c0_g5_i2	flavonoid 3'-hydroxylase	1481	20
TRUE	[BLASTED]	TRINITY_DN78996_c1_g5_i1	17.3 kDa class II heat sho	743	20
TRUE	[BLASTED]	TRINITY_DN78996_c1_g5_i3	17.3 kDa class II heat sho	1034	20
TRUE	[NO-BLAST	TRINITY_DN75611_c1_g1_i1	---NA---	1324	
TRUE	[BLASTED, I	TRINITY_DN75654_c0_g2_i1	uncharacterized protein	849	9
TRUE	[NO-BLAST	TRINITY_DN75631_c2_g1_i4	---NA---	433	
TRUE	[BLASTED, I	TRINITY_DN86779_c1_g2_i2	fe-S cluster assembly fac	2104	20
TRUE	[BLASTED, I	TRINITY_DN86779_c1_g2_i5	fe-S cluster assembly fac	2029	20
TRUE	[BLASTED, I	TRINITY_DN86779_c1_g2_i11	fe-S cluster assembly fac	1976	20
TRUE	[BLASTED, I	TRINITY_DN86774_c0_g2_i4	casein kinase 1-like prote	1505	20
TRUE	[BLASTED, I	TRINITY_DN86704_c0_g2_i5	DExH-box ATP-dependen	3636	20
TRUE	[BLASTED]	TRINITY_DN86749_c2_g2_i7	uncharacterized protein	2704	20
TRUE	[BLASTED]	TRINITY_DN86733_c0_g2_i1	general transcription fact	2114	20
TRUE	[BLASTED, I	TRINITY_DN86719_c0_g4_i6	DNA ligase 4 isoform X1	2315	20
TRUE	[BLASTED, I	TRINITY_DN86705_c1_g1_i11	histone H2B	506	20
TRUE	[BLASTED, I	TRINITY_DN86799_c0_g1_i12	mycophenolic acid acyl-g	1003	20
TRUE	[BLASTED, I	TRINITY_DN86769_c0_g4_i4	potassium transporter 4-	3431	20
TRUE	[BLASTED, I	TRINITY_DN86769_c0_g4_i16	potassium transporter 4-	2847	20
TRUE	[BLASTED]	TRINITY_DN86778_c0_g1_i3	Protein ALEX like	1894	20
TRUE	[BLASTED, I	TRINITY_DN86759_c2_g1_i17	putative glucose-6-phosp	3640	20
TRUE	[BLASTED, I	TRINITY_DN86759_c2_g1_i19	putative glucose-6-phosp	3692	20
TRUE	[BLASTED, I	TRINITY_DN86785_c0_g2_i5	65-kDa microtubule-asso	2053	20
TRUE	[BLASTED, I	TRINITY_DN86793_c2_g1_i6	peroxisome biogenesis p	3146	20
TRUE	[BLASTED, I	TRINITY_DN86732_c3_g1_i4	alpha-L-arabinofuranosic	898	20
TRUE	[BLASTED, I	TRINITY_DN86795_c2_g1_i2	Ca2+-binding transmemb	7012	20
TRUE	[BLASTED, I	TRINITY_DN85887_c1_g3_i1	methyl-CpG-binding dom	1039	20
TRUE	[BLASTED, I	TRINITY_DN85848_c0_g1_i15	ubiquitin-like-specific prc	1283	20
TRUE	[NO-BLAST	TRINITY_DN85836_c0_g3_i8	---NA---	524	
TRUE	[BLASTED, I	TRINITY_DN85836_c0_g1_i4	methionine aminopeptid	2948	20
TRUE	[BLASTED, I	TRINITY_DN85838_c0_g2_i10	splicing factor 1	2327	20
TRUE	[BLASTED]	TRINITY_DN85830_c0_g1_i26	inter alpha-trypsin inhibi	5114	20
TRUE	[BLASTED]	TRINITY_DN85830_c0_g1_i34	inter alpha-trypsin inhibi	3754	20
TRUE	[BLASTED, I	TRINITY_DN85849_c4_g1_i7	dnaJ protein homolog	959	20
TRUE	[BLASTED, I	TRINITY_DN85855_c0_g3_i2	importin subunit beta-1	2618	20
TRUE	[BLASTED, I	TRINITY_DN85840_c2_g3_i5	glucose-1-phosphate ade	2047	20
TRUE	[BLASTED, I	TRINITY_DN85899_c1_g1_i5	probable serine/threonir	3164	20
TRUE	[BLASTED, I	TRINITY_DN85825_c1_g4_i5	probable carboxylesteras	1336	20
TRUE	[BLASTED, I	TRINITY_DN85826_c2_g1_i6	transcription factor bHLH	2310	20
TRUE	[BLASTED, I	TRINITY_DN81974_c3_g1_i4	protein LHY-like	1971	20
TRUE	[BLASTED, I	TRINITY_DN81974_c3_g3_i1	hypothetical protein VITI	1107	15
TRUE	[BLASTED]	TRINITY_DN81985_c0_g1_i1	uncharacterized protein	473	1
TRUE	[BLASTED, I	TRINITY_DN81963_c3_g1_i4	callose synthase 10	6478	20
TRUE	[BLASTED, I	TRINITY_DN81963_c3_g1_i12	callose synthase 10	5861	20
TRUE	[BLASTED, I	TRINITY_DN81978_c4_g2_i1	ATP-dependent RNA heli	963	20
TRUE	[BLASTED, I	TRINITY_DN81957_c4_g1_i3	potassium channel AKT2,	2990	20
TRUE	[BLASTED]	TRINITY_DN81951_c0_g1_i7	plant UBX domain-contai	1134	20
TRUE	[BLASTED, I	TRINITY_DN81979_c0_g1_i7	ethylene-responsive tran	1427	20
TRUE	[BLASTED, I	TRINITY_DN81932_c0_g1_i2	endo-1,3;1,4-beta-D-gluc	1750	20
TRUE	[BLASTED, I	TRINITY_DN81973_c2_g1_i9	17.3 kDa class I heat shoc	652	20
TRUE	[BLASTED, I	TRINITY_DN81945_c1_g1_i10	transcription factor HHO	1281	20

TRUE	[BLASTED]	TRINITY_DN81990_c1_g2_i1	F-box/kelch-repeat prote	3846	20
TRUE	[BLASTED]	TRINITY_DN81990_c1_g2_i15	F-box/kelch-repeat prote	4398	20
TRUE	[BLASTED, I	TRINITY_DN81947_c0_g5_i2	zinc finger protein CONS	1221	20
TRUE	[BLASTED, I	TRINITY_DN81933_c1_g3_i5	anthranilate phosphoribo	1344	20
TRUE	[BLASTED, I	TRINITY_DN83227_c2_g1_i8	outer envelope pore pro	662	20
TRUE	[BLASTED, I	TRINITY_DN83278_c1_g2_i10	phosphoenolpyruvate ca	2610	20
TRUE	[BLASTED, I	TRINITY_DN83207_c0_g1_i3	rab11 family-interacting	2160	20
TRUE	[BLASTED, I	TRINITY_DN83290_c4_g1_i5	aberrant root formation	1385	20
TRUE	[BLASTED, I	TRINITY_DN83275_c4_g2_i1	heat shock 70 kDa protei	2362	20
TRUE	[BLASTED, I	TRINITY_DN83285_c1_g5_i2	auxin efflux carrier comp	2524	20
TRUE	[BLASTED, I	TRINITY_DN83285_c1_g4_i2	auxin:hydrogen symport	377	20
TRUE	[BLASTED, I	TRINITY_DN83243_c0_g1_i18	putative PAP-specific pho	1170	20
TRUE	[BLASTED, I	TRINITY_DN83215_c0_g1_i7	pyruvate decarboxylase	4188	20
TRUE	[BLASTED]	TRINITY_DN83281_c2_g7_i3	BTB/POZ domain-contair	2578	20
TRUE	[BLASTED, I	TRINITY_DN83208_c0_g1_i5	peptidyl-prolyl cis-trans i	1263	20
TRUE	[BLASTED, I	TRINITY_DN83296_c1_g1_i18	5'-methylthioadenosine/	1626	20
TRUE	[BLASTED]	TRINITY_DN83234_c1_g4_i3	Serine/threonine-proteir	2663	20
TRUE	[BLASTED, I	TRINITY_DN83223_c1_g1_i5	zinc finger protein CONS	3996	20
TRUE	[BLASTED, I	TRINITY_DN83219_c1_g1_i6	lipid phosphate phospha	1362	20
TRUE	[BLASTED]	TRINITY_DN83267_c1_g4_i1	17.3 kDa class I heat sho	1019	20
TRUE	[BLASTED, I	TRINITY_DN77563_c1_g5_i5	probable histone H2A va	410	20
TRUE	[BLASTED]	TRINITY_DN77542_c3_g1_i7	thylakoid lumenal 15 kDa	957	20
TRUE	[BLASTED, I	TRINITY_DN77540_c0_g1_i4	anamorsin homolog	1761	20
TRUE	[BLASTED, I	TRINITY_DN77560_c1_g1_i4	putative LOV domain-cor	1518	20
TRUE	[BLASTED, I	TRINITY_DN77572_c2_g3_i1	histone h4	382	20
TRUE	[BLASTED, I	TRINITY_DN77564_c0_g1_i3	4-coumarate--CoA ligase	1912	20
TRUE	[BLASTED, I	TRINITY_DN85388_c4_g4_i9	chloroplast stem-loop bi	1305	20
TRUE	[BLASTED, I	TRINITY_DN85342_c4_g5_i1	bark storage protein A	512	20
TRUE	[BLASTED, I	TRINITY_DN85342_c4_g5_i2	bark storage protein A	831	20
TRUE	[BLASTED, I	TRINITY_DN85342_c4_g3_i12	bark storage protein A	613	20
TRUE	[BLASTED, I	TRINITY_DN85397_c0_g1_i6	uncharacterized protein	2279	20
TRUE	[BLASTED, I	TRINITY_DN85386_c1_g1_i41	ferrochelatase-2, chlorop	937	20
TRUE	[BLASTED, I	TRINITY_DN85339_c2_g2_i2	putative elongation facto	2743	20
TRUE	[BLASTED, I	TRINITY_DN85339_c2_g2_i4	putative elongation facto	2243	20
TRUE	[BLASTED]	TRINITY_DN85360_c0_g1_i6	uncharacterized protein	3977	20
TRUE	[BLASTED, I	TRINITY_DN85365_c0_g2_i4	ultraviolet-B receptor UV	4126	20
TRUE	[BLASTED]	TRINITY_DN85378_c4_g2_i14	uncharacterized protein	1193	20
TRUE	[BLASTED, I	TRINITY_DN85330_c1_g1_i1	zinc finger protein VAR3,	2598	20
TRUE	[BLASTED, I	TRINITY_DN85358_c4_g4_i2	phosphoribulokinase, chl	515	20
TRUE	[BLASTED, I	TRINITY_DN85389_c0_g1_i11	transmembrane 9 superf	4023	20
TRUE	[BLASTED, I	TRINITY_DN85389_c0_g1_i21	transmembrane 9 superf	1500	20
TRUE	[BLASTED, I	TRINITY_DN85389_c0_g1_i25	transmembrane 9 superf	3907	20
TRUE	[BLASTED, I	TRINITY_DN80581_c0_g1_i6	ephrin type-B receptor	1197	20
TRUE	[BLASTED, I	TRINITY_DN80517_c0_g1_i4	phosphoacetylglucosami	3383	20
TRUE	[BLASTED, I	TRINITY_DN80540_c0_g1_i15	chlorophyll a-b binding p	1179	20
TRUE	[BLASTED, I	TRINITY_DN80559_c0_g2_i13	protochlorophyllide-dep	1398	20
TRUE	[BLASTED, I	TRINITY_DN80531_c3_g1_i6	Heat shock factor proteir	3516	20
TRUE	[BLASTED]	TRINITY_DN80545_c0_g2_i2	J protein JJJ2-like	2673	20
TRUE	[BLASTED, I	TRINITY_DN80563_c1_g1_i7	enhancer of rudimentary	1898	20
TRUE	[BLASTED, I	TRINITY_DN80538_c4_g2_i7	double-stranded RNA-bir	2300	20

TRUE	[BLASTED,	TRINITY_DN78578_c0_g1_i4	uncharacterized protein	2739	20
TRUE	[BLASTED,	TRINITY_DN78520_c1_g2_i6	disease resistance protei	3249	20
TRUE	[BLASTED,	TRINITY_DN78533_c0_g1_i16	rab3 GTPase-activating p	1887	20
TRUE	[BLASTED]	TRINITY_DN78597_c0_g1_i13	uncharacterized protein	2751	20
TRUE	[BLASTED]	TRINITY_DN78597_c0_g1_i31	flavoprotein HI0933-like	1297	20
TRUE	[BLASTED,	TRINITY_DN78590_c0_g1_i4	serine/arginine-rich splic	2153	20
TRUE	[BLASTED,	TRINITY_DN78590_c0_g1_i7	serine/arginine-rich splic	2072	20
TRUE	[BLASTED,	TRINITY_DN78547_c0_g1_i7	putative endosulphine	1183	20
TRUE	[BLASTED,	TRINITY_DN78592_c1_g1_i1	lipid phosphate phospha	2708	20
TRUE	[BLASTED]	TRINITY_DN78542_c2_g5_i4	ubiquitin-like protein 5	790	20
TRUE	[BLASTED,	TRINITY_DN78530_c1_g1_i25	mitochondrial substrate	1255	20
TRUE	[BLASTED,	TRINITY_DN78569_c0_g4_i1	photosynthetic NDH subu	358	20
TRUE	[BLASTED,	TRINITY_DN78556_c1_g1_i7	CASP-like protein 2C1	1013	20
TRUE	[BLASTED,	TRINITY_DN78598_c0_g1_i4	Synaptic vesicle transpor	1455	20
TRUE	[BLASTED,	TRINITY_DN78509_c1_g3_i2	squamosa promoter-binc	1634	20
TRUE	[BLASTED,	TRINITY_DN78509_c1_g3_i5	squamosa promoter-binc	1578	20
TRUE	[BLASTED,	TRINITY_DN87100_c0_g2_i1	xylose isomerase	2317	20
TRUE	[BLASTED]	TRINITY_DN87158_c0_g2_i1	ultraviolet-B receptor UV	3732	20
TRUE	[BLASTED,	TRINITY_DN87158_c0_g2_i6	ultraviolet-B receptor UV	918	20
TRUE	[BLASTED]	TRINITY_DN87158_c0_g1_i11	ultraviolet-B receptor UV	1249	20
TRUE	[BLASTED]	TRINITY_DN87158_c0_g1_i12	ultraviolet-B receptor UV	1865	20
TRUE	[BLASTED]	TRINITY_DN87158_c0_g2_i11	ultraviolet-B receptor UV	2364	20
TRUE	[BLASTED]	TRINITY_DN87158_c0_g1_i20	ultraviolet-B receptor UV	1154	20
TRUE	[BLASTED]	TRINITY_DN87158_c0_g2_i14	ultraviolet-B receptor UV	4026	20
TRUE	[NO-BLAST	TRINITY_DN87160_c1_g1_i7	---NA---	1944	
TRUE	[BLASTED,	TRINITY_DN87144_c2_g2_i2	spermatogenesis-associa	2781	20
TRUE	[BLASTED,	TRINITY_DN87144_c2_g2_i24	spermatogenesis-associa	2543	20
TRUE	[BLASTED,	TRINITY_DN87182_c1_g1_i20	NADP-dependent glycera	1345	20
TRUE	[BLASTED,	TRINITY_DN87170_c1_g1_i11	flowering time control pr	3140	20
TRUE	[BLASTED,	TRINITY_DN87152_c2_g2_i3	E3 ubiquitin-protein ligas	1090	20
TRUE	[BLASTED,	TRINITY_DN87124_c0_g1_i8	zinc finger CCCH domain-	3187	20
TRUE	[BLASTED,	TRINITY_DN87190_c4_g2_i2	protein SUPPRESSOR OF	1936	20
TRUE	[BLASTED,	TRINITY_DN87104_c0_g1_i5	calmodulin-binding trans	2951	20
TRUE	[NO-BLAST	TRINITY_DN87163_c2_g1_i12	---NA---	1509	
TRUE	[BLASTED,	TRINITY_DN87118_c2_g3_i14	probable acyl-activating	2734	20
TRUE	[BLASTED,	TRINITY_DN87178_c0_g1_i7	cytochrome P450 71A6-l	1962	20
TRUE	[BLASTED,	TRINITY_DN87178_c0_g1_i9	cytochrome P450 71A6-l	1548	20
TRUE	[BLASTED,	TRINITY_DN87178_c0_g4_i4	cytochrome P450 71A6-l	616	20
TRUE	[BLASTED,	TRINITY_DN87178_c0_g1_i12	cytochrome P450 71A6-l	1569	20
TRUE	[BLASTED,	TRINITY_DN87108_c2_g2_i2	cytochrome P450 CYP72	1520	20
TRUE	[BLASTED,	TRINITY_DN87108_c2_g7_i2	cytochrome P450 CYP72	450	20
TRUE	[BLASTED,	TRINITY_DN87108_c2_g2_i10	cytochrome P450 CYP72	1736	20
TRUE	[BLASTED,	TRINITY_DN87180_c3_g1_i2	stromal 70 kDa heat shoc	1302	20
TRUE	[BLASTED,	TRINITY_DN87112_c1_g1_i3	malate dehydrogenase, g	1570	20
TRUE	[BLASTED,	TRINITY_DN87112_c1_g1_i12	malate dehydrogenase, g	2334	20
TRUE	[BLASTED,	TRINITY_DN87166_c2_g1_i4	glycerate dehydrogenase	1447	20
TRUE	[BLASTED,	TRINITY_DN87166_c2_g2_i1	glycerate dehydrogenase	315	20
TRUE	[BLASTED,	TRINITY_DN87136_c1_g1_i6	protein transport proteir	1952	20
TRUE	[BLASTED,	TRINITY_DN87146_c1_g4_i4	polyadenylate-binding pr	2280	20
TRUE	[BLASTED,	TRINITY_DN87128_c2_g1_i1	U1 small nuclear ribonuc	1508	20

TRUE	[BLASTED, I	TRINITY_DN87128_c2_g1_i6	U1 small nuclear ribonuc	1447	20
TRUE	[BLASTED, I	TRINITY_DN87549_c0_g2_i8	Copper amine oxidase	2283	20
TRUE	[BLASTED]	TRINITY_DN87550_c0_g1_i1	uncharacterized protein	1667	6
TRUE	[BLASTED, I	TRINITY_DN87512_c0_g1_i13	glucose-1-phosphate ade	2705	20
TRUE	[BLASTED, I	TRINITY_DN87512_c0_g1_i40	glucose-1-phosphate ade	2425	20
TRUE	[BLASTED, I	TRINITY_DN87586_c1_g3_i3	glutamate--glyoxylate am	1299	20
TRUE	[BLASTED, I	TRINITY_DN87521_c1_g4_i3	probable LRR receptor-lil	3344	20
TRUE	[BLASTED, I	TRINITY_DN87521_c1_g7_i2	membrane steroid-bindi	1307	20
TRUE	[BLASTED, I	TRINITY_DN87504_c1_g1_i18	auxin response factor 19	3768	20
TRUE	[BLASTED, I	TRINITY_DN87555_c1_g1_i1	callose synthase 9	8773	20
TRUE	[BLASTED, I	TRINITY_DN87555_c1_g1_i3	callose synthase 9	8856	20
TRUE	[BLASTED, I	TRINITY_DN87571_c0_g2_i3	aquaporin TIP2-1	850	20
TRUE	[BLASTED, I	TRINITY_DN87593_c3_g1_i2	nuclear export mediator	1760	20
TRUE	[BLASTED, I	TRINITY_DN87593_c3_g1_i3	nuclear export mediator	4324	20
TRUE	[BLASTED, I	TRINITY_DN87554_c3_g1_i2	protein FAR-RED ELONG/	4512	20
TRUE	[BLASTED, I	TRINITY_DN87559_c2_g1_i5	serine/threonine-protein	2013	20
TRUE	[BLASTED, I	TRINITY_DN87570_c0_g2_i3	probable Xaa-Pro aminop	2737	20
TRUE	[BLASTED, I	TRINITY_DN87528_c0_g2_i1	magnesium-chelatase su	4437	20
TRUE	[BLASTED, I	TRINITY_DN87518_c0_g3_i3	probable sucrose-phosph	2461	20
TRUE	[BLASTED, I	TRINITY_DN87579_c0_g1_i1	rust resistance kinase Lr1	2978	20
TRUE	[BLASTED, I	TRINITY_DN83360_c2_g1_i19	ABC transporter B family	2375	20
TRUE	[BLASTED, I	TRINITY_DN83317_c2_g2_i25	splicing factor U2af small	3547	20
TRUE	[BLASTED]	TRINITY_DN83364_c0_g1_i2	peroxisomal 2,4-dienoyl-	2177	20
TRUE	[BLASTED, I	TRINITY_DN83330_c1_g1_i4	protein VAC14 homolog	1789	20
TRUE	[BLASTED, I	TRINITY_DN83306_c1_g3_i10	subtilisin-like protease SI	2510	20
TRUE	[BLASTED, I	TRINITY_DN83393_c0_g1_i5	peptidyl-prolyl cis-trans i	3089	20
TRUE	[BLASTED, I	TRINITY_DN83343_c0_g2_i4	uncharacterized membra	1627	20
TRUE	[BLASTED, I	TRINITY_DN83379_c1_g1_i9	conserved oligomeric Go	6100	20
TRUE	[BLASTED, I	TRINITY_DN83320_c0_g3_i5	dol-P-Man:Man(7)GlcNA	1389	20
TRUE	[BLASTED, I	TRINITY_DN83302_c3_g3_i20	polypyrimidine tract-binc	1719	20
TRUE	[BLASTED, I	TRINITY_DN83302_c3_g3_i21	polypyrimidine tract-binc	1386	20
TRUE	[BLASTED, I	TRINITY_DN83365_c0_g2_i3	protein CHLORORESPIRA	1844	20
TRUE	[BLASTED, I	TRINITY_DN83365_c0_g2_i8	protein CHLORORESPIRA	1914	20
TRUE	[BLASTED, I	TRINITY_DN83368_c2_g3_i17	methylthioribose-1-phos	2187	20
TRUE	[BLASTED, I	TRINITY_DN83323_c1_g2_i12	LCM domain-containing	735	20
TRUE	[BLASTED, I	TRINITY_DN83369_c0_g1_i5	phenylalanine--tRNA liga	1577	20
TRUE	[BLASTED, I	TRINITY_DN83369_c1_g1_i23	proline transporter 1-like	1492	20
TRUE	[BLASTED, I	TRINITY_DN87333_c0_g3_i1	phosphatidylinositol/pho	814	20
TRUE	[NO-BLAST	TRINITY_DN87384_c1_g3_i2	---NA---	622	
TRUE	[BLASTED, I	TRINITY_DN87388_c3_g1_i15	lecithin-cholesterol acylt	1627	20
TRUE	[BLASTED, I	TRINITY_DN87348_c1_g4_i5	homeobox-leucine zippe	1073	20
TRUE	[BLASTED]	TRINITY_DN87355_c1_g3_i11	UPF0400 protein C337.0	1975	20
TRUE	[BLASTED, I	TRINITY_DN87301_c3_g2_i9	retrotransposon protein,	4914	20
TRUE	[BLASTED, I	TRINITY_DN87367_c0_g3_i6	protein STRUBBELIG-REC	1906	20
TRUE	[BLASTED, I	TRINITY_DN87396_c2_g1_i22	ATPase 11, plasma meml	1251	20
TRUE	[BLASTED, I	TRINITY_DN87390_c2_g1_i6	glycine dehydrogenase (c	2555	20
TRUE	[BLASTED, I	TRINITY_DN87390_c2_g1_i8	glycine dehydrogenase (c	2076	20
TRUE	[BLASTED, I	TRINITY_DN87340_c1_g7_i1	rhodanese-like domain-c	1021	20
TRUE	[BLASTED, I	TRINITY_DN87356_c2_g1_i11	GDP-L-galactose phosph	369	4
TRUE	[BLASTED, I	TRINITY_DN87313_c2_g3_i1	elongation factor Tu, mit	1769	20

TRUE	[BLASTED,	TRINITY_DN87313_c2_g3_i8	elongation factor Tu, mit	1826	20
TRUE	[NO-BLAST	TRINITY_DN72088_c0_g1_i1	---NA---	903	
TRUE	[NO-BLAST	TRINITY_DN72088_c0_g1_i2	---NA---	837	
TRUE	[BLASTED,	TRINITY_DN75430_c0_g1_i1	transmembrane protein	2212	20
TRUE	[BLASTED,	TRINITY_DN75409_c0_g1_i6	aspartic proteinase-like p	2385	20
TRUE	[BLASTED,	TRINITY_DN70413_c0_g1_i2	metal tolerance protein I	1745	20
TRUE	[BLASTED,	TRINITY_DN78433_c1_g1_i4	putative late blight resist	3740	20
TRUE	[BLASTED,	TRINITY_DN78433_c1_g1_i5	putative late blight resist	3012	20
TRUE	[BLASTED,	TRINITY_DN78450_c1_g5_i2	RNA polymerase sigma fa	2122	20
TRUE	[BLASTED,	TRINITY_DN78450_c1_g3_i4	RNA polymerase sigma fa	775	20
TRUE	[BLASTED,	TRINITY_DN78458_c0_g3_i2	protein NETWORKED 1A-	6586	20
TRUE	[BLASTED]	TRINITY_DN78424_c2_g2_i2	protein PLASTID MOVEM	1247	20
TRUE	[BLASTED,	TRINITY_DN78409_c1_g7_i2	BAG family molecular cha	1266	20
TRUE	[BLASTED,	TRINITY_DN78430_c3_g5_i5	histone H4	675	20
TRUE	[BLASTED,	TRINITY_DN78488_c2_g3_i1	ABC transporter G family	2972	20
TRUE	[BLASTED,	TRINITY_DN78488_c2_g4_i2	B-box zinc finger protein	1426	20
TRUE	[BLASTED,	TRINITY_DN78406_c0_g1_i7	RING-H2 finger protein A	1339	20
TRUE	[BLASTED,	TRINITY_DN65813_c0_g1_i2	hemoglobin subunit alph	558	20
TRUE	[BLASTED]	TRINITY_DN83909_c2_g1_i13	protein LNK4 isoform X1	638	4
TRUE	[BLASTED]	TRINITY_DN83971_c0_g2_i6	golgin candidate 4	2878	20
TRUE	[BLASTED,	TRINITY_DN83989_c0_g4_i3	C-terminal binding prote	2694	20
TRUE	[BLASTED]	TRINITY_DN83902_c0_g1_i18	oxysterol-binding protein	2187	20
TRUE	[BLASTED,	TRINITY_DN83917_c1_g1_i3	photosystem II 22 kDa pr	502	20
TRUE	[BLASTED,	TRINITY_DN83998_c0_g1_i6	protein indeterminate-do	2025	20
TRUE	[BLASTED,	TRINITY_DN83967_c2_g1_i2	LRR receptor-like serine/	3442	20
TRUE	[BLASTED,	TRINITY_DN83972_c2_g1_i6	pentatricopeptide repeat	3619	20
TRUE	[BLASTED,	TRINITY_DN83980_c1_g1_i3	proteasome subunit alph	1170	20
TRUE	[BLASTED,	TRINITY_DN83975_c2_g2_i9	caffeoyl-CoA O-methyltra	1813	20
TRUE	[BLASTED,	TRINITY_DN83938_c0_g1_i15	probable galactinol--sucr	2456	20
TRUE	[BLASTED,	TRINITY_DN82744_c2_g1_i3	protease Do-like 2, chlor	1567	20
TRUE	[BLASTED,	TRINITY_DN82731_c0_g2_i3	zinc finger protein CONS	566	20
TRUE	[BLASTED,	TRINITY_DN82746_c1_g1_i8	2-oxoisovalerate dehydro	1864	20
TRUE	[BLASTED,	TRINITY_DN82705_c2_g1_i4	nuclear transcription fact	2121	20
TRUE	[BLASTED,	TRINITY_DN82758_c0_g1_i10	vacuolar protein sorting-	1501	20
TRUE	[BLASTED,	TRINITY_DN82758_c0_g1_i17	vacuolar protein sorting-	1546	20
TRUE	[BLASTED,	TRINITY_DN82755_c5_g1_i3	Transmembrane protein	1169	20
TRUE	[BLASTED,	TRINITY_DN82702_c2_g1_i7	plant UBX domain-contai	1702	20
TRUE	[BLASTED,	TRINITY_DN82786_c0_g1_i14	protein DETOXIFICATION	1672	20
TRUE	[BLASTED,	TRINITY_DN82786_c1_g2_i3	pto-interacting protein 1	853	20
TRUE	[BLASTED,	TRINITY_DN82742_c0_g2_i1	PTI1-like tyrosine-protein	1775	20
TRUE	[BLASTED]	TRINITY_DN82757_c1_g1_i1	uncharacterized protein	848	20
TRUE	[BLASTED,	TRINITY_DN82733_c0_g1_i3	bidirectional sugar transp	921	20
TRUE	[BLASTED,	TRINITY_DN82779_c1_g1_i5	myosin-2 heavy chain	3607	20
TRUE	[BLASTED]	TRINITY_DN82728_c0_g1_i9	Zinc finger C3H1 domain-	5445	20
TRUE	[BLASTED,	TRINITY_DN87090_c0_g1_i10	vacuolar protein sorting-	6052	20
TRUE	[BLASTED,	TRINITY_DN87090_c0_g1_i31	vacuolar protein sorting-	4360	20
TRUE	[BLASTED,	TRINITY_DN87033_c0_g2_i1	Alanine--tRNA ligase	3514	20
TRUE	[BLASTED,	TRINITY_DN87033_c0_g2_i6	Alanine--tRNA ligase	3477	20
TRUE	[BLASTED,	TRINITY_DN87033_c0_g2_i7	Alanine--tRNA ligase	3399	20
TRUE	[BLASTED,	TRINITY_DN87038_c2_g1_i6	squamosa promoter-bind	3486	20

TRUE	[BLASTED, I	TRINITY_DN87038_c2_g1_i8	squamosa promoter-binc	2919	20
TRUE	[BLASTED]	TRINITY_DN87065_c1_g1_i11	actin-related protein 4	740	20
TRUE	[BLASTED, I	TRINITY_DN87080_c0_g2_i6	serine hydroxymethyltra	2718	20
TRUE	[BLASTED]	TRINITY_DN87063_c0_g3_i8	upstream activation fact	1627	4
TRUE	[BLASTED]	TRINITY_DN87095_c2_g2_i4	uncharacterized protein	2704	20
TRUE	[BLASTED, I	TRINITY_DN87066_c0_g1_i1	acetyl-coenzyme A carbc	1934	20
TRUE	[BLASTED, I	TRINITY_DN87018_c0_g1_i6	protein-tyrosine-phospha	3739	20
TRUE	[BLASTED, I	TRINITY_DN87041_c0_g2_i12	ABC transporter G family	3895	20
TRUE	[BLASTED, I	TRINITY_DN87006_c0_g5_i2	transcription terminatior	2830	20
TRUE	[BLASTED]	TRINITY_DN87010_c1_g2_i5	transcription elongation	3289	3
TRUE	[BLASTED, I	TRINITY_DN87089_c0_g1_i16	V-type proton ATPase su	3738	20
TRUE	[BLASTED, I	TRINITY_DN87053_c0_g1_i9	probable protein phosph	3225	20
TRUE	[BLASTED, I	TRINITY_DN87053_c0_g1_i10	probable protein phosph	2726	20
TRUE	[BLASTED]	TRINITY_DN87035_c0_g4_i6	protein ACCUMULATION	2641	20
TRUE	[BLASTED]	TRINITY_DN87030_c1_g1_i5	CLK4-associating serine/a	4654	20
TRUE	[BLASTED]	TRINITY_DN87030_c1_g3_i5	CLK4-associating serine/a	1547	20
TRUE	[BLASTED, I	TRINITY_DN87059_c1_g2_i24	4-hydroxy-3-methylbut-2	1096	20
TRUE	[BLASTED, I	TRINITY_DN87091_c2_g1_i17	calcium-transporting ATF	510	20
TRUE	[BLASTED]	TRINITY_DN87055_c0_g1_i2	protein OPAQUE10-like	3204	20
TRUE	[BLASTED, I	TRINITY_DN87008_c0_g4_i3	uncharacterized protein	2494	20
TRUE	[BLASTED, I	TRINITY_DN87629_c1_g3_i4	cytochrome b6-f comple:	485	20
TRUE	[BLASTED]	TRINITY_DN87627_c3_g1_i5	putative late blight resist	4404	20
TRUE	[BLASTED]	TRINITY_DN87627_c3_g1_i11	putative late blight resist	3682	20
TRUE	[BLASTED, I	TRINITY_DN87648_c0_g2_i12	cyclin-dependent kinase	1861	20
TRUE	[BLASTED, I	TRINITY_DN87606_c1_g3_i10	calmodulin-binding trans	2785	20
TRUE	[BLASTED, I	TRINITY_DN87667_c0_g2_i5	conserved oligomeric Go	2698	20
TRUE	[BLASTED, I	TRINITY_DN87692_c0_g1_i4	ABC transporter A family	3281	20
TRUE	[BLASTED, I	TRINITY_DN87670_c0_g1_i23	SCY1-like protein 2	3206	20
TRUE	[BLASTED, I	TRINITY_DN87613_c4_g1_i2	zinc finger protein CONS	1245	20
TRUE	[BLASTED, I	TRINITY_DN87613_c5_g1_i1	plastocyanin	705	20
TRUE	[BLASTED, I	TRINITY_DN87613_c5_g1_i2	plastocyanin, chloroplast	419	20
TRUE	[BLASTED, I	TRINITY_DN87613_c5_g1_i4	plastocyanin, chloroplast	630	20
TRUE	[BLASTED, I	TRINITY_DN87613_c5_g1_i5	plastocyanin, chloroplast	589	20
TRUE	[BLASTED, I	TRINITY_DN87658_c1_g4_i6	alpha-L-arabinofuranosic	690	20
TRUE	[BLASTED, I	TRINITY_DN87649_c2_g1_i7	Protein kinase superfami	5282	20
TRUE	[BLASTED, I	TRINITY_DN87628_c1_g4_i3	pumilio homolog 2-like is	2111	20
TRUE	[NO-BLAST	TRINITY_DN87676_c0_g4_i2	---NA---	330	
TRUE	[BLASTED, I	TRINITY_DN87681_c1_g2_i8	endonuclease MutS2	1362	20
TRUE	[BLASTED, I	TRINITY_DN87611_c1_g2_i12	pentatricopeptide repeat	2013	20
TRUE	[BLASTED, I	TRINITY_DN87688_c1_g2_i3	plasma membrane ATPas	1727	20
TRUE	[BLASTED, I	TRINITY_DN87684_c0_g3_i5	chaperone protein ClpC,	1320	20
TRUE	[BLASTED, I	TRINITY_DN87614_c1_g3_i7	sterol 3-beta-glucosyltra	1323	20
TRUE	[BLASTED, I	TRINITY_DN87631_c1_g1_i4	protein ABSCISIC ACID-IN	3581	20
TRUE	[BLASTED, I	TRINITY_DN80669_c1_g1_i5	cytochrome P450 98A2-l	4287	20
TRUE	[BLASTED, I	TRINITY_DN80669_c1_g1_i8	cytochrome P450 98A2-l	3437	20
TRUE	[BLASTED, I	TRINITY_DN80648_c1_g1_i10	cytochrome P450 71A6-l	611	20
TRUE	[BLASTED, I	TRINITY_DN80679_c1_g1_i3	lon protease homolog 1,	2189	20
TRUE	[BLASTED, I	TRINITY_DN80633_c0_g2_i7	zinc finger protein 4-like	1603	20
TRUE	[BLASTED, I	TRINITY_DN80601_c0_g1_i6	elongation factor 1-delta	1417	20
TRUE	[BLASTED, I	TRINITY_DN80650_c0_g3_i2	probable E3 ubiquitin-pr	736	20

TRUE	[BLASTED, I	TRINITY_DN80675_c1_g1_i8	O-glucosyltransferase ru	1009	20
TRUE	[BLASTED, I	TRINITY_DN80642_c1_g1_i3	probable calcium-binding	3012	20
TRUE	[BLASTED, I	TRINITY_DN77754_c2_g3_i6	annexin D5	2905	20
TRUE	[BLASTED, I	TRINITY_DN77717_c2_g2_i2	E3 ubiquitin-protein ligas	882	20
TRUE	[NO-BLAST	TRINITY_DN77778_c2_g1_i2	---NA---	956	
TRUE	[BLASTED, I	TRINITY_DN77739_c2_g1_i1	prohibitin-1, mitochondr	2151	20
TRUE	[BLASTED, I	TRINITY_DN77739_c2_g1_i2	prohibitin-1, mitochondr	1467	20
TRUE	[BLASTED, I	TRINITY_DN77739_c2_g1_i9	prohibitin-1, mitochondr	1522	20
TRUE	[BLASTED, I	TRINITY_DN77790_c0_g7_i2	E3 ubiquitin-protein ligas	1057	20
TRUE	[BLASTED, I	TRINITY_DN77788_c0_g1_i6	retrotransposon protein,	2062	20
TRUE	[BLASTED, I	TRINITY_DN77748_c4_g1_i1	carboxymethylenebuten	1591	20
TRUE	[BLASTED, I	TRINITY_DN77748_c4_g1_i2	carboxymethylenebuten	1944	20
TRUE	[BLASTED, I	TRINITY_DN77748_c5_g2_i2	glutamate decarboxylase	1174	20
TRUE	[BLASTED, I	TRINITY_DN77787_c0_g2_i6	serine/threonine-protein	1315	20
TRUE	[BLASTED]	TRINITY_DN74725_c0_g1_i1	titin-like	687	1
TRUE	[BLASTED]	TRINITY_DN74245_c0_g1_i1	uncharacterized protein	1807	1
TRUE	[NO-BLAST	TRINITY_DN66081_c0_g1_i1	---NA---	808	
TRUE	[NO-BLAST	TRINITY_DN66077_c0_g1_i1	---NA---	1267	
TRUE	[BLASTED]	TRINITY_DN76515_c0_g1_i6	BTB/POZ domain-contair	1344	20
TRUE	[BLASTED, I	TRINITY_DN76548_c0_g2_i11	autophagy-related prote	3025	20
TRUE	[BLASTED, I	TRINITY_DN82944_c0_g1_i9	cleavage stimulating fact	2186	20
TRUE	[BLASTED, I	TRINITY_DN82931_c0_g1_i2	small G protein signaling	5163	20
TRUE	[BLASTED, I	TRINITY_DN82902_c2_g2_i19	glucose-6-phosphate isoi	1260	20
TRUE	[BLASTED, I	TRINITY_DN82945_c0_g1_i5	acylamino-acid-releasing	2908	20
TRUE	[BLASTED, I	TRINITY_DN82950_c1_g2_i4	AP-5 complex subunit ze	4464	20
TRUE	[BLASTED]	TRINITY_DN82989_c1_g2_i9	uncharacterized protein	1960	20
TRUE	[NO-BLAST	TRINITY_DN82929_c1_g2_i1	---NA---	1656	
TRUE	[BLASTED, I	TRINITY_DN82943_c1_g1_i9	stress enhanced protein	2324	20
TRUE	[BLASTED, I	TRINITY_DN82940_c4_g1_i14	pullulanase 1, chloroplas	2957	20
TRUE	[BLASTED, I	TRINITY_DN82955_c2_g4_i1	transcription factor MYB	478	20
TRUE	[BLASTED]	TRINITY_DN82996_c1_g3_i2	putative late blight resist	1783	20
TRUE	[BLASTED]	TRINITY_DN82984_c2_g1_i2	cinnamoyl-CoA reductase	1131	1
TRUE	[BLASTED, I	TRINITY_DN82962_c3_g1_i2	probable RNA-binding pr	1115	20
TRUE	[BLASTED, I	TRINITY_DN87796_c0_g1_i20	magnesium transporter I	3235	20
TRUE	[BLASTED]	TRINITY_DN87728_c0_g7_i1	probable transcriptional	2156	20
TRUE	[BLASTED, I	TRINITY_DN87707_c1_g1_i2	serine/threonine-protein	2577	20
TRUE	[BLASTED]	TRINITY_DN87712_c0_g2_i6	Germ cell-less protein-lik	2588	20
TRUE	[BLASTED, I	TRINITY_DN87790_c2_g1_i4	protein LHY-like isoform	1887	20
TRUE	[BLASTED, I	TRINITY_DN87790_c2_g1_i5	protein LHY-like isoform	1843	20
TRUE	[BLASTED, I	TRINITY_DN87790_c2_g1_i6	protein LHY isoform X1	3771	20
TRUE	[BLASTED, I	TRINITY_DN87790_c2_g1_i7	protein LHY isoform X1	3124	20
TRUE	[BLASTED, I	TRINITY_DN87790_c2_g5_i2	protein LHY-like	1583	20
TRUE	[BLASTED, I	TRINITY_DN87762_c3_g5_i1	peroxisomal (S)-2-hydrox	427	20
TRUE	[BLASTED, I	TRINITY_DN87730_c1_g1_i19	calcium sensing receptor	2264	20
TRUE	[BLASTED, I	TRINITY_DN87720_c0_g2_i6	1,4-alpha-glucan-branchi	1888	20
TRUE	[BLASTED, I	TRINITY_DN87766_c1_g7_i4	B-box zinc finger protein	1300	20
TRUE	[BLASTED, I	TRINITY_DN87737_c1_g1_i7	kinesin-like protein KIN-1	2184	20
TRUE	[BLASTED, I	TRINITY_DN87754_c1_g6_i1	glycolate oxidase	237	20
TRUE	[BLASTED, I	TRINITY_DN87754_c1_g8_i2	peroxisomal (S)-2-hydrox	974	20
TRUE	[BLASTED, I	TRINITY_DN87754_c1_g1_i9	peroxisomal (S)-2-hydrox	390	20

TRUE	[BLASTED, I	TRINITY_DN87747_c1_g2_i10	phosphatidylinositol:cera	2285	20
TRUE	[BLASTED, I	TRINITY_DN87745_c0_g1_i2	eukaryotic translation ini	2193	20
TRUE	[BLASTED, I	TRINITY_DN87745_c0_g1_i13	eukaryotic translation ini	1616	20
TRUE	[BLASTED]	TRINITY_DN87784_c1_g3_i1	Glyoxalase-like domain p	515	20
TRUE	[BLASTED]	TRINITY_DN87784_c1_g3_i3	Glyoxalase-like domain p	703	20
TRUE	[BLASTED, I	TRINITY_DN87722_c2_g3_i6	SNARE-interacting protei	2946	20
TRUE	[BLASTED, I	TRINITY_DN87774_c0_g3_i9	probable LRR receptor-lil	1056	20
TRUE	[BLASTED, I	TRINITY_DN87727_c2_g3_i2	putative pre-16S rRNA nu	927	20
TRUE	[NO-BLAST	TRINITY_DN71870_c0_g1_i1	---NA---	1259	
TRUE	[BLASTED, I	TRINITY_DN76091_c0_g4_i2	peptidyl-prolyl cis-trans i	826	20
TRUE	[BLASTED]	TRINITY_DN76080_c0_g1_i2	abscisic acid receptor PYI	1069	20
TRUE	[BLASTED, I	TRINITY_DN78887_c2_g3_i6	probable phosphoinositi	5409	20
TRUE	[BLASTED, I	TRINITY_DN78887_c2_g2_i3	pathogenesis-related prc	883	20
TRUE	[BLASTED]	TRINITY_DN78805_c0_g1_i11	uncharacterized protein	2738	20
TRUE	[BLASTED, I	TRINITY_DN78805_c0_g1_i16	protein trichome birefrin	3516	20
TRUE	[BLASTED, I	TRINITY_DN78821_c3_g5_i8	DNA-directed RNA polym	2424	20
TRUE	[BLASTED, I	TRINITY_DN78848_c1_g1_i8	ubiquitin-conjugating en:	600	20
TRUE	[BLASTED, I	TRINITY_DN78834_c1_g1_i2	carbonic anhydrase 2-like	1443	20
TRUE	[NO-BLAST	TRINITY_DN78846_c0_g6_i2	---NA---	1188	
TRUE	[BLASTED, I	TRINITY_DN78846_c0_g12_i1	ethylene-responsive tran	839	20
TRUE	[BLASTED]	TRINITY_DN78803_c2_g1_i9	Cell wall integrity transcr	2019	20
TRUE	[BLASTED]	TRINITY_DN78840_c1_g1_i4	pentatricopeptide repeat	2185	20
TRUE	[BLASTED]	TRINITY_DN78849_c2_g3_i3	uncharacterized protein	645	3
TRUE	[BLASTED]	TRINITY_DN78849_c2_g3_i7	uncharacterized protein	957	3
TRUE	[BLASTED, I	TRINITY_DN78875_c2_g1_i1	GTP-binding protein ERG	3272	20
TRUE	[BLASTED]	TRINITY_DN78877_c2_g1_i2	uncharacterized protein	927	20
TRUE	[BLASTED]	TRINITY_DN86046_c3_g6_i1	rhodanese-like domain-c	649	20
TRUE	[BLASTED, I	TRINITY_DN86072_c1_g2_i3	auxin response factor 19	3032	20
TRUE	[BLASTED, I	TRINITY_DN86072_c1_g2_i8	auxin response factor 19	3002	20
TRUE	[BLASTED, I	TRINITY_DN86018_c0_g1_i6	transcription factor E2FB	2875	20
TRUE	[BLASTED, I	TRINITY_DN86018_c0_g1_i21	transcription factor E2FB	2914	20
TRUE	[BLASTED, I	TRINITY_DN86050_c1_g1_i1	polyadenylation and clea	1186	20
TRUE	[BLASTED, I	TRINITY_DN86064_c1_g1_i8	protein BTR1-like isoforn	2488	20
TRUE	[BLASTED]	TRINITY_DN86084_c1_g1_i5	uncharacterized protein	967	3
TRUE	[BLASTED, I	TRINITY_DN86051_c2_g2_i13	galactinol synthase 2-like	470	20
TRUE	[BLASTED, I	TRINITY_DN86051_c2_g2_i20	galactinol synthase 1	2217	20
TRUE	[BLASTED, I	TRINITY_DN86040_c0_g1_i7	midasin isoform X2	5005	20
TRUE	[BLASTED, I	TRINITY_DN86085_c1_g4_i4	putative potassium trans	2836	20
TRUE	[BLASTED]	TRINITY_DN86076_c1_g5_i1	uncharacterized protein	7024	20
TRUE	[BLASTED, I	TRINITY_DN86039_c1_g3_i5	homeobox protein LUMI	1668	20
TRUE	[BLASTED, I	TRINITY_DN86086_c2_g2_i9	probable NOT transcripti	1989	20
TRUE	[BLASTED, I	TRINITY_DN65695_c0_g1_i1	cytochrome oxidase subu	1434	20
TRUE	[BLASTED, I	TRINITY_DN77813_c2_g2_i8	F-box protein	2104	20
TRUE	[BLASTED, I	TRINITY_DN77808_c4_g1_i2	protein ABHD17C-like	1249	20
TRUE	[BLASTED, I	TRINITY_DN77832_c0_g1_i6	protease 2	2375	20
TRUE	[BLASTED, I	TRINITY_DN77816_c0_g5_i3	GDP-mannose 3,5-epime	1701	20
TRUE	[BLASTED, I	TRINITY_DN77819_c1_g5_i1	40s ribosomal protein s1	700	20
TRUE	[BLASTED, I	TRINITY_DN77865_c2_g8_i1	autophagy-related prote	1605	20
TRUE	[BLASTED, I	TRINITY_DN77859_c3_g4_i1	serine/threonine-proteir	3880	20
TRUE	[BLASTED, I	TRINITY_DN84032_c2_g1_i13	zinc finger CCCH domain-	3045	20

TRUE	[BLASTED, I	TRINITY_DN84043_c0_g2_i4	secoisolariciresinol dehy	1100	20
TRUE	[BLASTED, I	TRINITY_DN84087_c3_g4_i1	two-component respons	1074	20
TRUE	[BLASTED, I	TRINITY_DN84056_c1_g1_i6	tropinone reductase hon	1177	20
TRUE	[BLASTED, I	TRINITY_DN84091_c0_g4_i1	putative lipase/calmodul	1980	20
TRUE	[BLASTED, I	TRINITY_DN84078_c0_g4_i10	clathrin interactor EPSIN	1634	20
TRUE	[BLASTED, I	TRINITY_DN84003_c1_g1_i1	integrin-linked protein ki	2942	20
TRUE	[BLASTED, I	TRINITY_DN84021_c1_g1_i11	putative G3BP-like prote	2869	20
TRUE	[BLASTED, I	TRINITY_DN84037_c1_g1_i3	PX domain-containing pr	1748	20
TRUE	[BLASTED]	TRINITY_DN84070_c2_g1_i2	DNA-directed RNA polym	1800	10
TRUE	[NO-BLAST	TRINITY_DN79031_c0_g3_i1	---NA---	456	
TRUE	[BLASTED, I	TRINITY_DN79020_c1_g1_i10	probable N-acetylglucosa	1485	20
TRUE	[BLASTED, I	TRINITY_DN79004_c0_g3_i4	thaumatin-like protein	931	20
TRUE	[BLASTED, I	TRINITY_DN79004_c0_g3_i5	thaumatin-like protein	782	20
TRUE	[BLASTED, I	TRINITY_DN79046_c2_g1_i1	cell division cycle protein	1333	20
TRUE	[BLASTED, I	TRINITY_DN79061_c1_g1_i1	reticulon-like protein B2	923	20
TRUE	[NO-BLAST	TRINITY_DN79019_c1_g1_i3	---NA---	585	
TRUE	[BLASTED, I	TRINITY_DN79023_c4_g1_i14	probable protein phosph	2867	20
TRUE	[BLASTED, I	TRINITY_DN79023_c4_g1_i15	probable protein phosph	2927	20
TRUE	[BLASTED, I	TRINITY_DN79086_c0_g5_i1	ribosomal biogenesis pro	783	3
TRUE	[BLASTED]	TRINITY_DN79086_c0_g5_i4	ribosomal biogenesis pro	771	2
TRUE	[BLASTED, I	TRINITY_DN79093_c1_g1_i9	two-component respons	2981	20
TRUE	[BLASTED, I	TRINITY_DN79093_c1_g1_i12	two-component respons	2833	20
TRUE	[BLASTED, I	TRINITY_DN79037_c0_g3_i1	histone H4	409	20
TRUE	[BLASTED]	TRINITY_DN87940_c1_g5_i2	ACT domain-containing p	1365	20
TRUE	[BLASTED]	TRINITY_DN87940_c1_g1_i9	ACT domain-containing p	3158	20
TRUE	[BLASTED]	TRINITY_DN87981_c1_g7_i1	MATH domain-containing	1229	20
TRUE	[BLASTED]	TRINITY_DN87970_c1_g3_i3	Protein phosphatase 1 re	862	20
TRUE	[BLASTED, I	TRINITY_DN87963_c2_g1_i1	cullin-1 isoform X1	1024	20
TRUE	[BLASTED]	TRINITY_DN87947_c2_g3_i1	decapping 5-like protein	2231	20
TRUE	[BLASTED]	TRINITY_DN87967_c2_g2_i6	granule-bound starch syn	942	20
TRUE	[BLASTED, I	TRINITY_DN87965_c1_g3_i7	integrator complex subu	2426	20
TRUE	[BLASTED, I	TRINITY_DN87969_c1_g3_i8	Mitotic apparatus protei	2756	20
TRUE	[BLASTED, I	TRINITY_DN87951_c0_g1_i24	transcription initiation fa	1726	20
TRUE	[BLASTED, I	TRINITY_DN87949_c2_g3_i1	multicopper oxidase LPR	2547	20
TRUE	[BLASTED, I	TRINITY_DN87949_c2_g3_i4	multicopper oxidase LPR	2701	20
TRUE	[BLASTED]	TRINITY_DN87907_c1_g1_i12	ultraviolet-B receptor UV	2000	20
TRUE	[BLASTED]	TRINITY_DN87907_c1_g1_i39	ultraviolet-B receptor UV	1904	20
TRUE	[BLASTED]	TRINITY_DN87907_c1_g1_i57	ultraviolet-B receptor UV	2003	20
TRUE	[BLASTED, I	TRINITY_DN87990_c0_g1_i12	putative chloride channe	2422	20
TRUE	[BLASTED, I	TRINITY_DN87990_c0_g1_i20	putative chloride channe	3081	20
TRUE	[BLASTED, I	TRINITY_DN87901_c1_g1_i10	heat shock protein 83	3232	20
TRUE	[BLASTED, I	TRINITY_DN87978_c0_g2_i14	vam6/Vps39-like protein	1892	20
TRUE	[BLASTED, I	TRINITY_DN87952_c0_g1_i2	acid vacuolar invertase	2591	20
TRUE	[BLASTED, I	TRINITY_DN87952_c0_g2_i14	acid beta-fructofuranosic	1674	20
TRUE	[BLASTED, I	TRINITY_DN87937_c2_g2_i2	lysine-specific demethyla	2746	20
TRUE	[BLASTED, I	TRINITY_DN87950_c0_g1_i2	muscle M-line assembly	1558	20
TRUE	[BLASTED, I	TRINITY_DN87985_c1_g2_i5	histidine-containing phos	3783	20
TRUE	[BLASTED, I	TRINITY_DN82197_c2_g1_i3	protein SMG7L	3305	20
TRUE	[BLASTED, I	TRINITY_DN82179_c0_g2_i1	flocculation protein FLO1	3163	20
TRUE	[BLASTED]	TRINITY_DN82136_c2_g1_i17	RAB6A-GEF complex part	3556	20

TRUE	[BLASTED, I	TRINITY_DN82188_c5_g1_i1	F-box protein At1g67340	1578	20
TRUE	[BLASTED, I	TRINITY_DN82174_c3_g1_i3	ubiquitin-conjugating en:	949	20
TRUE	[BLASTED, I	TRINITY_DN82161_c0_g3_i6	heavy metal-associated i	1298	20
TRUE	[BLASTED, I	TRINITY_DN82101_c3_g1_i3	probable leucine-rich rep	875	20
TRUE	[BLASTED, I	TRINITY_DN82158_c0_g2_i3	30S ribosomal protein S2	817	20
TRUE	[NO-BLAST	TRINITY_DN82195_c0_g1_i2	---NA---	1784	
TRUE	[BLASTED, I	TRINITY_DN82177_c1_g4_i1	stromal 70 kDa heat shoc	1162	20
TRUE	[BLASTED, I	TRINITY_DN82177_c1_g4_i2	stromal 70 kDa heat shoc	1281	20
TRUE	[BLASTED, I	TRINITY_DN83558_c1_g1_i1	Glycosyltransferase fami	1748	20
TRUE	[BLASTED, I	TRINITY_DN83558_c1_g1_i2	protein O-linked-mannos	1533	20
TRUE	[BLASTED]	TRINITY_DN83567_c3_g1_i22	Phosphoglucosamine mu	2068	20
TRUE	[BLASTED, I	TRINITY_DN83555_c0_g2_i16	1-phosphatidylinositol-3-	2258	20
TRUE	[BLASTED, I	TRINITY_DN83555_c0_g1_i5	1-phosphatidylinositol-3-	1270	20
TRUE	[BLASTED]	TRINITY_DN83556_c2_g7_i1	hsp70-Hsp90 organizing	503	20
TRUE	[BLASTED]	TRINITY_DN83556_c2_g1_i7	hsp70-Hsp90 organizing	844	20
TRUE	[BLASTED]	TRINITY_DN83526_c1_g1_i2	ER membrane protein co	2008	20
TRUE	[BLASTED, I	TRINITY_DN83510_c0_g2_i1	zinc finger protein var3, (	2069	20
TRUE	[BLASTED, I	TRINITY_DN83510_c0_g2_i4	zinc finger protein VAR3,	1265	20
TRUE	[BLASTED, I	TRINITY_DN83589_c0_g1_i11	mitochondrial thiamine p	1777	20
TRUE	[BLASTED]	TRINITY_DN83579_c2_g5_i5	protein MAIN-LIKE 2-like	547	20
TRUE	[BLASTED, I	TRINITY_DN83505_c0_g1_i1	protein CHAPERONE-LIKE	1125	20
TRUE	[BLASTED, I	TRINITY_DN83571_c0_g2_i3	BURP domain-containing	1146	20
TRUE	[BLASTED, I	TRINITY_DN83584_c1_g1_i9	DEAD-box ATP-depender	1887	20
TRUE	[BLASTED, I	TRINITY_DN83541_c0_g1_i6	expansin-A8	1246	20
TRUE	[BLASTED, I	TRINITY_DN83520_c3_g5_i3	pentatricopeptide repeat	1690	20
TRUE	[BLASTED, I	TRINITY_DN83552_c0_g1_i1	cinnamoyl-CoA reductase	631	20
TRUE	[BLASTED, I	TRINITY_DN83552_c0_g3_i1	cinnamoyl-CoA reductase	535	20
TRUE	[BLASTED]	TRINITY_DN86948_c1_g4_i1	Serine/threonine-proteir	2148	20
TRUE	[BLASTED, I	TRINITY_DN86981_c0_g1_i1	glucan endo-1,3-beta-glu	2063	20
TRUE	[BLASTED]	TRINITY_DN86949_c0_g1_i5	nucleolar and coiled-bod	2393	20
TRUE	[BLASTED, I	TRINITY_DN86993_c1_g6_i1	BAG family molecular cha	1473	20
TRUE	[BLASTED, I	TRINITY_DN86993_c1_g5_i2	ferredoxin	1567	20
TRUE	[BLASTED, I	TRINITY_DN86993_c1_g12_i1	ferredoxin	1041	20
TRUE	[BLASTED]	TRINITY_DN86933_c1_g2_i3	LRR receptor-like serine/	941	20
TRUE	[BLASTED, I	TRINITY_DN86957_c0_g1_i16	DNA ligase 1 isoform X6	3159	20
TRUE	[BLASTED, I	TRINITY_DN86938_c3_g1_i20	autophagy-related prote	6132	20
TRUE	[BLASTED, I	TRINITY_DN86952_c1_g1_i7	ARM REPEAT PROTEIN IN	829	20
TRUE	[BLASTED, I	TRINITY_DN86964_c3_g2_i9	sulfoquinovosyl transfera	793	20
TRUE	[BLASTED, I	TRINITY_DN86918_c1_g2_i9	probable acyl-CoA dehyd	3044	20
TRUE	[BLASTED, I	TRINITY_DN86918_c1_g2_i14	probable acyl-CoA dehyd	3636	20
TRUE	[NO-BLAST	TRINITY_DN86961_c2_g7_i2	---NA---	745	
TRUE	[BLASTED, I	TRINITY_DN86910_c0_g2_i1	putative clathrin assemb	2789	20
TRUE	[BLASTED, I	TRINITY_DN86910_c0_g2_i24	putative clathrin assemb	2113	20
TRUE	[BLASTED, I	TRINITY_DN86903_c0_g2_i5	putative At5g37260	2405	20
TRUE	[BLASTED, I	TRINITY_DN86903_c0_g2_i9	At5g37260-like protein	2164	20
TRUE	[BLASTED, I	TRINITY_DN86989_c0_g1_i7	MAP protein	1930	20
TRUE	[BLASTED, I	TRINITY_DN86972_c0_g2_i15	casein kinase I-like	2108	20
TRUE	[BLASTED, I	TRINITY_DN86972_c0_g2_i17	Casein kinase 1-like prote	1388	20
TRUE	[BLASTED]	TRINITY_DN86987_c0_g1_i1	F-box protein At3g07870	1510	20
TRUE	[NO-BLAST	TRINITY_DN76877_c0_g1_i5	---NA---	1139	

TRUE	[BLASTED]	TRINITY_DN76869_c2_g1_i13	uncharacterized protein	1127	20
TRUE	[BLASTED, I	TRINITY_DN76827_c2_g2_i3	chloroplast photosystem	1127	20
TRUE	[BLASTED, I	TRINITY_DN76893_c0_g1_i1	ferruginol synthase-like	1114	20
TRUE	[BLASTED]	TRINITY_DN76808_c0_g3_i2	Formimidoyltetrahydrofo	1930	20
TRUE	[BLASTED]	TRINITY_DN32426_c0_g1_i1	ctenidin-1-like	666	1
TRUE	[BLASTED, I	TRINITY_DN85230_c1_g1_i2	probable protein phosph	3118	20
TRUE	[BLASTED, I	TRINITY_DN85220_c3_g1_i5	nucleolar complex protei	1222	20
TRUE	[BLASTED, I	TRINITY_DN85206_c0_g1_i6	putative alpha/beta hydr	517	20
TRUE	[BLASTED, I	TRINITY_DN85253_c2_g2_i1	nitrate reductase	1629	20
TRUE	[BLASTED, I	TRINITY_DN85239_c3_g4_i1	uncharacterized protein	996	20
TRUE	[BLASTED, I	TRINITY_DN85244_c1_g2_i8	mitochondrial substrate	2690	20
TRUE	[BLASTED]	TRINITY_DN85274_c2_g4_i1	protein RETICULATA-REL	1192	20
TRUE	[BLASTED]	TRINITY_DN85274_c2_g4_i9	protein RETICULATA-REL	2244	20
TRUE	[BLASTED]	TRINITY_DN85224_c1_g1_i8	DUF239 domain-containi	2612	20
TRUE	[BLASTED, I	TRINITY_DN85272_c0_g3_i5	FIP1[V]-like protein	3058	20
TRUE	[BLASTED, I	TRINITY_DN85225_c1_g1_i2	type 2 light-harvesting ch	1198	20
TRUE	[BLASTED, I	TRINITY_DN85268_c3_g2_i1	probable galactinol--sucr	2830	20
TRUE	[BLASTED, I	TRINITY_DN85218_c0_g1_i8	Nucleic acid-binding, OB-	2995	20
TRUE	[BLASTED, I	TRINITY_DN85256_c0_g1_i6	threonylcarbamoyladeno	2012	20
TRUE	[BLASTED]	TRINITY_DN85299_c1_g1_i10	uncharacterized protein	1443	20
TRUE	[BLASTED, I	TRINITY_DN85205_c4_g2_i17	ferredoxin--NADP reduct	1303	20
TRUE	[BLASTED, I	TRINITY_DN85294_c1_g1_i2	fructose-1,6-bisphosphat	2108	20
TRUE	[BLASTED, I	TRINITY_DN85294_c1_g2_i1	fructose-1,6-bisphosphat	2043	20
TRUE	[BLASTED, I	TRINITY_DN85294_c1_g1_i20	fructose-1,6-bisphosphat	2239	20
TRUE	[BLASTED, I	TRINITY_DN85222_c0_g1_i2	protein strawberry notch	2226	20
TRUE	[BLASTED, I	TRINITY_DN85228_c1_g2_i5	photosystem II core com	2235	20
TRUE	[BLASTED, I	TRINITY_DN85283_c2_g3_i4	putative LOV domain-cor	416	20
TRUE	[BLASTED]	TRINITY_DN85223_c0_g1_i11	uncharacterized protein	2609	20
TRUE	[BLASTED]	TRINITY_DN85262_c4_g2_i11	salicylic acid-binding prot	732	20
TRUE	[BLASTED]	TRINITY_DN85288_c1_g5_i1	stem-specific protein TSJ	605	20
TRUE	[BLASTED]	TRINITY_DN85288_c1_g5_i2	stem-specific protein TSJ	690	20
TRUE	[BLASTED]	TRINITY_DN85288_c1_g3_i9	stem-specific protein TSJ	641	20
TRUE	[BLASTED, I	TRINITY_DN85217_c1_g1_i6	Tyrosine kinase	2508	20
TRUE	[BLASTED]	TRINITY_DN80704_c0_g1_i2	Protein FAM214A like	2942	20
TRUE	[BLASTED]	TRINITY_DN80704_c0_g1_i5	Protein FAM214A like	3483	20
TRUE	[BLASTED, I	TRINITY_DN80713_c0_g2_i1	serine/arginine-rich splic	1492	20
TRUE	[BLASTED, I	TRINITY_DN80713_c0_g3_i8	serine/arginine-rich splic	2046	20
TRUE	[NO-BLAST	TRINITY_DN80713_c0_g6_i2	---NA---	3739	
TRUE	[BLASTED, I	TRINITY_DN80737_c1_g1_i3	putative ion channel POL	2695	20
TRUE	[BLASTED, I	TRINITY_DN80750_c1_g6_i2	UDP-glycosyltransferase	1912	20
TRUE	[BLASTED, I	TRINITY_DN80727_c3_g3_i3	myb-related protein 306-	1672	20
TRUE	[BLASTED, I	TRINITY_DN80708_c2_g1_i16	protein arv1 homolog	1189	20
TRUE	[BLASTED, I	TRINITY_DN80711_c0_g1_i5	uncharacterized protein	2342	20
TRUE	[BLASTED]	TRINITY_DN80729_c0_g2_i3	uncharacterized WD repe	1882	20
TRUE	[BLASTED, I	TRINITY_DN80757_c2_g2_i13	putative 3,4-dihydroxy-2	3427	20
TRUE	[BLASTED, I	TRINITY_DN86227_c0_g2_i2	ribonuclease II, chloropla	2340	20
TRUE	[BLASTED, I	TRINITY_DN86227_c0_g2_i4	ribonuclease II, chloropla	3513	20
TRUE	[BLASTED, I	TRINITY_DN86251_c2_g1_i1	photosystem II 22 kDa pr	1374	20
TRUE	[BLASTED, I	TRINITY_DN86251_c2_g4_i1	photosystem II 22 kDa pr	356	20
TRUE	[BLASTED, I	TRINITY_DN86279_c0_g1_i19	RNA-binding protein 39-l	2467	20

TRUE	[BLASTED, I	TRINITY_DN86260_c0_g1_i28	nucleobase-ascorbate tra	894	20
TRUE	[BLASTED, I	TRINITY_DN86243_c0_g1_i16	NAD kinase 2, chloroplas	3929	20
TRUE	[BLASTED, I	TRINITY_DN86294_c3_g1_i7	NAD/FAD-utilizing protei	2804	20
TRUE	[BLASTED, I	TRINITY_DN86296_c1_g1_i1	probable serine/threonir	1237	20
TRUE	[BLASTED, I	TRINITY_DN86278_c2_g1_i4	serine/threonine-protein	2127	20
TRUE	[BLASTED]	TRINITY_DN86259_c1_g5_i1	protein PLASTID MOVEM	2084	20
TRUE	[BLASTED]	TRINITY_DN86223_c1_g1_i6	neurofilament heavy poly	1457	20
TRUE	[BLASTED, I	TRINITY_DN86233_c5_g1_i9	serine--glyoxylate amino	1889	20
TRUE	[BLASTED, I	TRINITY_DN86233_c5_g1_i10	serine--glyoxylate amino	1513	20
TRUE	[BLASTED, I	TRINITY_DN86270_c1_g2_i8	peroxisomal membrane p	1650	20
TRUE	[BLASTED, I	TRINITY_DN86286_c0_g3_i3	lysine histidine transport	983	20
TRUE	[BLASTED, I	TRINITY_DN86215_c1_g1_i8	asparagine synthetase [g	2040	20
TRUE	[BLASTED, I	TRINITY_DN86215_c1_g1_i10	asparagine synthetase [g	1316	20
TRUE	[BLASTED, I	TRINITY_DN86215_c1_g1_i14	asparagine synthetase [g	1989	20
TRUE	[BLASTED, I	TRINITY_DN86244_c2_g1_i19	probable inositol transpo	3518	20
TRUE	[BLASTED]	TRINITY_DN85932_c0_g1_i7	ultraviolet-B receptor UV	1594	20
TRUE	[BLASTED, I	TRINITY_DN85949_c1_g1_i13	serine/arginine-rich splic	1454	20
TRUE	[BLASTED]	TRINITY_DN85908_c0_g1_i2	WD repeat-containing pr	4373	20
TRUE	[BLASTED]	TRINITY_DN85908_c0_g1_i10	WD repeat-containing pr	4377	20
TRUE	[BLASTED, I	TRINITY_DN85978_c0_g1_i1	transcriptional repressor	3370	20
TRUE	[BLASTED, I	TRINITY_DN85978_c0_g1_i2	transcriptional repressor	3382	20
TRUE	[BLASTED, I	TRINITY_DN85916_c0_g1_i7	probable methyltransfera	1910	20
TRUE	[BLASTED, I	TRINITY_DN85948_c0_g1_i1	S-adenosylmethionine ca	2687	20
TRUE	[BLASTED, I	TRINITY_DN85975_c2_g3_i13	GPI inositol-deacylase A i	5010	20
TRUE	[BLASTED, I	TRINITY_DN85976_c0_g2_i2	F-box/kelch-repeat prote	2995	20
TRUE	[BLASTED]	TRINITY_DN85947_c2_g1_i7	Threonine protease PRSS	3553	20
TRUE	[BLASTED, I	TRINITY_DN85937_c3_g1_i10	uncharacterized transpor	1942	20
TRUE	[BLASTED, I	TRINITY_DN85901_c0_g1_i18	protein SAWADEE HOME	418	20
TRUE	[BLASTED, I	TRINITY_DN85990_c1_g1_i9	branched-chain-amino-a	1762	20
TRUE	[BLASTED, I	TRINITY_DN85931_c1_g1_i1	serine/threonine-protein	2898	20
TRUE	[BLASTED, I	TRINITY_DN85921_c0_g1_i8	protein REDUCED WALL A	2850	20
TRUE	[BLASTED]	TRINITY_DN85962_c2_g1_i9	probable glucan 1,3-beta	3048	20
TRUE	[BLASTED]	TRINITY_DN82283_c0_g1_i16	SMC5-SMC6 complex loc	1848	20
TRUE	[BLASTED, I	TRINITY_DN82207_c3_g2_i7	1-aminocyclopropane-1-	974	20
TRUE	[BLASTED, I	TRINITY_DN82252_c0_g2_i9	probable methyltransfera	2302	20
TRUE	[BLASTED, I	TRINITY_DN82244_c0_g1_i3	adenine nucleotide trans	2139	20
TRUE	[BLASTED, I	TRINITY_DN82269_c0_g2_i9	nuclear pore complex pr	1720	20
TRUE	[BLASTED, I	TRINITY_DN82269_c0_g2_i15	nuclear pore complex pr	1700	20
TRUE	[BLASTED, I	TRINITY_DN82230_c4_g1_i9	phosphatidylcholine tran	1129	20
TRUE	[BLASTED, I	TRINITY_DN82230_c4_g1_i11	phosphatidylcholine tran	658	20
TRUE	[BLASTED, I	TRINITY_DN82261_c2_g2_i2	transcription initiation fa	2303	20
TRUE	[BLASTED, I	TRINITY_DN82261_c3_g1_i8	eukaryotic translation ini	1658	20
TRUE	[BLASTED, I	TRINITY_DN82265_c1_g1_i3	uncharacterized protein	531	20
TRUE	[BLASTED, I	TRINITY_DN82226_c0_g1_i7	DNA repair protein RAD5	2411	20
TRUE	[BLASTED, I	TRINITY_DN82277_c0_g1_i11	3-hydroxyisobutyryl-CoA	1613	20
TRUE	[BLASTED, I	TRINITY_DN82220_c4_g2_i5	protein CHLOROPLAST IN	2741	20
TRUE	[BLASTED, I	TRINITY_DN82258_c0_g6_i1	S-adenosylmethionine sy	521	20
TRUE	[BLASTED, I	TRINITY_DN75744_c0_g1_i1	pathogenesis-related pro	652	20
TRUE	[BLASTED, I	TRINITY_DN76436_c0_g1_i3	methyl-CpG-binding dom	2943	20
TRUE	[BLASTED, I	TRINITY_DN76475_c0_g1_i18	SNARE associated Golgi p	1354	20

TRUE	[BLASTED,	TRINITY_DN76413_c0_g1_i3	acidic mammalian chitina	1551	20
TRUE	[NO-BLAST	TRINITY_DN76464_c2_g2_i5	---NA---	2063	
TRUE	[NO-BLAST	TRINITY_DN76464_c2_g2_i6	---NA---	976	
TRUE	[BLASTED]	TRINITY_DN83458_c0_g1_i3	Tuberin like	1545	20
TRUE	[BLASTED]	TRINITY_DN83458_c0_g1_i12	Tuberin like	1580	20
TRUE	[BLASTED,	TRINITY_DN83476_c0_g4_i5	probable quinone oxidor	1943	20
TRUE	[BLASTED,	TRINITY_DN83429_c1_g1_i17	UDP-galactose/UDP-gluc	1793	20
TRUE	[BLASTED]	TRINITY_DN83499_c1_g1_i6	uncharacterized protein	5101	20
TRUE	[BLASTED,	TRINITY_DN83449_c1_g1_i1	type IV inositol polyphos	2243	20
TRUE	[BLASTED,	TRINITY_DN83431_c1_g1_i2	WAS/WASL-interacting p	1766	20
TRUE	[BLASTED]	TRINITY_DN83486_c0_g1_i15	3-phosphoshikimate 1-ca	2694	20
TRUE	[BLASTED,	TRINITY_DN83488_c2_g6_i1	ABC transporter B family	1468	20
TRUE	[BLASTED,	TRINITY_DN83473_c2_g2_i8	Ribonuclease H-like dom	2992	16
TRUE	[BLASTED,	TRINITY_DN79990_c2_g3_i9	rop guanine nucleotide e	2482	20
TRUE	[BLASTED]	TRINITY_DN79901_c1_g1_i10	protein LNK1-like isoforn	621	20
TRUE	[BLASTED,	TRINITY_DN79927_c0_g2_i8	uncharacterized oxidore	2117	20
TRUE	[BLASTED]	TRINITY_DN79959_c0_g1_i2	heavy metal-associated i	1157	1
TRUE	[BLASTED,	TRINITY_DN79966_c1_g7_i2	ATP-dependent helicase/	708	20
TRUE	[BLASTED,	TRINITY_DN79965_c0_g2_i10	cold-responsive protein k	3996	20
TRUE	[BLASTED,	TRINITY_DN79989_c0_g4_i13	nicotinate-nucleotide pyı	1620	20
TRUE	[NO-BLAST	TRINITY_DN79911_c3_g2_i1	---NA---	1850	
TRUE	[BLASTED,	TRINITY_DN79952_c1_g3_i1	cytochrome P450 78A3-l	1674	20
TRUE	[BLASTED,	TRINITY_DN79953_c1_g1_i7	aminodeoxychorismate s	2176	20
TRUE	[BLASTED,	TRINITY_DN79941_c1_g1_i3	structure-specific endonı	1539	20
TRUE	[BLASTED,	TRINITY_DN79900_c2_g1_i1	polyadenylate-binding pı	4110	20
TRUE	[BLASTED]	TRINITY_DN79991_c0_g4_i2	plant intracellular Ras-gı	2201	20
TRUE	[BLASTED]	TRINITY_DN77185_c0_g1_i5	protein ROOT PRIMORDI	3009	20
TRUE	[BLASTED,	TRINITY_DN77155_c2_g2_i4	histone-lysine N-methylt	1605	20
TRUE	[NO-BLAST	TRINITY_DN77181_c0_g3_i1	---NA---	2180	
TRUE	[BLASTED,	TRINITY_DN77150_c0_g1_i3	uncharacterized protein	2554	20
TRUE	[BLASTED]	TRINITY_DN84503_c1_g1_i13	protein NPGR2-like	3304	20
TRUE	[BLASTED,	TRINITY_DN84571_c0_g2_i2	thioredoxin H-type	914	20
TRUE	[BLASTED,	TRINITY_DN84546_c1_g1_i9	probable purine permeas	3271	20
TRUE	[BLASTED,	TRINITY_DN84519_c0_g2_i9	phosphatidylinositol 4-pł	2414	20
TRUE	[BLASTED,	TRINITY_DN84511_c3_g1_i4	plasma membrane-assoc	1228	20
TRUE	[BLASTED,	TRINITY_DN84529_c1_g1_i5	shaggy-related protein ki	1492	20
TRUE	[BLASTED,	TRINITY_DN84501_c3_g1_i4	transmembrane protein	1947	20
TRUE	[BLASTED,	TRINITY_DN84509_c0_g1_i2	protein SHOOT GRAVITR	1332	20
TRUE	[BLASTED,	TRINITY_DN81391_c0_g3_i6	homeobox-leucine zippe	1743	20
TRUE	[BLASTED]	TRINITY_DN81378_c3_g1_i18	neurochondrin isoform X	1920	20
TRUE	[BLASTED]	TRINITY_DN81346_c1_g6_i1	Large delta antigen like	875	20
TRUE	[BLASTED,	TRINITY_DN81372_c1_g2_i1	glycine-rich RNA-binding	1114	20
TRUE	[BLASTED,	TRINITY_DN81367_c0_g2_i21	translation initiation fact	1349	20
TRUE	[BLASTED]	TRINITY_DN81357_c1_g3_i11	Protein ApaG like	1468	20
TRUE	[BLASTED,	TRINITY_DN81359_c0_g1_i12	ADP-ribosylation factor-l	1576	20
TRUE	[BLASTED,	TRINITY_DN81384_c1_g1_i8	neuroguidin	1551	20
TRUE	[BLASTED,	TRINITY_DN81341_c1_g1_i6	thioredoxin-like 3-1, chlo	782	20
TRUE	[BLASTED]	TRINITY_DN81306_c2_g1_i2	protein FAR1-RELATED SI	1241	20
TRUE	[BLASTED]	TRINITY_DN81395_c0_g1_i1	DDB1- and CUL4-associat	1611	20
TRUE	[BLASTED,	TRINITY_DN81337_c0_g4_i5	ras guanine nucleotide e:	3046	20

TRUE	[BLASTED, I	TRINITY_DN81330_c0_g1_i8	integrin-linked protein ki	1951	20
TRUE	[BLASTED, I	TRINITY_DN81317_c1_g1_i1	probable sulfate transpo	2498	20
TRUE	[BLASTED]	TRINITY_DN81332_c1_g4_i2	uncharacterized protein	448	5
TRUE	[BLASTED]	TRINITY_DN82820_c0_g1_i15	uncharacterized protein	2073	20
TRUE	[BLASTED, I	TRINITY_DN82872_c3_g1_i2	callose synthase 3	2072	20
TRUE	[BLASTED, I	TRINITY_DN82874_c0_g1_i11	DNA polymerase delta sr	2520	20
TRUE	[BLASTED, I	TRINITY_DN82865_c0_g1_i4	tyrosyl-DNA phosphodie:	2500	20
TRUE	[BLASTED, I	TRINITY_DN82842_c2_g1_i8	ylmG homolog protein 1-	2894	20
TRUE	[BLASTED, I	TRINITY_DN82810_c0_g2_i2	aminopeptidase M1	1868	20
TRUE	[BLASTED, I	TRINITY_DN82873_c1_g2_i17	vacuolar protein sorting-	1586	20
TRUE	[BLASTED]	TRINITY_DN82811_c0_g2_i1	uncharacterized protein	6587	20
TRUE	[BLASTED, I	TRINITY_DN82890_c0_g3_i3	heat stress transcription	1022	20
TRUE	[NO-BLAST	TRINITY_DN84308_c2_g4_i3	---NA---	2068	
TRUE	[BLASTED, I	TRINITY_DN84317_c2_g1_i9	FACT complex subunit SF	4052	20
TRUE	[BLASTED, I	TRINITY_DN84317_c2_g1_i10	FACT complex subunit SF	4050	20
TRUE	[BLASTED, I	TRINITY_DN84307_c1_g1_i9	ubiquitin receptor RAD2:	1134	20
TRUE	[BLASTED, I	TRINITY_DN84343_c0_g1_i7	probable acyl-activating	2440	20
TRUE	[BLASTED]	TRINITY_DN84303_c2_g1_i9	ultraviolet-B receptor UV	1419	20
TRUE	[BLASTED, I	TRINITY_DN84322_c1_g5_i1	uncharacterized protein	1064	20
TRUE	[BLASTED, I	TRINITY_DN84300_c1_g1_i4	homeobox-DDT domain	2916	20
TRUE	[BLASTED, I	TRINITY_DN84379_c1_g6_i1	non-specific lipid-transfe	269	20
TRUE	[BLASTED]	TRINITY_DN84345_c1_g2_i5	F-box protein At2g02240	843	20
TRUE	[BLASTED, I	TRINITY_DN84452_c0_g1_i13	serine/threonine-protein	1468	20
TRUE	[BLASTED]	TRINITY_DN84431_c1_g8_i1	salicylic acid-binding prot	414	3
TRUE	[BLASTED]	TRINITY_DN84474_c2_g3_i5	protein TSS	2375	20
TRUE	[BLASTED, I	TRINITY_DN84464_c3_g1_i3	N-carbamoylputrescine a	1377	20
TRUE	[BLASTED, I	TRINITY_DN84464_c3_g1_i12	N-carbamoylputrescine a	1156	20
TRUE	[BLASTED, I	TRINITY_DN84455_c4_g1_i3	Binding protein, putative	1774	20
TRUE	[BLASTED, I	TRINITY_DN84425_c0_g1_i7	probable GABA transport	1799	20
TRUE	[BLASTED, I	TRINITY_DN84424_c2_g1_i1	LRR receptor-like serine/	3408	20
TRUE	[BLASTED, I	TRINITY_DN84420_c0_g1_i1	anthocyanidin 3-O-gluco:	1615	20
TRUE	[BLASTED, I	TRINITY_DN84461_c3_g2_i18	FAD synthase isoform X1	1994	20
TRUE	[BLASTED, I	TRINITY_DN84448_c2_g1_i3	protein ECERIFERUM 1-li	2605	20
TRUE	[BLASTED, I	TRINITY_DN84434_c0_g4_i2	metallothionein-like prot	278	20
TRUE	[BLASTED, I	TRINITY_DN84479_c2_g3_i2	amino acid permease 6-l	1887	20
TRUE	[BLASTED, I	TRINITY_DN84479_c2_g3_i12	amino acid permease 6	1849	20
TRUE	[BLASTED, I	TRINITY_DN84499_c0_g1_i7	beta-glucuronosyltransfe	1228	20
TRUE	[BLASTED, I	TRINITY_DN84407_c3_g1_i1	high affinity nitrate trans	220	20
TRUE	[BLASTED, I	TRINITY_DN84410_c0_g2_i1	E3 ubiquitin-protein ligas	1103	20
TRUE	[BLASTED, I	TRINITY_DN82018_c0_g2_i3	B3 domain-containing pr	2543	20
TRUE	[BLASTED, I	TRINITY_DN82083_c1_g3_i14	probable leucine-rich rep	2626	20
TRUE	[BLASTED]	TRINITY_DN82087_c1_g4_i2	probable receptor-like pr	407	1
TRUE	[BLASTED]	TRINITY_DN82051_c1_g2_i11	stress response protein N	1966	20
TRUE	[BLASTED, I	TRINITY_DN82098_c1_g2_i7	transcriptional activator	2915	20
TRUE	[BLASTED, I	TRINITY_DN82022_c1_g1_i21	uncharacterized protein	2336	20
TRUE	[BLASTED, I	TRINITY_DN82035_c0_g2_i2	galactinol synthase 2	954	20
TRUE	[BLASTED, I	TRINITY_DN82024_c2_g1_i14	pyruvate kinase, cytosoli	2118	20
TRUE	[BLASTED, I	TRINITY_DN82042_c5_g1_i12	haloacid dehalogenase-li	1373	20
TRUE	[BLASTED]	TRINITY_DN82001_c1_g3_i5	uncharacterized protein	1871	20
TRUE	[BLASTED, I	TRINITY_DN82071_c6_g2_i35	aspartate aminotransfera	2572	20

TRUE	[BLASTED, I	TRINITY_DN82059_c0_g1_i4	growth-regulating factor	1955	20
TRUE	[BLASTED, I	TRINITY_DN82096_c1_g2_i10	nodulin homeobox isofor	3384	20
TRUE	[BLASTED, I	TRINITY_DN82096_c1_g2_i22	nodulin homeobox isofor	3814	20
TRUE	[BLASTED]	TRINITY_DN86517_c0_g1_i13	PREDICTED: uncharacteri	2636	20
TRUE	[NO-BLAST	TRINITY_DN86530_c0_g2_i2	---NA---	1056	
TRUE	[NO-BLAST	TRINITY_DN86530_c0_g2_i4	---NA---	1083	
TRUE	[BLASTED, I	TRINITY_DN86592_c0_g3_i3	heat shock cognate 70 kI	983	20
TRUE	[BLASTED, I	TRINITY_DN86518_c0_g1_i7	Zinc finger, CCHC-type do	6336	20
TRUE	[BLASTED, I	TRINITY_DN86518_c0_g1_i8	Zinc finger, CCHC-type do	3814	20
TRUE	[BLASTED, I	TRINITY_DN86518_c0_g1_i11	Zinc finger, CCHC-type do	3802	20
TRUE	[BLASTED]	TRINITY_DN86509_c1_g1_i6	Bromodomain-containing	2443	20
TRUE	[BLASTED, I	TRINITY_DN86564_c1_g1_i10	auxin response factor 19	4404	20
TRUE	[BLASTED, I	TRINITY_DN86515_c0_g2_i9	ubiquitin carboxyl-termir	2717	20
TRUE	[BLASTED, I	TRINITY_DN86515_c0_g2_i14	ubiquitin carboxyl-termir	1703	20
TRUE	[BLASTED]	TRINITY_DN86507_c1_g1_i4	probable starch synthase	756	20
TRUE	[BLASTED]	TRINITY_DN86507_c1_g1_i14	probable starch synthase	5209	20
TRUE	[BLASTED, I	TRINITY_DN86507_c1_g1_i18	probable starch synthase	2746	20
TRUE	[BLASTED]	TRINITY_DN86507_c1_g1_i29	probable starch synthase	5572	20
TRUE	[BLASTED]	TRINITY_DN86507_c1_g1_i32	probable starch synthase	5195	20
TRUE	[NO-BLAST	TRINITY_DN86586_c1_g1_i2	---NA---	1752	
TRUE	[BLASTED, I	TRINITY_DN86597_c0_g1_i14	probable serine/threonir	1887	20
TRUE	[BLASTED, I	TRINITY_DN86545_c0_g1_i11	dnaJ protein P58IPK hom	2052	20
TRUE	[BLASTED]	TRINITY_DN86579_c1_g3_i1	uncharacterized protein	1045	20
TRUE	[BLASTED, I	TRINITY_DN86594_c0_g1_i5	TPR repeat-containing th	1102	20
TRUE	[BLASTED, I	TRINITY_DN86573_c2_g1_i3	mitochondrial uncoupling	1801	20
TRUE	[BLASTED, I	TRINITY_DN86511_c0_g1_i23	KH domain-containing pr	777	20
TRUE	[BLASTED, I	TRINITY_DN79214_c0_g2_i4	CBL-interacting protein k	2307	20
TRUE	[BLASTED, I	TRINITY_DN79281_c0_g3_i1	ubiquitin-conjugating en:	2680	20
TRUE	[BLASTED, I	TRINITY_DN79261_c1_g5_i4	eukaryotic translation ini	4350	20
TRUE	[BLASTED, I	TRINITY_DN79260_c1_g1_i11	cytochrome P450 90A1	1854	20
TRUE	[BLASTED, I	TRINITY_DN79223_c1_g2_i11	ABC transporter A family	6137	20
TRUE	[BLASTED, I	TRINITY_DN79223_c1_g3_i10	DNA-directed RNA polym	1392	20
TRUE	[BLASTED, I	TRINITY_DN79268_c3_g1_i7	formin-like protein 20	1708	20
TRUE	[BLASTED, I	TRINITY_DN79257_c0_g3_i3	splicing factor U2AF-assc	1856	20
TRUE	[BLASTED, I	TRINITY_DN79201_c0_g1_i7	protein trichome berefri	2319	20
TRUE	[BLASTED, I	TRINITY_DN79201_c0_g1_i8	protein trichome berefri	1875	20
TRUE	[BLASTED, I	TRINITY_DN79201_c0_g1_i9	protein trichome berefri	1976	20
TRUE	[BLASTED, I	TRINITY_DN79244_c2_g7_i1	cleavage and polyadenyl	4924	20
TRUE	[BLASTED, I	TRINITY_DN79244_c2_g7_i7	cleavage and polyadenyl	5053	20
TRUE	[BLASTED, I	TRINITY_DN81031_c0_g5_i4	hypothetical protein CDL	5548	20
TRUE	[BLASTED, I	TRINITY_DN81039_c1_g4_i2	COP1-interactive protein	5320	20
TRUE	[BLASTED, I	TRINITY_DN81022_c0_g1_i8	branched-chain-amino-a	936	20
TRUE	[BLASTED, I	TRINITY_DN81067_c1_g3_i6	calcineurin B-like protein	1809	20
TRUE	[BLASTED, I	TRINITY_DN81072_c1_g3_i5	probable aquaporin PIP2	1841	20
TRUE	[BLASTED, I	TRINITY_DN81078_c1_g2_i1	ATP-dependent zinc met	1163	20
TRUE	[BLASTED, I	TRINITY_DN81043_c1_g1_i12	carbon catabolite repres:	2737	20
TRUE	[BLASTED, I	TRINITY_DN81010_c0_g1_i1	chloride channel protein	840	20
TRUE	[BLASTED, I	TRINITY_DN81025_c2_g2_i5	YTH domain-containing f	3398	20
TRUE	[BLASTED]	TRINITY_DN80913_c1_g5_i2	beta-glucosidase-like	1537	20
TRUE	[BLASTED]	TRINITY_DN80982_c1_g3_i2	Signaling mucin like	3066	20

TRUE	[BLASTED]	TRINITY_DN80969_c0_g1_i5	UPF0301 protein SCO294	1153	20
TRUE	[BLASTED, I	TRINITY_DN80993_c0_g1_i3	heavy metal-associated i	1456	20
TRUE	[BLASTED]	TRINITY_DN80986_c1_g3_i1	putative WEB family prot	2063	20
TRUE	[NO-BLAST	TRINITY_DN80998_c2_g4_i1	---NA---	1363	
TRUE	[BLASTED, I	TRINITY_DN80989_c1_g2_i3	oxygen-evolving enhance	1010	20
TRUE	[BLASTED, I	TRINITY_DN80909_c0_g1_i3	thiosulfate sulfurtransfer	915	20
TRUE	[BLASTED, I	TRINITY_DN80910_c1_g1_i19	sister chromatid cohesio	2385	20
TRUE	[BLASTED, I	TRINITY_DN80983_c0_g1_i23	uncharacterized protein	2406	20
TRUE	[BLASTED, I	TRINITY_DN80911_c0_g2_i3	3-oxoacyl-[acyl-carrier-pı	1667	20
TRUE	[BLASTED, I	TRINITY_DN81285_c2_g2_i10	DExH-box ATP-depender	5042	20
TRUE	[BLASTED, I	TRINITY_DN81280_c0_g1_i13	putative B3 domain-cont	1287	20
TRUE	[BLASTED, I	TRINITY_DN81213_c3_g1_i1	nuclear transcription fact	1648	20
TRUE	[BLASTED, I	TRINITY_DN81213_c3_g1_i3	nuclear transcription fact	533	20
TRUE	[BLASTED, I	TRINITY_DN81213_c3_g2_i5	translation initiation fact	1383	20
TRUE	[BLASTED, I	TRINITY_DN81297_c0_g3_i11	protein DETOXIFICATION	2904	20
TRUE	[BLASTED]	TRINITY_DN81288_c4_g2_i8	probable aldo-keto redu	2439	20
TRUE	[BLASTED, I	TRINITY_DN81237_c2_g1_i3	transcription factor bHLH	1225	20
TRUE	[BLASTED]	TRINITY_DN81293_c0_g1_i11	probable plastid-lipid-ass	1645	20
TRUE	[BLASTED, I	TRINITY_DN81294_c0_g1_i25	3-hydroxyisobutyryl-CoA	2815	20
TRUE	[BLASTED, I	TRINITY_DN81210_c1_g1_i6	rac-like GTP-binding prot	1359	20
TRUE	[BLASTED, I	TRINITY_DN81222_c3_g5_i2	uncharacterized protein	1711	20
TRUE	[BLASTED, I	TRINITY_DN64854_c0_g1_i1	histone H2A.1	710	20
TRUE	[BLASTED, I	TRINITY_DN64854_c0_g1_i2	histone H2A.1-like	798	20
TRUE	[BLASTED, I	TRINITY_DN87266_c4_g1_i9	polypyrimidine tract-bin	2271	20
TRUE	[BLASTED]	TRINITY_DN87221_c2_g5_i1	uncharacterized protein	574	2
TRUE	[BLASTED, I	TRINITY_DN87261_c0_g1_i3	WPP domain-associated	3565	20
TRUE	[BLASTED, I	TRINITY_DN87261_c0_g1_i5	WPP domain-associated	3438	20
TRUE	[BLASTED, I	TRINITY_DN87261_c0_g1_i12	WPP domain-associated	3572	20
TRUE	[BLASTED, I	TRINITY_DN87261_c0_g1_i13	WPP domain-associated	3587	20
TRUE	[BLASTED, I	TRINITY_DN87242_c1_g1_i18	ubiquitin carboxyl-termir	3225	20
TRUE	[BLASTED, I	TRINITY_DN87247_c2_g1_i22	asparagine--tRNA ligase,	1043	20
TRUE	[BLASTED, I	TRINITY_DN87215_c1_g3_i4	dual specificity protein ki	1875	20
TRUE	[BLASTED, I	TRINITY_DN87256_c0_g2_i1	autophagy-related prote	1451	20
TRUE	[BLASTED]	TRINITY_DN87232_c0_g1_i4	4-coumarate--CoA ligase	2154	5
TRUE	[BLASTED, I	TRINITY_DN87253_c3_g2_i6	nuclear pore complex pr	1921	20
TRUE	[BLASTED]	TRINITY_DN87272_c0_g1_i2	protein LNK1-like isoforn	897	20
TRUE	[BLASTED]	TRINITY_DN87272_c0_g1_i3	protein LNK1-like isoforn	3051	20
TRUE	[BLASTED]	TRINITY_DN87272_c0_g1_i11	protein LNK1-like isoforn	1008	5
TRUE	[BLASTED]	TRINITY_DN87272_c0_g1_i12	protein LNK1-like isoforn	1166	5
TRUE	[BLASTED, I	TRINITY_DN87238_c0_g7_i2	protein E6-like	1325	17
TRUE	[BLASTED, I	TRINITY_DN87220_c1_g2_i3	heat shock 70 kDa protei	2841	20
TRUE	[BLASTED]	TRINITY_DN87241_c2_g1_i13	PHD finger protein rhino	1234	20
TRUE	[BLASTED, I	TRINITY_DN83051_c4_g1_i7	indole-3-glycerol phosph	2039	20
TRUE	[BLASTED]	TRINITY_DN83085_c0_g3_i4	COP1-interacting protein	1630	20
TRUE	[BLASTED, I	TRINITY_DN83033_c0_g2_i6	nuclear intron maturase	3808	20
TRUE	[BLASTED, I	TRINITY_DN83033_c0_g2_i23	pheophytinase, chloropl	1883	20
TRUE	[NO-BLAST	TRINITY_DN83079_c0_g3_i7	---NA---	2845	
TRUE	[BLASTED, I	TRINITY_DN83000_c0_g1_i7	probable polygalacturon	1340	20
TRUE	[BLASTED, I	TRINITY_DN83000_c1_g1_i8	zinc finger CCCH domain	1832	20
TRUE	[BLASTED, I	TRINITY_DN83082_c0_g1_i3	probable protein phosph	2835	20

TRUE	[BLASTED, I	TRINITY_DN83005_c1_g2_i10	splicing factor U2af small	1429	20
TRUE	[BLASTED, I	TRINITY_DN83009_c1_g1_i14	type I inositol polyphospl	2481	20
TRUE	[BLASTED, I	TRINITY_DN83065_c1_g2_i16	ubiquitin carboxyl-termir	2538	20
TRUE	[BLASTED, I	TRINITY_DN83039_c0_g2_i9	probable plastidic glucos	2263	20
TRUE	[BLASTED, I	TRINITY_DN83028_c0_g2_i7	O-fucosyltransferase 23-	2364	20
TRUE	[BLASTED]	TRINITY_DN83077_c4_g1_i2	protein FAR1-RELATED SI	1367	20
TRUE	[BLASTED]	TRINITY_DN83011_c0_g1_i1	transcriptional corepress	3340	20
TRUE	[BLASTED, I	TRINITY_DN83010_c0_g1_i5	D-amino-acid transamina	2095	20
TRUE	[BLASTED, I	TRINITY_DN83010_c0_g1_i10	(6-4)DNA photolyase	2865	20
TRUE	[BLASTED, I	TRINITY_DN82403_c1_g1_i4	aldehyde dehydrogenase	2218	20
TRUE	[BLASTED, I	TRINITY_DN82403_c1_g1_i5	aldehyde dehydrogenase	1283	20
TRUE	[BLASTED, I	TRINITY_DN82498_c1_g1_i3	DNA repair protein recA	1911	20
TRUE	[BLASTED, I	TRINITY_DN82410_c2_g2_i5	dol-P-Man:Man(6)GlcNA	1588	20
TRUE	[BLASTED, I	TRINITY_DN82401_c1_g1_i6	disease resistance protei	2568	20
TRUE	[BLASTED, I	TRINITY_DN82474_c0_g1_i1	nucleolar MIF4G domain	3053	20
TRUE	[BLASTED]	TRINITY_DN82406_c1_g2_i3	vegetative cell wall prote	1540	20
TRUE	[BLASTED, I	TRINITY_DN82444_c1_g1_i4	aquaporin PIP2-1-like	934	20
TRUE	[BLASTED, I	TRINITY_DN82421_c1_g3_i2	chalcone synthase	1354	20
TRUE	[BLASTED, I	TRINITY_DN82460_c3_g2_i10	DUF724 domain-containi	2034	20
TRUE	[BLASTED, I	TRINITY_DN82493_c0_g1_i6	butyrate--CoA ligase AAE	1985	20
TRUE	[BLASTED, I	TRINITY_DN82422_c0_g5_i4	mediator of RNA polyme	2556	20
TRUE	[BLASTED]	TRINITY_DN85482_c0_g1_i8	with TLDc domain	1551	20
TRUE	[BLASTED, I	TRINITY_DN85435_c0_g1_i15	NAD-dependent malic er	2647	20
TRUE	[BLASTED]	TRINITY_DN85411_c0_g3_i3	uncharacterized protein	3986	20
TRUE	[BLASTED]	TRINITY_DN85424_c1_g1_i2	WD repeat-containing pr	3362	20
TRUE	[BLASTED]	TRINITY_DN85492_c1_g3_i1	protein EXORDIUM-like 5	1579	20
TRUE	[BLASTED]	TRINITY_DN85432_c0_g1_i3	Transposon, En/Spm-like	1148	20
TRUE	[BLASTED, I	TRINITY_DN85432_c0_g1_i11	Transposon, En/Spm-like	1082	20
TRUE	[BLASTED, I	TRINITY_DN85473_c0_g4_i1	phosphatidylinositol 4-ki	5264	20
TRUE	[BLASTED, I	TRINITY_DN85473_c0_g4_i2	phosphatidylinositol 4-ki	4808	20
TRUE	[BLASTED, I	TRINITY_DN85429_c3_g2_i15	malate dehydrogenase [I	1314	20
TRUE	[BLASTED]	TRINITY_DN85470_c0_g2_i3	metacaspase-1-like isofo	1987	20
TRUE	[BLASTED, I	TRINITY_DN85407_c1_g5_i1	ferredoxin-2, mitochondı	513	20
TRUE	[BLASTED]	TRINITY_DN85422_c0_g1_i9	F-box protein At1g61340	1172	20
TRUE	[NO-BLAST	TRINITY_DN85491_c1_g1_i1	---NA---	1031	
TRUE	[BLASTED, I	TRINITY_DN85491_c2_g3_i9	alternative NAD(P)H-ubic	4221	20
TRUE	[BLASTED]	TRINITY_DN85449_c2_g1_i1	protein LNK1-like isoforn	2696	20
TRUE	[BLASTED, I	TRINITY_DN85480_c1_g1_i10	DExH-box ATP-dependen	3853	20
TRUE	[BLASTED, I	TRINITY_DN85494_c0_g2_i4	extra-large guanine nucle	2667	20
TRUE	[BLASTED, I	TRINITY_DN85417_c0_g1_i9	receptor-like serine/thre	4572	20
TRUE	[BLASTED, I	TRINITY_DN85405_c4_g1_i5	tonoplast dicarboxylate t	1796	20
TRUE	[BLASTED, I	TRINITY_DN73004_c0_g1_i1	probable histone H2B.3	651	20
TRUE	[BLASTED, I	TRINITY_DN78632_c4_g2_i16	guanine nucleotide-bindi	1214	18
TRUE	[BLASTED, I	TRINITY_DN78671_c4_g1_i7	acetyltransferase NSI	1367	20
TRUE	[BLASTED, I	TRINITY_DN78610_c0_g4_i1	beta-carotene isomerase	1097	20
TRUE	[BLASTED, I	TRINITY_DN78610_c0_g4_i2	beta-carotene isomerase	676	20
TRUE	[BLASTED, I	TRINITY_DN78647_c3_g3_i4	neutral/alkaline invertas	2688	20
TRUE	[BLASTED]	TRINITY_DN78616_c0_g1_i7	uncharacterized protein	2859	20
TRUE	[BLASTED, I	TRINITY_DN78604_c0_g1_i5	cullin-3A-like	2870	20
TRUE	[BLASTED, I	TRINITY_DN78604_c0_g1_i14	cullin-3A-like	2020	20

TRUE	[BLASTED, I	TRINITY_DN78688_c1_g2_i8	soluble inorganic pyroph	1715	20
TRUE	[BLASTED, I	TRINITY_DN78697_c1_g5_i1	photosynthetic NDH subu	1042	20
TRUE	[BLASTED, I	TRINITY_DN84224_c1_g2_i1	acyl-lipid (9-3)-desaturas	1568	20
TRUE	[BLASTED, I	TRINITY_DN84298_c0_g1_i6	heparanase-like protein :	2359	20
TRUE	[BLASTED, I	TRINITY_DN84298_c0_g1_i11	heparanase-like protein :	4145	20
TRUE	[BLASTED]	TRINITY_DN84280_c0_g3_i1	cysteine proteinase inhib	301	1
TRUE	[BLASTED]	TRINITY_DN84280_c0_g3_i3	cysteine proteinase inhib	596	1
TRUE	[BLASTED]	TRINITY_DN84280_c1_g3_i9	Serine-rich adhesin for pl	4428	20
TRUE	[BLASTED, I	TRINITY_DN84238_c1_g5_i10	lysophospholipid acyltrar	3141	20
TRUE	[BLASTED, I	TRINITY_DN84262_c2_g5_i2	cellulose synthase-like pr	2181	20
TRUE	[BLASTED, I	TRINITY_DN84218_c1_g1_i7	pyruvate, phosphate diki	1366	20
TRUE	[BLASTED, I	TRINITY_DN84218_c1_g1_i8	pyruvate, phosphate diki	1237	20
TRUE	[BLASTED, I	TRINITY_DN84245_c1_g1_i32	inactive protein kinase SI	3831	20
TRUE	[BLASTED, I	TRINITY_DN84242_c1_g2_i4	scarecrow-like protein 9	2766	20
TRUE	[BLASTED, I	TRINITY_DN84276_c3_g2_i2	Ty3/gypsy retrotranspos	3456	20
TRUE	[BLASTED, I	TRINITY_DN84279_c0_g3_i2	probable metal-nicotiana	2752	20
TRUE	[BLASTED, I	TRINITY_DN84267_c2_g1_i25	uncharacterized protein	2240	20
TRUE	[BLASTED, I	TRINITY_DN84241_c1_g2_i2	serine/threonine-protein	1583	20
TRUE	[BLASTED]	TRINITY_DN84272_c1_g1_i13	defective in cullin neddyl	3632	20
TRUE	[BLASTED]	TRINITY_DN84212_c0_g1_i4	uncharacterized WD repe	1849	20
TRUE	[BLASTED, I	TRINITY_DN84255_c3_g1_i3	aldehyde dehydrogenase	1788	20
TRUE	[BLASTED, I	TRINITY_DN84255_c3_g1_i17	aldehyde dehydrogenase	2571	20
TRUE	[BLASTED, I	TRINITY_DN79326_c0_g1_i2	thioredoxin H2-like	823	20
TRUE	[BLASTED, I	TRINITY_DN79301_c3_g7_i1	alkane hydroxylase MAH	1993	20
TRUE	[BLASTED, I	TRINITY_DN79304_c2_g1_i2	NAD(P)H-quinone oxidor	1174	20
TRUE	[BLASTED, I	TRINITY_DN79304_c2_g1_i6	NAD(P)H-quinone oxidor	1250	20
TRUE	[BLASTED, I	TRINITY_DN79318_c3_g1_i4	subtilisin-like protease SI	3887	20
TRUE	[BLASTED, I	TRINITY_DN79379_c0_g1_i4	protein LIKE COV 1-like	1411	20
TRUE	[BLASTED, I	TRINITY_DN79382_c0_g2_i1	glucan endo-1,3-beta-glu	1520	20
TRUE	[BLASTED]	TRINITY_DN79332_c1_g3_i10	ubiquitin domain-contair	2285	20
TRUE	[BLASTED, I	TRINITY_DN79320_c0_g11_i2	galactan beta-1,4-galactc	2909	20
TRUE	[BLASTED]	TRINITY_DN74021_c0_g1_i1	vegetative cell wall prote	1353	1
TRUE	[BLASTED, I	TRINITY_DN82599_c2_g2_i1	protein RETICULATA-REL	2171	20
TRUE	[BLASTED, I	TRINITY_DN82503_c2_g2_i3	homeobox-DDT domain		3496	20
TRUE	[BLASTED, I	TRINITY_DN82535_c0_g5_i4	probable 2-carboxy-D-ara	902	20
TRUE	[BLASTED, I	TRINITY_DN82507_c0_g2_i3	ruBisCO large subunit-bir	2181	20
TRUE	[BLASTED, I	TRINITY_DN82516_c0_g2_i5	ubiquitin-conjugating en:	2341	20
TRUE	[BLASTED, I	TRINITY_DN82573_c3_g1_i1	quinone oxidoreductase	1005	20
TRUE	[BLASTED, I	TRINITY_DN82501_c0_g2_i3	triosephosphate isomera	2545	20
TRUE	[BLASTED, I	TRINITY_DN82501_c0_g1_i4	triosephosphate isomera	1437	20
TRUE	[BLASTED, I	TRINITY_DN82541_c0_g1_i4	zinc finger protein JACKD	2307	20
TRUE	[BLASTED]	TRINITY_DN82502_c1_g1_i24	uncharacterized protein	2992	20
TRUE	[BLASTED, I	TRINITY_DN82588_c2_g2_i7	monodehydroascorbate	1665	20
TRUE	[BLASTED]	TRINITY_DN82588_c2_g5_i2	amino acid ABC transpor	366	20
TRUE	[BLASTED, I	TRINITY_DN82547_c0_g3_i1	glycerol-3-phosphate del	847	20
TRUE	[BLASTED, I	TRINITY_DN82571_c0_g1_i2	pathogenesis-related lea	896	20
TRUE	[BLASTED, I	TRINITY_DN82521_c2_g1_i7	protein DOWNY MILDEW	1926	20
TRUE	[BLASTED, I	TRINITY_DN82570_c0_g2_i4	photosystem I chlorophy	1393	20
TRUE	[BLASTED, I	TRINITY_DN82570_c0_g2_i17	photosystem I chlorophy	1202	20
TRUE	[BLASTED, I	TRINITY_DN76952_c0_g1_i1	transcription factor CPC-	557	20

TRUE	[BLASTED, I	TRINITY_DN76957_c0_g2_i6	zinc finger protein CONS	2334	20
TRUE	[BLASTED, I	TRINITY_DN76957_c0_g1_i6	zinc finger protein CONS	1386	20
TRUE	[BLASTED, I	TRINITY_DN76989_c1_g1_i3	regulator of nonsense tra	1930	20
TRUE	[BLASTED, I	TRINITY_DN76917_c0_g3_i2	15 kDa selenoprotein	893	20
TRUE	[BLASTED, I	TRINITY_DN76939_c1_g4_i1	heavy metal-associated i	1288	20
TRUE	[BLASTED, I	TRINITY_DN76939_c1_g4_i2	heavy metal-associated i	864	20
TRUE	[BLASTED, I	TRINITY_DN76994_c1_g1_i6	pollen receptor-like kina	4248	20
TRUE	[BLASTED]	TRINITY_DN76983_c1_g5_i2	36.4 kDa proline-rich pro	1272	20
TRUE	[BLASTED, I	TRINITY_DN72196_c0_g1_i2	VAN3-binding protein	1669	20
TRUE	[BLASTED, I	TRINITY_DN79632_c0_g1_i2	protein IQ-DOMAIN 1	1687	20
TRUE	[BLASTED, I	TRINITY_DN79668_c0_g1_i6	vacuolar protein sorting-	2016	20
TRUE	[BLASTED, I	TRINITY_DN79627_c1_g5_i3	programmed cell death p	2262	20
TRUE	[BLASTED, I	TRINITY_DN79694_c1_g5_i1	inactive beta-amylase 9	2117	20
TRUE	[BLASTED, I	TRINITY_DN79694_c1_g5_i7	inactive beta-amylase 9	1452	20
TRUE	[BLASTED, I	TRINITY_DN79694_c1_g5_i8	inactive beta-amylase 9	1674	20
TRUE	[BLASTED, I	TRINITY_DN75085_c0_g1_i2	U-box domain-containing	2647	20
TRUE	[BLASTED, I	TRINITY_DN81554_c0_g4_i1	matrix metalloproteinase	1877	20
TRUE	[BLASTED, I	TRINITY_DN81575_c1_g1_i10	calcium sensing receptor	1359	20
TRUE	[BLASTED, I	TRINITY_DN81566_c0_g1_i14	cytokinin riboside 5'-mor	660	20
TRUE	[BLASTED]	TRINITY_DN81591_c0_g1_i18	protein SRC2 homolog	3984	20
TRUE	[BLASTED, I	TRINITY_DN81548_c3_g1_i16	ABSCISIC ACID-INSENSITI	1832	20
TRUE	[BLASTED, I	TRINITY_DN81548_c3_g1_i25	ABSCISIC ACID-INSENSITI	2346	20
TRUE	[BLASTED, I	TRINITY_DN81512_c0_g5_i1	DNA mismatch repair pro	1297	20
TRUE	[BLASTED, I	TRINITY_DN81578_c0_g1_i4	alcohol-forming fatty acy	2381	20
TRUE	[BLASTED, I	TRINITY_DN81574_c0_g2_i1	metallothiol transferase	911	20
TRUE	[BLASTED, I	TRINITY_DN81560_c0_g1_i13	Alpha/Beta hydrolase fol	2019	20
TRUE	[BLASTED, I	TRINITY_DN81560_c0_g1_i14	Alpha/Beta hydrolase fol	2653	20
TRUE	[BLASTED, I	TRINITY_DN81568_c0_g1_i7	serine/arginine-rich splic	1152	20
TRUE	[BLASTED, I	TRINITY_DN81596_c0_g3_i1	monothiol glutaredoxin-S	636	20
TRUE	[BLASTED, I	TRINITY_DN81576_c1_g3_i5	telomere repeat-binding	2544	20
TRUE	[BLASTED, I	TRINITY_DN81576_c1_g2_i4	telomere repeat-binding	2553	20
TRUE	[BLASTED, I	TRINITY_DN65085_c0_g1_i1	nicotianamine synthase-l	1207	20
TRUE	[BLASTED, I	TRINITY_DN78098_c3_g2_i4	inositol-phosphate phosp	546	20
TRUE	[BLASTED]	TRINITY_DN78047_c0_g2_i6	Translation initiation fact	832	20
TRUE	[BLASTED]	TRINITY_DN78047_c0_g1_i9	vasodilator-stimulated pl	948	20
TRUE	[BLASTED, I	TRINITY_DN78084_c1_g1_i4	ubiquitin-conjugating en:	913	20
TRUE	[BLASTED, I	TRINITY_DN78060_c0_g2_i1	alpha-glucan phosphoryl	2611	20
TRUE	[BLASTED, I	TRINITY_DN78060_c0_g2_i10	alpha-glucan phosphoryl	4271	20
TRUE	[BLASTED, I	TRINITY_DN78090_c4_g1_i14	vacuolar protein-sorting-	3562	20
TRUE	[BLASTED, I	TRINITY_DN78031_c2_g3_i7	putative calcium-transpo	1077	20
TRUE	[BLASTED]	TRINITY_DN78079_c0_g1_i9	Lipid-binding serum glyc	2956	20
TRUE	[BLASTED, I	TRINITY_DN78013_c1_g1_i11	rhomboid-like protein 1	4823	20
TRUE	[BLASTED, I	TRINITY_DN68159_c0_g1_i2	2-keto-3-deoxy-L-rhamn	1177	20
TRUE	[BLASTED]	TRINITY_DN88095_c4_g2_i1	hypothetical protein H47	744	20
TRUE	[BLASTED, I	TRINITY_DN88009_c0_g1_i4	probable CCR4-associate	2608	20
TRUE	[BLASTED, I	TRINITY_DN88009_c0_g1_i8	glycine-rich RNA-binding	1762	12
TRUE	[BLASTED]	TRINITY_DN88009_c0_g1_i11	glycine-rich RNA-binding	1782	8
TRUE	[BLASTED, I	TRINITY_DN88067_c0_g2_i1	protein trichome birefrin	1894	20
TRUE	[BLASTED, I	TRINITY_DN88034_c2_g1_i17	endoribonuclease Dicer I	2439	20
TRUE	[BLASTED, I	TRINITY_DN88032_c0_g1_i1	DNA polymerase zeta cat	6395	20

TRUE	[BLASTED, I	TRINITY_DN88096_c1_g4_i2	protein argonaute 1-like	477	20
TRUE	[BLASTED, I	TRINITY_DN88096_c1_g1_i7	protein argonaute 1	2175	20
TRUE	[BLASTED, I	TRINITY_DN88046_c1_g1_i2	pentatricopeptide repeat	3388	20
TRUE	[BLASTED, I	TRINITY_DN88059_c1_g2_i1	INO80 complex subunit E	5069	20
TRUE	[BLASTED, I	TRINITY_DN88059_c1_g2_i21	INO80 complex subunit E	5055	20
TRUE	[BLASTED, I	TRINITY_DN88021_c1_g1_i15	phospholipid-transportin	3915	20
TRUE	[BLASTED, I	TRINITY_DN88072_c1_g1_i3	arabinogalactan peptide	374	20
TRUE	[BLASTED, I	TRINITY_DN88062_c1_g4_i3	chlorophyll a-b binding p	876	20
TRUE	[BLASTED, I	TRINITY_DN88062_c1_g4_i4	Chlorophyll a-b binding p	1260	20
TRUE	[BLASTED, I	TRINITY_DN88060_c3_g2_i10	serine carboxypeptidase-	1253	20
TRUE	[BLASTED, I	TRINITY_DN88076_c1_g1_i7	paired amphipathic helix	6337	20
TRUE	[BLASTED]	TRINITY_DN76616_c0_g1_i1	pentatricopeptide repeat	2287	20
TRUE	[BLASTED, I	TRINITY_DN76657_c0_g3_i1	NAD(P)H-quinone oxidor	942	20
TRUE	[BLASTED]	TRINITY_DN81827_c1_g1_i5	E3 ubiquitin-protein ligas	3340	20
TRUE	[BLASTED, I	TRINITY_DN81829_c1_g1_i3	protein DETOXIFICATION	1997	20
TRUE	[BLASTED, I	TRINITY_DN81842_c2_g1_i4	zeaxanthin epoxidase, ch	2267	20
TRUE	[BLASTED, I	TRINITY_DN81808_c1_g3_i3	HVA22-like protein k	811	20
TRUE	[BLASTED, I	TRINITY_DN81849_c0_g1_i13	protein-lysine N-methylt	1236	20
TRUE	[BLASTED, I	TRINITY_DN81879_c0_g2_i12	ISWI chromatin-remodel	3979	20
TRUE	[NO-BLAST	TRINITY_DN81885_c3_g4_i2	---NA---	1017	
TRUE	[BLASTED, I	TRINITY_DN81894_c0_g6_i1	heavy metal-associated i	797	20
TRUE	[BLASTED, I	TRINITY_DN81875_c1_g1_i1	probable mediator of RN	379	20
TRUE	[BLASTED, I	TRINITY_DN81875_c6_g5_i2	heat shock cognate 70 kD	1072	20
TRUE	[BLASTED, I	TRINITY_DN81843_c1_g1_i3	aquaporin TIP1-3-like	1045	20
TRUE	[BLASTED, I	TRINITY_DN81856_c0_g1_i18	bifunctional 3-dehydroqu	1151	20
TRUE	[NO-BLAST	TRINITY_DN81834_c0_g7_i1	---NA---	483	
TRUE	[BLASTED]	TRINITY_DN81834_c0_g4_i6	protein LNK1-like isoforn	1988	20
TRUE	[BLASTED]	TRINITY_DN81834_c0_g4_i10	protein LNK1-like isoforn	2531	20
TRUE	[BLASTED, I	TRINITY_DN81886_c0_g1_i5	Cell division control prot	4481	20
TRUE	[BLASTED, I	TRINITY_DN81814_c0_g6_i1	suppressor of disruption	1028	20
TRUE	[BLASTED, I	TRINITY_DN81817_c3_g1_i4	2-alkenal reductase (NAD	1910	20
TRUE	[BLASTED, I	TRINITY_DN77231_c0_g3_i2	autophagy-related prote	1230	20
TRUE	[BLASTED, I	TRINITY_DN79754_c2_g1_i1	Chloroplast sensor kinase	985	20
TRUE	[NO-BLAST	TRINITY_DN79736_c0_g2_i2	---NA---	2507	
TRUE	[BLASTED, I	TRINITY_DN79794_c0_g2_i5	ABC transporter G family	1707	20
TRUE	[BLASTED, I	TRINITY_DN79794_c0_g1_i3	protein FAM32A-like	820	20
TRUE	[BLASTED, I	TRINITY_DN79794_c0_g2_i15	ABC transporter G family	3047	20
TRUE	[BLASTED, I	TRINITY_DN79786_c0_g2_i7	oxalate--CoA ligase-like	2364	20
TRUE	[BLASTED, I	TRINITY_DN79790_c1_g1_i5	vacuolar-processing enzy	1532	20
TRUE	[BLASTED, I	TRINITY_DN79770_c0_g1_i14	serine/threonine-proteir	4311	20
TRUE	[BLASTED]	TRINITY_DN79780_c7_g1_i8	uncharacterized protein	1323	20
TRUE	[BLASTED, I	TRINITY_DN79725_c1_g3_i2	copper-transporting ATP	3046	20
TRUE	[BLASTED]	TRINITY_DN79702_c0_g5_i2	LOB domain-containing p	1129	20
TRUE	[BLASTED]	TRINITY_DN79702_c0_g6_i1	LOB domain-containing p	1038	20
TRUE	[BLASTED, I	TRINITY_DN79705_c0_g3_i3	formate dehydrogenase,	2295	20
TRUE	[BLASTED]	TRINITY_DN79703_c0_g3_i1	thaumatin-like protein 1l	1551	20
TRUE	[BLASTED, I	TRINITY_DN85095_c0_g2_i12	dnaJ protein ERDJ2A-like	1467	20
TRUE	[BLASTED]	TRINITY_DN85014_c1_g11_i2	perakine reductase-like	457	20
TRUE	[BLASTED, I	TRINITY_DN85059_c1_g2_i8	UDP-galactose transporte	1586	20
TRUE	[BLASTED, I	TRINITY_DN85011_c0_g1_i4	probable serine/threonir	2701	20

TRUE	[BLASTED,	TRINITY_DN85057_c0_g1_i28	hypothetical protein CQ	1022	20
TRUE	[BLASTED]	TRINITY_DN85041_c1_g1_i19	B2 protein-like	1463	20
TRUE	[BLASTED]	TRINITY_DN85074_c1_g2_i8	uncharacterized protein	4532	20
TRUE	[BLASTED,	TRINITY_DN85053_c0_g1_i5	L-ascorbate oxidase hom	2077	20
TRUE	[BLASTED,	TRINITY_DN85053_c0_g1_i7	L-ascorbate oxidase hom	2071	20
TRUE	[BLASTED,	TRINITY_DN85050_c0_g1_i14	kinesin-like protein KIN-	3013	20
TRUE	[BLASTED,	TRINITY_DN85003_c1_g1_i6	PH, RCC1 and FYVE doma	2011	20
TRUE	[BLASTED,	TRINITY_DN85086_c0_g2_i13	calcineurin B-like protein	3477	20
TRUE	[BLASTED,	TRINITY_DN85077_c5_g2_i7	ATP synthase CF1 alpha	2968	20
TRUE	[BLASTED,	TRINITY_DN85644_c8_g4_i1	heat shock cognate 70 k	2676	20
TRUE	[BLASTED,	TRINITY_DN85681_c2_g1_i7	SET and MYND domain-c	4599	20
TRUE	[BLASTED,	TRINITY_DN85640_c1_g2_i3	homeobox protein HAT3	1488	20
TRUE	[BLASTED,	TRINITY_DN85640_c1_g2_i8	pathogenesis-related ho	2092	20
TRUE	[BLASTED,	TRINITY_DN85616_c2_g2_i8	DEAD-box ATP-depender	2188	20
TRUE	[BLASTED,	TRINITY_DN85620_c2_g6_i3	SUMO-conjugating enzy	1197	20
TRUE	[BLASTED,	TRINITY_DN85620_c2_g5_i3	probable GTP-binding pr	1124	20
TRUE	[BLASTED,	TRINITY_DN85620_c2_g5_i4	probable GTP-binding pr	1090	20
TRUE	[BLASTED]	TRINITY_DN85610_c1_g1_i1	zinc finger MYND domai	1795	20
TRUE	[BLASTED,	TRINITY_DN85677_c0_g1_i4	zinc transporter 5-like	1118	20
TRUE	[BLASTED,	TRINITY_DN85653_c1_g1_i13	RNA polymerase sigma f	2844	20
TRUE	[BLASTED,	TRINITY_DN85605_c2_g2_i1	sister chromatid cohesio	6623	20
TRUE	[BLASTED,	TRINITY_DN85605_c2_g2_i2	sister chromatid cohesio	6703	20
TRUE	[BLASTED,	TRINITY_DN85671_c2_g2_i12	uncharacterized aarF do	1994	20
TRUE	[BLASTED,	TRINITY_DN85694_c0_g1_i4	DEAD-box ATP-depender	2553	20
TRUE	[BLASTED,	TRINITY_DN85694_c0_g1_i10	DEAD-box ATP-depender	2267	20
TRUE	[BLASTED]	TRINITY_DN85631_c0_g1_i4	PREDICTED: uncharacteri	643	20
TRUE	[BLASTED,	TRINITY_DN85629_c3_g1_i1	transcription factor bHL	2436	20
TRUE	[BLASTED,	TRINITY_DN84676_c2_g1_i2	protein NUCLEAR FUSIO	2698	20
TRUE	[BLASTED,	TRINITY_DN84668_c0_g1_i2	protein phosphatase 2C	1606	20
TRUE	[BLASTED,	TRINITY_DN84616_c2_g3_i2	glycine-rich RNA-binding	911	20
TRUE	[BLASTED,	TRINITY_DN84646_c3_g1_i15	hydroxyproline O-galact	846	20
TRUE	[BLASTED,	TRINITY_DN84692_c1_g1_i11	dehydrogenase/reducta	1750	20
TRUE	[BLASTED,	TRINITY_DN84696_c0_g1_i11	diacylglycerol kinase 5	2303	20
TRUE	[BLASTED,	TRINITY_DN84626_c2_g2_i4	probable nucleoredoxin	2092	20
TRUE	[BLASTED,	TRINITY_DN84637_c2_g1_i4	CLP protease regulatory	2835	20
TRUE	[BLASTED,	TRINITY_DN84673_c0_g3_i1	CBL-interacting serine/th	1451	20
TRUE	[BLASTED,	TRINITY_DN84643_c3_g1_i3	FAM10 family protein At	2144	20
TRUE	[BLASTED,	TRINITY_DN84622_c0_g1_i2	ABC transporter G family	2470	20
TRUE	[BLASTED,	TRINITY_DN84622_c0_g1_i3	ABC transporter G family	2083	20
TRUE	[BLASTED,	TRINITY_DN84622_c1_g6_i3	probable E3 ubiquitin lig	2515	20
TRUE	[BLASTED,	TRINITY_DN84629_c1_g2_i4	crooked neck-like protei	2910	20
TRUE	[BLASTED]	TRINITY_DN84690_c0_g1_i3	WPP domain-interacting	1504	3
TRUE	[BLASTED,	TRINITY_DN84682_c1_g2_i2	CDP-diacylglycerol--serin	1838	20
TRUE	[BLASTED,	TRINITY_DN84682_c1_g2_i3	CDP-diacylglycerol--serin	2250	20
TRUE	[BLASTED,	TRINITY_DN84608_c0_g1_i12	beta carbonic anhydrase	2169	20
TRUE	[BLASTED]	TRINITY_DN84615_c1_g3_i4	fold protein	1308	20
TRUE	[BLASTED,	TRINITY_DN84614_c6_g1_i20	UDP-glucose 4-epimeras	880	20
TRUE	[BLASTED]	TRINITY_DN84601_c0_g1_i5	Polyadenylate-binding pr	2569	20
TRUE	[BLASTED,	TRINITY_DN84672_c0_g1_i3	30S ribosomal protein S1	1223	20
TRUE	[BLASTED,	TRINITY_DN84672_c0_g1_i9	30S ribosomal protein S1	1161	20

TRUE	[BLASTED, I	TRINITY_DN84650_c1_g1_i19	bifunctional 3-dehydroqu	1165	20
TRUE	[BLASTED, I	TRINITY_DN73379_c0_g1_i1	dirigent protein 1-like	990	20
TRUE	[BLASTED]	TRINITY_DN86851_c2_g3_i7	integrator complex subu	2064	20
TRUE	[BLASTED, I	TRINITY_DN86856_c0_g1_i9	probable bifunctional me	2208	20
TRUE	[BLASTED, I	TRINITY_DN86856_c0_g1_i20	probable bifunctional me	2091	20
TRUE	[BLASTED, I	TRINITY_DN86868_c2_g2_i3	50S ribosomal protein	776	20
TRUE	[BLASTED, I	TRINITY_DN86868_c3_g2_i11	dolichol kinase EVAN isof	1910	20
TRUE	[BLASTED, I	TRINITY_DN86806_c2_g2_i8	chlorophyll a-b binding p	906	20
TRUE	[BLASTED, I	TRINITY_DN86813_c4_g3_i1	WAT1-related protein At	1462	20
TRUE	[BLASTED, I	TRINITY_DN86813_c4_g5_i1	protein PLASTID MOVEM	3225	20
TRUE	[BLASTED, I	TRINITY_DN86808_c2_g1_i12	suppressor of mec-8 and	1336	20
TRUE	[BLASTED, I	TRINITY_DN86865_c0_g1_i2	transcription factor BIM1	1197	20
TRUE	[BLASTED, I	TRINITY_DN86865_c0_g1_i9	transcription factor BIM1	2766	20
TRUE	[BLASTED, I	TRINITY_DN86881_c0_g2_i1	histone deacetylase HDT	887	20
TRUE	[BLASTED, I	TRINITY_DN86881_c0_g2_i8	histone deacetylase HDT	973	20
TRUE	[BLASTED, I	TRINITY_DN86869_c0_g1_i17	long chain acyl-CoA synth	2534	20
TRUE	[BLASTED, I	TRINITY_DN86869_c0_g1_i19	long chain acyl-CoA synth	2581	20
TRUE	[BLASTED]	TRINITY_DN86848_c0_g7_i5	protein ENHANCED DOW	3043	20
TRUE	[BLASTED, I	TRINITY_DN86889_c2_g3_i6	40S ribosomal protein S2	1171	20
TRUE	[BLASTED]	TRINITY_DN86836_c2_g1_i6	protein PLASTID REDOX I	2039	20
TRUE	[BLASTED]	TRINITY_DN86810_c4_g4_i3	EIN3-binding F-box prote	1285	20
TRUE	[BLASTED, I	TRINITY_DN86886_c0_g2_i10	mitogen-activated protei	3360	20
TRUE	[BLASTED, I	TRINITY_DN88601_c2_g2_i4	apoptotic chromatin con	1385	20
TRUE	[NO-BLAST	TRINITY_DN88653_c2_g2_i2	---NA---	1395	
TRUE	[BLASTED, I	TRINITY_DN88648_c2_g1_i4	NADP-dependent D-sorb	1028	20
TRUE	[BLASTED, I	TRINITY_DN88648_c2_g1_i9	NADP-dependent D-sorb	916	20
TRUE	[BLASTED, I	TRINITY_DN88648_c2_g1_i12	NADP-dependent D-sorb	1414	20
TRUE	[BLASTED]	TRINITY_DN88672_c1_g1_i14	protein FAR1-RELATED SI	3021	20
TRUE	[BLASTED, I	TRINITY_DN88686_c6_g1_i6	ribulose bisphosphate ca	806	20
TRUE	[BLASTED, I	TRINITY_DN88686_c6_g4_i2	ribulose bisphosphate ca	334	20
TRUE	[BLASTED, I	TRINITY_DN88686_c6_g2_i2	Ribulose bisphosphate ca	387	20
TRUE	[BLASTED, I	TRINITY_DN88686_c6_g6_i2	ribulose bisphosphate ca	250	20
TRUE	[BLASTED, I	TRINITY_DN88675_c1_g1_i25	vacuolar cation/proton e	3995	20
TRUE	[BLASTED]	TRINITY_DN88669_c3_g1_i8	GYF domain containing p	1683	20
TRUE	[BLASTED, I	TRINITY_DN88618_c2_g2_i9	probable 1-acylglycerol-3	1266	20
TRUE	[BLASTED, I	TRINITY_DN88630_c5_g1_i4	fructose-bisphosphate al	427	20
TRUE	[BLASTED, I	TRINITY_DN88612_c0_g2_i6	UNC93-like protein 3	2861	20
TRUE	[BLASTED, I	TRINITY_DN88684_c2_g2_i1	probable leucine-rich rep	652	20
TRUE	[BLASTED, I	TRINITY_DN88684_c2_g3_i2	probable LRR receptor-lil	2219	20
TRUE	[BLASTED, I	TRINITY_DN88655_c1_g1_i2	C2 domain-containing pr	3620	20
TRUE	[BLASTED, I	TRINITY_DN88655_c1_g1_i9	C2 domain-containing pr	2836	20
TRUE	[BLASTED, I	TRINITY_DN88687_c1_g1_i1	plasma membrane ATPas	3213	20
TRUE	[BLASTED, I	TRINITY_DN74883_c0_g1_i4	Serine--tRNA ligase	3278	20
TRUE	[BLASTED, I	TRINITY_DN87806_c3_g3_i10	probable zinc metallopep	1859	20
TRUE	[BLASTED, I	TRINITY_DN87806_c3_g3_i12	probable zinc metallopep	2190	20
TRUE	[BLASTED, I	TRINITY_DN87806_c3_g3_i14	probable zinc metallopep	2293	20
TRUE	[BLASTED, I	TRINITY_DN87845_c1_g4_i1	thermospermine synthas	1243	20
TRUE	[BLASTED]	TRINITY_DN87870_c0_g1_i1	serine/threonine-protein	1998	20
TRUE	[BLASTED, I	TRINITY_DN87835_c4_g2_i25	serine hydroxymethyltra	3108	20
TRUE	[BLASTED, I	TRINITY_DN87866_c0_g1_i19	exocyst complex compor	4407	20

TRUE	[BLASTED, I	TRINITY_DN87849_c0_g1_i3	sphinganine C4-monooxy	1433	20
TRUE	[BLASTED]	TRINITY_DN87820_c0_g1_i1	Protein like	762	20
TRUE	[BLASTED]	TRINITY_DN87820_c0_g1_i2	Protein like	767	20
TRUE	[BLASTED, I	TRINITY_DN87820_c1_g1_i8	splicing factor U2af large	1149	20
TRUE	[BLASTED, I	TRINITY_DN87889_c1_g1_i1	sodium/hydrogen exchar	1275	20
TRUE	[BLASTED, I	TRINITY_DN87838_c0_g4_i3	transcription terminatior	1779	20
TRUE	[BLASTED, I	TRINITY_DN87860_c0_g1_i3	mediator of RNA polyme	1700	20
TRUE	[BLASTED, I	TRINITY_DN87880_c1_g5_i3	NADPH--cytochrome P45	970	20
TRUE	[BLASTED, I	TRINITY_DN87825_c0_g4_i3	zinc finger CCCH domain-	2934	20
TRUE	[BLASTED, I	TRINITY_DN87807_c1_g2_i6	topless-related protein 3	970	20
TRUE	[BLASTED]	TRINITY_DN87888_c1_g1_i3	MAGUK p55 subfamily m	874	20
TRUE	[BLASTED]	TRINITY_DN76217_c1_g1_i9	Zinc finger, FYVE/PHD-ty	1681	20
TRUE	[BLASTED, I	TRINITY_DN76299_c0_g1_i7	PI-PLC X-box domain-con	2339	20
TRUE	[BLASTED]	TRINITY_DN76284_c0_g3_i2	F-box/kelch-repeat prote	1446	20

of ON- *versus* OFF-crop trees

l Zeinalabedini[5] and Seyed Alireza Salami [6]

les, ON- *vs.* OFF-trees

e-Value	sim mean	#GO	GO IDs
3.05E-131	83.54	1	P:GO:0009408
0	97.43	7	P:GO:0006537; C:GO:0009536; F:GO:0015930;
0	83.31	5	F:GO:0000155; P:GO:0000160; F:GO:0005524;
0	83.25	3	F:GO:0005524; C:GO:0016021; F:GO:0016887
0	91.89	3	F:GO:0005524; C:GO:0016021; F:GO:0016887
0	79.22	1	C:GO:0016021
5.56E-19	82.31	10	F:GO:0004471; F:GO:0004473; P:GO:0006108;
0	93.95	5	F:GO:0004088; F:GO:0005524; P:GO:0006541;
0	86.78	20	F:GO:0000155; P:GO:0000160; C:GO:0005622;
6.42E-09	62.69	2	C:GO:0016020; C:GO:0016021
0	87.47	5	C:GO:0000139; C:GO:0016021; P:GO:0045489; F:GO:0C
0	93.02	3	P:GO:0001676; F:GO:0004467; F:GO:0102391
0	93.02	3	P:GO:0001676; F:GO:0004467; F:GO:0102391
0	90.41	5	C:GO:0000139; P:GO:0006886; F:GO:0008270; C:GO:0C
1.77E-121	82.13	4	F:GO:0003723; C:GO:0005634; C:GO:0005737; P:GO:0C
4.17E-110	79.97	5	F:GO:0003723; C:GO:0005634; C:GO:0005737; P:GO:0C
9.26E-38	65.31		
1.94E-50	69.72	1	C:GO:0044424
7.10E-99	93.3		
0	71.92	3	C:GO:0005634; P:GO:0009416; P:GO:0009909
5.32E-75	86.31	2	C:GO:0005634; F:GO:0008270
3.27E-168	69.83	1	C:GO:0005622
1.70E-111	91.5	3	F:GO:0016621; F:GO:0050662; P:GO:0055114
5.30E-136	84.13	2	F:GO:0004402; P:GO:0016573
0	75.19	3	P:GO:0009987; F:GO:0016491; P:GO:0055114
1.66E-77	88.24	1	F:GO:0003723
1.11E-172	97.41	5	P:GO:0006006; F:GO:0016620; F:GO:0050661; F:GO:00
1.45E-54	68.9		
1.42E-104	92.79	2	F:GO:0004722; P:GO:0006470
0	97.94	2	F:GO:0005524; C:GO:0016021
0	93.26	1	F:GO:0005524
0	98.09	4	F:GO:0005524; C:GO:0005783; F:GO:0032440; P:GO:00
4.84E-126	72.53	3	F:GO:0004252; C:GO:0005777; P:GO:0016485
0	86.08	6	P:GO:0000381; P:GO:0000389; F:GO:0003723; C:GO:0C
0	86.81	6	P:GO:0000381; P:GO:0000389; F:GO:0003723; C:GO:0C
0	86.08	6	P:GO:0000381; P:GO:0000389; F:GO:0003723;
7.53E-52	56.54	1	F:GO:0016491
1.42E-88	85.85	24	F:GO:0000155; P:GO:0000160; C:GO:0005622;
0	65.25		
1.14E-70	69.19		
2.41E-155	74.44	1	F:GO:0043531
1.68E-155	74.44	1	F:GO:0043531
0	68.39		
0	73.18	4	C:GO:0005737; P:GO:0009987; F:GO:0031625; F:GO:00

0	79.41	2	F:GO:0008168; P:GO:0032259
1.45E-89	92.34	4	P:GO:0000165; F:GO:0004707; F:GO:0005524; C:GO:00
0	87.62	3	F:GO:0016614; F:GO:0050660; P:GO:0055114
0	94.38	8	C:GO:0000408; P:GO:0002949; C:GO:0005634; C:GO:00
7.13E-60	61.04	1	F:GO:0003677
0	86.76	1	C:GO:0016021
3.72E-109	91.96	4	F:GO:0005524; C:GO:0016021; F:GO:0042626; P:GO:00
4.94E-96	90.18		
0	79	3	F:GO:0003779; C:GO:0005856; P:GO:0030036
0	82.09	5	F:GO:0008171; F:GO:0008757; P:GO:0019438;
3.84E-63	85.81	3	F:GO:0008171; P:GO:0032259; F:GO:0046983
1.10E-157	76.07	2	C:GO:0005643; P:GO:0016973
1.08E-68	85.92	10	C:GO:0005737; C:GO:0005868; C:GO:0005874;
1.58E-101	79.03	1	F:GO:0016740
1.19E-133	76.26		
2.68E-37	91.72	5	C:GO:0009535; C:GO:0009654; P:GO:0009735; P:GO:00
1.42E-28	93.6	5	C:GO:0009535; C:GO:0009654; P:GO:0009735; P:GO:00
0	88.02	3	F:GO:0004252; P:GO:0006508; F:GO:0008240
1.57E-119	79.71	7	P:GO:0006629; P:GO:0010345; P:GO:0035336;
0	56.63	7	P:GO:0006807; F:GO:0016740; F:GO:0016787;
0	85.47	3	P:GO:0007035; C:GO:0043291; P:GO:0070072
0	85.47	3	P:GO:0007035; C:GO:0043291; P:GO:0070072
0	69.7	1	F:GO:0043531
0	74.84		
4.78E-69	85.61	1	C:GO:0016021
2.01E-30	86.83	2	F:GO:0004527; P:GO:0090305
0	93.35	4	F:GO:0004674; F:GO:0005524; P:GO:0006468; C:GO:00
6.87E-145	96.85	4	F:GO:0004821; F:GO:0005524; C:GO:0005737; P:GO:00
6.54E-135	73.27	1	F:GO:0003676
2.09E-93	70.11	1	F:GO:0003676
2.17E-179	90.27	4	C:GO:0005774; C:GO:0005783; C:GO:0005794; C:GO:00
0	83.17	2	C:GO:0000145; P:GO:0006893
2.00E-63	96.04	2	F:GO:0004332; P:GO:0006096
0	80.85	5	F:GO:0003723; F:GO:0004519; P:GO:0009451; C:GO:00
0	83.7	2	F:GO:0003723; F:GO:0046872
0	83.82	2	F:GO:0003723; F:GO:0046872
2.13E-131	73.56	4	F:GO:0004497; P:GO:0044550; P:GO:0055114; F:GO:00
1.44E-87	80.97	3	C:GO:0016021; F:GO:0016491; P:GO:0055114
6.23E-150	67.94	5	C:GO:0005622; F:GO:0008270; C:GO:0016020; C:GO:00
7.39E-51	85.55	5	C:GO:0005634; P:GO:0008152; F:GO:0008422; F:GO:00
2.23E-147	78.72		
1.27E-166	92.34	5	P:GO:0000413; F:GO:0003755; F:GO:0005528; C:GO:00
0	65.11	1	P:GO:0044237
3.92E-94	77.97	2	F:GO:0004553; P:GO:0005975
2.99E-70	94.91	8	P:GO:0000398; F:GO:0003676; F:GO:0004004;
0	87.5	8	F:GO:0008517; C:GO:0009941; F:GO:0015231; F:GO:00
0	67.88	2	F:GO:0003824; P:GO:0008152
0	68.09	2	F:GO:0003824; P:GO:0008152

3.45E-65	96.62	3	F:GO:0015267; C:GO:0016021; P:GO:0055085
0	96.6	4	F:GO:0004594; F:GO:0005524; P:GO:0015937; P:GO:00
7.00E-74	69.02	1	C:GO:0005829
0	86.11	4	F:GO:0004970; P:GO:0006811; C:GO:0016021; P:GO:00
0	69.4	1	C:GO:0009506
3.82E-144	65.98	1	C:GO:0005634
4.40E-103	90.21	1	C:GO:0016021
0	86.17	1	C:GO:0016021
0	68		
0	86.55		
2.15E-136	91.1	4	F:GO:0003684; C:GO:0005634; P:GO:0006289; P:GO:00
4.41E-137	91.35	4	F:GO:0003684; C:GO:0005634; P:GO:0006289; P:GO:00
0	91.91	8	P:GO:0006809; F:GO:0009703; F:GO:0020037; F:GO:00
2.14E-125	90.83	2	F:GO:0016491; P:GO:0055114
1.79E-169	91.17	2	F:GO:0016491; P:GO:0055114
0	86.12	3	C:GO:0016021; F:GO:0022857; P:GO:0055085
0	98.85	4	F:GO:0005524; P:GO:0006457; P:GO:0006950; F:GO:00
1.17E-137	98.17	5	F:GO:0005524; C:GO:0005737; P:GO:0006457; P:GO:00
0	98.98	4	F:GO:0005524; P:GO:0006457; P:GO:0006950; F:GO:00
6.56E-137	70.21		
0	90.21		
1.21E-58	100	6	C:GO:0000786; F:GO:0003677; C:GO:0005730; C:GO:00
7.50E-70	96.96	9	F:GO:0004367; P:GO:0005975; P:GO:0006650; P:GO:00
0	99.66	6	C:GO:0000502; F:GO:0005524; C:GO:0005737; F:GO:00
0	84.35		
0	84.37		
0	84.37		
0	73.13	2	F:GO:0003700; P:GO:0006355
5.79E-164	66.14	2	F:GO:0003700; P:GO:0006355
0	81.3	1	F:GO:0046982
0	94.2	6	C:GO:0016021; F:GO:0016702; F:GO:0016717; P:GO:00
0	86.1	5	C:GO:0005829; P:GO:0016099; F:GO:0016628; F:GO:00
0	79.27	1	P:GO:0006355
1.60E-131	95.57	2	P:GO:0005985; F:GO:0016157
3.51E-178	93.61	1	F:GO:0019904
4.20E-16	64.9	2	F:GO:0016301; P:GO:0016310
1.91E-105	72.69		
2.24E-154	90.79	4	C:GO:0005789; P:GO:0009733; C:GO:0016021; P:GO:00
0	91.6	1	C:GO:0016021
0	88.34	1	C:GO:0016021
1.38E-126	84.38	5	F:GO:0003838; C:GO:0016021; P:GO:0032259; F:GO:00
8.95E-73	62.8		
0	81.56	3	F:GO:0003677; C:GO:0005634; P:GO:0006355
0	80.38	3	F:GO:0003677; C:GO:0005634; P:GO:0006355
1.26E-113	66.44	1	F:GO:0016874
2.89E-37	83.8	1	F:GO:0003723
0	86.45	10	F:GO:0000155; F:GO:0005524; C:GO:0005789; P:GO:00

0	88.16	10	F:GO:0000155; F:GO:0005524; C:GO:0005789; P:GO:00
0	97.18		
0	92.26	3	P:GO:0005975; P:GO:0009845; F:GO:0016985
4.97E-167	71.74	1	P:GO:0050789
8.39E-66	85.76		
8.55E-167	71.73	1	P:GO:0050789
1.07E-135	84.73	3	F:GO:0003735; P:GO:0006412; C:GO:0022627
0	90.81	13	P:GO:0000209; C:GO:0005634; C:GO:0005829; P:GO:00
1.46E-108	81.72	4	F:GO:0003676; P:GO:0015074; F:GO:0016301; P:GO:00
3.06E-93	72.21		
4.65E-80	74.13		
5.88E-147	97.12	3	P:GO:0005975; P:GO:0016311; F:GO:0050278
2.02E-14	58.22	7	F:GO:0003824; P:GO:0006506; P:GO:0008152; C:GO:00
4.71E-85	87.86	3	C:GO:0016021; F:GO:0016301; P:GO:0016310
1.99E-86	77.61	1	C:GO:0016021
0	83.21	3	P:GO:0006506; C:GO:0016021; F:GO:0016746
5.85E-34	92.64	4	C:GO:0000786; F:GO:0003677; C:GO:0005634; P:GO:00
0	72.43	2	F:GO:0003676; F:GO:0003723
3.82E-121	76.49	3	C:GO:0016021; F:GO:0022857; P:GO:0055085
0	94.64	1	F:GO:0003723
1.20E-85	81.14	1	C:GO:0016021
1.92E-107	83.83	1	C:GO:0016021
8.13E-47	90.99	1	F:GO:0005524
0	99.43	3	F:GO:0005524; F:GO:0032440; P:GO:0055114
0	97.27	2	F:GO:0005524; P:GO:0019538
0	96.62	2	F:GO:0005524; P:GO:0019538
2.90E-133	74.24	2	C:GO:0005773; P:GO:0010200
0	79.16	6	F:GO:0004693; F:GO:0005524; C:GO:0005634; P:GO:00
2.55E-54	86.88		
1.36E-146	94.81		
0	91.63	4	C:GO:0005887; F:GO:0008271; C:GO:0009507; P:GO:19
7.54E-109	90.42	1	C:GO:0016021
1.70E-108	87.96	1	C:GO:0016021
6.49E-34	93.82		
3.99E-34	93.73		
1.05E-63	91.87	2	P:GO:0006629; F:GO:0008970
0	84.22	2	P:GO:0006629; F:GO:0008970
1.60E-67	78.44	3	F:GO:0003962; P:GO:0009086; F:GO:0030170
5.65E-57	62.52		
0	84.66	4	F:GO:0004014; P:GO:0006557; P:GO:0006597; P:GO:00
0	81.04		
1.10E-116	81.43	2	C:GO:0005773; P:GO:0006950
9.13E-64	99.36	6	C:GO:0000786; F:GO:0003677; C:GO:0005634; P:GO:00
7.51E-78	98.27	6	C:GO:0000786; P:GO:0006334; C:GO:0010369; C:GO:00
9.47E-77	99.41	6	C:GO:0000786; P:GO:0006334; C:GO:0010369; C:GO:00
3.18E-77	96.67	4	C:GO:0000786; F:GO:0003677; C:GO:0030875; F:GO:00
4.59E-77	96.38	4	C:GO:0000786; F:GO:0003677; C:GO:0030875; F:GO:00
2.57E-71	91.51	3	F:GO:0003977; C:GO:0005829; P:GO:0006048

0	85.4	3	F:GO:0004017; F:GO:0005524; P:GO:0046939
1.82E-74	87.05	3	F:GO:0004017; F:GO:0005524; P:GO:0046939
9.99E-87	76.81	2	F:GO:0008233; C:GO:0016021
0	88.58	7	F:GO:0004674; F:GO:0005524; C:GO:0005634; C:GO:00
7.88E-178	84.79	2	F:GO:0003993; P:GO:0016311
0	92.89	8	F:GO:0003836; C:GO:0005768; C:GO:0005802; P:GO:00
0	89.64	1	F:GO:0016798
2.39E-117	80.51		
0	89.32		
0	89.32		
0	80.83	3	P:GO:0006886; P:GO:0016192; C:GO:0030123
4.05E-130	60.85	6	F:GO:0003743; P:GO:0006413; P:GO:0008152; F:GO:00
1.59E-162	57.9	6	F:GO:0003743; P:GO:0006413; P:GO:0008152; F:GO:00
7.83E-105	90.37		
0	58.22		
0	87.84	4	F:GO:0004672; F:GO:0005509; F:GO:0005524; P:GO:00
0	92.28	4	F:GO:0004672; F:GO:0005509; F:GO:0005524; P:GO:00
0	88.75	5	F:GO:0004134; P:GO:0005977; F:GO:0016787; F:GO:01
4.69E-170	78.32	1	F:GO:0003676
6.52E-17	89.36		
0	84.17	3	F:GO:0003827; P:GO:0006486; C:GO:0016021
0	84.17	3	F:GO:0003827; P:GO:0006486; C:GO:0016021
1.33E-40	84.91	6	F:GO:0004843; C:GO:0005634; C:GO:0005829; P:GO:00
5.59E-85	83.48	3	F:GO:0005524; P:GO:0006508; F:GO:0008233
5.69E-21	100	7	F:GO:0005086; C:GO:0005784; P:GO:0006616; F:GO:00
2.26E-175	80.85	1	C:GO:0016021
0	88.44		
0	97.07	10	F:GO:0005524; C:GO:0005739; C:GO:0005829; P:GO:00
8.93E-150	91.27	6	F:GO:0003854; F:GO:0047568; F:GO:0047787; F:GO:00
1.38E-32	100		
0	75.66	1	F:GO:0008270
0	81.12	7	F:GO:0004675; F:GO:0005524; C:GO:0005886; P:GO:00
0	81.82	4	F:GO:0005504; P:GO:0006631; C:GO:0009570; F:GO:00
2.41E-54	70.86	2	F:GO:0005516; P:GO:0006950
1.20E-173	94.91	6	C:GO:0005887; P:GO:0006833; C:GO:0009705; F:GO:00
2.86E-39	88.54	3	F:GO:0004197; P:GO:0006508; P:GO:0050790
1.06E-92	97.35	5	F:GO:0005506; C:GO:0016021; F:GO:0016710; F:GO:00
2.59E-172	82.53	1	F:GO:0003723
3.05E-10	69.35		
0	84.49	1	C:GO:0016021
0	79.26	3	P:GO:0008152; C:GO:0016021; F:GO:0016758
8.77E-117	94.89	5	P:GO:0000186; F:GO:0004709; F:GO:0005524; C:GO:00
0	78.99	1	F:GO:0016757
0	83.34	3	P:GO:0008152; C:GO:0009536; F:GO:0016758
7.90E-130	91.3	2	C:GO:0005634; P:GO:0006333
0	86.24	1	F:GO:0003676
0	89.88	3	P:GO:0016226; F:GO:0016746; C:GO:0097361
7.95E-144	77.19	1	C:GO:0016021

0	94.05		
8.34E-80	81.34	2	F:GO:0008168; P:GO:0032259
0	83.82	7	F:GO:0003777; F:GO:0005524; P:GO:0007018; F:GO:00
3.42E-20	97.28	2	F:GO:0003723; C:GO:0016021
2.80E-166	80.86		
0	83.22		
0	97.75	6	F:GO:0004674; F:GO:0005524; C:GO:0005634; C:GO:00
7.84E-152	79.48	8	P:GO:0001101; F:GO:0003677; C:GO:0005634; P:GO:00
5.20E-130	84.4	5	F:GO:0005509; C:GO:0009535; C:GO:0009654; P:GO:00
1.90E-101	67.91	1	F:GO:0016740
8.36E-37	94.15	3	F:GO:0003735; C:GO:0005840; P:GO:0006414
9.88E-71	92.69	2	F:GO:0003676; F:GO:0008270
0	93.68	3	F:GO:0003985; C:GO:0005829; P:GO:0006635
0	93.68	3	F:GO:0003985; C:GO:0005829; P:GO:0006635
0	89.99	2	F:GO:0004722; P:GO:0006470
2.07E-61	79.18		
0	86.36	4	F:GO:0000287; C:GO:0005829; F:GO:0008253; P:GO:00
0	86.3	4	F:GO:0000287; C:GO:0005829; F:GO:0008253; P:GO:00
0	85.46	4	P:GO:0008643; C:GO:0016021; F:GO:0022857; P:GO:00
1.48E-61	98.32		
9.74E-167	85.1	4	F:GO:0004331; F:GO:0016301; P:GO:0016310; P:GO:00
0	92.78	3	F:GO:0004672; F:GO:0005524; P:GO:0006468
0	94.7	4	F:GO:0004672; F:GO:0005524; P:GO:0006468; C:GO:00
0	98.04	3	F:GO:0004674; F:GO:0005524; P:GO:0006468
7.52E-11	76.79		
4.60E-128	58.1	1	F:GO:0016740
1.60E-148	97.78	6	F:GO:0000149; F:GO:0005484; P:GO:0006887; P:GO:00
3.63E-49	88	2	C:GO:0009507; C:GO:0016021
8.02E-50	87.72	3	C:GO:0009535; C:GO:0016021; P:GO:0055085
1.47E-44	60.23	4	C:GO:0009535; C:GO:0016020; C:GO:0016021; P:GO:00
1.24E-64	66.1	2	C:GO:0009507; C:GO:0016020
8.98E-44	87.13	1	C:GO:0009507
1.60E-39	86.93	2	C:GO:0009507; C:GO:0016021
2.99E-119	88.3	4	F:GO:0004316; P:GO:0055114; F:GO:0102131; F:GO:01
0	76.08		
2.27E-167	61.74	2	C:GO:0016020; C:GO:0016021
1.64E-166	61.74	2	C:GO:0016020; C:GO:0016021
0	86.85	5	F:GO:0004650; C:GO:0005576; P:GO:0005975; C:GO:00
1.84E-31	94.3	6	F:GO:0003676; F:GO:0004004; F:GO:0005524; C:GO:00
0	74.72	1	P:GO:0009987
0	75.44	3	P:GO:0006812; C:GO:0016020; P:GO:0055085
0	95.63	5	C:GO:0009654; P:GO:0010207; F:GO:0010242; P:GO:00
5.39E-34	68.43	4	F:GO:0003677; F:GO:0004386; F:GO:0005524; C:GO:00
9.21E-99	93.95	6	F:GO:0003743; P:GO:0006355; P:GO:0006413; F:GO:00
0	86.5	3	P:GO:0000272; F:GO:0016161; F:GO:0102229
1.04E-138	79.87	1	C:GO:0016021
1.12E-112	94.67	6	F:GO:0005524; P:GO:0006457; P:GO:0009408; F:GO:00

3.05E-37	89.83	5	F:GO:0000977; F:GO:0003700; C:GO:0005634;
4.57E-24	84.57	1	C:GO:0016021
6.01E-153	80.79		
4.97E-115	77.51		
0	79.16	9	F:GO:0004573; C:GO:0005634; P:GO:0005975; P:GO:00
1.14E-37	97.1	7	P:GO:0000209; F:GO:0005524; C:GO:0005737; P:GO:00
5.97E-119	95.98	3	C:GO:0005634; P:GO:0009691; F:GO:0016787
0	85.15	2	F:GO:0102560; F:GO:0102561
5.50E-57	75.37	2	C:GO:0009507; C:GO:0009522
0	94.19	2	F:GO:0005524; F:GO:0016887
0	83.7	2	F:GO:0005524; F:GO:0016887
7.44E-25	88.74	8	P:GO:0001732; P:GO:0002191; F:GO:0003743; C:GO:00
4.05E-37	84.23	5	P:GO:0006486; F:GO:0016757; F:GO:0045543; F:GO:00
0	87.69	5	F:GO:0004825; F:GO:0005524; C:GO:0005739; P:GO:00
8.76E-154	83.51	4	F:GO:0003676; F:GO:0004519; P:GO:0006308; P:GO:00
0	81.44	5	F:GO:0004791; C:GO:0005737; P:GO:0019430; P:GO:00
0	75.76	2	F:GO:0016301; P:GO:0016310
0	66.26	1	F:GO:0003677
0	72.6	1	F:GO:0003677
2.91E-80	64.04	2	F:GO:0003677; F:GO:0046872
1.34E-69	66.91	3	F:GO:0003677; C:GO:0005634; F:GO:0008270
7.68E-116	70.92	5	F:GO:0003700; C:GO:0005634; P:GO:0006351; P:GO:00
0	73.81	2	P:GO:0006355; F:GO:0140110
0	73.81	2	P:GO:0006355; F:GO:0140110
2.73E-138	94.55	4	F:GO:0004674; F:GO:0005509; F:GO:0005524; P:GO:00
0	81.84	2	F:GO:0003677; F:GO:0046983
8.80E-23	74.13		
0	78.5		
0	69.58	3	P:GO:0006629; F:GO:0016787; P:GO:0044237
4.52E-130	84.89	2	F:GO:0008270; F:GO:0016874
0	80.75	2	C:GO:0000795; P:GO:0007131
6.36E-99	82.72	5	C:GO:0005769; C:GO:0005886; F:GO:0015095;
1.09E-35	87.2	5	C:GO:0005769; C:GO:0005886; F:GO:0015095;
1.08E-177	90.86	5	F:GO:0004712; F:GO:0005524; C:GO:0005622; P:GO:00
0	76.68	1	C:GO:0016020
0	83.28	1	F:GO:0016746
0	83.82	7	F:GO:0004672; F:GO:0005524; P:GO:0006468; P:GO:00
1.97E-101	89.48	3	F:GO:0030941; C:GO:0031359; P:GO:0045036
0	86.11	2	F:GO:0003677; C:GO:0005634
0	88.06	1	C:GO:0016021
6.31E-34	57.1	4	F:GO:0004190; P:GO:0006508; F:GO:0016301; P:GO:00
1.03E-74	92.85	6	F:GO:0005506; C:GO:0005634; C:GO:0005737; F:GO:00
7.68E-79	91.83	5	F:GO:0000287; F:GO:0004743; P:GO:0006096; F:GO:00
0	84.82	9	F:GO:0003677; F:GO:0003910; F:GO:0005524; C:GO:00
0	91.35	1	F:GO:0047172
1.16E-74	94.46	5	C:GO:0000786; C:GO:0000790; F:GO:0003677; P:GO:00
3.04E-74	94.46	5	C:GO:0000786; C:GO:0000790; F:GO:0003677; P:GO:00
2.27E-93	99.86	7	C:GO:0009522; C:GO:0009523; C:GO:0009535; P:GO:00
1.76E-92	99.14	7	C:GO:0009522; C:GO:0009523; C:GO:0009535; P:GO:00

3.00E-76	84.03	5	P:GO:0009744; F:GO:0016301; F:GO:0019887; P:GO:00
0	94.13	1	P:GO:0006897
0	94.1	1	P:GO:0006897
0	85.54	6	F:GO:0003855; F:GO:0004764; P:GO:0009423; C:GO:00
5.88E-174	92.47	4	C:GO:0005743; C:GO:0016021; F:GO:0022857; P:GO:00
0	86.56	3	P:GO:0006470; F:GO:0008138; C:GO:0009507
4.54E-85	63.81	2	C:GO:0016020; C:GO:0016021
7.62E-104	97.76	8	F:GO:0003723; F:GO:0003735; C:GO:0005634; P:GO:00
0	62.51	3	F:GO:0003677; C:GO:0005634; P:GO:0006355
0	84.44	2	F:GO:0003824; F:GO:0050662
0	84.72	2	F:GO:0003824; F:GO:0050662
0	81.06	2	P:GO:0009725; C:GO:0016021
0	76.36		
0	89.16	9	C:GO:0005774; C:GO:0005886; P:GO:0006885; P:GO:00
0	73.62	5	F:GO:0003677; F:GO:0003700; C:GO:0005634; P:GO:00
7.05E-78	91.4	1	F:GO:0008270
0	86.69	3	C:GO:0016021; F:GO:0022857; P:GO:0055085
7.14E-115	94.92	5	F:GO:0004623; F:GO:0042171; P:GO:0055089; P:GO:00
0	87.25	4	P:GO:0006855; F:GO:0015238; F:GO:0015297; C:GO:00
0	87.31	4	P:GO:0006855; F:GO:0015238; F:GO:0015297; C:GO:00
0	86.25		
2.52E-108	82.8	4	P:GO:0000105; F:GO:0004400; C:GO:0009507; F:GO:00
3.55E-146	90.52	6	F:GO:0005506; C:GO:0016021; F:GO:0016709; F:GO:00
0	88.62	6	F:GO:0003942; C:GO:0005737; P:GO:0006526; F:GO:00
0	95.95	2	C:GO:0005886; P:GO:0007186
4.26E-67	74.25	1	F:GO:0003723
9.56E-17	97.62		
1.43E-177	85.02	3	F:GO:0004527; C:GO:0016021; P:GO:0090305
2.74E-147	76.4		
0	92.32	4	F:GO:0004693; F:GO:0005524; P:GO:0006468; P:GO:00
2.51E-20	82.94	3	F:GO:0004482; P:GO:0006370; P:GO:0106005
0	91.99	3	F:GO:0005089; F:GO:0016301; P:GO:0016310
0	70.37		
0	75.35		
0	75.65		
2.49E-37	50.17	4	F:GO:0003676; F:GO:0003743; P:GO:0006413; P:GO:00
0	89.49	6	C:GO:0005886; C:GO:0016021; F:GO:0016760; P:GO:00
3.35E-175	83.76		
0	90.04	4	C:GO:0000148; F:GO:0003843; P:GO:0006075; C:GO:00
8.72E-21	77.83	2	P:GO:0006950; P:GO:0009415
0	84.41		
0	73.88		
3.24E-67	90.05	3	F:GO:0004022; F:GO:0008270; P:GO:0055114
1.23E-135	74.85		
0	65.17	1	P:GO:0006396
0	85.24	5	P:GO:0010228; F:GO:0042800; P:GO:0048440; P:GO:00
6.49E-101	89.01	6	C:GO:0000139; F:GO:0004571; F:GO:0005509; C:GO:00
0	91.28	8	C:GO:0000139; F:GO:0004571; F:GO:0005509; C:GO:00

1.68E-63	63.28		
1.85E-55	70.31		
3.49E-138	63.24		
2.53E-173	88.61	2	F:GO:0008253; P:GO:0016311
1.51E-138	63.5	8	C:GO:0005622; P:GO:0006807; P:GO:0006950; P:GO:0(
0	78.32	2	P:GO:0009725; P:GO:0050794
4.12E-77	89.02	1	C:GO:0005886
0	58.91	3	F:GO:0003677; C:GO:0005634; P:GO:0006355
0	93.47	7	F:GO:0000049; F:GO:0004813; F:GO:0005524; C:GO:00
3.27E-97	87.18	3	P:GO:0005975; P:GO:0016311; F:GO:0050278
5.52E-71	84.76	3	P:GO:0005975; P:GO:0016311; F:GO:0050278
2.35E-16	100		
0	92.86	7	F:GO:0005506; C:GO:0005789; C:GO:0016021; P:GO:0(
9.15E-167	92.74	7	F:GO:0005506; C:GO:0005789; C:GO:0016021; P:GO:0(
4.53E-67	96.81	4	F:GO:0003743; P:GO:0006413; C:GO:0009534; P:GO:0(
0	65.88		
1.58E-147	86.16	7	F:GO:0004420; C:GO:0005789; P:GO:0008299; P:GO:0(
2.11E-171	91.57	4	F:GO:0005047; F:GO:0005525; C:GO:0005785; C:GO:0(
0	70.54	2	F:GO:0003677; C:GO:0005634
0	70.54	2	F:GO:0003677; C:GO:0005634
0	95.16		
9.21E-71	99.21		
6.76E-90	71.69		
0	76.89		
2.05E-116	81.27	3	F:GO:0003677; C:GO:0005634; P:GO:0006355
0	79.37	1	F:GO:0003723
4.41E-56	80.18	1	F:GO:0003677
0	91.72	4	C:GO:0000139; P:GO:0006891; P:GO:0015031; C:GO:0(
0	76.8	1	F:GO:0016779
0	87.27	7	C:GO:0000325; F:GO:0005524; C:GO:0005774;
2.44E-85	88.81	7	P:GO:0006021; F:GO:0008934; P:GO:0046854; P:GO:0(
0	81.17	2	F:GO:0004190; P:GO:0006508
0	79.06	3	F:GO:0004190; P:GO:0006508; P:GO:0030163
0	82.62		
0	83.98	2	F:GO:0003677; C:GO:0005634
1.13E-121	93.05	5	C:GO:0000139; C:GO:0016021; P:GO:0045489; F:GO:0(
1.64E-39	90.36	2	C:GO:0005576; C:GO:0005618
0	73.17	4	C:GO:0000784; F:GO:0003677; F:GO:0003691; P:GO:00
2.91E-18	93.43	6	C:GO:0000276; F:GO:0003743; P:GO:0006413;
0	84		
2.76E-145	83.16	1	C:GO:0005634
1.44E-172	83.8	1	C:GO:0005634
0	82.7	1	C:GO:0005634
0	71.34	2	F:GO:0003677; C:GO:0005634
1.15E-124	82.87	1	F:GO:0003723
4.28E-79	70.12	1	P:GO:2000026
3.28E-29	62.64	2	C:GO:0016020; C:GO:0016021
0	85.85	2	P:GO:0006355; F:GO:0008270

1.56E-123	74.48		
0	89.5		
0	89.5		
0	72.38		
0	81.97	6	F:GO:0004497; F:GO:0005506; C:GO:0016021; F:GO:00
0	85.16	9	F:GO:0003723; F:GO:0004647; F:GO:0008420; P:GO:00
0	82.22		
0	65.21		
0	92.88	3	C:GO:0005634; C:GO:0005737; P:GO:0015031
1.79E-161	66.81	3	F:GO:0003676; F:GO:0003723; F:GO:0008270
0	80.33	3	F:GO:0000989; C:GO:0005634; P:GO:0006355
5.86E-73	85.16		
0	74.06	2	F:GO:0003700; P:GO:0006355
0	93.71	8	F:GO:0005524; C:GO:0005739; C:GO:0009507;
1.66E-151	82.04		
3.83E-82	88.09		
0	82.58	5	C:GO:0005783; P:GO:0006081; C:GO:0016021; F:GO:00
0	72.24	1	F:GO:0046983
8.93E-152	74.7	1	F:GO:0046983
0	71.65	1	F:GO:0046983
0	87.53	1	P:GO:0006355
0	91.91	8	P:GO:0001676; F:GO:0004467; C:GO:0005783;
3.42E-39	92.57	1	C:GO:0016021
0	74.35	1	F:GO:0016740
0	79.41	4	F:GO:0004386; F:GO:0005524; F:GO:0016874; F:GO:00
3.04E-90	73.2	1	C:GO:0016020
2.23E-09	89.19		
4.32E-179	89.63	4	P:GO:0002084; F:GO:0004622; C:GO:0005737; F:GO:00
0	80.4	4	F:GO:0004556; F:GO:0005509; P:GO:0005975; F:GO:01
0	84.99	5	F:GO:0005524; C:GO:0005886; C:GO:0016021; F:GO:00
0	75.59	2	C:GO:0016020; C:GO:0016021
0	80.16	3	F:GO:0005247; C:GO:0016021; P:GO:1902476
0	93.99	4	F:GO:0003677; F:GO:0003899; P:GO:0006351; C:GO:00
0	95.82	5	F:GO:0004827; F:GO:0005524; C:GO:0005737; P:GO:00
0	88.68	4	P:GO:0006855; F:GO:0015238; F:GO:0015297; C:GO:00
3.02E-114	82.65	5	C:GO:0005737; P:GO:0006629; C:GO:0016021; F:GO:00
1.18E-116	72.15	4	C:GO:0016020; C:GO:0016021; F:GO:0016874; F:GO:00
0	93.39	4	C:GO:0000139; F:GO:0015165; C:GO:0016021; P:GO:00
4.16E-128	76.76	1	F:GO:0016787
5.36E-38	98.56	2	F:GO:0004332; P:GO:0006096
2.02E-112	98.94	2	F:GO:0004332; P:GO:0006096

0	88.38	5	F:GO:0003975; C:GO:0005789; P:GO:0006489; F:GO:00
0	84.09	1	C:GO:0016021
2.82E-120	79.77	7	F:GO:0004364; P:GO:0006749; P:GO:0006952;
4.71E-115	70.38	6	P:GO:0006950; C:GO:0009507; P:GO:0009628; P:GO:00
2.46E-167	69.13	1	C:GO:0009536
3.73E-16	83.18	1	C:GO:0016020
1.72E-122	66.17	8	F:GO:0008270; C:GO:0016020; C:GO:0016021; P:GO:00
4.98E-138	85.17		
0	82.04		
1.62E-112	60.04		
0	90.09	3	P:GO:0006486; C:GO:0016021; F:GO:0016757
1.48E-143	90.9	6	F:GO:0003712; F:GO:0004402; C:GO:0005634;
9.56E-120	94.33	1	F:GO:0003723
0	92.7	4	P:GO:0000055; C:GO:0005634; P:GO:0030036; P:GO:00
3.86E-136	94.8	9	F:GO:0004362; C:GO:0005623; P:GO:0006749;
2.42E-31	83.39	6	F:GO:0003723; F:GO:0004004; F:GO:0005524; C:GO:00
0	91.68	4	P:GO:0005975; C:GO:0009505; C:GO:0016021; F:GO:00
5.67E-103	93.76	2	F:GO:0004518; P:GO:0090305
0	88.17		
4.64E-47	63.93	3	F:GO:0000166; F:GO:0005524; P:GO:0051301
0	77.7	2	C:GO:0005794; C:GO:0016021
0	79.84	2	C:GO:0016020; F:GO:0016757
1.09E-16	89.76		
1.73E-41	75.54	1	P:GO:0006950
4.06E-180	79.32		
0	88.59	4	P:GO:0000719; F:GO:0003904; P:GO:0009650; F:GO:00
0	90.41	4	P:GO:0000719; F:GO:0003904; P:GO:0009650; F:GO:00
4.42E-47	78.46	2	F:GO:0016787; P:GO:0044237
0	90.95	3	P:GO:0008152; C:GO:0016021; F:GO:0034480
0	92.08	4	F:GO:0004674; F:GO:0005524; P:GO:0006468; P:GO:00
0	76.86	2	P:GO:0009791; P:GO:0048608
4.49E-114	56.92	9	F:GO:0003676; F:GO:0004672; P:GO:0006468; F:GO:00
0	85.37		
0	92.77	11	F:GO:0005524; C:GO:0005739; C:GO:0005774; C:GO:00
0	89.75	5	F:GO:0005524; C:GO:0005886; C:GO:0016021; F:GO:00
0	84.61	1	C:GO:0016021
5.97E-69	74.74	4	F:GO:0003677; F:GO:0003700; C:GO:0005634; P:GO:00
0	86.11	10	F:GO:0003873; F:GO:0005524; C:GO:0005829; C:GO:00
0	96.52	3	F:GO:0003676; F:GO:0004386; F:GO:0005524
0	73.75		
1.49E-43	85.49	1	C:GO:0019028
7.64E-74	69.75		
2.98E-61	67.12		
6.25E-43	73.99	9	F:GO:0003723; F:GO:0004519; C:GO:0005634; P:GO:00
0	93.77	7	F:GO:0005506; P:GO:0006537; F:GO:0010181; F:GO:00
5.98E-52	81.25	6	F:GO:0005506; C:GO:0016021; F:GO:0016709; F:GO:00
0	89.91	6	F:GO:0005506; C:GO:0016021; F:GO:0016709; F:GO:00
3.46E-128	94.25	3	C:GO:0016021; F:GO:0022857; P:GO:0055085
3.70E-69	89.65	6	C:GO:0005737; P:GO:0009813; F:GO:0031418; F:GO:00
0	90.94	7	C:GO:0005737; F:GO:0031418; F:GO:0045431; F:GO:00

1.80E-69	80.89	2	P:GO:0006979; P:GO:0009061
0	78.07	1	F:GO:0005524
0	72.77	1	F:GO:0003677
3.26E-77	89.2	1	C:GO:0016021
6.64E-108	83.74	3	C:GO:0016021; F:GO:0022857; P:GO:0055085
3.58E-149	99.07	7	C:GO:0009522; C:GO:0009523; C:GO:0009535;
4.57E-149	82.44	6	F:GO:0004326; F:GO:0005524; C:GO:0005737; P:GO:00
0	70.83		
3.71E-45	83.65		
3.62E-125	90.18	8	F:GO:0004127; F:GO:0005524; C:GO:0005634; C:GO:00
1.98E-54	84.81	1	C:GO:0016020
0	89.74	1	C:GO:0016021
0	90.06	7	P:GO:0000302; F:GO:0004130; F:GO:0020037; P:GO:00
4.30E-158	60.75	2	C:GO:0005622; F:GO:0008270
3.36E-157	60.73	2	C:GO:0005622; F:GO:0008270
1.01E-142	60.93	2	C:GO:0005622; F:GO:0008270
4.51E-61	80.6		
4.55E-81	97.11	6	F:GO:0004478; F:GO:0005524;
9.60E-87	99.44	6	F:GO:0004478; F:GO:0005524; C:GO:0005829;
3.83E-24	87.06	2	C:GO:0016021; F:GO:0016874
9.20E-61	94.42	2	F:GO:0004519; P:GO:0090305
0	81.66		
1.17E-122	87.72	3	C:GO:0009507; P:GO:0016311; F:GO:0050124
1.13E-44	94.88	11	C:GO:0000307; P:GO:0007346; F:GO:0016301;
0	90.9	1	C:GO:0016021
0	84.03		
1.35E-100	94.91	1	C:GO:0016021
6.98E-27	92.33	7	F:GO:0003735; C:GO:0005762; P:GO:0006412; C:GO:00
3.48E-92	88.94	3	F:GO:0003735; C:GO:0005840; P:GO:0006412
0	84.16	5	F:GO:0004559; P:GO:0006013; P:GO:0006517; F:GO:00
3.24E-159	92.11	3	C:GO:0005829; P:GO:0006508; F:GO:0016920
3.58E-170	76.21	4	C:GO:0005634; F:GO:0008270; P:GO:0009416; P:GO:00
0	85.66	2	F:GO:0004185; P:GO:0051603
0	84	3	F:GO:0004185; P:GO:0006508; C:GO:0016021
1.03E-151	79.23	3	C:GO:0005634; P:GO:0006355; P:GO:0009734
1.13E-85	94.56	2	F:GO:0003954; P:GO:0022900
0	83.71	3	F:GO:0003841; P:GO:0008654; C:GO:0016021
5.95E-39	88.8	2	C:GO:0000502; P:GO:0043248
0	88.43	6	F:GO:0005524; F:GO:0015623; P:GO:0015688; C:GO:00
0	83.51	6	F:GO:0005524; F:GO:0015623; P:GO:0015688; C:GO:00
0	93.97	5	F:GO:0000049; F:GO:0004825; F:GO:0005524; C:GO:00
0	98.64	10	F:GO:0005524; C:GO:0005739; C:GO:0005829; P:GO:00
1.61E-91	97.13	2	F:GO:0003824; F:GO:0050662
0	94.56		
0	87.46		
0	79.58	2	P:GO:0006368; C:GO:0070449
8.26E-57	50.52	2	C:GO:0016020; C:GO:0016021
0	80.11	3	C:GO:0005779; F:GO:0008270; P:GO:0016558

0	75.89	3	C:GO:0005886; C:GO:0016021; P:GO:0055085
5.44E-71	95.32	3	F:GO:0015267; C:GO:0016021; P:GO:0055085
9.80E-132	94.72	3	F:GO:0015267; C:GO:0016021; P:GO:0055085
0	93.08	4	P:GO:0005985; F:GO:0016157; F:GO:0046524; F:GO:00
9.25E-89	74.43	1	F:GO:0016757
1.79E-144	83.54	4	F:GO:0008168; P:GO:0032259; P:GO:0055114; F:GO:01
0	86.13	5	F:GO:0005507; P:GO:0046274; C:GO:0048046; F:GO:00
0	65.27	2	C:GO:0009507; P:GO:0010150
5.54E-178	95.77	12	P:GO:0009416; C:GO:0009522; C:GO:0009523; C:GO:00
2.80E-128	97.18	7	C:GO:0009522; C:GO:0009523; C:GO:0009535;
1.56E-134	66.12	3	C:GO:0005622; C:GO:0005634; F:GO:0008270
0	91.07	6	F:GO:0004601; F:GO:0005509; C:GO:0016021; F:GO:00
1.88E-152	93.57	3	F:GO:0003983; C:GO:0005737; P:GO:0006011
6.74E-173	100	13	F:GO:0004176; F:GO:0004222; F:GO:0005524; C:GO:00
0	93.54	4	P:GO:0005975; F:GO:0008422; C:GO:0016021; P:GO:19
0	92.31	6	C:GO:0009507; F:GO:0009540; P:GO:0009688;
1.96E-94	82.65	8	C:GO:0009535; F:GO:0009540; P:GO:0009688; C:GO:00
4.76E-118	94.57	6	C:GO:0009507; F:GO:0009540; P:GO:0009688; C:GO:00
0	75.11	1	F:GO:0046983
0	77.08	1	F:GO:0046983
0	82.78	1	C:GO:0016021
6.27E-34	86.87	7	F:GO:0003677; C:GO:0005634; F:GO:0008270;
1.92E-106	81.96	4	F:GO:0003677; C:GO:0005634; F:GO:0008270; P:GO:00
2.44E-44	96.62	4	F:GO:0003677; C:GO:0005634; F:GO:0008270; P:GO:00
0	70.76	2	F:GO:0005488; C:GO:0016020
3.02E-51	88.58	4	F:GO:0003857; C:GO:0005777; P:GO:0006635; C:GO:00
0	92.95	3	F:GO:0005524; C:GO:0016021; F:GO:0016787
0	82.08	2	F:GO:0005524; C:GO:0016020
2.47E-74	91.63		
3.33E-98	77.73	1	P:GO:0007165
1.24E-128	73.04	4	F:GO:0003677; C:GO:0005634; P:GO:0006351; P:GO:00
0	87.12	4	C:GO:0005819; C:GO:0005874; P:GO:0032147; P:GO:00
6.61E-177	96.77		
5.84E-44	94.94	5	C:GO:0005634; C:GO:0005829; P:GO:0051131; F:GO:00
3.23E-44	91.24		
0	99.05	2	F:GO:0004332; P:GO:0006096
2.93E-166	82.01	1	C:GO:0016021
2.00E-58	91.29	3	C:GO:0016021; F:GO:0016491; P:GO:0055114
6.13E-148	79.2	2	F:GO:0003993; P:GO:0016311
2.46E-132	78.4	2	F:GO:0003993; P:GO:0016311
0	81.19	2	F:GO:0003993; P:GO:0016311
0	79.29	1	F:GO:0043167
0	81.61	2	F:GO:0005524; F:GO:0008270
1.02E-54	81.04	2	F:GO:0003700; P:GO:0006355
0	86.82	2	F:GO:0033728; P:GO:0055114
0	92.42	1	F:GO:0005524
0	91.1	1	F:GO:0005524
1.08E-106	80.8		

0	73.34	3	C:GO:0005622; P:GO:0006886; P:GO:0016192
0	86.91	5	P:GO:0006486; C:GO:0016021; C:GO:0032580; P:GO:00
0	88.84	4	F:GO:0008728; P:GO:0015969; F:GO:0016301; P:GO:00
0	77.36		
3.54E-126	84.95	1	F:GO:0016788
1.03E-122	84.99	1	F:GO:0016788
0	90.64	3	P:GO:0006508; F:GO:0008233; C:GO:0016021
8.86E-71	90.32	5	C:GO:0000786; C:GO:0005634; P:GO:0006334; F:GO:00
3.31E-85	81.22		
3.86E-146	81.44		
0	88.24	1	C:GO:0016021
0	65.32	5	F:GO:0003676; F:GO:0004523; C:GO:0016020; C:GO:00
2.84E-144	79.63	1	F:GO:0003723
1.99E-161	85.29	1	F:GO:0003676
0	65.7		
0	63.83		
0	83.79		
2.84E-16	76.32		
2.83E-88	78.93	3	F:GO:0003677; C:GO:0005634; P:GO:0006355
0	77.88		
2.07E-118	83.7	2	C:GO:0005737; C:GO:0005886
2.54E-44	84.61		
0	80.89	2	C:GO:0016021; F:GO:0035091
1.02E-146	73.88		
1.72E-79	87.89		
2.09E-77	93.1	1	C:GO:0016021
4.75E-156	72.12	4	F:GO:0003677; C:GO:0005634; P:GO:0006351; P:GO:00
9.43E-87	92.73	5	F:GO:0003677; C:GO:0005634; P:GO:0006355; P:GO:00
9.23E-76	87.55	1	C:GO:0016021
0	86.17	8	F:GO:0004674; F:GO:0005524; C:GO:0005829; P:GO:00
0	82.12	5	C:GO:0005783; P:GO:0009058; C:GO:0016021; F:GO:00
1.04E-84	91.83		
0	90.66	2	F:GO:0004084; P:GO:0009081
3.37E-71	87.23	4	C:GO:0005737; P:GO:0030001; F:GO:0046914; P:GO:00
1.50E-36	95.83	4	C:GO:0005737; P:GO:0030001; F:GO:0046914; P:GO:00
0	80.56		
3.54E-97	83.5	4	F:GO:0003677; F:GO:0003700; C:GO:0005634; P:GO:00
1.63E-15	54.96		
0	64.87		
5.01E-131	91.42	2	C:GO:0009507; F:GO:0048037
0	78.53	1	C:GO:0016021
0	87.15	2	C:GO:0005783; C:GO:0005794
1.18E-134	96.69	11	P:GO:0009416; C:GO:0009522; C:GO:0009523;
0	88.83	6	F:GO:0003756; C:GO:0005789; C:GO:0016021; F:GO:00
8.61E-97	80.34	3	F:GO:0004017; F:GO:0005524; P:GO:0046939
7.18E-132	85.01	2	C:GO:0005622; F:GO:0008270
5.73E-127	74.54	2	C:GO:0005622; F:GO:0008270
4.82E-85	81.58	1	F:GO:0003723
3.93E-96	92.23	2	C:GO:0005789; C:GO:0016021

2.88E-153	85.4	6	F:GO:0005506; C:GO:0016021; F:GO:0016711; F:GO:00
1.17E-83	90.06		
6.88E-81	89.92		
1.48E-13	92.05	1	C:GO:0016021
0	90.6	1	F:GO:0005524
0	96.31	1	F:GO:0005524
0	89.72	1	F:GO:0005524
2.75E-133	74.59	6	F:GO:0000166; F:GO:0004674; C:GO:0005634; C:GO:00
0	86.06	4	F:GO:0003723; F:GO:0003724; F:GO:0005524; P:GO:00
0	93.21		
0	74.21		
0	91.51	7	F:GO:0003677; F:GO:0003910; F:GO:0005524; P:GO:00
3.68E-64	99.34	4	C:GO:0000786; F:GO:0003677; C:GO:0005634; F:GO:00
0	88.36	2	C:GO:0005777; F:GO:0030600
8.70E-168	89.11	3	F:GO:0015079; C:GO:0016021; P:GO:0071805
0	86.9	3	F:GO:0015079; C:GO:0016021; P:GO:0071805
2.35E-23	64.59		
2.12E-86	92.64	3	P:GO:0005975; F:GO:0030246; F:GO:0047938
6.60E-94	93.61	3	P:GO:0005975; F:GO:0030246; F:GO:0047938
0	90.7	2	P:GO:0000226; F:GO:0008017
3.67E-163	87.04	3	C:GO:0005622; P:GO:0006625; P:GO:0009733
7.95E-70	90.54	3	C:GO:0016021; P:GO:0046373; F:GO:0046556
0	81.58	1	C:GO:0016021
9.16E-124	79.82	3	F:GO:0003677; C:GO:0005634; F:GO:0008270
2.81E-144	76.26	1	F:GO:0008233
0	88.16	4	P:GO:0006508; F:GO:0046872; F:GO:0070006; P:GO:00
0	63.76	2	F:GO:0003676; F:GO:0003723
0	82.55		
0	81.46		
1.46E-112	94.96	6	F:GO:0005524; P:GO:0006457; P:GO:0009408; F:GO:00
0	96.5	5	C:GO:0005634; P:GO:0006886; P:GO:0006913; F:GO:00
0	98.76	5	F:GO:0005524; P:GO:0005978; F:GO:0008878; C:GO:00
0	87.22	6	F:GO:0004693; F:GO:0005524; C:GO:0005634; C:GO:00
0	79.48	2	P:GO:0008152; F:GO:0052689
0	79.68	1	F:GO:0046983
0	71.94	4	F:GO:0003677; C:GO:0005634; P:GO:0006351; P:GO:00
5.15E-10	69.2	1	P:GO:0008152
3.57E-16	77.97		
0	91.33	4	C:GO:0000148; F:GO:0003843; P:GO:0006075; C:GO:00
0	93.05	4	C:GO:0000148; F:GO:0003843; P:GO:0006075; C:GO:00
0	77.79	1	P:GO:0006979
0	86.76	5	F:GO:0005249; C:GO:0005887; P:GO:0034765; P:GO:00
4.32E-51	87.18		
1.93E-106	62.79	5	F:GO:0003677; F:GO:0003700; C:GO:0005634; P:GO:00
2.30E-91	86.06	1	F:GO:0016787
5.17E-90	83.4	1	C:GO:0016020
2.38E-146	72.21	2	F:GO:0003677; P:GO:0006355

5.95E-98	70.15		
4.74E-104	71.13		
7.06E-160	63.23	2	C:GO:0005622; F:GO:0008270
2.06E-141	95.32	4	P:GO:0000162; F:GO:0004048; C:GO:0009570; C:GO:00
6.95E-61	81.33	1	C:GO:0016021
0	91.77	6	C:GO:0005829; P:GO:0006099; F:GO:0008964; P:GO:00
0	70.89	5	F:GO:0001046; F:GO:0001228; C:GO:0005634; P:GO:00
0	76.89	3	C:GO:0005737; C:GO:0016020; F:GO:0055105
0	93.53	1	F:GO:0005524
0	87.42	8	C:GO:0005783; C:GO:0005886; P:GO:0009926; P:GO:00
7.92E-31	96.66	3	P:GO:0009734; C:GO:0016021; P:GO:0055085
0	72.39	2	P:GO:0006796; F:GO:0016787
0	84.16	3	F:GO:0000287; F:GO:0004737; F:GO:0030976
4.16E-91	84.81		
1.47E-17	85.14	5	P:GO:0000413; F:GO:0003755; C:GO:0005768; C:GO:00
3.48E-101	86.55	2	F:GO:0003824; P:GO:0009116
0	65.97		
0	65.41	3	C:GO:0005622; C:GO:0005634; F:GO:0008270
0	84.23	3	F:GO:0008195; C:GO:0016021; P:GO:0016311
1.97E-77	91.8		
2.57E-54	99.2	4	C:GO:0000786; F:GO:0003677; C:GO:0005634; F:GO:00
5.40E-109	88.75		
9.32E-99	75.3	7	C:GO:0005758; F:GO:0009055; P:GO:0016226; P:GO:00
0	79.57	6	F:GO:0000155; P:GO:0000160; F:GO:0005524; C:GO:00
1.06E-33	98.84	6	C:GO:0000786; F:GO:0003677; C:GO:0005634; P:GO:00
0	91.88	2	P:GO:0008152; F:GO:0016874
3.13E-94	88.14	3	F:GO:0003824; C:GO:0016021; F:GO:0050662
7.98E-46	89.45	3	F:GO:0003824; P:GO:0009116; C:GO:0016021
9.72E-81	62.99	4	F:GO:0003824; P:GO:0009116; C:GO:0016020; C:GO:00
4.38E-138	83.35	2	F:GO:0003824; P:GO:0009116
0	78.05	1	C:GO:0016021
1.70E-29	95.6	2	F:GO:0004325; P:GO:0006783
0	86.87	11	P:GO:0000302; F:GO:0003746; F:GO:0003924; F:GO:00
5.05E-152	94.72	2	F:GO:0003924; F:GO:0005525
0	74.02		
1.27E-83	70.16	1	P:GO:0050896
0	86.66		
0	68.9	5	C:GO:0009507; C:GO:0009570; P:GO:0009658; F:GO:00
2.08E-69	96.68	4	F:GO:0005524; P:GO:0005975; F:GO:0008974; P:GO:00
0	90.47	1	C:GO:0016021
0	92.91	1	C:GO:0016021
0	90.68	1	C:GO:0016021
1.63E-119	87.83	3	C:GO:0005773; C:GO:0009506; C:GO:0016021
0	87.98	5	F:GO:0004610; C:GO:0005829; P:GO:0005975; P:GO:00
1.94E-176	94.34	11	P:GO:0009416; C:GO:0009522; C:GO:0009523; C:GO:00
0	77.92	5	C:GO:0009536; F:GO:0010277; C:GO:0016021; F:GO:00
0	77.73	9	P:GO:0001666; F:GO:0003677; P:GO:0006355; P:GO:00
0	75.36		
4.97E-34	97.67	3	P:GO:0006221; P:GO:0007049; P:GO:0045747
3.56E-50	68.33	1	F:GO:0003723

0	70.16	1	F:GO:0003677
0	62.47	1	F:GO:0043531
0	85.88	4	C:GO:0005622; P:GO:0006886; F:GO:0017112; P:GO:00
0	88.97		
0	89.5		
2.70E-90	95.91	1	F:GO:0003723
1.43E-90	95.91	1	F:GO:0003723
1.12E-111	76.45	3	F:GO:0004864; C:GO:0005737; P:GO:0035308
1.49E-40	67.48	4	P:GO:0006651; F:GO:0008195; C:GO:0009507; C:GO:00
9.92E-44	98.21		
7.68E-84	88.96	3	C:GO:0005743; C:GO:0016021; P:GO:0055085
3.74E-69	92.8	6	F:GO:0003824; P:GO:0005975; C:GO:0009535; P:GO:00
3.24E-122	77.39	4	F:GO:0003954; C:GO:0005886; C:GO:0016021; F:GO:00
1.10E-154	79.05	3	C:GO:0016021; F:GO:0022857; P:GO:0055085
0	82.9	4	F:GO:0003677; C:GO:0005634; C:GO:0016021; F:GO:00
0	83.5	4	F:GO:0003677; C:GO:0005634; C:GO:0016021; F:GO:00
4.17E-10	53.95	7	C:GO:0005737; P:GO:0005975; F:GO:0009045; C:GO:00
0	89.84		
4.58E-82	94.69	5	C:GO:0005634; P:GO:0009631; F:GO:0042393; P:GO:00
1.05E-180	93.71		
2.39E-73	96.99		
4.75E-59	94.57		
3.15E-62	94.64		
0	86.85		
0	91.56	1	F:GO:0003824
0	89.05	1	F:GO:0003824
0	97	2	F:GO:0008886; P:GO:0055114
0	79.08	1	F:GO:0003723
0	62.93	1	F:GO:0016874
0	84.97	2	F:GO:0003676; F:GO:0046872
1.46E-131	84.52	2	C:GO:0016021; P:GO:0031047
0	81.6	2	F:GO:0003677; C:GO:0005634
0	84.1	3	F:GO:0003987; P:GO:0008152; C:GO:0016021
2.92E-159	79.74	3	C:GO:0016020; F:GO:0016491; F:GO:0046872
0	82.38	6	F:GO:0004497; F:GO:0005506; C:GO:0016021; F:GO:00
1.74E-86	87.51	6	F:GO:0004497; F:GO:0005506; C:GO:0016021; F:GO:00
0	82.49	6	F:GO:0004497; F:GO:0005506; C:GO:0016021; F:GO:00
0	71.45	4	F:GO:0005506; F:GO:0016705; F:GO:0020037; P:GO:00
2.81E-24	82.3	6	F:GO:0004497; F:GO:0005506; C:GO:0016021; F:GO:00
0	68.54	4	F:GO:0005506; F:GO:0016705; F:GO:0020037; P:GO:00
5.53E-161	90.8	5	F:GO:0005524; P:GO:0006457; F:GO:0032440; F:GO:00
7.73E-113	96.97	11	C:GO:0005773; C:GO:0005777; P:GO:0005975; P:GO:00
0	97.43	11	C:GO:0005773; C:GO:0005777; P:GO:0005975; P:GO:00
0	97.39	4	F:GO:0008465; F:GO:0016618; F:GO:0051287; P:GO:00
7.37E-53	98.44	4	F:GO:0008465; F:GO:0016618; F:GO:0051287; P:GO:00
0	99.63	2	P:GO:0015031; C:GO:0016021
6.71E-83	93.5	1	F:GO:0003729
2.26E-101	89.64	7	C:GO:0000243; P:GO:0000398; F:GO:0003729; C:GO:00

1.16E-101	89.64	7	C:GO:0000243; P:GO:0000398; F:GO:0003729; C:GO:0(
0	95.1	5	F:GO:0005507; F:GO:0008131; P:GO:0009308; F:GO:00
8.13E-15	94.36		
2.32E-133	85.33	5	F:GO:0005524; P:GO:0005978; F:GO:0008878; C:GO:00
1.93E-134	86.08	5	F:GO:0005524; P:GO:0005978; F:GO:0008878; C:GO:00
5.29E-42	92.46	3	F:GO:0008483; P:GO:0009058; F:GO:0030170
0	83.6	4	F:GO:0004672; F:GO:0005524; P:GO:0006468; C:GO:00
1.59E-152	79.59	3	C:GO:0016021; C:GO:0043231; C:GO:0044444
0	81.56	4	F:GO:0003677; C:GO:0005634; P:GO:0006355; P:GO:0(
0	91.07	4	C:GO:0000148; F:GO:0003843; P:GO:0006075; C:GO:0(
0	91.07	4	C:GO:0000148; F:GO:0003843; P:GO:0006075; C:GO:0(
4.18E-110	94.46	3	F:GO:0015267; C:GO:0016021; P:GO:0055085
0	71.43	1	F:GO:0005488
0	81.36	2	F:GO:0003676; F:GO:0008270
0	87.06	2	P:GO:0006355; F:GO:0008270
3.24E-118	95.55	3	F:GO:0004672; F:GO:0005524; P:GO:0006468
0	86.44	3	P:GO:0006508; F:GO:0046872; F:GO:0070006
0	96.41	2	P:GO:0015995; F:GO:0016851
0	90.42	7	C:GO:0005829; C:GO:0005886; P:GO:0005985; C:GO:0(
0	88.86	5	F:GO:0004672; F:GO:0005524; P:GO:0006468; C:GO:00
0	83.83	5	F:GO:0005524; F:GO:0015440; C:GO:0016021; P:GO:00
2.64E-159	85.03	5	P:GO:0000398; C:GO:0005681; F:GO:0030628; F:GO:00
7.23E-42	85.02		
1.04E-160	92.76	2	P:GO:0006661; C:GO:0070772
0	76.29	3	F:GO:0004252; P:GO:0006508; C:GO:0016020
0	89.43	2	P:GO:0000413; F:GO:0003755
1.33E-127	89.2	2	C:GO:0005794; C:GO:0016021
0	93.43	9	C:GO:0005801; C:GO:0005829; P:GO:0006886; P:GO:0(
0	89.55	5	F:GO:0000030; C:GO:0005789; P:GO:0006487; C:GO:0(
0	94.31	8	C:GO:0000932; F:GO:0003729; C:GO:0005634; P:GO:0(
6.52E-83	95	5	F:GO:0003723; C:GO:0005634; P:GO:0006397; F:GO:00
1.01E-80	90.57	1	C:GO:0016021
3.27E-70	90.44	1	C:GO:0016021
0	91.88	8	F:GO:0003743; C:GO:0005634; C:GO:0005737; P:GO:0(
4.88E-123	87.32	3	C:GO:0005829; F:GO:0008757; P:GO:0032259
0	91.82	6	F:GO:0000049; F:GO:0004826; F:GO:0005524; C:GO:00
3.60E-101	94.2	2	C:GO:0016021; F:GO:0030246
2.16E-80	93.3	1	C:GO:0016021
0	87.89	6	C:GO:0005829; F:GO:0008374; F:GO:0008970; P:GO:00
6.33E-96	91.05	3	F:GO:0003677; C:GO:0005634; F:GO:0008289
8.14E-122	70.54		
4.66E-129	74.11	3	F:GO:0005488; P:GO:0008152; F:GO:0016491
0	89.44	6	F:GO:0004675; F:GO:0005524; C:GO:0005886; P:GO:00
5.47E-12	80.18	6	F:GO:0005524; C:GO:0005886; F:GO:0008553; C:GO:00
2.53E-149	96.09	4	F:GO:0004375; C:GO:0005739; P:GO:0006546; P:GO:0(
2.98E-61	97.11	5	F:GO:0004375; C:GO:0005739; C:GO:0005960; P:GO:0(
3.90E-155	83.42	1	F:GO:0016740
8.05E-29	82.55	1	F:GO:0004645
0	94	5	F:GO:0003746; F:GO:0003924; F:GO:0005525; C:GO:00

0	95.13	5	F:GO:0003746; F:GO:0003924; F:GO:0005525; C:GO:00
1.15E-109	91.21	2	F:GO:0005215; C:GO:0016021
0	85.63	3	F:GO:0004190; P:GO:0006508; C:GO:0016021
0	80.65	3	F:GO:0008324; C:GO:0016021; P:GO:0098655
0	67.18	1	F:GO:0043531
0	67.18	1	F:GO:0043531
0	84.24	12	F:GO:0003700; C:GO:0005739; P:GO:0009553; P:GO:00
3.99E-172	93.87	12	F:GO:0003700; C:GO:0005739; P:GO:0009553; P:GO:00
0	73.66	4	F:GO:0003779; C:GO:0016020; C:GO:0016021; F:GO:00
0	77.33		
0	83.23	1	F:GO:0051087
1.30E-48	99.65	12	C:GO:0000786; F:GO:0003677; C:GO:0005730; C:GO:00
0	87.99	7	F:GO:0005524; C:GO:0005886; F:GO:0015412; P:GO:00
1.54E-158	84.56	5	F:GO:0000989; C:GO:0005634; P:GO:0006355; F:GO:00
6.28E-176	71.3	1	F:GO:0016874
7.36E-105	99.46	19	F:GO:0004601; F:GO:0005344; F:GO:0005506; C:GO:00
2.15E-44	86.7		
0	69.09		
0	88.23	20	P:GO:0000226; C:GO:0005802; C:GO:0005829; P:GO:00
0	94.61		
1.47E-87	95.37	2	C:GO:0009507; C:GO:0016021
0	73.85	2	F:GO:0003676; F:GO:0046872
0	79.06	4	F:GO:0004674; F:GO:0005524; P:GO:0006468; C:GO:00
0	87.85	6	F:GO:0003723; F:GO:0004519; C:GO:0005739; F:GO:00
1.19E-173	98.19	5	F:GO:0004298; C:GO:0005634; C:GO:0005737; P:GO:00
2.18E-167	97	4	P:GO:0009809; P:GO:0032259; F:GO:0042409; F:GO:00
0	86.24	3	F:GO:0016787; F:GO:0047268; F:GO:0047274
0	96.13	2	F:GO:0004252; P:GO:0006508
9.26E-70	87.04	4	C:GO:0005634; F:GO:0008270; P:GO:0009416; P:GO:00
0	83.27	3	F:GO:0003863; C:GO:0005739; P:GO:0055114
7.67E-26	83.18	4	F:GO:0003677; F:GO:0003700; P:GO:0006355; C:GO:00
5.28E-138	97.35	2	C:GO:0000813; P:GO:0043328
8.78E-138	97.35	2	C:GO:0000813; P:GO:0043328
0	82.1	1	F:GO:0016787
5.99E-98	92.61	10	P:GO:0000045; C:GO:0005634; C:GO:0005829; P:GO:00
0	88.12	4	P:GO:0006855; F:GO:0015238; F:GO:0015297; C:GO:00
2.00E-84	82.39	3	F:GO:0004674; F:GO:0005524; P:GO:0006468
0	92.31	4	F:GO:0004674; F:GO:0004715; F:GO:0005524; P:GO:00
6.31E-93	65.18		
3.98E-115	87.83	4	C:GO:0005886; C:GO:0016021; P:GO:0034219; F:GO:00
0	79.67	1	C:GO:0016021
0	59.34		
0	80.98	2	C:GO:0005829; P:GO:0042147
0	81.22	6	C:GO:0000938; C:GO:0005829; P:GO:0006896; C:GO:00
0	89.08	2	F:GO:0003677; F:GO:0046983
0	84.74	2	F:GO:0003677; F:GO:0046983
0	87.26	2	F:GO:0003677; F:GO:0046983
0	75.14	3	F:GO:0003677; C:GO:0016020; P:GO:0065007

0	76.09	2	F:GO:0003677; C:GO:0016020
2.80E-146	96.21		
0	89.9	6	F:GO:0004372; F:GO:0008168; P:GO:0019264; F:GO:00
4.38E-14	94.38		
0	66.18		
0	83.29	4	F:GO:0003989; P:GO:0006633; C:GO:0009317; F:GO:00
0	78.98	4	F:GO:0004725; F:GO:0008138; P:GO:0035335; F:GO:00
0	84.5	5	F:GO:0005524; C:GO:0005886; C:GO:0016021; F:GO:00
1.34E-60	83.45	2	F:GO:0003690; P:GO:0006355
1.06E-14	96.67		
0	83.21	4	C:GO:0000220; F:GO:0015078; P:GO:0015991; C:GO:00
0	95.97	3	F:GO:0004722; P:GO:0006470; F:GO:0046872
0	95.7	3	F:GO:0004722; P:GO:0006470; F:GO:0046872
0	87.3		
3.02E-109	85.64		
1.25E-71	88.73		
0	97.89	6	C:GO:0009507; P:GO:0019288; F:GO:0046872; P:GO:00
4.15E-104	87.73	6	F:GO:0005388; F:GO:0005516; F:GO:0005524; C:GO:00
0	65.14		
0	76.24	2	P:GO:0009657; C:GO:0016020
1.13E-82	93.82	7	F:GO:0009496; P:GO:0015979; C:GO:0016021; P:GO:00
1.00E-66	72.42		
3.18E-65	70.03		
1.38E-47	92.02	3	F:GO:0004672; F:GO:0005524; P:GO:0006468
0	79.86	2	F:GO:0003677; C:GO:0005634
0	86.28	7	C:GO:0005773; P:GO:0006891; P:GO:0007030; F:GO:00
0	88.49	7	F:GO:0005524; F:GO:0015419; P:GO:0015709; C:GO:00
0	90.6	3	F:GO:0004672; F:GO:0005524; P:GO:0006468
6.14E-132	61.87	2	C:GO:0005622; F:GO:0008270
7.64E-70	88.29	4	F:GO:0005507; F:GO:0009055; C:GO:0009535; P:GO:00
2.92E-64	88.38	4	F:GO:0005507; F:GO:0009055; C:GO:0009535; P:GO:00
3.88E-64	88.54	4	F:GO:0005507; F:GO:0009055; C:GO:0009535; P:GO:00
2.30E-59	91.88	4	F:GO:0005507; F:GO:0009055; C:GO:0009535; P:GO:00
4.66E-150	90.92	2	P:GO:0046373; F:GO:0046556
1.23E-31	83.44	3	F:GO:0004672; F:GO:0005524; P:GO:0006468
0	75.63	1	F:GO:0003723
0	82.55	9	F:GO:0003684; F:GO:0004519; F:GO:0005524; P:GO:00
8.24E-168	78.48	4	F:GO:0003723; F:GO:0004519; P:GO:0009451; C:GO:00
0	96.27	6	F:GO:0005524; C:GO:0005886; F:GO:0008553; C:GO:00
0	96.19	3	F:GO:0005524; P:GO:0006508; F:GO:0008233
5.39E-145	92.7	6	P:GO:0005975; F:GO:0016906; P:GO:0030259; F:GO:01
0	70.99	1	P:GO:0006355
0	90.27	6	F:GO:0005506; P:GO:0009809; C:GO:0016021; F:GO:00
0	92.57	6	F:GO:0004497; F:GO:0005506; C:GO:0016021; F:GO:00
3.82E-97	86.49	6	F:GO:0004497; F:GO:0005506; C:GO:0016021; F:GO:00
0	82.99	9	F:GO:0004176; F:GO:0004252; F:GO:0005524; C:GO:00
5.29E-157	80.28	1	F:GO:0003676
3.71E-47	94.52	5	F:GO:0003746; C:GO:0005840; C:GO:0005853; P:GO:00
7.60E-87	73.56	1	F:GO:0016874

5.76E-75	88.45	5	P:GO:0006664; C:GO:0012505; C:GO:0016021; F:GO:00
5.94E-113	65.56	1	F:GO:0005509
4.09E-126	87.81	11	F:GO:0005509; F:GO:0005544; P:GO:0009408; P:GO:00
4.08E-170	95.89	6	C:GO:0005634; P:GO:0006511; P:GO:0007275;
1.77E-179	97.63	1	C:GO:0016021
0	97.91	1	C:GO:0016021
0	97.91	1	C:GO:0016021
5.33E-83	72.34	1	F:GO:0016874
5.40E-52	57.51	7	F:GO:0003824; P:GO:0006807; P:GO:0043170; P:GO:00
0	82.79	1	F:GO:0016787
2.86E-116	83.91	1	F:GO:0016787
0	96.34	5	F:GO:0004351; F:GO:0005516; P:GO:0006536; C:GO:00
0	93.04	3	F:GO:0004674; F:GO:0005524; P:GO:0006468
5.27E-19	98.68		
2.48E-65	67.47		
0	89.86		
2.72E-67	91	10	P:GO:0000422; C:GO:0005829; P:GO:0006497; C:GO:00
0	68.66	3	F:GO:0003676; P:GO:0010468; P:GO:0031123
0	86.65	7	F:GO:0005096; C:GO:0005622; P:GO:0006886; C:GO:00
1.26E-56	94.28	6	F:GO:0004347; C:GO:0005829; P:GO:0006094; P:GO:00
0	86.47	2	F:GO:0004252; P:GO:0006508
6.24E-81	88.35	1	C:GO:0044599
2.05E-50	80.2		
1.17E-57	76.39	2	C:GO:0009535; P:GO:0055085
0	85.63	3	P:GO:0005975; F:GO:0010303; F:GO:0051060
2.89E-46	82.38	3	F:GO:0003677; C:GO:0005634; P:GO:0006355
0	70.14		
1.57E-13	100		
1.25E-67	91.52	2	F:GO:0003723; C:GO:0016021
3.65E-166	76.8	5	C:GO:0009507; F:GO:0015095; C:GO:0016021; P:GO:00
9.43E-173	83.2		
0	65.27	4	C:GO:0016020; P:GO:0016070; F:GO:0016301; P:GO:00
0	81.31		
8.81E-124	70.95	3	F:GO:0003677; C:GO:0005634; P:GO:0006355
5.77E-131	71.66	3	F:GO:0003677; C:GO:0005634; P:GO:0006355
0	70.56	4	F:GO:0003677; C:GO:0005634; P:GO:0006351; P:GO:00
0	71.35	4	F:GO:0003677; C:GO:0005634; P:GO:0006351; P:GO:00
4.82E-155	67.56	4	F:GO:0003677; C:GO:0005634; P:GO:0006351; P:GO:00
3.97E-69	99.03	3	F:GO:0010181; F:GO:0016491; P:GO:0055114
0	68.93	2	C:GO:0005634; C:GO:0009534
3.49E-155	91.59	5	F:GO:0003844; F:GO:0004553; P:GO:0005978; F:GO:00
4.11E-169	59.46	2	C:GO:0005622; F:GO:0008270
0	93.96	7	F:GO:0003777; F:GO:0005524; C:GO:0005871;
2.48E-22	93.91	3	F:GO:0010181; F:GO:0016491; P:GO:0055114
2.81E-27	93.41	6	F:GO:0010181; C:GO:0016021; F:GO:0052852;
9.36E-84	98.35	3	F:GO:0010181; F:GO:0016491; P:GO:0055114

4.41E-63	74.72	4	C:GO:0005802; C:GO:0016021; P:GO:0030148; F:GO:00
0	83.17	11	P:GO:0001732; P:GO:0002188; F:GO:0003729; F:GO:00
3.38E-95	81.6	11	P:GO:0001732; P:GO:0002188; F:GO:0003729; F:GO:00
1.04E-90	80.68		
5.07E-53	75.39		
0	90.73	1	P:GO:0006904
3.18E-149	95.56	4	F:GO:0004674; F:GO:0005524; P:GO:0006468; C:GO:00
1.87E-94	89.85	2	P:GO:0000967; F:GO:0016787
2.56E-78	94.37	9	P:GO:0000413; F:GO:0003755; C:GO:0005783; C:GO:00
3.87E-142	87.66		
0	91.74	2	C:GO:0016021; F:GO:0042578
3.84E-106	76.7	1	P:GO:0050896
3.82E-72	71.73		
3.19E-71	79.52	4	C:GO:0005794; C:GO:0016021; F:GO:0016413; P:GO:00
0	79.14	7	F:GO:0003677; F:GO:0003779; F:GO:0003899; P:GO:00
7.13E-84	98.62	2	F:GO:0005524; F:GO:0016740
0	84.74	3	F:GO:0004089; F:GO:0008270; P:GO:0015976
1.55E-129	76.98	4	F:GO:0003677; F:GO:0003700; C:GO:0005634; P:GO:00
0	78.54		
0	85.88		
8.21E-59	84.07		
5.61E-57	84.07		
1.00E-164	92.88	5	F:GO:0003723; F:GO:0005525; C:GO:0005739; C:GO:00
9.64E-115	86.34		
1.50E-99	76.77		
0	91.62	4	F:GO:0003677; C:GO:0005634; P:GO:0006355; P:GO:00
0	80.05	4	F:GO:0003677; C:GO:0005634; P:GO:0006355; P:GO:00
0	80.47	6	F:GO:0003677; F:GO:0003700; C:GO:0005634; C:GO:00
0	79.17	2	F:GO:0005488; C:GO:0044424
0	58.51	8	F:GO:0003676; C:GO:0005634; C:GO:0005737; P:GO:00
3.76E-72	86.74	1	F:GO:0003723
6.43E-102	65.14		
7.81E-74	91.88	2	P:GO:0006012; F:GO:0047216
0	90.1	9	P:GO:0006012; P:GO:0009408; P:GO:0009409; P:GO:00
0	83.5	4	P:GO:0000027; F:GO:0005524; C:GO:0005634; F:GO:00
2.05E-151	92.09	3	F:GO:0015079; C:GO:0016021; P:GO:0071805
0	57.03		
0	64.98	3	F:GO:0003677; C:GO:0005634; P:GO:0010228
0	71.97	2	C:GO:0005634; P:GO:0006355
5.51E-64	81.36	10	F:GO:0004129; C:GO:0005743; P:GO:0006119; P:GO:00
0	55.76	1	P:GO:0010099
1.56E-48	92.89	1	F:GO:0016787
0	89.02	5	F:GO:0004252; C:GO:0005737; P:GO:0006508; C:GO:00
0	98.18	4	P:GO:0006357; C:GO:0016592; F:GO:0047918; F:GO:00
3.75E-90	98.92	3	F:GO:0003735; P:GO:0006412; C:GO:0022627
0	75.8	2	P:GO:0006914; C:GO:1990316
1.97E-33	95.35	3	F:GO:0004721; P:GO:0006470; F:GO:0046872
0	79.83	4	F:GO:0003730; C:GO:0005829; F:GO:0046872; P:GO:00

2.69E-139	80.3	2	F:GO:0016491; P:GO:0055114
0	89.38	5	P:GO:0000160; F:GO:0003677; F:GO:0003700; C:GO:00
4.42E-94	82.1	3	C:GO:0016021; F:GO:0016491; P:GO:0055114
0	73.69	1	P:GO:0006629
0	82.45	3	C:GO:0005622; P:GO:0006623; F:GO:0030276
6.70E-162	97.48	9	F:GO:0004674; F:GO:0004712; F:GO:0004715; F:GO:00
5.75E-134	63.08	2	F:GO:0003676; F:GO:0003723
0	85.05	1	F:GO:0035091
5.03E-35	62.49		
5.04E-172	82.21	1	F:GO:0000225
6.33E-157	87.72	5	C:GO:0005576; C:GO:0005773; P:GO:0006952; P:GO:00
7.72E-118	89.35	5	C:GO:0005576; C:GO:0005773; P:GO:0006952; P:GO:00
0	96.26	4	F:GO:0005524; P:GO:0007049; F:GO:0016787; P:GO:00
7.09E-149	85.44	2	C:GO:0005789; C:GO:0016021
0	76.31	1	F:GO:0016787
0	76.31	1	F:GO:0016787
3.95E-57	73.98	2	C:GO:0016020; C:GO:0016021
1.16E-55	74.59		
0	68.12	5	P:GO:0000160; C:GO:0005622; C:GO:0005634; F:GO:00
0	65.38	5	P:GO:0000160; C:GO:0005622; C:GO:0005634; F:GO:00
1.69E-50	99.15	12	C:GO:0000786; F:GO:0003677; C:GO:0005730; C:GO:00
0	80.03		
0	81.56		
0	89.4		
4.37E-135	66.98		
0	88.42	3	P:GO:0006511; C:GO:0031461; F:GO:0031625
0	62.54		
9.06E-66	91.98		
0	69.69	1	P:GO:0016180
0	81.83	4	P:GO:0006913; P:GO:0006999; F:GO:0017056; C:GO:00
5.75E-150	93.19	9	F:GO:0003677; F:GO:0003743; C:GO:0005634; P:GO:00
0	88.11	3	F:GO:0005507; F:GO:0047705; P:GO:0055114
0	88.11	3	F:GO:0005507; F:GO:0047705; P:GO:0055114
0	77.45		
0	82.4		
0	74.21		
0	93.01	3	F:GO:0005247; C:GO:0016021; P:GO:1902476
0	91.9	3	F:GO:0005247; C:GO:0016021; P:GO:1902476
0	97.21	4	F:GO:0005524; P:GO:0006457; P:GO:0006950; F:GO:00
5.18E-137	79.15	3	C:GO:0005622; P:GO:0006886; P:GO:0016192
0	83.87	4	F:GO:0004575; C:GO:0005775; P:GO:0005985; C:GO:00
0	83.37	4	F:GO:0004575; C:GO:0005775; P:GO:0005975; C:GO:00
0	84.61	7	F:GO:0003676; C:GO:0005634; F:GO:0008168; P:GO:00
3.01E-115	91.74	4	F:GO:0003723; F:GO:0016301; P:GO:0016310; F:GO:00
1.01E-29	85.32	7	P:GO:0000160; C:GO:0005634; C:GO:0005737; P:GO:00
0	66.03	6	F:GO:0003676; P:GO:0010468; P:GO:0016070; P:GO:00
0	70.2	1	F:GO:0046872
0	91.96		

0	86.41	1	C:GO:0016021
8.51E-105	99.19	3	F:GO:0004842; F:GO:0005524; P:GO:0016567
6.65E-38	81.79	2	P:GO:0030001; F:GO:0046872
6.78E-87	78.56	4	F:GO:0004674; F:GO:0005524; P:GO:0006468; C:GO:00
2.47E-100	82.54	7	F:GO:0003723; F:GO:0003735; C:GO:0005840; P:GO:00
0	93.73	3	F:GO:0005524; P:GO:0006457; F:GO:0051082
0	92.99	3	F:GO:0005524; P:GO:0006457; F:GO:0051082
0	79.65	2	C:GO:0016021; F:GO:0016757
0	81.65	2	C:GO:0016021; F:GO:0016757
4.10E-86	66.1		
0	70.59	6	F:GO:0000166; P:GO:0016043; F:GO:0016307; P:GO:00
2.21E-135	59.57	1	F:GO:0016740
4.10E-55	93.42		
4.31E-155	84.95		
4.13E-128	85.15		
2.22E-142	54.91	1	F:GO:0046872
5.78E-80	53.46	1	F:GO:0046872
0	91.94	2	C:GO:0016021; P:GO:0055085
9.92E-105	97.52		
0	82.21	2	C:GO:0016021; C:GO:0031969
0	76.97	2	C:GO:0000326; P:GO:0048316
3.48E-106	83.31	5	P:GO:0000028; F:GO:0004386; P:GO:0006364; C:GO:00
2.40E-159	92.16	4	C:GO:0005576; C:GO:0005618; P:GO:0009664; C:GO:00
0	66.44	4	F:GO:0003723; F:GO:0004519; P:GO:0009451; C:GO:00
6.78E-114	92.9	6	F:GO:0003854; P:GO:0006694; C:GO:0016021; F:GO:00
1.12E-60	95.97	4	C:GO:0005829; F:GO:0016621; F:GO:0050662; P:GO:00
0	72.58		
0	87.78	2	P:GO:0005975; F:GO:0042973
3.73E-137	79.51		
0	69.68	1	F:GO:0051087
9.58E-78	81.46	6	F:GO:0009055; C:GO:0009507; P:GO:0009643; P:GO:00
1.96E-89	85.57	5	F:GO:0009055; C:GO:0009507; P:GO:0022900; F:GO:00
2.62E-164	70.41		
0	90.16	7	F:GO:0003677; F:GO:0003910; F:GO:0005524; P:GO:00
0	77.45	3	C:GO:0005737; P:GO:0016192; P:GO:0042594
6.82E-180	96.05	2	C:GO:0005634; C:GO:0005737
2.16E-106	83.56	4	P:GO:0009247; C:GO:0009941; P:GO:0046506; F:GO:00
0	90.11	4	F:GO:0003995; P:GO:0033539; P:GO:0048767; F:GO:00
0	90.73	6	F:GO:0003995; F:GO:0004672; P:GO:0006468; P:GO:00
0	96.34	4	F:GO:0005545; C:GO:0030136; F:GO:0030276; P:GO:00
0	95.46	4	F:GO:0005545; C:GO:0030136; F:GO:0030276; P:GO:00
0	63.32	4	F:GO:0003677; C:GO:0005634; P:GO:0006351; P:GO:00
0	73.19	4	F:GO:0003677; C:GO:0005634; P:GO:0006351; P:GO:00
0	81.52	1	C:GO:0016021
3.51E-86	85.94	8	F:GO:0004674; F:GO:0005524; C:GO:0005634; C:GO:00
1.22E-56	80.01	3	F:GO:0004674; F:GO:0005524; P:GO:0006468
0	60.82		

1.55E-101	77.2		
6.26E-56	78.13	4	C:GO:0009523; C:GO:0009535; C:GO:0016021; P:GO:00
0	80.65	6	F:GO:0004497; F:GO:0005506; C:GO:0016021; F:GO:00
3.55E-145	87.71		
2.60E-16	100		
2.62E-143	89.86	3	F:GO:0004722; P:GO:0006470; F:GO:0046872
1.66E-117	84.34	7	P:GO:0000462; C:GO:0005654; C:GO:0005730; P:GO:00
5.94E-119	93.86	1	F:GO:0030600
0	88.56	8	P:GO:0006809; F:GO:0009703; F:GO:0020037; F:GO:00
2.73E-156	76.08	1	C:GO:0016020
0	89.4	5	C:GO:0005743; P:GO:0006839; C:GO:0016021; F:GO:00
0	88.54		
0	89.94		
1.54E-119	90.13		
0	68.61	8	F:GO:0003677; F:GO:0003700; F:GO:0005524; C:GO:00
3.13E-26	99.15	8	C:GO:0009522; C:GO:0009523; C:GO:0009535; P:GO:00
0	89.51	6	P:GO:0006979; P:GO:0009414; C:GO:0009507; P:GO:00
1.26E-80	94.51	2	F:GO:0003676; C:GO:0005840
0	80.91	4	P:GO:0006400; C:GO:0016021; F:GO:0051539; F:GO:00
0	88.74		
5.56E-122	92.91	3	F:GO:0004324; C:GO:0009507; P:GO:0055114
0	90.05	8	C:GO:0005829; P:GO:0005986; P:GO:0006000; P:GO:00
2.94E-85	93.44	13	C:GO:0005634; C:GO:0005829; P:GO:0005983; P:GO:00
1.43E-169	89.05	3	P:GO:0005975; P:GO:0016311; F:GO:0042132
0	89.56	2	P:GO:0006355; F:GO:0046872
2.00E-93	84.2	4	C:GO:0009523; P:GO:0015979; C:GO:0016021; F:GO:00
4.08E-80	94.87	8	F:GO:0000155; P:GO:0000160; F:GO:0004674; F:GO:00
7.88E-151	76.06		
3.65E-134	72.26		
4.30E-64	95.26		
1.23E-55	87.25		
1.90E-101	93.81		
0	73.11	4	F:GO:0004672; C:GO:0005737; P:GO:0006468; P:GO:00
3.56E-99	64.77		
0	65.04		
6.16E-49	94.84	3	F:GO:0003723; P:GO:0009644; P:GO:0043484
4.91E-47	93.85	3	F:GO:0003723; P:GO:0009644; P:GO:0043484
0	86.33	2	P:GO:0006364; C:GO:0016021
7.66E-160	85.47	2	P:GO:0008152; F:GO:0050403
1.29E-132	98.45	1	F:GO:0003677
1.40E-49	87.36	1	C:GO:0016021
0	82.96	1	C:GO:0016021
0	88.49		
0	88.21	4	F:GO:0004371; F:GO:0005524; P:GO:0006071; P:GO:00
0	90.24	9	F:GO:0000175; C:GO:0000178; C:GO:0000932; C:GO:00
0	83.96	7	F:GO:0000175; C:GO:0005739; P:GO:0006364; F:GO:00
1.49E-118	84.45	2	C:GO:0009507; C:GO:0016021
2.01E-31	97.6	2	C:GO:0009507; C:GO:0016021
0	91.66	3	F:GO:0003723; C:GO:0005634; P:GO:0006397

7.78E-128	94.19	3	C:GO:0016021; F:GO:0022857; P:GO:0055085
0	76.69	4	F:GO:0003951; P:GO:0006741; P:GO:0016310; P:GO:00
0	96.06	2	P:GO:0002098; F:GO:0050660
2.90E-173	84.65	3	F:GO:0004672; F:GO:0005524; P:GO:0006468
6.98E-171	91.71	6	F:GO:0004697; F:GO:0005524; C:GO:0005634; C:GO:00
0	72.65		
0	54.36		
0	97.42	4	F:GO:0004760; C:GO:0005777; F:GO:0008453; P:GO:00
3.38E-169	97.07	4	F:GO:0004760; C:GO:0005777; F:GO:0008453; P:GO:00
0	78.95	1	C:GO:0016021
1.89E-58	87.7	3	P:GO:0003333; F:GO:0015171; C:GO:0016021
0	94.29	10	F:GO:0004066; F:GO:0005524; P:GO:0006529; P:GO:00
1.57E-166	97.88	10	F:GO:0004066; F:GO:0005524; P:GO:0006529; P:GO:00
0	96.85	10	F:GO:0004066; F:GO:0005524; P:GO:0006529; P:GO:00
2.90E-121	85.88	8	F:GO:0005351; F:GO:0005355; F:GO:0005366; C:GO:00
0	91.52		
3.76E-52	89.7	1	F:GO:0003723
0	77.8		
0	77.8		
0	77.14	5	P:GO:0000390; F:GO:0000978; F:GO:0001078; P:GO:00
0	76.8	5	P:GO:0000390; F:GO:0000978; F:GO:0001078; P:GO:00
0	95.45	3	F:GO:0000234; C:GO:0016021; P:GO:0032259
1.12E-124	86.23	2	C:GO:0016021; P:GO:0055085
0	89.42	2	C:GO:0016021; F:GO:0016788
0	88.31	1	C:GO:0016021
6.05E-136	71.13		
1.27E-42	91.14	3	P:GO:0009247; C:GO:0016021; F:GO:0016758
4.37E-56	84.54	3	F:GO:0003677; F:GO:0003682; C:GO:0005634
0	88.61	2	F:GO:0004084; P:GO:0009081
0	85.68	5	F:GO:0004722; C:GO:0005634; P:GO:0006470; P:GO:00
0	90.84	2	C:GO:0016021; F:GO:0047186
6.35E-83	68.37		
0	77.41		
0	82.29	3	F:GO:0046872; F:GO:0050590; P:GO:0055114
0	84.05	3	F:GO:0000234; C:GO:0016021; P:GO:0032259
0	88.99	5	C:GO:0005743; P:GO:0006839; C:GO:0016021; F:GO:00
1.58E-140	92.93	8	F:GO:0005543; C:GO:0005829; P:GO:0006606;
1.25E-140	92.93	8	F:GO:0005543; C:GO:0005829; P:GO:0006606;
2.17E-177	85.67	2	F:GO:0008289; C:GO:0016021
7.89E-58	62.17	3	F:GO:0008289; C:GO:0016020; C:GO:0016021
0	61.82	6	F:GO:0003743; C:GO:0005669; P:GO:0006413;
0	92.07	3	F:GO:0003743; P:GO:0006413; P:GO:0006417
6.34E-37	76.35	2	C:GO:0016021; P:GO:0033554
2.80E-91	87.34	4	F:GO:0003677; F:GO:0005524; P:GO:0006281; F:GO:00
0	86.82	1	F:GO:0003860
1.09E-172	71.25	1	C:GO:0005634
3.80E-99	98.67	6	F:GO:0004478; F:GO:0005524; C:GO:0005829; P:GO:00
2.82E-93	90.08	1	C:GO:0005576
0	74.89	4	F:GO:0003677; F:GO:0003824; P:GO:0006284; P:GO:00
0	86.65	1	C:GO:0016021

0	83.26	5	F:GO:0004568; C:GO:0005576; P:GO:0005975; P:GO:0C
2.20E-135	94.3		
3.30E-135	94.3		
0	88.88	6	C:GO:0005739; C:GO:0005777; P:GO:0006979; F:GO:0C
2.56E-167	96.14	5	P:GO:0008643; C:GO:0030173; C:GO:0030176; F:GO:0C
2.82E-29	85.9		
0	86.32	4	F:GO:0004445; P:GO:0046856; F:GO:0052658; F:GO:00
0	61.48	2	F:GO:0003700; P:GO:0006355
5.02E-131	63.86		
0	93.69	5	F:GO:0005524; P:GO:0006855; F:GO:0008559; C:GO:00
1.51E-67	59.44	3	F:GO:0003676; C:GO:0016020; C:GO:0016021
0	83.12	2	F:GO:0005089; P:GO:0080092
2.98E-62	79.25		
8.82E-177	87.88	2	F:GO:0047061; P:GO:0055114
1.32E-33	100		
2.75E-66	90.72	4	C:GO:0005886; P:GO:0006979; P:GO:0009555; C:GO:0C
0	80.71	4	F:GO:0004674; F:GO:0005524; C:GO:0005886; P:GO:00
0	91.78	4	F:GO:0004514; P:GO:0009435; C:GO:0009507; P:GO:0C
0	82.99	6	F:GO:0005506; C:GO:0016021; F:GO:0016709; F:GO:00
0	89.2	3	P:GO:0006541; P:GO:0009396; F:GO:0046820
6.93E-125	61.36	2	F:GO:0004518; P:GO:0009987
0	79.2	1	F:GO:0003676
0	89.81		
0	81.57		
2.70E-177	68.75	1	F:GO:0016740
0	91.5	6	F:GO:0005509; C:GO:0005794; P:GO:0006004; C:GO:0C
0	89.01		
4.67E-97	89.24	5	C:GO:0005623; P:GO:0006662; F:GO:0015035; P:GO:0C
0	87.15	2	F:GO:0005215; C:GO:0016021
0	87.86	3	F:GO:0005524; F:GO:0016308; P:GO:0046854
4.59E-91	86.47	2	C:GO:0046658; P:GO:0051716
0	96.32	3	F:GO:0004674; F:GO:0005524; P:GO:0006468
1.01E-148	91.38	1	C:GO:0016021
0	74.29	1	F:GO:0003676
2.55E-146	88.12	5	F:GO:0003700; C:GO:0005634; P:GO:0006355; F:GO:00
0	84.53		
3.68E-35	76.66		
4.08E-51	97.24	1	F:GO:0003723
1.96E-180	88.12	3	F:GO:0003743; P:GO:0006413; F:GO:0016853
4.74E-162	79.12		
1.01E-52	94.83	2	F:GO:0005525; C:GO:0005622
0	85.01	3	P:GO:0000462; C:GO:0005730; C:GO:0032040
2.40E-82	74.73	9	F:GO:0004791; C:GO:0005737; P:GO:0008152; F:GO:00
0	84.46		
0	81.81		
0	77.63	4	F:GO:0004674; F:GO:0005524; P:GO:0018105; P:GO:00

0	88.57	4	F:GO:0004672; F:GO:0005524; P:GO:0006468; P:GO:00
0	90.07	3	C:GO:0005887; F:GO:0008271; P:GO:1902358
1.55E-27	71.2		
9.85E-37	62.88		
0	96.76	4	C:GO:0000148; F:GO:0003843; P:GO:0006075; C:GO:00
6.52E-157	90.51	6	F:GO:0003677; F:GO:0003887; P:GO:0006271; P:GO:00
0	84.5	6	P:GO:0000012; F:GO:0003690; F:GO:0003697; C:GO:00
1.73E-56	68.59	4	C:GO:0009941; P:GO:0010020; C:GO:0042651; P:GO:00
0	90.76	8	C:GO:0005829; C:GO:0005886; P:GO:0006508;
2.07E-35	99.26	3	P:GO:0007034; C:GO:0010008; P:GO:0070676
0	74.47		
1.02E-95	64.51	7	F:GO:0003677; F:GO:0003700; C:GO:0005634; P:GO:00
0	91.11	6	F:GO:0031491; P:GO:0032968; P:GO:0034724; C:GO:00
0	91.11	6	F:GO:0031491; P:GO:0032968; P:GO:0034724; C:GO:00
9.34E-144	89.68	9	F:GO:0003684; C:GO:0005634; C:GO:0005829; P:GO:00
0	87.87	3	F:GO:0003987; P:GO:0008152; C:GO:0016021
0	84.32		
4.48E-127	84.91	1	C:GO:0016021
0	71.55	4	F:GO:0003677; C:GO:0005634; P:GO:0006351; P:GO:00
2.32E-30	85.96	1	P:GO:0006869
2.66E-90	69.74		
2.90E-144	82.56	3	F:GO:0004712; F:GO:0005524; P:GO:0006468
4.63E-30	80.9		
0	70.61		
0	95.16	2	P:GO:0006596; F:GO:0050126
2.90E-108	93.72	2	P:GO:0033388; F:GO:0050126
0	77.29	1	F:GO:0003677
7.71E-175	91.7	4	P:GO:0003333; C:GO:0005886; F:GO:0015171; C:GO:00
0	84.53	4	F:GO:0004674; F:GO:0005524; P:GO:0006468; C:GO:00
0	74.88	2	P:GO:0008152; F:GO:0016758
0	87.21	2	F:GO:0003824; P:GO:0008152
0	82.56	5	F:GO:0005506; P:GO:0008610; C:GO:0016021; F:GO:00
3.39E-38	83.11	4	F:GO:0005507; C:GO:0005623; P:GO:0006878; F:GO:00
0	91.92	7	C:GO:0005886; F:GO:0015172; F:GO:0015175; P:GO:00
0	87.89	7	C:GO:0005886; F:GO:0015172; F:GO:0015175; P:GO:00
1.50E-123	90.59	2	F:GO:0008375; C:GO:0016021
1.23E-28	90.25	8	C:GO:0005886; C:GO:0009705; F:GO:0015112; P:GO:00
6.29E-32	66.82	1	F:GO:0016874
0	72.43	5	F:GO:0003677; C:GO:0005634; P:GO:0006351; P:GO:00
0	92.76	4	F:GO:0004674; F:GO:0005524; P:GO:0006468; C:GO:00
3.62E-21	97.87		
0	95.37		
0	61.22	7	F:GO:0003824; P:GO:0006281; P:GO:0006284; P:GO:00
0	80.26	1	C:GO:0016021
8.29E-163	87.84	2	P:GO:0006012; F:GO:0047216
0	97.81	5	F:GO:0000287; F:GO:0004743; P:GO:0006096; F:GO:00
0	91.69	2	P:GO:0016311; F:GO:0050124
1.31E-40	55.14		
1.52E-164	94.87	4	F:GO:0004069; P:GO:0006520; P:GO:0009058; F:GO:00

6.63E-126	70.57	5	F:GO:0005524; C:GO:0005634; P:GO:0006351; P:GO:00
0	80.27	2	F:GO:0003677; C:GO:0005634
0	80.22	2	F:GO:0003677; C:GO:0005634
0	75.72		
0	97.86	16	F:GO:0005524; C:GO:0005618; C:GO:0005739; C:GO:00
0	63.01	1	F:GO:0005488
0	63.02	1	F:GO:0005488
0	63.02	1	F:GO:0005488
3.68E-180	70.03		
0	89.54	4	F:GO:0003677; C:GO:0005634; P:GO:0006355; P:GO:00
0	88.12	3	F:GO:0004843; P:GO:0006511; P:GO:0016579
0	88.04	3	F:GO:0004843; P:GO:0006511; P:GO:0016579
5.30E-92	81.85		
0	84.91		
6.30E-97	82.69	2	F:GO:0000166; F:GO:0003774
0	89.31		
0	89.99		
0	71.82	2	F:GO:0004672; P:GO:0016310
0	91.29	5	C:GO:0005788; C:GO:0005886; C:GO:0016021; F:GO:00
1.53E-135	75.96		
4.84E-179	76.35	2	C:GO:0005623; P:GO:0045454
0	91.2	17	C:GO:0005743; P:GO:0006839; F:GO:0015116; F:GO:00
8.41E-142	79.66	1	F:GO:0003723
0	87.59	6	F:GO:0004674; F:GO:0005524; C:GO:0005622; P:GO:00
1.14E-47	99.19	4	F:GO:0005524; C:GO:0016021; F:GO:0016740; F:GO:00
0	71.76	3	F:GO:0003723; F:GO:0003743; P:GO:0006413
0	90.7	13	F:GO:0004497; F:GO:0005506; P:GO:0009911; P:GO:00
0	86	4	F:GO:0005524; C:GO:0016021; F:GO:0042626; P:GO:00
7.54E-171	82.7	3	F:GO:0003899; C:GO:0005666; P:GO:0006383
1.12E-61	68.67	2	C:GO:0016020; C:GO:0044464
0	85.07	8	P:GO:0000184; P:GO:0000398; F:GO:0003729; F:GO:00
0	85.21	4	C:GO:0005794; C:GO:0016021; F:GO:0016413; P:GO:00
0	84.62	4	C:GO:0005794; C:GO:0016021; F:GO:0016413; P:GO:00
0	84.62	4	C:GO:0005794; C:GO:0016021; F:GO:0016413; P:GO:00
0	89.78	2	F:GO:0003676; C:GO:0005634
0	89.51	3	F:GO:0003676; C:GO:0005634; C:GO:0005829
0	69.24	1	F:GO:0046872
0	65.73	1	F:GO:0003779
7.27E-88	94.04	4	P:GO:0009082; F:GO:0052654; F:GO:0052655; F:GO:00
4.41E-138	87.6	2	F:GO:0005509; C:GO:0016021
0	94.82	4	C:GO:0005887; P:GO:0006833; F:GO:0015250; P:GO:00
0	88.04	6	F:GO:0004222; F:GO:0005524; P:GO:0006508; F:GO:00
0	90.62	2	F:GO:0004535; P:GO:0090503
7.20E-76	93.22	7	F:GO:0005247; F:GO:0009671; C:GO:0009705; P:GO:00
0	80.79	1	F:GO:0003723
0	86.32		
5.23E-160	53.86		

0	81.91		
9.53E-158	67.49	4	C:GO:0005737; P:GO:0030001; F:GO:0046914; P:GO:00
0	67.76		
5.61E-151	87.32	4	F:GO:0005509; C:GO:0009654; P:GO:0015979; C:GO:00
6.12E-121	74.35	2	F:GO:0005515; F:GO:0016740
2.27E-176	86	3	F:GO:0003676; F:GO:0004523; P:GO:0090502
5.86E-42	96.84	1	C:GO:0016021
0	91.04	3	F:GO:0004315; P:GO:0006633; F:GO:0033818
0	79.96	5	F:GO:0003723; F:GO:0004004; F:GO:0005524; C:GO:00
2.01E-151	61.37	4	F:GO:0003677; C:GO:0005634; P:GO:0006351; P:GO:00
6.97E-91	92.91	4	C:GO:0005634; P:GO:0006355; F:GO:0043565; F:GO:00
9.76E-88	95.61	4	C:GO:0005634; P:GO:0006355; F:GO:0043565; F:GO:00
0	89.16	3	F:GO:0003743; P:GO:0006413; F:GO:0016853
5.90E-139	84.62	4	P:GO:0006855; F:GO:0015238; F:GO:0015297; C:GO:00
4.18E-120	87.96		
1.01E-84	67.79	1	F:GO:0046983
2.12E-109	70.93		
6.86E-142	85.02	2	F:GO:0003860; F:GO:0004300
3.52E-21	91.17	7	F:GO:0003924; F:GO:0005515; F:GO:0005525; C:GO:00
2.54E-46	91.99	3	F:GO:0003677; C:GO:0005634; P:GO:0006355
3.96E-65	92.94	5	C:GO:0000786; C:GO:0000790; F:GO:0003677; P:GO:00
1.80E-60	94.44	4	C:GO:0000786; F:GO:0003677; C:GO:0005634; F:GO:00
1.99E-171	87.65	1	F:GO:0003723
2.34E-35	95.28		
0	70.19	1	C:GO:0005739
0	70.19	1	C:GO:0005739
0	70.05	1	C:GO:0005739
0	70.57	1	C:GO:0005739
0	84.91	3	F:GO:0004843; P:GO:0006511; P:GO:0016579
0	94.7	7	F:GO:0003676; F:GO:0004816; F:GO:0005524; C:GO:00
2.63E-142	76.7	2	F:GO:0004672; P:GO:0006468
0	90.71	9	P:GO:0000422; C:GO:0005829; P:GO:0006497; C:GO:00
5.37E-67	84.67		
0	84.16	3	C:GO:0005643; P:GO:0006913; F:GO:0017056
1.67E-38	70.63		
0	60.15		
5.16E-44	88.42		
4.00E-42	87.57		
2.00E-96	54.81	1	C:GO:0016020
0	85.13	5	F:GO:0005524; P:GO:0006457; F:GO:0032440; F:GO:00
0	79.3		
3.34E-117	80.36	2	F:GO:0004425; P:GO:0006568
0	72.99		
0	91.9	3	F:GO:0003964; P:GO:0006278; P:GO:0006397
6.34E-150	82.62	2	F:GO:0016746; F:GO:0016787
9.69E-136	74.19	3	P:GO:0008152; C:GO:0016020; F:GO:0016798
0	76.03	1	P:GO:0016575
0	73.27	1	F:GO:0003824

3.43E-72	92.83	5	P:GO:0000398; C:GO:0005681; F:GO:0030628; F:GO:00
0	89.4	4	F:GO:0004445; P:GO:0046856; F:GO:0052658; F:GO:00
0	87.85	3	P:GO:0006511; P:GO:0016579; F:GO:0036459
0	85.92	6	C:GO:0005768; C:GO:0005802; P:GO:0008643; C:GO:00
0	83.96	4	P:GO:0006004; C:GO:0016021; P:GO:0036066; F:GO:00
5.85E-115	81.79		
0	91.65		
1.16E-160	92.75	4	F:GO:0008483; F:GO:0008696; F:GO:0030170; P:GO:00
0	92.06	1	F:GO:0003914
2.90E-151	91.22	3	P:GO:0009699; F:GO:0050269; P:GO:0055114
4.77E-143	91.05	3	P:GO:0009699; F:GO:0050269; P:GO:0055114
4.48E-150	90.16	6	F:GO:0003697; F:GO:0005524; P:GO:0006281;
3.81E-131	91.04	5	F:GO:0000026; C:GO:0005789; C:GO:0016021; P:GO:00
0	73.31	1	F:GO:0043531
0	76.45	4	F:GO:0003723; C:GO:0005730; C:GO:0016021; P:GO:00
2.39E-133	83.71		
1.36E-157	97.04	3	F:GO:0015267; C:GO:0016021; P:GO:0055085
0	96.9	2	P:GO:0009813; F:GO:0016210
0	69.05	2	F:GO:0003682; C:GO:0009506
0	87.43	2	P:GO:0008152; F:GO:0016874
3.71E-51	61.57	2	P:GO:0006355; C:GO:0016592
2.86E-92	79.06		
0	93.51	12	F:GO:0004471; F:GO:0005524; C:GO:0005759; P:GO:00
0	63.77		
0	76		
0	89.04		
2.65E-51	69.85		
1.13E-56	83.05	1	C:GO:0016020
0	91.64	4	F:GO:0004430; C:GO:0005622; P:GO:0046854; P:GO:00
0	91.64	4	F:GO:0004430; C:GO:0005622; P:GO:0046854; P:GO:00
6.84E-138	98.76	9	C:GO:0005739; P:GO:0005975; P:GO:0006099; P:GO:00
0	78.18		
3.60E-77	91.76	6	C:GO:0005739; F:GO:0009055; C:GO:0009507; P:GO:00
5.29E-20	73.99		
0	82.38	6	F:GO:0003954; F:GO:0003955; C:GO:0005739; C:GO:00
0	75.73		
0	95.88	3	F:GO:0003676; F:GO:0004386; F:GO:0005524
0	88.66	6	F:GO:0001664; F:GO:0003924; F:GO:0005525; C:GO:00
0	78.6	4	F:GO:0004672; F:GO:0005524; P:GO:0006468; C:GO:00
0	90.7	4	F:GO:0005215; P:GO:0006814; C:GO:0016021; P:GO:00
5.24E-58	100	4	C:GO:0000786; F:GO:0003677; C:GO:0005634; F:GO:00
5.34E-19	90.46	7	C:GO:0005834; P:GO:0007186; P:GO:0009845; P:GO:00
2.82E-120	85.25	4	F:GO:0004059; C:GO:0005634; C:GO:0009507; P:GO:00
2.43E-175	85.72	1	F:GO:0016853
1.47E-42	96.79	1	F:GO:0016853
0	82.62	6	F:GO:0004575; P:GO:0005982; P:GO:0005987; C:GO:00
1.25E-112	75.43		
0	94.8	2	P:GO:0006511; F:GO:0031625
0	95.62	5	P:GO:0006511; P:GO:0016567; C:GO:0031461; F:GO:00

2.61E-141	94.67	5	F:GO:0000287; F:GO:0004427; C:GO:0005829; P:GO:00
1.74E-159	81.91	3	P:GO:0000413; F:GO:0003755; P:GO:0006457
0	88.33	5	F:GO:0003677; P:GO:0006629; C:GO:0016021; F:GO:00
0	84.01	2	C:GO:0016020; F:GO:0016798
0	85	3	F:GO:0004566; P:GO:0008152; C:GO:0016020
2.51E-38	89.33		
6.07E-36	90.41		
0	61.52		
0	91.86	2	C:GO:0016021; F:GO:0047184
0	83.16	8	F:GO:0000977; C:GO:0005794; C:GO:0005886; P:GO:00
0	85.3	6	F:GO:0005524; P:GO:0006090; F:GO:0016301; P:GO:00
0	85.35	6	F:GO:0005524; P:GO:0006090; F:GO:0016301; P:GO:00
1.97E-70	55.17	1	F:GO:0016301
0	78.03	4	F:GO:0003700; C:GO:0005634; P:GO:0006355; F:GO:00
5.30E-107	56.29	4	F:GO:0003676; C:GO:0005634; F:GO:0008270; P:GO:00
0	89.27	4	C:GO:0005886; C:GO:0016021; F:GO:0022857; P:GO:00
1.48E-147	75.8	1	C:GO:0016021
0	83.55	7	F:GO:0004674; F:GO:0005524; C:GO:0005634; C:GO:00
9.37E-103	69.94		
0	89.51		
0	78.54	2	P:GO:0008152; F:GO:0016491
0	86.14	11	F:GO:0004029; C:GO:0005773; C:GO:0005783;
8.24E-118	84.54	10	F:GO:0004791; C:GO:0005737; P:GO:0006662;
0	79.72	6	F:GO:0004497; F:GO:0005506; C:GO:0016021; F:GO:00
2.72E-127	79.8	3	C:GO:0009535; C:GO:0010598; C:GO:0016021
3.81E-87	73.27	3	C:GO:0009535; C:GO:0010598; C:GO:0016021
0	88.69	3	F:GO:0004252; P:GO:0006508; C:GO:0016021
5.80E-131	87.16	1	C:GO:0016021
0	86.66	2	P:GO:0005975; F:GO:0042973
1.02E-33	84.74		
0	81.63	3	P:GO:0009987; C:GO:0016021; F:GO:0016757
7.17E-129	100		
0	74.34	2	C:GO:0009941; C:GO:0016020
0	67.65	4	F:GO:0003677; C:GO:0005634; P:GO:0006351; P:GO:00
8.65E-106	80.54	2	P:GO:0016311; F:GO:0047538
0	95.95	11	F:GO:0005524; C:GO:0005739; C:GO:0005829;
8.64E-60	95.92	6	F:GO:0005524; C:GO:0005737; P:GO:0006511;
3.99E-33	86.8	2	F:GO:0016491; P:GO:0055114
2.30E-79	90.93	8	F:GO:0004807; C:GO:0005829; P:GO:0006094; P:GO:00
0	92.23	15	F:GO:0004807; C:GO:0005739; C:GO:0005829; P:GO:00
0	72.18	1	P:GO:0050794
4.90E-67	59.9		
0	92.6	4	C:GO:0005737; F:GO:0016656; F:GO:0050660; P:GO:00
1.00E-33	97.54		
2.57E-166	94.7	4	P:GO:0006072; C:GO:0009331; F:GO:0052591; P:GO:00
2.56E-114	84.42	1	C:GO:0005576
0	82.04	11	P:GO:0002229; P:GO:0009620; P:GO:0009751; P:GO:00
1.68E-84	93.33	12	P:GO:0009409; C:GO:0009523; C:GO:0009535; P:GO:00
4.09E-160	87.83	12	P:GO:0009409; C:GO:0009523; C:GO:0009535; P:GO:00
5.46E-53	89.92	7	F:GO:0000981; F:GO:0001135; C:GO:0005634; P:GO:00

0	76.72	1	C:GO:0005622
0	76.13	1	C:GO:0005622
0	86.01	6	P:GO:0000184; F:GO:0003677; F:GO:0004386; F:GO:00
2.09E-109	90.48	1	C:GO:0016021
4.62E-84	91.27	9	F:GO:0005507; C:GO:0005737; C:GO:0005886; C:GO:00
1.09E-108	89.36	9	F:GO:0005507; C:GO:0005737; C:GO:0005886; C:GO:00
0	76.39	4	F:GO:0004674; F:GO:0005524; P:GO:0006468; C:GO:00
2.70E-41	93.08		
0	83.68	4	P:GO:0009734; P:GO:0010087; P:GO:0010305; C:GO:00
0	70.01	2	C:GO:0016020; C:GO:0016021
1.72E-117	97.66	7	C:GO:0005768; C:GO:0005829; P:GO:0006886; P:GO:00
0	84.09	3	C:GO:0005829; P:GO:0009646; P:GO:0090549
0	81.39	3	P:GO:0000272; F:GO:0016161; F:GO:0102229
7.54E-116	79.06	3	P:GO:0000272; F:GO:0016161; F:GO:0102229
5.17E-173	78.48	3	P:GO:0000272; F:GO:0016161; F:GO:0102229
0	73.67	2	C:GO:0005634; C:GO:0005737
0	78.57	1	F:GO:0016740
0	86.58	1	C:GO:0016021
1.78E-79	96.77	3	C:GO:0005634; P:GO:0009691; F:GO:0016787
7.11E-48	87.3		
0	75.74	2	F:GO:0003700; P:GO:0006355
0	74.99	2	F:GO:0003700; P:GO:0006355
4.09E-172	73.65	3	F:GO:0005524; P:GO:0006298; F:GO:0030983
0	90.03	6	C:GO:0005783; P:GO:0006629; P:GO:0010025; P
2.66E-81	82.94	2	F:GO:0004364; F:GO:0016829
0	92.98	4	F:GO:0008970; C:GO:0016021; F:GO:0052739; F:GO:00
2.13E-161	93.43	4	F:GO:0008970; C:GO:0016021; F:GO:0052739; F:GO:00
4.68E-86	68.07	1	F:GO:0005488
6.07E-67	92.75	8	F:GO:0004791; C:GO:0005623; F:GO:0009055;
0	73.18	1	F:GO:0003677
0	72.38	1	F:GO:0003677
0	87.27	2	F:GO:0030410; P:GO:0030418
1.02E-49	93.66	7	P:GO:0006021; F:GO:0008934; P:GO:0046854;
1.11E-82	61.85		
6.15E-93	64.2		
7.48E-41	82.79	2	F:GO:0005524; F:GO:0016740
0	92.67	5	P:GO:0005975; F:GO:0008184; F:GO:0030170; F:GO:01
0	93.7	6	C:GO:0005737; P:GO:0005980; F:GO:0008184; F:GO:00
1.41E-56	92.02	3	C:GO:0000813; P:GO:0015031; P:GO:0032509
0	92.48	7	F:GO:0005388; F:GO:0005516; F:GO:0005524; C:GO:00
1.23E-84	82.5		
2.93E-135	92	2	P:GO:0006890; C:GO:0016021
0	82.59	4	C:GO:0005737; P:GO:0005975; C:GO:0016021; F:GO:01
1.49E-24	66.12		
0	94.34	7	C:GO:0000932; F:GO:0003676; F:GO:0004535; P:GO:00
5.19E-33	93.82	2	F:GO:0003723; F:GO:0008270
3.67E-18	95.25		
0	79.82	1	C:GO:0016021
0	78.44	6	F:GO:0003676; P:GO:0006396; F:GO:0016891; P:GO:00
0	75.26	6	F:GO:0003676; P:GO:0006281; F:GO:0016779; P:GO:00

5.59E-40	80.85	2	F:GO:0003676; P:GO:0031047
0	84.77	2	F:GO:0003676; P:GO:0031047
0	85.02	1	F:GO:0008270
0	70.97	2	P:GO:0006338; C:GO:0031011
0	72.47	2	P:GO:0006338; C:GO:0031011
0	95.74	9	C:GO:0000139; F:GO:0000287; F:GO:0004012; F:GO:00
1.82E-12	91.78	1	C:GO:0016021
4.74E-177	98.74	7	C:GO:0009522; C:GO:0009523; C:GO:0009535;
7.86E-151	99.67	7	C:GO:0009522; C:GO:0009523; C:GO:0009535; P:GO:0(
5.81E-143	75.38	1	F:GO:0008233
0	80.83	2	C:GO:0005634; P:GO:0006355
0	79.18		
1.82E-120	88.32	4	C:GO:0005829; C:GO:0009535; C:GO:0010598; C:GO:0(
0	83		
0	87.3	5	P:GO:0006855; F:GO:0015238; F:GO:0015297; C:GO:00
8.26E-67	78.4	6	C:GO:0009507; F:GO:0009540; P:GO:0009688; C:GO:0(
3.39E-108	82.83	1	C:GO:0016020
6.28E-172	87.49	3	C:GO:0016021; F:GO:0016279; P:GO:0018026
0	97.55	8	F:GO:0003677; F:GO:0004386; F:GO:0005524; C:GO:00
4.25E-103	89.11	4	C:GO:0005737; P:GO:0030001; F:GO:0046914; P:GO:0(
2.59E-56	98.82	20	F:GO:0002020; F:GO:0005524; C:GO:0005618; C:GO:00
0	99.09	1	F:GO:0005524
1.31E-174	94.41	6	C:GO:0005887; P:GO:0006833; C:GO:0009705; F:GO:0(
0	91.6	5	F:GO:0003855; F:GO:0004764; P:GO:0019632; F:GO:00
0	63.75		
0	63.75		
7.53E-150	74.88	6	C:GO:0005783; C:GO:0005794; P:GO:0006506; P:GO:0(
0	88.88	2	P:GO:0016311; F:GO:0033883
2.12E-98	95.1	2	F:GO:0016491; P:GO:0055114
1.28E-122	74.82	2	P:GO:0000045; C:GO:0000407
2.37E-151	82.35	11	F:GO:0000155; P:GO:0000160; C:GO:0009570; P:GO:0(
0	91.03	7	F:GO:0005524; F:GO:0015419; P:GO:0015709; C:GO:00
1.94E-42	73.2	1	C:GO:0005730
3.35E-178	93.04	7	F:GO:0005524; F:GO:0015419; P:GO:0015709; C:GO:00
9.43E-35	96.3	2	P:GO:0008152; F:GO:0050203
0	88.38	4	F:GO:0004197; C:GO:0005773; P:GO:0006624; P:GO:0(
0	72.32	4	F:GO:0004672; C:GO:0005622; P:GO:0006468; P:GO:0(
1.55E-41	58.8		
0	88.2	6	F:GO:0000166; F:GO:0004008; C:GO:0016021; F:GO:00
6.54E-145	80.85		
3.21E-143	79.1		
2.34E-99	88.05	3	F:GO:0016618; F:GO:0051287; P:GO:0055114
0	84.29		
0	83.87	2	C:GO:0030176; P:GO:0031204
1.45E-104	91.63		
0	93.52	2	C:GO:0016021; P:GO:0090480
0	79.81	3	F:GO:0004674; F:GO:0005524; P:GO:0006468

1.61E-39	80.86	2	F:GO:0003677; C:GO:0016021
1.11E-09	91.77		
0	63.73		
0	94.22	3	F:GO:0005507; F:GO:0008447; P:GO:0055114
0	94.22	3	F:GO:0005507; F:GO:0008447; P:GO:0055114
0	72.67	5	F:GO:0000166; F:GO:0003777; C:GO:0005871; P:GO:00
0	90.32	1	F:GO:0046872
9.53E-82	98.68	1	F:GO:0005509
0	99.54	6	F:GO:0005524; C:GO:0009535; P:GO:0015986;
0	98.41	1	F:GO:0005524
0	80.01	2	F:GO:0008168; P:GO:0032259
0	62.14	3	F:GO:0003677; C:GO:0005634; F:GO:0046872
0	62.39	3	F:GO:0003677; C:GO:0005634; F:GO:0046872
0	81.7	3	F:GO:0003676; F:GO:0004386; F:GO:0005524
3.84E-108	96.84	4	F:GO:0005524; F:GO:0016874; P:GO:0016925; F:GO:00
4.64E-145	75.3	1	F:GO:0005525
2.96E-145	75.3	1	F:GO:0005525
0	89.6		
2.19E-133	88.64	4	F:GO:0005385; C:GO:0005886; C:GO:0016021; P:GO:00
7.05E-105	96.16	3	F:GO:0003700; P:GO:0006352; P:GO:0006355
0	69.04	1	C:GO:0000228
0	68.36	1	C:GO:0000228
0	84.49	6	F:GO:0004672; F:GO:0005524; P:GO:0006468; F:GO:00
0	90.78	13	P:GO:0000373; F:GO:0003729; F:GO:0004004; F:GO:00
0	92.41	13	P:GO:0000373; F:GO:0003729; F:GO:0004004; F:GO:00
3.55E-41	80.35		
0	81.24	1	F:GO:0046983
0	84.52	1	C:GO:0016021
0	72.78	2	F:GO:0004721; F:GO:0043169
1.76E-20	99.14	2	F:GO:0003723; C:GO:0016021
0	82.06	5	C:GO:0000139; P:GO:0006486; F:GO:0008378; C:GO:00
0	95.65	2	F:GO:0016630; P:GO:0055114
0	92.47	5	F:GO:0003951; F:GO:0004143; F:GO:0005524; P:GO:00
1.92E-161	82.8	7	F:GO:0004791; C:GO:0005737; P:GO:0035556; P:GO:00
8.07E-149	86.91	7	F:GO:0004176; F:GO:0005524; C:GO:0005759; P:GO:00
0	91.67	4	F:GO:0004674; F:GO:0005524; P:GO:0006468; P:GO:00
7.37E-102	85.78	1	F:GO:0046983
2.45E-170	90.14	5	F:GO:0005524; C:GO:0005886; C:GO:0016021; F:GO:00
0	83.4	5	F:GO:0005524; C:GO:0005886; C:GO:0016021; F:GO:00
0	93.25	2	F:GO:0008270; C:GO:0016021
0	92.2	8	P:GO:0000245; C:GO:0000974; C:GO:0016021; C:GO:00
5.33E-18	61.76		
0	95.88	3	F:GO:0003882; P:GO:0006659; C:GO:0016021
0	92.97	3	F:GO:0003882; P:GO:0006659; C:GO:0016021
1.81E-155	91.33	3	F:GO:0004089; F:GO:0008270; P:GO:0015976
2.34E-59	94.19		
3.12E-56	79.88	2	F:GO:0003978; P:GO:0006012
0	60.15		
0	88.37	6	P:GO:0000462; P:GO:0000466; F:GO:0003723;
3.56E-174	91.86	6	P:GO:0000462; P:GO:0000466; F:GO:0003723;

6.52E-156	90.11	5	F:GO:0003855; F:GO:0004764; P:GO:0019632; F:GO:00
3.09E-134	76.11	1	C:GO:0005576
0	83.17		
0	83.3	10	F:GO:0000287; C:GO:0005737; F:GO:0008270; P:GO:00
0	89.61	10	F:GO:0000287; C:GO:0005737; F:GO:0008270; P:GO:00
3.61E-62	62.67	2	C:GO:0016020; C:GO:0016021
0	71.68	7	P:GO:0006796; C:GO:0016020; F:GO:0016301; P:GO:00
0	97.82	12	P:GO:0009416; C:GO:0009522; C:GO:0009523; C:GO:00
0	90.6	4	C:GO:0005886; C:GO:0016021; F:GO:0022857; P:GO:00
0	82.91	2	C:GO:0005623; P:GO:0009902
0	85.42	2	C:GO:0005634; P:GO:0008380
1.20E-112	75.15	1	F:GO:0046983
0	83.82	1	F:GO:0046983
3.61E-66	82.87	3	F:GO:0003676; F:GO:0032041; P:GO:0070932
1.25E-61	81.53	3	F:GO:0003676; F:GO:0032041; P:GO:0070932
0	84.37	3	P:GO:0001676; F:GO:0004467; F:GO:0102391
0	84.37	3	P:GO:0001676; F:GO:0004467; F:GO:0102391
0	57.64		
1.26E-36	98.86	3	F:GO:0003735; C:GO:0005840; P:GO:0006412
1.53E-90	82.83		
0	77.92		
2.14E-165	96.58	6	P:GO:0000165; F:GO:0004707; F:GO:0005524; C:GO:00
0	68.18	1	F:GO:0003676
1.68E-74	85.44	2	F:GO:0047641; P:GO:0055114
0	86.4	4	C:GO:0005829; P:GO:0046686; F:GO:0047641; P:GO:00
0	87.9	4	C:GO:0005829; P:GO:0046686; F:GO:0047641; P:GO:00
0	77.95		
4.19E-177	91.66	6	C:GO:0000502; F:GO:0005524; C:GO:0009570; C:GO:00
8.23E-27	96.04	1	F:GO:0005524
2.07E-88	95.81	2	F:GO:0004633; F:GO:0005524
5.20E-39	87.87	1	F:GO:0005524
3.96E-122	74.24	4	P:GO:0006816; F:GO:0008324; C:GO:0016020; P:GO:00
0	80.39		
1.21E-81	86.71	1	F:GO:0016746
2.46E-94	99.04	2	F:GO:0004332; P:GO:0006096
1.04E-36	84.04	1	C:GO:0016021
8.78E-70	81.75	3	C:GO:0016020; F:GO:0016301; P:GO:0016310
0	87.3	4	F:GO:0004674; F:GO:0005524; P:GO:0006468; C:GO:00
0	63.04	3	F:GO:0008289; C:GO:0016020; C:GO:0016021
0	64.63	3	F:GO:0008289; C:GO:0016020; C:GO:0016021
0	59.52	10	F:GO:0000166; F:GO:0005524; C:GO:0005886; P:GO:00
4.04E-130	78.79	4	F:GO:0004828; F:GO:0005524; C:GO:0005737; P:GO:00
0	95.52	6	P:GO:0006508; F:GO:0008237; P:GO:0009408; P:GO:00
0	92.81	6	P:GO:0006508; F:GO:0008237; P:GO:0009408; P:GO:00
0	92.18	6	P:GO:0006508; F:GO:0008237; P:GO:0009408; P
0	93.49	3	F:GO:0004766; P:GO:0006596; F:GO:0010487
0	81.96		
0	96.13	20	F:GO:0004372; C:GO:0005634; C:GO:0005739;
0	91.45	11	C:GO:0000145; C:GO:0005829; C:GO:0005886; P:GO:00

0	91.6	5	F:GO:0004497; F:GO:0005506; P:GO:0008610; C:GO:00
8.68E-90	76.6		
8.03E-96	78.81		
3.70E-90	92.21	4	F:GO:0003723; C:GO:0005634; P:GO:0006397; P:GO:00
0	85.88	9	C:GO:0005774; C:GO:0005886; P:GO:0006885; P:GO:00
0	91.39	9	F:GO:0003690; F:GO:0003727; P:GO:0006355; P:GO:00
8.02E-101	84.1	2	F:GO:0003712; P:GO:0006355
3.46E-92	97.16	7	F:GO:0003958; C:GO:0005789; F:GO:0010181; C:GO:00
0	72.07	1	F:GO:0005488
1.43E-58	91.15	1	P:GO:0006355
5.35E-41	81.94		
1.30E-43	79.47		
0	89.62	2	F:GO:0004435; P:GO:0006629
0	91.76		

GO Names	Enzyme Codes
P:response to heat	
P:glutamate biosynthetic process; C:plastid; F	
F:phosphorelay sensor kinase activity;	EC:2.7.13.3
F:ATP binding; C:integral component of membrane;	EC:3.6.1.3; EC:3.6.1.15
F:ATP binding; C:integral component of membrane	EC:3.6.1.3; EC:3.6.1.15
C:integral component of membrane	
F:malate dehydrogenase (decarboxylating) (NAD+) activity;	EC:1.1.1.38; EC:1.1.1.39;
F:carbamoyl-phosphate synthase (glutamine-hydrolyzing) activity;	EC:6.3.5.5
F:phosphorelay sensor kinase activity; P:phosphorelay signal trans	EC:2.7.13.3
C:membrane; C:integral component of membrane	
C:Golgi membrane; C:integral component of membrane;	EC:2.4.1.43
P:long-chain fatty acid metabolic process;	EC:6.2.1.3
P:long-chain fatty acid metabolic process;	EC:6.2.1.3
C:Golgi membrane; P:intracellular protein transport;	
F:RNA binding; C:nucleus; C:cytoplasm;	
F:RNA binding; C:nucleus; C:cytoplasm;	
C:intracellular part	
C:nucleus; P:response to light stimulus;	
C:nucleus; F:zinc ion binding	
C:intracellular	
F:cinnamoyl-CoA reductase activity;	EC:1.2.1.44
F:histone acetyltransferase activity; P:histone acetylation	EC:2.3.1.5; EC:2.3.1.48
P:cellular process; F:oxidoreductase activity; P:oxidation-reduction process	
F:RNA binding	
P:glucose metabolic process; F:oxidoreductase activity,	
F:protein serine/threonine phosphatase activity;	EC:3.1.3.16; EC:3.1.3.41
F:ATP binding; C:integral component of membrane	
F:ATP binding	
F:ATP binding; C:endoplasmic reticulum	EC:1.3.1.74
F:serine-type endopeptidase activity; C:peroxisome;	EC:3.4.21
P:regulation of alternative mRNA splicing, via spliceosome;	
P:regulation of alternative mRNA splicing, via spliceosome;	
P:regulation of alternative mRNA splicing, via spliceosome; P:mRNA 3'-splice site recognitio	
F:oxidoreductase activity	
F:phosphorelay sensor kinase activity;	EC:2.7.13.3
F:ADP binding	
F:ADP binding	
C:cytoplasm; P:cellular process; F:ubiquitin protein ligase binding;	

F:methyltransferase activity; P:methylation	
P:MAPK cascade; F:MAP kinase activity; F:ATP binding; C:intracellu	EC:2.7.11; EC:2.7.11.24
F:oxidoreductase activity, acting on CH-OH group of donors;	
C:EKC/KEOPS complex; P:tRNA threonylcarbamoyladenosine modi	EC:2.3.1.234
F:DNA binding	
C:integral component of membrane	
F:ATP binding; C:integral component of membrane;	EC:3.6.1.3; EC:3.6.1.15
F:actin binding; C:cytoskeleton; P:actin cytoskeleton organization	
F:O-methyltransferase activity;	
F:O-methyltransferase activity; P:methylation;	
C:nuclear pore; P:poly(A)+ mRNA export from nucleus	
C:cytoplasm; C:cytoplasmic dynein complex;	EC:3.6.1.3; EC:3.6.1.15; E
F:transferase activity	
C:chloroplast thylakoid membrane; C:photosystem II oxygen evolving complex;	
C:chloroplast thylakoid membrane; C:photosystem II oxygen evolving complex;	
F:serine-type endopeptidase activity; P:proteolysis; F:tripeptidyl-p	EC:3.4.21; EC:3.4.14
P:lipid metabolic process; P:suberin biosynthetic process;	EC:1.2.1.84
P:nitrogen compound metabolic process; F:transferase activity;	
P:vacuolar acidification; C:RAVE complex;	
P:vacuolar acidification; C:RAVE complex;	
F:ADP binding	
C:integral component of membrane	
F:exonuclease activity;	
F:protein serine/threonine kinase activity; F:ATP binding;	EC:2.7.11
F:histidine-tRNA ligase activity; F:ATP binding; C:cytoplasm;	EC:6.1.1.21
F:nucleic acid binding	
F:nucleic acid binding	
C:vacuolar membrane; C:endoplasmic reticulum;	
C:exocyst; P:Golgi to plasma membrane transport	
F:fructose-bisphosphate aldolase activity; P:glycolytic process	EC:4.1.2.13
F:RNA binding; F:endonuclease activity;	
F:RNA binding; F:metal ion binding	
F:RNA binding; F:metal ion binding	
F:monooxygenase activity; P:secondary metabolite biosynthetic process;	
C:integral component of membrane;	
C:intracellular; F:zinc ion binding; C:membrane;	
C:nucleus; P:metabolic process; F:beta-glucosidase activity;	EC:3.2.1.21
P:protein peptidyl-prolyl isomerization; F:peptidyl-prolyl cis-trans	EC:5.2.1.8
P:cellular metabolic process	
F:hydrolase activity, hydrolyzing O-glycosyl compounds; P:car	
P:mRNA splicing, via spliceosome; F:nucleic acid binding;	EC:3.6.1.3; EC:3.6.1.15
F:folic acid transmembrane transporter activity; C:chloroplast envelope;	
F:catalytic activity; P:metabolic process	
F:catalytic activity; P:metabolic process	

F:channel activity; C:integral component of membrane; P:transmembrane transport	
F:pantothenate kinase activity; F:ATP binding;	EC:2.7.1.33
C:cytosol	
F:ionotropic glutamate receptor activity; P:ion transport;	
C:plasmodesma	
C:nucleus	
C:integral component of membrane	
C:integral component of membrane	
F:damaged DNA binding; C:nucleus; P:nucleotide-excision repair;	
F:damaged DNA binding; C:nucleus; P:nucleotide-excision repair;	
P:nitric oxide biosynthetic process; F:nitrate reductase (NADH) act	EC:1.7.1.1; EC:1.7.1.3; EC
F:oxidoreductase activity; P:oxidation-reduction process	
F:oxidoreductase activity; P:oxidation-reduction process	
C:integral component of membrane; F:transmembrane transporter activity;	
F:ATP binding; P:protein folding; P:response to stress; F:unfolded protein binding	
F:ATP binding; C:cytoplasm; P:protein folding;	
F:ATP binding; P:protein folding; P:response to stress; F:unfolded protein binding	
C:nucleosome; F:DNA binding; C:nucleolus;	
F:glycerol-3-phosphate dehydrogenase [NAD+] activity;	EC:1.1.1.8; EC:1.1.1.94
C:proteasome complex; F:ATP binding;	EC:3.6.1.3; EC:3.6.1.15
F:DNA-binding transcription factor activity; P:regulation of transcription, DNA-templated	
F:DNA-binding transcription factor activity; P:regulation of transcription, DNA-templated	
F:protein heterodimerization activity	
C:integral component of membrane;	EC:1.13.11; EC:1.14.19
C:cytosol; P:monoterpenoid biosynthetic process;	
P:regulation of transcription, DNA-templated	
P:sucrose metabolic process; F:sucrose synthase activity	EC:2.4.1.13
F:protein domain specific binding	
F:kinase activity; P:phosphorylation	
C:endoplasmic reticulum membrane; P:response to auxin;	
C:integral component of membrane	
C:integral component of membrane	
F:sterol 24-C-methyltransferase activity;	EC:2.1.1.161; EC:2.1.1.16
F:DNA binding; C:nucleus; P:regulation of transcription, DNA-templated	
F:DNA binding; C:nucleus; P:regulation of transcription, DNA-templated	
F:ligase activity	
F:RNA binding	
F:phosphorelay sensor kinase activity;	EC:2.7.13.3

F:phosphorelay sensor kinase activity;	EC:2.7.13.3
P:carbohydrate metabolic process;	EC:3.2.1.25; EC:3.2.1.78
P:regulation of biological process	
P:regulation of biological process	
F:structural constituent of ribosome; P:translation; C:cytosolic small ribosomal subunit	
P:protein polyubiquitination; C:nucleus; C:cytosol;	
F:nucleic acid binding; P:DNA integration; F:kinase activity; P:phosphorylation	
P:carbohydrate metabolic process; P:dephosphorylation;	EC:3.1.3.37; EC:3.1.3.41
F:catalytic activity; P:GPI anchor biosynthetic process;	
C:integral component of membrane; F:kinase activity; P:phosphorylation	
C:integral component of membrane	
P:GPI anchor biosynthetic process; C:integral component of membrane;	
C:nucleosome; F:DNA binding; C:nucleus; P:nucleosome assembly	
F:nucleic acid binding; F:RNA binding	
C:integral component of membrane; F:transmembrane transporter activity;	
F:RNA binding	
C:integral component of membrane	
C:integral component of membrane	
F:ATP binding	
F:ATP binding; F:2-alkenal reductase [NAD(P)] activity;	EC:1.3.1.74
F:ATP binding; P:protein metabolic process	
F:ATP binding; P:protein metabolic process	
C:vacuole; P:response to chitin	
F:cyclin-dependent protein serine/threonine kinase activity;	EC:2.7.11; EC:2.7.11.22
C:integral component of plasma membrane;	
C:integral component of membrane	
C:integral component of membrane	
P:lipid metabolic process; F:phosphatidylcholine 1-acylhydrolase a	EC:3.1.1.32; EC:3.1.1.1
P:lipid metabolic process; F:phosphatidylcholine 1-acylhydrolase a	EC:3.1.1.32; EC:3.1.1.1
F:cystathionine gamma-synthase activity;	EC:2.5.1.48
F:adenosylmethionine decarboxylase activity	EC:4.1.1.50
C:vacuole; P:response to stress	
C:nucleosome; F:DNA binding; C	
C:nucleosome; P:nucleosome assembly;	
C:nucleosome; P:nucleosome assembly;	
C:nucleosome; F:DNA binding; C:rDNA protrusion;	
C:nucleosome; F:DNA binding; C:rDNA protrusion;	
F:UDP-N-acetylglucosamine diphosphorylase activity;	EC:2.7.7.23

F:adenylate kinase activity; F:ATP binding; P:nucleotide phosphory	EC:2.7.4.4; EC:2.7.4.3
F:adenylate kinase activity; F:ATP binding; P:nucleotide phosphory	EC:2.7.4.4; EC:2.7.4.3
F:peptidase activity; C:integral component of membrane	
F:protein serine/threonine kinase activity;	EC:2.7.11
F:acid phosphatase activity; P:dephosphorylation	EC:3.1.3.41; EC:3.1.3.2
F:beta-galactoside (CMP) alpha-2,3-sialyltransferase activity;	EC:2.4.99; EC:2.4.99.4
F:hydrolase activity, acting on glycosyl bonds	
P:intracellular protein transport; P:vesicle-mediated transport;	
F:translation initiation factor activity; P:translational initiation;	
F:translation initiation factor activity; P:translational initiation;	
F:protein kinase activity; F:calcium ion binding; F:ATP binding;	
F:protein kinase activity; F:calcium ion binding; F:ATP binding;	
F:4-alpha-glucanotransferase activity; P:glycogen metabolic proce	EC:2.4.1.25
F:nucleic acid binding	
F:alpha-1,3-mannosylglycoprotein 2-beta-N-acetylglucosaminyltra	EC:2.4.1.101
F:alpha-1,3-mannosylglycoprotein 2-beta-N-acetylglucosaminyltra	EC:2.4.1.101
F:thiol-dependent ubiquitin-specific protease activity; C:nucleus; (	EC:3.4.19.12
F:ATP binding; P:proteolysis; F:peptidase activity	
F:ARF guanyl-nucleotide exchange factor activity; C:Sec61 translocon complex;	
C:integral component of membrane	
F:ATP binding; C:mitochondrion; C:cytosol;	
F:3-beta-hydroxy-delta5-steroid dehydrogenase activity;	EC:1.1.1.145; EC:1.3.99.6
F:zinc ion binding	
F:transmembrane receptor protein serine/threonine kinase activit	EC:2.7.11
F:fatty acid binding; P:fatty acid metabolic process; C:chloroplast stroma;	
F:calmodulin binding; P:response to stress	
C:integral component of plasma membrane;	
F:cysteine-type endopeptidase activity; P:proteolysis	EC:3.4.22
F:iron ion binding; C:integral component of membrane	EC:1.14.13; EC:1.14.13.11
F:RNA binding	
C:integral component of membrane	
P:metabolic process; C:integral component of membrane;	
P:activation of MAPKK activity; F:MAP kinase kinase kinase activity	EC:2.7.11.25; EC:2.7.11
F:transferase activity, transferring glycosyl groups	
P:metabolic process; C:plastid; F:transferase activity, transferring hexosyl groups	
C:nucleus; P:chromatin assembly or disassembly	
F:nucleic acid binding	
P:iron-sulfur cluster assembly; F:transferase activity, transferring acyl groups;	
C:integral component of membrane	

F:methyltransferase activity; P:methylation	
F:microtubule motor activity; F:ATP binding; P:microtubule-based	EC:3.6.1.15
F:RNA binding; C:integral component of membrane	
F:protein serine/threonine kinase activity; F:ATP binding; C:nucleu	EC:2.7.11
P:response to acid chemical; F:DNA binding; C:nucleus; P	
F:calcium ion binding; C:chloroplast thylakoid membrane;	
F:transferase activity	
F:structural constituent of ribosome; C:ribosome; P:translational elongation	
F:nucleic acid binding; F:zinc ion binding	
F:acetyl-CoA C-acetyltransferase activity; C:cytosol;	EC:2.3.1.16; EC:2.3.1.9
F:acetyl-CoA C-acetyltransferase activity; C:cytosol;	EC:2.3.1.16; EC:2.3.1.9
F:protein serine/threonine phosphatase activity;	EC:3.1.3.16; EC:3.1.3.41
F:magnesium ion binding; C:cytosol; F:5'-nucleotidase activity;	EC:3.1.3.41; EC:3.1.3.5; E
F:magnesium ion binding; C:cytosol; F:5'-nucleotidase activity;	EC:3.1.3.41; EC:3.1.3.5; E
P:carbohydrate transport; C:integral component of membrane;	
F:fructose-2,6-bisphosphate 2-phosphatase activity;	EC:3.1.3.23; EC:3.1.3.46;
F:protein kinase activity; F:ATP binding; P:protein phosphorylation	
F:protein kinase activity; F:ATP binding;	
F:protein serine/threonine kinase activity; F:ATP binding;	EC:2.7.11
F:transferase activity	
F:SNARE binding; F:SNAP receptor activity; P:exocytosis;	
C:chloroplast; C:integral component of membrane	
C:chloroplast thylakoid membrane; C:integral component of membrane;	
C:chloroplast thylakoid membrane; C:membrane;	
C:chloroplast; C:membrane	
C:chloroplast	
C:chloroplast; C:integral component of membrane	
F:3-oxoacyl-[acyl-carrier-protein] reductase (NADPH) activity;	EC:2.3.1.85; EC:1.1.1.100
C:membrane; C:integral component of membrane	
C:membrane; C:integral component of membrane	
F:polygalacturonase activity; C:extracellular region;	EC:3.2.1.15
F:nucleic acid binding; F:ATP-dependent RNA helicase activity;	EC:3.6.1.3; EC:3.6.1.15
P:cellular process	
P:cation transport; C:membrane; P:transmembrane transport	
C:photosystem II oxygen evolving complex; P:photosystem II assembly;	
F:DNA binding; F:helicase activity; F:ATP binding; C:nucleus	
F:translation initiation factor activity;	
P:polysaccharide catabolic process; F:beta-amylase activity;	EC:3.2.1.2
C:integral component of membrane	
F:ATP binding; P:protein folding; P:response to heat;	

F:RNA polymerase II regulatory region sequence-specific DNA	
C:integral component of membrane	
F:mannosyl-oligosaccharide glucosidase activity; C:nucleus;	EC:3.2.1.106; EC:3.2.1.12
P:protein polyubiquitination; F:ATP binding; C:cytoplasm;	
C:nucleus; P:cytokinin biosynthetic process; F:hydrolase activity	
F:5-phospho-alpha-D-ribose 1,2-cyclic phosphate 1-phosphohydrc	EC:3.1.4.57
C:chloroplast; C:photosystem I	
F:ATP binding; F:ATPase activity	EC:3.6.1.3; EC:3.6.1.15
F:ATP binding; F:ATPase activity	EC:3.6.1.3; EC:3.6.1.15
P:formation of cytoplasmic translation initiation complex;	
P:protein glycosylation; F:transferase activity, transferring glycosy	EC:1.14.11; EC:1.14.11.1[illegible]
F:methionine-tRNA ligase activity; F:ATP binding; C:mitochondrior	EC:6.1.1.10
F:nucleic acid binding; F:endonuclease activity; P:DNA catabolic process;	
F:thioredoxin-disulfide reductase activity; C:cytoplasm;	EC:1.8.1.9
F:kinase activity; P:phosphorylation	
F:DNA binding	
F:DNA binding	
F:DNA binding; F:metal ion binding	
F:DNA binding; C:nucleus; F:zinc ion binding	
F:DNA-binding transcription factor activity; C:nucleus;	
P:regulation of transcription, DNA-templated; F:transcription regulator activity	
P:regulation of transcription, DNA-templated; F:transcription regulator activity	
F:protein serine/threonine kinase activity; F:calcium ion binding;	EC:2.7.11
F:DNA binding; F:protein dimerization activity	
P:lipid metabolic process; F:hydrolase activity; P:cellular metabolic process	
F:zinc ion binding; F:ligase activity	
C:synaptonemal complex; P:reciprocal meiotic recombination	
C:early endosome; C:plasma membrane;	
C:early endosome; C:plasma membrane	
F:protein serine/threonine/tyrosine kinase activity;	EC:2.7.12.1
C:membrane	
F:transferase activity, transferring acyl groups	
F:protein kinase activity; F:ATP binding;	
F:chloroplast targeting sequence binding;	
F:DNA binding; C:nucleus	
C:integral component of membrane	
F:aspartic-type endopeptidase activity; P:proteolysis	
F:iron ion binding; C:nucleus; C:cytoplasm;	EC:1.13.11; EC:1.13.11.5[illegible]
F:magnesium ion binding; F:pyruvate kinase activity; P:glycolytic p	EC:2.7.1.40
F:DNA binding; F:DNA ligase (ATP) activity; F:ATP binding;	EC:6.5.1.1
F:shikimate O-hydroxycinnamoyltransferase activity	EC:2.3.1.133
C:nucleosome; C:nuclear chromatin; F:DNA binding; P	
C:nucleosome; C:nuclear chromatin; F:DNA binding; P	
C:photosystem I; C:photosystem II;	
C:photosystem I; C:photosystem II; C:chloroplast thylakoid membrane;	

P:response to sucrose; F:kinase activity; F:protein kinase regulator activity;	
P:endocytosis	
P:endocytosis	
F:3-dehydroquinate dehydratase activity;	EC:1.1.1.25; EC:4.2.1.10;
C:mitochondrial inner membrane;	
P:protein dephosphorylation;	EC:3.1.3.16; EC:3.1.3.41
C:membrane; C:integral component of membrane	
F:RNA binding; F:structural constituent of ribosome;	
F:DNA binding; C:nucleus; P:regulation of transcription, DNA-templated	
F:catalytic activity; F:coenzyme binding	
F:catalytic activity; F:coenzyme binding	
P:response to hormone; C:integral component of membrane	
C:vacuolar membrane; C:plasma membrane; P:regulation of pH;	
F:DNA binding; F:DNA-binding transcription factor activity; C:nucleus;	
F:zinc ion binding	
C:integral component of membrane;	
F:phospholipase A2 activity; F:lysophosphatidic acid acyltransfera	EC:3.1.1.1; EC:3.1.1.4
P:drug transmembrane transport; F:drug transmembrane transporter activity;	
P:drug transmembrane transport;	
P:histidine biosynthetic process;	EC:2.6.1.9
F:iron ion binding; C:integral component of membrane;	EC:1.14.13
F:N-acetyl-gamma-glutamyl-phosphate reductase activity;	EC:1.2.1.38
C:plasma membrane; P:G-protein coupled receptor signaling pathway	
F:RNA binding	
F:exonuclease activity; C:integral component of membrane;	
F:cyclin-dependent protein serine/threonine kinase activity; F	EC:2.7.11; EC:2.7.11.22
F:mRNA (guanine-N7-)-methyltransferase activity; P:7-methylguar	EC:2.1.1.56
F:Rho guanyl-nucleotide exchange factor activity; F:kinase activity; P:phosphorylation	
F:nucleic acid binding; F:translation initiation factor activity;	
C:plasma membrane; C:integral component of membrane;	EC:2.4.1.12
C:1,3-beta-D-glucan synthase complex;	EC:2.4.1.34
P:response to stress; P:response to water	
F:alcohol dehydrogenase (NAD) activity; F:zinc ion binding;	EC:1.1.1.1
P:RNA processing	
P:vegetative to reproductive phase transition of meristem;	EC:2.1.1.43
C:Golgi membrane;	EC:3.2.1.113; EC:3.2.1.24
C:Golgi membrane; F:mannosyl-oligosaccharide 1,2-alpha-mannos	EC:3.2.1.113; EC:3.2.1.24

F:5'-nucleotidase activity; P:dephosphorylation	EC:3.1.3.41; EC:3.1.3.5; E
C:intracellular; P:nitrogen compound metabolic process; P:response to stress;	
P:response to hormone; P:regulation of cellular process	
C:plasma membrane	
F:DNA binding; C:nucleus; P:regulation of transcription, DNA-templated	
F:tRNA binding; F:alanine-tRNA ligase activity; F:ATP binding;	EC:6.1.1.7
P:carbohydrate metabolic process; P:dephosphorylation;	EC:3.1.3.37; EC:3.1.3.41
P:carbohydrate metabolic process; P:dephosphorylation; F	EC:3.1.3.37; EC:3.1.3.41
F:iron ion binding; C:endoplasmic reticulum membrane;	EC:1.14.13.152; EC:1.14.1
F:iron ion binding; C:endoplasmic reticulum membrane;	EC:1.14.13.152; EC:1.14.1
F:translation initiation factor activity; P:translational initiation; C:chloroplast thylakoid; P:ph	
F:hydroxymethylglutaryl-CoA reductase (NADPH) activity;	EC:1.1.1.34
F:signal recognition particle binding; F:GTP binding;	
F:DNA binding; C:nucleus	
F:DNA binding; C:nucleus	
F:DNA binding; C:nucleus; P:regulation of transcription, DNA-templated	
F:RNA binding	
F:DNA binding	
C:Golgi membrane; P:intra-Golgi vesicle-mediated transport;	
F:nucleotidyltransferase activity	
C:plant-type vacuole; F:ATP binding; C:vacuolar membrane	EC:3.6.1.3; EC:3.6.3.44; E
P:inositol biosynthetic process; F:inositol monophosphate 1-phosp	EC:3.1.3.25; EC:3.1.3.41
F:aspartic-type endopeptidase activity; P:proteolysis	EC:3.4.23
F:aspartic-type endopeptidase activity; P:proteolysis;	EC:3.4.23
F:DNA binding; C:nucleus	
C:Golgi membrane; C:integral component of membrane;	EC:2.4.1.43
C:extracellular region; C:cell wall	
C:nuclear chromosome, telomeric region; F:DNA binding;	
C:mitochondrial proton-transporting ATP synthase complex, coupling factor F(o);	
C:nucleus	
C:nucleus	
C:nucleus	
F:DNA binding; C:nucleus	
F:RNA binding	
P:regulation of multicellular organismal development	
C:membrane; C:integral component of membrane	
P:regulation of transcription, DNA-templated; F:zinc ion binding	

F:monooxygenase activity; F:iron ion binding;
F:RNA binding; F:phosphoserine phosphatase activity; EC:3.1.3.16; EC:3.1.3.3; E

C:nucleus; C:cytoplasm; P:protein transport

F:nucleic acid binding; F:RNA binding; F:zinc ion binding
F:transcription factor activity, transcription factor binding; C:nucleus; P

F:DNA-binding transcription factor activity;
F:ATP binding; C:mitochondrion; C:chloroplast; EC:6.3.5.7

C:endoplasmic reticulum; P:cellular aldehyde metabolic process;
F:protein dimerization activity
F:protein dimerization activity
F:protein dimerization activity
P:regulation of transcription, DNA-templated
P:long-chain fatty acid metabolic process; F:long-chain fatty acid-(EC:6.2.1.3

C:integral component of membrane

F:transferase activity
F:helicase activity; F:ATP binding; F:ligase activity; F:metal ion binc EC:3.6.1.15

C:membrane

P:protein depalmitoylation; F:lysophospholipase activity; C:cytopl EC:3.1.2.22; EC:3.1.1.5; E
F:alpha-amylase activity; F:calcium ion binding; P:carbohydrate m EC:3.2.1.1
F:ATP binding; C:plasma membrane; C:integral component of mer EC:3.6.1.3; EC:3.6.1.15
C:membrane; C:integral component of membrane
F:voltage-gated chloride channel activity;
F:DNA binding; F:DNA-directed 5'-3' RNA polymerase activity; EC:2.7.7.6

F:proline-tRNA ligase activity; F:ATP binding; C:cytoplasm; EC:6.1.1.15
P:drug transmembrane transport; F:drug transmembrane transporter activity;
C:cytoplasm; P:lipid metabolic process;
C:membrane; C:integral component of membrane;
C:Golgi membrane; F:pyrimidine nucleotide-sugar transmembrane transporter activity;
F:hydrolase activity
F:fructose-bisphosphate aldolase activity; P:glycolytic process EC:4.1.2.13
F:fructose-bisphosphate aldolase activity; P:glycolytic process EC:4.1.2.13

F:UDP-N-acetylglucosamine-dolichyl-phosphate N-acetylglucosam	EC:2.7.8.13; EC:2.7.8.15
C:integral component of membrane	
F:glutathione transferase activity; P:glutathione metabolic proces	EC:2.5.1.18
P:response to stress; C:chloroplast; P:response to abiotic stimulus;	
C:plastid	
C:membrane	
F:zinc ion binding; C:membrane; C:integral component of membrane;	
P:protein glycosylation; C:integral component of membrane;	
F:transcription coregulator activity; F:histone acetyltransferase ac	EC:2.3.1.5; EC:2.3.1.48
F:RNA binding	
P:ribosomal large subunit export from nucleus; C:nucleus;	
F:glutathione-disulfide reductase activity; C:cell; P:glutathione me	EC:1.8.1.7
F:RNA binding; F:ATP-dependent RNA helicase activity; F:ATP bind	EC:3.6.1.3; EC:3.6.1.15
P:carbohydrate metabolic process; C:plant-type cell wall; C:integra	EC:3.2.1.22
F:nuclease activity; P:nucleic acid phosphodiester bond hydrolysis	
F:nucleotide binding; F:ATP binding; P:cell division	
C:Golgi apparatus; C:integral component of membrane	
C:membrane; F:transferase activity, transferring glycosyl groups	
P:response to stress	
P:photoreactive repair; F:deoxyribodipyrimidine photo-lyase activ	EC:4.1.99.3; EC:4.1.99
P:photoreactive repair; F:deoxyribodipyrimidine photo-lyase activ	EC:4.1.99.3; EC:4.1.99
F:hydrolase activity; P:cellular metabolic process	
P:metabolic process; C:integral component of membrane;	EC:3.1.4.3
F:protein serine/threonine kinase activity; F:ATP binding;	EC:2.7.11
P:post-embryonic development; P:reproductive structure development	
F:nucleic acid binding; F:protein kinase activity;	
F:ATP binding; C:mitochondrion; C:vacuolar membrane;	
F:ATP binding; C:plasma membrane; C:integral component of mer	EC:3.6.1.3; EC:3.6.1.15
C:integral component of membrane	
F:DNA binding; F:DNA-binding transcription factor activity; C:nucleus;	
F:6-phosphofructo-2-kinase activity; F:ATP binding; C:cytosol;	EC:2.7.1.105
F:nucleic acid binding; F:helicase activity; F:ATP binding	EC:3.6.1.15
C:viral capsid	
F:RNA binding; F:endonuclease activity; C:nucleus;	
F:iron ion binding; P:glutamate biosynthetic process;	EC:1.4.1.14
F:iron ion binding; C:integral component of membrane;	EC:1.14.13
F:iron ion binding; C:integral component of membrane;	EC:1.14.13
C:integral component of membrane; F:transmembrane transporter activity;	
C:cytoplasm; P:flavonoid biosynthetic process; F:L-ascorbic acid bi	EC:1.14.11; EC:1.14.11.9
C:cytoplasm; F:L-ascorbic acid binding; F:flavonol synthase activity	EC:1.14.11; EC:1.14.11.9;

P:response to oxidative stress; P:anaerobic respiration	
F:ATP binding	
F:DNA binding	
C:integral component of membrane	
C:integral component of membrane; F:transmembrane transporter activity;	
C:photosystem I; C:photosystem II; C:chloroplast thylakoid membrane;	
F:tetrahydrofolylpolyglutamate synthase activity; F:ATP binding	EC:6.3.2.17; EC:6.3.2.12
F:cytidylate kinase activity; F:ATP binding; C:nucleus; C:cytoplasm;	EC:2.7.4.4; EC:2.7.4.14
C:membrane	
C:integral component of membrane	
P:response to reactive oxygen species; F:cytochrome-c peroxidase	EC:1.11.1.5; EC:1.11.1.7
C:intracellular; F:zinc ion binding	
C:intracellular; F:zinc ion binding	
C:intracellular; F:zinc ion binding	
F:methionine adenosyltransferase activity; F:ATP binding; C:cytop	EC:2.5.1.6
F:methionine adenosyltransferase activity; F:ATP binding; C:cytos	EC:2.5.1.6
C:integral component of membrane; F:ligase activity	
F:endonuclease activity; P:nucleic acid phosphodiester bond hydrolysis	
C:chloroplast; P:dephosphorylation; F:N-acylneuraminate-9-phosp	EC:3.1.3.29; EC:3.1.3.41
C:cyclin-dependent protein kinase holoenzyme complex;	
C:integral component of membrane	
C:integral component of membrane	
F:structural constituent of ribosome; C:mitochondrial large ribosomal subunit;	
F:structural constituent of ribosome; C:ribosome; P:translation	
F:alpha-mannosidase activity; P:mannose metabolic process;	EC:3.2.1.24
C:cytosol; P:proteolysis; F:pyroglutamyl-peptidase activity	EC:3.4.19
C:nucleus; F:zinc ion binding; P:response to light stimulus;	
F:serine-type carboxypeptidase activity; P:proteolysis involved in	EC:3.4.21; EC:3.4.16
F:serine-type carboxypeptidase activity; P:proteolysis; C:integral c	EC:3.4.21; EC:3.4.16
C:nucleus; P:regulation of transcription, DNA-templated;	
F:NADH dehydrogenase activity; P:electron transport chain	EC:1.6.99.3
F:1-acylglycerol-3-phosphate O-acyltransferase activity;	EC:2.3.1.51
C:proteasome complex; P:proteasome assembly	
F:ATP binding; F:iron-chelate-transporting ATPase activity	EC:3.6.1.3; EC:3.6.3.34; E
F:ATP binding; F:iron-chelate-transporting ATPase activity;	EC:3.6.1.3; EC:3.6.3.34; E
F:tRNA binding; F:methionine-tRNA ligase activity; F:ATP binding;	EC:6.1.1.10
F:ATP binding; C:mitochondrion; C:cytosol; P:'de novo' protein folding;	
F:catalytic activity; F:coenzyme binding	
P:transcription elongation from RNA polymerase II promoter;	
C:membrane; C:integral component of membrane	
C:integral component of peroxisomal membrane; F:zinc ion binding;	

C:plasma membrane; C:integral component of membrane;	
F:channel activity; C:integral component of membrane;	
F:channel activity; C:integral component of membrane;	
P:sucrose metabolic process; F:sucrose synthase activity;	EC:2.4.1.14; EC:2.4.1.13;
F:transferase activity, transferring glycosyl groups	
F:methyltransferase activity; P:methylation;	EC:1.14.11; EC:1.14.11.3
F:copper ion binding; P:lignin catabolic process;	EC:1.10.3; EC:1.10.3.2
C:chloroplast; P:leaf senescence	
P:response to light stimulus; C:photosystem I; C:photosystem II;	
C:photosystem I; C:photosystem II; C:chloroplast thylakoid membrane;	
C:intracellular; C:nucleus; F:zinc ion binding	
F:peroxidase activity; F:calcium ion binding; C:integral component	EC:1.6.3.1; EC:1.11.1.7
F:UTP:glucose-1-phosphate uridylyltransferase activity;	EC:2.7.7.64; EC:2.7.7.9
F:ATP-dependent peptidase activity; F:metalloendopeptidase acti	EC:3.4.24; EC:3.6.1.3; EC:
P:carbohydrate metabolic process; F:beta-glucosidase activity;	EC:3.2.1.21
C:chloroplast; F:zeaxanthin epoxidase [overall] activity;	EC:1.14.13.90; EC:1.14.1
C:chloroplast thylakoid membrane; F:zeaxanthin epoxidase [overa	EC:1.14.13.90; EC:1.14.1
C:chloroplast; F:zeaxanthin epoxidase [overall] activity; P:abscisic	EC:1.14.13.90; EC:1.14.1
F:protein dimerization activity	
F:protein dimerization activity	
C:integral component of membrane	
F:DNA binding; C:nucleus; F:zinc ion binding;	
F:DNA binding; C:nucleus; F:zinc ion binding;	
F:DNA binding; C:nucleus; F:zinc ion binding;	
F:binding; C:membrane	
F:3-hydroxyacyl-CoA dehydrogenase activity; C:peroxisome;	EC:1.1.1.35
F:ATP binding; C:integral component of membrane; F:hydrolase activity	
F:ATP binding; C:membrane	
P:signal transduction	
F:DNA binding; C:nucleus; P:transcription, DNA-templated;	
C:spindle; C:microtubule; P:activation of protein kinase activity;	
C:nucleus; C:cytosol; P:chaperone-mediated protein complex assembly;	
F:fructose-bisphosphate aldolase activity; P:glycolytic process	EC:4.1.2.13
C:integral component of membrane	
C:integral component of membrane; F:oxidoreductase activity;	
F:acid phosphatase activity; P:dephosphorylation	EC:3.1.3.41; EC:3.1.3.2
F:acid phosphatase activity; P:dephosphorylation	
F:acid phosphatase activity; P:dephosphorylation	EC:3.1.3.41; EC:3.1.3.2
F:ion binding	
F:ATP binding; F:zinc ion binding	
F:DNA-binding transcription factor activity	
F:divinyl chlorophyllide a 8-vinyl-reductase activity;	EC:1.3.1.75
F:ATP binding	
F:ATP binding	

C:intracellular; P:intracellular protein transport; P:v
P:protein glycosylation; C:integral component of membrane;
F:GTP diphosphokinase activity; P:guanosine tetraphosphate meta EC:2.7.6.5

F:hydrolase activity, acting on ester bonds
F:hydrolase activity, acting on ester bonds
P:proteolysis; F:peptidase activity; C:integral component of membrane
C:nucleosome; C:nucleus; P:nucleosome assembly;

C:integral component of membrane
F:nucleic acid binding; F:RNA-DNA hybrid ribonuclease activity;
F:RNA binding
F:nucleic acid binding

F:DNA binding; C:nucleus; P:regulation of transcription, DNA-templated

C:cytoplasm; C:plasma membrane

C:integral component of membrane; F:phosphatidylinositol binding

C:integral component of membrane
F:DNA binding; C:nucleus; P:transcription, DNA-templated;
F:DNA binding; C:nucleus; P:regulation of transcription, DNA-templated;
C:integral component of membrane
F:protein serine/threonine kinase activity; F:ATP binding EC:2.7.11
C:endoplasmic reticulum; P:biosynthetic process; C EC:4.3.3.2

F:branched-chain-amino-acid transaminase activity; EC:2.6.1.42
C:cytoplasm; P:metal ion transport; F:transition metal ion binding;
C:cytoplasm; P:metal ion transport; F:transition metal ion binding;

F:DNA binding; F:DNA-binding transcription factor activity; C:nucleus;

C:chloroplast; F:cofactor binding
C:integral component of membrane
C:endoplasmic reticulum; C:Golgi apparatus
P:response to light stimulus; C:photosystem I; C:photosystem II;
F:protein disulfide isomerase activity; C:endoplasmic reticulum me EC:5.3.4.1
F:adenylate kinase activity; F:ATP binding; P:nucleotide phosphory EC:2.7.4.4; EC:2.7.4.3
C:intracellular; F:zinc ion binding
C:intracellular; F:zinc ion binding
F:RNA binding
C:endoplasmic reticulum membrane; C:integral component of membrane

F:iron ion binding; C:integral component of membrane; F:flavonoi EC:1.14.13; EC:1.14.13.21

C:integral component of membrane

F:ATP binding
F:ATP binding
F:ATP binding
F:nucleotide binding; F:protein serine/threonine kinase activity; EC:2.7.11
F:RNA binding; F:RNA helicase activity; F:ATP binding; P:RNA catal EC:3.6.1.15

F:DNA binding; F:DNA ligase (ATP) activity; F:ATP binding; P:DNA r EC:6.5.1.1
C:nucleosome; F:DNA binding; C:nucleus; F:protein heterodimerization activity
C:peroxisome; F:feruloyl esterase activity EC:3.1.1.73; EC:3.1.1.1
F:potassium ion transmembrane transporter activity;
F:potassium ion transmembrane transporter activity;

P:carbohydrate metabolic process; F:carbohydrate binding; EC:5.1.3.15
P:carbohydrate metabolic process; F:carbohydrate binding; F EC:5.1.3.15
P:microtubule cytoskeleton organization; F:microtubule binding
C:intracellular; P:protein targeting to peroxisome; P:response to auxin
C:integral component of membrane; P:L-arabinose metabolic proc EC:3.2.1.55
C:integral component of membrane
F:DNA binding; C:nucleus; F:zinc ion binding
F:peptidase activity

P:proteolysis; F:metal ion binding; F:metalloaminopeptidase activi EC:3.4.11
F:nucleic acid binding; F:RNA binding

F:ATP binding; P:protein folding; P:response to heat; F:heat shock protein binding;
C:nucleus; P:intracellular protein transport; P:nucleocytoplasmic transport;
F:ATP binding; P:glycogen biosynthetic process; EC:2.7.7.27
F:cyclin-dependent protein serine/threonine kinase activity; EC:2.7.11; EC:2.7.11.22
P:metabolic process; F:carboxylic ester hydrolase activity EC:3.1.1.1
F:protein dimerization activity
F:DNA binding; C:nucleus; P:transcription, DNA-templated;
P:metabolic process

C:1,3-beta-D-glucan synthase complex; F:1,3-beta-D-glucan syntha EC:2.4.1.34
C:1,3-beta-D-glucan synthase complex; F:1,3-beta-D-glucan syntha EC:2.4.1.34
P:response to oxidative stress
F:voltage-gated potassium channel activity;

F:DNA binding; F:DNA-binding transcription factor activity
F:hydrolase activity
C:membrane
F:DNA binding; P:regulation of transcription, DNA-templated

C:intracellular; F:zinc ion binding	
P:tryptophan biosynthetic process; F:anthranilate phosphoribosylt	EC:2.4.2.18
C:integral component of membrane	
C:cytosol; P:tricarboxylic acid cycle;	EC:4.1.1.31; EC:4.1.1.32
F:core promoter sequence-specific DNA binding;	
C:cytoplasm; C:membrane; F:ubiquitin-protein transferase inhibitor activity	
F:ATP binding	
C:endoplasmic reticulum; C:plasma membrane; P:auxin polar transport;	
P:auxin-activated signaling pathway; C:integral component of membrane;	
P:phosphate-containing compound metabolic process; F:hydrolase activity	
F:magnesium ion binding; F:pyruvate decarboxylase activity;	EC:4.1.1.1
P:protein peptidyl-prolyl isomerization; F:peptidyl-prolyl cis-trans	EC:5.2.1.8
F:catalytic activity; P:nucleoside metabolic process	
C:intracellular; C:nucleus; F:zinc ion binding	
F:phosphatidate phosphatase activity; C:integral component of me	EC:3.1.3.4; EC:3.1.3.41
C:nucleosome; F:DNA binding; C:nucleus; F:protein heterodimerization activity	
C:mitochondrial intermembrane space; F:electron transfer activity;	
F:phosphorelay sensor kinase activity; P:phosphorelay signal trans	EC:2.7.13.3
C:nucleosome; F:DNA binding; C:nucleus; P:nucleosome assembly;	
P:metabolic process; F:ligase activity	
F:catalytic activity; C:integral component of membrane; F:coenzyme binding	
F:catalytic activity; P:nucleoside metabolic process;	
F:catalytic activity; P:nucleoside metabolic process;	
F:catalytic activity; P:nucleoside metabolic process	
C:integral component of membrane	
F:ferrochelatase activity; P:heme biosynthetic process	EC:4.99.1.1
P:response to reactive oxygen species; F:translation elongation fa	EC:3.6.1.15
F:GTPase activity; F:GTP binding	EC:3.6.1.15
P:response to stimulus	
C:chloroplast; C:chloroplast stroma; P:chloroplast organization;	
F:ATP binding; P:carbohydrate metabolic process;	EC:2.7.1.19
C:integral component of membrane	
C:integral component of membrane	
C:integral component of membrane	
C:vacuole; C:plasmodesma; C:integral component of membrane	
F:phosphoacetylglucosamine mutase activity; C:cytosol;	EC:5.4.2.3
P:response to light stimulus; C:photosystem I; C:photosystem II;	
C:plastid; F:chlorophyllide a oxygenase [overall] activity;	EC:1.13.12; EC:1.14.13.12
P:response to hypoxia; F:DNA binding;	
P:pyrimidine nucleotide biosynthetic process;	
F:RNA binding	

F:DNA binding	
F:ADP binding	
C:intracellular; P:intracellular protein transport;	
F:RNA binding	
F:RNA binding	
F:protein phosphatase inhibitor activity; C:cytoplasm;	
P:diacylglycerol biosynthetic process	EC:3.1.3.4; EC:3.1.3.41
C:mitochondrial inner membrane; C:integral component of membrane;	
F:catalytic activity; P:carbohydrate metabolic process;	
F:NADH dehydrogenase activity; C:plasma membrane;	EC:1.6.99.3
C:integral component of membrane;	
F:DNA binding; C:nucleus; C:integral component of membrane; F:metal ion binding	
F:DNA binding; C:nucleus; C:integral component of membrane; F:metal ion binding	
C:cytoplasm; P:carbohydrate metabolic process; F	
C:nucleus; P:cold acclimation;	
F:catalytic activity	
F:catalytic activity	
F:glyceraldehyde-3-phosphate dehydrogenase (NADP+) (non-phos	EC:1.2.1.9
F:RNA binding	
F:ligase activity	
F:nucleic acid binding; F:metal ion binding	
C:integral component of membrane; P:gene silencing by RNA	
F:DNA binding; C:nucleus	
F:acetate-CoA ligase activity; P:metabolic process; C:integral comp	EC:6.2.1.1
C:membrane; F:oxidoreductase activity; F:metal ion binding	
F:monooxygenase activity; F:iron ion binding; C:integral component of membrane;	
F:monooxygenase activity; F:iron ion binding;	
F:monooxygenase activity; F:iron ion binding;	
F:iron ion binding; F:oxidoreductase activity,	
F:monooxygenase activity; F:iron ion binding;	
F:iron ion binding; F:oxidoreductase activity, a	
F:ATP binding; P:protein folding; F:2-alkenal reductase [NAD(P)] a	EC:1.3.1.74
C:vacuole; C:peroxisome; P:carbohydrate metabolic process;	EC:1.1.1.37
C:vacuole; C:peroxisome; P:carbohydrate metabolic process;	EC:1.1.1.37
F:glycerate dehydrogenase activity; F:hydroxypyruvate reductase	EC:1.1.1.29; EC:1.1.1.81
F:glycerate dehydrogenase activity; F:hydroxypyruvate reductase	EC:1.1.1.29; EC:1.1.1.81
P:protein transport; C:integral component of membrane	
F:mRNA binding	
C:commitment complex; P:mRNA splicing, via spliceosome;	

C:commitment complex; P:mRNA splicing, via spliceosome;	
F:copper ion binding; F:primary amine oxidase activity;	EC:1.4.3.21
F:ATP binding; P:glycogen biosynthetic process; F:g	EC:2.7.7.27
F:ATP binding; P:glycogen biosynthetic process; F	EC:2.7.7.27
F:transaminase activity; P:biosynthetic process;	
F:protein kinase activity; F:ATP binding; P:protein phosphorylation;	
C:integral component of membrane; C:intracellular membrane-bounded organelle; C	
F:DNA binding; C:nucleus; P:regulation of transcription,	
C:1,3-beta-D-glucan synthase complex; F:1,3-beta-D-glucan syntha	EC:2.4.1.34
C:1,3-beta-D-glucan synthase complex; F:1,3-beta-D-glucan syntha	EC:2.4.1.34
F:channel activity; C:integral component of membrane; P:transmembrane transport	
F:binding	
F:nucleic acid binding; F:zinc ion binding	
P:regulation of transcription, DNA-templated; F:zinc ion binding	
F:protein kinase activity; F:ATP binding; P:protein phosphorylation	
P:proteolysis; F:metal ion binding; F:metalloaminopeptidase activi	EC:3.4.11
P:chlorophyll biosynthetic process; F:magnesium chelatase activit	EC:6.6.1.1
C:cytosol; C:plasma membrane; P:sucrose metabolic process; C:pl	EC:2.4.1.14; EC:2.4.1.13
F:protein kinase activity; F:ATP binding; P:protein phosphorylation;	
F:ATP binding; F:peptide-transporting ATPase activity;	EC:3.6.1.3; EC:3.6.3.43; E
P:mRNA splicing, via spliceosome; C:spliceosomal complex; F	
P:phosphatidylinositol biosynthetic process; C:PAS complex	
F:serine-type endopeptidase activity; P:proteolysis; C:membrane	EC:3.4.21
P:protein peptidyl-prolyl isomerization; F:peptidyl-prolyl cis-trans	EC:5.2.1.8
C:Golgi apparatus; C:integral component of membrane	
C:cis-Golgi network; C:cytosol; P:intracellular protein transport;	
F:mannosyltransferase activity; C:endoplasmic reticulum membrane;	
C:P-body; F:mRNA binding; C:nucleus; P:mRNA processing;	
F:RNA binding; C:nucleus; P:mRNA processing; F:transaminase activity; F:pyridoxal phospha	
C:integral component of membrane	
C:integral component of membrane	
F:translation initiation factor activity; C:nucleus; C:cytoplasm;	EC:5.3.1.23
C:cytosol; F:S-adenosylmethionine-dependent methyltransferase activity; P:methylation	
F:tRNA binding; F:phenylalanine-tRNA ligase activity;	EC:6.1.1.20
C:integral component of membrane; F:carbohydrate binding	
C:integral component of membrane	
C:cytosol; F:O-acyltransferase activity; F:phosphatidylcholine 1-ac	EC:3.1.1.32; EC:3.1.1.1
F:DNA binding; C:nucleus; F:lipid binding	
F:binding; P:metabolic process; F:oxidoreductase activity	
F:transmembrane receptor protein serine/threonine kinase activit	EC:2.7.11
F:ATP binding; C:plasma membrane; F:proton-exporting ATPase a	EC:3.6.1.3; EC:3.6.3.6; EC
F:glycine dehydrogenase (decarboxylating) activity; C:mitochondri	EC:1.4.4.2
F:glycine dehydrogenase (decarboxylating) activity; C:mitochondri	EC:1.4.4.2
F:transferase activity	
F:phosphorylase activity	EC:2.4.1.1
F:translation elongation factor activity; F:GTPase activity; F:GTP bi	EC:3.6.1.15

F:translation elongation factor activity; F:GTPase activity; F:GTP bi	EC:3.6.1.15
F:transporter activity; C:integral component of membrane	
F:aspartic-type endopeptidase activity; P:proteolysis;	EC:3.4.23
F:cation transmembrane transporter activity; C	
F:ADP binding	
F:ADP binding	
F:DNA-binding transcription factor activity; C:mitochondrion;	
F:DNA-binding transcription factor activity; C:mitochondrion;	
F:actin binding; C:membrane; C:integral component of membrane; F:ligase activity	
F:chaperone binding	
C:nucleosome; F:DNA binding; C:nucleolus; C:vacuolar membrane;	
F:ATP binding; C:plasma membrane; F:ATPase-coupled molybdate	EC:3.6.1.3; EC:3.6.1.15; E
F:transcription factor activity, transcription factor binding; C:nucleus;	
F:ligase activity	
F:peroxidase activity; F:oxygen carrier activity; F:iron ion binding;	EC:1.11.1.7
P:microtubule cytoskeleton organization; C:trans-Golgi network;	EC:1.1.1.29
C:chloroplast; C:integral component of membrane	
F:nucleic acid binding; F:metal ion binding	
F:protein serine/threonine kinase activity; F:ATP binding;	EC:2.7.11
F:RNA binding; F:endonuclease activity; C:mitochondrion;	
F:threonine-type endopeptidase activity; C:nucleus; C:cytoplasm;	EC:3.4.25
P:lignin biosynthetic process; P:methylation; F:caffeoyl-CoA O-me	EC:2.1.1.104
F:hydrolase activity; F:galactinol-raffinose galactosyltransferase ac	EC:2.4.1.67; EC:2.4.1.82
F:serine-type endopeptidase activity; P:proteolysis	EC:3.4.21
C:nucleus; F:zinc ion binding; P:response to light stimulus;	
F:3-methyl-2-oxobutanoate dehydrogenase	EC:1.2.4.4
F:DNA binding; F:DNA-binding transcription factor activity;	
C:ESCRT I complex;	
C:ESCRT I complex;	
F:hydrolase activity	
P:autophagosome assembly;	
P:drug transmembrane transport;	
F:protein serine/threonine kinase activity; F:ATP binding; P	EC:2.7.11
F:protein serine/threonine kinase activity;	EC:2.7.10; EC:2.7.11; EC:2
C:plasma membrane; C:integral component of membrane;	
C:integral component of membrane	
C:cytosol; P:retrograde transport, endosome to Golgi	
C:GARP complex; C:cytosol; P:Golgi to vacuole transport;	
F:DNA binding; F:protein dimerization activity	
F:DNA binding; F:protein dimerization activity	
F:DNA binding; F:protein dimerization activity	
F:DNA binding; C:membrane; P:biological regulation	

F:DNA binding; C:membrane	
F:glycine hydroxymethyltransferase activity; F:methyltransferase a	EC:2.1.2.1
F:acetyl-CoA carboxylase activity; P:fatty acid biosynthetic process	EC:6.4.1.2
F:protein tyrosine phosphatase activity;	EC:3.1.3.16; EC:3.1.3.48;
F:ATP binding; C:plasma membrane;	EC:3.6.1.3; EC:3.6.1.15
F:double-stranded DNA binding; P:regulation of transcription, DNA-templated	
C:vacuolar proton-transporting V-type ATPase, V0 domain;	
F:protein serine/threonine phosphatase activity;	EC:3.1.3.16; EC:3.1.3.41
F:protein serine/threonine phosphatase activity	EC:3.1.3.16; EC:3.1.3.41
C:chloroplast; P:isopentenyl diphosphate biosynthetic process,	EC:1.17.1; EC:1.17.1.2
F:calcium-transporting ATPase activity; F:calmodulin binding;	EC:3.6.1.3; EC:3.6.3.8; EC
P:plastid organization; C:membrane	
F:plastoquinol--plastocyanin reductase activity; P:photosynthesis;	EC:1.10.9; EC:1.10.9.1
F:protein kinase activity; F:ATP binding; P:protein phosphorylation	
F:DNA binding; C:nucleus	
C:vacuole; P:intra-Golgi vesicle-mediated transport; P:Golgi organization;	
F:ATP binding; F:ATPase-coupled sulfate transmembrane transpor	EC:3.6.1.3; EC:3.6.3.25; E
F:protein kinase activity; F:ATP binding; P:protein phosphorylation	
C:intracellular; F:zinc ion binding	
F:copper ion binding; F:electron transfer activity;	
F:copper ion binding; F:electron transfer activity;	
F:copper ion binding; F:electron transfer activity;	
F:copper ion binding; F:electron transfer activity; C	
P:L-arabinose metabolic process; F:alpha-L-arabinofuranosidase a	EC:3.2.1.55
F:protein kinase activity; F:ATP binding; P:protein phosphorylation	
F:RNA binding	
F:damaged DNA binding; F:endonuclease activity; F:ATP binding;	EC:3.6.1.3; EC:3.6.1.15
F:RNA binding; F:endonuclease activity; P:RNA modification;	
F:ATP binding; C:plasma membrane; F:proton-exporting ATPase a	EC:3.6.1.3; EC:3.6.3.6; EC
F:ATP binding; P:proteolysis; F:peptidase activity	
P:carbohydrate metabolic process; F:sterol 3-beta-glucosyltransfe	EC:2.4.1.173
P:regulation of transcription, DNA-templated	
F:iron ion binding; P:lignin biosynthetic process; C:integral compo	EC:1.14.13
F:monooxygenase activity; F:iron ion binding; C:integral component of membrane;	
F:monooxygenase activity; F:iron ion binding; C:integral component of membrane;	
F:ATP-dependent peptidase activity; F:serine-type endopeptidase	EC:3.4.21; EC:3.6.1.3; EC:
F:nucleic acid binding	
F:translation elongation factor activity; C:ribosome;	
F:ligase activity	

P:glycolipid metabolic process; C:endomembrane system;	EC:2.4.2.26
F:calcium ion binding	
F:calcium ion binding; F:calcium-dependent phospholipid binding;	
C:nucleus; P:ubiquitin-dependent protein catabolic process;	
C:integral component of membrane	
C:integral component of membrane	
C:integral component of membrane	
F:ligase activity	
F:catalytic activity; P:nitrogen compound metabolic process;	
F:hydrolase activity	
F:hydrolase activity	
F:glutamate decarboxylase activity; F:calmodulin binding;	EC:4.1.1.15
F:protein serine/threonine kinase activity; F:ATP binding;	EC:2.7.11
P:autophagy of mitochondrion; C:cytosol; P:protein lipidation;	
F:nucleic acid binding; P:regulation of gene expression;	
F:GTPase activator activity; C:intracellular; P:intracellular protein transport;	
F:glucose-6-phosphate isomerase activity; C:cytosol;	EC:5.3.1.9
F:serine-type endopeptidase activity; P:proteolysis	EC:3.4.21
C:AP-5 adaptor complex	
C:chloroplast thylakoid membrane; P:transmembrane transport	
P:carbohydrate metabolic process; F:limit dextrinase activity;	EC:3.2.1.41; EC:3.2.1.142
F:DNA binding; C:nucleus; P:regulation of transcription, DNA-templated	
F:RNA binding; C:integral component of membrane	
C:chloroplast; F:magnesium ion transmembrane transporter activity;	
C:membrane; P:RNA metabolic process; F:kinase activity; P:phosphorylation	
F:DNA binding; C:nucleus; P:regulation of transcription, DNA-templated	
F:DNA binding; C:nucleus; P:regulation of transcription, DNA-templated	
F:DNA binding; C:nucleus; P:transcription, DNA-templated;	
F:DNA binding; C:nucleus; P:transcription, DNA-templated	
F:DNA binding; C:nucleus; P:transcription, DNA-templated;	
F:FMN binding; F:oxidoreductase activity; P:oxidation-reduction process	
C:nucleus; C:chloroplast thylakoid	
F:1,4-alpha-glucan branching enzyme activity; F:hydrolase activity	EC:2.4.1.18
C:intracellular; F:zinc ion binding	
F:microtubule motor activity; F:ATP binding; C:kinesin complex;	EC:3.6.1.3; EC:3.6.1.15
F:FMN binding; F:oxidoreductase activity; P:oxidation-reduction process	
F:FMN binding; C:integral component of membrane;	EC:1.1.3.15
F:FMN binding; F:oxidoreductase activity; P:oxidation-reduction process	

C:trans-Golgi network; C:integral component of membrane;	
P:formation of cytoplasmic translation initiation complex;	
P:formation of cytoplasmic translation initiation complex;	
P:vesicle docking involved in exocytosis	
F:protein serine/threonine kinase activity; F:ATP binding;	EC:2.7.11
P:rRNA 5'-end processing; F:hydrolase activity	
P:protein peptidyl-prolyl isomerization; F:peptidyl-prolyl cis-trans	EC:5.2.1.8
C:integral component of membrane; F:phosphoric ester hydrolase activity	
P:response to stimulus	
C:Golgi apparatus; C:integral component of membrane;	
F:DNA binding; F:actin binding; F:DNA-directed 5'-3' RNA polymera	EC:2.7.7.6
F:ATP binding; F:transferase activity	
F:carbonate dehydratase activity; F:zinc ion binding; P:carbon utili	EC:4.2.1.1
F:DNA binding; F:DNA-binding transcription factor activity; C:nucleus;	
F:RNA binding; F:GTP binding; C:mitochondrion; C:cytosol;	
F:DNA binding; C:nucleus; P:regulation of transcription, DNA-templated;	
F:DNA binding; C:nucleus; P:regulation of transcription, DNA-templated;	
F:DNA binding; F:DNA-binding transcription factor activity; C:nucleus;	
F:binding; C:intracellular part	
F:nucleic acid binding; C:nucleus; C:cytoplasm; P:mRNA polyadenylation;	
F:RNA binding	
P:galactose metabolic process; F:inositol 3-alpha-galactosyltransfe	EC:2.4.1.123
P:galactose metabolic process; P:response to heat; P:response to	EC:2.4.1.123
P:ribosomal large subunit assembly; F:ATP binding; C:nucleus; F:A	EC:3.6.1.3; EC:3.6.1.15
F:potassium ion transmembrane transporter activity;	
F:DNA binding; C:nucleus; P:vegetative to reproductive phase transition of meristem	
C:nucleus; P:regulation of transcription, DNA-templated	
F:cytochrome-c oxidase activity; C:mitochondrial inner membrane	EC:1.9.3.1
P:regulation of photomorphogenesis	
F:hydrolase activity	
F:serine-type endopeptidase activity; C:cytoplasm; P:proteolysis;	EC:3.4.21
P:regulation of transcription by RNA polymerase II; C:mediator co	EC:5.1.3.18
F:structural constituent of ribosome; P:translation;	
P:autophagy; C:Atg1/ULK1 kinase complex	
F:phosphoprotein phosphatase activity; P:protein dephosphorylat	EC:3.1.3.16; EC:3.1.3.41
F:mRNA 3'-UTR binding; C:cytosol; F:metal ion binding; P:3'-UTR-mediated mRNA destabiliza	

F:oxidoreductase activity; P:oxidation-reduction process	
P:phosphorelay signal transduction system; F:DNA binding;	
C:integral component of membrane; F:oxidoreductase activity;	
P:lipid metabolic process	
C:intracellular; P:protein targeting to vacuole; F:clathrin binding	
F:protein serine/threonine kinase activity;	EC:2.7.10; EC:2.7.11; EC:2
F:nucleic acid binding; F:RNA binding	
F:phosphatidylinositol binding	
F:N-acetylglucosaminylphosphatidylinositol deacetylase activity	EC:3.5.1.89
C:extracellular region; C:vacuole; P:defense response;	
C:extracellular region; C:vacuole; P:defense response;	
F:ATP binding; P:cell cycle; F:hydrolase activity; P:cell division	
C:endoplasmic reticulum membrane; C:integral component of membrane	
F:hydrolase activity	
F:hydrolase activity	
C:membrane; C:integral component of membrane	
P:phosphorelay signal transduction system; C:intracellular; C:nucleus;	
P:phosphorelay signal transduction system; C:intracellular; C:nucleus;	
C:nucleosome; F:DNA binding; C:nucleolus; C:vacuolar membrane;	
P:ubiquitin-dependent protein catabolic process; C:cullin-RING ubiquitin ligase complex	
P:snRNA processing	
P:nucleocytoplasmic transport; P:nuclear pore organization;	
F:DNA binding; F:translation initiation factor activity; C:nucleus;	EC:2.4.1.1
F:copper ion binding; F:bilirubin oxidase activity; P:oxidation-redu	EC:1.3.3.5
F:copper ion binding; F:bilirubin oxidase activity; P:oxidation-redu	EC:1.3.3.5
F:voltage-gated chloride channel activity; C:integral component of membrane;	
F:voltage-gated chloride channel activity; C:integral component of membrane;	
F:ATP binding; P:protein folding; P:response to stress; F:unfolded protein binding	
C:intracellular; P:intracellular protein transport; P:vesicle-mediated transport	
F:sucrose alpha-glucosidase activity; C:vacuolar lumen; P:sucrose	EC:3.2.1.20; EC:3.2.1.26;
F:sucrose alpha-glucosidase activity; C:vacuolar lumen; P:carbohy	EC:3.2.1.20; EC:3.2.1.26;
F:nucleic acid binding; C:nucleus; F:methyltransferase activity;	EC:1.14.11; EC:1.14.11.27
F:RNA binding; F:kinase activity; P:phosphorylation; F:metal ion binding	
P:phosphorelay signal transduction system; C:nucleus; C:cytoplasm;	
F:nucleic acid binding; P:regulation of gene expression;	
F:metal ion binding	

C:integral component of membrane	
F:ubiquitin-protein transferase activity; F:ATP binding; P:protein ubiquitination	
P:metal ion transport; F:metal ion binding	
F:protein serine/threonine kinase activity; F:ATP binding;	EC:2.7.11
F:RNA binding; F:structural constituent of ribosome; C:ribosome;	
F:ATP binding; P:protein folding; F:unfolded protein binding	
F:ATP binding; P:protein folding; F:unfolded protein binding	
C:integral component of membrane; F:transferase activity, transferring glycosyl groups	
C:integral component of membrane; F:transferase activity, transferring glycosyl groups	
F:nucleotide binding; P:cellular component organization;	
F:transferase activity	
F:metal ion binding	
F:metal ion binding	
C:integral component of membrane; P:transmembrane transport	
C:integral component of membrane; C:chloroplast membrane	
C:protein storage vacuole; P:seed development	
P:ribosomal small subunit assembly; F:helicase activity; P:rRNA pr	EC:3.6.1.15
C:extracellular region; C:cell wall; P:plant-type cell wall organization;	
F:RNA binding; F:endonuclease activity; P:RNA modification;	
F:3-beta-hydroxy-delta5-steroid dehydrogenase activity;	EC:1.2.1.44; EC:1.1.1.145
C:cytosol; F:cinnamoyl-CoA reductase activity; F:coenzyme binding	EC:1.2.1.44
P:carbohydrate metabolic process; F:glucan endo-1,3-beta-D-gluc	EC:3.2.1.21; EC:3.2.1.39
F:chaperone binding	
F:electron transfer activity; C:chloroplast;	
F:electron transfer activity; C:chloroplast; P:electron transport chain;	
F:DNA binding; F:DNA ligase (ATP) activity; F:ATP binding; P:DNA r	EC:6.5.1.1
C:cytoplasm; P:vesicle-mediated transport; P:response to starvation	
C:nucleus; C:cytoplasm	
P:glycolipid biosynthetic process; C:chloroplast envelope;	
F:acyl-CoA dehydrogenase activity; P:fatty acid beta-oxidation usi	EC:1.3.99.3
F:acyl-CoA dehydrogenase activity; F:protein kinase activity; P:pro	EC:1.3.99.3
F:1-phosphatidylinositol binding; C:clathrin-coated vesicle;	
F:1-phosphatidylinositol binding; C:clathrin-coated vesicle; F	
F:DNA binding; C:nucleus; P:transcription, DNA-templated;	
F:DNA binding; C:nucleus; P:transcription, DNA-templated;	
C:integral component of membrane	
F:protein serine/threonine kinase activity; F:ATP binding; C:nucleu	EC:2.7.11
F:protein serine/threonine kinase activity; F:ATP binding;	EC:2.7.11

C:photosystem II; C:chloroplast thylakoid membrane;
F:monooxygenase activity; F:iron ion binding; C:integral component of membrane

F:protein serine/threonine phosphatase activity; P:protein dephos EC:3.1.3.16; EC:3.1.3.41
P:maturation of SSU-rRNA from tricistronic rRNA transcript (SSU-rRNA, 5.8S rRNA, LSU-rRNA
F:feruloyl esterase activity EC:3.1.1.73; EC:3.1.1.1
P:nitric oxide biosynthetic process; F:nitrate reductase (NADH) act EC:1.7.1.1; EC:1.7.1.3; EC
C:membrane
C:mitochondrial inner membrane; P:mitochondrial transport;

F:DNA binding; F:DNA-binding transcription factor activity; F:ATP binding;
C:photosystem I; C:photosystem II; C:chloroplast thylakoid membrane;
P:response to oxidative stress; P:response to water deprivation; EC:2.4.1.82
F:nucleic acid binding; C:ribosome
P:tRNA modification; C:integral component of membrane; F:4 iron EC:2.8.4.5

F:ferredoxin-NADP+ reductase activity; C:chloroplast; EC:1.18.1; EC:1.18.1.2
C:cytosol; P:sucrose biosynthetic process; P:fructose metabolic pr EC:3.1.3.23; EC:3.1.3.11;
C:nucleus; C:cytosol; P:starch catabolic process; P:sucrose biosynt EC:3.1.3.23; EC:3.1.3.11;
P:carbohydrate metabolic process; P:dephosphorylation; EC:3.1.3.23; EC:3.1.3.11;
P:regulation of transcription, DNA-templated; F:metal ion binding
C:photosystem II; P:photosynthesis; C:integral component of membrane;
F:phosphorelay sensor kinase activity; P:phosphorelay signal trans EC:2.7.11; EC:2.7.13.3

F:protein kinase activity; C:cytoplasm; P:protein phosphorylation;

F:RNA binding; P:response to high light intensity; P:regulation of RNA splicing
F:RNA binding; P:response to high light intensity; P:regulation of RNA splicing

P:rRNA processing; C:integral component of membrane
P:metabolic process; F:trans-zeatin O-beta-D-glucosyltransferase a EC:2.4.1.203
F:DNA binding
C:integral component of membrane
C:integral component of membrane

F:glycerone kinase activity; F:ATP binding; P:glycerol metabolic pr EC:2.7.1.29
F:3'-5'-exoribonuclease activity; C:exosome (RNase complex); C:P- EC:3.1.13; EC:3.1.15
F:3'-5'-exoribonuclease activity; C:mitochondrion; P:rRNA process EC:3.1.13; EC:3.1.15; EC:3
C:chloroplast; C:integral component of membrane
C:chloroplast; C:integral component of membrane
F:RNA binding; C:nucleus; P:mRNA processing

C:integral component of membrane; F:transmembrane transporter activity;	
F:NAD+ kinase activity; P:NADP biosynthetic process; P:phosphory	EC:2.7.1.23
P:tRNA wobble uridine modification; F:flavin adenine dinucleotide binding	
F:protein kinase activity; F:ATP binding; P:protein phosphorylation	
F:protein kinase C activity; F:ATP binding; C:nucleus; C:ribosome;	EC:2.7.11; EC:2.7.11.13
F:serine-pyruvate transaminase activity; C:peroxisome;	EC:2.6.1.44; EC:2.6.1.51
F:serine-pyruvate transaminase activity; C:peroxisome;	EC:2.6.1.44; EC:2.6.1.51
C:integral component of membrane	
P:amino acid transmembrane transport;	
F:asparagine synthase (glutamine-hydrolyzing) activity;	EC:6.3.5.4
F:asparagine synthase (glutamine-hydrolyzing) activity;	EC:6.3.5.4
F:asparagine synthase (glutamine-hydrolyzing) activity;	EC:6.3.5.4
F:carbohydrate:proton symporter activity; F:glucose transmembrane transporter activity;	
F:RNA binding	
P:spliceosomal complex disassembly;	
P:spliceosomal complex disassembly;	
F:phosphoethanolamine N-methyltransferase activity;	EC:2.1.1.103
C:integral component of membrane; P:transmembrane transport	
C:integral component of membrane; F	
C:integral component of membrane	
P:glycolipid biosynthetic process; C:integral component of membrane;	
F:DNA binding; F:chromatin binding; C:nucleus	
F:branched-chain-amino-acid transaminase activity;	EC:2.6.1.42
F:protein serine/threonine phosphatase activity; C:nucleus;	EC:3.1.3.16; EC:3.1.3.41
C:integral component of membrane; F:N-acetylneuraminate 7-O(	EC:2.3.1.45
F:metal ion binding; F:desacetoxyvindoline 4-hydroxylase activity	EC:1.14.11; EC:1.14.11.2
F:phosphoethanolamine N-methyltransferase activity; C:integral c	EC:2.1.1.103
C:mitochondrial inner membrane; P:mitochondrial transport;	
F:phospholipid binding; C:cytosol; P:protein import into nucleus;	
F:phospholipid binding; C:cytosol; P:protein import into nucleus;	
F:lipid binding; C:integral component of membrane	
F:lipid binding; C:membrane; C:integral component of membrane	
F:translation initiation factor activity; C:transcription factor TFIID complex	
F:translation initiation factor activity; P:translational initiation;	
C:integral component of membrane; P:cellular response to stress	
F:DNA binding; F:ATP binding; P:DNA repair; F:DNA-dependent AT	EC:3.6.1.3; EC:3.6.1.15
F:3-hydroxyisobutyryl-CoA hydrolase activity	EC:3.1.2.4
C:nucleus	
F:methionine adenosyltransferase activity;	EC:2.5.1.6
C:extracellular region	
F:DNA binding; F:catalytic activity; P:base-excision repair;	
C:integral component of membrane	

F:chitinase activity; C:extracellular region;	EC:3.2.1.14
C:mitochondrion; C:peroxisome; P:response to oxidative stress;	
P:carbohydrate transport; C:integral component of Golgi membrane;	
F:inositol-polyphosphate 5-phosphatase activity; P:phosphatidylin	EC:3.1.3.56; EC:3.1.3.41
F:DNA-binding transcription factor activity; P:regulation of transcription, DNA-templated	
F:ATP binding; P:drug transmembrane transport;	EC:3.6.1.3; EC:3.6.3.44; E
F:nucleic acid binding; C:membrane; C:integral component of membrane	
F:Rho guanyl-nucleotide exchange factor activity; P:regulation of pollen tube growth	
F:glucose-fructose oxidoreductase activity; P:oxidation-reduction	EC:1.1.99.28
C:plasma membrane; P:response to oxidative stress;	
F:protein serine/threonine kinase activity; F:ATP binding; C:plasma	EC:2.7.11
F:nicotinate-nucleotide diphosphorylase (carboxylating) activity; P	EC:2.4.2.19
F:iron ion binding; C:integral component of membrane;	EC:1.14.13
P:glutamine metabolic process; P:folic acid-containing compound	EC:2.6.1.85
F:nuclease activity; P:cellular process	
F:nucleic acid binding	
F:transferase activity	
F:calcium ion binding; C:Golgi apparatus;	
C:cell; P:glycerol ether metabolic process;	
F:transporter activity; C:integral component of membrane	
F:ATP binding; F:1-phosphatidylinositol-4-phosphate 5-kinase acti	EC:2.7.1.68
C:anchored component of plasma membrane;	
F:protein serine/threonine kinase activity; F:ATP binding;	EC:2.7.11
C:integral component of membrane	
F:nucleic acid binding	
F:DNA-binding transcription factor activity; C:nucleus;	
F:RNA binding	
F:translation initiation factor activity; P:translational initiation	
F:GTP binding; C:intracellular	
P:maturation of SSU-rRNA from tricistronic rRNA transcript	
F:thioredoxin-disulfide reductase activity; C:cytoplasm;	EC:1.8.1.9; EC:1.8.1.8
F:protein serine/threonine kinase activity; F:ATP binding;	EC:2.7.11

F:protein kinase activity; F:ATP binding; P:protein phosphorylation;	
C:integral component of plasma membrane;	
C:1,3-beta-D-glucan synthase complex; F:1,3-beta-D-glucan syntha	EC:2.4.1.34
F:DNA binding; F:DNA-directed DNA polymerase activity;	EC:2.7.7.7
P:single strand break repair; F:double-stranded DNA binding;	
C:chloroplast envelope; P:chloroplast fission;	
C:cytosol; C:plasma membrane; P:proteolysis;	EC:3.4.11
P:vacuolar transport; C:endosome membrane;	
F:DNA binding; F:DNA-binding transcription factor activity;	
F:nucleosome binding; P:positive regulation of transcription elongation from RNA polymera	
F:nucleosome binding; P:positive regulation of transcription elongation from RNA polymera	
F:damaged DNA binding; C:nucleus; C:cytosol; P:nucleotide-excision repair	
F:acetate-CoA ligase activity; P:metabolic process; C:integral comp	EC:6.2.1.1
C:integral component of membrane	
F:DNA binding; C:nucleus; P:transcription, DNA-templated;	
P:lipid transport	
F:protein serine/threonine/tyrosine kinase activity; F:ATP binding;	EC:2.7.12.1
P:polyamine biosynthetic process; F:N-carbamoylputrescine amid	EC:3.5.1.53
P:putrescine biosynthetic process from arginine; F:N-carbamoylpu	EC:3.5.1.53
F:DNA binding	
P:amino acid transmembrane transport; C:plasma membrane;	
F:protein serine/threonine kinase activity; F:ATP binding;	EC:2.7.11
P:metabolic process; F:transferase activity, transferring hexosyl groups	
F:catalytic activity; P:metabolic process	
F:iron ion binding; P:lipid biosynthetic process;	
F:copper ion binding; C:cell; P:cellular copper ion homeostasis; F:zinc ion binding	
C:plasma membrane; F:acidic amino acid transmembrane transporter activity	
C:plasma membrane; F:acidic amino acid transmembrane transporter activity	
F:acetylglucosaminyltransferase activity; C:integral component of membrane	
C:plasma membrane; C:plant-type vacuole membrane;	
F:ligase activity	
F:DNA binding; C:nucleus; P:transcription, DNA-templated;	
F:protein serine/threonine kinase activity; F:ATP binding;	EC:2.7.11
F:catalytic activity; P:DNA repair; P:base-excision repair;	
C:integral component of membrane	
P:galactose metabolic process; F:inositol 3-alpha-galactosyltransfe	EC:2.4.1.123
F:magnesium ion binding; F:pyruvate kinase activity;	EC:2.7.1.40
P:dephosphorylation; F:N-acylneuraminate-9-phosphatase activity	EC:3.1.3.29; EC:3.1.3.41
F:L-aspartate:2-oxoglutarate aminotransferase activity;	EC:2.6.1.1

F:ATP binding; C:nucleus; P:transcription, DNA-templated;	
F:DNA binding; C:nucleus	
F:DNA binding; C:nucleus	
F:ATP binding; C:cell wall; C:mitochondrion;	
F:binding	
F:binding	
F:binding	
F:DNA binding; C:nucleus; P:regulation of transcription, DNA-templated;	
F:thiol-dependent ubiquitin-specific protease activity;	EC:3.4.19.12
F:thiol-dependent ubiquitin-specific protease activity;	EC:3.4.19.12
F:nucleotide binding; F:motor activity	EC:3.6.1.15
F:protein kinase activity; P:phosphorylation	
C:endoplasmic reticulum lumen; C:plasma membrane;	EC:3.1.1.11; EC:3.1.1.1
C:cell; P:cell redox homeostasis	
C:mitochondrial inner membrane; P:mitochondrial transport;	
F:RNA binding	
F:protein serine/threonine kinase activity; F:ATP binding;	EC:2.7.11
F:ATP binding; C:integral component of membrane; F:transferase activity; F:ligase activity	
F:RNA binding; F:translation initiation factor activity; P:translational initiation	
F:monooxygenase activity; F:iron ion binding;	
F:ATP binding; C:integral component of membrane; F	EC:3.6.1.3; EC:3.6.1.15
F:DNA-directed 5'-3' RNA polymerase activity; C:RNA polymerase I	EC:2.7.7.6
C:membrane; C:cell part	
P:nuclear-transcribed mRNA catabolic process, nonsense-mediated decay;	
C:Golgi apparatus; C:integral component of membrane; F:O-acetyltransferase activity;	
C:Golgi apparatus; C:integral component of membrane; F:O-acetyltransferase activity;	
C:Golgi apparatus; C:integral component of membrane; F:O-acetyltransferase activity;	
F:nucleic acid binding; C:nucleus	
F:nucleic acid binding; C:nucleus; C:cytosol	
F:metal ion binding	
F:actin binding	
P:branched-chain amino acid biosynthetic process; F:L-leucine tra	EC:2.6.1.42
F:calcium ion binding; C:integral component of membrane	
C:integral component of plasma membrane; P:water transport;	
F:metalloendopeptidase activity; F:ATP binding; P:proteolysis;	EC:3.4.24
F:poly(A)-specific ribonuclease activity; P:RNA phosphodiester bo	EC:3.1.13; EC:3.1.15; EC:3
F:voltage-gated chloride channel activity; F:nitrate:proton symporter activity;	
F:RNA binding	

C:cytoplasm; P:metal ion transport; F:transition metal ion binding;	
F:calcium ion binding; C:photosystem II oxygen evolving complex;	
F:protein binding; F:transferase activity	
F:nucleic acid binding; F:RNA-DNA hybrid ribonuclease activity;	
C:integral component of membrane	
F:3-oxoacyl-[acyl-carrier-protein] synthase activity; P:fatty acid bic	EC:2.3.1.41; EC:2.3.1.85;
F:RNA binding; F:ATP-dependent RNA helicase activity; F:ATP bind	EC:3.6.1.3; EC:3.6.1.15
F:DNA binding; C:nucleus; P:transcription, DNA-templated;	
C:nucleus; P:regulation of transcription, DNA-templated;	
C:nucleus; P:regulation of transcription, DNA-templated;	
F:translation initiation factor activity; P:translational initiation;	
P:drug transmembrane transport; F:drug transmembrane transporter activity; F	
F:protein dimerization activity	
F:3-hydroxyisobutyryl-CoA hydrolase activity;	EC:3.1.2.4; EC:4.2.1.17
F:GTPase activity; F:protein binding; F:GTP binding; C:nucleus; C:c	EC:3.6.1.15
F:DNA binding; C:nucleus; P:regulation of transcription, DNA-templated	
C:nucleosome; C:nuclear chromatin; F:DNA binding; P:	
C:nucleosome; F:DNA binding; C:nucleus	
F:RNA binding	
C:mitochondrion	
C:mitochondrion	
C:mitochondrion	
C:mitochondrion	
F:thiol-dependent ubiquitin-specific protease activity;	EC:3.4.19.12
F:nucleic acid binding; F:asparagine-tRNA ligase activity;	EC:6.1.1.22
F:protein kinase activity; P:protein phosphorylation	
P:autophagy of mitochondrion; C:cytosol; P:protein lipidation;	
C:nuclear pore; P:nucleocytoplasmic transport; F:structural constituent of nuclear pore	
C:membrane	
F:ATP binding; P:protein folding; F:2-alkenal reductase [NAD(P)] ac	EC:1.3.1.74
F:indole-3-glycerol-phosphate synthase activity; P:tryptophan met	EC:4.1.1.48
F:RNA-directed DNA polymerase activity; P:RNA-dependent DNA b	EC:2.7.7.49
F:transferase activity, transferring acyl groups; F:hydrolase activity	
P:metabolic process; C:membrane; F:hydrolase activity, acting on glycosyl bonds	
P:histone deacetylation	
F:catalytic activity	

P:mRNA splicing, via spliceosome; C:spliceosomal complex;	
F:inositol-polyphosphate 5-phosphatase activity;	EC:3.1.3.56; EC:3.1.3.41
P:ubiquitin-dependent protein catabolic process;	EC:3.4.19.12
C:endosome; C:trans-Golgi network; P:carbohydrate transport;	
P:fucose metabolic process; C:integral component of membrane;	EC:2.4.1.221
F:transaminase activity; F:4-amino-4-deoxychorismate lyase activi	EC:4.1.3.38
F:DNA (6-4) photolyase activity	EC:4.1.99; EC:4.1.99.13
P:phenylpropanoid biosynthetic process;	EC:1.2.1.68; EC:1.2.1.5
P:phenylpropanoid biosynthetic process; F:coniferyl-aldehyde deh	EC:1.2.1.68; EC:1.2.1.5
F:single-stranded DNA binding; F:ATP binding; P:DNA repair;	EC:3.6.1.3; EC:3.6.1.8; EC
F:alpha-1,2-mannosyltransferase activity; C:endoplasmic reticulum membrane;	
F:ADP binding	
F:RNA binding; C:nucleolus; C:integral component of membrane;	
F:channel activity; C:integral component of membrane;	
P:flavonoid biosynthetic process; F:naringenin-chalcone synthase	EC:2.3.1.74
F:chromatin binding; C:plasmodesma	
P:metabolic process; F:ligase activity	
P:regulation of transcription, DNA-templated; C:mediator complex	
F:malate dehydrogenase (decarboxylating) (NAD+) activity;	EC:1.1.1.38; EC:1.1.1.39
C:membrane	
F:1-phosphatidylinositol 4-kinase activity; C:intracellular;	EC:2.7.1.67
F:1-phosphatidylinositol 4-kinase activity; C:intracellular;	EC:2.7.1.67
C:mitochondrion; P:carbohydrate metabolic process;	EC:1.1.1.37; EC:1.1.1.82
C:mitochondrion; F:electron transfer activity; C:chloroplast;	
F:NADH dehydrogenase activity; F:NAD(P)H dehydrogenase (quinc	EC:1.6.99.3; EC:1.6.5.2
F:nucleic acid binding; F:helicase activity; F:ATP binding	EC:3.6.1.15
F:G-protein coupled receptor binding; F:GTPase activity;	EC:3.6.1.15
F:protein kinase activity; F:ATP binding; P:protein phosphorylation;	
F:transporter activity; P:sodium ion transport;	
C:nucleosome; F:DNA binding; C:nucleus;	
C:heterotrimeric G-protein complex; P:G-protein coupled receptor signaling pathway;	
F:aralkylamine N-acetyltransferase activity; C:nucleus; C	EC:2.3.1.87; EC:2.3.1.5
F:isomerase activity	
F:isomerase activity	
F:sucrose alpha-glucosidase activity; P:starch metabolic process;	EC:3.2.1.97; EC:3.2.1.20;
P:ubiquitin-dependent protein catabolic process; F:ubiquitin protein ligase binding	
P:ubiquitin-dependent protein catabolic process	

F:magnesium ion binding; F:inorganic diphosphatase activity;	EC:3.6.1.1
P:protein peptidyl-prolyl isomerization; F:peptidyl-prolyl cis-trans	EC:5.2.1.8
F:DNA binding; P:lipid metabolic process; C:integral component of	EC:1.14.19.3; EC:1.14.19
C:membrane; F:hydrolase activity, acting on glycosyl bonds	
F:beta-glucuronidase activity; P:metabolic process; C:membrane	EC:3.2.1.31
C:integral component of membrane; F:1-acylglycerophosphocholi	EC:2.3.1.23
F:RNA polymerase II regulatory region sequence-specific DNA binc	EC:2.4.1.12
F:ATP binding; P:pyruvate metabolic process; F:kinase activity;	EC:2.7.9.1
F:ATP binding; P:pyruvate metabolic process; F:kinase activity;	EC:2.7.9.1
F:kinase activity	
F:DNA-binding transcription factor activity; C:nucleus;	
F:nucleic acid binding; C:nucleus; F:zinc ion binding; P:DNA integration	
C:plasma membrane; C:integral component of membrane	
C:integral component of membrane	
F:protein serine/threonine kinase activity; F:ATP binding; C:nucleu	EC:2.7.11
P:metabolic process; F:oxidoreductase activity	
F:aldehyde dehydrogenase (NAD) activity; C:vacuole;	EC:1.2.1.3
F:thioredoxin-disulfide reductase activity; C:cytoplasm;	EC:1.8.1.9; EC:1.8.1.8
F:monooxygenase activity; F:iron ion binding; C:integral component of membrane;	
C:chloroplast thylakoid membrane;	
C:chloroplast thylakoid membrane;	
F:serine-type endopeptidase activity; P:p	EC:3.4.21
C:integral component of membrane	
P:carbohydrate metabolic process;	EC:3.2.1.21; EC:3.2.1.39
P:cellular process; C:integral component of membrane;	
C:chloroplast envelope; C:membrane	
F:DNA binding; C:nucleus; P:transcription, DNA-templated; P:regulation of transcription, DN	
P:dephosphorylation; F:2-carboxy-D-arabinitol-1-phosphatase acti	EC:3.1.3.63; EC:3.1.3.41
F:ATP binding; C:mitochondrion; C:cytosol; P:'de novo' protein folding;	
F:ATP binding; C:cytoplasm; P:ubiquitin-dependent protein catabolic process;	
F:oxidoreductase activity; P:oxidation-reduction process	
F:triose-phosphate isomerase activity; C:cytosol; P:gluconeogenes	EC:5.3.1.1
F:triose-phosphate isomerase activity; C:mitochondrion; C:cytosol	EC:5.3.1.1
P:regulation of cellular process	
C:cytoplasm; F:monodehydroascorbate reductase (NADH) activity	EC:1.6.5.4
P:glycerol-3-phosphate metabolic process; C:glycerol-3-phosphate	EC:1.1.5.3
C:extracellular region	
P:defense response to oomycetes; P:response to fungus; P:respon	EC:1.14.11; EC:1.14.11.9;
P:response to cold; C:photosystem II; C:chloroplast thylakoid membrane;	
P:response to cold; C:photosystem II; C:chloroplast thylakoid membrane;	
F:RNA polymerase II transcription factor activity, sequence-specific DNA binding;	

C:intracellular	
C:intracellular	
P:nuclear-transcribed mRNA catabolic process, nonsense-mediate	EC:3.6.1.15
C:integral component of membrane	
F:copper ion binding; C:cytoplasm; C:plasma membrane; C:plasmodesma;	
F:copper ion binding; C:cytoplasm; C:plasma membrane; C:plasmodesma;	
F:protein serine/threonine kinase activity; F:ATP binding; P:protei	EC:2.7.11
P:auxin-activated signaling pathway; P:phloem or xylem histogenesis;	
C:membrane; C:integral component of membrane	
C:endosome; C:cytosol; P:intracellular protein transport;	
C:cytosol; P:response to absence of light; P:response to carbon starvation	
P:polysaccharide catabolic process; F:beta-amylase activity; F:amy	EC:3.2.1.2
P:polysaccharide catabolic process; F:beta-amylase activity; F:amy	EC:3.2.1.2
P:polysaccharide catabolic process; F:beta-amylase activity;	EC:3.2.1.2
C:nucleus; C:cytoplasm	
F:transferase activity	
C:integral component of membrane	
C:nucleus; P:cytokinin biosynthetic process; F:hydrolase activity	
F:DNA-binding transcription factor activity; P:regulation of transcription, DNA-templated	
F:DNA-binding transcription factor activity; P:regulation of transcription, DNA-templated	
F:ATP binding; P:mismatch repair; F:mismatched DNA binding	
C:endoplasmic reticulum; P:lipid metabolic process; P:wax biosynt	EC:1.2.1.84
F:glutathione transferase activity; F:lyase activity	EC:2.5.1.18
F:phosphatidylcholine 1-acylhydrolase activity; C:integral compon	EC:3.1.1.32; EC:3.1.1.1
F:phosphatidylcholine 1-acylhydrolase activity; C:integral compon	EC:3.1.1.32; EC:3.1.1.1
F:binding	
F:thioredoxin-disulfide reductase activity; C:cell; F:electron transfe	EC:1.8.1.9
F:DNA binding	
F:DNA binding	
F:nicotianamine synthase activity; P:nicotianamine biosynthetic p	EC:2.5.1.43
P:inositol biosynthetic process; F:inositol monophosphate 1-phos	EC:3.1.3.25; EC:3.1.3.41
F:ATP binding; F:transferase activity	
P:carbohydrate metabolic process; F:glycogen phosphorylase acti	EC:2.4.1.1
C:cytoplasm; P:glycogen catabolic process; F:glycogen phosphoryl	EC:2.4.1.1
C:ESCRT I complex; P:protein transport; P:endosome transport via multivesicular body sorti	
F:calcium-transporting ATPase activity; F:calmodulin binding; F:AT	EC:3.6.1.3; EC:3.6.3.8; EC
P:retrograde vesicle-mediated transport, Golgi to ER; C:integral component of membrane	
C:cytoplasm; P:carbohydrate metabolic process; C:integral compo	EC:4.1.2.53
C:P-body; F:nucleic acid binding; F:poly(A)-specific ribonuclease a	EC:3.1.13; EC:3.1.15; EC:
F:RNA binding; F:zinc ion binding	
C:integral component of membrane	
F:nucleic acid binding; P:RNA processing; F:endoribonuclease acti	EC:3.1.30; EC:3.1.26
F:nucleic acid binding; P:DNA repair; F:nucleotidyltransferase activity	

F:nucleic acid binding; P:gene silencing by RNA	
F:nucleic acid binding; P:gene silencing by RNA	
F:zinc ion binding	
P:chromatin remodeling; C:Ino80 complex	
P:chromatin remodeling; C:Ino80 complex	
C:Golgi membrane; F:magnesium ion binding; F:phospholipid-tran	EC:3.6.1.3; EC:3.6.3.1; EC
C:integral component of membrane	
C:photosystem I; C:photosystem II; C:chloroplast thylakoid membrane;	
C:photosystem I; C:photosystem II; C:chloroplast thylakoid membrane;	
F:peptidase activity	
C:nucleus; P:regulation of transcription, DNA-templated	
C:cytosol; C:chloroplast thylakoid membrane; C:NAD(P)H dehydrogenase complex	
P:drug transmembrane transport; F:drug transmembrane transporter activity;	
C:chloroplast; F:zeaxanthin epoxidase [overall] activity; P:abscisic	EC:1.14.13.90; EC:1.14.13
C:membrane	
C:integral component of membrane; F:protein-lysine N-methyltransferase activity;	
F:DNA binding; F:helicase activity; F:ATP binding; C:NURF complex	EC:3.6.1.3; EC:3.6.1.15
C:cytoplasm; P:metal ion transport; F:transition metal ion binding;	
F:protease binding; F:ATP binding; C:cell wall; C:nucleolus;	
F:ATP binding	
C:integral component of plasma membrane; P:water transport;	
F:3-dehydroquinate dehydratase activity; F:shikimate 3-dehydrog	EC:1.1.1.25; EC:4.2.1.10;
C:endoplasmic reticulum; C:Golgi apparatus; P:GPI anchor biosynthetic process;	
P:dephosphorylation; F:pyridoxal phosphatase activity	EC:3.1.3.41; EC:3.1.3.74
F:oxidoreductase activity; P:oxidation-reduction process	
P:autophagosome assembly; C:phagophore assembly site	
F:phosphorelay sensor kinase activity; P:phosphorelay signal trans	EC:2.7.13.3
F:ATP binding; F:ATPase-coupled sulfate transmembrane transpor	EC:3.6.1.3; EC:3.6.3.25; E
C:nucleolus	
F:ATP binding; F:ATPase-coupled sulfate transmembrane transpor	EC:3.6.1.3; EC:3.6.3.25; E
P:metabolic process; F:oxalate-CoA ligase activity	EC:6.2.1.8
F:cysteine-type endopeptidase activity; C:vacuole; P:vacuolar prot	EC:3.4.22
F:protein kinase activity; C:intracellular; P:protein phosphorylation;	
F:nucleotide binding; F:copper-exporting ATPase activity;	EC:3.6.1.3; EC:3.6.1.15; E
F:hydroxypyruvate reductase activity; F:NAD binding; P:oxidation-	EC:1.1.1.81
C:integral component of endoplasmic reticulum membrane;	
C:integral component of membrane; P:purine nucleotide-sugar transmembrane transport	
F:protein serine/threonine kinase activity; F:ATP binding; P:protei	EC:2.7.11

F:DNA binding; C:integral component of membrane	
F:copper ion binding; F:L-ascorbate oxidase activity; P:oxidation-re	EC:1.10.3; EC:1.10.3.3
F:copper ion binding; F:L-ascorbate oxidase activity; P:oxidation-re	EC:1.10.3; EC:1.10.3.3
F:nucleotide binding; F:microtubule motor activity; C:kinesin comp	EC:3.6.1.3; EC:3.6.1.15
F:metal ion binding	
F:calcium ion binding	
F:ATP binding; C:chloroplast thylakoid membrane; P:ATP synthesis	EC:3.6.1.3; EC:3.6.1.15
F:ATP binding	
F:methyltransferase activity; P:methylation	
F:DNA binding; C:nucleus; F:metal ion binding	
F:DNA binding; C:nucleus; F:metal ion binding	
F:nucleic acid binding; F:helicase activity; F:ATP binding	EC:3.6.1.15
F:ATP binding; F:ligase activity; P:protein sumoylation; F:SUMO transferase activity	
F:GTP binding	
F:GTP binding	
F:zinc ion transmembrane transporter activity; C:plasma membrane;	
F:DNA-binding transcription factor activity; P:DNA-templated transcription,	
C:nuclear chromosome	
C:nuclear chromosome	
F:protein kinase activity; F:ATP binding; P:protein phosphorylation	EC:3.6.3.46
P:Group II intron splicing; F:mRNA binding; F:ATP-dependent RNA	EC:3.6.1.3; EC:3.6.1.15
P:Group II intron splicing; F:mRNA binding; F:ATP-dependent RNA	EC:3.6.1.3; EC:3.6.1.15
F:protein dimerization activity	
C:integral component of membrane	
F:phosphoprotein phosphatase activity; F:cation binding	EC:3.1.3.16; EC:3.1.3.41
F:RNA binding; C:integral component of membrane	
C:Golgi membrane; P:protein glycosylation; F:galactosyltransferase activity;	
F:protochlorophyllide reductase activity; P:oxidation-reduction pr	EC:1.3.1.33
F:NAD+ kinase activity; F:diacylglycerol kinase activity; F:ATP bindi	EC:2.7.1.23; EC:2.7.1.107
F:thioredoxin-disulfide reductase activity; C:cytoplasm; P:intracell	EC:1.8.1.9; EC:1.8.1.8
F:ATP-dependent peptidase activity; F:ATP binding; C:mitochondri	EC:3.6.1.3; EC:3.6.1.15
F:protein serine/threonine kinase activity; F:ATP binding; P:protei	EC:2.7.11
F:protein dimerization activity	
F:ATP binding; C:plasma membrane; C:integral component of mem	EC:3.6.1.3; EC:3.6.1.15
F:ATP binding; C:plasma membrane; C:integral component of mem	EC:3.6.1.3; EC:3.6.1.15
F:zinc ion binding; C:integral component of membrane	
P:spliceosomal complex assembly; C:Prp19 complex;	
F:CDP-diacylglycerol-serine O-phosphatidyltransferase activity;	EC:2.7.8.8
F:CDP-diacylglycerol-serine O-phosphatidyltransferase activity;	EC:2.7.8.8
F:carbonate dehydratase activity; F:zinc ion binding; P:carbon utili	EC:4.2.1.1
F:UDP-glucose 4-epimerase activity; P:galactose metabolic proces	EC:5.1.3.2
P:maturation of SSU-rRNA from tricistronic rRNA transcript	
P:maturation of SSU-rRNA from tricistronic rRNA transcript	

F:3-dehydroquinate dehydratase activity; F:shikimate 3-dehydrog	EC:1.1.1.25; EC:4.2.1.10;
C:extracellular region	
F:magnesium ion binding; C:cytoplasm; F:zinc ion binding;	EC:4.2.1.109; EC:3.1.3.77
F:magnesium ion binding; C:cytoplasm; F:zinc ion binding	EC:4.2.1.109; EC:3.1.3.77
C:membrane; C:integral component of membrane	
P:phosphate-containing compound metabolic process;	
P:phosphate-containing compound metabolic process;	
P:phosphate-containing compound metabolic process;	
P:phosphate-containing compound metabolic process;	
P:phosphate-containing compound metabolic process;	
P:phosphate-containing compound metabolic process;	
P:phosphate-containing compound metabolic process;	
P:phosphate-containing compound metabolic process;	EC:3.5.1.98
P:phosphate-containing compound metabolic process;	EC:3.5.1.98
P:phosphate-containing compound metabolic process;	EC:6.2.1.3
P:phosphate-containing compound metabolic process;	EC:6.2.1.3
F:structural constituent of ribosome; C:ribosome; P:translation	
P:MAPK cascade; F:MAP kinase activity; F:ATP binding; C:nucleus	EC:2.7.11; EC:2.7.11.24
F:nucleic acid binding	
F:aldose-6-phosphate reductase (NADPH) activity; P:oxidation-red	EC:1.1.1.200
C:cytosol; P:response to cadmium ion; F:aldose-6-phosphate redu	EC:1.1.1.200
C:cytosol; P:response to cadmium ion; F:aldose-6-phosphate redu	EC:1.1.1.200
C:proteasome complex; F:ATP binding; C:chloroplast stroma; C:thylakoid;	
F:ATP binding	
F:phosphopantothenoylcysteine decarboxylase activity; F:ATP bin	EC:4.1.1.36
F:ATP binding	
P:calcium ion transport; F:cation transmembrane transporter activity;	
F:transferase activity, transferring acyl groups	
F:fructose-bisphosphate aldolase activity; P:glycolytic process	EC:4.1.2.13
C:integral component of membrane	
C:membrane; F:kinase activity; P:phosphorylation	
F:protein serine/threonine kinase activity; F:ATP binding; P:protei	EC:2.7.11
F:lipid binding; C:membrane; C:integral component of membrane	
F:lipid binding; C:membrane; C:integral component of membrane	
F:nucleotide binding; F:ATP binding; C:plasma membrane; P:ion transport;	
F:serine-tRNA ligase activity; F:ATP binding; C:cytoplasm; P:seryl-t	EC:6.1.1.11
P:proteolysis; F:metallopeptidase activity; P:response to heat;	
P:proteolysis; F:metallopeptidase activity; P:response to heat;	
P:proteolysis; F:metallopeptidase activity; P:response to heat;	
F:spermidine synthase activity; P:polyamine biosynthetic process;	EC:2.5.1.16; EC:2.5.1.79
F:glycine hydroxymethyltransferase activity; C:nucleus; C:mitocho	EC:2.1.2.1
C:exocyst; C:cytosol; C:plasma membrane; P:protein targeting to membrane	

F:monooxygenase activity; F:iron ion binding; P:lipid biosynthetic process;

F:RNA binding; C:nucleus; P:mRNA processing; P:RNA splicing
C:vacuolar membrane; C:plasma membrane; P:regulation of pH;
F:double-stranded DNA binding; F:single-stranded RNA binding;
F:transcription coregulator activity; P:regulation of transcription, DNA-templated
F:NADPH-hemoprotein reductase activity; C:endoplasmic reticulu EC:1.6.2.4
F:binding
P:regulation of transcription, DNA-templated

F:phosphatidylinositol phospholipase C activity; P:lipid metabolic EC:3.1.4.11; EC:3.1.4.3

Enzyme Names InterPro IDs InterPro GO IDs

Histidine kinase
Adenosinetriphosphatase; Nucleoside-triphosphate phosphatase
Adenosinetriphosphatase; Nucleoside-triphosphate phosphatase

Malate dehydrogenase (oxaloacetate-decarboxylating); Malate dehydrogenase (decarboxylating); Mal
Carbamoyl-phosphate synthase (glutamine-hydrolyzing)
Histidine kinase

Polygalacturonate 4-alpha-galacturonosyltransferase
Long-chain-fatty-acid--CoA ligase
Long-chain-fatty-acid--CoA ligase

Cinnamoyl-CoA reductase
Arylamine N-acetyltransferase; Histone acetyltransferase

Protein-serine/threonine phosphatase; 4-nitrophenylphosphatase

2-alkenal reductase (NAD(P)(+))
Acting on peptide bonds (peptidases)

[illegible]; F:RNA binding; C:U2 snRNP; C:U2-type prespliceosome; C:catalytic step 2 spliceosome

Histidine kinase

Transferring phosphorus-containing groups; Mitogen-activated protein kinase

N(6)-L-threonylcarbamoyladenine synthase

Adenosinetriphosphatase; Nucleoside-triphosphate phosphatase

Adenosinetriphosphatase; Nucleoside-triphosphate phosphatase; Plus-end-directed kinesin ATPase

Acting on peptide bonds (peptidases); Acting on peptide bonds (peptidases)
Alcohol-forming fatty acyl-CoA reductase

Transferring phosphorus-containing groups
Histidine--tRNA ligase

Fructose-bisphosphate aldolase

Beta-glucosidase

Peptidylprolyl isomerase

Adenosinetriphosphatase; Nucleoside-triphosphate phosphatase

Pantothenate kinase

Nitrate reductase (NADH); Nitrate reductase (NADPH); Nitrate reductase

Glycerol-3-phosphate dehydrogenase (NAD(+)); Glycerol-3-phosphate dehydrogenase (NAD(P)(+))
Adenosinetriphosphatase; Nucleoside-triphosphate phosphatase

Acting on single donors with incorporation of molecular oxygen (oxygenases).

Sucrose synthase

Dimethylglycine N-methyltransferase; Glycine/sarcosine/dimethylglycine N-methyltransferase;

Histidine kinase

Histidine kinase

Beta-mannosidase; Mannan endo-1,4-beta-mannosidase

Sedoheptulose-bisphosphatase; 4-nitrophenylphosphatase

2-alkenal reductase (NAD(P)(+))

Transferring phosphorus-containing groups; Cyclin-dependent kinase

Phospholipase A(1); Carboxylesterase
Phospholipase A(1); Carboxylesterase
Cystathionine gamma-synthase

Adenosylmethionine decarboxylase

UDP-N-acetylglucosamine diphosphorylase

Nucleoside-phosphate kinase; Adenylate kinase
Nucleoside-phosphate kinase; Adenylate kinase

Transferring phosphorus-containing groups
4-nitrophenylphosphatase; Acid phosphatase
Glycosyltransferases; Beta-galactoside alpha-2,3-sialyltransferase

4-alpha-glucanotransferase

Alpha-1,3-mannosyl-glycoprotein 2-beta-N-acetylglucosaminyltransferase
Alpha-1,3-mannosyl-glycoprotein 2-beta-N-acetylglucosaminyltransferase
Ubiquitinyl hydrolase 1

3-beta-hydroxy-Delta(5)-steroid dehydrogenase;

Transferring phosphorus-containing groups

Acting on peptide bonds (peptidases)
Acting on paired donors, with incorporation or reduction of molecular oxygen.

Mitogen-activated protein kinase kinase kinase; Transferring phosphorus-containing groups

Nucleoside-triphosphate phosphatase

Transferring phosphorus-containing groups

Acetyl-CoA C-acyltransferase; Acetyl-CoA C-acetyltransferase
Acetyl-CoA C-acyltransferase; Acetyl-CoA C-acetyltransferase
Protein-serine/threonine phosphatase; 4-nitrophenylphosphatase

4-nitrophenylphosphatase; 5'-nucleotidase; Nucleotidase
4-nitrophenylphosphatase; 5'-nucleotidase; Nucleotidase

Sugar-phosphatase; Fructose-2,6-bisphosphate 2-phosphatase; 4-nitrophenylphosphatase

Transferring phosphorus-containing groups

Fatty-acid synthase; 3-oxoacyl-[acyl-carrier-protein] reductase

Polygalacturonase

Adenosinetriphosphatase; Nucleoside-triphosphate phosphatase

Beta-amylase

Mannosyl-oligosaccharide glucosidase; Raucaffricine beta-glucosidase

Phosphoribosyl 1,2-cyclic phosphate 1,2-diphosphodiesterase

Adenosinetriphosphatase; Nucleoside-triphosphate phosphatase
Adenosinetriphosphatase; Nucleoside-triphosphate phosphatase

Acting on paired donors, with incorporation or reduction of molecular oxygen.
Methionine--tRNA ligase

Thioredoxin-disulfide reductase

Transferring phosphorus-containing groups

Dual-specificity kinase

Acting on single donors with incorporation of molecular oxygen (oxygenases).
Pyruvate kinase
DNA ligase (ATP)
Shikimate O-hydroxycinnamoyltransferase

Shikimate dehydrogenase; 3-dehydroquinate dehydratase; Quinate/shikimate dehydrogenase

Protein-serine/threonine phosphatase; 4-nitrophenylphosphatase

Carboxylesterase; Phospholipase A(2)

Histidinol-phosphate transaminase
Acting on paired donors, with incorporation or reduction of molecular oxygen.
N-acetyl-gamma-glutamyl-phosphate reductase

Transferring phosphorus-containing groups; Cyclin-dependent kinase
mRNA (guanine-N(7)-)-methyltransferase

Cellulose synthase (UDP-forming)

1,3-beta-glucan synthase

Alcohol dehydrogenase

Histone-lysine N-methyltransferase
Mannosyl-oligosaccharide 1,2-alpha-mannosidase; Alpha-mannosidase
Mannosyl-oligosaccharide 1,2-alpha-mannosidase; Alpha-mannosidase

4-nitrophenylphosphatase; 5'-nucleotidase; Nucleotidase

Alanine--tRNA ligase
Sedoheptulose-bisphosphatase; 4-nitrophenylphosphatase
Sedoheptulose-bisphosphatase; 4-nitrophenylphosphatase

Geraniol 8-hydroxylase; Acting on paired donors, with incorporation or reduction of molecular oxygen
Geraniol 8-hydroxylase; Acting on paired donors, with incorporation or reduction of molecular oxygen
otosystem II assembly

Hydroxymethylglutaryl-CoA reductase (NADPH)

Adenosinetriphosphatase; Xenobiotic-transporting ATPase; Nucleoside-triphosphate phosphatase
Inositol-phosphate phosphatase; 4-nitrophenylphosphatase
Acting on peptide bonds (peptidases)
Acting on peptide bonds (peptidases)

Polygalacturonate 4-alpha-galacturonosyltransferase

Protein-serine/threonine phosphatase; Phosphoserine phosphatase; 4-nitrophenylphosphatase

Glutaminyl-tRNA synthase (glutamine-hydrolyzing)

Long-chain-fatty-acid--CoA ligase

Nucleoside-triphosphate phosphatase

Palmitoyl-protein hydrolase; Lysophospholipase; Carboxylesterase
Alpha-amylase
Adenosinetriphosphatase; Nucleoside-triphosphate phosphatase

DNA-directed RNA polymerase

Proline--tRNA ligase

Fructose-bisphosphate aldolase
Fructose-bisphosphate aldolase

Phospho-N-acetylmuramoyl-pentapeptide-transferase; UDP-N-acetylglucosamine

Glutathione transferase

Arylamine N-acetyltransferase; Histone acetyltransferase

Glutathione-disulfide reductase
Adenosinetriphosphatase; Nucleoside-triphosphate phosphatase
Alpha-galactosidase

Deoxyribodipyrimidine photo-lyase; Carbon-carbon lyases
Deoxyribodipyrimidine photo-lyase; Carbon-carbon lyases

Phospholipase C
Transferring phosphorus-containing groups

Adenosinetriphosphatase; Nucleoside-triphosphate phosphatase

6-phosphofructo-2-kinase
Nucleoside-triphosphate phosphatase

Glutamate synthase (NADH)
Acting on paired donors, with incorporation or reduction of molecular oxygen.
Acting on paired donors, with incorporation or reduction of molecular oxygen.

Acting on paired donors, with incorporation or reduction of molecular oxygen.
Acting on paired donors, with incorporation or reduction of molecular oxygen.

Tetrahydrofolate synthase; Dihydrofolate synthase

Nucleoside-phosphate kinase; UMP/CMP kinase

Cytochrome-c peroxidase; Peroxidase

Methionine adenosyltransferase
Methionine adenosyltransferase

N-acylneuraminate-9-phosphatase; 4-nitrophenylphosphatase

Alpha-mannosidase
Acting on peptide bonds (peptidases)

Acting on peptide bonds (peptidases); Acting on peptide bonds (peptidases)
Acting on peptide bonds (peptidases); Acting on peptide bonds (peptidases)

NADH dehydrogenase
1-acylglycerol-3-phosphate O-acyltransferase

Adenosinetriphosphatase; Iron-chelate-transporting ATPase; Nucleoside-triphosphate phosphatase
Adenosinetriphosphatase; Iron-chelate-transporting ATPase; Nucleoside-triphosphate phosphatase
Methionine--tRNA ligase

Sucrose-phosphate synthase; Sucrose synthase; Glutamine N-phenylacetyltransferase

Acting on paired donors, with incorporation or reduction of molecular oxygen.
Acting on diphenols and related substances as donors; Laccase

NAD(P)H oxidase (H(2)O(2)-forming); Peroxidase
UTP-monosaccharide-1-phosphate uridylyltransferase; UTP--glucose-1-phosphate uridylyltransferase
Acting on peptide bonds (peptidases); Adenosinetriphosphatase;
Beta-glucosidase
Acting on paired donors, with incorporation or reduction of molecular oxygen.
Acting on paired donors, with incorporation or reduction of molecular oxygen.
Acting on paired donors, with incorporation or reduction of molecular oxygen.

3-hydroxyacyl-CoA dehydrogenase

Fructose-bisphosphate aldolase

4-nitrophenylphosphatase; Acid phosphatase

4-nitrophenylphosphatase; Acid phosphatase

3,8-divinyl protochlorophyllide a 8-vinyl-reductase (NADPH)

GTP diphosphokinase

Transferring phosphorus-containing groups
Strictosidine synthase

Branched-chain-amino-acid transaminase

Protein disulfide-isomerase
Nucleoside-phosphate kinase; Adenylate kinase

Acting on paired donors, with incorporation or reduction of molecular oxygen.

Transferring phosphorus-containing groups
Nucleoside-triphosphate phosphatase

DNA ligase (ATP)

Feruloyl esterase; Carboxylesterase

Glucose-6-phosphate 1-epimerase
Glucose-6-phosphate 1-epimerase

Non-reducing end alpha-L-arabinofuranosidase

Acting on peptide bonds (peptidases)

Glucose-1-phosphate adenylyltransferase
Transferring phosphorus-containing groups; Cyclin-dependent kinase
Carboxylesterase

1,3-beta-glucan synthase
1,3-beta-glucan synthase

Anthranilate phosphoribosyltransferase

Phosphoenolpyruvate carboxylase; Phosphoenolpyruvate carboxykinase (GTP)

Pyruvate decarboxylase

Peptidylprolyl isomerase

Phosphatidate phosphatase; 4-nitrophenylphosphatase

Histidine kinase

Protoporphyrin ferrochelatase
Nucleoside-triphosphate phosphatase
Nucleoside-triphosphate phosphatase

Phosphoribulokinase

Phosphoacetylglucosamine mutase

Acting on single donors with incorporation of molecular oxygen (oxygenases).

Phosphatidate phosphatase; 4-nitrophenylphosphatase

NADH dehydrogenase

Glyceraldehyde-3-phosphate dehydrogenase (NADP(+))

Acetate--CoA ligase

2-alkenal reductase (NAD(P)(+))
Malate dehydrogenase
Malate dehydrogenase
Glycerate dehydrogenase; Hydroxypyruvate reductase
Glycerate dehydrogenase; Hydroxypyruvate reductase

Primary-amine oxidase

Glucose-1-phosphate adenylyltransferase
Glucose-1-phosphate adenylyltransferase

1,3-beta-glucan synthase
1,3-beta-glucan synthase

Acting on peptide bonds (peptidases)
Magnesium chelatase
Sucrose-phosphate synthase; Sucrose synthase

Adenosinetriphosphatase; Peptide-transporting ATPase; Nucleoside-triphosphate phosphatase

Acting on peptide bonds (peptidases)
Peptidylprolyl isomerase

te binding

S-methyl-5-thioribose-1-phosphate isomerase

Phenylalanine--tRNA ligase

Phospholipase A(1); Carboxylesterase

Transferring phosphorus-containing groups
Adenosinetriphosphatase; Proton-exporting ATPase; Nucleoside-triphosphate phosphatase
Glycine dehydrogenase (aminomethyl-transferring)
Glycine dehydrogenase (aminomethyl-transferring)

Glycogen phosphorylase
Nucleoside-triphosphate phosphatase

Nucleoside-triphosphate phosphatase

Acting on peptide bonds (peptidases)

Adenosinetriphosphatase; Nucleoside-triphosphate phosphatase; Molybdate-transporting ATPase

Peroxidase

Glycerate dehydrogenase

Transferring phosphorus-containing groups

Acting on peptide bonds (peptidases)
Caffeoyl-CoA O-methyltransferase
Galactinol--raffinose galactosyltransferase; Galactinol--sucrose galactosyltransferase
Acting on peptide bonds (peptidases)

3-methyl-2-oxobutanoate dehydrogenase (2-methylpropanoyl-transferring)

Transferring phosphorus-containing groups
Transferring phosphorus-containing groups; Transferring phosphorus-containing groups;

Glycine hydroxymethyltransferase

Acetyl-CoA carboxylase
Protein-serine/threonine phosphatase; Protein-tyrosine-phosphatase;
Adenosinetriphosphatase; Nucleoside-triphosphate phosphatase

Protein-serine/threonine phosphatase; 4-nitrophenylphosphatase
Protein-serine/threonine phosphatase; 4-nitrophenylphosphatase

Acting on CH or CH(2) groups; Acting on CH or CH(2) groups
Adenosinetriphosphatase; Calcium-transporting ATPase; Nucleoside-triphosphate phosphatase

Acting on diphenols and related substances as donors; Plastoquinol--plastocyanin reductase

Adenosinetriphosphatase; Sulfate-transporting ATPase; Nucleoside-triphosphate phosphatase

Non-reducing end alpha-L-arabinofuranosidase

Adenosinetriphosphatase; Nucleoside-triphosphate phosphatase

Adenosinetriphosphatase; Proton-exporting ATPase; Nucleoside-triphosphate phosphatase

Sterol 3-beta-glucosyltransferase

Acting on paired donors, with incorporation or reduction of molecular oxygen.

Acting on peptide bonds (peptidases); Adenosinetriphosphatase;

Protein xylosyltransferase

Glutamate decarboxylase
Transferring phosphorus-containing groups

Glucose-6-phosphate isomerase
Acting on peptide bonds (peptidases)

Pullulanase; Limit dextrinase

1,4-alpha-glucan branching enzyme

Adenosinetriphosphatase; Nucleoside-triphosphate phosphatase

(S)-2-hydroxy-acid oxidase

Transferring phosphorus-containing groups

Peptidylprolyl isomerase

DNA-directed RNA polymerase

Carbonic anhydrase

Inositol 3-alpha-galactosyltransferase
Inositol 3-alpha-galactosyltransferase
Adenosinetriphosphatase; Nucleoside-triphosphate phosphatase

Cytochrome-c oxidase

Acting on peptide bonds (peptidases)
GDP-mannose 3,5-epimerase

Protein-serine/threonine phosphatase; 4-nitrophenylphosphatase
ation

Transferring phosphorus-containing groups; Transferring phosphorus-containing groups;

N-acetylglucosaminylphosphatidylinositol deacetylase

Glycogen phosphorylase
Bilirubin oxidase
Bilirubin oxidase

Alpha-glucosidase; Beta-fructofuranosidase; Sucrose alpha-glucosidase
Alpha-glucosidase; Beta-fructofuranosidase; Sucrose alpha-glucosidase
Acting on paired donors, with incorporation or reduction of molecular oxygen.

Transferring phosphorus-containing groups

Nucleoside-triphosphate phosphatase

Cinnamoyl-CoA reductase; 3-beta-hydroxy-Delta(5)-steroid dehydrogenase
Cinnamoyl-CoA reductase

Beta-glucosidase; Glucan endo-1,3-beta-D-glucosidase

DNA ligase (ATP)

Acting on the CH-CH group of donors
Acting on the CH-CH group of donors

Transferring phosphorus-containing groups
Transferring phosphorus-containing groups

Protein-serine/threonine phosphatase; 4-nitrophenylphosphatase
A)
Feruloyl esterase; Carboxylesterase
Nitrate reductase (NADH); Nitrate reductase (NADPH); Nitrate reductase

Galactinol--sucrose galactosyltransferase

tRNA (N(6)-L-threonylcarbamoyladenosine(37)-C(2))-methylthiotransferase

Acting on iron-sulfur proteins as donors; Ferredoxin--NADP(+) reductase
Sugar-phosphatase; Fructose-bisphosphatase; 4-nitrophenylphosphatase
Sugar-phosphatase; Fructose-bisphosphatase; 4-nitrophenylphosphatase
Sugar-phosphatase; Fructose-bisphosphatase; 4-nitrophenylphosphatase

Transferring phosphorus-containing groups; Histidine kinase

Trans-zeatin O-beta-D-glucosyltransferase

Glycerone kinase
Acting on ester bonds; Acting on ester bonds
Acting on ester bonds; Acting on ester bonds; Exoribonuclease II

NAD(+) kinase

Transferring phosphorus-containing groups; Protein kinase C

Alanine--glyoxylate transaminase; Serine--pyruvate transaminase
Alanine--glyoxylate transaminase; Serine--pyruvate transaminase

Asparagine synthase (glutamine-hydrolyzing)
Asparagine synthase (glutamine-hydrolyzing)
Asparagine synthase (glutamine-hydrolyzing)

Phosphoethanolamine N-methyltransferase

Branched-chain-amino-acid transaminase
Protein-serine/threonine phosphatase; 4-nitrophenylphosphatase
N-acetylneuraminate 7-O(or 9-O)-acetyltransferase

Acting on paired donors, with incorporation or reduction of molecular oxygen.
Phosphoethanolamine N-methyltransferase

Adenosinetriphosphatase; Nucleoside-triphosphate phosphatase
3-hydroxyisobutyryl-CoA hydrolase

Methionine adenosyltransferase

Chitinase

Inositol-polyphosphate 5-phosphatase; 4-nitrophenylphosphatase

Adenosinetriphosphatase; Xenobiotic-transporting ATPase; Nucleoside-triphosphate phosphatase

Glucose-fructose oxidoreductase

Transferring phosphorus-containing groups
Nicotinate-nucleotide diphosphorylase (carboxylating)

Acting on paired donors, with incorporation or reduction of molecular oxygen.
Aminodeoxychorismate synthase

1-phosphatidylinositol-4-phosphate 5-kinase

Transferring phosphorus-containing groups

Thioredoxin-disulfide reductase; Protein-disulfide reductase

Transferring phosphorus-containing groups

1,3-beta-glucan synthase
DNA-directed DNA polymerase

Acting on peptide bonds (peptidases)

se II promoter;
se II promoter;

Acetate--CoA ligase

Dual-specificity kinase

N-carbamoylputrescine amidase
N-carbamoylputrescine amidase

Transferring phosphorus-containing groups

Transferring phosphorus-containing groups

Inositol 3-alpha-galactosyltransferase
Pyruvate kinase
N-acylneuraminate-9-phosphatase; 4-nitrophenylphosphatase

Aspartate transaminase

Ubiquitinyl hydrolase 1
Ubiquitinyl hydrolase 1

Nucleoside-triphosphate phosphatase

Pectinesterase; Carboxylesterase

Transferring phosphorus-containing groups

Adenosinetriphosphatase; Nucleoside-triphosphate phosphatase
DNA-directed RNA polymerase

Branched-chain-amino-acid transaminase

Acting on peptide bonds (peptidases)
Acting on ester bonds; Acting on ester bonds; Poly(A)-specific ribonuclease

Beta-ketoacyl-[acyl-carrier-protein] synthase I; Fatty-acid synthase;
Adenosinetriphosphatase; Nucleoside-triphosphate phosphatase

3-hydroxyisobutyryl-CoA hydrolase; Enoyl-CoA hydratase
Nucleoside-triphosphate phosphatase

Ubiquitinyl hydrolase 1
Asparagine--tRNA ligase

2-alkenal reductase (NAD(P)(+))

Indole-3-glycerol-phosphate synthase

RNA-directed DNA polymerase

Inositol-polyphosphate 5-phosphatase; 4-nitrophenylphosphatase
Ubiquitinyl hydrolase 1

Peptide-O-fucosyltransferase

Aminodeoxychorismate lyase
Carbon-carbon lyases; (6-4)DNA photolyase
Coniferyl-aldehyde dehydrogenase; Aldehyde dehydrogenase (NAD(P)(+))
Coniferyl-aldehyde dehydrogenase; Aldehyde dehydrogenase (NAD(P)(+))
Adenosinetriphosphatase; ATP diphosphatase; Acting on acid anhydrides;

Chalcone synthase

Malate dehydrogenase (oxaloacetate-decarboxylating);

1-phosphatidylinositol 4-kinase
1-phosphatidylinositol 4-kinase
Malate dehydrogenase; Malate dehydrogenase (NADP(+))

NADH dehydrogenase; NAD(P)H dehydrogenase (quinone)

Nucleoside-triphosphate phosphatase
Nucleoside-triphosphate phosphatase

Aralkylamine N-acetyltransferase; Arylamine N-acetyltransferase

Endo-alpha-N-acetylgalactosaminidase; Alpha-glucosidase;

Inorganic diphosphatase
Peptidylprolyl isomerase
Acyl-CoA 6-desaturase; Acting on paired donors, with incorporation or reduction of molecular oxygen.

Beta-glucuronidase

1-acylglycerophosphocholine O-acyltransferase
Cellulose synthase (UDP-forming)
Pyruvate, phosphate dikinase
Pyruvate, phosphate dikinase

Transferring phosphorus-containing groups

Aldehyde dehydrogenase (NAD(+))
Thioredoxin-disulfide reductase; Protein-disulfide reductase

Acting on peptide bonds (peptidases)

Beta-glucosidase; Glucan endo-1,3-beta-D-glucosidase

A-templated
2-carboxy-D-arabinitol-1-phosphatase; 4-nitrophenylphosphatase

Triose-phosphate isomerase
Triose-phosphate isomerase

Monodehydroascorbate reductase (NADH)

Glycerol-3-phosphate dehydrogenase

Acting on paired donors, with incorporation or reduction of molecular oxygen.

Nucleoside-triphosphate phosphatase

Transferring phosphorus-containing groups

Beta-amylase
Beta-amylase
Beta-amylase

Alcohol-forming fatty acyl-CoA reductase
Glutathione transferase
Phospholipase A(1); Carboxylesterase
Phospholipase A(1); Carboxylesterase

Thioredoxin-disulfide reductase

Nicotianamine synthase
Inositol-phosphate phosphatase; 4-nitrophenylphosphatase

Glycogen phosphorylase
Glycogen phosphorylase
ng pathway
Adenosinetriphosphatase; Calcium-transporting ATPase; Nucleoside-triphosphate phosphatase

2-keto-3-deoxy-L-rhamnonate aldolase

Acting on ester bonds; Acting on ester bonds; Poly(A)-specific ribonuclease

Acting on ester bonds; Acting on ester bonds

Adenosinetriphosphatase; Phospholipid-translocating ATPase;

Acting on paired donors, with incorporation or reduction of molecular oxygen.

Adenosinetriphosphatase; Nucleoside-triphosphate phosphatase

Shikimate dehydrogenase; 3-dehydroquinate dehydratase; Quinate/shikimate dehydrogenase

4-nitrophenylphosphatase; Pyridoxal phosphatase

Histidine kinase

Adenosinetriphosphatase; Sulfate-transporting ATPase; Nucleoside-triphosphate phosphatase

Adenosinetriphosphatase; Sulfate-transporting ATPase; Nucleoside-triphosphate phosphatase
Oxalate--CoA ligase
Acting on peptide bonds (peptidases)

Adenosinetriphosphatase; Nucleoside-triphosphate phosphatase; Cu(2+)-exporting ATPase

Hydroxypyruvate reductase

Transferring phosphorus-containing groups

Acting on diphenols and related substances as donors; L-ascorbate oxidase
Acting on diphenols and related substances as donors; L-ascorbate oxidase
Adenosinetriphosphatase; Nucleoside-triphosphate phosphatase

Adenosinetriphosphatase; Nucleoside-triphosphate phosphatase

Nucleoside-triphosphate phosphatase

Cadmium-transporting ATPase
Adenosinetriphosphatase; Nucleoside-triphosphate phosphatase
Adenosinetriphosphatase; Nucleoside-triphosphate phosphatase

Protein-serine/threonine phosphatase; 4-nitrophenylphosphatase

Protochlorophyllide reductase
NAD(+) kinase; Diacylglycerol kinase (ATP)
Thioredoxin-disulfide reductase; Protein-disulfide reductase
Adenosinetriphosphatase; Nucleoside-triphosphate phosphatase
Transferring phosphorus-containing groups

Adenosinetriphosphatase; Nucleoside-triphosphate phosphatase
Adenosinetriphosphatase; Nucleoside-triphosphate phosphatase

CDP-diacylglycerol--serine O-phosphatidyltransferase
CDP-diacylglycerol--serine O-phosphatidyltransferase
Carbonic anhydrase

UDP-glucose 4-epimerase

Shikimate dehydrogenase; 3-dehydroquinate dehydratase; Quinate/shikimate dehydrogenase

Methylthioribulose 1-phosphate dehydratase; Acireductone synthase;
Methylthioribulose 1-phosphate dehydratase; Acireductone synthase;

Histone deacetylase
Histone deacetylase
Long-chain-fatty-acid--CoA ligase
Long-chain-fatty-acid--CoA ligase

Transferring phosphorus-containing groups; Mitogen-activated protein kinase

Aldose-6-phosphate reductase (NADPH)
Aldose-6-phosphate reductase (NADPH)
Aldose-6-phosphate reductase (NADPH)

Phosphopantothenoylcysteine decarboxylase

Fructose-bisphosphate aldolase

Transferring phosphorus-containing groups

Serine--tRNA ligase

Spermidine synthase; Thermospermine synthase

Glycine hydroxymethyltransferase

NADPH--hemoprotein reductase

Phosphoinositide phospholipase C; Phospholipase C

InterPro GO Names

ate dehydrogenase (oxaloacetate-decarboxylating) (NADP(+))

.
. T
