# Supplementary material for: Differential expression of genes in olive leaves and buds of ON- versus OFF-crop trees
Source: Sci Rep. 2020 Sep 25;10:15762. doi: 10.1038/s41598-020-72895-7 (PMC7519672; doi:10.1038/s41598-020-72895-7)
Supplement: Supplementary file 6 — Supplementary Table 6. [file 41598_2020_72895_MOESM6_ESM.pdf]

Alternate bearing in olive: Differential expression of genes in leaves and buds of O

Ebrahim Dastkar[1], Ali Soleimani[1*], Hossein Jafary[2], Juan de Dios Alche[3], Abbas Bahari[4], Mehrshad Zein

Supplementary table S6. Results of KEGG pathway enrichment analysis of olive's

##Databases: KEGG PATHWAY

##Statistical test method: hypergeometric test / Fisher's exact test

##FDR correction method: Benjamini and Hochberg

#Term	Databas	ID	Input nu	Backgrc	P-Value	Corrected	Input
Metabolic pathways	KEGG	cic0110	223	1810	9.48E-17	1.00E-14	TRINITY_DN80186_c0_g2
Carbon metabolism	KEGG	cic0120	49	233	9.75E-11	5.16E-09	TRINITY_DN80186_c0_g2
Carbon fixation in photosynth	KEGG	cic0071	24	60	2.19E-10	7.74E-09	TRINITY_DN80186_c0_g2
Protein processing in endopla	KEGG	cic0414	43	208	2.11E-09	5.59E-08	TRINITY_DN77648_c2_g2
Photosynthesis - antenna prot	KEGG	cic0019	13	17	1.05E-08	2.22E-07	TRINITY_DN85155_c0_g1
Photosynthesis	KEGG	cic0019	19	47	1.43E-08	2.52E-07	TRINITY_DN87613_c5_g1
Biosynthesis of secondary me	KEGG	cic0111	118	1069	5.89E-07	8.92E-06	TRINITY_DN80186_c0_g2
Fructose and mannose metabo	KEGG	cic0005	18	59	1.05E-06	1.39E-05	TRINITY_DN80186_c0_g2
Glyoxylate and dicarboxylate	KEGG	cic0063	17	60	4.81E-06	5.67E-05	TRINITY_DN87835_c4_g2
Fatty acid degradation	KEGG	cic0007	13	38	1.19E-05	0.00013	TRINITY_DN86918_c1_g2
Pyruvate metabolism	KEGG	cic0062	16	70	8.46E-05	0.00082	TRINITY_DN84176_c2_g1
Galactose metabolism	KEGG	cic0005	14	56	0.0001	0.00091	TRINITY_DN82035_c0_g2
Glycolysis / Gluconeogenesis	KEGG	cic0001	20	106	0.00012	0.00099	TRINITY_DN80186_c0_g2
Glycerolipid metabolism	KEGG	cic0056	14	58	0.00014	0.00107	TRINITY_DN80757_c2_g2
Fatty acid metabolism	KEGG	cic0121	14	68	0.00058	0.00407	TRINITY_DN86918_c1_g2
Peroxisome	KEGG	cic0414	17	98	0.00088	0.00544	TRINITY_DN81578_c0_g1
Valine, leucine and isoleucine	KEGG	cic0028	11	47	0.00089	0.00544	TRINITY_DN86918_c1_g2
Spliceosome	KEGG	cic0304	25	176	0.00092	0.00544	TRINITY_DN88409_c1_g1
Alanine, aspartate and glutam	KEGG	cic0025	10	43	0.00157	0.00832	TRINITY_DN86215_c1_g1
Circadian rhythm - plant	KEGG	cic0471	10	43	0.00157	0.00832	TRINITY_DN87790_c2_g5
Pentose phosphate pathway	KEGG	cic0003	10	47	0.00276	0.01394	TRINITY_DN80186_c0_g2
Propanoate metabolism	KEGG	cic0064	8	32	0.0031	0.01394	TRINITY_DN86918_c1_g2
Biosynthesis of amino acids	KEGG	cic0123	27	216	0.00312	0.01394	TRINITY_DN80186_c0_g2
Flavonoid biosynthesis	KEGG	cic0094	10	48	0.00316	0.01394	TRINITY_DN79503_c1_g2
N-Glycan biosynthesis	KEGG	cic0051	9	43	0.00486	0.02062	TRINITY_DN84797_c0_g2

beta-Alanine metabolism	KEGG	cic0041	9	44	0.00554	0.02257	TRINITY_DN86918_c1_g2
Inositol phosphate metabolisr	KEGG	cic0056	10	56	0.00809	0.03178	TRINITY_DN78762_c2_g1
Nitrogen metabolism	KEGG	cic0091	7	31	0.00884	0.0328	TRINITY_DN88143_c3_g1
Phosphatidylinositol signaling	KEGG	cic0407	9	48	0.00897	0.0328	TRINITY_DN78762_c2_g1
Nicotinate and nicotinamide r	KEGG	cic0076	5	17	0.01062	0.0373	TRINITY_DN81752_c0_g1
Cysteine and methionine meta	KEGG	cic0027	14	98	0.01091	0.0373	TRINITY_DN82071_c6_g2
Stilbenoid, diarylheptanoid ar	KEGG	cic0094	6	25	0.01179	0.03906	TRINITY_DN80669_c1_g1
Aminoacyl-tRNA biosynthesi	KEGG	cic0097	9	52	0.01383	0.04441	TRINITY_DN74883_c0_g1
Ascorbate and aldarate metab	KEGG	cic0005	7	36	0.01719	0.05222	TRINITY_DN77816_c0_g5
Glycine, serine and threonine	KEGG	cic0026	10	64	0.01754	0.05222	TRINITY_DN87835_c4_g2
Glycerophospholipid metabol	KEGG	cic0056	11	74	0.01774	0.05222	TRINITY_DN84238_c1_g5
Nucleotide excision repair	KEGG	cic0342	9	61	0.03142	0.09001	TRINITY_DN82874_c0_g1
Histidine metabolism	KEGG	cic0034	4	16	0.03399	0.09482	TRINITY_DN84255_c3_g1
Selenocompound metabolism	KEGG	cic0045	4	17	0.03996	0.10618	TRINITY_DN83113_c3_g1
Fatty acid biosynthesis	KEGG	cic0006	7	44	0.04007	0.10618	TRINITY_DN88159_c0_g2
Starch and sucrose metabolisı	KEGG	cic0050	22	213	0.04202	0.10864	TRINITY_DN78767_c0_g1
Lysine degradation	KEGG	cic0031	5	27	0.04839	0.12212	TRINITY_DN81742_c2_g1
Plant hormone signal transduc	KEGG	cic0407	25	255	0.05115	0.12609	TRINITY_DN78372_c0_g2
RNA transport	KEGG	cic0301	17	160	0.05566	0.1326	TRINITY_DN87253_c3_g2
Amino sugar and nucleotide s	KEGG	cic0052	14	125	0.05629	0.1326	TRINITY_DN88193_c1_g1
Phenylalanine, tyrosine and tı	KEGG	cic0040	7	50	0.06637	0.15295	TRINITY_DN82071_c6_g2
Arginine and proline metabol	KEGG	cic0033	7	52	0.07701	0.17006	TRINITY_DN82071_c6_g2
Cyanoamino acid metabolism	KEGG	cic0046	7	52	0.07701	0.17006	TRINITY_DN87835_c4_g2
Synthesis and degradation of	KEGG	cic0007	2	6	0.08646	0.18654	TRINITY_DN81742_c2_g1
Glycosaminoglycan degradati	KEGG	cic0053	3	14	0.08799	0.18654	TRINITY_DN84298_c0_g1
Tryptophan metabolism	KEGG	cic0038	5	34	0.09691	0.20143	TRINITY_DN81742_c2_g1
Endocytosis	KEGG	cic0414	14	140	0.10811	0.22039	TRINITY_DN83275_c4_g2
Phenylpropanoid biosynthesis	KEGG	cic0094	17	179	0.11452	0.22903	TRINITY_DN80669_c1_g1
Protein export	KEGG	cic0306	6	48	0.12356	0.24253	TRINITY_DN84939_c2_g3
Butanoate metabolism	KEGG	cic0065	3	17	0.12858	0.24782	TRINITY_DN81742_c2_g1
Glycosylphosphatidylinositol	KEGG	cic0056	4	29	0.15219	0.28808	TRINITY_DN81491_c1_g1
Phenylalanine metabolism	KEGG	cic0036	6	52	0.15779	0.28969	TRINITY_DN82071_c6_g2
Pentose and glucuronate inter	KEGG	cic0004	9	88	0.15851	0.28969	TRINITY_DN84255_c3_g1

RNA degradation	KEGG	cic0301	10	101	0.16288	0.29263	TRINITY_DN81043_c1_g1
Pyrimidine metabolism	KEGG	cic0024	10	102	0.16944	0.29934	TRINITY_DN78821_c3_g5
Basal transcription factors	KEGG	cic0302	5	45	0.20635	0.35858	TRINITY_DN83281_c2_g7
Folate biosynthesis	KEGG	cic0079	3	23	0.22397	0.3799	TRINITY_DN79953_c1_g1
mRNA surveillance pathway	KEGG	cic0301	11	123	0.22579	0.3799	TRINITY_DN76989_c1_g1
Brassinosteroid biosynthesis	KEGG	cic0090	2	13	0.24382	0.40383	TRINITY_DN80847_c1_g1
Ubiquitin mediated proteolysi	KEGG	cic0412	11	129	0.26976	0.4374	TRINITY_DN78604_c0_g1
Mismatch repair	KEGG	cic0343	4	38	0.27235	0.4374	TRINITY_DN86957_c0_g1
Oxidative phosphorylation	KEGG	cic0019	9	104	0.28405	0.44689	TRINITY_DN85077_c5_g2
Tropane, piperidine and pyrid	KEGG	cic0096	4	39	0.28668	0.44689	TRINITY_DN82071_c6_g2
Arginine biosynthesis	KEGG	cic0022	3	28	0.31101	0.47779	TRINITY_DN82071_c6_g2
Carotenoid biosynthesis	KEGG	cic0090	4	41	0.31564	0.47797	TRINITY_DN81842_c2_g1
Phagosome	KEGG	cic0414	7	82	0.33019	0.49195	TRINITY_DN85077_c5_g2
One carbon pool by folate	KEGG	cic0067	2	17	0.33933	0.49195	TRINITY_DN87835_c4_g2
Homologous recombination	KEGG	cic0344	4	43	0.34483	0.49195	TRINITY_DN82498_c1_g1
Regulation of autophagy	KEGG	cic0414	3	30	0.34632	0.49195	TRINITY_DN77865_c2_g8
Purine metabolism	KEGG	cic0023	11	139	0.34808	0.49195	TRINITY_DN78821_c3_g5
Sphingolipid metabolism	KEGG	cic0060	3	31	0.36392	0.50757	TRINITY_DN80228_c2_g2
Cutin, suberine and wax biosy	KEGG	cic0007	3	33	0.39885	0.54907	TRINITY_DN81578_c0_g1
ABC transporters	KEGG	cic0201	2	22	0.4527	0.61521	TRINITY_DN79223_c1_g2
Ether lipid metabolism	KEGG	cic0056	2	23	0.47405	0.63467	TRINITY_DN77488_c1_g5
Taurine and hypotaurine meta	KEGG	cic0043	1	9	0.479	0.63467	TRINITY_DN77748_c5_g2
Non-homologous end-joining	KEGG	cic0345	1	10	0.51189	0.66988	TRINITY_DN86719_c0_g4
Terpenoid backbone biosynth	KEGG	cic0090	4	56	0.5292	0.67843	TRINITY_DN84958_c3_g3
Base excision repair	KEGG	cic0341	3	41	0.53123	0.67843	TRINITY_DN86957_c0_g1
Porphyrin and chlorophyll me	KEGG	cic0086	3	42	0.54662	0.68979	TRINITY_DN87528_c0_g2
2-Oxocarboxylic acid metabo	KEGG	cic0121	4	58	0.55546	0.6917	TRINITY_DN82071_c6_g2
Glycosphingolipid biosynthes	KEGG	cic0060	1	12	0.57158	0.6917	TRINITY_DN86622_c1_g1
Biosynthesis of unsaturated fa	KEGG	cic0104	2	28	0.57278	0.6917	TRINITY_DN79102_c2_g1
Ribosome biogenesis in eukar	KEGG	cic0300	5	75	0.57424	0.6917	TRINITY_DN86040_c0_g1
Pantothenate and CoA biosyn	KEGG	cic0077	2	30	0.60831	0.71892	TRINITY_DN88400_c1_g1
DNA replication	KEGG	cic0303	3	47	0.61895	0.71892	TRINITY_DN86957_c0_g1
Proteasome	KEGG	cic0305	3	47	0.61895	0.71892	TRINITY_DN83980_c1_g1

AGE-RAGE signaling pathwa	KEGG	cic0493	1	14	0.62397	0.71892	TRINITY_DN81210_c1_g1
RNA polymerase	KEGG	cic0302	2	33	0.6573	0.74919	TRINITY_DN78821_c3_g5
Isoquinoline alkaloid biosynth	KEGG	cic0095	2	34	0.67251	0.75038	TRINITY_DN82071_c6_g2
Sulfur metabolism	KEGG	cic0092	2	34	0.67251	0.75038	TRINITY_DN79487_c1_g1
Tyrosine metabolism	KEGG	cic0035	3	53	0.69481	0.76718	TRINITY_DN82071_c6_g2
alpha-Linolenic acid metaboli	KEGG	cic0059	2	39	0.74043	0.80664	TRINITY_DN87232_c0_g1
Valine, leucine and isoleucine	KEGG	cic0029	1	20	0.74576	0.80664	TRINITY_DN81022_c0_g1
Plant-pathogen interaction	KEGG	cic0462	11	197	0.76855	0.82289	TRINITY_DN84723_c2_g2
Other glycan degradation	KEGG	cic0051	1	23	0.79096	0.83841	TRINITY_DN77393_c0_g3
Citrate cycle (TCA cycle)	KEGG	cic0002	2	45	0.80568	0.84556	TRINITY_DN87112_c1_g1
Diterpenoid biosynthesis	KEGG	cic0090	1	28	0.84915	0.88245	TRINITY_DN83177_c0_g1
Fatty acid elongation	KEGG	cic0006	1	32	0.88381	0.90955	TRINITY_DN79102_c2_g1
Ubiquinone and other terpeno	KEGG	cic0013	2	61	0.91394	0.93152	TRINITY_DN77564_c0_g1
Glutathione metabolism	KEGG	cic0048	4	108	0.92861	0.93745	TRINITY_DN79168_c1_g1
Ribosome	KEGG	cic0301	10	281	0.98969	0.98969	TRINITY_DN77819_c1_g5

#Term	Databas	ID	Input nu	Backgro	P-Value	Corrected	Input

#Term	Databas	ID	Input nu	Backgro	P-Value	Corrected	Input

N- versus OFF-crop trees

alabedini[illegible] and Seyed Alireza Salami [illegible]

bud samples ON- *vs.* OFF-trees

Hyperlink

http://www.genome.jp/kegg-bin/show_pathway?cic01100/cic:CICLE_v10028099mg%09red/cic:CICLE_v1

http://www.genome.jp/kegg-bin/show_pathway?cic01200/cic:CICLE_v10028730mg%09red/cic:CICLE_v1

http://www.genome.jp/kegg-bin/show_pathway?cic00710/cic:CICLE_v10031559mg%09red/cic:CICLE_v1

http://www.genome.jp/kegg-bin/show_pathway?cic04141/cic:CICLE_v10025230mg%09red/cic:CICLE_v1

http://www.genome.jp/kegg-bin/show_pathway?cic00196/cic:CICLE_v10016280mg%09red/cic:CICLE_v1

http://www.genome.jp/kegg-bin/show_pathway?cic00195/cic:CICLE_v10022793mg%09red/cic:CICLE_v1

http://www.genome.jp/kegg-bin/show_pathway?cic01110/cic:CICLE_v10000949mg%09red/cic:CICLE_v1

http://www.genome.jp/kegg-bin/show_pathway?cic00051/cic:CICLE_v10008154mg%09red/cic:CICLE_v1

http://www.genome.jp/kegg-bin/show_pathway?cic00630/cic:CICLE_v10015490mg%09red/cic:CICLE_v1

http://www.genome.jp/kegg-bin/show_pathway?cic00071/cic:CICLE_v10007590mg%09red/cic:CICLE_v1

http://www.genome.jp/kegg-bin/show_pathway?cic00620/cic:CICLE_v10031559mg%09red/cic:CICLE_v1

http://www.genome.jp/kegg-bin/show_pathway?cic00052/cic:CICLE_v10014333mg%09red/cic:CICLE_v1

http://www.genome.jp/kegg-bin/show_pathway?cic00010/cic:CICLE_v10008091mg%09red/cic:CICLE_v1

http://www.genome.jp/kegg-bin/show_pathway?cic00561/cic:CICLE_v100194052m%09red/cic:CICLE_v1

http://www.genome.jp/kegg-bin/show_pathway?cic01212/cic:CICLE_v10020462mg%09red/cic:CICLE_v1

http://www.genome.jp/kegg-bin/show_pathway?cic04146/cic:CICLE_v100206971m%09red/cic:CICLE_v1

http://www.genome.jp/kegg-bin/show_pathway?cic00280/cic:CICLE_v10001030mg%09red/cic:CICLE_v1

http://www.genome.jp/kegg-bin/show_pathway?cic03040/cic:CICLE_v10029160mg%09red/cic:CICLE_v1

http://www.genome.jp/kegg-bin/show_pathway?cic00250/cic:CICLE_v10015490mg%09red/cic:CICLE_v1

http://www.genome.jp/kegg-bin/show_pathway?cic04712/cic:CICLE_v10018964mg%09red/cic:CICLE_v1

http://www.genome.jp/kegg-bin/show_pathway?cic00030/cic:CICLE_v10008091mg%09red/cic:CICLE_v1

http://www.genome.jp/kegg-bin/show_pathway?cic00640/cic:CICLE_v10001030mg%09red/cic:CICLE_v1

http://www.genome.jp/kegg-bin/show_pathway?cic01230/cic:CICLE_v100041202m%09red/cic:CICLE_v1

http://www.genome.jp/kegg-bin/show_pathway?cic00941/cic:CICLE_v10008265mg%09red/cic:CICLE_v1

http://www.genome.jp/kegg-bin/show_pathway?cic00510/cic:CICLE_v10028099mg%09red/cic:CICLE_v1

http://www.genome.jp/kegg-bin/show_pathway?cic00410/cic:CICLE_v10004291mg%09red/cic:CICLE_v1

http://www.genome.jp/kegg-bin/show_pathway?cic00562/cic:CICLE_v10014860mg%09red/cic:CICLE_v1

http://www.genome.jp/kegg-bin/show_pathway?cic00910/cic:CICLE_v100041202m%09red/cic:CICLE_v1

http://www.genome.jp/kegg-bin/show_pathway?cic04070/cic:CICLE_v10017102mg%09red/cic:CICLE_v1

http://www.genome.jp/kegg-bin/show_pathway?cic00760/cic:CICLE_v10015763mg%09red/cic:CICLE_v1

http://www.genome.jp/kegg-bin/show_pathway?cic00270/cic:CICLE_v10005146mg%09red/cic:CICLE_v1

http://www.genome.jp/kegg-bin/show_pathway?cic00945/cic:CICLE_v10010833mg%09red/cic:CICLE_v1

http://www.genome.jp/kegg-bin/show_pathway?cic00970/cic:CICLE_v10019519mg%09red/cic:CICLE_v1

http://www.genome.jp/kegg-bin/show_pathway?cic00053/cic:CICLE_v10001685mg%09red/cic:CICLE_v1

http://www.genome.jp/kegg-bin/show_pathway?cic00260/cic:CICLE_v10015490mg%09red/cic:CICLE_v1

http://www.genome.jp/kegg-bin/show_pathway?cic00564/cic:CICLE_v10020062mg%09red/cic:CICLE_v1

http://www.genome.jp/kegg-bin/show_pathway?cic03420/cic:CICLE_v10010910mg%09red/cic:CICLE_v1

http://www.genome.jp/kegg-bin/show_pathway?cic00340/cic:CICLE_v10000949mg%09red/cic:CICLE_v1

http://www.genome.jp/kegg-bin/show_pathway?cic00450/cic:CICLE_v10011434mg%09red/cic:CICLE_v1

http://www.genome.jp/kegg-bin/show_pathway?cic00061/cic:CICLE_v10020462mg%09red/cic:CICLE_v1

http://www.genome.jp/kegg-bin/show_pathway?cic00500/cic:CICLE_v10011444mg%09red/cic:CICLE_v1

http://www.genome.jp/kegg-bin/show_pathway?cic00310/cic:CICLE_v10000949mg%09red/cic:CICLE_v1

http://www.genome.jp/kegg-bin/show_pathway?cic04075/cic:CICLE_v10021954mg%09red/cic:CICLE_v1

http://www.genome.jp/kegg-bin/show_pathway?cic03013/cic:CICLE_v10029160mg%09red/cic:CICLE_v1

http://www.genome.jp/kegg-bin/show_pathway?cic00520/cic:CICLE_v10011444mg%09red/cic:CICLE_v1

http://www.genome.jp/kegg-bin/show_pathway?cic00400/cic:CICLE_v10004711mg%09red/cic:CICLE_v1

http://www.genome.jp/kegg-bin/show_pathway?cic00330/cic:CICLE_v10000949mg%09red/cic:CICLE_v1

http://www.genome.jp/kegg-bin/show_pathway?cic00460/cic:CICLE_v10031212mg%09red/cic:CICLE_v1

http://www.genome.jp/kegg-bin/show_pathway?cic00072/cic:CICLE_v10015403mg%09red

http://www.genome.jp/kegg-bin/show_pathway?cic00531/cic:CICLE_v10030724mg%09red/cic:CICLE_v1

http://www.genome.jp/kegg-bin/show_pathway?cic00380/cic:CICLE_v10000949mg%09red/cic:CICLE_v1

http://www.genome.jp/kegg-bin/show_pathway?cic04144/cic:CICLE_v10016702mg%09red/cic:CICLE_v1

http://www.genome.jp/kegg-bin/show_pathway?cic00940/cic:CICLE_v10020880mg%09red/cic:CICLE_v1

http://www.genome.jp/kegg-bin/show_pathway?cic03060/cic:CICLE_v10019973mg%09red/cic:CICLE_v1

http://www.genome.jp/kegg-bin/show_pathway?cic00650/cic:CICLE_v10019831mg%09red/cic:CICLE_v1

http://www.genome.jp/kegg-bin/show_pathway?cic00563/cic:CICLE_v10002226mg%09red/cic:CICLE_v1

http://www.genome.jp/kegg-bin/show_pathway?cic00360/cic:CICLE_v10019532mg%09red/cic:CICLE_v1

http://www.genome.jp/kegg-bin/show_pathway?cic00040/cic:CICLE_v10028892mg%09red/cic:CICLE_v1

http://www.genome.jp/kegg-bin/show_pathway?cic03018/cic:CICLE_v10025257mg%09red/cic:CICLE_v1

http://www.genome.jp/kegg-bin/show_pathway?cic00240/cic:CICLE_v10015763mg%09red/cic:CICLE_v1

http://www.genome.jp/kegg-bin/show_pathway?cic03022/cic:CICLE_v10018514mg%09red/cic:CICLE_v1

http://www.genome.jp/kegg-bin/show_pathway?cic00790/cic:CICLE_v10008846mg%09red/cic:CICLE_v1

http://www.genome.jp/kegg-bin/show_pathway?cic03015/cic:CICLE_v10029160mg%09red/cic:CICLE_v1

http://www.genome.jp/kegg-bin/show_pathway?cic00905/cic:CICLE_v10031576mg%09red/cic:CICLE_v1

http://www.genome.jp/kegg-bin/show_pathway?cic04120/cic:CICLE_v10030804mg%09red/cic:CICLE_v1

http://www.genome.jp/kegg-bin/show_pathway?cic03430/cic:CICLE_v10010910mg%09red/cic:CICLE_v1

http://www.genome.jp/kegg-bin/show_pathway?cic00190/cic:CICLE_v10028070mg%09red/cic:CICLE_v1

http://www.genome.jp/kegg-bin/show_pathway?cic00960/cic:CICLE_v10001274mg%09red/cic:CICLE_v1

http://www.genome.jp/kegg-bin/show_pathway?cic00220/cic:CICLE_v10025334mg%09red/cic:CICLE_v1

http://www.genome.jp/kegg-bin/show_pathway?cic00906/cic:CICLE_v10025089mg%09red

http://www.genome.jp/kegg-bin/show_pathway?cic04145/cic:CICLE_v10028070mg%09red/cic:CICLE_v1

http://www.genome.jp/kegg-bin/show_pathway?cic00670/cic:CICLE_v10000656mg%09red/cic:CICLE_v1

http://www.genome.jp/kegg-bin/show_pathway?cic03440/cic:CICLE_v10020181mg%09red/cic:CICLE_v1

http://www.genome.jp/kegg-bin/show_pathway?cic04140/cic:CICLE_v10015231mg%09red/cic:CICLE_v1

http://www.genome.jp/kegg-bin/show_pathway?cic00230/cic:CICLE_v10015763mg%09red/cic:CICLE_v1

http://www.genome.jp/kegg-bin/show_pathway?cic00600/cic:CICLE_v10034010mg%09red/cic:CICLE_v1

http://www.genome.jp/kegg-bin/show_pathway?cic00073/cic:CICLE_v100206971m%09red/cic:CICLE_v1

http://www.genome.jp/kegg-bin/show_pathway?cic02010/cic:CICLE_v10010936mg%09red/cic:CICLE_v1

http://www.genome.jp/kegg-bin/show_pathway?cic00565/cic:CICLE_v10014860mg%09red/cic:CICLE_v1

http://www.genome.jp/kegg-bin/show_pathway?cic00430/cic:CICLE_v10019831mg%09red

http://www.genome.jp/kegg-bin/show_pathway?cic03450/cic:CICLE_v10007283mg%09red

http://www.genome.jp/kegg-bin/show_pathway?cic00900/cic:CICLE_v10000761mg%09red/cic:CICLE_v1

http://www.genome.jp/kegg-bin/show_pathway?cic03410/cic:CICLE_v10027871mg%09red/cic:CICLE_v1

http://www.genome.jp/kegg-bin/show_pathway?cic00860/cic:CICLE_v10004154mg%09red/cic:CICLE_v1

http://www.genome.jp/kegg-bin/show_pathway?cic01210/cic:CICLE_v10025334mg%09red/cic:CICLE_v1

http://www.genome.jp/kegg-bin/show_pathway?cic00603/cic:CICLE_v10034010mg%09red

http://www.genome.jp/kegg-bin/show_pathway?cic01040/cic:CICLE_v10011949mg%09red/cic:CICLE_v1

http://www.genome.jp/kegg-bin/show_pathway?cic03008/cic:CICLE_v10019400mg%09red/cic:CICLE_v1

http://www.genome.jp/kegg-bin/show_pathway?cic00770/cic:CICLE_v10018751mg%09red/cic:CICLE_v1

http://www.genome.jp/kegg-bin/show_pathway?cic03030/cic:CICLE_v10027871mg%09red/cic:CICLE_v1

http://www.genome.jp/kegg-bin/show_pathway?cic03050/cic:CICLE_v10013059mg%09red/cic:CICLE_v1

http://www.genome.jp/kegg-bin/show_pathway?cic04933/cic:CICLE_v10026539mg%09red

http://www.genome.jp/kegg-bin/show_pathway?cic03020/cic:CICLE_v10004721mg%09red/cic:CICLE_v1

http://www.genome.jp/kegg-bin/show_pathway?cic00950/cic:CICLE_v10008224mg%09red/cic:CICLE_v1

http://www.genome.jp/kegg-bin/show_pathway?cic00920/cic:CICLE_v10011434mg%09red/cic:CICLE_v1

http://www.genome.jp/kegg-bin/show_pathway?cic00350/cic:CICLE_v10008224mg%09red/cic:CICLE_v1

http://www.genome.jp/kegg-bin/show_pathway?cic00592/cic:CICLE_v10007590mg%09red/cic:CICLE_v1

http://www.genome.jp/kegg-bin/show_pathway?cic00290/cic:CICLE_v10025674mg%09red

http://www.genome.jp/kegg-bin/show_pathway?cic04626/cic:CICLE_v10018741mg%09red/cic:CICLE_v1

http://www.genome.jp/kegg-bin/show_pathway?cic00511/cic:CICLE_v10030627mg%09red

http://www.genome.jp/kegg-bin/show_pathway?cic00020/cic:CICLE_v10028730mg%09red

http://www.genome.jp/kegg-bin/show_pathway?cic00904/cic:CICLE_v10021090mg%09red

http://www.genome.jp/kegg-bin/show_pathway?cic00062/cic:CICLE_v100162851m%09red

http://www.genome.jp/kegg-bin/show_pathway?cic00130/cic:CICLE_v10000921mg%09red/cic:CICLE_v1

http://www.genome.jp/kegg-bin/show_pathway?cic00480/cic:CICLE_v10022212mg%09red/cic:CICLE_v1

http://www.genome.jp/kegg-bin/show_pathway?cic03010/cic:CICLE_v10022792mg%09red/cic:CICLE_v1

Hyperlink

Hyperlink

00149972m%09red/cic:CICLE_v10000949mg%09red/cic:CICLE_v10005043mg%09red/cic:CICLE_v1002
0014963mg%09red/cic:CICLE_v10001344mg%09red/cic:CICLE_v10008327mg%09red/cic:CICLE_v1001
0028730mg%09red/cic:CICLE_v10025334mg%09red/cic:CICLE_v10015476mg%09red/cic:CICLE_v1003
0019144mg%09red/cic:CICLE_v10029467mg%09red/cic:CICLE_v10004456mg%09red/cic:CICLE_v1003
0016287mg%09red/cic:CICLE_v10016074mg%09red/cic:CICLE_v10002178mg%09red/cic:CICLE_v1000
0024455mg%09red/cic:CICLE_v10016525mg%09red/cic:CICLE_v10002698mg%09red/cic:CICLE_v1001
0005043mg%09red/cic:CICLE_v100111191m%09red/cic:CICLE_v10028639mg%09red/cic:CICLE_v1003
00194052m%09red/cic:CICLE_v10011654mg%09red/cic:CICLE_v10028924mg%09red/cic:CICLE_v1002
0028730mg%09red/cic:CICLE_v10015403mg%09red/cic:CICLE_v10004137mg%09red/cic:CICLE_v1001
0004291mg%09red/cic:CICLE_v10015403mg%09red/cic:CICLE_v10000949mg%09red/cic:CICLE_v1001
0006837mg%09red/cic:CICLE_v10028730mg%09red/cic:CICLE_v10015403mg%09red/cic:CICLE_v1000
0021027mg%09red/cic:CICLE_v10020919mg%09red/cic:CICLE_v10004465mg%09red/cic:CICLE_v1001
0000949mg%09red/cic:CICLE_v10014963mg%09red/cic:CICLE_v10014703mg%09red/cic:CICLE_v1000
0000949mg%09red/cic:CICLE_v10015037mg%09red/cic:CICLE_v10020062mg%09red/cic:CICLE_v1000
0007590mg%09red/cic:CICLE_v100162851m%09red/cic:CICLE_v10004291mg%09red/cic:CICLE_v1001
0007566mg%09red/cic:CICLE_v10015490mg%09red/cic:CICLE_v10015533mg%09red/cic:CICLE_v1002
0004291mg%09red/cic:CICLE_v10015403mg%09red/cic:CICLE_v10000949mg%09red/cic:CICLE_v1002
0012216mg%09red/cic:CICLE_v10032170mg%09red/cic:CICLE_v10004187mg%09red/cic:CICLE_v1002
00041202m%09red/cic:CICLE_v10025334mg%09red/cic:CICLE_v10025269mg%09red/cic:CICLE_v1000
0027794mg%09red/cic:CICLE_v10015535mg%09red/cic:CICLE_v10007592mg%09red
0017877mg%09red/cic:CICLE_v10000722mg%09red/cic:CICLE_v10028594mg%09red/cic:CICLE_v1000
0004291mg%09red/cic:CICLE_v10015403mg%09red/cic:CICLE_v10008327mg%09red/cic:CICLE_v1001
0014963mg%09red/cic:CICLE_v10020016mg%09red/cic:CICLE_v10015498mg%09red/cic:CICLE_v1002
0011520mg%09red/cic:CICLE_v10010833mg%09red/cic:CICLE_v10012561mg%09red/cic:CICLE_v1001
0026261mg%09red/cic:CICLE_v100284181m%09red/cic:CICLE_v10025230mg%09red/cic:CICLE_v1000

0000949mg%09red/cic:CICLE_v10008327mg%09red/cic:CICLE_v10030749mg%09red/cic:CICLE_v1002
0017102mg%09red/cic:CICLE_v10010149mg%09red/cic:CICLE_v10027664mg%09red/cic:CICLE_v1002
0004137mg%09red/cic:CICLE_v10032517mg%09red/cic:CICLE_v10000220mg%09red/cic:CICLE_v1003
0012752mg%09red/cic:CICLE_v10027664mg%09red/cic:CICLE_v10000342mg%09red/cic:CICLE_v1001
0025780mg%09red/cic:CICLE_v10000146mg%09red/cic:CICLE_v10018157mg%09red
0007808mg%09red/cic:CICLE_v100292252m%09red/cic:CICLE_v10028730mg%09red/cic:CICLE_v1001
0008265mg%09red/cic:CICLE_v10012561mg%09red/cic:CICLE_v10000921mg%09red/cic:CICLE_v1001
0004652mg%09red/cic:CICLE_v10001143mg%09red/cic:CICLE_v10031253mg%09red/cic:CICLE_v1002
0011764mg%09red/cic:CICLE_v10000949mg%09red/cic:CICLE_v10017102mg%09red/cic:CICLE_v1000
0000656mg%09red/cic:CICLE_v10007310mg%09red/cic:CICLE_v10030749mg%09red/cic:CICLE_v1002
0026074mg%09red/cic:CICLE_v10014860mg%09red/cic:CICLE_v10031473mg%09red/cic:CICLE_v1000
0027871mg%09red/cic:CICLE_v10011886mg%09red/cic:CICLE_v10024418mg%09red/cic:CICLE_v1001
0001274mg%09red/cic:CICLE_v10025492mg%09red
0024095mg%09red/cic:CICLE_v10004686mg%09red/cic:CICLE_v10030995mg%09red
00111191m%09red/cic:CICLE_v10000466mg%09red/cic:CICLE_v10019205mg%09red/cic:CICLE_v1001
0010989mg%09red/cic:CICLE_v10004620mg%09red/cic:CICLE_v10014741mg%09red/cic:CICLE_v1000
0025492mg%09red/cic:CICLE_v10015403mg%09red
0013385mg%09red/cic:CICLE_v10031682mg%09red/cic:CICLE_v10001159mg%09red/cic:CICLE_v1002
0031552mg%09red/cic:CICLE_v10018664mg%09red/cic:CICLE_v10023883mg%09red/cic:CICLE_v1000
0014741mg%09red/cic:CICLE_v100311241m%09red/cic:CICLE_v10001524mg%09red/cic:CICLE_v1001
0001274mg%09red/cic:CICLE_v10020342mg%09red/cic:CICLE_v10008224mg%09red/cic:CICLE_v1002
0028933mg%09red/cic:CICLE_v10008224mg%09red/cic:CICLE_v10025492mg%09red/cic:CICLE_v1001
0031262mg%09red/cic:CICLE_v10000656mg%09red/cic:CICLE_v10031209mg%09red/cic:CICLE_v1002

0019304mg%09red
0025492mg%09red/cic:CICLE_v10015403mg%09red
0027981mg%09red/cic:CICLE_v10007888mg%09red/cic:CICLE_v10009534mg%09red/cic:CICLE_v1000
0031262mg%09red/cic:CICLE_v10008265mg%09red/cic:CICLE_v10008077mg%09red/cic:CICLE_v1001
0019184mg%09red/cic:CICLE_v10032463mg%09red/cic:CICLE_v10027536mg%09red
0015403mg%09red
0031452mg%09red/cic:CICLE_v10018715mg%09red/cic:CICLE_v10025261mg%09red
0012561mg%09red/cic:CICLE_v10001274mg%09red/cic:CICLE_v10008224mg%09red/cic:CICLE_v1003
0000949mg%09red/cic:CICLE_v10008154mg%09red/cic:CICLE_v10011657mg%09red/cic:CICLE_v1002

0026260mg%09red/cic:CICLE_v10011329mg%09red/cic:CICLE_v10024744mg%09red/cic:CICLE_v1002

0025780mg%09red/cic:CICLE_v10024291mg%09red/cic:CICLE_v10004721mg%09red/cic:CICLE_v1001

0014841mg%09red/cic:CICLE_v10008533mg%09red/cic:CICLE_v10021272mg%09red/cic:CICLE_v1001

0030655mg%09red/cic:CICLE_v10031375mg%09red

0031552mg%09red/cic:CICLE_v10005216mg%09red/cic:CICLE_v10011287mg%09red/cic:CICLE_v1003

0031156mg%09red

0022710mg%09red/cic:CICLE_v10022463mg%09red/cic:CICLE_v10004406mg%09red/cic:CICLE_v1000

0027871mg%09red/cic:CICLE_v10012551mg%09red/cic:CICLE_v10018525mg%09red

0002758mg%09red/cic:CICLE_v10009918mg%09red/cic:CICLE_v10024641mg%09red/cic:CICLE_v1001

0008224mg%09red/cic:CICLE_v10030749mg%09red/cic:CICLE_v10012494mg%09red

0008224mg%09red/cic:CICLE_v10028560mg%09red

0019973mg%09red/cic:CICLE_v10027830mg%09red/cic:CICLE_v10026539mg%09red/cic:CICLE_v1002

0025372mg%09red

0027772mg%09red/cic:CICLE_v10012551mg%09red/cic:CICLE_v10021754mg%09red

0008225mg%09red/cic:CICLE_v10025105mg%09red

0021337mg%09red/cic:CICLE_v10014963mg%09red/cic:CICLE_v10025780mg%09red/cic:CICLE_v1000

0032432mg%09red/cic:CICLE_v10027865mg%09red

00202442m%09red/cic:CICLE_v10019279mg%09red

0004128mg%09red

0020062mg%09red

0015403mg%09red/cic:CICLE_v10028384mg%09red

0012551mg%09red/cic:CICLE_v10010910mg%09red

0025338mg%09red/cic:CICLE_v10005043mg%09red

0008224mg%09red/cic:CICLE_v10025674mg%09red/cic:CICLE_v10028560mg%09red

00162851m%09red

0012260mg%09red/cic:CICLE_v10000344mg%09red/cic:CICLE_v10024676mg%09red/cic:CICLE_v1001

0025674mg%09red

0012551mg%09red/cic:CICLE_v10010910mg%09red

0001269mg%09red/cic:CICLE_v10010323mg%09red

0026399mg%09red

0030749mg%09red

0015399mg%09red

0030749mg%09red/cic:CICLE_v10001274mg%09red

0033676mg%09red

0007633mg%09red/cic:CICLE_v10012752mg%09red/cic:CICLE_v10029873mg%09red/cic:CICLE_v1000[illegible]

0019532mg%09red

0004819mg%09red/cic:CICLE_v10001685mg%09red

0009636mg%09red/cic:CICLE_v10016825mg%09red/cic:CICLE_v10029569mg%09red/cic:CICLE_v1000[illegible]

6034mg%09red/cic:CICLE_v100111191m%09red/cic:CICLE_v10028924mg%09red/cic:CICLE_v1002061

11191m%09red/cic:CICLE_v10006837mg%09red/cic:CICLE_v10020614mg%09red/cic:CICLE_v1002863

1765mg%09red/cic:CICLE_v10024268mg%09red/cic:CICLE_v10028924mg%09red/cic:CICLE_v1000134

3622mg%09red/cic:CICLE_v10022710mg%09red/cic:CICLE_v10019973mg%09red/cic:CICLE_v1002753

9198mg%09red/cic:CICLE_v10031886mg%09red/cic:CICLE_v10029116mg%09red/cic:CICLE_v1002145

67062m%09red/cic:CICLE_v10032629mg%09red/cic:CICLE_v10022750mg%09red/cic:CICLE_v1002603

3676mg%09red/cic:CICLE_v10015533mg%09red/cic:CICLE_v10025674mg%09red/cic:CICLE_v1001710

7872mg%09red/cic:CICLE_v10028892mg%09red/cic:CICLE_v10017877mg%09red/cic:CICLE_v1002859

5533mg%09red/cic:CICLE_v10000656mg%09red/cic:CICLE_v10007310mg%09red/cic:CICLE_v1002069

9205mg%09red/cic:CICLE_v10000466mg%09red/cic:CICLE_v10019200mg%09red/cic:CICLE_v1002549

0949mg%09red/cic:CICLE_v10014963mg%09red/cic:CICLE_v10024268mg%09red/cic:CICLE_v1001111

1657mg%09red/cic:CICLE_v10004399mg%09red/cic:CICLE_v10028892mg%09red/cic:CICLE_v1003401

8965mg%09red/cic:CICLE_v10025492mg%09red/cic:CICLE_v10028924mg%09red/cic:CICLE_v1001147

8803mg%09red/cic:CICLE_v10011473mg%09red/cic:CICLE_v10025492mg%09red/cic:CICLE_v1002889

5403mg%09red/cic:CICLE_v10011949mg%09red/cic:CICLE_v10019205mg%09red/cic:CICLE_v1001920

02442m%09red/cic:CICLE_v10019205mg%09red/cic:CICLE_v10000466mg%09red/cic:CICLE_v1002587

5674mg%09red/cic:CICLE_v10008327mg%09red/cic:CICLE_v10025492mg%09red/cic:CICLE_v1002061

7981mg%09red/cic:CICLE_v10014912mg%09red/cic:CICLE_v10025009mg%09red/cic:CICLE_v1002164

8224mg%09red/cic:CICLE_v10028078mg%09red/cic:CICLE_v10019831mg%09red/cic:CICLE_v1002770

1734mg%09red

11191m%09red/cic:CICLE_v10020614mg%09red

8594mg%09red/cic:CICLE_v10025372mg%09red/cic:CICLE_v10025674mg%09red/cic:CICLE_v1002856

5535mg%09red/cic:CICLE_v10019637mg%09red/cic:CICLE_v10003741mg%09red/cic:CICLE_v1000092

5020mg%09red/cic:CICLE_v100194961m%09red/cic:CICLE_v10019843mg%09red

5492mg%09red/cic:CICLE_v10019831mg%09red/cic:CICLE_v10020614mg%09red

8924mg%09red/cic:CICLE_v10000342mg%09red/cic:CICLE_v10015670mg%09red

1126mg%09red/cic:CICLE_v10028905mg%09red

5037mg%09red/cic:CICLE_v10010149mg%09red/cic:CICLE_v10015670mg%09red

1434mg%09red/cic:CICLE_v10025674mg%09red/cic:CICLE_v10008682mg%09red/cic:CICLE_v1000699[illegible]

1520mg%09red

4095mg%09red/cic:CICLE_v10030103mg%09red/cic:CICLE_v10030995mg%09red/cic:CICLE_v1001336[illegible]

1524mg%09red/cic:CICLE_v10025492mg%09red

1430mg%09red/cic:CICLE_v10028639mg%09red/cic:CICLE_v10025372mg%09red

8803mg%09red/cic:CICLE_v10001624mg%09red/cic:CICLE_v10015037mg%09red/cic:CICLE_v1003096[illegible]

4310mg%09red/cic:CICLE_v10012551mg%09red/cic:CICLE_v10008533mg%09red

9200mg%09red

7401mg%09red/cic:CICLE_v10027815mg%09red/cic:CICLE_v10007483mg%09red/cic:CICLE_v1003120[illegible]

9873mg%09red/cic:CICLE_v10028703mg%09red/cic:CICLE_v10031020mg%09red/cic:CICLE_v1002048[illegible]

2741mg%09red/cic:CICLE_v10011515mg%09red/cic:CICLE_v10018460mg%09red/cic:CICLE_v1002579[illegible]

0227mg%09red/cic:CICLE_v10011265mg%09red/cic:CICLE_v10020919mg%09red/cic:CICLE_v1001122[illegible]

0016mg%09red/cic:CICLE_v10015498mg%09red

3866mg%09red

5372mg%09red

9355mg%09red/cic:CICLE_v10000342mg%09red/cic:CICLE_v10011260mg%09red/cic:CICLE_v1002133[illegible]

9532mg%09red/cic:CICLE_v10011520mg%09red/cic:CICLE_v10010833mg%09red/cic:CICLE_v1001256[illegible]

0749mg%09red/cic:CICLE_v10000921mg%09red

5492mg%09red

7687mg%09red/cic:CICLE_v10011287mg%09red/cic:CICLE_v10014640mg%09red/cic:CICLE_v1002796

2551mg%09red/cic:CICLE_v10026399mg%09red/cic:CICLE_v10004686mg%09red/cic:CICLE_v1001583

2002mg%09red

0915mg%09red/cic:CICLE_v10025009mg%09red/cic:CICLE_v10000901mg%09red/cic:CICLE_v100007

2741mg%09red/cic:CICLE_v10022662mg%09red/cic:CICLE_v10032916mg%09red/cic:CICLE_v1001431

3498mg%09red/cic:CICLE_v10007367mg%09red/cic:CICLE_v10027830mg%09red/cic:CICLE_v1001318

7536mg%09red/cic:CICLE_v10010149mg%09red

4721mg%09red/cic:CICLE_v10012551mg%09red/cic:CICLE_v10026399mg%09red/cic:CICLE_v1001623

1515mg%09red

7381mg%09red/cic:CICLE_v10028110mg%09red/cic:CICLE_v10004456mg%09red/cic:CICLE_v1002045

0894mg%09red/cic:CICLE_v10005900mg%09red/cic:CICLE_v10003001mg%09red/cic:CICLE_v1003187

4mg%09red/cic:CICLE_v10028639mg%09red/cic:CICLE_v10022793mg%09red/cic:CICLE_v100284181m
9mg%09red/cic:CICLE_v10025372mg%09red/cic:CICLE_v10004291mg%09red/cic:CICLE_v10015533mg
4mg%09red/cic:CICLE_v10006837mg%09red/cic:CICLE_v10030580mg%09red/cic:CICLE_v10001734mg
6mg%09red/cic:CICLE_v10011610mg%09red/cic:CICLE_v10027981mg%09red/cic:CICLE_v10028531mg
2mg%09red
4mg%09red/cic:CICLE_v10002099mg%09red/cic:CICLE_v10027108mg%09red/cic:CICLE_v10032789mg
2mg%09red/cic:CICLE_v10028560mg%09red/cic:CICLE_v10015037mg%09red/cic:CICLE_v10001030mg
4mg%09red/cic:CICLE_v10001734mg%09red
2mg%09red/cic:CICLE_v10028639mg%09red/cic:CICLE_v10025372mg%09red
2mg%09red
91m%09red/cic:CICLE_v10025492mg%09red/cic:CICLE_v10011472mg%09red/cic:CICLE_v10030580mg
0mg%09red/cic:CICLE_v10001308mg%09red
2mg%09red/cic:CICLE_v10017877mg%09red/cic:CICLE_v10000722mg%09red/cic:CICLE_v10028594mg
2mg%09red/cic:CICLE_v10034010mg%09red/cic:CICLE_v10011320mg%09red
0mg%09red/cic:CICLE_v100111191m%09red/cic:CICLE_v10000466mg%09red
7mg%09red/cic:CICLE_v10019200mg%09red/cic:CICLE_v10008646mg%09red/cic:CICLE_v10005524mg
4mg%09red
6mg%09red/cic:CICLE_v10014300mg%09red/cic:CICLE_v10002266mg%09red/cic:CICLE_v10011260mg
3mg%09red

0mg%09red/cic:CICLE_v10008224mg%09red/cic:CICLE_v10011472mg%09red/cic:CICLE_v10000656mg
1mg%09red

3mg%09red/cic:CICLE_v10008224mg%09red/cic:CICLE_v10011912mg%09red/cic:CICLE_v10028614mg

4mg%09red/cic:CICLE_v10028310mg%09red

3mg%09red/cic:CICLE_v10015341mg%09red/cic:CICLE_v10011320mg%09red

9mg%09red/cic:CICLE_v10011265mg%09red/cic:CICLE_v10031262mg%09red/cic:CICLE_v10004465mg

1mg%09red/cic:CICLE_v10004981mg%09red/cic:CICLE_v10028435mg%09red/cic:CICLE_v10012769mg

0mg%09red/cic:CICLE_v10011287mg%09red/cic:CICLE_v10025009mg%09red/cic:CICLE_v10017475mg

8mg%09red/cic:CICLE_v10004731mg%09red/cic:CICLE_v10011657mg%09red/cic:CICLE_v100149972m

9mg%09red/cic:CICLE_v10033622mg%09red

1mg%09red/cic:CICLE_v10031209mg%09red/cic:CICLE_v10000921mg%09red/cic:CICLE_v10026185mg

4mg%09red/cic:CICLE_v10018430mg%09red

8mg%09red/cic:CICLE_v10027703mg%09red

9mg%09red/cic:CICLE_v10004147mg%09red/cic:CICLE_v10014158mg%09red

0mg%09red/cic:CICLE_v10021159mg%09red/cic:CICLE_v10014194mg%09red

9mg%09red/cic:CICLE_v10007368mg%09red

7mg%09red/cic:CICLE_v10007794mg%09red/cic:CICLE_v10011472mg%09red

8mg%09red

7mg%09red

1%09red/cic:CICLE_v10016280mg%09red/cic:CICLE_v10033676mg%09red/cic:CICLE_v10015533mg%0
g%09red/cic:CICLE_v10028594mg%09red/cic:CICLE_v10011472mg%09red/cic:CICLE_v10030580mg%0
g%09red/cic:CICLE_v10017877mg%09red/cic:CICLE_v10028594mg%09red/cic:CICLE_v10007668mg%0
g%09red/cic:CICLE_v10004406mg%09red/cic:CICLE_v10003244mg%09red/cic:CICLE_v10032916mg%0

g%09red/cic:CICLE_v10002793mg%09red
g%09red/cic:CICLE_v10021337mg%09red/cic:CICLE_v10031576mg%09red/cic:CICLE_v10025780mg%0

g%09red/cic:CICLE_v10007668mg%09red

g%09red/cic:CICLE_v10001734mg%09red

g%09red/cic:CICLE_v10020692mg%09red

g%09red/cic:CICLE_v10025502mg%09red/cic:CICLE_v10019082mg%09red/cic:CICLE_v10000727mg%0

g%09red/cic:CICLE_v10011912mg%09red/cic:CICLE_v10021430mg%09red/cic:CICLE_v10004711mg%0

g%09red/cic:CICLE_v10013866mg%09red

g%09red/cic:CICLE_v10011657mg%09red/cic:CICLE_v10004221mg%09red/cic:CICLE_v10031212mg%0

g%09red/cic:CICLE_v10007708mg%09red/cic:CICLE_v10018972mg%09red/cic:CICLE_v10022032mg%0

g%09red/cic:CICLE_v10000901mg%09red/cic:CICLE_v10019079mg%09red/cic:CICLE_v10000154mg%0

n%09red/cic:CICLE_v10000722mg%09red

g%09red/cic:CICLE_v10031212mg%09red/cic:CICLE_v10010275mg%09red

9red/cic:CICLE_v10011473mg%09red/cic:CICLE_v10028933mg%09red/cic:CICLE_v10025674mg%09rec
9red/cic:CICLE_v10000656mg%09red/cic:CICLE_v10031765mg%09red/cic:CICLE_v10015476mg%09rec
9red
9red/cic:CICLE_v10011886mg%09red/cic:CICLE_v10011260mg%09red/cic:CICLE_v10027213mg%09rec
9red/cic:CICLE_v10031262mg%09red/cic:CICLE_v10001524mg%09red/cic:CICLE_v10008803mg%09rec
9red/cic:CICLE_v10033622mg%09red/cic:CICLE_v10004206mg%09red
9red/cic:CICLE_v10017877mg%09red/cic:CICLE_v10005146mg%09red/cic:CICLE_v10025334mg%09rec

9red/cic:CICLE_v10007311mg%09red/cic:CICLE_v10000722mg%09red/cic:CICLE_v10004731mg%09re[illegible]

9red/cic:CICLE_v10007286mg%09red/cic:CICLE_v10019131mg%09red/cic:CICLE_v10030589mg%09re[illegible]
9red

d/cic:CICLE_v10017102mg%09red/cic:CICLE_v10028560mg%09red/cic:CICLE_v10027664mg%09red/cic
d/cic:CICLE_v10021430mg%09red/cic:CICLE_v10017877mg%09red/cic:CICLE_v10000722mg%09red/cic

d/cic:CICLE_v10027715mg%09red/cic:CICLE_v10019948mg%09red/cic:CICLE_v10025307mg%09red/cic

d/cic:CICLE_v10020692mg%09red/cic:CICLE_v10001943mg%09red/cic:CICLE_v10025269mg%09red/cic

d/cic:CICLE_v10001274mg%09red/cic:CICLE_v10020342mg%09red/cic:CICLE_v10028924mg%09red/cic

d

d/cic:CICLE_v10014068mg%09red/cic:CICLE_v10017466mg%09red/cic:CICLE_v10007648mg%09red

:CICLE_v10005020mg%09red/cic:CICLE_v10015037mg%09red/cic:CICLE_v10015670mg%09red/cic:CI
:CICLE_v10020692mg%09red/cic:CICLE_v10008091mg%09red/cic:CICLE_v10031559mg%09red/cic:CI
:CICLE_v10016469mg%09red/cic:CICLE_v10007633mg%09red/cic:CICLE_v10029453mg%09red/cic:CI
:CICLE_v10020342mg%09red/cic:CICLE_v10028730mg%09red/cic:CICLE_v10014963mg%09red/cic:CI
:CICLE_v10028614mg%09red/cic:CICLE_v10011434mg%09red

CLE_v10001030mg%09red/cic:CICLE_v10021337mg%09red/cic:CICLE_v10018751mg%09red/cic:CICL
CLE_v10015490mg%09red/cic:CICLE_v10015403mg%09red/cic:CICLE_v10025334mg%09red/cic:CICL

CLE_v10016067mg%09red/cic:CICLE_v10015385mg%09red/cic:CICLE_v10007326mg%09red/cic:CICL

CLE_v10025492mg%09red/cic:CICLE_v10028384mg%09red/cic:CICLE_v10025372mg%09red/cic:CICL

E_v10019205mg%09red/cic:CICLE_v10025780mg%09red/cic:CICLE_v10018157mg%09red/cic:CICLE_v

E_v100194052m%09red/cic:CICLE_v10007310mg%09red/cic:CICLE_v10024268mg%09red/cic:CICLE_v

E_v10027840mg%09red/cic:CICLE_v10009756mg%09red/cic:CICLE_v10019184mg%09red

E_v10011520mg%09red/cic:CICLE_v10008224mg%09red/cic:CICLE_v10025338mg%09red/cic:CICLE_v

10015399mg%09red/cic:CICLE_v10019200mg%09red/cic:CICLE_v10031262mg%09red/cic:CICLE_v100[illegible]
10028924mg%09red/cic:CICLE_v10001734mg%09red/cic:CICLE_v10007668mg%09red

10020880mg%09red/cic:CICLE_v10000656mg%09red/cic:CICLE_v10011912mg%09red/cic:CICLE_v100[illegible]

)1524mg%09red/cic:CICLE_v10015476mg%09red/cic:CICLE_v10008803m

28078mg%09red/cic:CICLE_v10004711mg%09red/cic:CICLE_v100162851
