# Supplementary material for: Differential expression of genes in olive leaves and buds of ON- versus OFF-crop trees
Source: Sci Rep. 2020 Sep 25;10:15762. doi: 10.1038/s41598-020-72895-7 (PMC7519672; doi:10.1038/s41598-020-72895-7)
Supplement: Supplementary file 7 — Supplementary Table 7. [file 41598_2020_72895_MOESM7_ESM.pdf]

Alternate bearing in olive: Differential expression of genes in leaves and buds of ON- *versus* OFF-crop trees

Ebrahim Dastkar[1], Ali Soleimani[1*], Hossein Jafary[2], Juan de Dios Alche[3], Abbas Bahari[4], Mehrshad Zeinalabedini[5] and Seyed Alireza Salami[6]

Supplementary table S7. List of 82 differentially expressed transcripts associated with some biosynthetic pathways and flower-related genes in olive's bud samples, ON- *vs.* OFF-trees

	Seq-ID	GenBank Accession	Description	GO Names	log2 Fold Change	padj*
**Photosynthesis and carbohydrates metabolism**	TRINITY_DN82606_c0_g3_i4	XP_021990082.1	ruBisCO large subunit-binding protein subunit beta, chloroplastic	F:ATP binding; C:mitochondrion; C:cytosol; P:'de novo' protein folding; P:mitochondrion organization; C:chloroplast; P:protein refolding; F:protein binding involved in protein folding; F:unfolded protein binding; P:chaperone-mediated protein folding	1.53	1.41E-02
	TRINITY_DN85546_c2_g1_i7	XP_022872965.1	ruBisCO large subunit-binding protein subunit beta, chloroplastic	F:ATP binding; C:mitochondrion; C:cytosol; P:'de novo' protein folding; P:mitochondrion organization; C:chloroplast; P:protein refolding; F:protein binding involved in protein folding; F:unfolded protein binding; P:chaperone-mediated protein folding	1.67	1.78E-02
	TRINITY_DN82507_c0_g2_i3	XP_022883294.1	ruBisCO large subunit-binding protein subunit alpha	F:ATP binding; C:mitochondrion; C:cytosol; P:'de novo' protein folding; P:mitochondrion organization; C:chloroplast; P:chloroplast organization; P:protein refolding; F:protein binding involved in protein folding; F:unfolded protein binding; P:chaperone-mediated protein	1.69	2.44E-02
	TRINITY_DN88686_c6_g1_i6	XP_022890894.1	ribulosebisphosphate carboxylase/oxygenaseactivase, chloroplastic isoform X2	C:proteasome complex; F:ATP binding; C:chloroplast stroma; C:thylakoid; P:positive regulation of catalytic activity; F:ribulose-1,5-bisphosphate carboxylase/oxygenase activator	3.16	6.72E-08
	TRINITY_DN88686_c6_g4_i2	XP_022890894.1	ribulosebisphosphate carboxylase/oxygenaseactivase, chloroplastic	F:ATP binding	3.03	2.72E-08
	TRINITY_DN88686_c6_g2_i2	ABS72022.1	Ribulosebisphosphate carboxylase/oxygenase activase	F:phosphopantothenoylcysteine decarboxylase activity; F:ATP binding	2.96	1.83E-06
	TRINITY_DN88686_c6_g6_i2	XP_022852897.1	ribulosebisphosphate carboxylase/oxygenaseactivase 2, chloroplastic	F:ATP binding	6.20	1.10E-02
	TRINITY_DN81161_c2_g3_i3	XP_022842512.1	sucrose synthase	P:sucrose metabolic process; F:sucrose synthase activity	5.95	0.00
	TRINITY_DN81706_c3_g1_i6	XP_022893375.1	probable plastidic glucose transporter 2 isoform X1	P:carbohydrate transport; C:integral component of membrane; F:transmembrane transporter activity; P:transmembrane transport	8.48	0.03

* adjusted P-value

Supplementary table S8. Continue

	Seq-ID	GenBank Accession	Description	GO Names	log2 Fold Change	padj*
**Photosynthesis and carbohydrates metabolism**	TRINITY_DN84842_c0_g2_i5	XP_022866855.1	alpha-amylase 3, chloroplastic	F:alpha-amylase activity; F:calcium ion binding; P:carbohydrate metabolic process; F:alpha-amylase activity (releasing maltohexaose)	-6.30	0.04
	TRINITY_DN85185_c1_g1_i8	XP_022887206.1	probable sucrose-phosphate synthase 2	P:sucrose metabolic process; F:sucrose synthase activity; F:sucrose-phosphate synthase activity; F:glutamine N-phenylacetyltransferase activity	-6.92	0.04
	TRINITY_DN85119_c0_g1_i24	XP_009626949.1	UTP--glucose-1-phosphate uridylyltransferase	F:UTP:glucose-1-phosphate uridylyltransferase activity; C:cytoplasm; P:UDP-glucose metabolic process	9.24	0.00
	TRINITY_DN86759_c2_g1_i17	XP_022871791.1	putative glucose-6-phosphate 1-epimerase	P:carbohydrate metabolic process; F:carbohydrate binding; F:glucose-6-phosphate 1-epimerase activity	7.60	0.01
	TRINITY_DN86759_c2_g1_i19	XP_022871791.1	putative glucose-6-phosphate 1-epimerase	P:carbohydrate metabolic process; F:carbohydrate binding; F:glucose-6-phosphate 1-epimerase activity	-7.42	0.00
	TRINITY_DN85840_c2_g3_i5	XP_020870886.1	glucose-1-phosphate adenylyltransferase small subunit 2, chloroplastic	F:ATP binding; P:glycogen biosynthetic process; F:glucose-1-phosphate adenylyltransferase activity; C:chloroplast; P:starch biosynthetic process	-6.82	0.01
	TRINITY_DN87512_c0_g1_i13	XP_022862344.1	glucose-1-phosphate adenylyltransferase large subunit 3, chloroplastic/amyloplastic	F:ATP binding; P:glycogen biosynthetic process; F:glucose-1-phosphate adenylyltransferase activity; C:chloroplast; P:starch biosynthetic process	5.37	0.04
	TRINITY_DN87512_c0_g1_i40	XP_022862344.1	glucose-1-phosphate adenylyltransferase large subunit 3, chloroplastic/amyloplastic	F:ATP binding; P:glycogen biosynthetic process; F:glucose-1-phosphate adenylyltransferase activity; C:chloroplast; P:starch biosynthetic process	4.73	0.00
	TRINITY_DN87518_c0_g3_i3	XP_022882411.1	probable sucrose-phosphate synthase	C:cytosol; C:plasma membrane; P:sucrose metabolic process; C:plasmodesma; F:sucrose synthase activity; F:sucrose-phosphate synthase activity; P:nectar secretion	-7.77	0.00

* adjusted P-value

Supplementary table S8. Continue

	Seq-ID	GenBank Accession	Description	GO Names	log2 Fold Change	padj*
Photosynthesis and carbohydrates metabolism	TRINITY_DN83938_c0_g1_i15	XP_022879269.1	probable galactinol--sucrose galactosyltransferase 2	F:hydrolase activity; F:galactinol-raffinose galactosyltransferase activity; F:galactinol-sucrose galactosyltransferase activity	-9.27	0.00
	TRINITY_DN82733_c0_g1_i3	XP_022869917.1	bidirectional sugar transporter SWEET2	C:plasma membrane; C:integral component of membrane; P:carbohydrate transmembrane transport; F:sugar transmembrane transporter activity	6.73	0.01
	TRINITY_DN82902_c2_g2_i19	XP_011083695.1	glucose-6-phosphate isomerase, cytosolic	F:glucose-6-phosphate isomerase activity; C:cytosol; P:gluconeogenesis; P:glycolytic process; P:defense response to fungus, incompatible interaction; P:response to cadmium ion	-7.65	0.02
	TRINITY_DN86051_c2_g2_i13	XP_022895704.1	galactinol synthase 2-like	P:galactose metabolic process; F:inositol 3-alpha-galactosyltransferase activity	9.29	0.03
	TRINITY_DN86051_c2_g2_i20	XP_022875961.1	galactinol synthase 1	P:galactose metabolic process; P:response to heat; P:response to cold; P:response to water deprivation; P:response to high light intensity; P:response to salt stress; P:response to abscisic acid; P:response to hydrogen peroxide; F:inositol 3-alpha-galactosyltransferase activity	8.82	0.00
	TRINITY_DN87967_c2_g2_i6	XP_022867894.1	granule-bound starch synthase		-6.44	0.01
	TRINITY_DN85268_c3_g2_i1	XP_022873704.1	probable galactinol--sucrose galactosyltransferase 5	P:response to oxidative stress; P:response to water deprivation; C:chloroplast; P:response to abscisic acid; F:hydrolase activity; F:galactinol-sucrose galactosyltransferase activity	2.62	0.00
	TRINITY_DN83429_c1_g1_i17	XP_022881255.1	UDP-galactose/UDP-glucose transporter 2	P:carbohydrate transport; C:integral component of Golgi membrane; C:integral component of endoplasmic reticulum membrane; F:3'-phosphoadenosine 5'-phosphosulfate transmembrane transporter activity; P:3'-phospho-5'-adenylyl sulfate transmembrane transport	-8.44	0.01
	TRINITY_DN82035_c0_g2_i2	XP_022855583.1	galactinol synthase 2	P:galactose metabolic process; F:inositol 3-alpha-galactosyltransferase activity	5.73	0.03

* adjusted P-value

Supplementary table S8. Continue

	Seq-ID	GenBank Accession	Description	GO Names	log2 Fold Change	padj*
**Photosynthesis and carbohydrates metabolism**	TRINITY_DN86507_c1_g1_i4	XP_022894654.1	probable starch synthase 4, chloroplastic/amyloplastic isoform X2		7.54	0.05
	TRINITY_DN86507_c1_g1_i14	XP_022894654.1	probable starch synthase 4, chloroplastic/amyloplastic isoform X2		-5.50	0.04
	TRINITY_DN86507_c1_g1_i18	XP_022894654.1	probable starch synthase 4, chloroplastic/amyloplastic isoform X2	F:nucleotide binding; F:motor activity	6.26	0.02
	TRINITY_DN86507_c1_g1_i29	XP_022894654.1	probable starch synthase 4, chloroplastic/amyloplastic isoform X2		7.47	0.01
	TRINITY_DN86507_c1_g1_i32	XP_022894654.1	probable starch synthase 4, chloroplastic/amyloplastic isoform X2		-4.06	0.03
	TRINITY_DN83039_c0_g2_i9	XP_012856806.1	probable plastidic glucose transporter 3	C:endosome; C:trans-Golgi network; P:carbohydrate transport; C:integral component of membrane; F:transmembrane transporter activity; P:transmembrane transport	3.46	0.00
	TRINITY_DN84614_c6_g1_i20	EOY25045.1	UDP-glucose 4-epimerase GEPI48-like	F:UDP-glucose 4-epimerase activity; P:galactose metabolic process	-6.99	0.02
**Photorespiration**	TRINITY_DN87754_c1_g6_i1	XP_022848540.1	glycolate oxidase	F:FMN binding; F:oxidoreductase activity; P:oxidation-reduction process	2.23	0.00
	TRINITY_DN88143_c3_g1_i17	XP_022858270.1	ferredoxin-dependent glutamate synthase, chloroplastic	P:glutamate biosynthetic process; C:plastid; F:glutamate synthase activity; P:ammonia assimilation cycle; P:developmental growth; P:oxidation-reduction process; P:response to ammonium ion	5.73	0.04

* adjusted P-value

Supplementary table S8. Continue

	Seq-ID	GenBank Accession	Description	GO Names	log2 Fold Change	padj*
**Photorespiration**	TRINITY_DN88756_c3_g2_i1	XP_022890356.1	glutamate synthase 1 (NADH), chloroplastic isoform X1	F:iron ion binding; P:glutamate biosynthetic process; F:FMN binding; F:glutamate synthase (NADH) activity; F:flavin adenine dinucleotide binding; F:3 iron, 4 sulfur cluster binding; P:oxidation-reduction process	6.88	0.03
	TRINITY_DN87390_c2_g1_i6	XP_011081684.1	glycine dehydrogenase (decarboxylating), mitochondrial	F:glycine dehydrogenase (decarboxylating) activity; C:mitochondrion; P:glycine catabolic process; P:oxidation-reduction process	7.53	0.02
	TRINITY_DN87390_c2_g1_i8	XP_009620998.1	glycine dehydrogenase (decarboxylating), mitochondrial	F:glycine dehydrogenase (decarboxylating) activity; C:mitochondrion; C:glycine cleavage complex; P:glycine catabolic process; P:oxidation-reduction process	1.42	0.03
	TRINITY_DN87835_c4_g2_i25	XP_022894745.1	serine hydroxymethyltransferase, mitochondrial	F:glycine hydroxymethyltransferase activity; C:nucleus; C:mitochondrion; C:plasma membrane; P:circadian rhythm; F:methyltransferase activity; F:poly(U) RNA binding; P:response to cold; P:response to light stimulus; C:chloroplast P:photorespiration; C:stromule; P:glycine biosynthetic process from serine; C:cytosolic ribosome; F:pyridoxal phosphate binding;	2.84	0.01
	TRINITY_DN86233_c5_g1_i9	XP_022898325.1	serine--glyoxylate aminotransferase	F:serine-pyruvate transaminase activity; C:peroxisome; F:alanine-glyoxylate transaminase activity; P:glycine biosynthetic process, by transamination of glyoxylate	7.51	0.00
	TRINITY_DN86233_c5_g1_i10	XP_022898325.1	serine--glyoxylate aminotransferase	F:serine-pyruvate transaminase activity; C:peroxisome; F:alanine-glyoxylate transaminase activity; P:glycine biosynthetic process, by transamination of glyoxylate	1.57	0.03
	TRINITY_DN87586_c1_g3_i3	XP_022891471.1	glutamate--glyoxylate aminotransferase 2	F:transaminase activity; P:biosynthetic process; F:pyridoxal phosphate binding	6.02	0.02
**Glycolysis process**	TRINITY_DN82902_c2_g2_i19	XP_022894552.1	glucose-6-phosphate isomerase, cytosolic	F:glucose-6-phosphate isomerase activity; C:cytosol; P:gluconeogenesis; P:glycolytic process; P:defense response to fungus, incompatible interaction; P:response to cadmium ion	-7.65	0.02
	TRINITY_DN88793_c1_g1_i1	XP_022847182.1	6-phosphofructo-2-kinase/fructose-2,6-bisphosphatase-like isoform X1	F:6-phosphofructo-2-kinase activity; F:ATP binding; C:cytosol; C:plasma membrane; P:fructose metabolic process; P:fructose 6-phosphate metabolic process; P:fructose 2,6-bisphosphate metabolic process; P:regulation of carbon utilization; P:carbohydrate phosphorylation;	-7.36	0.00

* adjusted P-value

Supplementary table S8. Continue

	Seq-ID	GenBank Accession	Description	GO Names	log2 Fold Change	padj*
**Glycolysis process**	TRINITY_DN79175_c3_g1_i4	XP_022898632.1	fructose-bisphosphatealdolase 1, chloroplastic	F:fructose-bisphosphate aldolase activity; P:glycolytic process	1.28	0.02
	TRINITY_DN88630_c5_g1_i4	XP_022896057.1	fructose-bisphosphatealdolase 1, chloroplastic	F:fructose-bisphosphate aldolase activity; P:glycolytic process	1.44	0.02
	TRINITY_DN81629_c3_g2_i3	XP_022896057.1	fructose-bisphosphatealdolase 1, chloroplastic-like	F:fructose-bisphosphate aldolase activity; P:glycolytic process	1.46	0.02
	TRINITY_DN80186_c0_g2_i1		fructose-bisphosphatealdolase 1, chloroplastic	F:fructose-bisphosphate aldolase activity; P:glycolytic process	1.72	0.02
	TRINITY_DN79175_c3_g1_i3	XP_022876207.1	fructose-bisphosphatealdolase 1, chloroplastic	F:fructose-bisphosphate aldolase activity; P:glycolytic process	6.02	0.03
	TRINITY_DN82501_c0_g2_i3	XP_022876203.1	triosephosphateisomerase, chloroplastic	F:triose-phosphate isomerase activity; C:cytosol; P:gluconeogenesis; P:glycolytic process; C:chloroplast; P:reductive pentose-phosphate cycle; P:glycerol catabolic process; P:glyceraldehyde-3-phosphate biosynthetic process	-7.15	0.00
	TRINITY_DN82501_c0_g1_i4	XP_022862838.1	triosephosphateisomerase, chloroplastic	F:triose-phosphate isomerase activity; C:mitochondrion; C:cytosol; P:gluconeogenesis; P:glycolytic process; P:triglyceride mobilization; C:chloroplast stroma; C:thylakoid; P:chloroplast organization; C:chloroplast envelope; P:glycerol catabolic process; P:multicellular organism reproduction; P:glyceraldehyde-3-phosphate biosynthetic process; C:apoplast; P:primary root development	-7.35	0.00
	TRINITY_DN87428_c7_g4_i3	XP_022890830.1	glyceraldehyde-3-phosphate dehydrogenase A, chloroplastic	P:glucose metabolic process; F:oxidoreductase activity, acting on the aldehyde or oxo group of donors, NAD or NADP as acceptor; F:NADP binding; F:NAD binding; P:oxidation-reduction process	1.43	0.05
	TRINITY_DN87182_c1_g1_i20	XP_022888149.1	NADP-dependent glyceraldehyde-3-phosphate dehydrogenase	F:glyceraldehyde-3-phosphate dehydrogenase (NADP+) (non-phosphorylating) activity; P:oxidation-reduction process	1.87	0.00

* adjusted P-value

Supplementary table S8. Continue

	Seq-ID	GenBank Accession	Description	GO Names	log2 Fold Change	padj*
**Glycolysis process**	TRINITY_DN81702_c0_g1_i4	XP_022879247.1	2,3-bisphosphoglycerate-dependent phosphoglyceratemutase	F:fructose-2,6-bisphosphate 2-phosphatase activity; F:kinase activity; P:phosphorylation; P:dephosphorylation	-7.15	0.03
	TRINITY_DN84176_c2_g1_i6	XP_022846744.1	pyruvate kinase 1, cytosolic-like	F:magnesium ion binding; F:pyruvate kinase activity; P:glycolytic process; F:kinase activity; F:potassium ion binding	-3.91	0.04
	TRINITY_DN82024_c2_g1_i14	XP_022890796.1	pyruvate kinase, cytosolic isozyme	F:magnesium ion binding; F:pyruvate kinase activity; P:glycolytic process; F:kinase activity; F:potassium ion binding	2.33	0.03
**Phenolic compounds and antioxidant enzymes related genes**	TRINITY_DN88365_c5_g2_i3	XP_022880295.1	caffeic acid 3-O-methyltransferase-like	F:O-methyltransferase activity; F:S-adenosylmethionine-dependent methyltransferase activity; P:aromatic compound biosynthetic process; P:methylation; F:protein dimerization activity	-2.98	0.00
	TRINITY_DN88365_c5_g2_i5	XP_022880297.1	caffeic acid 3-O-methyltransferase-like	F:O-methyltransferase activity; P:methylation; F:protein dimerization activity	-8.53	0.04
	TRINITY_DN77564_c0_g1_i3	XP_022882151.1	4-coumarate--CoA ligase 2-like	P:metabolic process; F:ligase activity	6.37	0.02
	TRINITY_DN82421_c1_g3_i2	XP_022852825.1	chalcone synthase	P:flavonoid biosynthetic process; F:naringenin-chalcone synthase activity	7.73	0.03
	TRINITY_DN82493_c0_g1_i6	AHL44984.1	butyrate--CoA ligase AAE11, peroxisomal-like	P:metabolic process; F:ligase activity	4.19	0.00
	TRINITY_DN78706_c0_g4_i3	XP_022851859.1	protein OVEREXPRESSOR OF CATIONIC PEROXIDASE 3	F:DNA binding; C:nucleus	2.99	0.00
	TRINITY_DN82588_c2_g2_i7	XP_022848354.1	monodehydroascorbatereductase	C:cytoplasm; F:monodehydroascorbate reductase (NADH) activity; F:flavin adenine dinucleotide binding; P:oxidation-reduction process	2.53	0.03
	TRINITY_DN86688_c1_g4_i8	XP_022860602.1	glutathione reductase, chloroplastic	F:glutathione-disulfide reductase activity; C:cell; P:glutathione metabolic process; F:electron transfer activity; P:electron transport chain; P:cell redox homeostasis; F:flavin adenine dinucleotide binding; F:NADP binding; P:cellular oxidant detoxification	6.85	0.01

* adjusted P-value

Supplementary table S8. Continue

	Seq-ID	GenBank Accession	Description	GO Names	log2 Fold Change	padj*
Polyamines	TRINITY_DN79434_c1_g2_i3	XP_022844785.1	S-adenosylmethionine decarboxylase proenzyme	F:adenosylmethionine decarboxylase activity; P:S-adenosylmethioninamine biosynthetic process; P:spermine biosynthetic process; P:spermidine biosynthetic process	6.37	0.01
	TRINITY_DN87845_c1_g4_i1	PIN17113.1	thermospermine synthase ACAULIS5-like	F:spermidine synthase activity; P:polyamine biosynthetic process; F:thermospermine synthase activity	8.13	0.03
	TRINITY_DN84464_c3_g1_i3	XP_022851391.1	N-carbamoylputrescineamidase	P:polyamine biosynthetic process; F:N-carbamoylputrescine amidase activity	9.27	0.00
	TRINITY_DN84464_c3_g1_i12	XP_022864874.1	N-carbamoylputrescineamidase	P:putrescine biosynthetic process from arginine; F:N-carbamoylputrescine amidase activity	-8.59	0.00
	TRINITY_DN88108_c1_g1_i6	XP_022881374.1	carbamoyl-phosphate synthase large chain, chloroplastic-like	F:carbamoyl-phosphate synthase (glutamine-hydrolyzing) activity; F:ATP binding; P:glutamine metabolic process; F:transferase activity; F:metal ion binding	-7.37	0.05
	TRINITY_DN85150_c0_g2_i6	XP_022863205.1	zeaxanthinepoxidase, chloroplastic	C:chloroplast; F:zeaxanthin epoxidase(overall) activity; P:abscisic acid biosynthetic process; C:integral component of membrane; P:oxidation-reduction process; F:FAD binding	2.02	0.02
Phytohormones	TRINITY_DN85150_c0_g2_i12	XP_022863205.1	zeaxanthinepoxidase, chloroplastic	C:chloroplast thylakoid membrane; F:zeaxanthin epoxidase(overall) activity; P:abscisic acid biosynthetic process; C:integral component of membrane; C:chloroplast membrane; P:secondary metabolite biosynthetic process; P:oxidation-reduction process; F:FAD binding	2.96	0.00
	TRINITY_DN85150_c0_g2_i13	XP_022863205.1	zeaxanthinepoxidase	C:chloroplast; F:zeaxanthin epoxidase(overall) activity; P:abscisic acid biosynthetic process; C:integral component of membrane; P:oxidation-reduction process; F:FAD binding	6.56	0.01
	TRINITY_DN81842_c2_g1_i4	XP_022863205.1	zeaxanthinepoxidase, chloroplastic	C:chloroplast; F:zeaxanthin epoxidase(overall) activity; P:abscisic acid biosynthetic process; C:membrane; P:oxidation-reduction process;	2.74	0.02
	TRINITY_DN81566_c0_g1_i14	XP_022887256.1	cytokininriboside 5'-monophosphate phosphoribohydrolase LOG3	C:nucleus; P:cytokinin biosynthetic process; F:hydrolase activity	4.78	0.01
	TRINITY_DN77656_c0_g1_i3	XP_022894450.1	cytokininriboside 5'-monophosphate phosphoribohydrolase LOG1	C:nucleus; P:cytokinin biosynthetic process; F:hydrolase activity	6.60	0.04

* adjusted P-value

Supplementary table s8. Continue

	Seq-ID	GenBank Accession	Description	GO Names	log2 Fold Change	padj*
Flowering control genes	TRINITY_DN86487_c3_g1_i1	XP_022855576.1	MADS-box transcription factor 23-like	F:RNA polymerase II regulatory region sequence-specific DNA binding; F:DNA-binding transcription factor activity; C:nucleus; P:positive regulation of transcription by RNA polymerase II; F:protein dimerization	-8.51	0.02
	TRINITY_DN87170_c1_g1_i11	XP_022860886.1	flowering time control protein FCA-like	F:RNA binding	-5.16	0.05
	TRINITY_DN88181_c1_g2_i11	XP_022876625.1	flowering locus K homology domain	F:RNA binding; C:nucleus; C:cytoplasm; P:positive regulation of flower development	-6.19	0.04
	TRINITY_DN88181_c1_g2_i12	XP_022876624.1	flowering locus K homology domain	F:RNA binding; C:nucleus; C:cytoplasm; P:positive regulation of flower development; C:membrane	8.84	0.00
	TRINITY_DN83713_c0_g1_i11	XP_022889081.1	flowering locus K homology domain-like	F:RNA binding	-6.39	0.04
	TRINITY_DN79407_c1_g2_i8	XP_022850620.1	protein UPSTREAM OF FLC isoform X1	-	-8.85	0.05

* adjusted P-value
