# Supplementary material for: Differential expression of genes in olive leaves and buds of ON- versus OFF-crop trees
Source: Sci Rep. 2020 Sep 25;10:15762. doi: 10.1038/s41598-020-72895-7 (PMC7519672; doi:10.1038/s41598-020-72895-7)
Supplement: Supplementary file 8 — Supplementary Table 8. [file 41598_2020_72895_MOESM8_ESM.pdf]

Alternate bearing in olive: Differential expression of genes in leaves and buds of ON- *versus* OFF-crop trees

Ebrahim Dastkar[1], Ali Soleimani[1*], Hossein Jafary[2], Juan de Dios Alche[3], Abbas Bahari[4], Mehrshad Zeinalabedini[5] and Seyed Alireza Salami[6]

Supplementary table S8. List of primers used for validating of the RNA-Seq results

Seq-ID	GenBank Accession	Description	Forward primer (5`-3`)	Reverse primer (5`-3`)	product size (bp)	product Tm (°C)
TRINITY_DN81593_c2_g2_i7	XM_023022187	Peroxidase 42-like	CCGCTCAAGAACAACATT	AACTGGAAGAAGAGATGGT	94	83
TRINITY_DN78486_c0_g1_i14	XM_023032593	Superoxide dismutase (Fe), chloroplastic-like	TTCCGCTGTTATATGTAG	CTTATGTAGACAACCTCAA	93	84
TRINITY_DN78706_c0_g4_i3	XM_022996091	Protein overexpressor of cationic peroxidase 3 isoform X3	GGTGAAGACGATGATATAAC	CCTACATTAGAAGCAAGTG	107	84.5
TRINITY_DN82507_c0_g2_i3	XM_023027526	Rubisco large subunit-binding protein subunit alpha	GGAGATTGATAGAGGATACA	CTTCTGGTCCGTTACTAA	100	83.5
Actin	AF545569.1	(Dündar *et al.*, 2013)	GAATTGCCAGATGGACAGGT	GAACCACCACTGAGGACGAT	188	87
